# The global burden of smoking-related prostate cancer from 1990 to 2021 and projections to 2031

**DOI:** 10.18332/tid/204300

**Published:** 2025-05-16

**Authors:** Qian Cai, Bohao Liu, Chen Zou, Huabin Su, Xiao Zhao, Fang Jia, Xiaoyang Li, Weian Zhu, Yun Luo

**Affiliations:** 1 Department of Urology, The Third Affiliated Hospital of Sun Yat-sen University, Guangzhou, China; 2 Department of Urology, Affiliated Hospital of Yunnan University, Kunming, China; 3 Department of Neurosurgery, The Third Affiliated Hospital of Sun Yatsen University, Guangzhou, China; 4 Guangdong Provincial Clinical Research Center for Urological Diseases, Guangzhou, China; 5 Department of Urology, The First People's Hospital of Kashgar, Kashgar, China

**Keywords:** prostate cancer, smoking, global burden of disease, sociodemographic index, projections

## Abstract

**INTRODUCTION:**

Smoking is a significant risk factor for prostate cancer (PCa), a major health threat for aging males globally. This study evaluates the worldwide burden of smoking-related PCa from 1990 to 2021 and projects trends to 2031.

**METHODS:**

Using Global Burden of Disease (GBD) 2021 data, we analyzed age-standardized rates (ASRs) and estimated annual percentage changes for mortality, years lived with disability (YLDs), years of life lost (YLLs), and disability-adjusted life years (DALYs) across different age groups, sociodemographic index (SDI) levels, regions, and countries, employing hierarchical clustering and autoregressive integrated moving average (ARIMA) modeling.

**RESULTS:**

From 1990 to 2021, global smoking-related prostate cancer burden declined, with annual reductions in ASRs for mortality, YLLs, and DALYs, while YLDs initially increased before declining. Age-specific analysis revealed the highest ASRs for mortality, YLLs, and DALYs in the 90–94 years age group, whereas YLDs peaked at 70–74 years of age. SDI regions exhibited elevated ASRs but the most pronounced declines, and were the only areas with negative YLD trends. The disparity in disability rates between high and low SDI countries diminished from 7.33 (95% CI: 6.04–8.63) in 1990 to 3.78 (95% CI: 2.64–4.92) in 2021, and the concentration index decreased from 0.34 (95% CI: 0.28–0.39) to 0.15 (95% CI: 0.10–0.20). The ARIMA models predict that DALYs will decrease from 3.215 (95% CI: 3.169–3.26) in 2022 to 2.69 (95% CI: 2.159–3.221) in 2031, YLLS will decrease from 2.827 (95% CI: 2.787–2.866) to 2.336 (95% CI: 1.855–2.817), YLDs and deaths will stabilize in a gradually decreasing trend.

**CONCLUSIONS:**

Despite improved global equity in smoking-related PCa burden, targeted interventions for elderly populations, enhanced tobacco control policies, and region-specific prevention strategies remain essential to further reduce this preventable disease burden worldwide.

## INTRODUCTION

Prostate cancer significantly impacts male mortality worldwide, with GLOBOCAN 2020 reporting 1.41 million new cases and 375000 deaths globally^1^. In high-sociodemographic index (SDI) countries such as North America and Europe, age-standardized incidence rates exceeded 60 per 100000, while in low-SDI regions like Africa and parts of Asia, these rates are 10–20 per 100000 ^2^. Smoking remains a crucial risk factor for prostate cancer. Meta-analyses show that current smokers face a 24% higher mortality risk compared to never smokers (HR=1.24, 95% CI: 1.18–1.31)^3^. Paradoxically, current smokers show lower incidence rates than former smokers, possibly due to reduced prostate-specific antigen (PSA) screening^4^.

Furthermore, the correlation between smoking and increased mortality risk from prostate cancer has been firmly established. Smokers demonstrate a significantly elevated mortality risk compared to nonsmokers^5^. This risk is even more pronounced among obese smokers, who exhibit lower cancer survival rates^5,6^. The detrimental effects of smoking may be related to reduced PSA testing, leading to later stage cancer diagnoses^7^. According to the 2022 Global Cancer Statistics, the annual number of prostate cancer deaths attributable to smoking is increasing, particularly in low-income countries and regions^8^. Despite declining smoking rates in some high-income countries, smoking remains a substantial contributor to the prostate cancer burden elsewhere.

Recent advancements in prostate cancer diagnosis and treatment are notable. Modern medical technologies have significantly enhanced early detection capabilities, mainly through the integrated use of magnetic resonance imaging (MRI), Prostate-Specific Membrane Antigen Positron Emission Tomography (PSMA-PET) CT/MRI, and targeted biopsy, thereby increasing the detection rate of clinically significant prostate cancer^9^. In addition, treatment strategies for prostate cancer are constantly improving, including hormone endocrine therapy, radiation therapy, and surgical intervention aimed at enhancing patients’ survival rates and quality of life. However, the mortality rate of prostate cancer remains high in some regions, especially in countries and areas with limited medical resources^10^.

Previous research on smoking-related prostate cancer has mainly focused on individual countries or regions, with limited studies adopting a global perspective. Recent updates to the Global Burden of Disease (GBD) data highlight some differences from earlier research findings^8^. Based on the GBD 2021 database, this retrospective study systematically assesses the evolution of the global smoking-related prostate cancer burden from 1990 to 2021^11^. Given the observational nature of the GBD 2021 dataset, this study aims to quantify associations between smoking and PCa burden, recognizing that these relationships do not imply causality, and warrant further investigation. This multidimensional analysis helps to understand disease burden patterns and provides key evidence for targeted prevention strategies. In addition, the ARIMA models provide valuable insights for predicting public health trends, innovatively integrating time, space, and demographic dimensions to provide a comprehensive perspective on the relationship between smoking and prostate cancer.

## METHODS

### Data sources and definitions

This study is a retrospective ecological analysis based on secondary data from the GBD 2021. The smokingrelated prostate cancer data analyzed in this study were obtained from GBD 2021, which are derived from censuses, household surveys, civil registration and vital statistics, disease registries, health service use, satellite imaging, and disease notifications, and provide the burden of 371 diseases and injuries in 21 GBD regions and 204 countries and territories over the period 1990 to 2021 (included 32 time points) from updated epidemiological data estimates^12-15^. The GBD 2021 data, derived from diverse observational sources, enable the estimation of disease burden trends and associations with risk factors such as smoking; however, these data do not permit causal inference. All these data are freely available through the Global Health Data Exchange and detailed information on the data is available in previous reports^16,17^. Notably, the uncertainty intervals (UIs) reported in the results represent the 95% uncertainty intervals derived from the GBD methodology, which uses a simulation approach to calculate the 95% UIs, specifically Monte Carlo simulation techniques. The proportion of prostate cancer burden attributable to smoking was calculated using population attributable fraction (PAF). PAF quantifies the extent of impact, representing the fraction of disease outcomes that could potentially be avoided if the risk factor were eliminated from the population. The attributable burden is derived by multiplying the relevant PAF by the total prostate cancer burden for each age, sex, location, and year group^18^.

Within the context of the GBD, prostate cancer is defined as malignant neoplasms of the prostate, the International Classification of Diseases 10th Revision (ICD-10) code C61^19^. And smoking is defined as current daily or occasional use of any smoked tobacco product^20^.

### Disability-adjusted life years (DALYs)

DALYs are the total healthy life years lost from morbidity to mortality and are the sum of years of life lost (YLLs) and years lived with disability (YLDs).

### Sociodemographic index (SDI)

The SDI is a composite indicator introduced in 2015 by the Institute for Health Metrics and Evaluation (IHME) that emphasizes the interconnections between social development and population health outcomes. Values range from 0 to 1, with higher values representing more significant development. Two hundred four countries and territories are categorized into five SDI regions 2021: low, low-middle, middle, high-middle, and high^21^.

### Estimated annual percentage change and percentage change (EAPC)

The EAPC is a statistical indicator used to quantify the average annual change rate of health-related indicators in a specific time period, such as incidence rate, mortality, and disability-adjusted life years^22^. The EAPC is widely used in GBD research to track trends in metrics such as prevalence and incidence over specific periods^23^.

### Cross-country inequality analysis

Two standardized indicators, the slope index of absolute gradient inequality and the concentration index of relative gradient inequality, are used to quantify the distributional inequality of the burden across countries. The slope index of inequality is computed by regressing national rates of DALYs on the relative positional scales associated with the SDI for all population age groups.

### Statistical analysis

We analyzed the corresponding age-standardized rates (ASRs) of smoking-related prostate cancer deaths, YLDs, YLLs, and DALYs globally from 1990 to 2021, to investigate the dynamics of the disease burden. The results were then stratified by subtype, including age group, SDI region, GBD region, and country. The results of EAPC were analyzed by hierarchical clustering, and the 21 GBD regions were divided into four groups (significant increase, minor increase, remained stable or minor decrease, and significant decrease) based on the degree of fit to the expected curve. Hierarchical clustering analysis is an algorithm used to partition a set of data into multiple distinct clusters. Unlike other clustering algorithms, such as K-means, hierarchical clustering analysis does not require the number of clusters to be specified in advance. Instead, it merges similar samples into a cluster based on the distance between each pair of samples, until all samples are merged^24^. In addition, a cross-country inequality analysis based on the standard health equity analysis methodology recommended by the World Health Organization (WHO) was conducted to determine the extent of inequality in the burden of smoking-related prostate cancer associated with sociodemographic development level in each country, and its trend over time. Finally, we used the autoregressive integrated moving average (ARIMA) models to predict the burden of smoking-related prostate cancer in the following decade. Using the fitted ARIMA models, we forecast the prevalence of smoking-related prostate cancer from 2022 to 2031. This projection was presented with a 95% confidence interval to account for potential variability in the predictions, providing a range within which the actual prevalence may fall. The study began by compiling a detailed time series dataset covering the prevalence of prostate cancer from 1990 to 2021^24^. Subsequently, the model selection was performed using the *auto.arima()* function from the forecast package, and all the observed and fitted values showed good consistency. Then, the models underwent testing and diagnosis through a white-noise test of residuals, specifically utilizing the Ljung-Box test. The outcome of passing the white-noise test (p>0.05) indicated the model’s suitability for the selected time series. Following the diagnostic confirmation, the ARIMA models were employed to forecast the prevalence of prostate cancer from 2022 to 2031. Parallel projections were conducted to estimate DALYs, YLDs, and YLLs for prostate cancer over the same period, providing a comprehensive outlook on the burden of disease. All statistical analyses were conducted using RStudio (version 4.3.0), with data visualization created using the *ggplot2* package^25-27^. The level of statistical significance was set at p<0.05. All tests were two-tailed.

## RESULTS

### Temporal trends of smoking-related prostate cancer burden by country from 1990 to 2021

**Figure 1:**
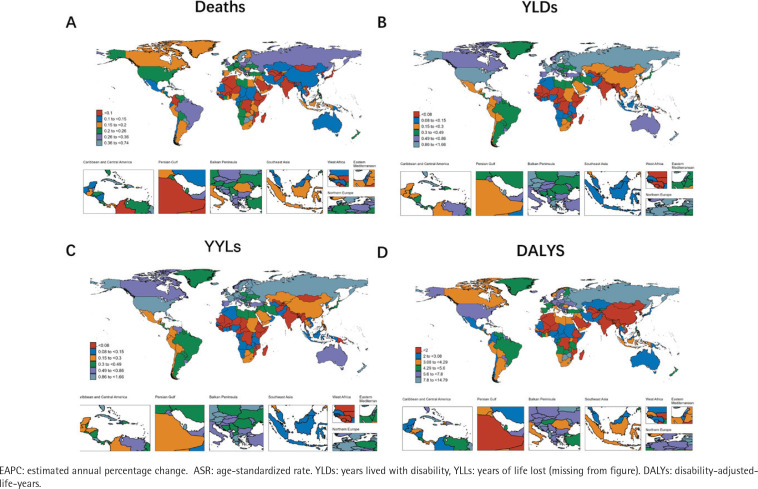
The trend of smoking-related prostate cancer-related ASRs of deaths, YLDs, YLLs and DALYs for different countries between 1990 and 2021

We analyzed the burden of disease of smoking-related prostate cancer at the national level. For the ASR of death, the country of Georgia had the most significant upward slope with an EAPC of 5.36 (95% UI: 4.52– 6.20), while Australia, Ireland, Canada, and Spain had the largest decreases with -4.55 (95% UI: -5.03 – -4.07), -4.53 (95% UI: -4.95 – -4.10), -4.47 (95% UI: -4.76 – -4.18) and -4.26 (95% UI: -4.47 – -4.06), respectively. For YLDs, Georgia remained the most burdened country at 5.25 (95% UI: 4.61 – 5.88), while among the most burdened countries, in addition to Canada (EAPC= -3.26; 95% UI: -3.63 – -2.90), there was Madagascar (EAPC= -2.66; 95% UI: -3.13 – -2.19), USA (EAPC= -2.34; 95% UI: -2.49 – -2.19), and Tajikistan (EAPC= -2.09; 95% UI: -2.30 – -1.88) among the top countries. Consistent with the results of the other analyses by subtype, the highest and lowest EAPC values for YLDs and DALYs were localized in the same countries as the results for deaths, i.e. Georgia, Australia, Ireland, Canada, and Spain (Figure 1; and Supplementary file Table S1).

### Exposure to smoking and temporal trend for smoking-related prostate cancer burden from 1990 to 2021

In 2021, the global deaths, YLDs, YLLs, and DALYs for prostate cancer attributable to smoking were estimated at 3.00 % (95% UI: 1.41–4.92), 4.04% (95% UI: 1.91–6.42 %), 3.39% (95% UI: 1.61–5.46) and 3.46% (95% UI: 1.64–5.56) of the total deaths, YLLs, and DALYs, respectively. We analyzed trends in the burden of disease for smoking-related prostate cancer from 1990 to 2021. As shown in Figure 1, the ASRs for deaths, YLDs, YLLs, and DALYs declined annually. YLDs, on the other hand, showed an upward trend until 2009 and a fluctuating but generally decreasing trend after that. Globally, the ASR for deaths decreased from 0.279 (95% UI: 0.130–0.454) in 1990 to 0.155 (95% UI: 0.070–0.258) in 2021, showing a significant downward trend (EAPC= -2.19: 95% UI: -2.29 – -2.09). The ASR of YLDs decreased from 0.506 (95% UI: 0.222–0.841) to 0.391 (95% UI: 0.169–0.665). Similarly, the ASR of YLLs decreased from 5.220 (95% UI: 2.444–8.291) in 1990 to 2.871 (95% UI: 1.328–4.667) in 2021. Similarly, the ASR of DALYs has decreased (EAPC= -2.07; 95% UI: -2.16 – -1.99) (Table 1; and Supplementary file Figure S1).

**Table:** Table 1. The global EAPC of smoking-related prostate cancer-related ASRs of deaths, YLDs, YLLs and DALYs between 1990 and 2021

*Measure*	*Age*	*EAPC (95% CI)*
Deaths	All ages	-0.67 (-0.79 – -0.56)
Deaths	Age-standardized	-2.19 (-2.29 – -2.09)
DALYs	All ages	-0.71 (-0.81 – -0.60)
DALYs	Age-standardized	-2.07 (-2.16 – -1.99)
YLDs	All ages	0.26 (0.17–0.35)
YLDs	Age-standardized	-1.08 (-1.21 – -0.95)
YLLs	All ages	-0.82 (-0.93 – -0.71)
YLLs	Age-standardized	-2.19 (-2.28 – -2.10)

EAPC: estimated annual percentage change. ASR: age-standardized rate. YLDs: years lived with disability. YLLs: years of life lost. DALYs: disability-adjusted-life-years.

### Disease burden of smoking-related prostate cancer by age in 2021

**Figure 2:**
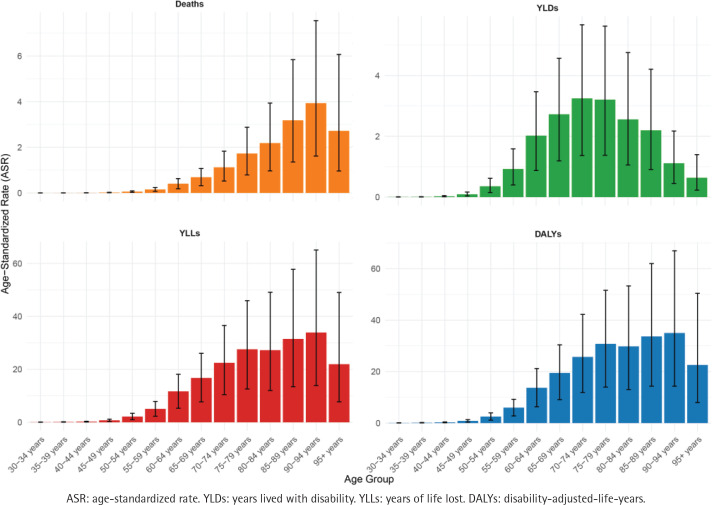
The smoking-related prostate cancer-related ASRs of deaths, YLDs, YLLs and DALYs for different age groups in 2021

We examined the 2021 disease burden of smoking-related prostate cancer from an age perspective. ASRs for deaths, YLDs, YLLs, and DALYs generally increased with age, peaking in older groups before declining. The highest ASR burden regarding deaths, YLLs, and DALYs were all clustered in the 90–94 years age group, with ASR values of 3.934 (95% UI: 1.614–7.550), 33.912 (95% UI: 13.913–65.077) and 35.027 (95% UI: 14.332–66.982), respectively. While the 70–74 years age group had the highest ASR for YLDs with 3.251 (95% UI: 1.368–5.671) (Figure 2; and Supplementary file Table S2).

### Temporal trend for smoking-related prostate cancer burden by SDI from 1990 to 2021

Trends in the smoking-related prostate cancer burden were analyzed by SDI subtype. The results showed higher SDI levels were associated with elevated ASR, with the most pronounced declines observed in high-SDI regions from 1990 to 2021. Except for YLDs, the ASR for the other three indicators in each SDI region showed a steady downward trend. The EAPC for mortality was -3.17 (95% UI: -3.31 – -3.02) for high-SDI regions, -1.43 (95% UI: -1.59 – -1.27) for high-middle SDI regions, -1.06 (95% UI: -1.17 – -0.94) for middle-SDI regions, -0.31 (95% UI: -0.39 – -0.23) for low-middle SDI regions, and -0.66 (95% UI: -0.78 – -0.55) for low-SDI regions. The EAPC values for ASRs of YLLs and DALYs were similar to those of deaths, with the highest negative EAPC values found in the high-SDI region, at -3.22 (95% UI: -3.35 – -3.09) and -2.91 (95% UI: -3.02 – -2.80), respectively. The low-middle region had the least negative EAPC values of -0.38 (95% UI: -0.44 – -0.31) and -0.32 (95% UI: -0.38 – -0.25), respectively. YLDs showed positive values, except in high-SDI regions (EAPC= -1.33, 95% UI: -1.49 – -1.18), which is negative (Supplementary file Figure S2, S3 and Table S3).

**Figure 3:**
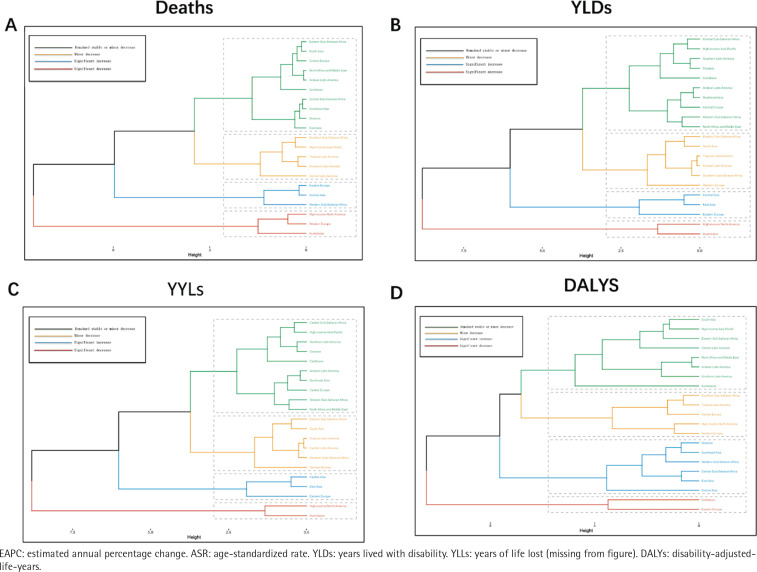
Results of cluster analysis based on the EAPC values of smoking-related prostate cancer-related ASRs for deaths, YLDs, YLLs and DALYs from 1990 to 2021. The hierarchical clustering method grouped regions based on their EAPC values for these four metrics, revealing distinct patterns of change in smokingrelated prostate cancer burden. 21 GBD regions are characterized into four groups: significant increase, minor increase, remained stable or minor decrease, and significant decrease

### Temporal trends of smoking-related prostate cancer burden by GBD region from 1990 to 2021

Disease burden metrics (ASRs for deaths, YLDs, YLLs, and DALYs) were analyzed for different GBD regions. Stratified cluster analyses were also performed to better observe changes in the burden of smoking-related prostate cancer from 1990 to 2021 in these GBD regions.

Based on the EAPC values of smoking related prostate cancer from 1990 to 2021, the K-means clustering algorithm was used to classify the changing trends of Deaths, DALYs, YLDs, and YLLs in 21 GBD regions, and compared with the hierarchical clustering method. K-means divided into four categories: significant increase, slight increase, basic stability, slight decrease, and significant decrease. Overall, the K-means clustering demonstrated a high level of consistency with the hierarchical method in identifying global patterns of burden change. Regarding YLDs and YLLs, High-income North America and Australasia showed a significant decrease, while Eastern Europe, Central Asia, and East Asia exhibited a significant increase. In contrast, deaths and DALYs presented slightly different results. High-income North America, Australasia, and Western Europe experienced a significant decrease in deaths, while Eastern Europe, Central Asia, and Western Sub-Saharan Africa exhibited a significant increase. The Caribbean and Eastern Europe showed a significant decrease in DALYs, while Oceania, Southeast Asia, Central Sub-Saharan Africa, Western Sub-Saharan Africa, East Asia, and Central Asia demonstrated a significant increase (Figure 3).

For deaths, Eastern Sub-Saharan Africa, South Asia, Eastern Europe, high-income North America, and Western Europe closely followed expected trends over the study period. Western Sub-Saharan Africa, Central Latin America, Southern Sub-Saharan Africa, and Tropical Latin America exceeded expectations. Nevertheless, East Asia, Southern Asia, and high-income Asia Pacific showed lower values than expected, while the remaining regions fluctuated. For YLDs, most regions followed the projected trend line, with only minor variations. Lower SDI regions closely followed the predicted trend line, with only minor differences between high and low. Western Sub-Saharan Africa, Eastern Sub-Saharan Africa, South Asia, Central Sub-Saharan Africa, North Africa, and the Middle East are equal to the predicted curves, almost coinciding with each other, and the distribution of higher SDI regions is polarized and more evenly distributed above and below the predicted trend. On the other hand, the results for YLLs and DALYs are similar to deaths, with almost identical distributions between regions and prediction lines (Supplementary file Figure S4 and Table S4).

### Cross-country inequalities in smoking-related prostate cancer from 1990 to 2021

Significant SDI-related inequalities were observed in the high burden of smoking-related prostate cancer disease, demonstrating a disproportionate increase. The inequality slope index showed that the difference in the rate of DALYs between countries with the highest and lowest SDI was 7.33 (95% CI: 6.04–8.63) in 1990, and the value becomes smaller by 2021, with a difference of 3.78 (95% CI: 2.64–4.92). In addition, the concentration index was 0.34 (95% CI: 0.28–0.39) in 1990 and 0.15 (95% CI: 0.10–0.20) in 2021 (Supplementary file Figure S5).

### Predicted results of disease burden for smoking-related prostate cancer from 2022 to 2031

The ARIMA model predictions show a steady downward trend in the burden of smoking-related prostate cancer. Over the next decade or so, the values of YLLs and DALYs show a significant decline, while the values of deaths and YLDs show a stable but slightly decreasing trend. In other words, the absolute values of the slopes of YLLs and DALYs are more significant than those of the slopes of deaths and YLDs (Supplementary file Figure S6 and Tables S5 and S6).

## DISCUSSION

Using the GBD 2021 database, this study systematically examined the association between smoking, a significant lifestyle factor, and the disease burden of prostate cancer. A notable strength of this research lies in its utilization of long-term observational data spanning 31 years (1990–2021), covering regions with different levels of social development, and assessing disease burden through YLDs, YLLs, and DALYs. The age-standardized mortality rate for smoking-related prostate cancer globally decreased from 0.279 (95% UI: 0.130–0.454) in 1990 to 0.155 (95% UI: 0.070–0.258) in 2021, with an average annual decline of 2.19% (95% UI: -2.29 – -2.09). While this downward trend is evident overall, the high-SDI region has the most significant decline (EAPC=-3.17; 95% UI: 3.31 – -3.02), while the low SDI and medium SDI regions have the smallest decline (EAPC=0.31; 95% UI: 0.39 – -0.23). Furthermore, the cross-national inequality index decreased from 7.33 (95% CI: 6.04–8.63) in 1990 to 3.78 (95% CI: 2.64–4.92) in 2021. Age-stratified analysis showed that older populations, particularly those aged 90–94 years, bore the highest burden of deaths, YLLs, and DALYs, whereas YLDs peak in individuals aged 70– 74 years. Due to insufficient medical infrastructure, weak tobacco control policies, and socio-economic challenges, the burden of prostate cancer in low SDI areas is slowly decreasing. In contrast, high-SDI regions have achieved significant declines due to superior healthcare systems, strategic resource allocation, and effective smoking control measures.

From 1990 to 2021, prostate cancer burden trends differed markedly across regions. Mortality rates exceeded projections in regions such as Western Sub-Saharan Africa and Central Latin America. In contrast, high-income countries in Asia Pacific, East Asia, and South Asia reported lower-than-expected unemployment rates. The mortality rate in countries such as Georgia has increased, which may be related to rising smoking rates and weak public health policies. In contrast, countries such as Australia, Ireland, and Spain have achieved a decline through strong anti-smoking initiatives and health education programs. Although the concentration index dropped to 0.15 in 2021, indicating a reduction in inequality, significant differences still exist, with a particularly severe impact on low SDI groups. It is worth noting that as the most populous countries in the world, the SDI of China and India has improved over time, with China’s index surpassing India’s. Both countries have made significant strides in controlling the disease burden since 1990, with China exhibiting a more favorable disease burden concentration index and superior health indicators among affluent groups in 2021 compared to India. This progress can be attributed to the effective implementation of public health strategies and the positive impact of socio-economic development on health outcomes. The ARIMA models predict that the global burden of prostate cancer will continue to decline, and there is a need to strengthen public health interventions, including tobacco control, healthcare accessibility, and awareness campaigns, especially in areas with insufficient medical resources.

Prostate cancer is an increasingly important global public health problem, which is characterized by an increase in incidence rate, especially among young people aged 40–49 years. In contrast, the incidence rate in the elderly population is decreasing, which may be due to the progress of diagnostic methods and the impact of PSA screening^28^. Notable racial disparities exist, with African American men exhibiting higher incidence and mortality rates compared to Caucasian men, the influence of socio-economic determinants^29^. While the mortality rate in high-income countries has generally decreased, in low- and middle-income countries, the mortality rate remains high. Especially in the United States, age-adjusted mortality rates have stabilized, and despite advances in medicine^30^, there is a significant correlation between lifestyle factors such as smoking and an increased risk of prostate cancer mortality. Consequently, implementing public health measures to reduce tobacco consumption is crucial^31^. The latest advances in drug therapy, including enzalutamide and abiraterone, can improve the survival rate of patients with advanced prostate cancer. However, there is still a risk between aggressive treatment strategies and overtreatment.

Cessation of smoking can substantially reduce the risk of prostate cancer^5^. Smoking-induced chronic inflammation and immunosuppression contribute to tumor progression and impair immunotherapy responses^6^. Smoking negatively impacts the prognosis of prostate cancer, leading to higher mortality and recurrence rates among smokers. Additionally, smoking increases the burden of urinary system cancer through various molecular and clinical pathways. A better understanding of the mechanisms by which smoking facilitates cancer development and progression offers potential therapeutic opportunities^32^. Carcinogens introduced by smoking, such as polycyclic aromatic hydrocarbons and nitrosamines, can cause DNA damage and mutations in prostate cells. These mutations may lead to the inactivation of the p53 tumor suppressor gene, potentially resulting in more aggressive tumor phenotypes and accelerating tumor progression^31^. Smoking also promotes systemic and local inflammation, particularly in prostate tissue, which may contribute to chronic prostatitis. This, in turn, can alter the tumor microenvironment, facilitating tumor growth, invasion, and metastasis^7^. Furthermore, smoking may influence circulating hormone levels, including increasing estrogen metabolites and altering androgen metabolism. These changes could accelerate the progression of prostate cancer, especially more aggressive variants^4^. Additionally, the immunosuppressive effects of smoking may impair the body’s immune response against cancer cells, potentially reducing the efficacy of immunotherapy. This is particularly relevant in advanced PCa, where the importance of immunotherapy is increasing, and smoking may diminish its effectiveness by suppressing the immune system. The adverse outcomes of smoking on prostate cancer may also be associated with biological changes, such as oxidative stress and alterations in detoxification pathways, which could enhance PCa cell resistance to chemotherapy and androgen deprivation therapy (ADT)^5,6^. Therefore, there is an urgent need for smoking cessation interventions and public health policies to decrease the incidence and mortality rates of smoking-related prostate cancer^33^.

### Limitations

This study has several important limitations that should be acknowledged. Firstly, the detection bias may be due to the low participation rate of smokers in PSA screening, especially in areas with limited opportunities for routine testing, which may underestimate the incidence rate of PCa and delay the diagnosis of invasive diseases. Secondly, the summary nature of GBD data excludes stratification based on PCa molecular subtypes, inertness, and invasiveness, or local and metastatic clinical stages, which limits the understanding of the different effects of smoking on disease progression or treatment outcomes. Thirdly, although our analysis identified population-level associations, observational design inherently limits causal inference, and linear trend assumptions (such as EAPC) may oversimplify complex temporal dynamics affected by sudden policy shifts. Additionally, although GBD has been adjusted, residual confusion still exists because self-reported smoking data are susceptible to social expectation bias, and emerging risks such as secondhand smoke and e-cigarettes remain unexplained. Finally, although the ARIMA models are robust for short-term forecasting, they rely on historical trends and cannot fully capture future behavior or changes in the healthcare system, amplifying the uncertainty of long-term forecasting. These limitations highlight the necessity for future research to integrate clinical registration, causal inference methods, and dynamic modeling to improve risk assessment and provide information for targeted interventions.

## CONCLUSIONS

The global burden of smoking-related prostate cancer displayed a complex pattern of change between 1990 and 2021. High SDI regions saw a significant drop in ASRs due to better healthcare and public health efforts, while low SDI areas experienced only slight decreases or even increases. This highlights the need for targeted measures to address health disparities. Smoking negatively affects prostate cancer’s occurrence and progression, so we recommend strengthening smoking cessation programs and health policies. The ARIMA models support consistent public health efforts, suggesting a continued decline in the global disease burden. Early screening and robust tobacco control are crucial for reducing prostate cancer in aging males.

## Supplementary Material



## Data Availability

The data (https://ghdx.healthdata.org/gbd-2021/sources) that support the findings of this study are available from the corresponding author upon reasonable request. IICD-10 code C61 (https://www.healthdata.org/research-analysis/diseases-injuries-risks/factsheets/2021-prostate-cancer-level-3-disease). Smoking refers to the current daily or occasional use of any smoking tobacco products (https://www.healthdata.org/research-analysis/diseases-injuries-risks/factsheets/2021-smoking-level-3-risk).
