# Supplementary material for: The global burden of smoking-related prostate cancer from 1990 to 2021 and projections to 2031
Source: Tob Induc Dis. 2025 May 16;23:10.18332/tid/204300. doi: 10.18332/tid/204300 (PMC12083077; doi:10.18332/tid/204300)
Supplement: Supplementary file 1 [file TID-23-67-s1.pdf]

Supplementary Materials

Figure legend

Figure S1. The trend of smoking-related prostate cancer-related ASRs of deaths, YLDs, YLLs and DALYs between 1990 and 2021.

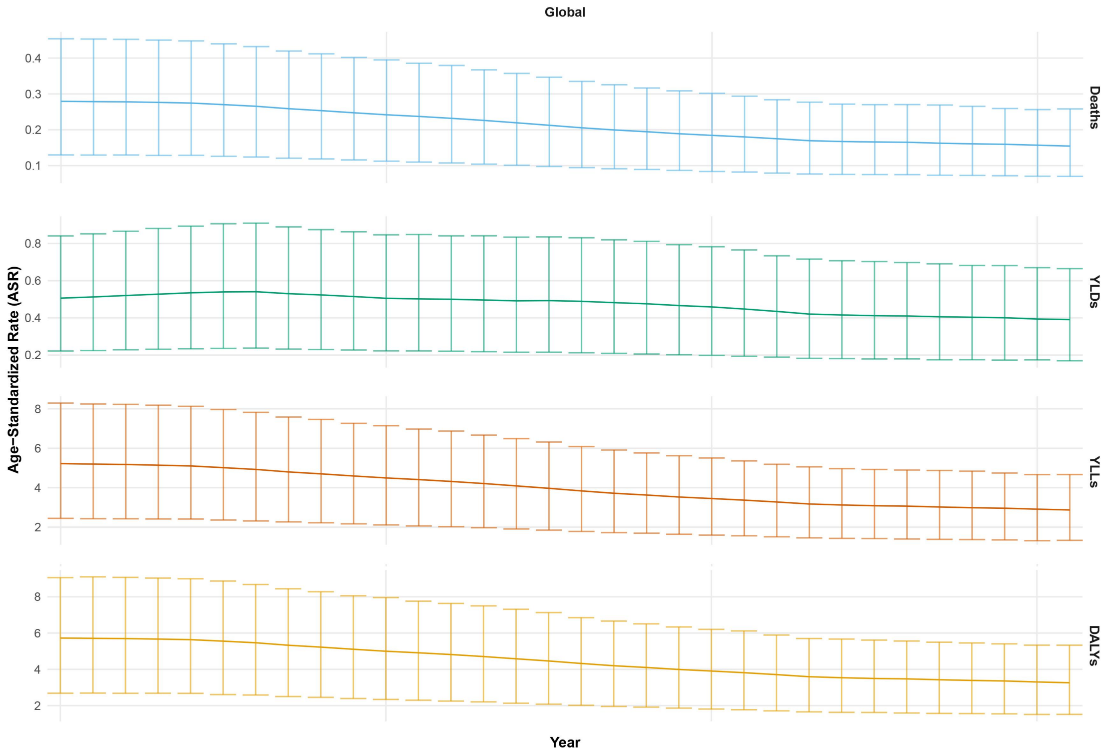

1

ASR: age-standardized rate, YLDs: Years Lived with Disability, YLLs: Years of Life Lost, DALYs: disability-adjusted-life-years.

Figure S2. The EAPC of smoking-related prostate cancer-related ASRs of deaths, YLDs, YLLs and DALYs for different SDI regions between 1990 and 2021.

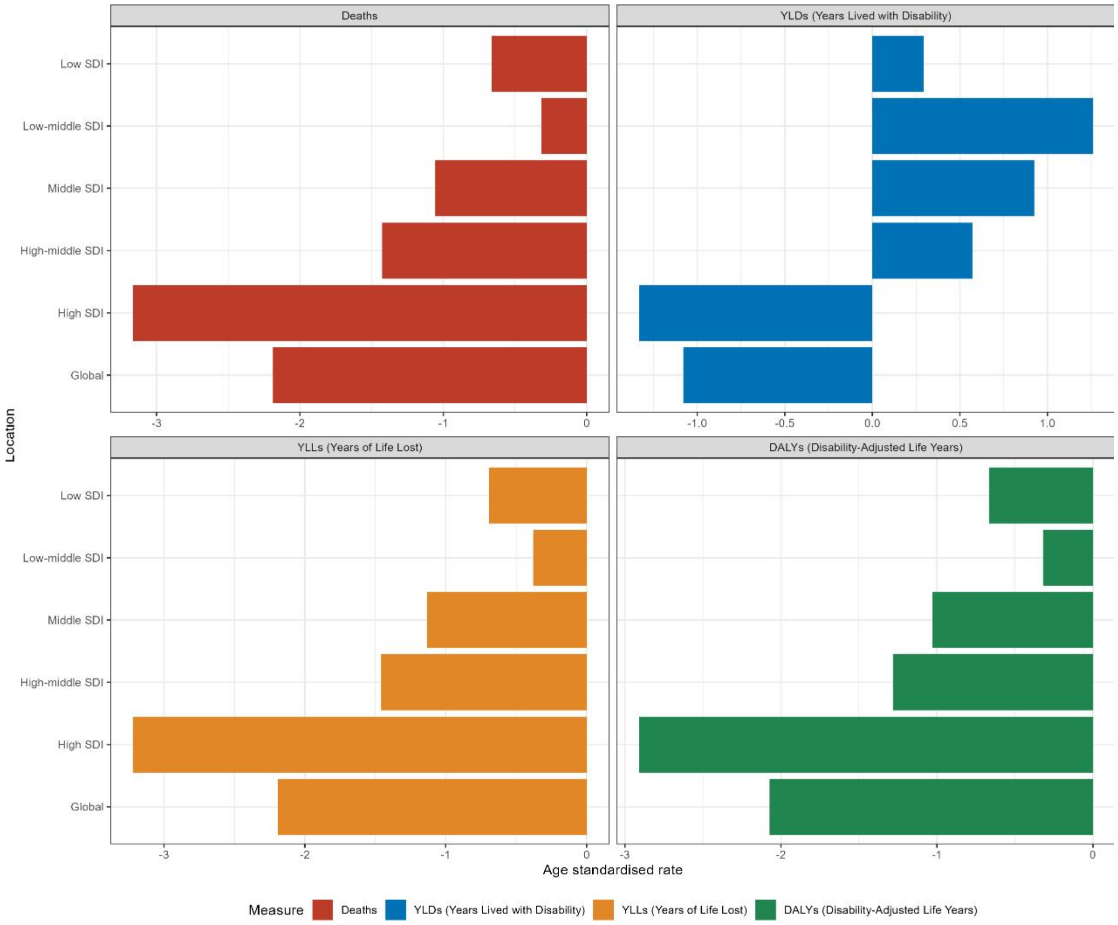

2

EAPC: estimated annual percentage change, ASR: age-standardized rate, YLDs: Years Lived with Disability, YLLs: Years of Life Lost, DALYs: disability-adjusted-life-years.

Figure S3. The trend of smoking-related prostate cancer-related ASRs of deaths, YLDs, YLLs and DALYs for different SDI regions between 1990 and 2021.

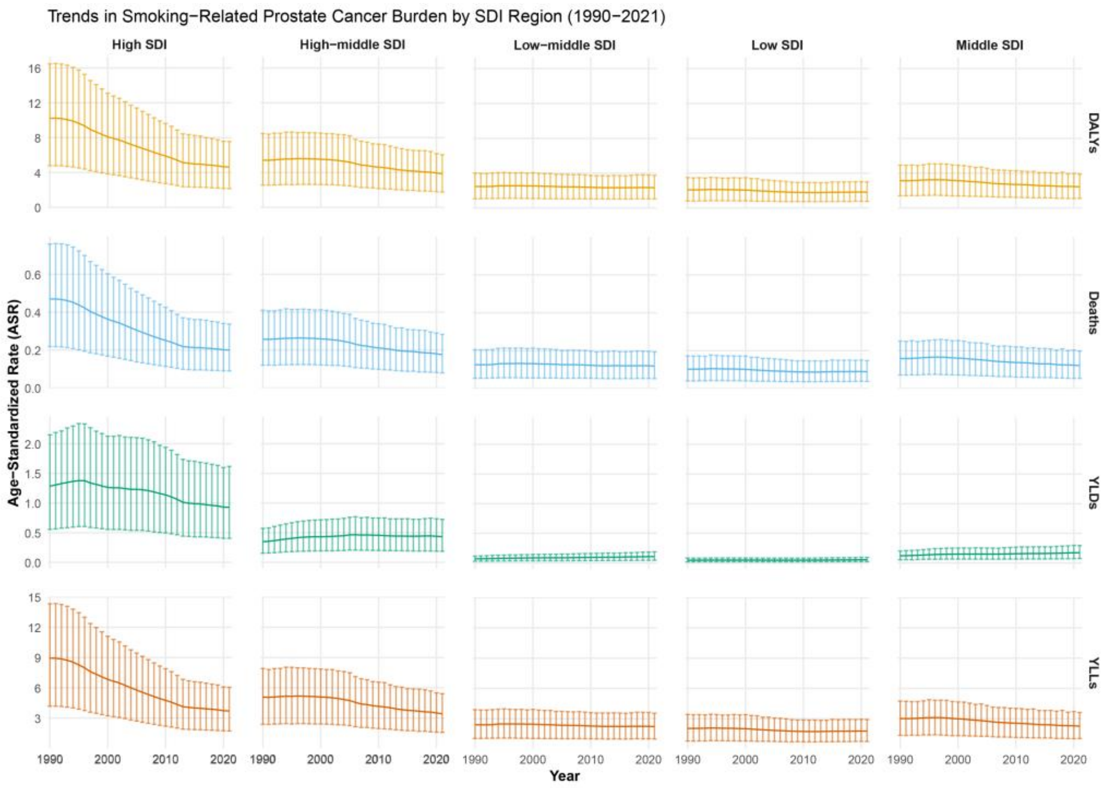

EAPC: estimated annual percentage change. ASR: age-standardized rate. YLDs: years lived with disability. YLLs: years of life lost. DALYs: disability-adjusted-life-years.

Figure S4. The trend of smoking-related prostate cancer-related ASRs of deaths, YLDs, YLLs and DALYs for different GBD regions between 1990 and 2021. The solid line displays the global expected values based on the SDI values. The figure displays a significant positive ($\rho > 0$) correlation ($P < 0.001$) between SDI and smoking-related prostate cancer, suggesting a strong association between the prostate cancer burden due to smoking and the level of regional socioeconomic development. (A.Deaths,B.YLDs,C.YYLs,D.DALYS)

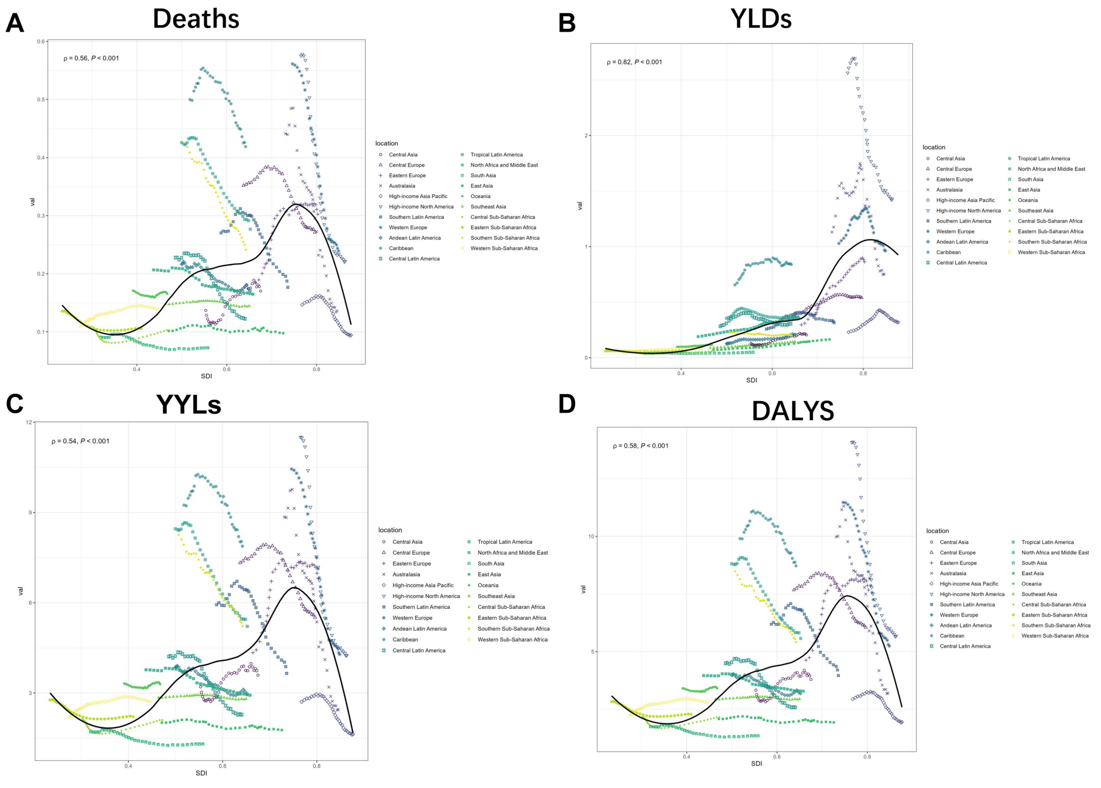

3

ASR: age-standardized rate, YLDs: Years Lived with Disability, YLLs: Years of Life Lost, DALYs: disability-adjusted-life-years.

Figure S5. Health inequality in smoking-related prostate cancer burden, 1990-2021, as assessed by regression curves and concentration curves. The regression curves illustrate the relationship between ASRs of DALYs and the relative position of countries on the SDI scale, with steeper slopes indicating greater inequality. The concentration curves depict the cumulative share of the population (ranked by SDI) against the cumulative share of DALYs, with the concentration index (CI) quantifying the degree of inequality: a CI of 0 indicates perfect equality, while a CI of 1 represents complete inequality. The CI decreased from 0.34 in 1990 to 0.15 in 2021, suggesting a reduction in inequality over time.

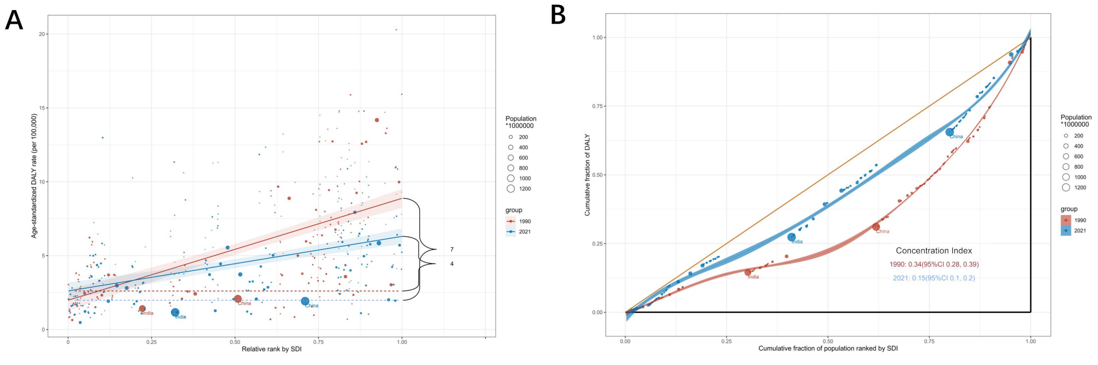

4

ASR: age-standardized rate, YLDs: Years Lived with Disability,DALYs:disability-adjusted-life-years, CI: concentration index ,SDI: socio-demographic index.

Figure S6. The predicted results in smoking-related prostate cancer-related ASRs of deaths, YLDs, YLLs and DALYs from 2022 to 2031.

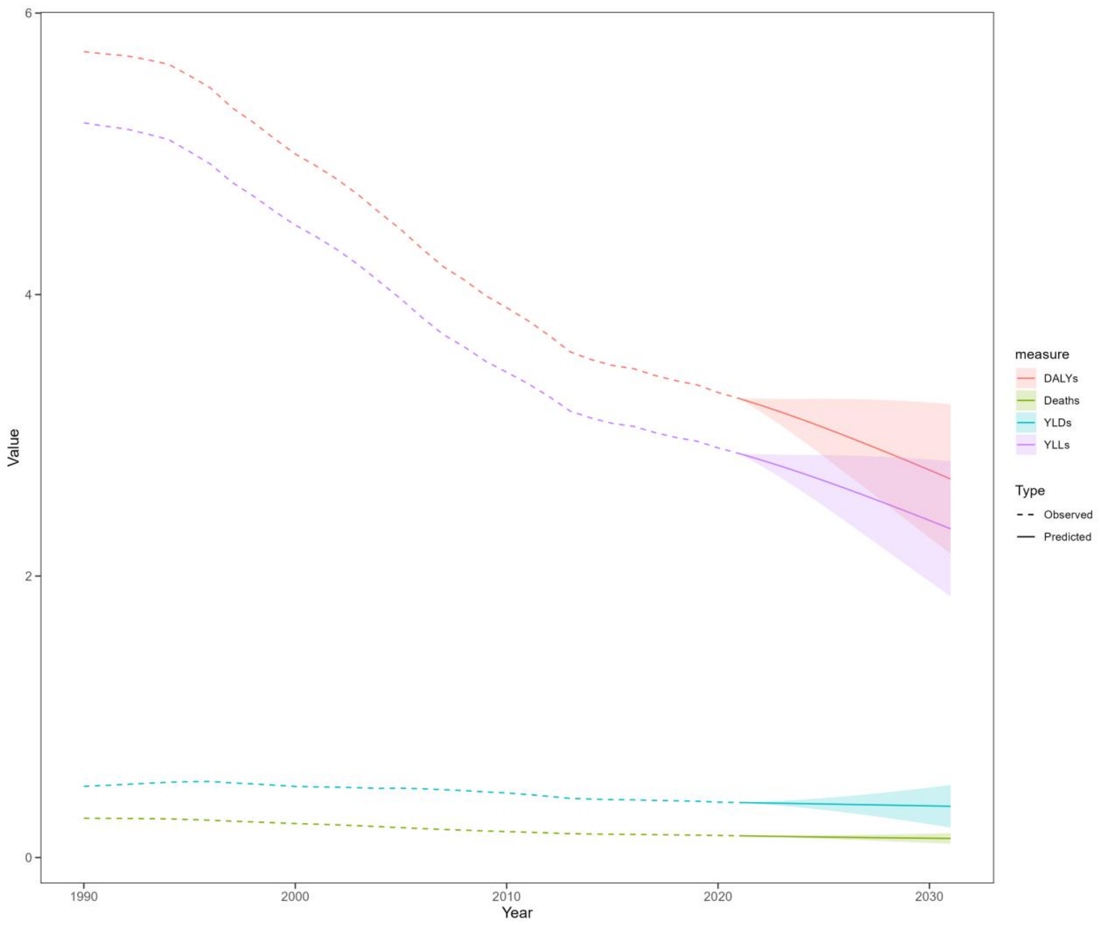

5

ASR: age-standardized rate, YLDs: Years Lived with Disability, YLLs: Years of Life Lost, DALYs: disability-adjusted-life-years.

Table legend

Table S1. The EAPC of smoking-related prostate cancer-related ASRs of deaths, YLDs, YLLs and DALYs for different countries between 1990 and 2021.

Table S2. The EAPC of smoking-related prostate cancer-related ASRs of deaths, YLDs, YLLs and DALYs for different age groups between 1990 and 2021.

Table S3. The EAPC of smoking-related prostate cancer-related ASRs of deaths, YLDs, YLLs and DALYs for different SDI regions between 1990 and 2021.

Table S4. The EAPC of smoking-related prostate cancer-related ASRs of deaths, YLDs, YLLs and DALYs for different GBD regions between 1990 and 2021.

Table S5. Ten year predictions of smoking related prostate cancer burden: the ARIMA models results and confidence intervals between from 2022 to 2031.

Table S6. The ARIMA models parameters and statistical validation: fitting results of smoking related prostate cancer burden indicators between 1990 and 2021.

.

Table S1. The EAPC of smoking-related prostate cancer-related ASRs of deaths, YLDs, YLLs and DALYs for different countries between 1990 and 2021.

	location	measure	sex	cause	age	EAPC	LCI	UCI	EAPC_95CI
1	Taiwan (Province of China)	Deaths	Both	Prostate cancer	All ages	3.012709611	2.649791285	3.376911035	3.01 (2.65 to 3.38)
2	Taiwan (Province of China)	Deaths	Both	Prostate cancer	Age-standardized	-0.399751905	-0.790367863	-0.007597984	-0.4 (-0.79 to -0.01)
3	Taiwan (Province of China)	DALYs (Disability-Adjusted Life Years)	Both	Prostate cancer	All ages	2.421455106	2.098205746	2.745727895	2.42 (2.1 to 2.75)
4	Taiwan (Province of China)	DALYs (Disability-Adjusted Life Years)	Both	Prostate cancer	Age-standardized	-0.504590534	-0.867285582	-0.140568501	-0.5 (-0.87 to -0.14)
5	Taiwan (Province of China)	YLDs (Years Lived with Disability)	Both	Prostate cancer	All ages	4.43737344	3.893068712	4.984529827	4.44 (3.89 to 4.98)
6	Taiwan (Province of China)	YLDs (Years Lived with Disability)	Both	Prostate cancer	Age-standardized	1.606221879	1.023227939	2.192580213	1.61 (1.02 to 2.19)
7	Taiwan (Province of China)	YLLs (Years of Life Lost)	Both	Prostate cancer	All ages	2.192656725	1.88379318	2.502456598	2.19 (1.88 to 2.5)
8	Taiwan (Province of China)	YLLs (Years of Life Lost)	Both	Prostate cancer	Age-standardized	-0.740989895	-1.089620432	-0.391130535	-0.74 (-1.09 to

									-0.39)
9	China	Deaths	Both	Prostate cancer	All ages	2.680911485	2.536944879	2.825080226	2.68 (2.54 to 2.83)
10	China	Deaths	Both	Prostate cancer	Age-standardized	-0.229012425	-0.350093679	-0.107784049	-0.23 (-0.35 to -0.11)
11	China	DALYs (Disability-Adjusted Life Years)	Both	Prostate cancer	All ages	2.241048505	2.069742186	2.412642331	2.24 (2.07 to 2.41)
12	China	DALYs (Disability-Adjusted Life Years)	Both	Prostate cancer	Age-standardized	-0.343657436	-0.454793757	-0.232397038	-0.34 (-0.45 to -0.23)
13	China	YLDs (Years Lived with Disability)	Both	Prostate cancer	All ages	5.756812458	5.539299641	5.974773562	5.76 (5.54 to 5.97)
14	China	YLDs (Years Lived with Disability)	Both	Prostate cancer	Age-standardized	3.060838138	2.907787418	3.214116485	3.06 (2.91 to 3.21)
15	China	YLLs (Years of Life Lost)	Both	Prostate cancer	All ages	2.038541005	1.873544525	2.203804717	2.04 (1.87 to 2.2)
16	China	YLLs (Years of Life Lost)	Both	Prostate cancer	Age-standardized	-0.530614627	-0.64172506	-0.419379941	-0.53 (-0.64 to

									-0.42)
17	Cambodia	Deaths	Both	Prostate cancer	All ages	2.876342637	2.737515354	3.015357515	2.88 (2.74 to 3.02)
18	Cambodia	Deaths	Both	Prostate cancer	Age-standardized	1.02164138	0.813685268	1.230026459	1.02 (0.81 to 1.23)
19	Cambodia	DALYs (Disability-Adjusted Life Years)	Both	Prostate cancer	All ages	2.708918284	2.601318183	2.816631227	2.71 (2.6 to 2.82)
20	Cambodia	DALYs (Disability-Adjusted Life Years)	Both	Prostate cancer	Age-standardized	0.854870321	0.651685563	1.058465247	0.85 (0.65 to 1.06)
21	Cambodia	YLDs (Years Lived with Disability)	Both	Prostate cancer	All ages	4.431717226	4.344040868	4.519467256	4.43 (4.34 to 4.52)
22	Cambodia	YLDs (Years Lived with Disability)	Both	Prostate cancer	Age-standardized	2.437813721	2.2847663	2.591090144	2.44 (2.28 to 2.59)
23	Cambodia	YLLs (Years of Life Lost)	Both	Prostate cancer	All ages	2.657932906	2.547982939	2.768000759	2.66 (2.55 to 2.77)
24	Cambodia	YLLs (Years of Life Lost)	Both	Prostate cancer	Age-standardized	0.809324192	0.60363562	1.015433304	0.81 (0.6 to 1.02)

25	Indonesia	Deaths	Both	Prostate cancer	All ages	2.842772049	2.68810227	2.997674793	2.84 (2.69 to 3)
26	Indonesia	Deaths	Both	Prostate cancer	Age-standardized	1.383655166	1.220224694	1.547349514	1.38 (1.22 to 1.55)
27	Indonesia	DALYs (Disability-Adjusted Life Years)	Both	Prostate cancer	All ages	2.905813739	2.819282509	2.992417792	2.91 (2.82 to 2.99)
28	Indonesia	DALYs (Disability-Adjusted Life Years)	Both	Prostate cancer	Age-standardized	1.364022993	1.243992564	1.484195726	1.36 (1.24 to 1.48)
29	Indonesia	YLDs (Years Lived with Disability)	Both	Prostate cancer	All ages	4.284464716	4.200002992	4.368994903	4.28 (4.2 to 4.37)
30	Indonesia	YLDs (Years Lived with Disability)	Both	Prostate cancer	Age-standardized	2.63258691	2.584402108	2.680794346	2.63 (2.58 to 2.68)
31	Indonesia	YLLs (Years of Life Lost)	Both	Prostate cancer	All ages	2.858530904	2.769616134	2.947522602	2.86 (2.77 to 2.95)
32	Indonesia	YLLs (Years of Life Lost)	Both	Prostate cancer	Age-standardized	1.321919854	1.19842925	1.445561152	1.32 (1.2 to 1.45)
33	Malaysia	Deaths	Both	Prostate cancer	All ages	0.642391266	0.497274839	0.787717239	0.64 (0.5 to 0.79)

34	Malaysia	Deaths	Both	Prostate cancer	Age-standardized	-1.29547116	-1.532733801	-1.057636821	-1.3 (-1.53 to -1.06)
35	Malaysia	DALYs (Disability-Adjusted Life Years)	Both	Prostate cancer	All ages	0.7749848 3	0.669922707	0.880156598	0.77 (0.67 to 0.88)
36	Malaysia	DALYs (Disability-Adjusted Life Years)	Both	Prostate cancer	Age-standardized	-1.300211264	-1.495310799	-1.104725312	-1.3 (-1.5 to -1.1)
37	Malaysia	YLDs (Years Lived with Disability)	Both	Prostate cancer	All ages	2.927258191	2.834927736	3.019671546	2.93 (2.83 to 3.02)
38	Malaysia	YLDs (Years Lived with Disability)	Both	Prostate cancer	Age-standardized	0.686971753	0.441471458	0.933072103	0.69 (0.44 to 0.93)
39	Malaysia	YLLs (Years of Life Lost)	Both	Prostate cancer	All ages	0.667768575	0.561829592	0.773819162	0.67 (0.56 to 0.77)
40	Malaysia	YLLs (Years of Life Lost)	Both	Prostate cancer	Age-standardized	-1.396365052	-1.591819353	-1.200522547	-1.4 (-1.59 to -1.2)
41	Myanmar	Deaths	Both	Prostate cancer	All ages	-0.639877433	-0.772627397	-0.506949871	-0.64 (-0.77 to -0.51)
42	Myanmar	Deaths	Both	Prostate cancer	Age-standardized	-2.100993047	-2.204988099	-1.996887406	-2.1 (-2.2 to -2)

43	Myanmar	DALYs (Disability-Adjusted Life Years)	Both	Prostate cancer	All ages	-0.90565271	-1.091082799	-0.719874985	-0.91 (-1.09 to -0.72)
44	Myanmar	DALYs (Disability-Adjusted Life Years)	Both	Prostate cancer	Age-standardized	-2.200926442	-2.316738177	-2.084977403	-2.2 (-2.32 to -2.08)
45	Myanmar	YLDs (Years Lived with Disability)	Both	Prostate cancer	All ages	0.510723416	0.220872463	0.801412652	0.51 (0.22 to 0.8)
46	Myanmar	YLDs (Years Lived with Disability)	Both	Prostate cancer	Age-standardized	-0.837823543	-1.048939221	-0.626257443	-0.84 (-1.05 to -0.63)
47	Myanmar	YLLs (Years of Life Lost)	Both	Prostate cancer	All ages	-0.94654368	-1.128343866	-0.764409209	-0.95 (-1.13 to -0.76)
48	Myanmar	YLLs (Years of Life Lost)	Both	Prostate cancer	Age-standardized	-2.239375287	-2.352061907	-2.126558627	-2.24 (-2.35 to -2.13)
49	Lao People's Democratic Republic	Deaths	Both	Prostate cancer	All ages	0.848702622	0.740735046	0.95678591	0.85 (0.74 to 0.96)
50	Lao People's Democratic Republic	Deaths	Both	Prostate cancer	Age-standardized	0.340052832	0.273209232	0.406940991	0.34 (0.27 to 0.41)

51	Lao People's Democratic Republic	DALYs (Disability-Adjusted Life Years)	Both	Prostate cancer	All ages	0.635319585	0.4850561 7	0.7858077	0.64 (0.49 to 0.79)
52	Lao People's Democratic Republic	DALYs (Disability-Adjusted Life Years)	Both	Prostate cancer	Age-standardized	0.103227878	0.048298074	0.1581878 4	0.1 (0.05 to 0.16)
53	Lao People's Democratic Republic	YLDs (Years Lived with Disability)	Both	Prostate cancer	All ages	1.997104567	1.765375851	2.229360949	2 (1.77 to 2.23)
54	Lao People's Democratic Republic	YLDs (Years Lived with Disability)	Both	Prostate cancer	Age-standardized	1.386301315	1.329498729	1.443135744	1.39 (1.33 to 1.44)
55	Lao People's Democratic Republic	YLLs (Years of Life Lost)	Both	Prostate cancer	All ages	0.599188491	0.451678922	0.746914672	0.6 (0.45 to 0.75)
56	Lao People's Democratic Republic	YLLs (Years of Life Lost)	Both	Prostate cancer	Age-standardized	0.069726802	0.013144262	0.126341354	0.07 (0.01 to 0.13)
57	Democratic People's Republic of Korea	Deaths	Both	Prostate cancer	All ages	1.403535835	1.341546505	1.465563082	1.4 (1.34 to 1.47)
58	Democratic People's Republic of Korea	Deaths	Both	Prostate cancer	Age-standardized	-0.45246714	-0.481815944	-0.423109682	-0.45 (-0.48 to -0.42)
59	Democratic People's Republic of	DALYs (Disability-Adjusted	Both	Prostate canc	All ages	1.187981595	1.083180248	1.292891598	1.19 (1.08 to

	Korea	Life Years)		er					1.29)
60	Democratic People's Republic of Korea	DALYs (Disability-Adjusted Life Years)	Both	Prostate cancer	Age-standardized	-0.274478303	-0.331456671	-0.217467362	-0.27 (-0.33 to -0.22)
61	Democratic People's Republic of Korea	YLDs (Years Lived with Disability)	Both	Prostate cancer	All ages	2.778954487	2.658244839	2.89980607	2.78 (2.66 to 2.9)
62	Democratic People's Republic of Korea	YLDs (Years Lived with Disability)	Both	Prostate cancer	Age-standardized	1.336734837	1.251908779	1.421631959	1.34 (1.25 to 1.42)
63	Democratic People's Republic of Korea	YLLs (Years of Life Lost)	Both	Prostate cancer	All ages	1.109191881	1.002734081	1.215761889	1.11 (1 to 1.22)
64	Democratic People's Republic of Korea	YLLs (Years of Life Lost)	Both	Prostate cancer	Age-standardized	-0.353275516	-0.41221677	-0.294299377	-0.35 (-0.41 to -0.29)
65	Maldives	Deaths	Both	Prostate cancer	All ages	-0.335950527	-0.512975397	-0.158610663	-0.34 (-0.51 to -0.16)
66	Maldives	Deaths	Both	Prostate cancer	Age-standardized	-2.69578441	-2.83560006	-2.555767571	-2.7 (-2.84 to -2.56)
67	Maldives	DALYs (Disability-Adjusted Life Years)	Both	Prostate cancer	All ages	-0.948296646	-1.13900025	-0.757225174	-0.95 (-1.14 to -0.7

									6)
68	Maldives	DALYs (Disability-Adjusted Life Years)	Both	Prostate cancer	Age-standardized	-2.820241354	-2.993321384	-2.646852513	-2.82 (-2.99 to -2.65)
69	Maldives	YLDs (Years Lived with Disability)	Both	Prostate cancer	All ages	2.039336026	1.672644857	2.407349699	2.04 (1.67 to 2.41)
70	Maldives	YLDs (Years Lived with Disability)	Both	Prostate cancer	Age-standardized	0.233222297	0.058345131	0.408405105	0.23 (0.06 to 0.41)
71	Maldives	YLLs (Years of Life Lost)	Both	Prostate cancer	All ages	-1.095856598	-1.285571969	-0.905776621	-1.1 (-1.29 to -0.91)
72	Maldives	YLLs (Years of Life Lost)	Both	Prostate cancer	Age-standardized	-2.96441054	-3.140827408	-2.787672352	-2.96 (-3.14 to -2.79)
73	Sri Lanka	Deaths	Both	Prostate cancer	All ages	-0.398442658	-0.727039935	-0.068757711	-0.4 (-0.73 to -0.07)
74	Sri Lanka	Deaths	Both	Prostate cancer	Age-standardized	-2.932172827	-3.228441525	-2.634997094	-2.93 (-3.23 to -2.63)

75	Sri Lanka	DALYs (Disability-Adjusted Life Years)	Both	Prostate cancer	All ages	-0.295450454	-0.596342746	0.006352632	-0.3 (-0.6 to 0.01)
76	Sri Lanka	DALYs (Disability-Adjusted Life Years)	Both	Prostate cancer	Age-standardized	-2.722550453	-2.985978408	-2.458407197	-2.72 (-2.99 to -2.46)
77	Sri Lanka	YLDs (Years Lived with Disability)	Both	Prostate cancer	All ages	2.270377621	1.961045383	2.58064832	2.27 (1.96 to 2.58)
78	Sri Lanka	YLDs (Years Lived with Disability)	Both	Prostate cancer	Age-standardized	-0.243520595	-0.505799354	0.019449563	-0.24 (-0.51 to 0.02)
79	Sri Lanka	YLLs (Years of Life Lost)	Both	Prostate cancer	All ages	-0.440822891	-0.737246621	-0.143513965	-0.44 (-0.74 to -0.14)
80	Sri Lanka	YLLs (Years of Life Lost)	Both	Prostate cancer	Age-standardized	-2.857678392	-3.117397563	-2.597262976	-2.86 (-3.12 to -2.6)
81	Philippines	Deaths	Both	Prostate cancer	All ages	0.712584829	0.66559789	0.7595937	0.71 (0.67 to 0.76)
82	Philippines	Deaths	Both	Prostate cancer	Age-standardized	-0.986538332	-1.07486961	-0.898128181	-0.99 (-1.07 to -0.9)

83	Philippines	DALYs (Disability-Adjusted Life Years)	Both	Prostate cancer	All ages	0.801533898	0.769625702	0.833452198	0.8 (0.77 to 0.83)
84	Philippines	DALYs (Disability-Adjusted Life Years)	Both	Prostate cancer	Age-standardized	-0.792119053	-0.85820459	-0.725989464	-0.79 (-0.86 to -0.73)
85	Philippines	YLDs (Years Lived with Disability)	Both	Prostate cancer	All ages	1.63019447	1.553676592	1.706770002	1.63 (1.55 to 1.71)
86	Philippines	YLDs (Years Lived with Disability)	Both	Prostate cancer	Age-standardized	0.032611637	-0.016727048	0.081974669	0.03 (-0.02 to 0.08)
87	Philippines	YLLs (Years of Life Lost)	Both	Prostate cancer	All ages	0.770795652	0.739057395	0.802543907	0.77 (0.74 to 0.8)
88	Philippines	YLLs (Years of Life Lost)	Both	Prostate cancer	Age-standardized	-0.821997536	-0.890349464	-0.753598467	-0.82 (-0.89 to -0.75)
89	Timor-Leste	Deaths	Both	Prostate cancer	All ages	3.085364542	2.924721568	3.246258244	3.09 (2.92 to 3.25)
90	Timor-Leste	Deaths	Both	Prostate cancer	Age-standardized	0.115797966	0.046044342	0.185600223	0.12 (0.05 to 0.19)
91	Timor-Leste	DALYs (Disability-Adjusted	Both	Prostate canc	All ages	2.737283113	2.592213347	2.882558014	2.74 (2.59 to

		Life Years)		er					2.88)
92	Timor-Leste	DALYs (Disability-Adjusted Life Years)	Both	Prostate cancer	Age-standardized	0.067962799	-0.004557421	0.140535612	0.07 (0 to 0.14)
93	Timor-Leste	YLDs (Years Lived with Disability)	Both	Prostate cancer	All ages	4.095368962	3.882562634	4.30861123	4.1 (3.88 to 4.31)
94	Timor-Leste	YLDs (Years Lived with Disability)	Both	Prostate cancer	Age-standardized	1.252075442	1.170155303	1.334061914	1.25 (1.17 to 1.33)
95	Timor-Leste	YLLs (Years of Life Lost)	Both	Prostate cancer	All ages	2.698164645	2.554136516	2.842395048	2.7 (2.55 to 2.84)
96	Timor-Leste	YLLs (Years of Life Lost)	Both	Prostate cancer	Age-standardized	0.034906629	-0.038533781	0.108400995	0.03 (-0.04 to 0.11)
97	Thailand	Deaths	Both	Prostate cancer	All ages	2.394184517	2.182251853	2.606556742	2.39 (2.18 to 2.61)
98	Thailand	Deaths	Both	Prostate cancer	Age-standardized	-1.503387505	-1.633977689	-1.37262395	-1.5 (-1.63 to -1.37)
99	Thailand	DALYs (Disability-Adjusted Life Years)	Both	Prostate cancer	All ages	2.320799758	2.131378111	2.510572722	2.32 (2.13 to 2.51)

100	Thailand	DALYs (Disability-Adjusted Life Years)	Both	Prostate cancer	Age-standardized	-1.216691428	-1.336617539	-1.096619546	-1.22 (-1.34 to -1.1)
101	Thailand	YLDs (Years Lived with Disability)	Both	Prostate cancer	All ages	4.755193095	4.594592395	4.916040392	4.76 (4.59 to 4.92)
102	Thailand	YLDs (Years Lived with Disability)	Both	Prostate cancer	Age-standardized	1.213514098	1.085692382	1.341497444	1.21 (1.09 to 1.34)
103	Thailand	YLLs (Years of Life Lost)	Both	Prostate cancer	All ages	2.171709087	1.983498636	2.360266881	2.17 (1.98 to 2.36)
104	Thailand	YLLs (Years of Life Lost)	Both	Prostate cancer	Age-standardized	-1.362406631	-1.481010319	-1.24366016	-1.36 (-1.48 to -1.24)
105	Viet Nam	Deaths	Both	Prostate cancer	All ages	1.401783786	1.220154636	1.583738852	1.4 (1.22 to 1.58)
106	Viet Nam	Deaths	Both	Prostate cancer	Age-standardized	-0.123913126	-0.352718329	0.105417449	-0.12 (-0.35 to 0.11)
107	Viet Nam	DALYs (Disability-Adjusted Life Years)	Both	Prostate cancer	All ages	1.609576374	1.478181991	1.741140886	1.61 (1.48 to 1.74)

108	Viet Nam	DALYs (Disability-Adjusted Life Years)	Both	Prostate cancer	Age-standardized	0.043301831	-0.15824375	0.24525426 2	0.04 (-0.16 to 0.25)
109	Viet Nam	YLDs (Years Lived with Disability)	Both	Prostate cancer	All ages	3.944170338	3.79995549 2	4.08858554 9	3.94 (3.8 to 4.09)
110	Viet Nam	YLDs (Years Lived with Disability)	Both	Prostate cancer	Age-standardized	2.362180971	2.15489845 1	2.56988408 7	2.36 (2.15 to 2.57)
111	Viet Nam	YLLs (Years of Life Lost)	Both	Prostate cancer	All ages	1.484007693	1.35170279 9	1.61648529 8	1.48 (1.35 to 1.62)
112	Viet Nam	YLLs (Years of Life Lost)	Both	Prostate cancer	Age-standardized	-0.079158987	-0.28420818 7	0.12631186 2	-0.08 (-0.28 to 0.13)
113	Kiribati	Deaths	Both	Prostate cancer	All ages	0.973478284	0.85387088 4	1.09322753 3	0.97 (0.85 to 1.09)
114	Kiribati	Deaths	Both	Prostate cancer	Age-standardized	0.589162395	0.35907834 9	0.81977393 4	0.59 (0.36 to 0.82)
115	Kiribati	DALYs (Disability-Adjusted Life Years)	Both	Prostate cancer	All ages	1.091290148	0.99367399 6	1.18900065 2	1.09 (0.99 to 1.19)
116	Kiribati	DALYs (Disability-Adjusted	Both	Prostate canc	Age-standardized	0.533489047	0.30230193 5	0.76520902 3	0.53 (0.3 to

		Life Years)		er					0.77)
117	Kiribati	YLDs (Years Lived with Disability)	Both	Prostate cancer	All ages	1.620146029	1.478176929	1.762313745	1.62 (1.48 to 1.76)
118	Kiribati	YLDs (Years Lived with Disability)	Both	Prostate cancer	Age-standardized	1.010695308	0.735510464	1.286631889	1.01 (0.74 to 1.29)
119	Kiribati	YLLs (Years of Life Lost)	Both	Prostate cancer	All ages	1.078076278	0.981426459	1.174818601	1.08 (0.98 to 1.17)
120	Kiribati	YLLs (Years of Life Lost)	Both	Prostate cancer	Age-standardized	0.521715356	0.291479543	0.752479715	0.52 (0.29 to 0.75)
121	Micronesia (Federated States of)	Deaths	Both	Prostate cancer	All ages	1.102146035	0.833255023	1.371754097	1.1 (0.83 to 1.37)
122	Micronesia (Federated States of)	Deaths	Both	Prostate cancer	Age-standardized	-0.015417903	-0.049289021	0.018464693	-0.02 (-0.05 to 0.02)
123	Micronesia (Federated States of)	DALYs (Disability-Adjusted Life Years)	Both	Prostate cancer	All ages	1.512816863	1.214512246	1.812000658	1.51 (1.21 to 1.81)
124	Micronesia (Federated States of)	DALYs (Disability-Adjusted Life Years)	Both	Prostate cancer	Age-standardized	0.041553951	0.014914579	0.068200419	0.04 (0.01 to 0.07)

125	Micronesia (Federated States of)	YLDs (Years Lived with Disability)	Both	Prostate cancer	All ages	2.750555023	2.430124777	3.071987666	2.75 (2.43 to 3.07)
126	Micronesia (Federated States of)	YLDs (Years Lived with Disability)	Both	Prostate cancer	Age-standardized	1.193263129	1.163360085	1.223175013	1.19 (1.16 to 1.22)
127	Micronesia (Federated States of)	YLLs (Years of Life Lost)	Both	Prostate cancer	All ages	1.474497564	1.177417575	1.772449848	1.47 (1.18 to 1.77)
128	Micronesia (Federated States of)	YLLs (Years of Life Lost)	Both	Prostate cancer	Age-standardized	0.006646985	-0.020425606	0.033726906	0.01 (-0.02 to 0.03)
129	Fiji	Deaths	Both	Prostate cancer	All ages	1.951162767	1.631288653	2.272043653	1.95 (1.63 to 2.27)
130	Fiji	Deaths	Both	Prostate cancer	Age-standardized	-0.458452843	-0.719820827	-0.196396773	-0.46 (-0.72 to -0.2)
131	Fiji	DALYs (Disability-Adjusted Life Years)	Both	Prostate cancer	All ages	1.950972654	1.672903863	2.229801944	1.95 (1.67 to 2.23)
132	Fiji	DALYs (Disability-Adjusted Life Years)	Both	Prostate cancer	Age-standardized	-0.405001867	-0.641846645	-0.167592512	-0.41 (-0.64 to -0.17)
133	Fiji	YLDs (Years Lived with	Both	Prostate canc	All ages	2.5063418	2.249380356	2.763949009	2.51 (2.25 to

		Disability)		er					2.76)
134	Fiji	YLDs (Years Lived with Disability)	Both	Prostate cancer	Age-standardized	0.099951522	-0.12504874	0.325458669	0.1 (-0.13 to 0.33)
135	Fiji	YLLs (Years of Life Lost)	Both	Prostate cancer	All ages	1.930936151	1.652053956	2.210583459	1.93 (1.65 to 2.21)
136	Fiji	YLLs (Years of Life Lost)	Both	Prostate cancer	Age-standardized	-0.42268162	-0.660006134	-0.184790135	-0.42 (-0.66 to -0.18)
137	Samoa	Deaths	Both	Prostate cancer	All ages	0.53232868	0.474665345	0.590025109	0.53 (0.47 to 0.59)
138	Samoa	Deaths	Both	Prostate cancer	Age-standardized	-0.329480434	-0.374497237	-0.28444329	-0.33 (-0.37 to -0.28)
139	Samoa	DALYs (Disability-Adjusted Life Years)	Both	Prostate cancer	All ages	0.451092392	0.393355363	0.508862626	0.45 (0.39 to 0.51)
140	Samoa	DALYs (Disability-Adjusted Life Years)	Both	Prostate cancer	Age-standardized	-0.379967815	-0.42885313	-0.3310585	-0.38 (-0.43 to -0.33)
141	Samoa	YLDs (Years Lived with	Both	Prostate canc	All ages	1.359142761	1.235029686	1.483407997	1.36 (1.24 to

		Disability)		er					1.48)
142	Samoa	YLDs (Years Lived with Disability)	Both	Prostate cancer	Age-standardized	0.535996849	0.429110848	0.642996608	0.54 (0.43 to 0.64)
143	Samoa	YLLs (Years of Life Lost)	Both	Prostate cancer	All ages	0.415336163	0.358423975	0.472280625	0.42 (0.36 to 0.47)
144	Samoa	YLLs (Years of Life Lost)	Both	Prostate cancer	Age-standardized	-0.41530669	-0.4644306	-0.366158536	-0.42 (-0.46 to -0.37)
145	Marshall Islands	Deaths	Both	Prostate cancer	All ages	2.416878013	2.082011898	2.752842611	2.42 (2.08 to 2.75)
146	Marshall Islands	Deaths	Both	Prostate cancer	Age-standardized	1.172864021	1.082516495	1.263292299	1.17 (1.08 to 1.26)
147	Marshall Islands	DALYs (Disability-Adjusted Life Years)	Both	Prostate cancer	All ages	2.7013309	2.397802057	3.005759466	2.7 (2.4 to 3.01)
148	Marshall Islands	DALYs (Disability-Adjusted Life Years)	Both	Prostate cancer	Age-standardized	0.953903674	0.871115046	1.036760249	0.95 (0.87 to 1.04)
149	Marshall Islands	YLDs (Years Lived with Disability)	Both	Prostate cancer	All ages	3.438435792	3.028131395	3.850374206	3.44 (3.03 to 3.85)

150	Marshall Islands	YLDs (Years Lived with Disability)	Both	Prostate cancer	Age-standardized	1.518094688	1.419028903	1.61725724	1.52 (1.42 to 1.62)
151	Marshall Islands	YLLs (Years of Life Lost)	Both	Prostate cancer	All ages	2.68028133	2.379815115	2.981629358	2.68 (2.38 to 2.98)
152	Marshall Islands	YLLs (Years of Life Lost)	Both	Prostate cancer	Age-standardized	0.938269714	0.854718704	1.02188994	0.94 (0.85 to 1.02)
153	Papua New Guinea	Deaths	Both	Prostate cancer	All ages	0.987040976	0.81973011	1.154629494	0.99 (0.82 to 1.15)
154	Papua New Guinea	Deaths	Both	Prostate cancer	Age-standardized	0.757248762	0.683756033	0.830795135	0.76 (0.68 to 0.83)
155	Papua New Guinea	DALYs (Disability-Adjusted Life Years)	Both	Prostate cancer	All ages	1.036641346	0.823351403	1.2503825	1.04 (0.82 to 1.25)
156	Papua New Guinea	DALYs (Disability-Adjusted Life Years)	Both	Prostate cancer	Age-standardized	0.774054742	0.702990828	0.845168804	0.77 (0.7 to 0.85)
157	Papua New Guinea	YLDs (Years Lived with Disability)	Both	Prostate cancer	All ages	1.338929173	1.101352788	1.577063834	1.34 (1.1 to 1.58)
158	Papua New Guinea	YLDs (Years Lived with Disability)	Both	Prostate cancer	Age-standardized	1.081184124	0.9983694	1.164066753	1.08 (1 to 1.16)

159	Papua New Guinea	YLLs (Years of Life Lost)	Both	Prostate cancer	All ages	1.028057369	0.815400521	1.241162789	1.03 (0.82 to 1.24)
160	Papua New Guinea	YLLs (Years of Life Lost)	Both	Prostate cancer	Age-standardized	0.765519194	0.69448637	0.836602127	0.77 (0.69 to 0.84)
161	Solomon Islands	Deaths	Both	Prostate cancer	All ages	1.31556405	1.132440853	1.499018834	1.32 (1.13 to 1.5)
162	Solomon Islands	Deaths	Both	Prostate cancer	Age-standardized	0.516328303	0.33052882	0.702471863	0.52 (0.33 to 0.7)
163	Solomon Islands	DALYs (Disability-Adjusted Life Years)	Both	Prostate cancer	All ages	1.290151531	1.06541075	1.515392073	1.29 (1.07 to 1.52)
164	Solomon Islands	DALYs (Disability-Adjusted Life Years)	Both	Prostate cancer	Age-standardized	0.619337559	0.395521539	0.843652541	0.62 (0.4 to 0.84)
165	Solomon Islands	YLDs (Years Lived with Disability)	Both	Prostate cancer	All ages	1.877822709	1.6388482	2.117359098	1.88 (1.64 to 2.12)
166	Solomon Islands	YLDs (Years Lived with Disability)	Both	Prostate cancer	Age-standardized	1.216955898	0.974989696	1.459501923	1.22 (0.97 to 1.46)
167	Solomon Islands	YLLs (Years of Life Lost)	Both	Prostate cancer	All ages	1.273706658	1.049378622	1.498532699	1.27 (1.05 to 1.5)

168	Solomon Islands	YLLs (Years of Life Lost)	Both	Prostate cancer	Age-standardized	0.602960705	0.379669609	0.826748503	0.6 (0.38 to 0.83)
169	Vanuatu	Deaths	Both	Prostate cancer	All ages	0.491451938	0.397041151	0.585951506	0.49 (0.4 to 0.59)
170	Vanuatu	Deaths	Both	Prostate cancer	Age-standardized	-0.460555991	-0.567040132	-0.353957815	-0.46 (-0.57 to -0.35)
171	Vanuatu	DALYs (Disability-Adjusted Life Years)	Both	Prostate cancer	All ages	0.457223192	0.362900534	0.551634495	0.46 (0.36 to 0.55)
172	Vanuatu	DALYs (Disability-Adjusted Life Years)	Both	Prostate cancer	Age-standardized	-0.504971175	-0.608914728	-0.400918917	-0.5 (-0.61 to -0.4)
173	Vanuatu	YLDs (Years Lived with Disability)	Both	Prostate cancer	All ages	0.845815408	0.746248013	0.945481206	0.85 (0.75 to 0.95)
174	Vanuatu	YLDs (Years Lived with Disability)	Both	Prostate cancer	Age-standardized	-0.138439313	-0.237014457	-0.039766767	-0.14 (-0.24 to -0.04)
175	Vanuatu	YLLs (Years of Life Lost)	Both	Prostate cancer	All ages	0.445716772	0.35143546	0.540086662	0.45 (0.35 to 0.54)

176	Vanuatu	YLLs (Years of Life Lost)	Both	Prostate cancer	Age-standardized	-0.515542 8	-0.619783 982	-0.411192 277	-0.52 (-0.62 to -0.41)
177	Azerbaijan	Deaths	Both	Prostate cancer	All ages	1.846162294	1.541207349	2.152033099	1.85 (1.54 to 2.15)
178	Azerbaijan	Deaths	Both	Prostate cancer	Age-standardized	1.118083904	0.713330765	1.524463692	1.12 (0.71 to 1.52)
179	Azerbaijan	DALYs (Disability-Adjusted Life Years)	Both	Prostate cancer	All ages	1.62284692	1.443717408	1.802292739	1.62 (1.44 to 1.8)
180	Azerbaijan	DALYs (Disability-Adjusted Life Years)	Both	Prostate cancer	Age-standardized	0.819720135	0.46086023	1.179861937	0.82 (0.46 to 1.18)
181	Azerbaijan	YLDs (Years Lived with Disability)	Both	Prostate cancer	All ages	3.284399797	3.126331385	3.442710489	3.28 (3.13 to 3.44)
182	Azerbaijan	YLDs (Years Lived with Disability)	Both	Prostate cancer	Age-standardized	2.437830678	2.126457812	2.750152888	2.44 (2.13 to 2.75)
183	Azerbaijan	YLLs (Years of Life Lost)	Both	Prostate cancer	All ages	1.551717215	1.366529253	1.737243501	1.55 (1.37 to 1.74)
184	Azerbaijan	YLLs (Years of Life Lost)	Both	Prostate cancer	Age-standardized	0.751347055	0.387738176	1.116272941	0.75 (0.39 to 1.12

)

185	Tonga	Deaths	Both	Prostate cancer	All ages	0.934934529	0.786009855	1.08407926	0.93 (0.79 to 1.08)
186	Tonga	Deaths	Both	Prostate cancer	Age-standardized	-0.332921259	-0.42574005	-0.240015947	-0.33 (-0.43 to -0.24)
187	Tonga	DALYs (Disability-Adjusted Life Years)	Both	Prostate cancer	All ages	0.726751516	0.596105479	0.857567226	0.73 (0.6 to 0.86)
188	Tonga	DALYs (Disability-Adjusted Life Years)	Both	Prostate cancer	Age-standardized	-0.273601313	-0.359539169	-0.187589338	-0.27 (-0.36 to -0.19)
189	Tonga	YLDs (Years Lived with Disability)	Both	Prostate cancer	All ages	1.51143218 8	1.35062728 7	1.67249222 6	1.51 (1.35 to 1.67)
190	Tonga	YLDs (Years Lived with Disability)	Both	Prostate cancer	Age-standardized	0.596670459	0.481586443	0.711886285	0.6 (0.48 to 0.71)
191	Tonga	YLLs (Years of Life Lost)	Both	Prostate cancer	All ages	0.694941131	0.565039944	0.825010113	0.69 (0.57 to 0.83)
192	Tonga	YLLs (Years of Life Lost)	Both	Prostate cancer	Age-standardized	-0.30826897	-0.393475686	-0.222989366	-0.31 (-0.39 to

									-0.22)
193	Kazakhstan	Deaths	Both	Prostate cancer	All ages	0.470695099	0.114282905	0.828376139	0.47 (0.11 to 0.83)
194	Kazakhstan	Deaths	Both	Prostate cancer	Age-standardized	0.041310902	-0.325020915	0.408989085	0.04 (-0.33 to 0.41)
195	Kazakhstan	DALYs (Disability-Adjusted Life Years)	Both	Prostate cancer	All ages	0.398315516	0.042077109	0.755822448	0.4 (0.04 to 0.76)
196	Kazakhstan	DALYs (Disability-Adjusted Life Years)	Both	Prostate cancer	Age-standardized	-0.082825462	-0.46939957	0.305250089	-0.08 (-0.47 to 0.31)
197	Kazakhstan	YLDs (Years Lived with Disability)	Both	Prostate cancer	All ages	2.475440079	2.177328666	2.774421258	2.48 (2.18 to 2.77)
198	Kazakhstan	YLDs (Years Lived with Disability)	Both	Prostate cancer	Age-standardized	1.953924993	1.578477022	2.330760671	1.95 (1.58 to 2.33)
199	Kazakhstan	YLLs (Years of Life Lost)	Both	Prostate cancer	All ages	0.294546653	-0.074641751	0.665099075	0.29 (-0.07 to 0.67)
200	Kazakhstan	YLLs (Years of Life Lost)	Both	Prostate cancer	Age-standardized	-0.183654013	-0.580290178	0.214564537	-0.18 (-0.58 to

									0.21)
201	Armenia	Deaths	Both	Prostate cancer	All ages	4.298994475	3.996446354	4.602422773	4.3 (4 to 4.6)
202	Armenia	Deaths	Both	Prostate cancer	Age-standardized	2.131209559	1.860925321	2.402210986	2.13 (1.86 to 2.4)
203	Armenia	DALYs (Disability-Adjusted Life Years)	Both	Prostate cancer	All ages	3.600852763	3.387666961	3.814478155	3.6 (3.39 to 3.81)
204	Armenia	DALYs (Disability-Adjusted Life Years)	Both	Prostate cancer	Age-standardized	1.820654751	1.590101124	2.051731608	1.82 (1.59 to 2.05)
205	Armenia	YLDs (Years Lived with Disability)	Both	Prostate cancer	All ages	5.205452717	4.985621712	5.425744029	5.21 (4.99 to 5.43)
206	Armenia	YLDs (Years Lived with Disability)	Both	Prostate cancer	Age-standardized	3.49482321	3.225283173	3.765067065	3.49 (3.23 to 3.77)
207	Armenia	YLLs (Years of Life Lost)	Both	Prostate cancer	All ages	3.51250514	3.298590862	3.726862399	3.51 (3.3 to 3.73)
208	Armenia	YLLs (Years of Life Lost)	Both	Prostate cancer	Age-standardized	1.729475452	1.500083258	1.959386077	1.73 (1.5 to 1.96)
209	Georgia	Deaths	Both	Prostate canc	All ages	6.684390222	5.87848483	7.496429849	6.68 (5.88 to

				er					7.5)
210	Georgia	Deaths	Both	Prostate cancer	Age-standardized	5.355539674	4.519099818	6.198673345	5.36 (4.52 to 6.2)
211	Georgia	DALYs (Disability-Adjusted Life Years)	Both	Prostate cancer	All ages	6.029786025	5.301501727	6.763107268	6.03 (5.3 to 6.76)
212	Georgia	DALYs (Disability-Adjusted Life Years)	Both	Prostate cancer	Age-standardized	4.934324256	4.189760791	5.68420854	4.93 (4.19 to 5.68)
213	Georgia	YLDs (Years Lived with Disability)	Both	Prostate cancer	All ages	6.237825255	5.636145117	6.84293243	6.24 (5.64 to 6.84)
214	Georgia	YLDs (Years Lived with Disability)	Both	Prostate cancer	Age-standardized	5.245701649	4.614173464	5.881042204	5.25 (4.61 to 5.88)
215	Georgia	YLLs (Years of Life Lost)	Both	Prostate cancer	All ages	6.018869369	5.283432695	6.759443291	6.02 (5.28 to 6.76)
216	Georgia	YLLs (Years of Life Lost)	Both	Prostate cancer	Age-standardized	4.917886837	4.167105689	5.674079217	4.92 (4.17 to 5.67)
217	Mongolia	Deaths	Both	Prostate cancer	All ages	3.465494062	3.173005526	3.758811783	3.47 (3.17 to 3.76)

218	Mongolia	Deaths	Both	Prostate cancer	Age-standardized	2.466216555	2.268166664	2.664649985	2.47 (2.27 to 2.66)
219	Mongolia	DALYs (Disability-Adjusted Life Years)	Both	Prostate cancer	All ages	3.703366499	3.425989066	3.981487828	3.7 (3.43 to 3.98)
220	Mongolia	DALYs (Disability-Adjusted Life Years)	Both	Prostate cancer	Age-standardized	2.433923241	2.225865254	2.642404684	2.43 (2.23 to 2.64)
221	Mongolia	YLDs (Years Lived with Disability)	Both	Prostate cancer	All ages	5.625676565	5.148617589	6.104899956	5.63 (5.15 to 6.1)
222	Mongolia	YLDs (Years Lived with Disability)	Both	Prostate cancer	Age-standardized	4.272720541	3.911544183	4.635152277	4.27 (3.91 to 4.64)
223	Mongolia	YLLs (Years of Life Lost)	Both	Prostate cancer	All ages	3.638089456	3.36676595	3.910125148	3.64 (3.37 to 3.91)
224	Mongolia	YLLs (Years of Life Lost)	Both	Prostate cancer	Age-standardized	2.37146988	2.166061805	2.577290935	2.37 (2.17 to 2.58)
225	Turkmenistan	Deaths	Both	Prostate cancer	All ages	1.249145358	0.834858535	1.665134306	1.25 (0.83 to 1.67)
226	Turkmenistan	Deaths	Both	Prostate cancer	Age-standardized	-0.046261805	-0.381046125	0.289647607	-0.05 (-0.38 to 0.29

									)
227	Turkmenistan	DALYs (Disability-Adjusted Life Years)	Both	Prostate cancer	All ages	1.387120983	0.998311351	1.777427401	1.39 (1 to 1.78)
228	Turkmenistan	DALYs (Disability-Adjusted Life Years)	Both	Prostate cancer	Age-standardized	0.0267000 3	-0.278112421	0.332444178	0.03 (-0.28 to 0.33)
229	Turkmenistan	YLDs (Years Lived with Disability)	Both	Prostate cancer	All ages	2.467117727	2.002080521	2.934275082	2.47 (2 to 2.93)
230	Turkmenistan	YLDs (Years Lived with Disability)	Both	Prostate cancer	Age-standardized	1.088489224	0.738235918	1.439960313	1.09 (0.74 to 1.44)
231	Turkmenistan	YLLs (Years of Life Lost)	Both	Prostate cancer	All ages	1.344721133	0.959138942	1.731775935	1.34 (0.96 to 1.73)
232	Turkmenistan	YLLs (Years of Life Lost)	Both	Prostate cancer	Age-standardized	-0.014839948	-0.317742963	0.288983495	-0.01 (-0.32 to 0.29)
233	Kyrgyzstan	Deaths	Both	Prostate cancer	All ages	1.620423611	1.132839763	2.110358209	1.62 (1.13 to 2.11)
234	Kyrgyzstan	Deaths	Both	Prostate cancer	Age-standardized	2.032583794	1.54668879	2.520803777	2.03 (1.55 to 2.52)

235	Kyrgyzstan	DALYs (Disability-Adjusted Life Years)	Both	Prostate cancer	All ages	1.535184569	1.087711407	1.984638508	1.54 (1.09 to 1.98)
236	Kyrgyzstan	DALYs (Disability-Adjusted Life Years)	Both	Prostate cancer	Age-standardized	1.710490784	1.317573044	2.104932291	1.71 (1.32 to 2.1)
237	Kyrgyzstan	YLDs (Years Lived with Disability)	Both	Prostate cancer	All ages	2.995470982	2.420900534	3.57326471	3 (2.42 to 3.57)
238	Kyrgyzstan	YLDs (Years Lived with Disability)	Both	Prostate cancer	Age-standardized	3.143374205	2.651927188	3.637174028	3.14 (2.65 to 3.64)
239	Kyrgyzstan	YLLs (Years of Life Lost)	Both	Prostate cancer	All ages	1.472864317	1.03099102	1.916670209	1.47 (1.03 to 1.92)
240	Kyrgyzstan	YLLs (Years of Life Lost)	Both	Prostate cancer	Age-standardized	1.650074875	1.261177201	2.040466126	1.65 (1.26 to 2.04)
241	Tajikistan	Deaths	Both	Prostate cancer	All ages	-2.919121102	-3.129493434	-2.708291907	-2.92 (-3.13 to -2.71)
242	Tajikistan	Deaths	Both	Prostate cancer	Age-standardized	-2.945558626	-3.23311612	-2.657146611	-2.95 (-3.23 to -2.66)

243	Tajikistan	DALYs (Disability-Adjusted Life Years)	Both	Prostate cancer	All ages	-2.527602 43	-2.716743 729	-2.338093 396	-2.53 (-2.72 to -2.34)
244	Tajikistan	DALYs (Disability-Adjusted Life Years)	Both	Prostate cancer	Age-standardized	-2.815048 126	-3.079251 749	-2.550124 29	-2.82 (-3.08 to -2.55)
245	Tajikistan	YLDs (Years Lived with Disability)	Both	Prostate cancer	All ages	-1.840620 759	-2.092280 729	-1.588313 927	-1.84 (-2.09 to -1.59)
246	Tajikistan	YLDs (Years Lived with Disability)	Both	Prostate cancer	Age-standardized	-2.093758 621	-2.303923 398	-1.883141 735	-2.09 (-2.3 to -1.88)
247	Tajikistan	YLLs (Years of Life Lost)	Both	Prostate cancer	All ages	-2.551275 584	-2.739049 76	-2.363138 887	-2.55 (-2.74 to -2.36)
248	Tajikistan	YLLs (Years of Life Lost)	Both	Prostate cancer	Age-standardized	-2.839834 323	-3.106525 558	-2.572409 042	-2.84 (-3.11 to -2.57)
249	Uzbekistan	Deaths	Both	Prostate cancer	All ages	1.7752079	1.0423380 23	2.5133933 54	1.78 (1.04 to 2.51)
250	Uzbekistan	Deaths	Both	Prostate canc	Age-standardized	0.9439846 32	0.2838060 02	1.6085092 87	0.94 (0.28 to

				er					1.61)
251	Uzbekistan	DALYs (Disability-Adjusted Life Years)	Both	Prostate cancer	All ages	1.922076361	1.210850316	2.638300315	1.92 (1.21 to 2.64)
252	Uzbekistan	DALYs (Disability-Adjusted Life Years)	Both	Prostate cancer	Age-standardized	0.848247181	0.20562368	1.494991857	0.85 (0.21 to 1.49)
253	Uzbekistan	YLDs (Years Lived with Disability)	Both	Prostate cancer	All ages	2.97755155	2.243488037	3.716885319	2.98 (2.24 to 3.72)
254	Uzbekistan	YLDs (Years Lived with Disability)	Both	Prostate cancer	Age-standardized	1.882897528	1.253369153	2.516339905	1.88 (1.25 to 2.52)
255	Uzbekistan	YLLs (Years of Life Lost)	Both	Prostate cancer	All ages	1.878638778	1.168538543	2.593723194	1.88 (1.17 to 2.59)
256	Uzbekistan	YLLs (Years of Life Lost)	Both	Prostate cancer	Age-standardized	0.80592357	0.16277754	1.453199245	0.81 (0.16 to 1.45)
257	Albania	Deaths	Both	Prostate cancer	All ages	4.192804899	4.05014346	4.335661938	4.19 (4.05 to 4.34)
258	Albania	Deaths	Both	Prostate cancer	Age-standardized	0.071751593	-0.057022512	0.20069162	0.07 (-0.06 to 0.2)

259	Albania	DALYs (Disability-Adjusted Life Years)	Both	Prostate cancer	All ages	3.848773967	3.712256887	3.985470746	3.85 (3.71 to 3.99)
260	Albania	DALYs (Disability-Adjusted Life Years)	Both	Prostate cancer	Age-standardized	-0.029184275	-0.155553 3	0.0973446 9	-0.03 (-0.16 to 0.1)
261	Albania	YLDs (Years Lived with Disability)	Both	Prostate cancer	All ages	6.392669979	6.107698654	6.678406646	6.39 (6.11 to 6.68)
262	Albania	YLDs (Years Lived with Disability)	Both	Prostate cancer	Age-standardized	2.459168154	2.242201377	2.676595353	2.46 (2.24 to 2.68)
263	Albania	YLLs (Years of Life Lost)	Both	Prostate cancer	All ages	3.71617814	3.581141741	3.851390583	3.72 (3.58 to 3.85)
264	Albania	YLLs (Years of Life Lost)	Both	Prostate cancer	Age-standardized	-0.155354809	-0.283356503	-0.027188805	-0.16 (-0.28 to -0.03)
265	Bulgaria	Deaths	Both	Prostate cancer	All ages	0.458267632	0.122736547	0.794923149	0.46 (0.12 to 0.79)
266	Bulgaria	Deaths	Both	Prostate cancer	Age-standardized	-0.947367254	-1.240011255	-0.653856094	-0.95 (-1.24 to -0.65)

267	Bulgaria	DALYs (Disability-Adjusted Life Years)	Both	Prostate cancer	All ages	0.318675923	-0.012376151	0.650824089	0.32 (-0.01 to 0.65)
268	Bulgaria	DALYs (Disability-Adjusted Life Years)	Both	Prostate cancer	Age-standardized	-0.775299342	-1.088231967	-0.461376674	-0.78 (-1.09 to -0.46)
269	Bulgaria	YLDs (Years Lived with Disability)	Both	Prostate cancer	All ages	2.153213125	1.89228504	2.4148094	2.15 (1.89 to 2.41)
270	Bulgaria	YLDs (Years Lived with Disability)	Both	Prostate cancer	Age-standardized	1.118932596	0.889048646	1.349340356	1.12 (0.89 to 1.35)
271	Bulgaria	YLLs (Years of Life Lost)	Both	Prostate cancer	All ages	0.203324881	-0.13480328	0.542597892	0.2 (-0.13 to 0.54)
272	Bulgaria	YLLs (Years of Life Lost)	Both	Prostate cancer	Age-standardized	-0.892094637	-1.212983705	-0.570163227	-0.89 (-1.21 to -0.57)
273	Czechia	Deaths	Both	Prostate cancer	All ages	0.389239939	0.188514694	0.590367331	0.39 (0.19 to 0.59)
274	Czechia	Deaths	Both	Prostate cancer	Age-standardized	-1.143711739	-1.401674739	-0.885073829	-1.14 (-1.4 to -0.89)

275	Czechia	DALYs (Disability-Adjusted Life Years)	Both	Prostate cancer	All ages	0.575820398	0.33160011	0.82063515 1	0.58 (0.33 to 0.82)
276	Czechia	DALYs (Disability-Adjusted Life Years)	Both	Prostate cancer	Age-standardized	-0.829230712	-1.113743371	-0.543899461	-0.83 (-1.11 to -0.54)
277	Czechia	YLDs (Years Lived with Disability)	Both	Prostate cancer	All ages	2.845273177	2.340602847	3.35243218	2.85 (2.34 to 3.35)
278	Czechia	YLDs (Years Lived with Disability)	Both	Prostate cancer	Age-standardized	1.475050993	0.936930325	2.01604052	1.48 (0.94 to 2.02)
279	Czechia	YLLs (Years of Life Lost)	Both	Prostate cancer	All ages	0.329131371	0.103530522	0.555240652	0.33 (0.1 to 0.56)
280	Czechia	YLLs (Years of Life Lost)	Both	Prostate cancer	Age-standardized	-1.081294405	-1.347640741	-0.814228975	-1.08 (-1.35 to -0.81)
281	Bosnia and Herzegovina	Deaths	Both	Prostate cancer	All ages	4.385451059	3.743269204	5.031608089	4.39 (3.74 to 5.03)
282	Bosnia and Herzegovina	Deaths	Both	Prostate cancer	Age-standardized	1.376244867	0.886328731	1.868540095	1.38 (0.89 to 1.87)

283	Bosnia and Herzegovina	DALYs (Disability-Adjusted Life Years)	Both	Prostate cancer	All ages	4.009500349	3.383770998	4.639016921	4.01 (3.38 to 4.64)
284	Bosnia and Herzegovina	DALYs (Disability-Adjusted Life Years)	Both	Prostate cancer	Age-standardized	1.51008315	0.997467552	2.025300543	1.51 (1 to 2.03)
285	Bosnia and Herzegovina	YLDs (Years Lived with Disability)	Both	Prostate cancer	All ages	6.228487582	5.381926002	7.08184982	6.23 (5.38 to 7.08)
286	Bosnia and Herzegovina	YLDs (Years Lived with Disability)	Both	Prostate cancer	Age-standardized	3.860947074	3.118674156	4.608563051	3.86 (3.12 to 4.61)
287	Bosnia and Herzegovina	YLLs (Years of Life Lost)	Both	Prostate cancer	All ages	3.878771533	3.262447355	4.498774255	3.88 (3.26 to 4.5)
288	Bosnia and Herzegovina	YLLs (Years of Life Lost)	Both	Prostate cancer	Age-standardized	1.373539729	0.870703824	1.878882249	1.37 (0.87 to 1.88)
289	Croatia	Deaths	Both	Prostate cancer	All ages	1.764158324	1.443262557	2.086069181	1.76 (1.44 to 2.09)
290	Croatia	Deaths	Both	Prostate cancer	Age-standardized	-0.789153859	-1.074838752	-0.50264394	-0.79 (-1.07 to -0.5)
291	Croatia	DALYs (Disability-Adjusted Life Years)	Both	Prostate cancer	All ages	1.469595851	1.164961987	1.775147046	1.47 (1.16 to 1.78)

292	Croatia	DALYs (Disability-Adjusted Life Years)	Both	Prostate cancer	Age-standardized	-0.52983502	-0.808135258	-0.250753963	-0.53 (-0.81 to -0.25)
293	Croatia	YLDs (Years Lived with Disability)	Both	Prostate cancer	All ages	3.299080182	2.947537072	3.651823733	3.3 (2.95 to 3.65)
294	Croatia	YLDs (Years Lived with Disability)	Both	Prostate cancer	Age-standardized	1.553742005	1.213825998	1.894799585	1.55 (1.21 to 1.89)
295	Croatia	YLLs (Years of Life Lost)	Both	Prostate cancer	All ages	1.302184651	0.999565731	1.605710291	1.3 (1 to 1.61)
296	Croatia	YLLs (Years of Life Lost)	Both	Prostate cancer	Age-standardized	-0.719942251	-0.993777179	-0.44534994	-0.72 (-0.99 to -0.45)
297	North Macedonia	Deaths	Both	Prostate cancer	All ages	1.502702977	1.275280902	1.730635748	1.5 (1.28 to 1.73)
298	North Macedonia	Deaths	Both	Prostate cancer	Age-standardized	-0.431952982	-0.773699099	-0.089029855	-0.43 (-0.77 to -0.09)
299	North Macedonia	DALYs (Disability-Adjusted Life Years)	Both	Prostate cancer	All ages	1.547119286	1.313279208	1.781499087	1.55 (1.31 to 1.78)

300	North Macedonia	DALYs (Disability-Adjusted Life Years)	Both	Prostate cancer	Age-standardized	-0.249395408	-0.511217671	0.013115886	-0.25 (-0.51 to 0.01)
301	North Macedonia	YLDs (Years Lived with Disability)	Both	Prostate cancer	All ages	3.778320749	3.430016245	4.127798182	3.78 (3.43 to 4.13)
302	North Macedonia	YLDs (Years Lived with Disability)	Both	Prostate cancer	Age-standardized	1.954101159	1.621527928	2.28776279	1.95 (1.62 to 2.29)
303	North Macedonia	YLLs (Years of Life Lost)	Both	Prostate cancer	All ages	1.412193449	1.179986386	1.644933424	1.41 (1.18 to 1.64)
304	North Macedonia	YLLs (Years of Life Lost)	Both	Prostate cancer	Age-standardized	-0.377086983	-0.639888636	-0.113590236	-0.38 (-0.64 to -0.11)
305	Poland	Deaths	Both	Prostate cancer	All ages	0.08015919	-0.245545577	0.406927404	0.08 (-0.25 to 0.41)
306	Poland	Deaths	Both	Prostate cancer	Age-standardized	-1.731325566	-2.089746328	-1.371592731	-1.73 (-2.09 to -1.37)
307	Poland	DALYs (Disability-Adjusted Life Years)	Both	Prostate cancer	All ages	-0.190989934	-0.473534692	0.09235693 8	-0.19 (-0.47 to 0.09)

308	Poland	DALYs (Disability-Adjusted Life Years)	Both	Prostate cancer	Age-standardized	-1.824081853	-2.132538583	-1.514652934	-1.82 (-2.13 to -1.51)
309	Poland	YLDs (Years Lived with Disability)	Both	Prostate cancer	All ages	2.382901257	2.121548803	2.644922573	2.38 (2.12 to 2.64)
310	Poland	YLDs (Years Lived with Disability)	Both	Prostate cancer	Age-standardized	0.798656523	0.476476106	1.12187002	0.8 (0.48 to 1.12)
311	Poland	YLLs (Years of Life Lost)	Both	Prostate cancer	All ages	-0.335384287	-0.62458424	-0.045342712	-0.34 (-0.62 to -0.05)
312	Poland	YLLs (Years of Life Lost)	Both	Prostate cancer	Age-standardized	-1.971263943	-2.285534833	-1.65598229	-1.97 (-2.29 to -1.66)
313	Hungary	Deaths	Both	Prostate cancer	All ages	-1.651107559	-1.930412441	-1.371007208	-1.65 (-1.93 to -1.37)
314	Hungary	Deaths	Both	Prostate cancer	Age-standardized	-2.930717737	-3.208228805	-2.652411019	-2.93 (-3.21 to -2.65)
315	Hungary	DALYs (Disability-Adjusted Life Years)	Both	Prostate cancer	All ages	-1.506312503	-1.761984371	-1.249975229	-1.51 (-1.76 to

									-1.25)
316	Hungary	DALYs (Disability-Adjusted Life Years)	Both	Prostate cancer	Age-standardized	-2.593650154	-2.847798812	-2.338836648	-2.59 (-2.85 to -2.34)
317	Hungary	YLDs (Years Lived with Disability)	Both	Prostate cancer	All ages	0.559998784	0.221356598	0.899785222	0.56 (0.22 to 0.9)
318	Hungary	YLDs (Years Lived with Disability)	Both	Prostate cancer	Age-standardized	-0.441082709	-0.787022044	-0.093937141	-0.44 (-0.79 to -0.09)
319	Hungary	YLLs (Years of Life Lost)	Both	Prostate cancer	All ages	-1.664436517	-1.918683758	-1.409530215	-1.66 (-1.92 to -1.41)
320	Hungary	YLLs (Years of Life Lost)	Both	Prostate cancer	Age-standardized	-2.759295648	-3.011741008	-2.506193212	-2.76 (-3.01 to -2.51)
321	Serbia	Deaths	Both	Prostate cancer	All ages	1.327042433	0.647733892	2.010935877	1.33 (0.65 to 2.01)
322	Serbia	Deaths	Both	Prostate cancer	Age-standardized	-0.85696513	-1.360436472	-0.350923994	-0.86 (-1.36 to -0.35)

323	Serbia	DALYs (Disability-Adjusted Life Years)	Both	Prostate cancer	All ages	1.005799646	0.36641996	1.649252471	1.01 (0.37 to 1.65)
324	Serbia	DALYs (Disability-Adjusted Life Years)	Both	Prostate cancer	Age-standardized	-0.67038174	-1.180486363	-0.157643967	-0.67 (-1.18 to -0.16)
325	Serbia	YLDs (Years Lived with Disability)	Both	Prostate cancer	All ages	3.133409401	2.495376313	3.775414242	3.13 (2.5 to 3.78)
326	Serbia	YLDs (Years Lived with Disability)	Both	Prostate cancer	Age-standardized	1.652154172	1.127263099	2.179769641	1.65 (1.13 to 2.18)
327	Serbia	YLLs (Years of Life Lost)	Both	Prostate cancer	All ages	0.867509067	0.224043878	1.515105475	0.87 (0.22 to 1.52)
328	Serbia	YLLs (Years of Life Lost)	Both	Prostate cancer	Age-standardized	-0.81818281	-1.332060949	-0.301628313	-0.82 (-1.33 to -0.3)
329	Slovenia	Deaths	Both	Prostate cancer	All ages	2.03087316	1.636032092	2.427248127	2.03 (1.64 to 2.43)
330	Slovenia	Deaths	Both	Prostate cancer	Age-standardized	-0.043276602	-0.448051775	0.363144376	-0.04 (-0.45 to 0.36)

331	Slovenia	DALYs (Disability-Adjusted Life Years)	Both	Prostate cancer	All ages	1.889073341	1.419452739	2.360868512	1.89 (1.42 to 2.36)
332	Slovenia	DALYs (Disability-Adjusted Life Years)	Both	Prostate cancer	Age-standardized	0.133621539	-0.337593284	0.607064317	0.13 (-0.34 to 0.61)
333	Slovenia	YLDs (Years Lived with Disability)	Both	Prostate cancer	All ages	4.645909957	3.993171233	5.302745756	4.65 (3.99 to 5.3)
334	Slovenia	YLDs (Years Lived with Disability)	Both	Prostate cancer	Age-standardized	3.143781751	2.47603826	3.815876321	3.14 (2.48 to 3.82)
335	Slovenia	YLLs (Years of Life Lost)	Both	Prostate cancer	All ages	1.598808271	1.136444296	2.063286029	1.6 (1.14 to 2.06)
336	Slovenia	YLLs (Years of Life Lost)	Both	Prostate cancer	Age-standardized	-0.194753532	-0.65898602	0.271648371	-0.19 (-0.66 to 0.27)
337	Montenegro	Deaths	Both	Prostate cancer	All ages	1.948808167	1.739765679	2.158280171	1.95 (1.74 to 2.16)
338	Montenegro	Deaths	Both	Prostate cancer	Age-standardized	0.167583928	-0.015628075	0.35113165	0.17 (-0.02 to 0.35)
339	Montenegro	DALYs (Disability-Adjusted Life Years)	Both	Prostate cancer	All ages	1.862460157	1.62561696	2.099855328	1.86 (1.63 to 2.1)

340	Montenegro	DALYs (Disability-Adjusted Life Years)	Both	Prostate cancer	Age-standardized	0.18286967	0.01032294 4	0.35571409	0.18 (0.01 to 0.36)
341	Montenegro	YLDs (Years Lived with Disability)	Both	Prostate cancer	All ages	3.27979391 8	3.12711856 6	3.4326953	3.28 (3.13 to 3.43)
342	Montenegro	YLDs (Years Lived with Disability)	Both	Prostate cancer	Age-standardized	1.59938039 3	1.45007533	1.74890518 9	1.6 (1.45 to 1.75)
343	Montenegro	YLLs (Years of Life Lost)	Both	Prostate cancer	All ages	1.74126172 9	1.49333042 4	1.98979868 9	1.74 (1.49 to 1.99)
344	Montenegro	YLLs (Years of Life Lost)	Both	Prostate cancer	Age-standardized	0.06384138 7	-0.11799801 2	0.24601183 1	0.06 (-0.12 to 0.25)
345	Romania	Deaths	Both	Prostate cancer	All ages	1.37838840 3	1.13683216 7	1.62052157 4	1.38 (1.14 to 1.62)
346	Romania	Deaths	Both	Prostate cancer	Age-standardized	-0.43667872 2	-0.67739887 5	-0.19537515 4	-0.44 (-0.68 to -0.2)
347	Romania	DALYs (Disability-Adjusted Life Years)	Both	Prostate cancer	All ages	1.2567302	1.01854395 9	1.49547804 8	1.26 (1.02 to 1.5)
348	Romania	DALYs (Disability-Adjusted Life Years)	Both	Prostate cancer	Age-standardized	-0.18918446 4	-0.41971849 4	0.04188326 6	-0.19 (-0.42 to 0.04

									)
349	Romania	YLDs (Years Lived with Disability)	Both	Prostate cancer	All ages	3.999116675	3.670420679	4.328854829	4 (3.67 to 4.33)
350	Romania	YLDs (Years Lived with Disability)	Both	Prostate cancer	Age-standardized	2.6519654	2.348081409	2.95675166	2.65 (2.35 to 2.96)
351	Romania	YLLs (Years of Life Lost)	Both	Prostate cancer	All ages	1.048863195	0.80898225	1.289314951	1.05 (0.81 to 1.29)
352	Romania	YLLs (Years of Life Lost)	Both	Prostate cancer	Age-standardized	-0.403516795	-0.63771813	-0.168763437	-0.4 (-0.64 to -0.17)
353	Belarus	Deaths	Both	Prostate cancer	All ages	2.408155605	2.121099763	2.696018343	2.41 (2.12 to 2.7)
354	Belarus	Deaths	Both	Prostate cancer	Age-standardized	1.391820862	1.104908613	1.679547301	1.39 (1.1 to 1.68)
355	Belarus	DALYs (Disability-Adjusted Life Years)	Both	Prostate cancer	All ages	2.474296958	2.150711854	2.79890709	2.47 (2.15 to 2.8)
356	Belarus	DALYs (Disability-Adjusted Life Years)	Both	Prostate cancer	Age-standardized	1.597090457	1.269446484	1.92579448	1.6 (1.27 to 1.93)
357	Belarus	YLDs (Years Lived with	Both	Prostate canc	All ages	4.655732716	4.300140503	5.012537255	4.66 (4.3 to

		Disability)		er					5.01)
358	Belarus	YLDs (Years Lived with Disability)	Both	Prostate cancer	Age-standardized	3.824764868	3.462364343	4.188434783	3.82 (3.46 to 4.19)
359	Belarus	YLLs (Years of Life Lost)	Both	Prostate cancer	All ages	2.237193674	1.910125891	2.56531114	2.24 (1.91 to 2.57)
360	Belarus	YLLs (Years of Life Lost)	Both	Prostate cancer	Age-standardized	1.35638575	1.025302848	1.688553686	1.36 (1.03 to 1.69)
361	Slovakia	Deaths	Both	Prostate cancer	All ages	1.08665583	0.86794108	1.305844825	1.09 (0.87 to 1.31)
362	Slovakia	Deaths	Both	Prostate cancer	Age-standardized	-0.459438582	-0.586771755	-0.331942316	-0.46 (-0.59 to -0.33)
363	Slovakia	DALYs (Disability-Adjusted Life Years)	Both	Prostate cancer	All ages	1.217152188	0.98519812	1.449639034	1.22 (0.99 to 1.45)
364	Slovakia	DALYs (Disability-Adjusted Life Years)	Both	Prostate cancer	Age-standardized	-0.301993601	-0.428577291	-0.175248987	-0.3 (-0.43 to -0.18)
365	Slovakia	YLDs (Years Lived with Disability)	Both	Prostate cancer	All ages	3.337487992	3.095117179	3.580428605	3.34 (3.1 to 3.58)

366	Slovakia	YLDs (Years Lived with Disability)	Both	Prostate cancer	Age-standardized	1.795836628	1.630575797	1.961366188	1.8 (1.63 to 1.96)
367	Slovakia	YLLs (Years of Life Lost)	Both	Prostate cancer	All ages	1.039439014	0.811162444	1.268232494	1.04 (0.81 to 1.27)
368	Slovakia	YLLs (Years of Life Lost)	Both	Prostate cancer	Age-standardized	-0.476505715	-0.599154651	-0.353705445	-0.48 (-0.6 to -0.35)
369	Latvia	Deaths	Both	Prostate cancer	All ages	2.883706293	2.469739601	3.299345365	2.88 (2.47 to 3.3)
370	Latvia	Deaths	Both	Prostate cancer	Age-standardized	1.230783058	0.849096066	1.613914635	1.23 (0.85 to 1.61)
371	Latvia	DALYs (Disability-Adjusted Life Years)	Both	Prostate cancer	All ages	2.532086632	2.118730347	2.9471161	2.53 (2.12 to 2.95)
372	Latvia	DALYs (Disability-Adjusted Life Years)	Both	Prostate cancer	Age-standardized	1.238548786	0.853055441	1.625515612	1.24 (0.85 to 1.63)
373	Latvia	YLDs (Years Lived with Disability)	Both	Prostate cancer	All ages	3.742099038	3.38596475	4.099460103	3.74 (3.39 to 4.1)
374	Latvia	YLDs (Years Lived with Disability)	Both	Prostate cancer	Age-standardized	2.587792329	2.247910555	2.928803902	2.59 (2.25 to 2.93)

375	Latvia	YLLs (Years of Life Lost)	Both	Prostate cancer	All ages	2.418342422	1.995947239	2.842486868	2.42 (2 to 2.84)
376	Latvia	YLLs (Years of Life Lost)	Both	Prostate cancer	Age-standardized	1.110025866	0.715400381	1.506197583	1.11 (0.72 to 1.51)
377	Estonia	Deaths	Both	Prostate cancer	All ages	2.697046832	1.760443569	3.642270593	2.7 (1.76 to 3.64)
378	Estonia	Deaths	Both	Prostate cancer	Age-standardized	1.129325996	0.253992782	2.01230188	1.13 (0.25 to 2.01)
379	Estonia	DALYs (Disability-Adjusted Life Years)	Both	Prostate cancer	All ages	2.455571355	1.547741697	3.371516947	2.46 (1.55 to 3.37)
380	Estonia	DALYs (Disability-Adjusted Life Years)	Both	Prostate cancer	Age-standardized	1.221428719	0.379557604	2.070360505	1.22 (0.38 to 2.07)
381	Estonia	YLDs (Years Lived with Disability)	Both	Prostate cancer	All ages	4.988428994	4.058340935	5.926830311	4.99 (4.06 to 5.93)
382	Estonia	YLDs (Years Lived with Disability)	Both	Prostate cancer	Age-standardized	3.839513608	2.971768781	4.714570935	3.84 (2.97 to 4.71)
383	Estonia	YLLs (Years of Life Lost)	Both	Prostate cancer	All ages	2.11979323	1.20146322	3.046456419	2.12 (1.2 to 3.05)

384	Estonia	YLLs (Years of Life Lost)	Both	Prostate cancer	Age-standardized	0.871812944	0.018155616	1.732756258	0.87 (0.02 to 1.73)
385	Lithuania	Deaths	Both	Prostate cancer	All ages	3.239321322	2.652733884	3.829260691	3.24 (2.65 to 3.83)
386	Lithuania	Deaths	Both	Prostate cancer	Age-standardized	1.028469104	0.482189441	1.577718661	1.03 (0.48 to 1.58)
387	Lithuania	DALYs (Disability-Adjusted Life Years)	Both	Prostate cancer	All ages	3.187030911	2.569115299	3.808669082	3.19 (2.57 to 3.81)
388	Lithuania	DALYs (Disability-Adjusted Life Years)	Both	Prostate cancer	Age-standardized	1.410943784	0.836590841	1.988568172	1.41 (0.84 to 1.99)
389	Lithuania	YLDs (Years Lived with Disability)	Both	Prostate cancer	All ages	4.208647643	3.587939236	4.833075392	4.21 (3.59 to 4.83)
390	Lithuania	YLDs (Years Lived with Disability)	Both	Prostate cancer	Age-standardized	2.572840404	1.997009595	3.151922103	2.57 (2 to 3.15)
391	Lithuania	YLLs (Years of Life Lost)	Both	Prostate cancer	All ages	3.025750156	2.40450139	3.6507678	3.03 (2.4 to 3.65)
392	Lithuania	YLLs (Years of Life Lost)	Both	Prostate cancer	Age-standardized	1.225745858	0.647438712	1.807375882	1.23 (0.65 to 1.81)

393	Russian Federation	Deaths	Both	Prostate cancer	All ages	3.805911895	3.325927577	4.288125905	3.81 (3.33 to 4.29)
394	Russian Federation	Deaths	Both	Prostate cancer	Age-standardized	2.858884768	2.367909341	3.352215005	2.86 (2.37 to 3.35)
395	Russian Federation	DALYs (Disability-Adjusted Life Years)	Both	Prostate cancer	All ages	3.525393378	3.087773709	3.964870794	3.53 (3.09 to 3.96)
396	Russian Federation	DALYs (Disability-Adjusted Life Years)	Both	Prostate cancer	Age-standardized	2.625386701	2.141656526	3.111407763	2.63 (2.14 to 3.11)
397	Russian Federation	YLDs (Years Lived with Disability)	Both	Prostate cancer	All ages	5.536489787	5.274754206	5.798876099	5.54 (5.27 to 5.8)
398	Russian Federation	YLDs (Years Lived with Disability)	Both	Prostate cancer	Age-standardized	4.642848241	4.302136623	4.984672821	4.64 (4.3 to 4.98)
399	Russian Federation	YLLs (Years of Life Lost)	Both	Prostate cancer	All ages	3.31905332	2.852663076	3.787558432	3.32 (2.85 to 3.79)
400	Russian Federation	YLLs (Years of Life Lost)	Both	Prostate cancer	Age-standardized	2.419750773	1.911084191	2.930956252	2.42 (1.91 to 2.93)
401	Republic of Moldova	Deaths	Both	Prostate cancer	All ages	4.842824386	4.251999888	5.436997246	4.84 (4.25 to 5.44)

402	Republic of Moldova	Deaths	Both	Prostate cancer	Age-standardized	3.017251543	2.427323783	3.610576979	3.02 (2.43 to 3.61)
403	Republic of Moldova	DALYs (Disability-Adjusted Life Years)	Both	Prostate cancer	All ages	4.87904709	4.30981590 6	5.45138463 7	4.88 (4.31 to 5.45)
404	Republic of Moldova	DALYs (Disability-Adjusted Life Years)	Both	Prostate cancer	Age-standardized	3.216355474	2.673243109	3.76234075	3.22 (2.67 to 3.76)
405	Republic of Moldova	YLDs (Years Lived with Disability)	Both	Prostate cancer	All ages	6.471906166	5.788397908	7.159830631	6.47 (5.79 to 7.16)
406	Republic of Moldova	YLDs (Years Lived with Disability)	Both	Prostate cancer	Age-standardized	4.836213237	4.201799028	5.474489965	4.84 (4.2 to 5.47)
407	Republic of Moldova	YLLs (Years of Life Lost)	Both	Prostate cancer	All ages	4.749431067	4.188342095	5.313541691	4.75 (4.19 to 5.31)
408	Republic of Moldova	YLLs (Years of Life Lost)	Both	Prostate cancer	Age-standardized	3.085787935	2.548666829	3.62572233	3.09 (2.55 to 3.63)
409	Brunei Darussalam	Deaths	Both	Prostate cancer	All ages	-0.026008308	-0.347654859	0.296676418	-0.03 (-0.35 to 0.3)
410	Brunei Darussalam	Deaths	Both	Prostate cancer	Age-standardized	-1.785475865	-1.935520809	-1.635201343	-1.79 (-1.94 to

									-1.64)
411	Brunei Darussalam	DALYs (Disability-Adjusted Life Years)	Both	Prostate cancer	All ages	0.120913453	-0.230028868	0.473090219	0.12 (-0.23 to 0.47)
412	Brunei Darussalam	DALYs (Disability-Adjusted Life Years)	Both	Prostate cancer	Age-standardized	-2.075099382	-2.209256137	-1.940758581	-2.08 (-2.21 to -1.94)
413	Brunei Darussalam	YLDs (Years Lived with Disability)	Both	Prostate cancer	All ages	1.859980238	1.626556251	2.093940373	1.86 (1.63 to 2.09)
414	Brunei Darussalam	YLDs (Years Lived with Disability)	Both	Prostate cancer	Age-standardized	-0.554180517	-0.689427586	-0.418749261	-0.55 (-0.69 to -0.42)
415	Brunei Darussalam	YLLs (Years of Life Lost)	Both	Prostate cancer	All ages	0.035564207	-0.31983911	0.392234692	0.04 (-0.32 to 0.39)
416	Brunei Darussalam	YLLs (Years of Life Lost)	Both	Prostate cancer	Age-standardized	-2.14936547	-2.287386899	-2.011149081	-2.15 (-2.29 to -2.01)
417	Ukraine	Deaths	Both	Prostate cancer	All ages	0.190864987	-0.097726284	0.480289922	0.19 (-0.1 to 0.48)

418	Ukraine	Deaths	Both	Prostate cancer	Age-standardized	-0.648434967	-0.890213044	-0.406067074	-0.65 (-0.89 to -0.41)
419	Ukraine	DALYs (Disability-Adjusted Life Years)	Both	Prostate cancer	All ages	0.170769357	-0.106320499	0.448627819	0.17 (-0.11 to 0.45)
420	Ukraine	DALYs (Disability-Adjusted Life Years)	Both	Prostate cancer	Age-standardized	-0.521326742	-0.777728282	-0.264262632	-0.52 (-0.78 to -0.26)
421	Ukraine	YLDs (Years Lived with Disability)	Both	Prostate cancer	All ages	0.85723595	0.64243828 6	1.07249205	0.86 (0.64 to 1.07)
422	Ukraine	YLDs (Years Lived with Disability)	Both	Prostate cancer	Age-standardized	0.182108021	-0.017369338	0.381983362	0.18 (-0.02 to 0.38)
423	Ukraine	YLLs (Years of Life Lost)	Both	Prostate cancer	All ages	0.132774483	-0.148512998	0.414854367	0.13 (-0.15 to 0.41)
424	Ukraine	YLLs (Years of Life Lost)	Both	Prostate cancer	Age-standardized	-0.559805389	-0.820490796	-0.298434791	-0.56 (-0.82 to -0.3)
425	Republic of Korea	Deaths	Both	Prostate cancer	All ages	3.243513518	3.00883309 2	3.478728605	3.24 (3.01 to 3.48)

426	Republic of Korea	Deaths	Both	Prostate cancer	Age-standardized	-0.866323237	-1.171519178	-0.560184809	-0.87 (-1.17 to -0.56)
427	Republic of Korea	DALYs (Disability-Adjusted Life Years)	Both	Prostate cancer	All ages	2.814044269	2.565424976	3.063266216	2.81 (2.57 to 3.06)
428	Republic of Korea	DALYs (Disability-Adjusted Life Years)	Both	Prostate cancer	Age-standardized	-0.860589255	-1.142367408	-0.578007937	-0.86 (-1.14 to -0.58)
429	Republic of Korea	YLDs (Years Lived with Disability)	Both	Prostate cancer	All ages	7.335227162	6.652664724	8.022157907	7.34 (6.65 to 8.02)
430	Republic of Korea	YLDs (Years Lived with Disability)	Both	Prostate cancer	Age-standardized	3.476039768	2.825486081	4.130709363	3.48 (2.83 to 4.13)
431	Republic of Korea	YLLs (Years of Life Lost)	Both	Prostate cancer	All ages	2.341899759	2.106776867	2.577564073	2.34 (2.11 to 2.58)
432	Republic of Korea	YLLs (Years of Life Lost)	Both	Prostate cancer	Age-standardized	-1.307365737	-1.583355068	-1.030602449	-1.31 (-1.58 to -1.03)
433	Japan	Deaths	Both	Prostate cancer	All ages	1.224849384	0.902407876	1.54832128	1.22 (0.9 to 1.55)

434	Japan	Deaths	Both	Prostate cancer	Age-standardized	-2.042049306	-2.311364608	-1.771991536	-2.04 (-2.31 to -1.77)
435	Japan	DALYs (Disability-Adjusted Life Years)	Both	Prostate cancer	All ages	0.764108809	0.396457646	1.133106308	0.76 (0.4 to 1.13)
436	Japan	DALYs (Disability-Adjusted Life Years)	Both	Prostate cancer	Age-standardized	-1.802983514	-2.087994926	-1.517142465	-1.8 (-2.09 to -1.52)
437	Japan	YLDs (Years Lived with Disability)	Both	Prostate cancer	All ages	3.435184858	2.737532754	4.137574457	3.44 (2.74 to 4.14)
438	Japan	YLDs (Years Lived with Disability)	Both	Prostate cancer	Age-standardized	1.005698664	0.444323801	1.570211004	1.01 (0.44 to 1.57)
439	Japan	YLLs (Years of Life Lost)	Both	Prostate cancer	All ages	0.395090613	0.051907923	0.739450436	0.4 (0.05 to 0.74)
440	Japan	YLLs (Years of Life Lost)	Both	Prostate cancer	Age-standardized	-2.199690297	-2.474600293	-1.92400537	-2.2 (-2.47 to -1.92)
441	Singapore	Deaths	Both	Prostate cancer	All ages	0.444752697	0.039699448	0.851445976	0.44 (0.04 to 0.85)

442	Singapore	Deaths	Both	Prostate cancer	Age-standardized	-2.049446372	-2.262226108	-1.836203406	-2.05 (-2.26 to -1.84)
443	Singapore	DALYs (Disability-Adjusted Life Years)	Both	Prostate cancer	All ages	0.536447261	0.202284508	0.871724407	0.54 (0.2 to 0.87)
444	Singapore	DALYs (Disability-Adjusted Life Years)	Both	Prostate cancer	Age-standardized	-1.899122389	-2.073172979	-1.72476245	-1.9 (-2.07 to -1.72)
445	Singapore	YLDs (Years Lived with Disability)	Both	Prostate cancer	All ages	3.985490408	3.799719242	4.17159405	3.99 (3.8 to 4.17)
446	Singapore	YLDs (Years Lived with Disability)	Both	Prostate cancer	Age-standardized	1.420549242	1.159524562	1.682247451	1.42 (1.16 to 1.68)
447	Singapore	YLLs (Years of Life Lost)	Both	Prostate cancer	All ages	0.131786902	-0.216712228	0.481503187	0.13 (-0.22 to 0.48)
448	Singapore	YLLs (Years of Life Lost)	Both	Prostate cancer	Age-standardized	-2.287406096	-2.473676251	-2.100780174	-2.29 (-2.47 to -2.1)
449	New Zealand	Deaths	Both	Prostate cancer	All ages	-1.394549456	-1.658727337	-1.129661904	-1.39 (-1.66 to -1.13)

450	New Zealand	Deaths	Both	Prostate cancer	Age-standardized	-2.670736383	-2.907793566	-2.433100408	-2.67 (-2.91 to -2.43)
451	New Zealand	DALYs (Disability-Adjusted Life Years)	Both	Prostate cancer	All ages	-1.638609811	-1.888045428	-1.388540039	-1.64 (-1.89 to -1.39)
452	New Zealand	DALYs (Disability-Adjusted Life Years)	Both	Prostate cancer	Age-standardized	-2.843985745	-3.05781831	-2.629681515	-2.84 (-3.06 to -2.63)
453	New Zealand	YLDs (Years Lived with Disability)	Both	Prostate cancer	All ages	-0.764017882	-0.976934686	-0.550643269	-0.76 (-0.98 to -0.55)
454	New Zealand	YLDs (Years Lived with Disability)	Both	Prostate cancer	Age-standardized	-1.929890407	-2.126288583	-1.733098128	-1.93 (-2.13 to -1.73)
455	New Zealand	YLLs (Years of Life Lost)	Both	Prostate cancer	All ages	-1.82260323	-2.080009848	-1.564519957	-1.82 (-2.08 to -1.56)
456	New Zealand	YLLs (Years of Life Lost)	Both	Prostate cancer	Age-standardized	-3.040644576	-3.261119983	-2.819666688	-3.04 (-3.26 to -2.82)

457	Australia	Deaths	Both	Prostate cancer	All ages	-3.129806089	-3.602822653	-2.654468454	-3.13 (-3.6 to -2.65)
458	Australia	Deaths	Both	Prostate cancer	Age-standardized	-4.553921004	-5.033951185	-4.071464387	-4.55 (-5.03 to -4.07)
459	Australia	DALYs (Disability-Adjusted Life Years)	Both	Prostate cancer	All ages	-3.085974353	-3.656480277	-2.512090132	-3.09 (-3.66 to -2.51)
460	Australia	DALYs (Disability-Adjusted Life Years)	Both	Prostate cancer	Age-standardized	-4.288417206	-4.878592987	-3.69457971	-4.29 (-4.88 to -3.69)
461	Australia	YLDs (Years Lived with Disability)	Both	Prostate cancer	All ages	-0.656621612	-1.571203418	0.266458315	-0.66 (-1.57 to 0.27)
462	Australia	YLDs (Years Lived with Disability)	Both	Prostate cancer	Age-standardized	-1.7281398	-2.6738572	-0.773232871	-1.73 (-2.67 to -0.77)
463	Australia	YLLs (Years of Life Lost)	Both	Prostate cancer	All ages	-3.599918067	-4.11372584	-3.083357049	-3.6 (-4.11 to -3.08)

464	Australia	YLLs (Years of Life Lost)	Both	Prostate cancer	Age-standardized	-4.848906282	-5.380753437	-4.314069656	-4.85 (-5.38 to -4.31)
465	Andorra	Deaths	Both	Prostate cancer	All ages	0.313339084	0.074250023	0.552999356	0.31 (0.07 to 0.55)
466	Andorra	Deaths	Both	Prostate cancer	Age-standardized	-1.553913544	-1.740592273	-1.366880153	-1.55 (-1.74 to -1.37)
467	Andorra	DALYs (Disability-Adjusted Life Years)	Both	Prostate cancer	All ages	0.270985985	0.036306873	0.50621564	0.27 (0.04 to 0.51)
468	Andorra	DALYs (Disability-Adjusted Life Years)	Both	Prostate cancer	Age-standardized	-1.280949076	-1.460074271	-1.101498268	-1.28 (-1.46 to -1.1)
469	Andorra	YLDs (Years Lived with Disability)	Both	Prostate cancer	All ages	1.991461742	1.672485087	2.311439121	1.99 (1.67 to 2.31)
470	Andorra	YLDs (Years Lived with Disability)	Both	Prostate cancer	Age-standardized	0.589377681	0.301148858	0.878434768	0.59 (0.3 to 0.88)
471	Andorra	YLLs (Years of Life Lost)	Both	Prostate cancer	All ages	-0.009549429	-0.245215998	0.22667389 2	-0.01 (-0.25 to 0.23)

472	Andorra	YLLs (Years of Life Lost)	Both	Prostate cancer	Age-standardized	-1.589124008	-1.775656864	-1.402236917	-1.59 (-1.78 to -1.4)
473	Austria	Deaths	Both	Prostate cancer	All ages	0.014391198	-0.21115643	0.240448621	0.01 (-0.21 to 0.24)
474	Austria	Deaths	Both	Prostate cancer	Age-standardized	-1.086931176	-1.352083944	-0.821065711	-1.09 (-1.35 to -0.82)
475	Austria	DALYs (Disability-Adjusted Life Years)	Both	Prostate cancer	All ages	0.102693913	-0.201335919	0.407649952	0.1 (-0.2 to 0.41)
476	Austria	DALYs (Disability-Adjusted Life Years)	Both	Prostate cancer	Age-standardized	-0.80901035	-1.147940964	-0.468917657	-0.81 (-1.15 to -0.47)
477	Austria	YLDs (Years Lived with Disability)	Both	Prostate cancer	All ages	1.897610785	1.251498008	2.54784658	1.9 (1.25 to 2.55)
478	Austria	YLDs (Years Lived with Disability)	Both	Prostate cancer	Age-standardized	1.105039247	0.425299901	1.789379481	1.11 (0.43 to 1.79)
479	Austria	YLLs (Years of Life Lost)	Both	Prostate cancer	All ages	-0.241222668	-0.490959288	0.009140713	-0.24 (-0.49 to 0.01)

480	Austria	YLLs (Years of Life Lost)	Both	Prostate cancer	Age-standardized	-1.20565993	-1.488039053	-0.922471383	-1.21 (-1.49 to -0.92)
481	Belgium	Deaths	Both	Prostate cancer	All ages	-2.497198476	-2.738099643	-2.255700637	-2.5 (-2.74 to -2.26)
482	Belgium	Deaths	Both	Prostate cancer	Age-standardized	-3.58447471	-3.852262489	-3.315941097	-3.58 (-3.85 to -3.32)
483	Belgium	DALYs (Disability-Adjusted Life Years)	Both	Prostate cancer	All ages	-2.502799967	-2.849997204	-2.154361906	-2.5 (-2.85 to -2.15)
484	Belgium	DALYs (Disability-Adjusted Life Years)	Both	Prostate cancer	Age-standardized	-3.257028424	-3.641922022	-2.870597404	-3.26 (-3.64 to -2.87)
485	Belgium	YLDs (Years Lived with Disability)	Both	Prostate cancer	All ages	-0.705125351	-1.211906265	-0.195744661	-0.71 (-1.21 to -0.2)
486	Belgium	YLDs (Years Lived with Disability)	Both	Prostate cancer	Age-standardized	-1.216872888	-1.774288602	-0.656293926	-1.22 (-1.77 to -0.66)
487	Belgium	YLLs (Years of Life Lost)	Both	Prostate cancer	All ages	-2.747039486	-3.079749795	-2.413187041	-2.75 (-3.08 to -2.4

									1)
488	Belgium	YLLs (Years of Life Lost)	Both	Prostate cancer	Age-standardized	-3.548888255	-3.918144389	-3.178213018	-3.55 (-3.92 to -3.18)
489	Cyprus	Deaths	Both	Prostate cancer	All ages	0.160573327	-0.004159957	0.325577992	0.16 (0 to 0.33)
490	Cyprus	Deaths	Both	Prostate cancer	Age-standardized	-1.986654939	-2.177640499	-1.795296504	-1.99 (-2.18 to -1.8)
491	Cyprus	DALYs (Disability-Adjusted Life Years)	Both	Prostate cancer	All ages	0.573056228	0.456651194	0.689596147	0.57 (0.46 to 0.69)
492	Cyprus	DALYs (Disability-Adjusted Life Years)	Both	Prostate cancer	Age-standardized	-1.121272825	-1.24992093	-0.992457122	-1.12 (-1.25 to -0.99)
493	Cyprus	YLDs (Years Lived with Disability)	Both	Prostate cancer	All ages	3.915195057	3.592818043	4.238575295	3.92 (3.59 to 4.24)
494	Cyprus	YLDs (Years Lived with Disability)	Both	Prostate cancer	Age-standardized	2.536263693	2.178200486	2.895581662	2.54 (2.18 to 2.9)
495	Cyprus	YLLs (Years of Life Lost)	Both	Prostate cancer	All ages	0.106008394	-0.014684454	0.226846931	0.11 (-0.01 to 0.23)

496	Cyprus	YLLs (Years of Life Lost)	Both	Prostate cancer	Age-standardized	-1.604350339	-1.729973391	-1.478566696	-1.6 (-1.73 to -1.48)
497	Finland	Deaths	Both	Prostate cancer	All ages	0.267979843	0.049304794	0.487132845	0.27 (0.05 to 0.49)
498	Finland	Deaths	Both	Prostate cancer	Age-standardized	-1.554659932	-1.842272636	-1.266204491	-1.55 (-1.84 to -1.27)
499	Finland	DALYs (Disability-Adjusted Life Years)	Both	Prostate cancer	All ages	0.307695747	-0.030837324	0.647375217	0.31 (-0.03 to 0.65)
500	Finland	DALYs (Disability-Adjusted Life Years)	Both	Prostate cancer	Age-standardized	-1.290835415	-1.67968955	-0.900443373	-1.29 (-1.68 to -0.9)
501	Finland	YLDs (Years Lived with Disability)	Both	Prostate cancer	All ages	3.211175217	2.43756713	3.99062559	3.21 (2.44 to 3.99)
502	Finland	YLDs (Years Lived with Disability)	Both	Prostate cancer	Age-standardized	1.772779649	0.951522935	2.600717416	1.77 (0.95 to 2.6)
503	Finland	YLLs (Years of Life Lost)	Both	Prostate cancer	All ages	-0.202511185	-0.489299904	0.085104056	-0.2 (-0.49 to 0.09)
504	Finland	YLLs (Years of Life Lost)	Both	Prostate cancer	Age-standardized	-1.843529084	-2.183339249	-1.502538436	-1.84 (-2.18 to

-1.5)

505	Denmark	Deaths	Both	Prostate cancer	All ages	-0.923583242	-1.141956249	-0.704727858	-0.92 (-1.14 to -0.7)
506	Denmark	Deaths	Both	Prostate cancer	Age-standardized	-1.817344411	-2.122884088	-1.51085094	-1.82 (-2.12 to -1.51)
507	Denmark	DALYs (Disability-Adjusted Life Years)	Both	Prostate cancer	All ages	-0.964191852	-1.250033099	-0.67752321	-0.96 (-1.25 to -0.68)
508	Denmark	DALYs (Disability-Adjusted Life Years)	Both	Prostate cancer	Age-standardized	-1.912617067	-2.293236088	-1.530515335	-1.91 (-2.29 to -1.53)
509	Denmark	YLDs (Years Lived with Disability)	Both	Prostate cancer	All ages	2.264405519	1.565715487	2.967901973	2.26 (1.57 to 2.97)
510	Denmark	YLDs (Years Lived with Disability)	Both	Prostate cancer	Age-standardized	1.404013835	0.609770453	2.20452721	1.4 (0.61 to 2.2)
511	Denmark	YLLs (Years of Life Lost)	Both	Prostate cancer	All ages	-1.246064038	-1.505017094	-0.986430169	-1.25 (-1.51 to -0.99)

512	Denmark	YLLs (Years of Life Lost)	Both	Prostate cancer	Age-standardized	-2.207782504	-2.561282062	-1.853000479	-2.21 (-2.56 to -1.85)
513	France	Deaths	Both	Prostate cancer	All ages	-2.387272616	-2.581936542	-2.192219706	-2.39 (-2.58 to -2.19)
514	France	Deaths	Both	Prostate cancer	Age-standardized	-3.844098043	-4.056955379	-3.630768465	-3.84 (-4.06 to -3.63)
515	France	DALYs (Disability-Adjusted Life Years)	Both	Prostate cancer	All ages	-2.096818122	-2.263102355	-1.930250982	-2.1 (-2.26 to -1.93)
516	France	DALYs (Disability-Adjusted Life Years)	Both	Prostate cancer	Age-standardized	-3.189439186	-3.357123055	-3.021464371	-3.19 (-3.36 to -3.02)
517	France	YLDs (Years Lived with Disability)	Both	Prostate cancer	All ages	1.07364974	0.86597227 1	1.28175480 5	1.07 (0.87 to 1.28)
518	France	YLDs (Years Lived with Disability)	Both	Prostate cancer	Age-standardized	0.20491815 9	-0.046519452	0.45698827 3	0.2 (-0.05 to 0.46)
519	France	YLLs (Years of Life Lost)	Both	Prostate cancer	All ages	-2.641190817	-2.835223069	-2.446771094	-2.64 (-2.84 to -2.4

									5)
520	France	YLLs (Years of Life Lost)	Both	Prostate cancer	Age-standardized	-3.823932491	-4.020673492	-3.626788206	-3.82 (-4.02 to -3.63)
521	Germany	Deaths	Both	Prostate cancer	All ages	-1.242652373	-1.497544923	-0.987100243	-1.24 (-1.5 to -0.99)
522	Germany	Deaths	Both	Prostate cancer	Age-standardized	-2.726637588	-2.996240732	-2.456285134	-2.73 (-3 to -2.46)
523	Germany	DALYs (Disability-Adjusted Life Years)	Both	Prostate cancer	All ages	-1.130824736	-1.348289438	-0.912880662	-1.13 (-1.35 to -0.91)
524	Germany	DALYs (Disability-Adjusted Life Years)	Both	Prostate cancer	Age-standardized	-2.308248766	-2.545134611	-2.070787118	-2.31 (-2.55 to -2.07)
525	Germany	YLDs (Years Lived with Disability)	Both	Prostate cancer	All ages	0.922519059	0.409905002	1.437750121	0.92 (0.41 to 1.44)
526	Germany	YLDs (Years Lived with Disability)	Both	Prostate cancer	Age-standardized	-0.057327869	-0.563738413	0.4516617 3	-0.06 (-0.56 to 0.45

									)
527	Germany	YLLs (Years of Life Lost)	Both	Prostate cancer	All ages	-1.466178577	-1.68352662	-1.248350043	-1.47 (-1.68 to -1.25)
528	Germany	YLLs (Years of Life Lost)	Both	Prostate cancer	Age-standardized	-2.692904184	-2.933457017	-2.451755207	-2.69 (-2.93 to -2.45)
529	Iceland	Deaths	Both	Prostate cancer	All ages	-1.047029704	-1.240397064	-0.853283738	-1.05 (-1.24 to -0.85)
530	Iceland	Deaths	Both	Prostate cancer	Age-standardized	-2.347136229	-2.500699901	-2.193330691	-2.35 (-2.50 to -2.19)
531	Iceland	DALYs (Disability-Adjusted Life Years)	Both	Prostate cancer	All ages	-1.260489011	-1.457989074	-1.062593114	-1.26 (-1.46 to -1.06)
532	Iceland	DALYs (Disability-Adjusted Life Years)	Both	Prostate cancer	Age-standardized	-2.480394056	-2.6390133	-2.321516391	-2.48 (-2.64 to -2.32)
533	Iceland	YLDs (Years Lived with Disability)	Both	Prostate cancer	All ages	0.038851983	-0.191760038	0.269996845	0.04 (-0.19 to 0.27)

534	Iceland	YLDs (Years Lived with Disability)	Both	Prostate cancer	Age-standardized	-1.126220005	-1.392974367	-0.858744012	-1.13 (-1.39 to -0.86)
535	Iceland	YLLs (Years of Life Lost)	Both	Prostate cancer	All ages	-1.473072113	-1.679514725	-1.266196035	-1.47 (-1.68 to -1.27)
536	Iceland	YLLs (Years of Life Lost)	Both	Prostate cancer	Age-standardized	-2.708054938	-2.866345947	-2.549505975	-2.71 (-2.87 to -2.55)
537	Greece	Deaths	Both	Prostate cancer	All ages	-1.189696871	-1.456805793	-0.92186393	-1.19 (-1.46 to -0.92)
538	Greece	Deaths	Both	Prostate cancer	Age-standardized	-3.531399248	-3.870168907	-3.191435736	-3.53 (-3.87 to -3.19)
539	Greece	DALYs (Disability-Adjusted Life Years)	Both	Prostate cancer	All ages	-1.556108616	-1.809265601	-1.302298938	-1.56 (-1.81 to -1.3)
540	Greece	DALYs (Disability-Adjusted Life Years)	Both	Prostate cancer	Age-standardized	-3.250180266	-3.538161244	-2.961339539	-3.25 (-3.54 to -2.96)

541	Greece	YLDs (Years Lived with Disability)	Both	Prostate cancer	All ages	-0.760250369	-1.069754923	-0.449777526	-0.76 (-1.07 to -0.45)
542	Greece	YLDs (Years Lived with Disability)	Both	Prostate cancer	Age-standardized	-1.935706363	-2.244571152	-1.625865695	-1.94 (-2.24 to -1.63)
543	Greece	YLLs (Years of Life Lost)	Both	Prostate cancer	All ages	-1.660528422	-1.918854264	-1.401522201	-1.66 (-1.92 to -1.4)
544	Greece	YLLs (Years of Life Lost)	Both	Prostate cancer	Age-standardized	-3.433173709	-3.726362198	-3.139092353	-3.43 (-3.73 to -3.14)
545	Ireland	Deaths	Both	Prostate cancer	All ages	-3.634365999	-4.018623538	-3.248570101	-3.63 (-4.02 to -3.25)
546	Ireland	Deaths	Both	Prostate cancer	Age-standardized	-4.526121348	-4.954857178	-4.095451548	-4.53 (-4.95 to -4.1)
547	Ireland	DALYs (Disability-Adjusted Life Years)	Both	Prostate cancer	All ages	-3.550427381	-3.898304893	-3.201290591	-3.55 (-3.9 to -3.2)
548	Ireland	DALYs (Disability-Adjusted Life Years)	Both	Prostate cancer	Age-standardized	-4.370603147	-4.796917687	-3.942379592	-4.37 (-4.8 to -3.9

									4)
549	Ireland	YLDs (Years Lived with Disability)	Both	Prostate cancer	All ages	-0.9958449	-1.358948345	-0.63140485	-1 (-1.36 to -0.63)
550	Ireland	YLDs (Years Lived with Disability)	Both	Prostate cancer	Age-standardized	-1.767670294	-2.290454941	-1.242088543	-1.77 (-2.29 to -1.24)
551	Ireland	YLLs (Years of Life Lost)	Both	Prostate cancer	All ages	-3.912673659	-4.275621402	-3.548349765	-3.91 (-4.28 to -3.55)
552	Ireland	YLLs (Years of Life Lost)	Both	Prostate cancer	Age-standardized	-4.744478242	-5.175047053	-4.31195436	-4.74 (-5.18 to -4.31)
553	Italy	Deaths	Both	Prostate cancer	All ages	-1.568812129	-1.805454612	-1.331599352	-1.57 (-1.81 to -1.33)
554	Italy	Deaths	Both	Prostate cancer	Age-standardized	-3.206628338	-3.410935286	-3.001889237	-3.21 (-3.41 to -3)
555	Italy	DALYs (Disability-Adjusted Life Years)	Both	Prostate cancer	All ages	-1.867639395	-2.110710087	-1.623965131	-1.87 (-2.11 to -1.62)

556	Italy	DALYs (Disability-Adjusted Life Years)	Both	Prostate cancer	Age-standardized	-3.054906875	-3.251004648	-2.858411636	-3.05 (-3.25 to -2.86)
557	Italy	YLDs (Years Lived with Disability)	Both	Prostate cancer	All ages	-0.337714591	-0.806662219	0.133450041	-0.34 (-0.81 to 0.13)
558	Italy	YLDs (Years Lived with Disability)	Both	Prostate cancer	Age-standardized	-1.159564099	-1.58638074	-0.730896368	-1.16 (-1.59 to -0.73)
559	Italy	YLLs (Years of Life Lost)	Both	Prostate cancer	All ages	-2.141823271	-2.367189293	-1.915937037	-2.14 (-2.37 to -1.92)
560	Italy	YLLs (Years of Life Lost)	Both	Prostate cancer	Age-standardized	-3.42431576	-3.606235483	-3.242052708	-3.42 (-3.61 to -3.24)
561	Israel	Deaths	Both	Prostate cancer	All ages	-2.856654525	-3.239857176	-2.471934263	-2.86 (-3.24 to -2.47)
562	Israel	Deaths	Both	Prostate cancer	Age-standardized	-3.864573192	-4.291588029	-3.435653175	-3.86 (-4.29 to -3.44)

563	Israel	DALYs (Disability-Adjusted Life Years)	Both	Prostate cancer	All ages	-2.699451257	-3.103163972	-2.294056505	-2.7 (-3.1 to -2.29)
564	Israel	DALYs (Disability-Adjusted Life Years)	Both	Prostate cancer	Age-standardized	-3.526706086	-4.002027648	-3.049031032	-3.53 (-4 to -3.05)
565	Israel	YLDs (Years Lived with Disability)	Both	Prostate cancer	All ages	-0.328636899	-0.883979221	0.229816979	-0.33 (-0.88 to 0.23)
566	Israel	YLDs (Years Lived with Disability)	Both	Prostate cancer	Age-standardized	-1.050938319	-1.702088177	-0.395475082	-1.05 (-1.7 to -0.4)
567	Israel	YLLs (Years of Life Lost)	Both	Prostate cancer	All ages	-2.974360631	-3.371635479	-2.575452439	-2.97 (-3.37 to -2.58)
568	Israel	YLLs (Years of Life Lost)	Both	Prostate cancer	Age-standardized	-3.818476339	-4.284624726	-3.35005774	-3.82 (-4.28 to -3.35)
569	Luxembourg	Deaths	Both	Prostate cancer	All ages	-1.931139938	-2.048735255	-1.813403442	-1.93 (-2.05 to -1.81)
570	Luxembourg	Deaths	Both	Prostate cancer	Age-standardized	-2.446200286	-2.554589445	-2.337690565	-2.45 (-2.55 to

									-2.34)
571	Luxembourg	DALYs (Disability-Adjusted Life Years)	Both	Prostate cancer	All ages	-1.969143966	-2.07598578	-1.862185579	-1.97 (-2.08 to -1.86)
572	Luxembourg	DALYs (Disability-Adjusted Life Years)	Both	Prostate cancer	Age-standardized	-2.317767008	-2.420419155	-2.215006873	-2.32 (-2.42 to -2.22)
573	Luxembourg	YLDs (Years Lived with Disability)	Both	Prostate cancer	All ages	0.368610806	0.106501336	0.63140656	0.37 (0.11 to 0.63)
574	Luxembourg	YLDs (Years Lived with Disability)	Both	Prostate cancer	Age-standardized	0.139213366	-0.162066807	0.441402709	0.14 (-0.16 to 0.44)
575	Luxembourg	YLLs (Years of Life Lost)	Both	Prostate cancer	All ages	-2.292772285	-2.409686696	-2.175717809	-2.29 (-2.41 to -2.18)
576	Luxembourg	YLLs (Years of Life Lost)	Both	Prostate cancer	Age-standardized	-2.667107531	-2.775701318	-2.55839245	-2.67 (-2.78 to -2.56)
577	Malta	Deaths	Both	Prostate cancer	All ages	-0.975444603	-1.199336142	-0.751045706	-0.98 (-1.2 to -0.75)

578	Malta	Deaths	Both	Prostate cancer	Age-standardized	-3.391406974	-3.632203892	-3.15000837	-3.39 (-3.63 to -3.15)
579	Malta	DALYs (Disability-Adjusted Life Years)	Both	Prostate cancer	All ages	-0.86240696	-1.041863013	-0.682625471	-0.86 (-1.04 to -0.68)
580	Malta	DALYs (Disability-Adjusted Life Years)	Both	Prostate cancer	Age-standardized	-3.058436243	-3.273182741	-2.843212979	-3.06 (-3.27 to -2.84)
581	Malta	YLDs (Years Lived with Disability)	Both	Prostate cancer	All ages	1.318706747	1.093882166	1.54403132	1.32 (1.09 to 1.54)
582	Malta	YLDs (Years Lived with Disability)	Both	Prostate cancer	Age-standardized	-0.819123035	-1.090884136	-0.546615247	-0.82 (-1.09 to -0.55)
583	Malta	YLLs (Years of Life Lost)	Both	Prostate cancer	All ages	-1.20653522	-1.395105917	-1.017603902	-1.21 (-1.4 to -1.02)
584	Malta	YLLs (Years of Life Lost)	Both	Prostate cancer	Age-standardized	-3.415237133	-3.634319837	-3.195656355	-3.42 (-3.63 to -3.2)
585	Netherlands	Deaths	Both	Prostate cancer	All ages	-0.647064351	-0.762920481	-0.531072963	-0.65 (-0.76 to

									-0.53)
586	Netherlands	Deaths	Both	Prostate cancer	Age-standardized	-2.142334578	-2.272136337	-2.012360417	-2.14 (-2.27 to -2.01)
587	Netherlands	DALYs (Disability-Adjusted Life Years)	Both	Prostate cancer	All ages	-0.389604396	-0.483686025	-0.295433823	-0.39 (-0.48 to -0.3)
588	Netherlands	DALYs (Disability-Adjusted Life Years)	Both	Prostate cancer	Age-standardized	-1.827902036	-2.006927317	-1.648549691	-1.83 (-2.01 to -1.65)
589	Netherlands	YLDs (Years Lived with Disability)	Both	Prostate cancer	All ages	1.693160942	1.378654477	2.008643097	1.69 (1.38 to 2.01)
590	Netherlands	YLDs (Years Lived with Disability)	Both	Prostate cancer	Age-standardized	0.31924536	-0.107721794	0.748037488	0.32 (-0.11 to 0.75)
591	Netherlands	YLLs (Years of Life Lost)	Both	Prostate cancer	All ages	-0.660450816	-0.759432668	-0.561370241	-0.66 (-0.76 to -0.56)
592	Netherlands	YLLs (Years of Life Lost)	Both	Prostate cancer	Age-standardized	-2.115161457	-2.278440611	-1.951609487	-2.12 (-2.28 to -1.95)

593	Norway	Deaths	Both	Prostate cancer	All ages	-3.316063619	-3.592545871	-3.038788458	-3.32 (-3.59 to -3.04)
594	Norway	Deaths	Both	Prostate cancer	Age-standardized	-3.704848724	-4.026521999	-3.382097301	-3.7 (-4.03 to -3.38)
595	Norway	DALYs (Disability-Adjusted Life Years)	Both	Prostate cancer	All ages	-3.319386555	-3.594782616	-3.043203784	-3.32 (-3.59 to -3.04)
596	Norway	DALYs (Disability-Adjusted Life Years)	Both	Prostate cancer	Age-standardized	-3.687590414	-4.062907857	-3.310804685	-3.69 (-4.06 to -3.31)
597	Norway	YLDs (Years Lived with Disability)	Both	Prostate cancer	All ages	-0.743369812	-1.271874174	-0.212036298	-0.74 (-1.27 to -0.21)
598	Norway	YLDs (Years Lived with Disability)	Both	Prostate cancer	Age-standardized	-0.993767361	-1.683964957	-0.298724445	-0.99 (-1.68 to -0.3)
599	Norway	YLLs (Years of Life Lost)	Both	Prostate cancer	All ages	-3.678302439	-3.940385248	-3.41550458	-3.68 (-3.94 to -3.42)
600	Norway	YLLs (Years of Life Lost)	Both	Prostate cancer	Age-standardized	-4.083373509	-4.432732218	-3.732737672	-4.08 (-4.43 to

									-3.73)
601	Portugal	Deaths	Both	Prostate cancer	All ages	-2.00199596	-2.331353883	-1.671527378	-2 (-2.33 to -1.67)
602	Portugal	Deaths	Both	Prostate cancer	Age-standardized	-3.917861613	-4.258536486	-3.575974525	-3.92 (-4.26 to -3.58)
603	Portugal	DALYs (Disability-Adjusted Life Years)	Both	Prostate cancer	All ages	-2.022617428	-2.331193669	-1.713066267	-2.02 (-2.33 to -1.71)
604	Portugal	DALYs (Disability-Adjusted Life Years)	Both	Prostate cancer	Age-standardized	-3.404507432	-3.708679549	-3.099374474	-3.4 (-3.71 to -3.1)
605	Portugal	YLDs (Years Lived with Disability)	Both	Prostate cancer	All ages	0.622874966	0.207333537	1.04013957	0.62 (0.21 to 1.04)
606	Portugal	YLDs (Years Lived with Disability)	Both	Prostate cancer	Age-standardized	-0.403745597	-0.819675683	0.013928765	-0.4 (-0.82 to 0.01)
607	Portugal	YLLs (Years of Life Lost)	Both	Prostate cancer	All ages	-2.392675842	-2.703387311	-2.080972131	-2.39 (-2.7 to -2.08)

608	Portugal	YLLs (Years of Life Lost)	Both	Prostate cancer	Age-standardized	-3.851439893	-4.160873742	-3.541006982	-3.85 (-4.16 to -3.54)
609	Spain	Deaths	Both	Prostate cancer	All ages	-2.786934187	-3.067508671	-2.505547569	-2.79 (-3.07 to -2.51)
610	Spain	Deaths	Both	Prostate cancer	Age-standardized	-4.263842123	-4.471106786	-4.056127767	-4.26 (-4.47 to -4.06)
611	Spain	DALYs (Disability-Adjusted Life Years)	Both	Prostate cancer	All ages	-2.928746324	-3.192870131	-2.663901894	-2.93 (-3.19 to -2.66)
612	Spain	DALYs (Disability-Adjusted Life Years)	Both	Prostate cancer	Age-standardized	-3.94871515	-4.128822165	-3.768269779	-3.95 (-4.13 to -3.77)
613	Spain	YLDs (Years Lived with Disability)	Both	Prostate cancer	All ages	-0.286579495	-0.757482493	0.186557924	-0.29 (-0.76 to 0.19)
614	Spain	YLDs (Years Lived with Disability)	Both	Prostate cancer	Age-standardized	-0.964353763	-1.418466637	-0.508149031	-0.96 (-1.42 to -0.51)

615	Spain	YLLs (Years of Life Lost)	Both	Prostate cancer	All ages	-3.254299416	-3.531468326	-2.976334157	-3.25 (-3.53 to -2.98)
616	Spain	YLLs (Years of Life Lost)	Both	Prostate cancer	Age-standardized	-4.340310994	-4.534715192	-4.145510914	-4.34 (-4.53 to -4.15)
617	Sweden	Deaths	Both	Prostate cancer	All ages	-1.126887843	-1.380514525	-0.872608891	-1.13 (-1.38 to -0.87)
618	Sweden	Deaths	Both	Prostate cancer	Age-standardized	-1.781156565	-2.065859508	-1.495625966	-1.78 (-2.07 to -1.5)
619	Sweden	DALYs (Disability-Adjusted Life Years)	Both	Prostate cancer	All ages	-1.227731893	-1.489681762	-0.96508547	-1.23 (-1.49 to -0.97)
620	Sweden	DALYs (Disability-Adjusted Life Years)	Both	Prostate cancer	Age-standardized	-1.842299177	-2.181275451	-1.502148232	-1.84 (-2.18 to -1.5)
621	Sweden	YLDs (Years Lived with Disability)	Both	Prostate cancer	All ages	0.198221465	-0.263614646	0.662196138	0.2 (-0.26 to 0.66)
622	Sweden	YLDs (Years Lived with Disability)	Both	Prostate cancer	Age-standardized	-0.289736769	-0.838156673	0.2617162	-0.29 (-0.84 to 0.26

)

623	Sweden	YLLs (Years of Life Lost)	Both	Prostate cancer	All ages	-1.43782436	-1.679324623	-1.195730912	-1.44 (-1.68 to -1.2)
624	Sweden	YLLs (Years of Life Lost)	Both	Prostate cancer	Age-standardized	-2.08571558	-2.399236531	-1.771187513	-2.09 (-2.4 to -1.77)
625	Switzerland	Deaths	Both	Prostate cancer	All ages	-2.691079673	-3.001883343	-2.379280118	-2.69 (-3 to -2.38)
626	Switzerland	Deaths	Both	Prostate cancer	Age-standardized	-3.796074751	-4.044315327	-3.547191967	-3.8 (-4.04 to -3.55)
627	Switzerland	DALYs (Disability-Adjusted Life Years)	Both	Prostate cancer	All ages	-2.772740176	-2.973381297	-2.571684151	-2.77 (-2.97 to -2.57)
628	Switzerland	DALYs (Disability-Adjusted Life Years)	Both	Prostate cancer	Age-standardized	-3.704136438	-3.855888546	-3.552144807	-3.7 (-3.86 to -3.55)
629	Switzerland	YLDs (Years Lived with Disability)	Both	Prostate cancer	All ages	-1.129926939	-1.322566705	-0.936911099	-1.13 (-1.32 to -0.94)

630	Switzerland	YLDs (Years Lived with Disability)	Both	Prostate cancer	Age-standardized	-1.907576462	-2.166164501	-1.648304939	-1.91 (-2.17 to -1.65)
631	Switzerland	YLLs (Years of Life Lost)	Both	Prostate cancer	All ages	-3.047163335	-3.289225997	-2.804494 8	-3.05 (-3.29 to -2.8)
632	Switzerland	YLLs (Years of Life Lost)	Both	Prostate cancer	Age-standardized	-4.022549725	-4.209226799	-3.835508855	-4.02 (-4.21 to -3.84)
633	Chile	Deaths	Both	Prostate cancer	All ages	0.63031825	0.37999987	0.88126123 5	0.63 (0.38 to 0.88)
634	Chile	Deaths	Both	Prostate cancer	Age-standardized	-1.588460186	-1.888416961	-1.287586352	-1.59 (-1.89 to -1.29)
635	Chile	DALYs (Disability-Adjusted Life Years)	Both	Prostate cancer	All ages	0.422075834	0.217164945	0.627405697	0.42 (0.22 to 0.63)
636	Chile	DALYs (Disability-Adjusted Life Years)	Both	Prostate cancer	Age-standardized	-1.641130488	-1.893391598	-1.38822074	-1.64 (-1.89 to -1.39)
637	Chile	YLDs (Years Lived with Disability)	Both	Prostate cancer	All ages	3.321718221	3.001897899	3.642531583	3.32 (3 to 3.64)

638	Chile	YLDs (Years Lived with Disability)	Both	Prostate cancer	Age-standardized	1.2380959 6	0.8767393 82	1.6007469 75	1.24 (0.88 to 1.6)
639	Chile	YLLs (Years of Life Lost)	Both	Prostate cancer	All ages	0.2104443 95	0.0050506 38	0.4162599 96	0.21 (0.01 to 0.42)
640	Chile	YLLs (Years of Life Lost)	Both	Prostate cancer	Age-standardized	-1.850066 759	-2.103296 292	-1.596182 196	-1.85 (-2.1 to -1.6)
641	Argentina	Deaths	Both	Prostate cancer	All ages	-0.521891 199	-0.911173 469	-0.131079 588	-0.52 (-0.91 to -0.13)
642	Argentina	Deaths	Both	Prostate cancer	Age-standardized	-1.299358 628	-1.688510 363	-0.908666 492	-1.3 (-1.69 to -0.91)
643	Argentina	DALYs (Disability-Adjusted Life Years)	Both	Prostate cancer	All ages	-0.709468 952	-1.074777 425	-0.342811 478	-0.71 (-1.07 to -0.34)
644	Argentina	DALYs (Disability-Adjusted Life Years)	Both	Prostate cancer	Age-standardized	-1.352897 483	-1.751967 38	-0.952206 619	-1.35 (-1.75 to -0.95)
645	Argentina	YLDs (Years Lived with Disability)	Both	Prostate cancer	All ages	1.0481745 48	0.5619462 15	1.5367538 5	1.05 (0.56 to 1.54)

646	Argentina	YLDs (Years Lived with Disability)	Both	Prostate cancer	Age-standardized	0.4626940 3	-0.071277 609	0.9995189 59	0.46 (-0.07 to 1)
647	Argentina	YLLs (Years of Life Lost)	Both	Prostate cancer	All ages	-0.811987 399	-1.173319 113	-0.449334 579	-0.81 (-1.17 to -0.45)
648	Argentina	YLLs (Years of Life Lost)	Both	Prostate cancer	Age-standardized	-1.459290 037	-1.853736 628	-1.063258 178	-1.46 (-1.85 to -1.06)
649	United States of America	Deaths	Both	Prostate cancer	All ages	-2.460583 842	-2.813847 704	-2.106035 895	-2.46 (-2.81 to -2.11)
650	United States of America	Deaths	Both	Prostate cancer	Age-standardized	-3.475365 548	-3.712903 573	-3.237241 521	-3.48 (-3.71 to -3.24)
651	United States of America	DALYs (Disability-Adjusted Life Years)	Both	Prostate cancer	All ages	-2.178042 613	-2.527468 342	-1.827364 24	-2.18 (-2.53 to -1.83)
652	United States of America	DALYs (Disability-Adjusted Life Years)	Both	Prostate cancer	Age-standardized	-3.271925 272	-3.490712 109	-3.052642 444	-3.27 (-3.49 to -3.05)
653	United States of America	YLDs (Years Lived with Disability)	Both	Prostate cancer	All ages	-1.180572 647	-1.416595 391	-0.943984 832	-1.18 (-1.42 to

									-0.94)
654	United States of America	YLDs (Years Lived with Disability)	Both	Prostate cancer	Age-standardized	-2.338938755	-2.488897627	-2.188749266	-2.34 (-2.49 to -2.19)
655	United States of America	YLLs (Years of Life Lost)	Both	Prostate cancer	All ages	-2.467759153	-2.849415693	-2.084603273	-2.47 (-2.85 to -2.08)
656	United States of America	YLLs (Years of Life Lost)	Both	Prostate cancer	Age-standardized	-3.549854869	-3.800505545	-3.298551115	-3.55 (-3.8 to -3.3)
657	United Kingdom	Deaths	Both	Prostate cancer	All ages	-2.145437527	-2.352925782	-1.937508385	-2.15 (-2.35 to -1.94)
658	United Kingdom	Deaths	Both	Prostate cancer	Age-standardized	-2.89167059	-3.066358068	-2.716668302	-2.89 (-3.07 to -2.72)
659	United Kingdom	DALYs (Disability-Adjusted Life Years)	Both	Prostate cancer	All ages	-2.3480499	-2.521904087	-2.173885641	-2.35 (-2.52 to -2.17)
660	United Kingdom	DALYs (Disability-Adjusted Life Years)	Both	Prostate cancer	Age-standardized	-2.950912282	-3.093783157	-2.807830769	-2.95 (-3.09 to -2.8

									1)
661	United Kingdom	YLDs (Years Lived with Disability)	Both	Prostate cancer	All ages	-0.345759645	-0.513597628	-0.177638511	-0.35 (-0.51 to -0.18)
662	United Kingdom	YLDs (Years Lived with Disability)	Both	Prostate cancer	Age-standardized	-0.784342092	-1.022661528	-0.545448826	-0.78 (-1.02 to -0.55)
663	United Kingdom	YLLs (Years of Life Lost)	Both	Prostate cancer	All ages	-2.608690702	-2.806981091	-2.409995767	-2.61 (-2.81 to -2.41)
664	United Kingdom	YLLs (Years of Life Lost)	Both	Prostate cancer	Age-standardized	-3.246018215	-3.4092005	-3.082560247	-3.25 (-3.41 to -3.08)
665	Uruguay	Deaths	Both	Prostate cancer	All ages	0.112328364	-0.193881182	0.419477374	0.11 (-0.19 to 0.42)
666	Uruguay	Deaths	Both	Prostate cancer	Age-standardized	-0.819505709	-1.177396339	-0.460318961	-0.82 (-1.18 to -0.46)
667	Uruguay	DALYs (Disability-Adjusted Life Years)	Both	Prostate cancer	All ages	-0.240518114	-0.561383443	0.081382573	-0.24 (-0.56 to 0.08

									)
668	Uruguay	DALYs (Disability-Adjusted Life Years)	Both	Prostate cancer	Age-standardized	-0.955487222	-1.347203399	-0.562215676	-0.96 (-1.35 to -0.56)
669	Uruguay	YLDs (Years Lived with Disability)	Both	Prostate cancer	All ages	1.377297501	0.912688872	1.844045219	1.38 (0.91 to 1.84)
670	Uruguay	YLDs (Years Lived with Disability)	Both	Prostate cancer	Age-standardized	0.811220078	0.263632775	1.361798016	0.81 (0.26 to 1.36)
671	Uruguay	YLLs (Years of Life Lost)	Both	Prostate cancer	All ages	-0.34935794	-0.663440691	-0.034282121	-0.35 (-0.66 to -0.03)
672	Uruguay	YLLs (Years of Life Lost)	Both	Prostate cancer	Age-standardized	-1.077332535	-1.461727034	-0.691438526	-1.08 (-1.46 to -0.69)
673	Bahamas	Deaths	Both	Prostate cancer	All ages	2.138658226	2.030290603	2.247140948	2.14 (2.03 to 2.25)
674	Bahamas	Deaths	Both	Prostate cancer	Age-standardized	0.454316193	0.308922029	0.5999211	0.45 (0.31 to 0.6)
675	Bahamas	DALYs (Disability-Adjusted Life Years)	Both	Prostate cancer	All ages	2.010958256	1.914448317	2.107559587	2.01 (1.91 to 2.11

									)
676	Bahamas	DALYs (Disability-Adjusted Life Years)	Both	Prostate cancer	Age-standardized	0.212819746	0.080403606	0.345411085	0.21 (0.08 to 0.35)
677	Bahamas	YLDs (Years Lived with Disability)	Both	Prostate cancer	All ages	2.92779212	2.726418965	3.129560023	2.93 (2.73 to 3.13)
678	Bahamas	YLDs (Years Lived with Disability)	Both	Prostate cancer	Age-standardized	1.048377921	0.798123205	1.299253953	1.05 (0.8 to 1.3)
679	Bahamas	YLLs (Years of Life Lost)	Both	Prostate cancer	All ages	1.949520121	1.855349062	2.043778247	1.95 (1.86 to 2.04)
680	Bahamas	YLLs (Years of Life Lost)	Both	Prostate cancer	Age-standardized	0.157887418	0.030464557	0.285472596	0.16 (0.03 to 0.29)
681	Barbados	Deaths	Both	Prostate cancer	All ages	-0.108671345	-0.356986162	0.140262283	-0.11 (-0.36 to 0.14)
682	Barbados	Deaths	Both	Prostate cancer	Age-standardized	-0.884350475	-1.136569954	-0.631487536	-0.88 (-1.14 to -0.63)
683	Barbados	DALYs (Disability-Adjusted Life Years)	Both	Prostate cancer	All ages	0.055796193	-0.159896341	0.271954705	0.06 (-0.16 to 0.27)

684	Barbados	DALYs (Disability-Adjusted Life Years)	Both	Prostate cancer	Age-standardized	-0.974438 59	-1.208289 568	-0.740034 061	-0.97 (-1.21 to -0.74)
685	Barbados	YLDs (Years Lived with Disability)	Both	Prostate cancer	All ages	0.9875291 91	0.7501505 24	1.2254671 49	0.99 (0.75 to 1.23)
686	Barbados	YLDs (Years Lived with Disability)	Both	Prostate cancer	Age-standardized	-0.166224 5	-0.450124 214	0.1184848 49	-0.17 (-0.45 to 0.12)
687	Barbados	YLLs (Years of Life Lost)	Both	Prostate cancer	All ages	-0.018549 375	-0.233627 796	0.1969927 17	-0.02 (-0.23 to 0.2)
688	Barbados	YLLs (Years of Life Lost)	Both	Prostate cancer	Age-standardized	-1.039359 913	-1.270902 329	-0.807274 477	-1.04 (-1.27 to -0.81)
689	Canada	Deaths	Both	Prostate cancer	All ages	-2.712332 567	-3.010955 331	-2.412790 364	-2.71 (-3.01 to -2.41)
690	Canada	Deaths	Both	Prostate cancer	Age-standardized	-4.469037 532	-4.756481 324	-4.180726 239	-4.47 (-4.76 to -4.18)
691	Canada	DALYs (Disability-Adjusted Life Years)	Both	Prostate cancer	All ages	-2.889113 671	-3.199842 772	-2.577387 129	-2.89 (-3.2 to

									-2.58)
692	Canada	DALYs (Disability-Adjusted Life Years)	Both	Prostate cancer	Age-standardized	-4.488318505	-4.799069733	-4.176552935	-4.49 (-4.8 to -4.18)
693	Canada	YLDs (Years Lived with Disability)	Both	Prostate cancer	All ages	-1.721802266	-2.029096498	-1.41354418	-1.72 (-2.03 to -1.41)
694	Canada	YLDs (Years Lived with Disability)	Both	Prostate cancer	Age-standardized	-3.264883794	-3.627911984	-2.900488097	-3.26 (-3.63 to -2.9)
695	Canada	YLLs (Years of Life Lost)	Both	Prostate cancer	All ages	-3.070985272	-3.392487824	-2.748412784	-3.07 (-3.39 to -2.75)
696	Canada	YLLs (Years of Life Lost)	Both	Prostate cancer	Age-standardized	-4.685960784	-5.001402989	-4.369471154	-4.69 (-5 to -4.37)
697	Antigua and Barbuda	Deaths	Both	Prostate cancer	All ages	-0.142790204	-0.548042856	0.264113796	-0.14 (-0.55 to 0.26)
698	Antigua and Barbuda	Deaths	Both	Prostate cancer	Age-standardized	-0.526520781	-0.861970618	-0.189935894	-0.53 (-0.86 to -0.19)

699	Antigua and Barbuda	DALYs (Disability-Adjusted Life Years)	Both	Prostate cancer	All ages	0.101490051	-0.307932901	0.512594452	0.1 (-0.31 to 0.51)
700	Antigua and Barbuda	DALYs (Disability-Adjusted Life Years)	Both	Prostate cancer	Age-standardized	-0.74048244	-1.059005732	-0.420933718	-0.74 (-1.06 to -0.42)
701	Antigua and Barbuda	YLDs (Years Lived with Disability)	Both	Prostate cancer	All ages	1.145884653	0.694494163	1.599298623	1.15 (0.69 to 1.6)
702	Antigua and Barbuda	YLDs (Years Lived with Disability)	Both	Prostate cancer	Age-standardized	0.106131346	-0.253919646	0.467482005	0.11 (-0.25 to 0.47)
703	Antigua and Barbuda	YLLs (Years of Life Lost)	Both	Prostate cancer	All ages	0.017521697	-0.388137329	0.424832726	0.02 (-0.39 to 0.42)
704	Antigua and Barbuda	YLLs (Years of Life Lost)	Both	Prostate cancer	Age-standardized	-0.808072725	-1.124303965	-0.490830091	-0.81 (-1.12 to -0.49)
705	Cuba	Deaths	Both	Prostate cancer	All ages	1.654831816	1.591748268	1.717954537	1.65 (1.59 to 1.72)
706	Cuba	Deaths	Both	Prostate cancer	Age-standardized	-0.564626466	-0.661366864	-0.467791857	-0.56 (-0.66 to -0.47)

707	Cuba	DALYs (Disability-Adjusted Life Years)	Both	Prostate cancer	All ages	1.766094326	1.686545719	1.845705163	1.77 (1.69 to 1.85)
708	Cuba	DALYs (Disability-Adjusted Life Years)	Both	Prostate cancer	Age-standardized	-0.334080841	-0.457745566	-0.210262484	-0.33 (-0.46 to -0.21)
709	Cuba	YLDs (Years Lived with Disability)	Both	Prostate cancer	All ages	2.971894461	2.84767479	3.096264165	2.97 (2.85 to 3.1)
710	Cuba	YLDs (Years Lived with Disability)	Both	Prostate cancer	Age-standardized	0.909975415	0.736780313	1.083468288	0.91 (0.74 to 1.08)
711	Cuba	YLLs (Years of Life Lost)	Both	Prostate cancer	All ages	1.634606053	1.555861846	1.713411316	1.63 (1.56 to 1.71)
712	Cuba	YLLs (Years of Life Lost)	Both	Prostate cancer	Age-standardized	-0.470393982	-0.592449465	-0.348188636	-0.47 (-0.59 to -0.35)
713	Dominican Republic	Deaths	Both	Prostate cancer	All ages	1.651212051	1.081276461	2.22436116	1.65 (1.08 to 2.22)
714	Dominican Republic	Deaths	Both	Prostate cancer	Age-standardized	-0.576231066	-1.038593063	-0.111708848	-0.58 (-1.04 to -0.11)

715	Dominican Republic	DALYs (Disability-Adjusted Life Years)	Both	Prostate cancer	All ages	1.689438424	1.172697095	2.20881902	1.69 (1.17 to 2.21)
716	Dominican Republic	DALYs (Disability-Adjusted Life Years)	Both	Prostate cancer	Age-standardized	-0.351909551	-0.806272548	0.104534684	-0.35 (-0.81 to 0.1)
717	Dominican Republic	YLDs (Years Lived with Disability)	Both	Prostate cancer	All ages	2.891507712	2.235311475	3.551915738	2.89 (2.24 to 3.55)
718	Dominican Republic	YLDs (Years Lived with Disability)	Both	Prostate cancer	Age-standardized	0.901260663	0.295708404	1.510469045	0.9 (0.3 to 1.51)
719	Dominican Republic	YLLs (Years of Life Lost)	Both	Prostate cancer	All ages	1.631214537	1.119814975	2.145200433	1.63 (1.12 to 2.15)
720	Dominican Republic	YLLs (Years of Life Lost)	Both	Prostate cancer	Age-standardized	-0.411615795	-0.860379654	0.039179431	-0.41 (-0.86 to 0.04)
721	Belize	Deaths	Both	Prostate cancer	All ages	1.090231725	0.513884341	1.66988389	1.09 (0.51 to 1.67)
722	Belize	Deaths	Both	Prostate cancer	Age-standardized	0.498855212	-0.185968328	1.188377322	0.5 (-0.19 to 1.19)
723	Belize	DALYs (Disability-Adjusted	Both	Prostate canc	All ages	1.265809645	0.718539869	1.816053097	1.27 (0.72 to

		Life Years)		er					1.82)
724	Belize	DALYs (Disability-Adjusted Life Years)	Both	Prostate cancer	Age-standardized	0.433776042	-0.224717715	1.096615706	0.43 (-0.22 to 1.1)
725	Belize	YLDs (Years Lived with Disability)	Both	Prostate cancer	All ages	2.187619604	1.747075829	2.630070843	2.19 (1.75 to 2.63)
726	Belize	YLDs (Years Lived with Disability)	Both	Prostate cancer	Age-standardized	1.274517357	0.721384574	1.830687785	1.27 (0.72 to 1.83)
727	Belize	YLLs (Years of Life Lost)	Both	Prostate cancer	All ages	1.212165834	0.658526178	1.768850605	1.21 (0.66 to 1.77)
728	Belize	YLLs (Years of Life Lost)	Both	Prostate cancer	Age-standardized	0.385383817	-0.279376338	1.054575412	0.39 (-0.28 to 1.05)
729	Jamaica	Deaths	Both	Prostate cancer	All ages	1.735134266	1.074208241	2.400382098	1.74 (1.07 to 2.4)
730	Jamaica	Deaths	Both	Prostate cancer	Age-standardized	0.690687974	-0.006477666	1.392714328	0.69 (-0.01 to 1.39)
731	Jamaica	DALYs (Disability-Adjusted Life Years)	Both	Prostate cancer	All ages	1.699141435	1.021867671	2.380955798	1.7 (1.02 to 2.38)
732	Jamaica	DALYs (Disability-Adjusted	Both	Prostate canc	Age-standardized	0.601374685	-0.132668993	1.340813721	0.6 (-0.13 to

		Life Years)		er					1.34)
733	Jamaica	YLDs (Years Lived with Disability)	Both	Prostate cancer	All ages	2.414017362	1.673004248	3.160431127	2.41 (1.67 to 3.16)
734	Jamaica	YLDs (Years Lived with Disability)	Both	Prostate cancer	Age-standardized	1.316503288	0.504128057	2.135444952	1.32 (0.5 to 2.14)
735	Jamaica	YLLs (Years of Life Lost)	Both	Prostate cancer	All ages	1.645544943	0.972418383	2.323158861	1.65 (0.97 to 2.32)
736	Jamaica	YLLs (Years of Life Lost)	Both	Prostate cancer	Age-standardized	0.547072523	-0.181701504	1.281167334	0.55 (-0.18 to 1.28)
737	Dominica	Deaths	Both	Prostate cancer	All ages	0.843512378	0.602240138	1.085363256	0.84 (0.6 to 1.09)
738	Dominica	Deaths	Both	Prostate cancer	Age-standardized	-0.236109624	-0.420023762	-0.051855816	-0.24 (-0.42 to -0.05)
739	Dominica	DALYs (Disability-Adjusted Life Years)	Both	Prostate cancer	All ages	1.261283908	1.028136303	1.494969559	1.26 (1.03 to 1.49)
740	Dominica	DALYs (Disability-Adjusted Life Years)	Both	Prostate cancer	Age-standardized	0.000225354	-0.166788066	0.167518176	0 (-0.17 to 0.17)

741	Dominica	YLDs (Years Lived with Disability)	Both	Prostate cancer	All ages	1.752640086	1.521394974	1.984411927	1.75 (1.52 to 1.98)
742	Dominica	YLDs (Years Lived with Disability)	Both	Prostate cancer	Age-standardized	0.454529779	0.279928126	0.629435438	0.45 (0.28 to 0.63)
743	Dominica	YLLs (Years of Life Lost)	Both	Prostate cancer	All ages	1.232810653	0.99917791	1.466983839	1.23 (1 to 1.47)
744	Dominica	YLLs (Years of Life Lost)	Both	Prostate cancer	Age-standardized	-0.025879313	-0.19329422	0.141816415	-0.03 (-0.19 to 0.14)
745	Guyana	Deaths	Both	Prostate cancer	All ages	1.886279152	1.68343907	2.089523864	1.89 (1.68 to 2.09)
746	Guyana	Deaths	Both	Prostate cancer	Age-standardized	-0.18529286	-0.376735645	0.006517814	-0.19 (-0.38 to 0.01)
747	Guyana	DALYs (Disability-Adjusted Life Years)	Both	Prostate cancer	All ages	2.042524685	1.845173461	2.240258328	2.04 (1.85 to 2.24)
748	Guyana	DALYs (Disability-Adjusted Life Years)	Both	Prostate cancer	Age-standardized	-0.094315842	-0.285132884	0.096866353	-0.09 (-0.29 to 0.1)
749	Guyana	YLDs (Years Lived with	Both	Prostate canc	All ages	2.675942127	2.442910322	2.909504022	2.68 (2.44 to

		Disability)		er					2.91)
750	Guyana	YLDs (Years Lived with Disability)	Both	Prostate cancer	Age-standardized	0.504678275	0.279722614	0.730138576	0.5 (0.28 to 0.73)
751	Guyana	YLLs (Years of Life Lost)	Both	Prostate cancer	All ages	2.016662221	1.819806237	2.213898802	2.02 (1.82 to 2.21)
752	Guyana	YLLs (Years of Life Lost)	Both	Prostate cancer	Age-standardized	-0.118274764	-0.308615406	0.072429295	-0.12 (-0.31 to 0.07)
753	Grenada	Deaths	Both	Prostate cancer	All ages	-0.04795075	-0.55700005	0.463704377	-0.05 (-0.56 to 0.46)
754	Grenada	Deaths	Both	Prostate cancer	Age-standardized	-0.933687794	-1.933845474	0.0766703	-0.93 (-1.93 to 0.08)
755	Grenada	DALYs (Disability-Adjusted Life Years)	Both	Prostate cancer	All ages	0.552534185	0.178894938	0.927567002	0.55 (0.18 to 0.93)
756	Grenada	DALYs (Disability-Adjusted Life Years)	Both	Prostate cancer	Age-standardized	-0.78517427	-1.598441582	0.034814518	-0.79 (-1.6 to 0.03)

757	Grenada	YLDs (Years Lived with Disability)	Both	Prostate cancer	All ages	1.619738771	1.183671916	2.057684925	1.62 (1.18 to 2.06)
758	Grenada	YLDs (Years Lived with Disability)	Both	Prostate cancer	Age-standardized	0.132078577	-0.733928715	1.005641004	0.13 (-0.73 to 1.01)
759	Grenada	YLLs (Years of Life Lost)	Both	Prostate cancer	All ages	0.486059678	0.114629893	0.858867485	0.49 (0.11 to 0.86)
760	Grenada	YLLs (Years of Life Lost)	Both	Prostate cancer	Age-standardized	-0.842353069	-1.653836627	-0.024173718	-0.84 (-1.65 to -0.02)
761	Haiti	Deaths	Both	Prostate cancer	All ages	0.01417169	-0.096245561	0.124710978	0.01 (-0.1 to 0.12)
762	Haiti	Deaths	Both	Prostate cancer	Age-standardized	0.052083763	-0.001865794	0.106062426	0.05 (0 to 0.11)
763	Haiti	DALYs (Disability-Adjusted Life Years)	Both	Prostate cancer	All ages	-0.286751906	-0.448687081	-0.12455332	-0.29 (-0.45 to -0.12)
764	Haiti	DALYs (Disability-Adjusted Life Years)	Both	Prostate cancer	Age-standardized	-0.305226215	-0.393125652	-0.217249209	-0.31 (-0.39 to -0.22)

765	Haiti	YLDs (Years Lived with Disability)	Both	Prostate cancer	All ages	0.295854422	0.121947206	0.470063707	0.3 (0.12 to 0.47)
766	Haiti	YLDs (Years Lived with Disability)	Both	Prostate cancer	Age-standardized	0.22542844	0.120056037	0.330911743	0.23 (0.12 to 0.33)
767	Haiti	YLLs (Years of Life Lost)	Both	Prostate cancer	All ages	-0.302951117	-0.464903243	-0.140735548	-0.3 (-0.46 to -0.14)
768	Haiti	YLLs (Years of Life Lost)	Both	Prostate cancer	Age-standardized	-0.319594164	-0.407521116	-0.231589584	-0.32 (-0.41 to -0.23)
769	Saint Lucia	Deaths	Both	Prostate cancer	All ages	0.853067529	0.491851958	1.215581481	0.85 (0.49 to 1.22)
770	Saint Lucia	Deaths	Both	Prostate cancer	Age-standardized	-1.76338262	-2.138307144	-1.387021696	-1.76 (-2.14 to -1.39)
771	Saint Lucia	DALYs (Disability-Adjusted Life Years)	Both	Prostate cancer	All ages	0.723679266	0.393704165	1.054738932	0.72 (0.39 to 1.05)
772	Saint Lucia	DALYs (Disability-Adjusted Life Years)	Both	Prostate cancer	Age-standardized	-1.781277082	-2.154549518	-1.406580641	-1.78 (-2.15 to -1.41)

773	Saint Lucia	YLDs (Years Lived with Disability)	Both	Prostate cancer	All ages	1.824727301	1.454244262	2.196563241	1.82 (1.45 to 2.2)
774	Saint Lucia	YLDs (Years Lived with Disability)	Both	Prostate cancer	Age-standardized	-0.631577609	-1.091003536	-0.170017679	-0.63 (-1.09 to -0.17)
775	Saint Lucia	YLLs (Years of Life Lost)	Both	Prostate cancer	All ages	0.65413088	0.323888912	0.985459924	0.65 (0.32 to 0.99)
776	Saint Lucia	YLLs (Years of Life Lost)	Both	Prostate cancer	Age-standardized	-1.8528598	-2.223632495	-1.480681117	-1.85 (-2.22 to -1.48)
777	Saint Vincent and the Grenadines	Deaths	Both	Prostate cancer	All ages	3.132787266	2.864000202	3.40227668	3.13 (2.86 to 3.4)
778	Saint Vincent and the Grenadines	Deaths	Both	Prostate cancer	Age-standardized	1.031853415	0.724623476	1.340020465	1.03 (0.72 to 1.34)
779	Saint Vincent and the Grenadines	DALYs (Disability-Adjusted Life Years)	Both	Prostate cancer	All ages	3.07190563	2.837342362	3.307003918	3.07 (2.84 to 3.31)
780	Saint Vincent and the Grenadines	DALYs (Disability-Adjusted Life Years)	Both	Prostate cancer	Age-standardized	0.882056884	0.606984954	1.157880895	0.88 (0.61 to 1.16)
781	Saint Vincent and the Grenadines	YLDs (Years Lived with Disability)	Both	Prostate cancer	All ages	3.734044373	3.510440368	3.958131409	3.73 (3.51 to 3.96

)

782	Saint Vincent and the Grenadines	YLDs (Years Lived with Disability)	Both	Prostate cancer	Age-standardized	1.511488858	1.259423875	1.764181305	1.51 (1.26 to 1.76)
783	Saint Vincent and the Grenadines	YLLs (Years of Life Lost)	Both	Prostate cancer	All ages	3.034019404	2.798435124	3.270143574	3.03 (2.8 to 3.27)
784	Saint Vincent and the Grenadines	YLLs (Years of Life Lost)	Both	Prostate cancer	Age-standardized	0.846489093	0.569824161	1.123915124	0.85 (0.57 to 1.12)
785	Trinidad and Tobago	Deaths	Both	Prostate cancer	All ages	0.583122842	0.295519182	0.871551223	0.58 (0.3 to 0.87)
786	Trinidad and Tobago	Deaths	Both	Prostate cancer	Age-standardized	-1.757642619	-2.032766799	-1.481745801	-1.76 (-2.03 to -1.48)
787	Trinidad and Tobago	DALYs (Disability-Adjusted Life Years)	Both	Prostate cancer	All ages	0.740917135	0.480198078	1.002312687	0.74 (0.48 to 1)
788	Trinidad and Tobago	DALYs (Disability-Adjusted Life Years)	Both	Prostate cancer	Age-standardized	-1.683869 99	-1.953140719	-1.413859 75	-1.68 (-1.95 to -1.41)
789	Trinidad and Tobago	YLDs (Years Lived with Disability)	Both	Prostate cancer	All ages	2.066175114	1.799809632	2.333237558	2.07 (1.8 to 2.33)

790	Trinidad and Tobago	YLDs (Years Lived with Disability)	Both	Prostate cancer	Age-standardized	-0.428725633	-0.659167732	-0.197748975	-0.43 (-0.66 to -0.2)
791	Trinidad and Tobago	YLLs (Years of Life Lost)	Both	Prostate cancer	All ages	0.655246398	0.39335086	0.917825141	0.66 (0.39 to 0.92)
792	Trinidad and Tobago	YLLs (Years of Life Lost)	Both	Prostate cancer	Age-standardized	-1.76398333	-2.037041998	-1.490163547	-1.76 (-2.04 to -1.49)
793	Suriname	Deaths	Both	Prostate cancer	All ages	0.573125651	0.325952186	0.820908078	0.57 (0.33 to 0.82)
794	Suriname	Deaths	Both	Prostate cancer	Age-standardized	-1.1480364	-1.49077763	-0.804102678	-1.15 (-1.49 to -0.8)
795	Suriname	DALYs (Disability-Adjusted Life Years)	Both	Prostate cancer	All ages	0.525481148	0.260969928	0.790690208	0.53 (0.26 to 0.79)
796	Suriname	DALYs (Disability-Adjusted Life Years)	Both	Prostate cancer	Age-standardized	-1.12499749	-1.482478585	-0.766219238	-1.12 (-1.48 to -0.77)
797	Suriname	YLDs (Years Lived with Disability)	Both	Prostate cancer	All ages	1.336067303	1.051858552	1.621075392	1.34 (1.05 to 1.62)

798	Suriname	YLDs (Years Lived with Disability)	Both	Prostate cancer	Age-standardized	-0.313320434	-0.684899326	0.059648688	-0.31 (-0.68 to 0.06)
799	Suriname	YLLs (Years of Life Lost)	Both	Prostate cancer	All ages	0.486183397	0.222128806	0.750933691	0.49 (0.22 to 0.75)
800	Suriname	YLLs (Years of Life Lost)	Both	Prostate cancer	Age-standardized	-1.163758849	-1.521139441	-0.80508132	-1.16 (-1.52 to -0.81)
801	Bolivia (Plurinational State of)	Deaths	Both	Prostate cancer	All ages	1.088750455	0.966715037	1.210933373	1.09 (0.97 to 1.21)
802	Bolivia (Plurinational State of)	Deaths	Both	Prostate cancer	Age-standardized	-0.643843777	-0.73637361	-0.551227691	-0.64 (-0.74 to -0.55)
803	Bolivia (Plurinational State of)	DALYs (Disability-Adjusted Life Years)	Both	Prostate cancer	All ages	0.944922882	0.811857479	1.078163924	0.94 (0.81 to 1.08)
804	Bolivia (Plurinational State of)	DALYs (Disability-Adjusted Life Years)	Both	Prostate cancer	Age-standardized	-0.721405464	-0.822115149	-0.620593514	-0.72 (-0.82 to -0.62)
805	Bolivia (Plurinational State of)	YLDs (Years Lived with Disability)	Both	Prostate cancer	All ages	2.602801183	2.472989049	2.732777762	2.6 (2.47 to 2.73)

806	Bolivia (Plurinational State of)	YLDs (Years Lived with Disability)	Both	Prostate cancer	Age-standardized	0.863487505	0.764006587	0.963066636	0.86 (0.76 to 0.96)
807	Bolivia (Plurinational State of)	YLLs (Years of Life Lost)	Both	Prostate cancer	All ages	0.887925453	0.755777628	1.020246598	0.89 (0.76 to 1.02)
808	Bolivia (Plurinational State of)	YLLs (Years of Life Lost)	Both	Prostate cancer	Age-standardized	-0.774512498	-0.874349751	-0.674574692	-0.77 (-0.87 to -0.67)
809	Peru	Deaths	Both	Prostate cancer	All ages	2.031903297	1.870407731	2.193654883	2.03 (1.87 to 2.19)
810	Peru	Deaths	Both	Prostate cancer	Age-standardized	-0.313781496	-0.540889278	-0.08615513	-0.31 (-0.54 to -0.09)
811	Peru	DALYs (Disability-Adjusted Life Years)	Both	Prostate cancer	All ages	1.849025265	1.667206316	2.031169376	1.85 (1.67 to 2.03)
812	Peru	DALYs (Disability-Adjusted Life Years)	Both	Prostate cancer	Age-standardized	-0.273000311	-0.507425746	-0.03802252	-0.27 (-0.51 to -0.04)
813	Peru	YLDs (Years Lived with Disability)	Both	Prostate cancer	All ages	4.604239073	4.361964235	4.847076347	4.6 (4.36 to 4.85)

814	Peru	YLDs (Years Lived with Disability)	Both	Prostate cancer	Age-standardized	2.522125434	2.241730463	2.803289379	2.52 (2.24 to 2.8)
815	Peru	YLLs (Years of Life Lost)	Both	Prostate cancer	All ages	1.701389037	1.521640769	1.881455555	1.7 (1.52 to 1.88)
816	Peru	YLLs (Years of Life Lost)	Both	Prostate cancer	Age-standardized	-0.422410722	-0.653933265	-0.190348625	-0.42 (-0.65 to -0.19)
817	Ecuador	Deaths	Both	Prostate cancer	All ages	-0.142378445	-0.441077806	0.157217081	-0.14 (-0.44 to 0.16)
818	Ecuador	Deaths	Both	Prostate cancer	Age-standardized	-2.28081679	-2.570933891	-1.989835799	-2.28 (-2.57 to -1.99)
819	Ecuador	DALYs (Disability-Adjusted Life Years)	Both	Prostate cancer	All ages	-0.210501158	-0.514288271	0.094213591	-0.21 (-0.51 to 0.09)
820	Ecuador	DALYs (Disability-Adjusted Life Years)	Both	Prostate cancer	Age-standardized	-2.260114806	-2.559701739	-1.959606773	-2.26 (-2.56 to -1.96)
821	Ecuador	YLDs (Years Lived with Disability)	Both	Prostate cancer	All ages	1.596899522	1.234809021	1.960285126	1.6 (1.23 to 1.96)

822	Ecuador	YLDs (Years Lived with Disability)	Both	Prostate cancer	Age-standardized	-0.484232613	-0.847787064	-0.119345141	-0.48 (-0.85 to -0.12)
823	Ecuador	YLLs (Years of Life Lost)	Both	Prostate cancer	All ages	-0.297565278	-0.6029331	0.008740694	-0.3 (-0.6 to 0.01)
824	Ecuador	YLLs (Years of Life Lost)	Both	Prostate cancer	Age-standardized	-2.344253369	-2.644975324	-2.042602507	-2.34 (-2.64 to -2.04)
825	Costa Rica	Deaths	Both	Prostate cancer	All ages	2.374722109	2.073173579	2.677161486	2.37 (2.07 to 2.68)
826	Costa Rica	Deaths	Both	Prostate cancer	Age-standardized	-0.219275723	-0.636116826	0.199314068	-0.22 (-0.64 to 0.2)
827	Costa Rica	DALYs (Disability-Adjusted Life Years)	Both	Prostate cancer	All ages	2.590126138	2.341802591	2.839052219	2.59 (2.34 to 2.84)
828	Costa Rica	DALYs (Disability-Adjusted Life Years)	Both	Prostate cancer	Age-standardized	0.052786764	-0.306065466	0.412930696	0.05 (-0.31 to 0.41)
829	Costa Rica	YLDs (Years Lived with Disability)	Both	Prostate cancer	All ages	4.129643542	3.879621954	4.380266893	4.13 (3.88 to 4.38)

830	Costa Rica	YLDs (Years Lived with Disability)	Both	Prostate cancer	Age-standardized	1.57535708	1.219984677	1.931977157	1.58 (1.22 to 1.93)
831	Costa Rica	YLLs (Years of Life Lost)	Both	Prostate cancer	All ages	2.348970249	2.095501256	2.603068521	2.35 (2.1 to 2.6)
832	Costa Rica	YLLs (Years of Life Lost)	Both	Prostate cancer	Age-standardized	-0.183762299	-0.548727165	0.18254191	-0.18 (-0.55 to 0.18)
833	Colombia	Deaths	Both	Prostate cancer	All ages	-0.07829419	-0.364531465	0.2087654	-0.08 (-0.36 to 0.21)
834	Colombia	Deaths	Both	Prostate cancer	Age-standardized	-2.985333517	-3.310047866	-2.659528677	-2.99 (-3.31 to -2.66)
835	Colombia	DALYs (Disability-Adjusted Life Years)	Both	Prostate cancer	All ages	-0.208642345	-0.512786706	0.096431822	-0.21 (-0.51 to 0.1)
836	Colombia	DALYs (Disability-Adjusted Life Years)	Both	Prostate cancer	Age-standardized	-2.863847819	-3.199226811	-2.527306861	-2.86 (-3.2 to -2.53)
837	Colombia	YLDs (Years Lived with Disability)	Both	Prostate cancer	All ages	2.204584319	1.797066973	2.613733051	2.2 (1.8 to 2.61)

838	Colombia	YLDs (Years Lived with Disability)	Both	Prostate cancer	Age-standardized	-0.429466924	-0.869052455	0.012067901	-0.43 (-0.87 to 0.01)
839	Colombia	YLLs (Years of Life Lost)	Both	Prostate cancer	All ages	-0.485260519	-0.782899245	-0.186728915	-0.49 (-0.78 to -0.19)
840	Colombia	YLLs (Years of Life Lost)	Both	Prostate cancer	Age-standardized	-3.138863702	-3.468560944	-2.80804 04	-3.14 (-3.47 to -2.81)
841	Guatemala	Deaths	Both	Prostate cancer	All ages	2.652366169	1.998744872	3.310175956	2.65 (2 to 3.31)
842	Guatemala	Deaths	Both	Prostate cancer	Age-standardized	-0.059224509	-0.837171117	0.724825202	-0.06 (-0.84 to 0.72)
843	Guatemala	DALYs (Disability-Adjusted Life Years)	Both	Prostate cancer	All ages	2.28758669	1.647483578	2.931720712	2.29 (1.65 to 2.93)
844	Guatemala	DALYs (Disability-Adjusted Life Years)	Both	Prostate cancer	Age-standardized	0.057025321	-0.691397526	0.811088533	0.06 (-0.69 to 0.81)
845	Guatemala	YLDs (Years Lived with Disability)	Both	Prostate cancer	All ages	4.083476274	3.296263892	4.876687937	4.08 (3.3 to 4.88)

846	Guatemala	YLDs (Years Lived with Disability)	Both	Prostate cancer	Age-standardized	1.990205295	1.121707042	2.866162769	1.99 (1.12 to 2.87)
847	Guatemala	YLLs (Years of Life Lost)	Both	Prostate cancer	All ages	2.178923848	1.544675583	2.817133629	2.18 (1.54 to 2.82)
848	Guatemala	YLLs (Years of Life Lost)	Both	Prostate cancer	Age-standardized	-0.054106457	-0.798913295	0.696292429	-0.05 (-0.8 to 0.7)
849	El Salvador	Deaths	Both	Prostate cancer	All ages	2.614724532	2.347654831	2.882491135	2.61 (2.35 to 2.88)
850	El Salvador	Deaths	Both	Prostate cancer	Age-standardized	0.455672017	0.202425529	0.709558548	0.46 (0.2 to 0.71)
851	El Salvador	DALYs (Disability-Adjusted Life Years)	Both	Prostate cancer	All ages	2.675905121	2.424630212	2.927796475	2.68 (2.42 to 2.93)
852	El Salvador	DALYs (Disability-Adjusted Life Years)	Both	Prostate cancer	Age-standardized	0.736762666	0.484690002	0.989467672	0.74 (0.48 to 0.99)
853	El Salvador	YLDs (Years Lived with Disability)	Both	Prostate cancer	All ages	5.341579803	4.810332044	5.875520275	5.34 (4.81 to 5.88)
854	El Salvador	YLDs (Years Lived with Disability)	Both	Prostate cancer	Age-standardized	3.454318253	2.922875937	3.988504672	3.45 (2.92 to 3.99

									)
855	El Salvador	YLLs (Years of Life Lost)	Both	Prostate cancer	All ages	2.435913898	2.199020965	2.673355939	2.44 (2.2 to 2.67)
856	El Salvador	YLLs (Years of Life Lost)	Both	Prostate cancer	Age-standardized	0.490126879	0.251863875	0.728956149	0.49 (0.25 to 0.73)
857	Panama	Deaths	Both	Prostate cancer	All ages	0.625498428	0.202558991	1.050223026	0.63 (0.2 to 1.05)
858	Panama	Deaths	Both	Prostate cancer	Age-standardized	-1.337370376	-1.76624316	-0.906625202	-1.34 (-1.77 to -0.91)
859	Panama	DALYs (Disability-Adjusted Life Years)	Both	Prostate cancer	All ages	0.638803478	0.265718957	1.013276231	0.64 (0.27 to 1.01)
860	Panama	DALYs (Disability-Adjusted Life Years)	Both	Prostate cancer	Age-standardized	-1.179403651	-1.574156774	-0.783067304	-1.18 (-1.57 to -0.78)
861	Panama	YLDs (Years Lived with Disability)	Both	Prostate cancer	All ages	2.265930196	1.829962972	2.703763937	2.27 (1.83 to 2.7)
862	Panama	YLDs (Years Lived with Disability)	Both	Prostate cancer	Age-standardized	0.493660027	0.028518238	0.960964768	0.49 (0.03 to 0.96)

863	Panama	YLLs (Years of Life Lost)	Both	Prostate cancer	All ages	0.430946758	0.05800919 9	0.805190925	0.43 (0.06 to 0.81)
864	Panama	YLLs (Years of Life Lost)	Both	Prostate cancer	Age-standardized	-1.392146176	-1.786030919	-0.996681767	-1.39 (-1.79 to -1)
865	Honduras	Deaths	Both	Prostate cancer	All ages	1.86163768	1.718734926	2.004741195	1.86 (1.72 to 2)
866	Honduras	Deaths	Both	Prostate cancer	Age-standardized	0.624511995	0.529518529	0.719595223	0.62 (0.53 to 0.72)
867	Honduras	DALYs (Disability-Adjusted Life Years)	Both	Prostate cancer	All ages	1.925588571	1.781150936	2.070231177	1.93 (1.78 to 2.07)
868	Honduras	DALYs (Disability-Adjusted Life Years)	Both	Prostate cancer	Age-standardized	0.612395627	0.516044656	0.708838956	0.61 (0.52 to 0.71)
869	Honduras	YLDs (Years Lived with Disability)	Both	Prostate cancer	All ages	3.388770479	3.256361426	3.521349324	3.39 (3.26 to 3.52)
870	Honduras	YLDs (Years Lived with Disability)	Both	Prostate cancer	Age-standardized	1.995885774	1.887988964	2.103896844	2 (1.89 to 2.1)
871	Honduras	YLLs (Years of Life Lost)	Both	Prostate cancer	All ages	1.838944977	1.694573329	1.983521583	1.84 (1.69 to 1.98)

872	Honduras	YLLs (Years of Life Lost)	Both	Prostate cancer	Age-standardized	0.533184194	0.437420451	0.629039244	0.53 (0.44 to 0.63)
873	Nicaragua	Deaths	Both	Prostate cancer	All ages	2.074544549	1.851456662	2.298121071	2.07 (1.85 to 2.3)
874	Nicaragua	Deaths	Both	Prostate cancer	Age-standardized	-0.142590385	-0.3620696	0.077372292	-0.14 (-0.36 to 0.08)
875	Nicaragua	DALYs (Disability-Adjusted Life Years)	Both	Prostate cancer	All ages	2.21740634	2.050751178	2.384333659	2.22 (2.05 to 2.38)
876	Nicaragua	DALYs (Disability-Adjusted Life Years)	Both	Prostate cancer	Age-standardized	0.049662954	-0.139120115	0.238802911	0.05 (-0.14 to 0.24)
877	Nicaragua	YLDs (Years Lived with Disability)	Both	Prostate cancer	All ages	4.351174707	4.153794188	4.548929278	4.35 (4.15 to 4.55)
878	Nicaragua	YLDs (Years Lived with Disability)	Both	Prostate cancer	Age-standardized	2.150178214	1.930729742	2.37009914	2.15 (1.93 to 2.37)
879	Nicaragua	YLLs (Years of Life Lost)	Both	Prostate cancer	All ages	2.032121356	1.862713925	2.201810528	2.03 (1.86 to 2.2)
880	Nicaragua	YLLs (Years of Life Lost)	Both	Prostate cancer	Age-standardized	-0.128519304	-0.319852055	0.063180704	-0.13 (-0.32 to 0.06

)

881	Paraguay	Deaths	Both	Prostate cancer	All ages	1.684133683	1.469601437	1.899119504	1.68 (1.47 to 1.9)
882	Paraguay	Deaths	Both	Prostate cancer	Age-standardized	0.388834351	0.152779606	0.625445463	0.39 (0.15 to 0.63)
883	Paraguay	DALYs (Disability-Adjusted Life Years)	Both	Prostate cancer	All ages	1.684957245	1.505725194	1.864505771	1.68 (1.51 to 1.86)
884	Paraguay	DALYs (Disability-Adjusted Life Years)	Both	Prostate cancer	Age-standardized	0.382217462	0.15531403	0.609634948	0.38 (0.16 to 0.61)
885	Paraguay	YLDs (Years Lived with Disability)	Both	Prostate cancer	All ages	2.925265828	2.715960215	3.134997945	2.93 (2.72 to 3.13)
886	Paraguay	YLDs (Years Lived with Disability)	Both	Prostate cancer	Age-standardized	1.585952258	1.33951197	1.832991847	1.59 (1.34 to 1.83)
887	Paraguay	YLLs (Years of Life Lost)	Both	Prostate cancer	All ages	1.620002631	1.44014129	1.800182879	1.62 (1.44 to 1.8)
888	Paraguay	YLLs (Years of Life Lost)	Both	Prostate cancer	Age-standardized	0.320108008	0.092357698	0.548376541	0.32 (0.09 to 0.55)
889	Venezuela (Bolivarian Republic of)	Deaths	Both	Prostate canc	All ages	0.995770041	0.651999989	1.340714216	1 (0.65 to

				er					1.34)
890	Venezuela (Bolivarian Republic of)	Deaths	Both	Prostate cancer	Age-standardized	-1.457363691	-1.834060828	-1.079221035	-1.46 (-1.83 to -1.08)
891	Venezuela (Bolivarian Republic of)	DALYs (Disability-Adjusted Life Years)	Both	Prostate cancer	All ages	1.061010037	0.746119804	1.376884485	1.06 (0.75 to 1.38)
892	Venezuela (Bolivarian Republic of)	DALYs (Disability-Adjusted Life Years)	Both	Prostate cancer	Age-standardized	-1.416326975	-1.784269122	-1.04700642	-1.42 (-1.78 to -1.05)
893	Venezuela (Bolivarian Republic of)	YLDs (Years Lived with Disability)	Both	Prostate cancer	All ages	2.821384619	2.447579877	3.196553277	2.82 (2.45 to 3.2)
894	Venezuela (Bolivarian Republic of)	YLDs (Years Lived with Disability)	Both	Prostate cancer	Age-standardized	0.26732774	-0.222421183	0.759480551	0.27 (-0.22 to 0.76)
895	Venezuela (Bolivarian Republic of)	YLLs (Years of Life Lost)	Both	Prostate cancer	All ages	0.876922566	0.557667749	1.197190968	0.88 (0.56 to 1.2)
896	Venezuela (Bolivarian Republic of)	YLLs (Years of Life Lost)	Both	Prostate cancer	Age-standardized	-1.588760048	-1.953260442	-1.222904579	-1.59 (-1.95 to -1.22)
897	Brazil	Deaths	Both	Prostate cancer	All ages	0.742024977	0.629689388	0.854485969	0.74 (0.63 to 0.85)

898	Brazil	Deaths	Both	Prostate cancer	Age-standardized	-1.623647706	-1.756992314	-1.49012211	-1.62 (-1.76 to -1.49)
899	Brazil	DALYs (Disability-Adjusted Life Years)	Both	Prostate cancer	All ages	0.375019418	0.241177489	0.509040052	0.38 (0.24 to 0.51)
900	Brazil	DALYs (Disability-Adjusted Life Years)	Both	Prostate cancer	Age-standardized	-1.814631761	-1.96982966	-1.659188159	-1.81 (-1.97 to -1.66)
901	Brazil	YLDs (Years Lived with Disability)	Both	Prostate cancer	All ages	1.72570249	1.497390984	1.954527567	1.73 (1.5 to 1.95)
902	Brazil	YLDs (Years Lived with Disability)	Both	Prostate cancer	Age-standardized	-0.462597149	-0.711364856	-0.213206154	-0.46 (-0.71 to -0.21)
903	Brazil	YLLs (Years of Life Lost)	Both	Prostate cancer	All ages	0.299754346	0.170320306	0.429355632	0.3 (0.17 to 0.43)
904	Brazil	YLLs (Years of Life Lost)	Both	Prostate cancer	Age-standardized	-1.888566376	-2.040199947	-1.736698088	-1.89 (-2.04 to -1.74)
905	Algeria	Deaths	Both	Prostate cancer	All ages	1.243949119	1.021861003	1.466525477	1.24 (1.02 to 1.47)

906	Algeria	Deaths	Both	Prostate cancer	Age-standardized	-0.534681569	-0.796578804	-0.272092925	-0.53 (-0.8 to -0.27)
907	Algeria	DALYs (Disability-Adjusted Life Years)	Both	Prostate cancer	All ages	1.104682291	0.880561788	1.32930071	1.1 (0.88 to 1.33)
908	Algeria	DALYs (Disability-Adjusted Life Years)	Both	Prostate cancer	Age-standardized	-0.59351802	-0.797722463	-0.388893229	-0.59 (-0.8 to -0.39)
909	Algeria	YLDs (Years Lived with Disability)	Both	Prostate cancer	All ages	3.244269858	2.982180788	3.507025943	3.24 (2.98 to 3.51)
910	Algeria	YLDs (Years Lived with Disability)	Both	Prostate cancer	Age-standardized	1.456460733	1.25272083	1.6606106	1.46 (1.25 to 1.66)
911	Algeria	YLLs (Years of Life Lost)	Both	Prostate cancer	All ages	0.934126642	0.718286034	1.150429799	0.93 (0.72 to 1.15)
912	Algeria	YLLs (Years of Life Lost)	Both	Prostate cancer	Age-standardized	-0.737936333	-0.937676155	-0.537793776	-0.74 (-0.94 to -0.54)
913	Bahrain	Deaths	Both	Prostate cancer	All ages	-0.56889229	-1.15197059	0.017625434	-0.57 (-1.15 to 0.02)

914	Bahrain	Deaths	Both	Prostate cancer	Age-standardized	-1.351464669	-1.656911591	-1.045069049	-1.35 (-1.66 to -1.05)
915	Bahrain	DALYs (Disability-Adjusted Life Years)	Both	Prostate cancer	All ages	0.0406963 8	-0.529415265	0.614075597	0.04 (-0.53 to 0.61)
916	Bahrain	DALYs (Disability-Adjusted Life Years)	Both	Prostate cancer	Age-standardized	-1.181648523	-1.446679143	-0.91590518	-1.18 (-1.45 to -0.92)
917	Bahrain	YLDs (Years Lived with Disability)	Both	Prostate cancer	All ages	3.249843603	2.745656634	3.756504686	3.25 (2.75 to 3.76)
918	Bahrain	YLDs (Years Lived with Disability)	Both	Prostate cancer	Age-standardized	1.733468062	1.543558882	1.923732415	1.73 (1.54 to 1.92)
919	Bahrain	YLLs (Years of Life Lost)	Both	Prostate cancer	All ages	-0.341411028	-0.910238404	0.230681715	-0.34 (-0.91 to 0.23)
920	Bahrain	YLLs (Years of Life Lost)	Both	Prostate cancer	Age-standardized	-1.476346511	-1.751308236	-1.200615269	-1.48 (-1.75 to -1.2)
921	Egypt	Deaths	Both	Prostate cancer	All ages	2.796953753	2.522054722	3.072589888	2.8 (2.52 to 3.07)

922	Egypt	Deaths	Both	Prostate cancer	Age-standardized	2.599928005	2.218216247	2.983065184	2.6 (2.22 to 2.98)
923	Egypt	DALYs (Disability-Adjusted Life Years)	Both	Prostate cancer	All ages	2.719186493	2.484683216	2.954226355	2.72 (2.48 to 2.95)
924	Egypt	DALYs (Disability-Adjusted Life Years)	Both	Prostate cancer	Age-standardized	2.407334412	2.060806832	2.75503856	2.41 (2.06 to 2.76)
925	Egypt	YLDs (Years Lived with Disability)	Both	Prostate cancer	All ages	4.843624159	4.63997454	5.04767012	4.84 (4.64 to 5.05)
926	Egypt	YLDs (Years Lived with Disability)	Both	Prostate cancer	Age-standardized	4.277451234	3.969131887	4.586684898	4.28 (3.97 to 4.59)
927	Egypt	YLLs (Years of Life Lost)	Both	Prostate cancer	All ages	2.567862695	2.326945198	2.809347407	2.57 (2.33 to 2.81)
928	Egypt	YLLs (Years of Life Lost)	Both	Prostate cancer	Age-standardized	2.288530398	1.936255546	2.642022653	2.29 (1.94 to 2.64)
929	Iran (Islamic Republic of)	Deaths	Both	Prostate cancer	All ages	3.586060098	3.401046803	3.771404433	3.59 (3.4 to 3.77)
930	Iran (Islamic Republic of)	Deaths	Both	Prostate cancer	Age-standardized	0.914929399	0.756095133	1.074014055	0.91 (0.76 to 1.07

									)
931	Iran (Islamic Republic of)	DALYs (Disability-Adjusted Life Years)	Both	Prostate cancer	All ages	3.079814048	2.898952873	3.260993114	3.08 (2.9 to 3.26)
932	Iran (Islamic Republic of)	DALYs (Disability-Adjusted Life Years)	Both	Prostate cancer	Age-standardized	0.812559119	0.660755583	0.964591585	0.81 (0.66 to 0.96)
933	Iran (Islamic Republic of)	YLDs (Years Lived with Disability)	Both	Prostate cancer	All ages	5.111381745	4.902580389	5.320598706	5.11 (4.9 to 5.32)
934	Iran (Islamic Republic of)	YLDs (Years Lived with Disability)	Both	Prostate cancer	Age-standardized	2.975985485	2.824776042	3.127417289	2.98 (2.82 to 3.13)
935	Iran (Islamic Republic of)	YLLs (Years of Life Lost)	Both	Prostate cancer	All ages	2.89842741	2.717956715	3.079215183	2.9 (2.72 to 3.08)
936	Iran (Islamic Republic of)	YLLs (Years of Life Lost)	Both	Prostate cancer	Age-standardized	0.631507621	0.47813757	0.785111777	0.63 (0.48 to 0.79)
937	Iraq	Deaths	Both	Prostate cancer	All ages	0.308608759	-0.08009847	0.698828133	0.31 (-0.08 to 0.7)
938	Iraq	Deaths	Both	Prostate cancer	Age-standardized	-0.206121229	-0.398692344	-0.013177793	-0.21 (-0.4 to -0.01)

939	Iraq	DALYs (Disability-Adjusted Life Years)	Both	Prostate cancer	All ages	0.689600931	0.323305818	1.057233441	0.69 (0.32 to 1.06)
940	Iraq	DALYs (Disability-Adjusted Life Years)	Both	Prostate cancer	Age-standardized	-0.130263095	-0.315481823	0.055299779	-0.13 (-0.32 to 0.06)
941	Iraq	YLDs (Years Lived with Disability)	Both	Prostate cancer	All ages	3.48745928	2.961390566	4.016215878	3.49 (2.96 to 4.02)
942	Iraq	YLDs (Years Lived with Disability)	Both	Prostate cancer	Age-standardized	2.41496356	2.076529034	2.754520165	2.41 (2.08 to 2.75)
943	Iraq	YLLs (Years of Life Lost)	Both	Prostate cancer	All ages	0.471331365	0.123121681	0.820752058	0.47 (0.12 to 0.82)
944	Iraq	YLLs (Years of Life Lost)	Both	Prostate cancer	Age-standardized	-0.316371704	-0.487491144	-0.144958011	-0.32 (-0.49 to -0.14)
945	Kuwait	Deaths	Both	Prostate cancer	All ages	2.74168452	1.849042193	3.64215029	2.74 (1.85 to 3.64)
946	Kuwait	Deaths	Both	Prostate cancer	Age-standardized	1.542505626	0.885332393	2.203959726	1.54 (0.89 to 2.2)
947	Kuwait	DALYs (Disability-Adjusted	Both	Prostate canc	All ages	2.392660283	1.571969723	3.219981934	2.39 (1.57 to

		Life Years)		er					3.22)
948	Kuwait	DALYs (Disability-Adjusted Life Years)	Both	Prostate cancer	Age-standardized	1.332505823	0.700095785	1.968887479	1.33 (0.7 to 1.97)
949	Kuwait	YLDs (Years Lived with Disability)	Both	Prostate cancer	All ages	4.228781777	3.471853201	4.99124752	4.23 (3.47 to 4.99)
950	Kuwait	YLDs (Years Lived with Disability)	Both	Prostate cancer	Age-standardized	3.182902099	2.573344714	3.796081869	3.18 (2.57 to 3.8)
951	Kuwait	YLLs (Years of Life Lost)	Both	Prostate cancer	All ages	2.074833374	1.238256658	2.918323094	2.07 (1.24 to 2.92)
952	Kuwait	YLLs (Years of Life Lost)	Both	Prostate cancer	Age-standardized	1.041603646	0.399856559	1.687452724	1.04 (0.4 to 1.69)
953	Jordan	Deaths	Both	Prostate cancer	All ages	0.421213516	0.32482893	0.517690702	0.42 (0.32 to 0.52)
954	Jordan	Deaths	Both	Prostate cancer	Age-standardized	-1.199441179	-1.277405102	-1.121415686	-1.2 (-1.28 to -1.12)
955	Jordan	DALYs (Disability-Adjusted Life Years)	Both	Prostate cancer	All ages	0.665810663	0.56522862	0.766493306	0.67 (0.57 to 0.77)

956	Jordan	DALYs (Disability-Adjusted Life Years)	Both	Prostate cancer	Age-standardized	-1.00627854	-1.091315158	-0.921168813	-1.01 (-1.09 to -0.92)
957	Jordan	YLDs (Years Lived with Disability)	Both	Prostate cancer	All ages	3.730188775	3.60280454	3.857729633	3.73 (3.6 to 3.86)
958	Jordan	YLDs (Years Lived with Disability)	Both	Prostate cancer	Age-standardized	1.960592879	1.730466262	2.191240071	1.96 (1.73 to 2.19)
959	Jordan	YLLs (Years of Life Lost)	Both	Prostate cancer	All ages	0.336607342	0.22137539	0.451971785	0.34 (0.22 to 0.45)
960	Jordan	YLLs (Years of Life Lost)	Both	Prostate cancer	Age-standardized	-1.291045466	-1.377260722	-1.204754841	-1.29 (-1.38 to -1.2)
961	Lebanon	Deaths	Both	Prostate cancer	All ages	3.626971292	2.981515606	4.276472492	3.63 (2.98 to 4.28)
962	Lebanon	Deaths	Both	Prostate cancer	Age-standardized	1.325638139	0.888746966	1.764421236	1.33 (0.89 to 1.76)
963	Lebanon	DALYs (Disability-Adjusted Life Years)	Both	Prostate cancer	All ages	2.869133179	2.243690203	3.498402102	2.87 (2.24 to 3.5)
964	Lebanon	DALYs (Disability-Adjusted Life Years)	Both	Prostate cancer	Age-standardized	1.200503057	0.797491885	1.605125558	1.2 (0.8 to 1.61

									)
965	Lebanon	YLDs (Years Lived with Disability)	Both	Prostate cancer	All ages	5.229707401	4.506779507	5.957636164	5.23 (4.51 to 5.96)
966	Lebanon	YLDs (Years Lived with Disability)	Both	Prostate cancer	Age-standardized	4.001011772	3.518481562	4.485791197	4 (3.52 to 4.49)
967	Lebanon	YLLs (Years of Life Lost)	Both	Prostate cancer	All ages	2.612482585	1.989744415	3.239023125	2.61 (1.99 to 3.24)
968	Lebanon	YLLs (Years of Life Lost)	Both	Prostate cancer	Age-standardized	0.899998331	0.494221495	1.307413618	0.9 (0.49 to 1.31)
969	Libya	Deaths	Both	Prostate cancer	All ages	1.155457871	0.992166572	1.319013192	1.16 (0.99 to 1.32)
970	Libya	Deaths	Both	Prostate cancer	Age-standardized	-0.118472447	-0.203073947	-0.033799228	-0.12 (-0.2 to -0.03)
971	Libya	DALYs (Disability-Adjusted Life Years)	Both	Prostate cancer	All ages	1.358171735	1.15023565	1.566535278	1.36 (1.15 to 1.57)
972	Libya	DALYs (Disability-Adjusted Life Years)	Both	Prostate cancer	Age-standardized	-0.040986164	-0.115638592	0.033722057	-0.04 (-0.12 to 0.03

									)
973	Libya	YLDs (Years Lived with Disability)	Both	Prostate cancer	All ages	3.401856859	3.238804014	3.565167226	3.4 (3.24 to 3.57)
974	Libya	YLDs (Years Lived with Disability)	Both	Prostate cancer	Age-standardized	1.91553519	1.677721297	2.153905306	1.92 (1.68 to 2.15)
975	Libya	YLLs (Years of Life Lost)	Both	Prostate cancer	All ages	1.168992513	0.952559993	1.385889044	1.17 (0.95 to 1.39)
976	Libya	YLLs (Years of Life Lost)	Both	Prostate cancer	Age-standardized	-0.212702523	-0.283992965	-0.141361113	-0.21 (-0.28 to -0.14)
977	Palestine	Deaths	Both	Prostate cancer	All ages	-0.632661024	-0.766165321	-0.498977118	-0.63 (-0.77 to -0.5)
978	Palestine	Deaths	Both	Prostate cancer	Age-standardized	-0.868970089	-1.062056913	-0.675506438	-0.87 (-1.06 to -0.68)
979	Palestine	DALYs (Disability-Adjusted Life Years)	Both	Prostate cancer	All ages	-0.170782551	-0.312489017	-0.028874648	-0.17 (-0.31 to -0.03)

980	Palestine	DALYs (Disability-Adjusted Life Years)	Both	Prostate cancer	Age-standardized	-0.539093659	-0.692331294	-0.385619569	-0.54 (-0.69 to -0.39)
981	Palestine	YLDs (Years Lived with Disability)	Both	Prostate cancer	All ages	2.152814207	1.944825364	2.36122739	2.15 (1.94 to 2.36)
982	Palestine	YLDs (Years Lived with Disability)	Both	Prostate cancer	Age-standardized	1.715371207	1.596770161	1.834110704	1.72 (1.6 to 1.83)
983	Palestine	YLLs (Years of Life Lost)	Both	Prostate cancer	All ages	-0.369074741	-0.501794452	-0.236177996	-0.37 (-0.5 to -0.24)
984	Palestine	YLLs (Years of Life Lost)	Both	Prostate cancer	Age-standardized	-0.716656407	-0.880509755	-0.552532196	-0.72 (-0.88 to -0.55)
985	Morocco	Deaths	Both	Prostate cancer	All ages	1.004249483	0.589237213	1.420974015	1 (0.59 to 1.42)
986	Morocco	Deaths	Both	Prostate cancer	Age-standardized	-0.792439405	-1.070336426	-0.513761761	-0.79 (-1.07 to -0.51)
987	Morocco	DALYs (Disability-Adjusted Life Years)	Both	Prostate cancer	All ages	1.469070448	0.996963893	1.943383847	1.47 (1 to 1.94)

988	Morocco	DALYs (Disability-Adjusted Life Years)	Both	Prostate cancer	Age-standardized	-0.344742078	-0.667741315	-0.020692542	-0.34 (-0.67 to -0.02)
989	Morocco	YLDs (Years Lived with Disability)	Both	Prostate cancer	All ages	3.706814522	3.083638434	4.333757923	3.71 (3.08 to 4.33)
990	Morocco	YLDs (Years Lived with Disability)	Both	Prostate cancer	Age-standardized	1.837808181	1.375020143	2.302708898	1.84 (1.38 to 2.3)
991	Morocco	YLLs (Years of Life Lost)	Both	Prostate cancer	All ages	1.327401227	0.868837866	1.788049278	1.33 (0.87 to 1.79)
992	Morocco	YLLs (Years of Life Lost)	Both	Prostate cancer	Age-standardized	-0.478239216	-0.789122316	-0.166381946	-0.48 (-0.79 to -0.17)
993	Oman	Deaths	Both	Prostate cancer	All ages	-0.167141238	-0.51590821	0.182848427	-0.17 (-0.52 to 0.18)
994	Oman	Deaths	Both	Prostate cancer	Age-standardized	-0.442183408	-0.748531029	-0.134890221	-0.44 (-0.75 to -0.13)
995	Oman	DALYs (Disability-Adjusted Life Years)	Both	Prostate cancer	All ages	0.171013814	-0.107227928	0.450030572	0.17 (-0.11 to 0.45)

996	Oman	DALYs (Disability-Adjusted Life Years)	Both	Prostate cancer	Age-standardized	-0.402024117	-0.673982113	-0.129321491	-0.4 (-0.67 to -0.13)
997	Oman	YLDs (Years Lived with Disability)	Both	Prostate cancer	All ages	2.906235303	2.622101603	3.191155695	2.91 (2.62 to 3.19)
998	Oman	YLDs (Years Lived with Disability)	Both	Prostate cancer	Age-standardized	2.155704189	1.871544706	2.440656304	2.16 (1.87 to 2.44)
999	Oman	YLLs (Years of Life Lost)	Both	Prostate cancer	All ages	-0.169637202	-0.466587884	0.128199411	-0.17 (-0.47 to 0.13)
1000	Oman	YLLs (Years of Life Lost)	Both	Prostate cancer	Age-standardized	-0.697536872	-0.984944992	-0.409294502	-0.7 (-0.98 to -0.41)
1001	Qatar	Deaths	Both	Prostate cancer	All ages	-0.919343782	-1.449973268	-0.385857192	-0.92 (-1.45 to -0.39)
1002	Qatar	Deaths	Both	Prostate cancer	Age-standardized	-1.192383381	-1.561980717	-0.821398348	-1.19 (-1.56 to -0.82)
1003	Qatar	DALYs (Disability-Adjusted Life Years)	Both	Prostate cancer	All ages	-0.16350869	-0.617483905	0.292540266	-0.16 (-0.62 to 0.29)

1004	Qatar	DALYs (Disability-Adjusted Life Years)	Both	Prostate cancer	Age-standardized	-0.610607747	-0.920707226	-0.299537715	-0.61 (-0.92 to -0.3)
1005	Qatar	YLDs (Years Lived with Disability)	Both	Prostate cancer	All ages	3.284778573	2.908611165	3.662321007	3.28 (2.91 to 3.66)
1006	Qatar	YLDs (Years Lived with Disability)	Both	Prostate cancer	Age-standardized	2.745641888	2.452758258	3.03936279	2.75 (2.45 to 3.04)
1007	Qatar	YLLs (Years of Life Lost)	Both	Prostate cancer	All ages	-0.716520601	-1.188008746	-0.242782719	-0.72 (-1.19 to -0.24)
1008	Qatar	YLLs (Years of Life Lost)	Both	Prostate cancer	Age-standardized	-1.070778599	-1.397799453	-0.742673159	-1.07 (-1.4 to -0.74)
1009	Syrian Arab Republic	Deaths	Both	Prostate cancer	All ages	1.362194617	0.59956294	2.130607702	1.36 (0.6 to 2.13)
1010	Syrian Arab Republic	Deaths	Both	Prostate cancer	Age-standardized	-0.989079476	-1.106324718	-0.871695232	-0.99 (-1.11 to -0.87)
1011	Syrian Arab Republic	DALYs (Disability-Adjusted Life Years)	Both	Prostate cancer	All ages	1.483408781	0.725658639	2.24685941	1.48 (0.73 to 2.25)

1012	Syrian Arab Republic	DALYs (Disability-Adjusted Life Years)	Both	Prostate cancer	Age-standardized	-1.068014165	-1.210430581	-0.925392439	-1.07 (-1.21 to -0.93)
1013	Syrian Arab Republic	YLDs (Years Lived with Disability)	Both	Prostate cancer	All ages	4.373632974	3.769427214	4.981356769	4.37 (3.77 to 4.98)
1014	Syrian Arab Republic	YLDs (Years Lived with Disability)	Both	Prostate cancer	Age-standardized	1.574000809	1.394945724	1.75337209	1.57 (1.39 to 1.75)
1015	Syrian Arab Republic	YLLs (Years of Life Lost)	Both	Prostate cancer	All ages	1.21325907	0.448671225	1.983666748	1.21 (0.45 to 1.98)
1016	Syrian Arab Republic	YLLs (Years of Life Lost)	Both	Prostate cancer	Age-standardized	-1.289824438	-1.434507055	-1.144929443	-1.29 (-1.43 to -1.14)
1017	Saudi Arabia	Deaths	Both	Prostate cancer	All ages	-0.017070396	-0.305114181	0.271805621	-0.02 (-0.31 to 0.27)
1018	Saudi Arabia	Deaths	Both	Prostate cancer	Age-standardized	-0.378328217	-0.611254064	-0.144856488	-0.38 (-0.61 to -0.14)
1019	Saudi Arabia	DALYs (Disability-Adjusted Life Years)	Both	Prostate cancer	All ages	1.078774776	0.836897943	1.321231797	1.08 (0.84 to 1.32)

1020	Saudi Arabia	DALYs (Disability-Adjusted Life Years)	Both	Prostate cancer	Age-standardized	0.234876849	0.044449956	0.425666205	0.23 (0.04 to 0.43)
1021	Saudi Arabia	YLDs (Years Lived with Disability)	Both	Prostate cancer	All ages	4.66261546	4.500056695	4.825427098	4.66 (4.5 to 4.83)
1022	Saudi Arabia	YLDs (Years Lived with Disability)	Both	Prostate cancer	Age-standardized	3.701332183	3.558097491	3.844764988	3.7 (3.56 to 3.84)
1023	Saudi Arabia	YLLs (Years of Life Lost)	Both	Prostate cancer	All ages	0.583091321	0.31631431	0.850577788	0.58 (0.32 to 0.85)
1024	Saudi Arabia	YLLs (Years of Life Lost)	Both	Prostate cancer	Age-standardized	-0.212297518	-0.430311666	0.006193987	-0.21 (-0.43 to 0.01)
1025	Turkey	Deaths	Both	Prostate cancer	All ages	-0.515869915	-0.743629866	-0.287587331	-0.52 (-0.74 to -0.29)
1026	Turkey	Deaths	Both	Prostate cancer	Age-standardized	-3.032217051	-3.335035579	-2.728449896	-3.03 (-3.34 to -2.73)
1027	Turkey	DALYs (Disability-Adjusted Life Years)	Both	Prostate cancer	All ages	-0.425552923	-0.643386802	-0.207241456	-0.43 (-0.64 to -0.21)

1028	Turkey	DALYs (Disability-Adjusted Life Years)	Both	Prostate cancer	Age-standardized	-2.735196975	-2.99149299	-2.478223828	-2.74 (-2.99 to -2.48)
1029	Turkey	YLDs (Years Lived with Disability)	Both	Prostate cancer	All ages	3.179689617	3.026529531	3.333077392	3.18 (3.03 to 3.33)
1030	Turkey	YLDs (Years Lived with Disability)	Both	Prostate cancer	Age-standardized	0.908916063	0.741395454	1.076715239	0.91 (0.74 to 1.08)
1031	Turkey	YLLs (Years of Life Lost)	Both	Prostate cancer	All ages	-0.763493538	-0.990464953	-0.53600181	-0.76 (-0.99 to -0.54)
1032	Turkey	YLLs (Years of Life Lost)	Both	Prostate cancer	Age-standardized	-3.060676729	-3.328878699	-2.791730667	-3.06 (-3.33 to -2.79)
1033	United Arab Emirates	Deaths	Both	Prostate cancer	All ages	0.491473468	-0.279431645	1.268338181	0.49 (-0.28 to 1.27)
1034	United Arab Emirates	Deaths	Both	Prostate cancer	Age-standardized	2.653270854	2.133084642	3.176106488	2.65 (2.13 to 3.18)
1035	United Arab Emirates	DALYs (Disability-Adjusted Life Years)	Both	Prostate cancer	All ages	0.938244261	0.192618813	1.689418593	0.94 (0.19 to 1.69)

1036	United Arab Emirates	DALYs (Disability-Adjusted Life Years)	Both	Prostate cancer	Age-standardized	1.982365564	1.535727845	2.430967963	1.98 (1.54 to 2.43)
1037	United Arab Emirates	YLDs (Years Lived with Disability)	Both	Prostate cancer	All ages	3.37751691	2.51755095 9	4.24469666 4	3.38 (2.52 to 4.24)
1038	United Arab Emirates	YLDs (Years Lived with Disability)	Both	Prostate cancer	Age-standardized	3.58923483	3.14558900 7	4.03478884 5	3.59 (3.15 to 4.03)
1039	United Arab Emirates	YLLs (Years of Life Lost)	Both	Prostate cancer	All ages	0.71114279 1	-0.017848289	1.44544909 9	0.71 (-0.02 to 1.45)
1040	United Arab Emirates	YLLs (Years of Life Lost)	Both	Prostate cancer	Age-standardized	1.86111886 5	1.41296512 3	2.31125304 2	1.86 (1.41 to 2.31)
1041	Yemen	Deaths	Both	Prostate cancer	All ages	1.08608498 2	1.05490448 6	1.11727509 8	1.09 (1.05 to 1.12)
1042	Yemen	Deaths	Both	Prostate cancer	Age-standardized	0.79592633 8	0.73474156 7	0.85714827 3	0.8 (0.73 to 0.86)
1043	Yemen	DALYs (Disability-Adjusted Life Years)	Both	Prostate cancer	All ages	0.80896859 2	0.76385687 4	0.85410050 6	0.81 (0.76 to 0.85)
1044	Yemen	DALYs (Disability-Adjusted Life Years)	Both	Prostate cancer	Age-standardized	0.50123306	0.44587865 7	0.55661796 8	0.5 (0.45 to 0.56

)

1045	Yemen	YLDs (Years Lived with Disability)	Both	Prostate cancer	All ages	2.27898161	2.179422766	2.378637459	2.28 (2.18 to 2.38)
1046	Yemen	YLDs (Years Lived with Disability)	Both	Prostate cancer	Age-standardized	1.887052464	1.779457646	1.994761025	1.89 (1.78 to 1.99)
1047	Yemen	YLLs (Years of Life Lost)	Both	Prostate cancer	All ages	0.734983455	0.687882764	0.782106179	0.73 (0.69 to 0.78)
1048	Yemen	YLLs (Years of Life Lost)	Both	Prostate cancer	Age-standardized	0.435438278	0.378046008	0.492863362	0.44 (0.38 to 0.49)
1049	Afghanistan	Deaths	Both	Prostate cancer	All ages	-2.495000666	-2.710920744	-2.278601382	-2.5 (-2.71 to -2.28)
1050	Afghanistan	Deaths	Both	Prostate cancer	Age-standardized	0.349983242	0.136449943	0.563971884	0.35 (0.14 to 0.56)
1051	Afghanistan	DALYs (Disability-Adjusted Life Years)	Both	Prostate cancer	All ages	-2.644217591	-2.844983928	-2.443036379	-2.64 (-2.84 to -2.44)
1052	Afghanistan	DALYs (Disability-Adjusted Life Years)	Both	Prostate cancer	Age-standardized	0.281902502	0.069218496	0.495038541	0.28 (0.07 to 0.5)

1053	Afghanistan	YLDs (Years Lived with Disability)	Both	Prostate cancer	All ages	-1.643852046	-1.878472711	-1.408670373	-1.64 (-1.88 to -1.41)
1054	Afghanistan	YLDs (Years Lived with Disability)	Both	Prostate cancer	Age-standardized	1.330237743	1.175629915	1.48508183	1.33 (1.18 to 1.49)
1055	Afghanistan	YLLs (Years of Life Lost)	Both	Prostate cancer	All ages	-2.678045085	-2.87958332	-2.476088629	-2.68 (-2.88 to -2.48)
1056	Afghanistan	YLLs (Years of Life Lost)	Both	Prostate cancer	Age-standardized	0.248138043	0.031710362	0.465033986	0.25 (0.03 to 0.47)
1057	Bangladesh	Deaths	Both	Prostate cancer	All ages	1.759713127	1.200844952	2.321667577	1.76 (1.2 to 2.32)
1058	Bangladesh	Deaths	Both	Prostate cancer	Age-standardized	-0.854738094	-1.083606172	-0.625340471	-0.85 (-1.08 to -0.63)
1059	Bangladesh	DALYs (Disability-Adjusted Life Years)	Both	Prostate cancer	All ages	1.695760729	1.193130375	2.200887668	1.7 (1.19 to 2.2)
1060	Bangladesh	DALYs (Disability-Adjusted Life Years)	Both	Prostate cancer	Age-standardized	-0.934153449	-1.172435828	-0.695296549	-0.93 (-1.17 to -0.7)

1061	Bangladesh	YLDs (Years Lived with Disability)	Both	Prostate cancer	All ages	3.830879885	3.287957124	4.376656466	3.83 (3.29 to 4.38)
1062	Bangladesh	YLDs (Years Lived with Disability)	Both	Prostate cancer	Age-standardized	1.031680562	0.750857053	1.313286813	1.03 (0.75 to 1.31)
1063	Bangladesh	YLLs (Years of Life Lost)	Both	Prostate cancer	All ages	1.631851319	1.132023715	2.134149234	1.63 (1.13 to 2.13)
1064	Bangladesh	YLLs (Years of Life Lost)	Both	Prostate cancer	Age-standardized	-0.991689682	-1.227530119	-0.755286125	-0.99 (-1.23 to -0.76)
1065	Bhutan	Deaths	Both	Prostate cancer	All ages	2.978507506	2.831736325	3.125488173	2.98 (2.83 to 3.13)
1066	Bhutan	Deaths	Both	Prostate cancer	Age-standardized	-0.087936095	-0.154753498	-0.021073978	-0.09 (-0.15 to -0.02)
1067	Bhutan	DALYs (Disability-Adjusted Life Years)	Both	Prostate cancer	All ages	2.343893515	2.205906114	2.482067212	2.34 (2.21 to 2.48)
1068	Bhutan	DALYs (Disability-Adjusted Life Years)	Both	Prostate cancer	Age-standardized	-0.283425533	-0.359047777	-0.207745896	-0.28 (-0.36 to -0.21)

1069	Bhutan	YLDs (Years Lived with Disability)	Both	Prostate cancer	All ages	4.16550939	4.07029331 7	4.26081257 9	4.17 (4.07 to 4.26)
1070	Bhutan	YLDs (Years Lived with Disability)	Both	Prostate cancer	Age-standardized	1.47651724 5	1.37766962 5	1.57546124 6	1.48 (1.38 to 1.58)
1071	Bhutan	YLLs (Years of Life Lost)	Both	Prostate cancer	All ages	2.29270121 3	2.15289587 9	2.43269788 3	2.29 (2.15 to 2.43)
1072	Bhutan	YLLs (Years of Life Lost)	Both	Prostate cancer	Age-standardized	-0.332053902	-0.407033268	-0.257018086	-0.33 (-0.41 to -0.26)
1073	Nepal	Deaths	Both	Prostate cancer	All ages	0.85620112 1	0.59609044	1.11698437	0.86 (0.6 to 1.12)
1074	Nepal	Deaths	Both	Prostate cancer	Age-standardized	-1.125137692	-1.308705244	-0.94122887	-1.13 (-1.31 to -0.94)
1075	Nepal	DALYs (Disability-Adjusted Life Years)	Both	Prostate cancer	All ages	0.57112874 2	0.29871954	0.84427780 2	0.57 (0.3 to 0.84)
1076	Nepal	DALYs (Disability-Adjusted Life Years)	Both	Prostate cancer	Age-standardized	-1.248484812	-1.434547358	-1.062071035	-1.25 (-1.43 to -1.06)

1077	Nepal	YLDs (Years Lived with Disability)	Both	Prostate cancer	All ages	2.2526579 6	1.9830463 55	2.5229823 35	2.25 (1.98 to 2.52)
1078	Nepal	YLDs (Years Lived with Disability)	Both	Prostate cancer	Age-standardized	0.3037669 5	0.1196265 71	0.4882460 01	0.3 (0.12 to 0.49)
1079	Nepal	YLLs (Years of Life Lost)	Both	Prostate cancer	All ages	0.5245469 94	0.2530917 73	0.7967372 34	0.52 (0.25 to 0.8)
1080	Nepal	YLLs (Years of Life Lost)	Both	Prostate cancer	Age-standardized	-1.290286 227	-1.475612 365	-1.104611 487	-1.29 (-1.48 to -1.1)
1081	India	Deaths	Both	Prostate cancer	All ages	0.8705395 23	0.6562880 87	1.0852470 03	0.87 (0.66 to 1.09)
1082	India	Deaths	Both	Prostate cancer	Age-standardized	-0.973376 903	-1.187234 339	-0.759056 623	-0.97 (-1.19 to -0.76)
1083	India	DALYs (Disability-Adjusted Life Years)	Both	Prostate cancer	All ages	0.4819230 57	0.2464718 48	0.7179272 75	0.48 (0.25 to 0.72)
1084	India	DALYs (Disability-Adjusted Life Years)	Both	Prostate cancer	Age-standardized	-1.129993 902	-1.337545 202	-0.922005 985	-1.13 (-1.34 to -0.92)
108	India	YLDs (Years Lived with	Both	Prostate canc	All ages	2.1420040 47	1.8645672 86	2.4201964 3	2.14 (1.86 to

5		Disability)		er					2.42)
1086	India	YLDs (Years Lived with Disability)	Both	Prostate cancer	Age-standardized	0.417847392	0.184973929	0.651262154	0.42 (0.18 to 0.65)
1087	India	YLLs (Years of Life Lost)	Both	Prostate cancer	All ages	0.430954108	0.19754467	0.664907271	0.43 (0.2 to 0.66)
1088	India	YLLs (Years of Life Lost)	Both	Prostate cancer	Age-standardized	-1.175896598	-1.382436709	-0.968923919	-1.18 (-1.38 to -0.97)
1089	Pakistan	Deaths	Both	Prostate cancer	All ages	-1.496008367	-1.706442129	-1.285124095	-1.5 (-1.71 to -1.29)
1090	Pakistan	Deaths	Both	Prostate cancer	Age-standardized	-1.171824542	-1.448472637	-0.894399855	-1.17 (-1.45 to -0.89)
1091	Pakistan	DALYs (Disability-Adjusted Life Years)	Both	Prostate cancer	All ages	-1.305755569	-1.499818576	-1.111310224	-1.31 (-1.5 to -1.11)
1092	Pakistan	DALYs (Disability-Adjusted Life Years)	Both	Prostate cancer	Age-standardized	-1.135998932	-1.409297254	-0.861943014	-1.14 (-1.41 to -0.86)

1093	Pakistan	YLDs (Years Lived with Disability)	Both	Prostate cancer	All ages	-0.317959224	-0.438721323	-0.197050646	-0.32 (-0.44 to -0.2)
1094	Pakistan	YLDs (Years Lived with Disability)	Both	Prostate cancer	Age-standardized	-0.237322641	-0.438111969	-0.036128376	-0.24 (-0.44 to -0.04)
1095	Pakistan	YLLs (Years of Life Lost)	Both	Prostate cancer	All ages	-1.331451796	-1.527670542	-1.134842058	-1.33 (-1.53 to -1.13)
1096	Pakistan	YLLs (Years of Life Lost)	Both	Prostate cancer	Age-standardized	-1.158947181	-1.43430293	-0.882822191	-1.16 (-1.43 to -0.88)
1097	Angola	Deaths	Both	Prostate cancer	All ages	0.14656725	-0.195548696	0.489855924	0.15 (-0.2 to 0.49)
1098	Angola	Deaths	Both	Prostate cancer	Age-standardized	0.273162703	0.048512683	0.498317154	0.27 (0.05 to 0.5)
1099	Angola	DALYs (Disability-Adjusted Life Years)	Both	Prostate cancer	All ages	0.149810579	-0.205359969	0.506244902	0.15 (-0.21 to 0.51)
1100	Angola	DALYs (Disability-Adjusted Life Years)	Both	Prostate cancer	Age-standardized	0.273871561	0.03652138	0.511784887	0.27 (0.04 to 0.51)

1101	Angola	YLDs (Years Lived with Disability)	Both	Prostate cancer	All ages	1.2849300 8	0.8358377 74	1.7360225 08	1.28 (0.84 to 1.74)
1102	Angola	YLDs (Years Lived with Disability)	Both	Prostate cancer	Age-standardized	1.2798340 73	0.9549873 79	1.6057260 39	1.28 (0.95 to 1.61)
1103	Angola	YLLs (Years of Life Lost)	Both	Prostate cancer	All ages	0.1229462 39	-0.229635 207	0.4767736 82	0.12 (-0.23 to 0.48)
1104	Angola	YLLs (Years of Life Lost)	Both	Prostate cancer	Age-standardized	0.2505613 68	0.0154925 47	0.4861826 76	0.25 (0.02 to 0.49)
1105	Central African Republic	Deaths	Both	Prostate cancer	All ages	-1.229408 668	-1.661295 379	-0.795625 184	-1.23 (-1.66 to -0.8)
1106	Central African Republic	Deaths	Both	Prostate cancer	Age-standardized	-0.938314 9	-1.155014 315	-0.721140 411	-0.94 (-1.16 to -0.72)
1107	Central African Republic	DALYs (Disability-Adjusted Life Years)	Both	Prostate cancer	All ages	-1.081710 398	-1.537470 388	-0.623840 802	-1.08 (-1.54 to -0.62)
1108	Central African Republic	DALYs (Disability-Adjusted Life Years)	Both	Prostate cancer	Age-standardized	-0.902643 441	-1.142739 946	-0.661963 809	-0.9 (-1.14 to -0.66)

1109	Central African Republic	YLDs (Years Lived with Disability)	Both	Prostate cancer	All ages	-0.733594659	-1.182093668	-0.283060073	-0.73 (-1.18 to -0.28)
1110	Central African Republic	YLDs (Years Lived with Disability)	Both	Prostate cancer	Age-standardized	-0.600245384	-0.83598502	-0.363945332	-0.6 (-0.84 to -0.36)
1111	Central African Republic	YLLs (Years of Life Lost)	Both	Prostate cancer	All ages	-1.088819898	-1.54470632	-0.630822544	-1.09 (-1.54 to -0.63)
1112	Central African Republic	YLLs (Years of Life Lost)	Both	Prostate cancer	Age-standardized	-0.90875401	-1.148933833	-0.66799062	-0.91 (-1.15 to -0.67)
1113	Congo	Deaths	Both	Prostate cancer	All ages	0.709018499	0.277381023	1.142513931	0.71 (0.28 to 1.14)
1114	Congo	Deaths	Both	Prostate cancer	Age-standardized	0.724528086	0.41378266	1.03623516	0.72 (0.41 to 1.04)
1115	Congo	DALYs (Disability-Adjusted Life Years)	Both	Prostate cancer	All ages	0.714326697	0.256215261	1.17453143	0.71 (0.26 to 1.17)
1116	Congo	DALYs (Disability-Adjusted Life Years)	Both	Prostate cancer	Age-standardized	0.679602792	0.362943789	0.997260898	0.68 (0.36 to 1)

1117	Congo	YLDs (Years Lived with Disability)	Both	Prostate cancer	All ages	1.869531115	1.311063374	2.431077357	1.87 (1.31 to 2.43)
1118	Congo	YLDs (Years Lived with Disability)	Both	Prostate cancer	Age-standardized	1.741212043	1.33391122	2.150149968	1.74 (1.33 to 2.15)
1119	Congo	YLLs (Years of Life Lost)	Both	Prostate cancer	All ages	0.684613471	0.229468464	1.141825306	0.68 (0.23 to 1.14)
1120	Congo	YLLs (Years of Life Lost)	Both	Prostate cancer	Age-standardized	0.653069271	0.338958251	0.968163616	0.65 (0.34 to 0.97)
1121	Democratic Republic of the Congo	Deaths	Both	Prostate cancer	All ages	-0.885019662	-1.310879498	-0.457322171	-0.89 (-1.31 to -0.46)
1122	Democratic Republic of the Congo	Deaths	Both	Prostate cancer	Age-standardized	-0.899292934	-1.286621471	-0.510444608	-0.9 (-1.29 to -0.51)
1123	Democratic Republic of the Congo	DALYs (Disability-Adjusted Life Years)	Both	Prostate cancer	All ages	-0.704770764	-1.165733908	-0.241657689	-0.7 (-1.17 to -0.24)
1124	Democratic Republic of the Congo	DALYs (Disability-Adjusted Life Years)	Both	Prostate cancer	Age-standardized	-0.642878782	-1.021031024	-0.263281799	-0.64 (-1.02 to -0.26)

1125	Democratic Republic of the Congo	YLDs (Years Lived with Disability)	Both	Prostate cancer	All ages	0.138805208	-0.419107746	0.699843931	0.14 (-0.42 to 0.7)
1126	Democratic Republic of the Congo	YLDs (Years Lived with Disability)	Both	Prostate cancer	Age-standardized	0.147865134	-0.310836427	0.608677326	0.15 (-0.31 to 0.61)
1127	Democratic Republic of the Congo	YLLs (Years of Life Lost)	Both	Prostate cancer	All ages	-0.724886474	-1.183331702	-0.264314356	-0.72 (-1.18 to -0.26)
1128	Democratic Republic of the Congo	YLLs (Years of Life Lost)	Both	Prostate cancer	Age-standardized	-0.661356306	-1.037460703	-0.283822534	-0.66 (-1.04 to -0.28)
1129	Gabon	Deaths	Both	Prostate cancer	All ages	0.152522545	-0.014032375	0.319354909	0.15 (-0.01 to 0.32)
1130	Gabon	Deaths	Both	Prostate cancer	Age-standardized	0.439047925	0.400468286	0.47764239	0.44 (0.4 to 0.48)
1131	Gabon	DALYs (Disability-Adjusted Life Years)	Both	Prostate cancer	All ages	0.495316331	0.318547005	0.672397137	0.5 (0.32 to 0.67)
1132	Gabon	DALYs (Disability-Adjusted Life Years)	Both	Prostate cancer	Age-standardized	0.623135703	0.597133847	0.649144281	0.62 (0.6 to 0.65)
113	Gabon	YLDs (Years Lived with	Both	Prostate canc	All ages	2.038594891	1.758853654	2.319105154	2.04 (1.76 to

3		Disability)		er					2.32)
1134	Gabon	YLDs (Years Lived with Disability)	Both	Prostate cancer	Age-standardized	2.080523257	1.974164458	2.186992987	2.08 (1.97 to 2.19)
1135	Gabon	YLLs (Years of Life Lost)	Both	Prostate cancer	All ages	0.449862546	0.277012543	0.623010494	0.45 (0.28 to 0.62)
1136	Gabon	YLLs (Years of Life Lost)	Both	Prostate cancer	Age-standardized	0.581307705	0.556127143	0.606494573	0.58 (0.56 to 0.61)
1137	Equatorial Guinea	Deaths	Both	Prostate cancer	All ages	-0.997156147	-1.274885021	-0.71864598	-1 (-1.27 to -0.72)
1138	Equatorial Guinea	Deaths	Both	Prostate cancer	Age-standardized	0.108828022	-0.005181521	0.222967553	0.11 (-0.01 to 0.22)
1139	Equatorial Guinea	DALYs (Disability-Adjusted Life Years)	Both	Prostate cancer	All ages	-1.158500708	-1.458713615	-0.857373181	-1.16 (-1.46 to -0.86)
1140	Equatorial Guinea	DALYs (Disability-Adjusted Life Years)	Both	Prostate cancer	Age-standardized	-0.015968959	-0.139010523	0.107224207	-0.02 (-0.14 to 0.11)
1141	Equatorial Guinea	YLDs (Years Lived with Disability)	Both	Prostate cancer	All ages	1.391106188	1.067029804	1.71622174	1.39 (1.07 to 1.72

									)
1142	Equatorial Guinea	YLDs (Years Lived with Disability)	Both	Prostate cancer	Age-standardized	2.393910428	2.232752104	2.555322799	2.39 (2.23 to 2.56)
1143	Equatorial Guinea	YLLs (Years of Life Lost)	Both	Prostate cancer	All ages	-1.229351955	-1.527151643	-0.930651667	-1.23 (-1.53 to -0.93)
1144	Equatorial Guinea	YLLs (Years of Life Lost)	Both	Prostate cancer	Age-standardized	-0.081038704	-0.202171131	0.040240751	-0.08 (-0.2 to 0.04)
1145	Burundi	Deaths	Both	Prostate cancer	All ages	-2.821451742	-3.413586609	-2.22568672	-2.82 (-3.41 to -2.23)
1146	Burundi	Deaths	Both	Prostate cancer	Age-standardized	-2.087268946	-2.514022065	-1.65864768	-2.09 (-2.51 to -1.66)
1147	Burundi	DALYs (Disability-Adjusted Life Years)	Both	Prostate cancer	All ages	-2.579351752	-3.176784456	-1.978232681	-2.58 (-3.18 to -1.98)
1148	Burundi	DALYs (Disability-Adjusted Life Years)	Both	Prostate cancer	Age-standardized	-2.038692432	-2.471669758	-1.603792901	-2.04 (-2.47 to -1.6)

1149	Burundi	YLDs (Years Lived with Disability)	Both	Prostate cancer	All ages	-1.877776628	-2.482099406	-1.269708835	-1.88 (-2.48 to -1.27)
1150	Burundi	YLDs (Years Lived with Disability)	Both	Prostate cancer	Age-standardized	-1.393481688	-1.827010126	-0.958038803	-1.39 (-1.83 to -0.96)
1151	Burundi	YLLs (Years of Life Lost)	Both	Prostate cancer	All ages	-2.595555318	-3.19267324	-1.994754309	-2.6 (-3.19 to -1.99)
1152	Burundi	YLLs (Years of Life Lost)	Both	Prostate cancer	Age-standardized	-2.053458553	-2.486296439	-1.618699413	-2.05 (-2.49 to -1.62)
1153	Djibouti	Deaths	Both	Prostate cancer	All ages	1.019935829	0.86998567	1.170108898	1.02 (0.87 to 1.17)
1154	Djibouti	Deaths	Both	Prostate cancer	Age-standardized	-0.493889408	-0.554573459	-0.433168326	-0.49 (-0.55 to -0.43)
1155	Djibouti	DALYs (Disability-Adjusted Life Years)	Both	Prostate cancer	All ages	0.948768248	0.814063899	1.083652584	0.95 (0.81 to 1.08)
1156	Djibouti	DALYs (Disability-Adjusted Life Years)	Both	Prostate cancer	Age-standardized	-0.572869854	-0.635562214	-0.51013794	-0.57 (-0.64 to -0.5

1)

1157	Djibouti	YLDs (Years Lived with Disability)	Both	Prostate cancer	All ages	2.053390977	1.85216079	2.255018737	2.05 (1.85 to 2.26)
1158	Djibouti	YLDs (Years Lived with Disability)	Both	Prostate cancer	Age-standardized	0.361141819	0.218578029	0.50390841	0.36 (0.22 to 0.5)
1159	Djibouti	YLLs (Years of Life Lost)	Both	Prostate cancer	All ages	0.919892515	0.786629889	1.053331344	0.92 (0.79 to 1.05)
1160	Djibouti	YLLs (Years of Life Lost)	Both	Prostate cancer	Age-standardized	-0.596762754	-0.657788342	-0.535699679	-0.6 (-0.66 to -0.54)
1161	Comoros	Deaths	Both	Prostate cancer	All ages	0.329927252	0.199152822	0.460872361	0.33 (0.2 to 0.46)
1162	Comoros	Deaths	Both	Prostate cancer	Age-standardized	-1.088867031	-1.191739035	-0.985887924	-1.09 (-1.19 to -0.99)
1163	Comoros	DALYs (Disability-Adjusted Life Years)	Both	Prostate cancer	All ages	0.009758213	-0.158971089	0.178772664	0.01 (-0.16 to 0.18)
1164	Comoros	DALYs (Disability-Adjusted Life Years)	Both	Prostate cancer	Age-standardized	-1.337544257	-1.472064143	-1.202840712	-1.34 (-1.47 to -1.2)

1165	Comoros	YLDs (Years Lived with Disability)	Both	Prostate cancer	All ages	0.960809515	0.827473344	1.094322012	0.96 (0.83 to 1.09)
1166	Comoros	YLDs (Years Lived with Disability)	Both	Prostate cancer	Age-standardized	-0.463217872	-0.560582915	-0.365757494	-0.46 (-0.56 to -0.37)
1167	Comoros	YLLs (Years of Life Lost)	Both	Prostate cancer	All ages	-0.013841732	-0.184000293	0.156606901	-0.01 (-0.18 to 0.16)
1168	Comoros	YLLs (Years of Life Lost)	Both	Prostate cancer	Age-standardized	-1.358908937	-1.494906149	-1.222723966	-1.36 (-1.49 to -1.22)
1169	Eritrea	Deaths	Both	Prostate cancer	All ages	-0.152915533	-0.297349408	-0.008272424	-0.15 (-0.3 to -0.01)
1170	Eritrea	Deaths	Both	Prostate cancer	Age-standardized	-0.713004376	-0.803735665	-0.622190097	-0.71 (-0.8 to -0.62)
1171	Eritrea	DALYs (Disability-Adjusted Life Years)	Both	Prostate cancer	All ages	-0.376689968	-0.54275575	-0.210346902	-0.38 (-0.54 to -0.21)
117	Eritrea	DALYs (Disability-Adjusted	Both	Prostate canc	Age-standardized	-0.903336101	-1.001935048	-0.804638953	-0.9 (-1 to

2		Life Years)		er					-0.8)
1173	Eritrea	YLDs (Years Lived with Disability)	Both	Prostate cancer	All ages	0.368882427	0.187434051	0.550659423	0.37 (0.19 to 0.55)
1174	Eritrea	YLDs (Years Lived with Disability)	Both	Prostate cancer	Age-standardized	-0.232159239	-0.352027448	-0.112146839	-0.23 (-0.35 to -0.11)
1175	Eritrea	YLLs (Years of Life Lost)	Both	Prostate cancer	All ages	-0.393715831	-0.559397472	-0.227758141	-0.39 (-0.56 to -0.23)
1176	Eritrea	YLLs (Years of Life Lost)	Both	Prostate cancer	Age-standardized	-0.918629683	-1.016790995	-0.820371025	-0.92 (-1.02 to -0.82)
1177	Ethiopia	Deaths	Both	Prostate cancer	All ages	-0.436059309	-0.865971174	-0.004283058	-0.44 (-0.87 to 0)
1178	Ethiopia	Deaths	Both	Prostate cancer	Age-standardized	-0.441131696	-0.708275986	-0.173268655	-0.44 (-0.71 to -0.17)
1179	Ethiopia	DALYs (Disability-Adjusted Life Years)	Both	Prostate cancer	All ages	-0.832496254	-1.252547963	-0.41065773	-0.83 (-1.25 to -0.41)

1180	Ethiopia	DALYs (Disability-Adjusted Life Years)	Both	Prostate cancer	Age-standardized	-0.767893949	-1.041152657	-0.493880683	-0.77 (-1.04 to -0.49)
1181	Ethiopia	YLDs (Years Lived with Disability)	Both	Prostate cancer	All ages	0.522866559	0.011093359	1.037258586	0.52 (0.01 to 1.04)
1182	Ethiopia	YLDs (Years Lived with Disability)	Both	Prostate cancer	Age-standardized	0.510683017	0.148213598	0.874464332	0.51 (0.15 to 0.87)
1183	Ethiopia	YLLs (Years of Life Lost)	Both	Prostate cancer	All ages	-0.864873502	-1.282255035	-0.445727268	-0.86 (-1.28 to -0.45)
1184	Ethiopia	YLLs (Years of Life Lost)	Both	Prostate cancer	Age-standardized	-0.798183017	-1.068928518	-0.526696565	-0.8 (-1.07 to -0.53)
1185	Kenya	Deaths	Both	Prostate cancer	All ages	0.451747212	0.238339928	0.665608839	0.45 (0.24 to 0.67)
1186	Kenya	Deaths	Both	Prostate cancer	Age-standardized	-0.291388039	-0.394301305	-0.188368442	-0.29 (-0.39 to -0.19)
1187	Kenya	DALYs (Disability-Adjusted Life Years)	Both	Prostate cancer	All ages	0.628609399	0.431755064	0.825849584	0.63 (0.43 to 0.83)

1188	Kenya	DALYs (Disability-Adjusted Life Years)	Both	Prostate cancer	Age-standardized	-0.180487343	-0.254418098	-0.106501791	-0.18 (-0.25 to -0.11)
1189	Kenya	YLDs (Years Lived with Disability)	Both	Prostate cancer	All ages	1.288461658	1.012993773	1.564680759	1.29 (1.01 to 1.56)
1190	Kenya	YLDs (Years Lived with Disability)	Both	Prostate cancer	Age-standardized	0.403249985	0.276166344	0.530494683	0.4 (0.28 to 0.53)
1191	Kenya	YLLs (Years of Life Lost)	Both	Prostate cancer	All ages	0.609191995	0.414510577	0.804250857	0.61 (0.41 to 0.8)
1192	Kenya	YLLs (Years of Life Lost)	Both	Prostate cancer	Age-standardized	-0.197307633	-0.271074159	-0.123486544	-0.2 (-0.27 to -0.12)
1193	Madagascar	Deaths	Both	Prostate cancer	All ages	-4.025512409	-4.735245141	-3.310492091	-4.03 (-4.74 to -3.31)
1194	Madagascar	Deaths	Both	Prostate cancer	Age-standardized	-3.396948006	-3.886282152	-2.905122562	-3.4 (-3.89 to -2.91)
1195	Madagascar	DALYs (Disability-Adjusted Life Years)	Both	Prostate cancer	All ages	-3.725505698	-4.425243841	-3.020644514	-3.73 (-4.43 to -3.02)

1196	Madagascar	DALYs (Disability-Adjusted Life Years)	Both	Prostate cancer	Age-standardized	-3.373164286	-3.858729368	-2.885146839	-3.37 (-3.86 to -2.89)
1197	Madagascar	YLDs (Years Lived with Disability)	Both	Prostate cancer	All ages	-2.91050775	-3.604093652	-2.211931372	-2.91 (-3.6 to -2.21)
1198	Madagascar	YLDs (Years Lived with Disability)	Both	Prostate cancer	Age-standardized	-2.659973917	-3.128866762	-2.188811453	-2.66 (-3.13 to -2.19)
1199	Madagascar	YLLs (Years of Life Lost)	Both	Prostate cancer	All ages	-3.74540839	-4.445038206	-3.040656057	-3.75 (-4.45 to -3.04)
1200	Madagascar	YLLs (Years of Life Lost)	Both	Prostate cancer	Age-standardized	-3.390253317	-3.876034202	-2.902017445	-3.39 (-3.88 to -2.9)
1201	Malawi	Deaths	Both	Prostate cancer	All ages	0.519209974	0.351725698	0.686973777	0.52 (0.35 to 0.69)
1202	Malawi	Deaths	Both	Prostate cancer	Age-standardized	0.610426396	0.493856117	0.727131893	0.61 (0.49 to 0.73)
1203	Malawi	DALYs (Disability-Adjusted Life Years)	Both	Prostate cancer	All ages	0.653868261	0.499000574	0.808974596	0.65 (0.5 to 0.81)

1204	Malawi	DALYs (Disability-Adjusted Life Years)	Both	Prostate cancer	Age-standardized	0.831804582	0.690172943	0.973635441	0.83 (0.69 to 0.97)
1205	Malawi	YLDs (Years Lived with Disability)	Both	Prostate cancer	All ages	1.598654283	1.458543424	1.738958629	1.6 (1.46 to 1.74)
1206	Malawi	YLDs (Years Lived with Disability)	Both	Prostate cancer	Age-standardized	1.720598136	1.598226563	1.843117101	1.72 (1.6 to 1.84)
1207	Malawi	YLLs (Years of Life Lost)	Both	Prostate cancer	All ages	0.630188412	0.474741726	0.785875592	0.63 (0.47 to 0.79)
1208	Malawi	YLLs (Years of Life Lost)	Both	Prostate cancer	Age-standardized	0.809948537	0.667551657	0.952546841	0.81 (0.67 to 0.95)
1209	Mauritius	Deaths	Both	Prostate cancer	All ages	3.132657847	2.582008155	3.68626337	3.13 (2.58 to 3.69)
1210	Mauritius	Deaths	Both	Prostate cancer	Age-standardized	0.242292042	-0.156618261	0.642796136	0.24 (-0.16 to 0.64)
1211	Mauritius	DALYs (Disability-Adjusted Life Years)	Both	Prostate cancer	All ages	3.003681805	2.409019325	3.601797336	3 (2.41 to 3.6)
1212	Mauritius	DALYs (Disability-Adjusted Life Years)	Both	Prostate cancer	Age-standardized	0.183888797	-0.241416941	0.611007762	0.18 (-0.24 to 0.61)

1213	Mauritius	YLDs (Years Lived with Disability)	Both	Prostate cancer	All ages	4.090662868	3.543835004	4.640378599	4.09 (3.54 to 4.64)
1214	Mauritius	YLDs (Years Lived with Disability)	Both	Prostate cancer	Age-standardized	1.245528844	0.846552537	1.64608361	1.25 (0.85 to 1.65)
1215	Mauritius	YLLs (Years of Life Lost)	Both	Prostate cancer	All ages	2.942345249	2.344826459	3.543352528	2.94 (2.34 to 3.54)
1216	Mauritius	YLLs (Years of Life Lost)	Both	Prostate cancer	Age-standardized	0.125658434	-0.302441838	0.555596965	0.13 (-0.3 to 0.56)
1217	Mozambique	Deaths	Both	Prostate cancer	All ages	-0.666655781	-0.837825515	-0.495190581	-0.67 (-0.84 to -0.5)
1218	Mozambique	Deaths	Both	Prostate cancer	Age-standardized	0.025869782	-0.125496774	0.177465744	0.03 (-0.13 to 0.18)
1219	Mozambique	DALYs (Disability-Adjusted Life Years)	Both	Prostate cancer	All ages	-0.385419677	-0.584809353	-0.18563011	-0.39 (-0.58 to -0.19)
1220	Mozambique	DALYs (Disability-Adjusted Life Years)	Both	Prostate cancer	Age-standardized	0.296438343	0.154137464	0.438941407	0.3 (0.15 to 0.44)
122	Mozambique	YLDs (Years Lived with	Both	Prostate canc	All ages	0.273389542	0.060537726	0.486694143	0.27 (0.06 to

1		Disability)		er					0.49)
1222	Mozambique	YLDs (Years Lived with Disability)	Both	Prostate cancer	Age-standardized	0.887658928	0.737388699	1.038153315	0.89 (0.74 to 1.04)
1223	Mozambique	YLLs (Years of Life Lost)	Both	Prostate cancer	All ages	-0.401384861	-0.600375994	-0.201995361	-0.4 (-0.6 to -0.2)
1224	Mozambique	YLLs (Years of Life Lost)	Both	Prostate cancer	Age-standardized	0.282455063	0.140362843	0.424748901	0.28 (0.14 to 0.42)
1225	Mexico	Deaths	Both	Prostate cancer	All ages	-0.828249885	-1.097222861	-0.558545417	-0.83 (-1.1 to -0.56)
1226	Mexico	Deaths	Both	Prostate cancer	Age-standardized	-3.438416086	-3.75392156	-3.121876349	-3.44 (-3.75 to -3.12)
1227	Mexico	DALYs (Disability-Adjusted Life Years)	Both	Prostate cancer	All ages	-0.789959318	-1.027849241	-0.551497601	-0.79 (-1.03 to -0.55)
1228	Mexico	DALYs (Disability-Adjusted Life Years)	Both	Prostate cancer	Age-standardized	-3.220614008	-3.512504586	-2.927840413	-3.22 (-3.51 to -2.93)
122	Mexico	YLDs (Years Lived with	Both	Prostate canc	All ages	0.931129776	0.523446988	1.340465961	0.93 (0.52 to

9		Disability)		er					1.34)
1230	Mexico	YLDs (Years Lived with Disability)	Both	Prostate cancer	Age-standardized	-1.457022256	-1.913968511	-0.997947259	-1.46 (-1.91 to -1)
1231	Mexico	YLLs (Years of Life Lost)	Both	Prostate cancer	All ages	-0.953209018	-1.180549702	-0.725345322	-0.95 (-1.18 to -0.73)
1232	Mexico	YLLs (Years of Life Lost)	Both	Prostate cancer	Age-standardized	-3.382112238	-3.664551784	-3.098844626	-3.38 (-3.66 to -3.1)
1233	Rwanda	Deaths	Both	Prostate cancer	All ages	-0.219027088	-0.659222581	0.223118986	-0.22 (-0.66 to 0.22)
1234	Rwanda	Deaths	Both	Prostate cancer	Age-standardized	-0.744915606	-0.88971095	-0.599908722	-0.74 (-0.89 to -0.6)
1235	Rwanda	DALYs (Disability-Adjusted Life Years)	Both	Prostate cancer	All ages	-0.244485707	-0.689048869	0.202067532	-0.24 (-0.69 to 0.2)
1236	Rwanda	DALYs (Disability-Adjusted Life Years)	Both	Prostate cancer	Age-standardized	-0.818672595	-0.967573203	-0.669548108	-0.82 (-0.97 to -0.67)
123	Rwanda	YLDs (Years Lived with	Both	Prostate canc	All ages	1.121270846	0.585561572	1.659833258	1.12 (0.59 to

7		Disability)		er					1.66)
1238	Rwanda	YLDs (Years Lived with Disability)	Both	Prostate cancer	Age-standardized	0.417995781	0.240364502	0.595941832	0.42 (0.24 to 0.6)
1239	Rwanda	YLLs (Years of Life Lost)	Both	Prostate cancer	All ages	-0.277670322	-0.719679442	0.166306682	-0.28 (-0.72 to 0.17)
1240	Rwanda	YLLs (Years of Life Lost)	Both	Prostate cancer	Age-standardized	-0.848096644	-0.996840821	-0.699128991	-0.85 (-1 to -0.7)
1241	Somalia	Deaths	Both	Prostate cancer	All ages	-1.318467062	-1.479294372	-1.157377214	-1.32 (-1.48 to -1.16)
1242	Somalia	Deaths	Both	Prostate cancer	Age-standardized	-1.337477712	-1.462949533	-1.211846122	-1.34 (-1.46 to -1.21)
1243	Somalia	DALYs (Disability-Adjusted Life Years)	Both	Prostate cancer	All ages	-1.342475125	-1.477044052	-1.207722394	-1.34 (-1.48 to -1.21)
1244	Somalia	DALYs (Disability-Adjusted Life Years)	Both	Prostate cancer	Age-standardized	-1.276991275	-1.395748964	-1.158090556	-1.28 (-1.4 to -1.16)

1245	Somalia	YLDs (Years Lived with Disability)	Both	Prostate cancer	All ages	-0.958323458	-1.086230702	-0.830250815	-0.96 (-1.09 to -0.83)
1246	Somalia	YLDs (Years Lived with Disability)	Both	Prostate cancer	Age-standardized	-0.977932966	-1.087827943	-0.867915892	-0.98 (-1.09 to -0.87)
1247	Somalia	YLLs (Years of Life Lost)	Both	Prostate cancer	All ages	-1.350670298	-1.485414315	-1.215741984	-1.35 (-1.49 to -1.22)
1248	Somalia	YLLs (Years of Life Lost)	Both	Prostate cancer	Age-standardized	-1.283366242	-1.402316153	-1.164272827	-1.28 (-1.4 to -1.16)
1249	Seychelles	Deaths	Both	Prostate cancer	All ages	0.970064033	0.723982266	1.21674701	0.97 (0.72 to 1.22)
1250	Seychelles	Deaths	Both	Prostate cancer	Age-standardized	0.378208509	0.070462965	0.686900459	0.38 (0.07 to 0.69)
1251	Seychelles	DALYs (Disability-Adjusted Life Years)	Both	Prostate cancer	All ages	1.174017368	0.957624657	1.390873896	1.17 (0.96 to 1.39)
1252	Seychelles	DALYs (Disability-Adjusted Life Years)	Both	Prostate cancer	Age-standardized	0.322804099	0.020422811	0.626099544	0.32 (0.02 to 0.63)

1253	Seychelles	YLDs (Years Lived with Disability)	Both	Prostate cancer	All ages	2.688438602	2.392246291	2.985487714	2.69 (2.39 to 2.99)
1254	Seychelles	YLDs (Years Lived with Disability)	Both	Prostate cancer	Age-standardized	1.779565757	1.373885194	2.186869782	1.78 (1.37 to 2.19)
1255	Seychelles	YLLs (Years of Life Lost)	Both	Prostate cancer	All ages	1.100794938	0.886466331	1.315578877	1.1 (0.89 to 1.32)
1256	Seychelles	YLLs (Years of Life Lost)	Both	Prostate cancer	Age-standardized	0.253123336	-0.046109853	0.553252343	0.25 (-0.05 to 0.55)
1257	United Republic of Tanzania	Deaths	Both	Prostate cancer	All ages	-1.567000505	-1.834408927	-1.298863648	-1.57 (-1.83 to -1.3)
1258	United Republic of Tanzania	Deaths	Both	Prostate cancer	Age-standardized	-1.613621244	-1.835412974	-1.391328401	-1.61 (-1.84 to -1.39)
1259	United Republic of Tanzania	DALYs (Disability-Adjusted Life Years)	Both	Prostate cancer	All ages	-1.53299751	-1.807074307	-1.258155708	-1.53 (-1.81 to -1.26)
1260	United Republic of Tanzania	DALYs (Disability-Adjusted Life Years)	Both	Prostate cancer	Age-standardized	-1.545030353	-1.758966279	-1.330628546	-1.55 (-1.76 to -1.33)

1261	United Republic of Tanzania	YLDs (Years Lived with Disability)	Both	Prostate cancer	All ages	-0.65130484	-0.947846823	-0.353875072	-0.65 (-0.95 to -0.35)
1262	United Republic of Tanzania	YLDs (Years Lived with Disability)	Both	Prostate cancer	Age-standardized	-0.686218152	-0.922061616	-0.449813292	-0.69 (-0.92 to -0.45)
1263	United Republic of Tanzania	YLLs (Years of Life Lost)	Both	Prostate cancer	All ages	-1.556396314	-1.829605989	-1.282426292	-1.56 (-1.83 to -1.28)
1264	United Republic of Tanzania	YLLs (Years of Life Lost)	Both	Prostate cancer	Age-standardized	-1.56754395	-1.780679766	-1.35394563	-1.57 (-1.78 to -1.35)
1265	Zambia	Deaths	Both	Prostate cancer	All ages	0.633894321	0.102310447	1.16830112	0.63 (0.1 to 1.17)
1266	Zambia	Deaths	Both	Prostate cancer	Age-standardized	0.670152755	0.243638365	1.098481868	0.67 (0.24 to 1.1)
1267	Zambia	DALYs (Disability-Adjusted Life Years)	Both	Prostate cancer	All ages	0.957589381	0.361588187	1.557129952	0.96 (0.36 to 1.56)
1268	Zambia	DALYs (Disability-Adjusted Life Years)	Both	Prostate cancer	Age-standardized	0.991874003	0.515819997	1.470182653	0.99 (0.52 to 1.47)

1269	Zambia	YLDs (Years Lived with Disability)	Both	Prostate cancer	All ages	2.072574112	1.378243192	2.771660445	2.07 (1.38 to 2.77)
1270	Zambia	YLDs (Years Lived with Disability)	Both	Prostate cancer	Age-standardized	2.074360007	1.500191329	2.651776657	2.07 (1.5 to 2.65)
1271	Zambia	YLLs (Years of Life Lost)	Both	Prostate cancer	All ages	0.929337805	0.336181205	1.526000963	0.93 (0.34 to 1.53)
1272	Zambia	YLLs (Years of Life Lost)	Both	Prostate cancer	Age-standardized	0.964710934	0.491385335	1.440265949	0.96 (0.49 to 1.44)
1273	Uganda	Deaths	Both	Prostate cancer	All ages	-1.448232987	-1.746717307	-1.1488419	-1.45 (-1.75 to -1.15)
1274	Uganda	Deaths	Both	Prostate cancer	Age-standardized	-1.104501402	-1.417470591	-0.790538632	-1.1 (-1.42 to -0.79)
1275	Uganda	DALYs (Disability-Adjusted Life Years)	Both	Prostate cancer	All ages	-1.343569401	-1.656991968	-1.029147946	-1.34 (-1.66 to -1.03)
1276	Uganda	DALYs (Disability-Adjusted Life Years)	Both	Prostate cancer	Age-standardized	-0.997398416	-1.328315542	-0.665371487	-1 (-1.33 to -0.67)

1277	Uganda	YLDs (Years Lived with Disability)	Both	Prostate cancer	All ages	-0.347868866	-0.633433634	-0.061483428	-0.35 (-0.63 to -0.06)
1278	Uganda	YLDs (Years Lived with Disability)	Both	Prostate cancer	Age-standardized	-0.03377718	-0.316993564	0.250243871	-0.03 (-0.32 to 0.25)
1279	Uganda	YLLs (Years of Life Lost)	Both	Prostate cancer	All ages	-1.369424339	-1.683566581	-1.054278345	-1.37 (-1.68 to -1.05)
1280	Uganda	YLLs (Years of Life Lost)	Both	Prostate cancer	Age-standardized	-1.022038946	-1.354280643	-0.688678248	-1.02 (-1.35 to -0.69)
1281	Botswana	Deaths	Both	Prostate cancer	All ages	0.06397600 8	-0.078052146	0.206206041	0.06 (-0.08 to 0.21)
1282	Botswana	Deaths	Both	Prostate cancer	Age-standardized	-1.074663458	-1.204542955	-0.944613217	-1.07 (-1.2 to -0.94)
1283	Botswana	DALYs (Disability-Adjusted Life Years)	Both	Prostate cancer	All ages	0.085038105	-0.061812713	0.232104709	0.09 (-0.06 to 0.23)
1284	Botswana	DALYs (Disability-Adjusted Life Years)	Both	Prostate cancer	Age-standardized	-0.975914598	-1.136340172	-0.815228701	-0.98 (-1.14 to

									-0.82)
1285	Botswana	YLDs (Years Lived with Disability)	Both	Prostate cancer	All ages	1.240298014	1.090309101	1.390509468	1.24 (1.09 to 1.39)
1286	Botswana	YLDs (Years Lived with Disability)	Both	Prostate cancer	Age-standardized	0.139683585	0.033470443	0.246009501	0.14 (0.03 to 0.25)
1287	Botswana	YLLs (Years of Life Lost)	Both	Prostate cancer	All ages	0.050404562	-0.097367643	0.198395346	0.05 (-0.1 to 0.2)
1288	Botswana	YLLs (Years of Life Lost)	Both	Prostate cancer	Age-standardized	-1.008493631	-1.171242386	-0.845476866	-1.01 (-1.17 to -0.85)
1289	Namibia	Deaths	Both	Prostate cancer	All ages	1.292799238	1.138010649	1.447824726	1.29 (1.14 to 1.45)
1290	Namibia	Deaths	Both	Prostate cancer	Age-standardized	0.525063234	0.396381442	0.653909963	0.53 (0.4 to 0.65)
1291	Namibia	DALYs (Disability-Adjusted Life Years)	Both	Prostate cancer	All ages	1.082118825	0.940983749	1.223451236	1.08 (0.94 to 1.22)
1292	Namibia	DALYs (Disability-Adjusted Life Years)	Both	Prostate cancer	Age-standardized	0.493428484	0.375823924	0.611170835	0.49 (0.38 to 0.61)

1293	Namibia	YLDs (Years Lived with Disability)	Both	Prostate cancer	All ages	2.361366436	2.153369899	2.569786478	2.36 (2.15 to 2.57)
1294	Namibia	YLDs (Years Lived with Disability)	Both	Prostate cancer	Age-standardized	1.701214144	1.573734139	1.828854143	1.7 (1.57 to 1.83)
1295	Namibia	YLLs (Years of Life Lost)	Both	Prostate cancer	All ages	1.043571792	0.904454387	1.182880999	1.04 (0.9 to 1.18)
1296	Namibia	YLLs (Years of Life Lost)	Both	Prostate cancer	Age-standardized	0.458275665	0.339753677	0.576937651	0.46 (0.34 to 0.58)
1297	Eswatini	Deaths	Both	Prostate cancer	All ages	0.387902486	0.262781796	0.513179318	0.39 (0.26 to 0.51)
1298	Eswatini	Deaths	Both	Prostate cancer	Age-standardized	-0.664610553	-0.873163272	-0.45561906	-0.66 (-0.87 to -0.46)
1299	Eswatini	DALYs (Disability-Adjusted Life Years)	Both	Prostate cancer	All ages	0.491346235	0.324338429	0.658632055	0.49 (0.32 to 0.66)
1300	Eswatini	DALYs (Disability-Adjusted Life Years)	Both	Prostate cancer	Age-standardized	-0.669936189	-0.866021534	-0.473462991	-0.67 (-0.87 to -0.47)

1301	Eswatini	YLDs (Years Lived with Disability)	Both	Prostate cancer	All ages	1.288442422	1.125094441	1.45205426	1.29 (1.13 to 1.45)
1302	Eswatini	YLDs (Years Lived with Disability)	Both	Prostate cancer	Age-standardized	-0.017644943	-0.172641879	0.137592649	-0.02 (-0.17 to 0.14)
1303	Eswatini	YLLs (Years of Life Lost)	Both	Prostate cancer	All ages	0.468820316	0.300874841	0.637047001	0.47 (0.3 to 0.64)
1304	Eswatini	YLLs (Years of Life Lost)	Both	Prostate cancer	Age-standardized	-0.687778724	-0.885651783	-0.489510629	-0.69 (-0.89 to -0.49)
1305	Lesotho	Deaths	Both	Prostate cancer	All ages	1.819248358	1.749364959	1.889179755	1.82 (1.75 to 1.89)
1306	Lesotho	Deaths	Both	Prostate cancer	Age-standardized	1.790906712	1.68072079	1.901212036	1.79 (1.68 to 1.9)
1307	Lesotho	DALYs (Disability-Adjusted Life Years)	Both	Prostate cancer	All ages	2.261293109	2.156398031	2.366295893	2.26 (2.16 to 2.37)
1308	Lesotho	DALYs (Disability-Adjusted Life Years)	Both	Prostate cancer	Age-standardized	2.015543765	1.905684216	2.125521749	2.02 (1.91 to 2.13)
130	Lesotho	YLDs (Years Lived with	Both	Prostate canc	All ages	2.678297096	2.567130881	2.789583796	2.68 (2.57 to

9		Disability)		er					2.79)
1310	Lesotho	YLDs (Years Lived with Disability)	Both	Prostate cancer	Age-standardized	2.343046216	2.231281822	2.454932796	2.34 (2.23 to 2.45)
1311	Lesotho	YLLs (Years of Life Lost)	Both	Prostate cancer	All ages	2.250556469	2.145111678	2.356110111	2.25 (2.15 to 2.36)
1312	Lesotho	YLLs (Years of Life Lost)	Both	Prostate cancer	Age-standardized	2.007243734	1.896722066	2.11788528	2.01 (1.9 to 2.12)
1313	South Africa	Deaths	Both	Prostate cancer	All ages	-1.128098224	-1.292205363	-0.963718249	-1.13 (-1.29 to -0.96)
1314	South Africa	Deaths	Both	Prostate cancer	Age-standardized	-2.53654058	-2.61280107	-2.460220374	-2.54 (-2.61 to -2.46)
1315	South Africa	DALYs (Disability-Adjusted Life Years)	Both	Prostate cancer	All ages	-0.596688162	-0.768636487	-0.424441886	-0.6 (-0.77 to -0.42)
1316	South Africa	DALYs (Disability-Adjusted Life Years)	Both	Prostate cancer	Age-standardized	-2.02676849	-2.1145814	-1.938876803	-2.03 (-2.11 to -1.94)
131	South Africa	YLDs (Years Lived with	Both	Prostate canc	All ages	0.998040975	0.71972401	1.277127007	1 (0.72 to

7		Disability)		er					1.28)
1318	South Africa	YLDs (Years Lived with Disability)	Both	Prostate cancer	Age-standardized	-0.505791783	-0.692250887	-0.318982585	-0.51 (-0.69 to -0.32)
1319	South Africa	YLLs (Years of Life Lost)	Both	Prostate cancer	All ages	-0.652903403	-0.820491016	-0.485032611	-0.65 (-0.82 to -0.49)
1320	South Africa	YLLs (Years of Life Lost)	Both	Prostate cancer	Age-standardized	-2.0791656	-2.163842262	-1.994415651	-2.08 (-2.16 to -1.99)
1321	Zimbabwe	Deaths	Both	Prostate cancer	All ages	0.64609441	0.309742472	0.983574182	0.65 (0.31 to 0.98)
1322	Zimbabwe	Deaths	Both	Prostate cancer	Age-standardized	0.822046623	0.503748557	1.141352748	0.82 (0.5 to 1.14)
1323	Zimbabwe	DALYs (Disability-Adjusted Life Years)	Both	Prostate cancer	All ages	0.770269973	0.467320379	1.074133083	0.77 (0.47 to 1.07)
1324	Zimbabwe	DALYs (Disability-Adjusted Life Years)	Both	Prostate cancer	Age-standardized	0.690085099	0.361167042	1.020081135	0.69 (0.36 to 1.02)
132	Zimbabwe	YLDs (Years Lived with	Both	Prostate canc	All ages	0.499347612	0.060456218	0.9401641	0.5 (0.06 to

5		Disability)		er					0.94)
1326	Zimbabwe	YLDs (Years Lived with Disability)	Both	Prostate cancer	Age-standardized	0.358494849	-0.073036312	0.791889562	0.36 (-0.07 to 0.79)
1327	Zimbabwe	YLLs (Years of Life Lost)	Both	Prostate cancer	All ages	0.778108621	0.478077816	1.079035329	0.78 (0.48 to 1.08)
1328	Zimbabwe	YLLs (Years of Life Lost)	Both	Prostate cancer	Age-standardized	0.699390738	0.372115279	1.027733318	0.7 (0.37 to 1.03)
1329	Burkina Faso	Deaths	Both	Prostate cancer	All ages	1.186387996	0.873850646	1.499893681	1.19 (0.87 to 1.5)
1330	Burkina Faso	Deaths	Both	Prostate cancer	Age-standardized	1.679038037	1.437404324	1.921247344	1.68 (1.44 to 1.92)
1331	Burkina Faso	DALYs (Disability-Adjusted Life Years)	Both	Prostate cancer	All ages	1.097768231	0.757561552	1.439123615	1.1 (0.76 to 1.44)
1332	Burkina Faso	DALYs (Disability-Adjusted Life Years)	Both	Prostate cancer	Age-standardized	1.57630974	1.318551701	1.834723526	1.58 (1.32 to 1.83)
1333	Burkina Faso	YLDs (Years Lived with Disability)	Both	Prostate cancer	All ages	1.798541978	1.505647621	2.092281481	1.8 (1.51 to 2.09)

1334	Burkina Faso	YLDs (Years Lived with Disability)	Both	Prostate cancer	Age-standardized	2.23866195	2.016606122	2.461201119	2.24 (2.02 to 2.46)
1335	Burkina Faso	YLLs (Years of Life Lost)	Both	Prostate cancer	All ages	1.080120218	0.738758885	1.422638281	1.08 (0.74 to 1.42)
1336	Burkina Faso	YLLs (Years of Life Lost)	Both	Prostate cancer	Age-standardized	1.559851624	1.301191126	1.81917258	1.56 (1.3 to 1.82)
1337	Cabo Verde	Deaths	Both	Prostate cancer	All ages	0.460086007	0.059311368	0.862465896	0.46 (0.06 to 0.86)
1338	Cabo Verde	Deaths	Both	Prostate cancer	Age-standardized	0.114060227	-0.296113283	0.525921158	0.11 (-0.3 to 0.53)
1339	Cabo Verde	DALYs (Disability-Adjusted Life Years)	Both	Prostate cancer	All ages	0.909465319	0.50416477	1.316400312	0.91 (0.5 to 1.32)
1340	Cabo Verde	DALYs (Disability-Adjusted Life Years)	Both	Prostate cancer	Age-standardized	0.316508538	-0.077878998	0.712452701	0.32 (-0.08 to 0.71)
1341	Cabo Verde	YLDs (Years Lived with Disability)	Both	Prostate cancer	All ages	3.01336617	2.615797406	3.412475252	3.01 (2.62 to 3.41)
1342	Cabo Verde	YLDs (Years Lived with Disability)	Both	Prostate cancer	Age-standardized	2.400936243	2.077556849	2.725340096	2.4 (2.08 to 2.73

									)
1343	Cabo Verde	YLLs (Years of Life Lost)	Both	Prostate cancer	All ages	0.82865153	0.423736891	1.235198809	0.83 (0.42 to 1.24)
1344	Cabo Verde	YLLs (Years of Life Lost)	Both	Prostate cancer	Age-standardized	0.236241469	-0.161245698	0.635311149	0.24 (-0.16 to 0.64)
1345	Benin	Deaths	Both	Prostate cancer	All ages	-0.482662554	-0.587977882	-0.377235658	-0.48 (-0.59 to -0.38)
1346	Benin	Deaths	Both	Prostate cancer	Age-standardized	-0.014064894	-0.180968719	0.153118004	-0.01 (-0.18 to 0.15)
1347	Benin	DALYs (Disability-Adjusted Life Years)	Both	Prostate cancer	All ages	-0.342122938	-0.446828932	-0.23730682	-0.34 (-0.45 to -0.24)
1348	Benin	DALYs (Disability-Adjusted Life Years)	Both	Prostate cancer	Age-standardized	0.02524643	-0.141310272	0.192080936	0.03 (-0.14 to 0.19)
1349	Benin	YLDs (Years Lived with Disability)	Both	Prostate cancer	All ages	0.559314095	0.451987584	0.666755277	0.56 (0.45 to 0.67)
135	Benin	YLDs (Years Lived with	Both	Prostate canc	Age-standardized	0.882891796	0.717166471	1.048889814	0.88 (0.72 to

0		Disability)		er					1.05)
1351	Benin	YLLs (Years of Life Lost)	Both	Prostate cancer	All ages	-0.364830383	-0.469778749	-0.259771356	-0.36 (-0.47 to -0.26)
1352	Benin	YLLs (Years of Life Lost)	Both	Prostate cancer	Age-standardized	0.003848827	-0.163021882	0.170998449	0 (-0.16 to 0.17)
1353	Cameroon	Deaths	Both	Prostate cancer	All ages	0.50233157	0.403483208	0.601277248	0.5 (0.4 to 0.6)
1354	Cameroon	Deaths	Both	Prostate cancer	Age-standardized	0.855350026	0.706530379	1.004389591	0.86 (0.71 to 1)
1355	Cameroon	DALYs (Disability-Adjusted Life Years)	Both	Prostate cancer	All ages	0.506436329	0.404922783	0.60805251	0.51 (0.4 to 0.61)
1356	Cameroon	DALYs (Disability-Adjusted Life Years)	Both	Prostate cancer	Age-standardized	0.880764422	0.711487245	1.050326122	0.88 (0.71 to 1.05)
1357	Cameroon	YLDs (Years Lived with Disability)	Both	Prostate cancer	All ages	1.528881351	1.447874032	1.609953356	1.53 (1.45 to 1.61)
1358	Cameroon	YLDs (Years Lived with Disability)	Both	Prostate cancer	Age-standardized	1.846563663	1.718321467	1.974967542	1.85 (1.72 to 1.97)

1359	Cameroon	YLLs (Years of Life Lost)	Both	Prostate cancer	All ages	0.479314419	0.375709535	0.583026241	0.48 (0.38 to 0.58)
1360	Cameroon	YLLs (Years of Life Lost)	Both	Prostate cancer	Age-standardized	0.855525229	0.684287936	1.027053752	0.86 (0.68 to 1.03)
1361	Chad	Deaths	Both	Prostate cancer	All ages	0.328773754	0.28733344	0.370231192	0.33 (0.29 to 0.37)
1362	Chad	Deaths	Both	Prostate cancer	Age-standardized	1.947745827	1.853080123	2.042499515	1.95 (1.85 to 2.04)
1363	Chad	DALYs (Disability-Adjusted Life Years)	Both	Prostate cancer	All ages	0.543359329	0.489197382	0.597550468	0.54 (0.49 to 0.6)
1364	Chad	DALYs (Disability-Adjusted Life Years)	Both	Prostate cancer	Age-standardized	1.963387718	1.857196557	2.069689589	1.96 (1.86 to 2.07)
1365	Chad	YLDs (Years Lived with Disability)	Both	Prostate cancer	All ages	1.006203716	0.940452063	1.071998199	1.01 (0.94 to 1.07)
1366	Chad	YLDs (Years Lived with Disability)	Both	Prostate cancer	Age-standardized	2.378494624	2.311250218	2.445783226	2.38 (2.31 to 2.45)
1367	Chad	YLLs (Years of Life Lost)	Both	Prostate cancer	All ages	0.532930667	0.478054888	0.587836415	0.53 (0.48 to 0.59)

1368	Chad	YLLs (Years of Life Lost)	Both	Prostate cancer	Age-standardized	1.954126818	1.846766728	2.06160008	1.95 (1.85 to 2.06)
1369	Gambia	Deaths	Both	Prostate cancer	All ages	-0.638297539	-0.848731155	-0.427417308	-0.64 (-0.85 to -0.43)
1370	Gambia	Deaths	Both	Prostate cancer	Age-standardized	-1.179881605	-1.323748049	-1.035805408	-1.18 (-1.32 to -1.04)
1371	Gambia	DALYs (Disability-Adjusted Life Years)	Both	Prostate cancer	All ages	-0.83252969	-1.055461453	-0.60909564	-0.83 (-1.06 to -0.61)
1372	Gambia	DALYs (Disability-Adjusted Life Years)	Both	Prostate cancer	Age-standardized	-1.26824397	-1.429980352	-1.106242207	-1.27 (-1.43 to -1.11)
1373	Gambia	YLDs (Years Lived with Disability)	Both	Prostate cancer	All ages	0.061997584	-0.128020098	0.252376796	0.06 (-0.13 to 0.25)
1374	Gambia	YLDs (Years Lived with Disability)	Both	Prostate cancer	Age-standardized	-0.400009407	-0.540642143	-0.25917782	-0.4 (-0.54 to -0.26)
1375	Gambia	YLLs (Years of Life Lost)	Both	Prostate cancer	All ages	-0.858573345	-1.082695317	-0.633943569	-0.86 (-1.08 to -0.6

									3)
1376	Gambia	YLLs (Years of Life Lost)	Both	Prostate cancer	Age-standardized	-1.293296441	-1.455842479	-1.130482287	-1.29 (-1.46 to -1.13)
1377	Tunisia	Deaths	Both	Prostate cancer	All ages	1.460257176	1.377404997	1.543177066	1.46 (1.38 to 1.54)
1378	Tunisia	Deaths	Both	Prostate cancer	Age-standardized	-0.93250609	-1.033689497	-0.831219233	-0.93 (-1.03 to -0.83)
1379	Tunisia	DALYs (Disability-Adjusted Life Years)	Both	Prostate cancer	All ages	1.491752824	1.427852135	1.55569377	1.49 (1.43 to 1.56)
1380	Tunisia	DALYs (Disability-Adjusted Life Years)	Both	Prostate cancer	Age-standardized	-0.703316573	-0.758191565	-0.648411238	-0.7 (-0.76 to -0.65)
1381	Tunisia	YLDs (Years Lived with Disability)	Both	Prostate cancer	All ages	3.847315498	3.739150914	3.955592861	3.85 (3.74 to 3.96)
1382	Tunisia	YLDs (Years Lived with Disability)	Both	Prostate cancer	Age-standardized	1.725074842	1.683313693	1.766853142	1.73 (1.68 to 1.77)
138	Tunisia	YLLs (Years of Life Lost)	Both	Prostate canc	All ages	1.259288065	1.198384403	1.320228379	1.26 (1.2 to

3				er					1.32)
1384	Tunisia	YLLs (Years of Life Lost)	Both	Prostate cancer	Age-standardized	-0.928037389	-0.992792588	-0.863239837	-0.93 (-0.99 to -0.86)
1385	Guinea	Deaths	Both	Prostate cancer	All ages	-0.295742613	-0.468730712	-0.122453857	-0.3 (-0.47 to -0.12)
1386	Guinea	Deaths	Both	Prostate cancer	Age-standardized	0.726766122	0.671068055	0.782495004	0.73 (0.67 to 0.78)
1387	Guinea	DALYs (Disability-Adjusted Life Years)	Both	Prostate cancer	All ages	-0.22937774	-0.372739906	-0.085809278	-0.23 (-0.37 to -0.09)
1388	Guinea	DALYs (Disability-Adjusted Life Years)	Both	Prostate cancer	Age-standardized	0.855192735	0.784748786	0.92568592	0.86 (0.78 to 0.93)
1389	Guinea	YLDs (Years Lived with Disability)	Both	Prostate cancer	All ages	0.482197819	0.346966467	0.617611414	0.48 (0.35 to 0.62)
1390	Guinea	YLDs (Years Lived with Disability)	Both	Prostate cancer	Age-standardized	1.558918008	1.490348174	1.62753417	1.56 (1.49 to 1.63)
1391	Guinea	YLLs (Years of Life Lost)	Both	Prostate cancer	All ages	-0.246096659	-0.389884705	-0.102101054	-0.25 (-0.39 to

									-0.1)
1392	Guinea	YLLs (Years of Life Lost)	Both	Prostate cancer	Age-standardized	0.838806547	0.768119728	0.909542951	0.84 (0.77 to 0.91)
1393	C 幺 te d'Ivoire	Deaths	Both	Prostate cancer	All ages	-0.452899975	-0.77735682	-0.127382161	-0.45 (-0.78 to -0.13)
1394	C 幺 te d'Ivoire	Deaths	Both	Prostate cancer	Age-standardized	-1.290104694	-1.598375049	-0.980868596	-1.29 (-1.6 to -0.98)
1395	C 幺 te d'Ivoire	DALYs (Disability-Adjusted Life Years)	Both	Prostate cancer	All ages	-0.502331502	-0.811410238	-0.192289655	-0.5 (-0.81 to -0.19)
1396	C 幺 te d'Ivoire	DALYs (Disability-Adjusted Life Years)	Both	Prostate cancer	Age-standardized	-1.217863894	-1.521860264	-0.912929105	-1.22 (-1.52 to -0.91)
1397	C 幺 te d'Ivoire	YLDs (Years Lived with Disability)	Both	Prostate cancer	All ages	0.58076587	0.312911462	0.849335501	0.58 (0.31 to 0.85)
1398	C 幺 te d'Ivoire	YLDs (Years Lived with Disability)	Both	Prostate cancer	Age-standardized	-0.219814285	-0.476343021	0.03737567	-0.22 (-0.48 to 0.04)

1399	C 么 te d'Ivoire	YLLs (Years of Life Lost)	Both	Prostate cancer	All ages	-0.530674191	-0.841051207	-0.219325666	-0.53 (-0.84 to -0.22)
1400	C 么 te d'Ivoire	YLLs (Years of Life Lost)	Both	Prostate cancer	Age-standardized	-1.243298597	-1.548714696	-0.936935035	-1.24 (-1.55 to -0.94)
1401	Liberia	Deaths	Both	Prostate cancer	All ages	-1.429249252	-1.676107292	-1.181771434	-1.43 (-1.68 to -1.18)
1402	Liberia	Deaths	Both	Prostate cancer	Age-standardized	0.447236035	0.374997602	0.519526458	0.45 (0.37 to 0.52)
1403	Liberia	DALYs (Disability-Adjusted Life Years)	Both	Prostate cancer	All ages	-1.066589724	-1.334923422	-0.797526256	-1.07 (-1.33 to -0.8)
1404	Liberia	DALYs (Disability-Adjusted Life Years)	Both	Prostate cancer	Age-standardized	0.606115751	0.531978242	0.680307932	0.61 (0.53 to 0.68)
1405	Liberia	YLDs (Years Lived with Disability)	Both	Prostate cancer	All ages	0.272791897	0.022520206	0.523689807	0.27 (0.02 to 0.52)
1406	Liberia	YLDs (Years Lived with Disability)	Both	Prostate cancer	Age-standardized	1.896786958	1.789696338	2.003990245	1.9 (1.79 to 2)

1407	Liberia	YLLs (Years of Life Lost)	Both	Prostate cancer	All ages	-1.101357505	-1.369817887	-0.832166405	-1.1 (-1.37 to -0.83)
1408	Liberia	YLLs (Years of Life Lost)	Both	Prostate cancer	Age-standardized	0.573143173	0.499418009	0.646922422	0.57 (0.5 to 0.65)
1409	Ghana	Deaths	Both	Prostate cancer	All ages	1.204516996	0.919965805	1.489870501	1.2 (0.92 to 1.49)
1410	Ghana	Deaths	Both	Prostate cancer	Age-standardized	0.493316727	0.165888987	0.82181478	0.49 (0.17 to 0.82)
1411	Ghana	DALYs (Disability-Adjusted Life Years)	Both	Prostate cancer	All ages	1.194824889	0.917555849	1.472855719	1.19 (0.92 to 1.47)
1412	Ghana	DALYs (Disability-Adjusted Life Years)	Both	Prostate cancer	Age-standardized	0.626686059	0.309346851	0.945029203	0.63 (0.31 to 0.95)
1413	Ghana	YLDs (Years Lived with Disability)	Both	Prostate cancer	All ages	2.248960713	1.970608646	2.528072606	2.25 (1.97 to 2.53)
1414	Ghana	YLDs (Years Lived with Disability)	Both	Prostate cancer	Age-standardized	1.589724833	1.26729963	1.913176607	1.59 (1.27 to 1.91)
1415	Ghana	YLLs (Years of Life Lost)	Both	Prostate cancer	All ages	1.165699793	0.888790152	1.443369468	1.17 (0.89 to 1.44

1416	Ghana	YLLs (Years of Life Lost)	Both	Prostate cancer	Age-standardized	0.601116221	0.284196507	0.91903747	0.6 (0.28 to 0.92)
1417	Guinea-Bissau	Deaths	Both	Prostate cancer	All ages	1.529304153	1.272350731	1.786909531	1.53 (1.27 to 1.79)
1418	Guinea-Bissau	Deaths	Both	Prostate cancer	Age-standardized	1.997713265	1.812023522	2.183741678	2 (1.81 to 2.18)
1419	Guinea-Bissau	DALYs (Disability-Adjusted Life Years)	Both	Prostate cancer	All ages	1.776595582	1.511726668	2.042155605	1.78 (1.51 to 2.04)
1420	Guinea-Bissau	DALYs (Disability-Adjusted Life Years)	Both	Prostate cancer	Age-standardized	2.12096224	1.92067729	2.321640768	2.12 (1.92 to 2.32)
1421	Guinea-Bissau	YLDs (Years Lived with Disability)	Both	Prostate cancer	All ages	2.553780226	2.253432063	2.8550106	2.55 (2.25 to 2.86)
1422	Guinea-Bissau	YLDs (Years Lived with Disability)	Both	Prostate cancer	Age-standardized	2.824330863	2.595340978	3.053831847	2.82 (2.6 to 3.05)
1423	Guinea-Bissau	YLLs (Years of Life Lost)	Both	Prostate cancer	All ages	1.759100859	1.495109471	2.023778896	1.76 (1.5 to 2.02)

1424	Guinea-Bissau	YLLs (Years of Life Lost)	Both	Prostate cancer	Age-standardized	2.105292152	1.90565663	2.305318765	2.11 (1.91 to 2.31)
1425	Mauritania	Deaths	Both	Prostate cancer	All ages	0.994298478	0.830298253	1.158565449	0.99 (0.83 to 1.16)
1426	Mauritania	Deaths	Both	Prostate cancer	Age-standardized	1.026238033	0.925321885	1.127255089	1.03 (0.93 to 1.13)
1427	Mauritania	DALYs (Disability-Adjusted Life Years)	Both	Prostate cancer	All ages	0.913144532	0.72615748	1.100478704	0.91 (0.73 to 1.1)
1428	Mauritania	DALYs (Disability-Adjusted Life Years)	Both	Prostate cancer	Age-standardized	0.878083816	0.768749515	0.987536745	0.88 (0.77 to 0.99)
1429	Mauritania	YLDs (Years Lived with Disability)	Both	Prostate cancer	All ages	2.570642619	2.333725999	2.808107733	2.57 (2.33 to 2.81)
1430	Mauritania	YLDs (Years Lived with Disability)	Both	Prostate cancer	Age-standardized	2.452384738	2.313310988	2.591647529	2.45 (2.31 to 2.59)
1431	Mauritania	YLLs (Years of Life Lost)	Both	Prostate cancer	All ages	0.865375692	0.680573131	1.050517464	0.87 (0.68 to 1.05)
1432	Mauritania	YLLs (Years of Life Lost)	Both	Prostate cancer	Age-standardized	0.8334193	0.725175271	0.941779653	0.83 (0.73 to 0.94)

1433	Mali	Deaths	Both	Prostate cancer	All ages	1.999144213	1.704780818	2.294359582	2 (1.7 to 2.29)
1434	Mali	Deaths	Both	Prostate cancer	Age-standardized	2.618093316	2.405181227	2.831448073	2.62 (2.41 to 2.83)
1435	Mali	DALYs (Disability-Adjusted Life Years)	Both	Prostate cancer	All ages	1.851634123	1.570275927	2.133771705	1.85 (1.57 to 2.13)
1436	Mali	DALYs (Disability-Adjusted Life Years)	Both	Prostate cancer	Age-standardized	2.562262946	2.332414091	2.792628065	2.56 (2.33 to 2.79)
1437	Mali	YLDs (Years Lived with Disability)	Both	Prostate cancer	All ages	2.586578422	2.325646388	2.848175837	2.59 (2.33 to 2.85)
1438	Mali	YLDs (Years Lived with Disability)	Both	Prostate cancer	Age-standardized	3.215394369	2.994250924	3.437012641	3.22 (2.99 to 3.44)
1439	Mali	YLLs (Years of Life Lost)	Both	Prostate cancer	All ages	1.833392139	1.551634635	2.115931387	1.83 (1.55 to 2.12)
1440	Mali	YLLs (Years of Life Lost)	Both	Prostate cancer	Age-standardized	2.546490009	2.316497886	2.776999119	2.55 (2.32 to 2.78)
1441	Nigeria	Deaths	Both	Prostate cancer	All ages	-0.065976048	-0.180349801	0.048528754	-0.07 (-0.18 to

									0.05)
1442	Nigeria	Deaths	Both	Prostate cancer	Age-standardized	1.100726223	0.904640555	1.297192939	1.1 (0.9 to 1.3)
1443	Nigeria	DALYs (Disability-Adjusted Life Years)	Both	Prostate cancer	All ages	-0.150738446	-0.283100429	-0.018200769	-0.15 (-0.28 to -0.02)
1444	Nigeria	DALYs (Disability-Adjusted Life Years)	Both	Prostate cancer	Age-standardized	0.942117125	0.697291024	1.187538473	0.94 (0.7 to 1.19)
1445	Nigeria	YLDs (Years Lived with Disability)	Both	Prostate cancer	All ages	1.112036783	0.987324315	1.236903262	1.11 (0.99 to 1.24)
1446	Nigeria	YLDs (Years Lived with Disability)	Both	Prostate cancer	Age-standardized	2.132716925	1.883716078	2.382326323	2.13 (1.88 to 2.38)
1447	Nigeria	YLLs (Years of Life Lost)	Both	Prostate cancer	All ages	-0.183359403	-0.316678178	-0.049862325	-0.18 (-0.32 to -0.05)
1448	Nigeria	YLLs (Years of Life Lost)	Both	Prostate cancer	Age-standardized	0.911883374	0.666597427	1.157766989	0.91 (0.67 to 1.16)
1449	Niger	Deaths	Both	Prostate cancer	All ages	1.667118261	1.573967609	1.76035434	1.67 (1.57 to 1.76)

1450	Niger	Deaths	Both	Prostate cancer	Age-standardized	1.923034745	1.778822773	2.067451052	1.92 (1.78 to 2.07)
1451	Niger	DALYs (Disability-Adjusted Life Years)	Both	Prostate cancer	All ages	1.492138452	1.38513202	1.599257822	1.49 (1.39 to 1.6)
1452	Niger	DALYs (Disability-Adjusted Life Years)	Both	Prostate cancer	Age-standardized	1.684091783	1.577763008	1.79053186	1.68 (1.58 to 1.79)
1453	Niger	YLDs (Years Lived with Disability)	Both	Prostate cancer	All ages	2.275463408	2.125433259	2.425713962	2.28 (2.13 to 2.43)
1454	Niger	YLDs (Years Lived with Disability)	Both	Prostate cancer	Age-standardized	2.287383014	2.149319613	2.425633019	2.29 (2.15 to 2.43)
1455	Niger	YLLs (Years of Life Lost)	Both	Prostate cancer	All ages	1.474286467	1.36796743	1.580717016	1.47 (1.37 to 1.58)
1456	Niger	YLLs (Years of Life Lost)	Both	Prostate cancer	Age-standardized	1.67069871	1.564799623	1.776708217	1.67 (1.56 to 1.78)
1457	Senegal	Deaths	Both	Prostate cancer	All ages	1.886240961	1.666547693	2.106408968	1.89 (1.67 to 2.11)
1458	Senegal	Deaths	Both	Prostate cancer	Age-standardized	1.459728074	1.265150421	1.654679602	1.46 (1.27 to 1.65)

1459	Senegal	DALYs (Disability-Adjusted Life Years)	Both	Prostate cancer	All ages	1.816063001	1.594560119	2.038048818	1.82 (1.59 to 2.04)
1460	Senegal	DALYs (Disability-Adjusted Life Years)	Both	Prostate cancer	Age-standardized	1.353584145	1.16167187	1.545860495	1.35 (1.16 to 1.55)
1461	Senegal	YLDs (Years Lived with Disability)	Both	Prostate cancer	All ages	2.699756787	2.513306037	2.886546653	2.7 (2.51 to 2.89)
1462	Senegal	YLDs (Years Lived with Disability)	Both	Prostate cancer	Age-standardized	2.189060839	2.033339931	2.345019405	2.19 (2.03 to 2.35)
1463	Senegal	YLLs (Years of Life Lost)	Both	Prostate cancer	All ages	1.792174329	1.569943845	2.014891044	1.79 (1.57 to 2.01)
1464	Senegal	YLLs (Years of Life Lost)	Both	Prostate cancer	Age-standardized	1.331220249	1.138499159	1.524308571	1.33 (1.14 to 1.52)
1465	Sao Tome and Principe	Deaths	Both	Prostate cancer	All ages	-0.332607194	-0.532279615	-0.132533949	-0.33 (-0.53 to -0.13)
1466	Sao Tome and Principe	Deaths	Both	Prostate cancer	Age-standardized	0.384609995	0.151815319	0.617945782	0.38 (0.15 to 0.62)

1467	Sao Tome and Principe	DALYs (Disability-Adjusted Life Years)	Both	Prostate cancer	All ages	-0.21340527	-0.416543861	-0.009852301	-0.21 (-0.42 to -0.01)
1468	Sao Tome and Principe	DALYs (Disability-Adjusted Life Years)	Both	Prostate cancer	Age-standardized	0.294495732	0.090755349	0.498650841	0.29 (0.09 to 0.5)
1469	Sao Tome and Principe	YLDs (Years Lived with Disability)	Both	Prostate cancer	All ages	1.274360383	1.021242048	1.52811293	1.27 (1.02 to 1.53)
1470	Sao Tome and Principe	YLDs (Years Lived with Disability)	Both	Prostate cancer	Age-standardized	1.724256572	1.554068294	1.894730058	1.72 (1.55 to 1.89)
1471	Sao Tome and Principe	YLLs (Years of Life Lost)	Both	Prostate cancer	All ages	-0.259477327	-0.461026371	-0.057520181	-0.26 (-0.46 to -0.06)
1472	Sao Tome and Principe	YLLs (Years of Life Lost)	Both	Prostate cancer	Age-standardized	0.251042676	0.045463988	0.457043798	0.25 (0.05 to 0.46)
1473	Togo	Deaths	Both	Prostate cancer	All ages	1.463558859	1.320585906	1.60673356	1.46 (1.32 to 1.61)
1474	Togo	Deaths	Both	Prostate cancer	Age-standardized	0.395945222	0.325006103	0.466934502	0.4 (0.33 to 0.47)
147	Togo	DALYs (Disability-Adjusted	Both	Prostate canc	All ages	1.753820963	1.616557087	1.891270256	1.75 (1.62 to

5		Life Years)		er					1.89)
1476	Togo	DALYs (Disability-Adjusted Life Years)	Both	Prostate cancer	Age-standardized	0.600977314	0.528074104	0.673933393	0.6 (0.53 to 0.67)
1477	Togo	YLDs (Years Lived with Disability)	Both	Prostate cancer	All ages	2.729136127	2.52438767	2.934293481	2.73 (2.52 to 2.93)
1478	Togo	YLDs (Years Lived with Disability)	Both	Prostate cancer	Age-standardized	1.466157522	1.348415463	1.584036369	1.47 (1.35 to 1.58)
1479	Togo	YLLs (Years of Life Lost)	Both	Prostate cancer	All ages	1.727508975	1.592206497	1.862991652	1.73 (1.59 to 1.86)
1480	Togo	YLLs (Years of Life Lost)	Both	Prostate cancer	Age-standardized	0.578155574	0.505673663	0.650689758	0.58 (0.51 to 0.65)
1481	Sierra Leone	Deaths	Both	Prostate cancer	All ages	-0.510307954	-0.642067073	-0.378374108	-0.51 (-0.64 to -0.38)
1482	Sierra Leone	Deaths	Both	Prostate cancer	Age-standardized	0.532244823	0.436670429	0.627910164	0.53 (0.44 to 0.63)
1483	Sierra Leone	DALYs (Disability-Adjusted Life Years)	Both	Prostate cancer	All ages	-0.160404455	-0.306783245	-0.013810739	-0.16 (-0.31 to -0.0

1)

1484	Sierra Leone	DALYs (Disability-Adjusted Life Years)	Both	Prostate cancer	Age-standardized	0.702069039	0.587314756	0.816954238	0.7 (0.59 to 0.82)
1485	Sierra Leone	YLDs (Years Lived with Disability)	Both	Prostate cancer	All ages	0.620118588	0.419856519	0.820780029	0.62 (0.42 to 0.82)
1486	Sierra Leone	YLDs (Years Lived with Disability)	Both	Prostate cancer	Age-standardized	1.443439503	1.321896604	1.5651282	1.44 (1.32 to 1.57)
1487	Sierra Leone	YLLs (Years of Life Lost)	Both	Prostate cancer	All ages	-0.180000685	-0.324836318	-0.034954595	-0.18 (-0.32 to -0.03)
1488	Sierra Leone	YLLs (Years of Life Lost)	Both	Prostate cancer	Age-standardized	0.68363166	0.568818421	0.798575975	0.68 (0.57 to 0.8)
1489	Bermuda	Deaths	Both	Prostate cancer	All ages	2.34740091	2.141935647	2.55327948	2.35 (2.14 to 2.55)
1490	Bermuda	Deaths	Both	Prostate cancer	Age-standardized	-0.503039445	-0.632606072	-0.373303874	-0.5 (-0.63 to -0.37)
1491	Bermuda	DALYs (Disability-Adjusted Life Years)	Both	Prostate cancer	All ages	2.163054058	1.985012776	2.341406156	2.16 (1.99 to 2.34)

1492	Bermuda	DALYs (Disability-Adjusted Life Years)	Both	Prostate cancer	Age-standardized	-0.416593217	-0.55172588	-0.281276933	-0.42 (-0.55 to -0.28)
1493	Bermuda	YLDs (Years Lived with Disability)	Both	Prostate cancer	All ages	4.2342243 4	4.055637188	4.413117995	4.23 (4.06 to 4.41)
1494	Bermuda	YLDs (Years Lived with Disability)	Both	Prostate cancer	Age-standardized	1.716532503	1.448399155	1.985374542	1.72 (1.45 to 1.99)
1495	Bermuda	YLLs (Years of Life Lost)	Both	Prostate cancer	All ages	1.846089327	1.653439935	2.039103819	1.85 (1.65 to 2.04)
1496	Bermuda	YLLs (Years of Life Lost)	Both	Prostate cancer	Age-standardized	-0.743910272	-0.877245929	-0.610395257	-0.74 (-0.88 to -0.61)
1497	American Samoa	Deaths	Both	Prostate cancer	All ages	3.141718876	2.880656405	3.4034438	3.14 (2.88 to 3.4)
1498	American Samoa	Deaths	Both	Prostate cancer	Age-standardized	0.364568393	0.185139801	0.544318337	0.36 (0.19 to 0.54)
1499	American Samoa	DALYs (Disability-Adjusted Life Years)	Both	Prostate cancer	All ages	2.994505596	2.725291272	3.264425455	2.99 (2.73 to 3.26)
150	American Samoa	DALYs (Disability-Adjusted	Both	Prostate canc	Age-standardized	0.384141639	0.201777683	0.566837491	0.38 (0.2 to

0		Life Years)		er					0.57)
1501	American Samoa	YLDs (Years Lived with Disability)	Both	Prostate cancer	All ages	3.610849197	3.425091627	3.796940399	3.61 (3.43 to 3.8)
1502	American Samoa	YLDs (Years Lived with Disability)	Both	Prostate cancer	Age-standardized	1.005933603	0.843458475	1.168670505	1.01 (0.84 to 1.17)
1503	American Samoa	YLLs (Years of Life Lost)	Both	Prostate cancer	All ages	2.968278893	2.695126716	3.242157609	2.97 (2.7 to 3.24)
1504	American Samoa	YLLs (Years of Life Lost)	Both	Prostate cancer	Age-standardized	0.358517426	0.173647212	0.543728818	0.36 (0.17 to 0.54)
1505	Greenland	Deaths	Both	Prostate cancer	All ages	1.148234903	0.990731743	1.305983701	1.15 (0.99 to 1.31)
1506	Greenland	Deaths	Both	Prostate cancer	Age-standardized	-1.500585807	-1.61503331	-1.386005172	-1.5 (-1.62 to -1.39)
1507	Greenland	DALYs (Disability-Adjusted Life Years)	Both	Prostate cancer	All ages	1.029782063	0.905755749	1.153960822	1.03 (0.91 to 1.15)
1508	Greenland	DALYs (Disability-Adjusted Life Years)	Both	Prostate cancer	Age-standardized	-1.569368229	-1.663705266	-1.474940692	-1.57 (-1.66 to -1.47)

1509	Greenland	YLDs (Years Lived with Disability)	Both	Prostate cancer	All ages	3.0160266 5	2.9129780 78	3.1191784 05	3.02 (2.91 to 3.12)
1510	Greenland	YLDs (Years Lived with Disability)	Both	Prostate cancer	Age-standardized	0.2544103 48	0.1603594 4	0.3485495 7	0.25 (0.16 to 0.35)
1511	Greenland	YLLs (Years of Life Lost)	Both	Prostate cancer	All ages	0.9059160 9	0.7822003 9	1.0297836 57	0.91 (0.78 to 1.03)
1512	Greenland	YLLs (Years of Life Lost)	Both	Prostate cancer	Age-standardized	-1.676096 873	-1.770159 281	-1.581944 393	-1.68 (-1.77 to -1.58)
1513	Cook Islands	Deaths	Both	Prostate cancer	All ages	2.3036204 46	2.2132114 99	2.3941093 62	2.3 (2.21 to 2.39)
1514	Cook Islands	Deaths	Both	Prostate cancer	Age-standardized	-0.950328 805	-1.046155 413	-0.854409 399	-0.95 (-1.05 to -0.85)
1515	Cook Islands	DALYs (Disability-Adjusted Life Years)	Both	Prostate cancer	All ages	2.0635052 66	1.9566692 66	2.1704532 14	2.06 (1.96 to 2.17)
1516	Cook Islands	DALYs (Disability-Adjusted Life Years)	Both	Prostate cancer	Age-standardized	-0.892183 414	-0.985564 607	-0.798714 153	-0.89 (-0.99 to -0.8)

1517	Cook Islands	YLDs (Years Lived with Disability)	Both	Prostate cancer	All ages	4.059900337	3.864539368	4.255628765	4.06 (3.86 to 4.26)
1518	Cook Islands	YLDs (Years Lived with Disability)	Both	Prostate cancer	Age-standardized	1.127717496	0.928831133	1.326995776	1.13 (0.93 to 1.33)
1519	Cook Islands	YLLs (Years of Life Lost)	Both	Prostate cancer	All ages	1.932878682	1.826400176	2.039468531	1.93 (1.83 to 2.04)
1520	Cook Islands	YLLs (Years of Life Lost)	Both	Prostate cancer	Age-standardized	-1.02124024	-1.112370177	-0.930026323	-1.02 (-1.11 to -0.93)
1521	Guam	Deaths	Both	Prostate cancer	All ages	3.22351118	2.96425672 3	3.48341841 6	3.22 (2.96 to 3.48)
1522	Guam	Deaths	Both	Prostate cancer	Age-standardized	-0.168241507	-0.401023947	0.065084991	-0.17 (-0.4 to 0.07)
1523	Guam	DALYs (Disability-Adjusted Life Years)	Both	Prostate cancer	All ages	3.027560615	2.782193527	3.273513457	3.03 (2.78 to 3.27)
1524	Guam	DALYs (Disability-Adjusted Life Years)	Both	Prostate cancer	Age-standardized	0.061525108	-0.15221246	0.27572021	0.06 (-0.15 to 0.28)

1525	Guam	YLDs (Years Lived with Disability)	Both	Prostate cancer	All ages	3.901848702	3.637879287	4.166490456	3.9 (3.64 to 4.17)
1526	Guam	YLDs (Years Lived with Disability)	Both	Prostate cancer	Age-standardized	0.997890046	0.67805221	1.318743955	1 (0.68 to 1.32)
1527	Guam	YLLs (Years of Life Lost)	Both	Prostate cancer	All ages	2.971342032	2.713470859	3.229860613	2.97 (2.71 to 3.23)
1528	Guam	YLLs (Years of Life Lost)	Both	Prostate cancer	Age-standardized	0.002213475	-0.218567853	0.223483315	0 (-0.22 to 0.22)
1529	Monaco	Deaths	Both	Prostate cancer	All ages	-0.374512216	-0.449263864	-0.299704438	-0.37 (-0.45 to -0.3)
1530	Monaco	Deaths	Both	Prostate cancer	Age-standardized	-0.685264416	-0.737498896	-0.633002449	-0.69 (-0.74 to -0.63)
1531	Monaco	DALYs (Disability-Adjusted Life Years)	Both	Prostate cancer	All ages	-0.370593553	-0.438823687	-0.302316661	-0.37 (-0.44 to -0.3)
1532	Monaco	DALYs (Disability-Adjusted Life Years)	Both	Prostate cancer	Age-standardized	-0.640662289	-0.697679131	-0.583612709	-0.64 (-0.7 to -0.58)

1533	Monaco	YLDs (Years Lived with Disability)	Both	Prostate cancer	All ages	1.023287198	0.902478561	1.144240477	1.02 (0.9 to 1.14)
1534	Monaco	YLDs (Years Lived with Disability)	Both	Prostate cancer	Age-standardized	0.851058271	0.676379564	1.026040056	0.85 (0.68 to 1.03)
1535	Monaco	YLLs (Years of Life Lost)	Both	Prostate cancer	All ages	-0.583193957	-0.655139897	-0.511195913	-0.58 (-0.66 to -0.51)
1536	Monaco	YLLs (Years of Life Lost)	Both	Prostate cancer	Age-standardized	-0.886351806	-0.936789004	-0.835888929	-0.89 (-0.94 to -0.84)
1537	Nauru	Deaths	Both	Prostate cancer	All ages	-0.120962241	-0.174635555	-0.067260069	-0.12 (-0.17 to -0.07)
1538	Nauru	Deaths	Both	Prostate cancer	Age-standardized	-0.79180181	-0.820474576	-0.763120755	-0.79 (-0.82 to -0.76)
1539	Nauru	DALYs (Disability-Adjusted Life Years)	Both	Prostate cancer	All ages	-0.057043197	-0.104710242	-0.009353406	-0.06 (-0.1 to -0.01)
1540	Nauru	DALYs (Disability-Adjusted Life Years)	Both	Prostate cancer	Age-standardized	-0.646597161	-0.687610986	-0.605566398	-0.65 (-0.69 to

									-0.61)
1541	Nauru	YLDs (Years Lived with Disability)	Both	Prostate cancer	All ages	0.718725127	0.479529351	0.958490318	0.72 (0.48 to 0.96)
1542	Nauru	YLDs (Years Lived with Disability)	Both	Prostate cancer	Age-standardized	0.110446805	-0.039017466	0.260134559	0.11 (-0.04 to 0.26)
1543	Nauru	YLLs (Years of Life Lost)	Both	Prostate cancer	All ages	-0.082122453	-0.125467071	-0.038759023	-0.08 (-0.13 to -0.04)
1544	Nauru	YLLs (Years of Life Lost)	Both	Prostate cancer	Age-standardized	-0.670050912	-0.715316691	-0.624764495	-0.67 (-0.72 to -0.62)
1545	Niue	Deaths	Both	Prostate cancer	All ages	1.472159721	1.138023181	1.807400171	1.47 (1.14 to 1.81)
1546	Niue	Deaths	Both	Prostate cancer	Age-standardized	0.54037335	0.431950946	0.648912801	0.54 (0.43 to 0.65)
1547	Niue	DALYs (Disability-Adjusted Life Years)	Both	Prostate cancer	All ages	1.456055865	1.134646091	1.778487092	1.46 (1.13 to 1.78)
154	Niue	DALYs (Disability-Adjusted	Both	Prostate canc	Age-standardized	0.381669068	0.296644882	0.466765331	0.38 (0.3 to

8		Life Years)		er					0.47)
1549	Niue	YLDs (Years Lived with Disability)	Both	Prostate cancer	All ages	2.720454038	2.345683559	3.096596855	2.72 (2.35 to 3.1)
1550	Niue	YLDs (Years Lived with Disability)	Both	Prostate cancer	Age-standardized	1.589799792	1.469732085	1.710009573	1.59 (1.47 to 1.71)
1551	Niue	YLLs (Years of Life Lost)	Both	Prostate cancer	All ages	1.400092891	1.079965073	1.721234578	1.4 (1.08 to 1.72)
1552	Niue	YLLs (Years of Life Lost)	Both	Prostate cancer	Age-standardized	0.328235659	0.243758961	0.412783547	0.33 (0.24 to 0.41)
1553	Northern Mariana Islands	Deaths	Both	Prostate cancer	All ages	5.331534049	4.729423005	5.937106754	5.33 (4.73 to 5.94)
1554	Northern Mariana Islands	Deaths	Both	Prostate cancer	Age-standardized	0.478254904	0.314660662	0.642115937	0.48 (0.31 to 0.64)
1555	Northern Mariana Islands	DALYs (Disability-Adjusted Life Years)	Both	Prostate cancer	All ages	5.264575179	4.644790625	5.88803056	5.26 (4.64 to 5.89)
1556	Northern Mariana Islands	DALYs (Disability-Adjusted Life Years)	Both	Prostate cancer	Age-standardized	0.516102489	0.369920378	0.662497505	0.52 (0.37 to 0.66)

1557	Northern Mariana Islands	YLDs (Years Lived with Disability)	Both	Prostate cancer	All ages	5.941893461	5.47396609	6.411896756	5.94 (5.47 to 6.41)
1558	Northern Mariana Islands	YLDs (Years Lived with Disability)	Both	Prostate cancer	Age-standardized	1.110531638	0.87723675	1.344366059	1.11 (0.88 to 1.34)
1559	Northern Mariana Islands	YLLs (Years of Life Lost)	Both	Prostate cancer	All ages	5.222721794	4.593601149	5.85562654	5.22 (4.59 to 5.86)
1560	Northern Mariana Islands	YLLs (Years of Life Lost)	Both	Prostate cancer	Age-standardized	0.481133173	0.334303369	0.628177847	0.48 (0.33 to 0.63)
1561	Puerto Rico	Deaths	Both	Prostate cancer	All ages	0.46926291	0.305891438	0.63290047	0.47 (0.31 to 0.63)
1562	Puerto Rico	Deaths	Both	Prostate cancer	Age-standardized	-2.40630481	-2.587418762	-2.224854121	-2.41 (-2.59 to -2.22)
1563	Puerto Rico	DALYs (Disability-Adjusted Life Years)	Both	Prostate cancer	All ages	0.627388355	0.452565301	0.802515662	0.63 (0.45 to 0.8)
1564	Puerto Rico	DALYs (Disability-Adjusted Life Years)	Both	Prostate cancer	Age-standardized	-1.924170368	-2.104970836	-1.743035984	-1.92 (-2.1 to -1.74)
156	Puerto Rico	YLDs (Years Lived with	Both	Prostate canc	All ages	2.503286356	2.281362435	2.725691793	2.5 (2.28 to

5		Disability)		er					2.73)
1566	Puerto Rico	YLDs (Years Lived with Disability)	Both	Prostate cancer	Age-standardized	0.063604187	-0.183901874	0.311723968	0.06 (-0.18 to 0.31)
1567	Puerto Rico	YLLs (Years of Life Lost)	Both	Prostate cancer	All ages	0.401701523	0.230526098	0.573169285	0.4 (0.23 to 0.57)
1568	Puerto Rico	YLLs (Years of Life Lost)	Both	Prostate cancer	Age-standardized	-2.167448291	-2.344456508	-1.990119234	-2.17 (-2.34 to -1.99)
1569	Palau	Deaths	Both	Prostate cancer	All ages	1.300719358	0.962672984	1.63989759	1.3 (0.96 to 1.64)
1570	Palau	Deaths	Both	Prostate cancer	Age-standardized	-0.608345084	-0.662808889	-0.553851419	-0.61 (-0.66 to -0.55)
1571	Palau	DALYs (Disability-Adjusted Life Years)	Both	Prostate cancer	All ages	1.623723334	1.253964974	1.994831974	1.62 (1.25 to 1.99)
1572	Palau	DALYs (Disability-Adjusted Life Years)	Both	Prostate cancer	Age-standardized	-0.619888215	-0.687314407	-0.552416245	-0.62 (-0.69 to -0.55)
157	Palau	YLDs (Years Lived with	Both	Prostate canc	All ages	2.633734218	2.289413851	2.979213616	2.63 (2.29 to

3		Disability)		er					2.98)
1574	Palau	YLDs (Years Lived with Disability)	Both	Prostate cancer	Age-standardized	0.2627734 62	0.1553016 33	0.3703606 13	0.26 (0.16 to 0.37)
1575	Palau	YLLs (Years of Life Lost)	Both	Prostate cancer	All ages	1.5763626 23	1.2059935 45	1.9480870 87	1.58 (1.21 to 1.95)
1576	Palau	YLLs (Years of Life Lost)	Both	Prostate cancer	Age-standardized	-0.659474 196	-0.727297 316	-0.591604 738	-0.66 (-0.73 to -0.59)
1577	San Marino	Deaths	Both	Prostate cancer	All ages	0.0330754 45	-0.438338 291	0.5067212 74	0.03 (-0.44 to 0.51)
1578	San Marino	Deaths	Both	Prostate cancer	Age-standardized	-1.691449 699	-2.092381 898	-1.288875 68	-1.69 (-2.09 to -1.29)
1579	San Marino	DALYs (Disability-Adjusted Life Years)	Both	Prostate cancer	All ages	-0.334423 138	-0.710284 563	0.0428611 12	-0.33 (-0.71 to 0.04)
1580	San Marino	DALYs (Disability-Adjusted Life Years)	Both	Prostate cancer	Age-standardized	-1.671796 079	-2.019486 043	-1.322872 315	-1.67 (-2.02 to -1.32)

1581	San Marino	YLDs (Years Lived with Disability)	Both	Prostate cancer	All ages	0.565327439	0.194641406	0.937384884	0.57 (0.19 to 0.94)
1582	San Marino	YLDs (Years Lived with Disability)	Both	Prostate cancer	Age-standardized	-0.442349138	-0.847130186	-0.035915615	-0.44 (-0.85 to -0.04)
1583	San Marino	YLLs (Years of Life Lost)	Both	Prostate cancer	All ages	-0.469559669	-0.852598711	-0.085040822	-0.47 (-0.85 to -0.09)
1584	San Marino	YLLs (Years of Life Lost)	Both	Prostate cancer	Age-standardized	-1.872879887	-2.220567886	-1.523955565	-1.87 (-2.22 to -1.52)
1585	Saint Kitts and Nevis	Deaths	Both	Prostate cancer	All ages	-0.865223628	-1.081353614	-0.648621413	-0.87 (-1.08 to -0.65)
1586	Saint Kitts and Nevis	Deaths	Both	Prostate cancer	Age-standardized	-0.294595599	-0.499445218	-0.08932424	-0.29 (-0.5 to -0.09)
1587	Saint Kitts and Nevis	DALYs (Disability-Adjusted Life Years)	Both	Prostate cancer	All ages	-0.307035752	-0.578289494	-0.035041945	-0.31 (-0.58 to -0.04)

1588	Saint Kitts and Nevis	DALYs (Disability-Adjusted Life Years)	Both	Prostate cancer	Age-standardized	-0.23264943	-0.447265535	-0.017570655	-0.23 (-0.45 to -0.02)
1589	Saint Kitts and Nevis	YLDs (Years Lived with Disability)	Both	Prostate cancer	All ages	1.574072251	1.335333112	1.813373842	1.57 (1.34 to 1.81)
1590	Saint Kitts and Nevis	YLDs (Years Lived with Disability)	Both	Prostate cancer	Age-standardized	1.515545073	1.145439232	1.887005184	1.52 (1.15 to 1.89)
1591	Saint Kitts and Nevis	YLLs (Years of Life Lost)	Both	Prostate cancer	All ages	-0.417487957	-0.690259598	-0.1439671	-0.42 (-0.69 to -0.14)
1592	Saint Kitts and Nevis	YLLs (Years of Life Lost)	Both	Prostate cancer	Age-standardized	-0.33245303	-0.54159333	-0.122872982	-0.33 (-0.54 to -0.12)
1593	Tuvalu	Deaths	Both	Prostate cancer	All ages	0.972924232	0.81778241	1.128304791	0.97 (0.82 to 1.13)
1594	Tuvalu	Deaths	Both	Prostate cancer	Age-standardized	0.618645843	0.515761961	0.721635033	0.62 (0.52 to 0.72)
1595	Tuvalu	DALYs (Disability-Adjusted Life Years)	Both	Prostate cancer	All ages	0.812936955	0.718903167	0.907058536	0.81 (0.72 to 0.91)

1596	Tuvalu	DALYs (Disability-Adjusted Life Years)	Both	Prostate cancer	Age-standardized	0.493328142	0.417155363	0.569558704	0.49 (0.42 to 0.57)
1597	Tuvalu	YLDs (Years Lived with Disability)	Both	Prostate cancer	All ages	1.991783771	1.85112967	2.132632113	1.99 (1.85 to 2.13)
1598	Tuvalu	YLDs (Years Lived with Disability)	Both	Prostate cancer	Age-standardized	1.643416131	1.50892465	1.778085803	1.64 (1.51 to 1.78)
1599	Tuvalu	YLLs (Years of Life Lost)	Both	Prostate cancer	All ages	0.775574272	0.682564932	0.868669532	0.78 (0.68 to 0.87)
1600	Tuvalu	YLLs (Years of Life Lost)	Both	Prostate cancer	Age-standardized	0.457906343	0.382820634	0.533048216	0.46 (0.38 to 0.53)
1601	Tokelau	Deaths	Both	Prostate cancer	All ages	0.74243008	0.436554057	1.049237639	0.74 (0.44 to 1.05)
1602	Tokelau	Deaths	Both	Prostate cancer	Age-standardized	-0.338555035	-0.425133903	-0.251900888	-0.34 (-0.43 to -0.25)
1603	Tokelau	DALYs (Disability-Adjusted Life Years)	Both	Prostate cancer	All ages	0.460218152	0.23215894	0.68879627	0.46 (0.23 to 0.69)

1604	Tokelau	DALYs (Disability-Adjusted Life Years)	Both	Prostate cancer	Age-standardized	-0.345762756	-0.412783675	-0.278696733	-0.35 (-0.41 to -0.28)
1605	Tokelau	YLDs (Years Lived with Disability)	Both	Prostate cancer	All ages	1.887696317	1.676587713	2.099243241	1.89 (1.68 to 2.1)
1606	Tokelau	YLDs (Years Lived with Disability)	Both	Prostate cancer	Age-standardized	1.16939451	1.076681119	1.262192943	1.17 (1.08 to 1.26)
1607	Tokelau	YLLs (Years of Life Lost)	Both	Prostate cancer	All ages	0.407360101	0.177418134	0.637829865	0.41 (0.18 to 0.64)
1608	Tokelau	YLLs (Years of Life Lost)	Both	Prostate cancer	Age-standardized	-0.401274295	-0.468126788	-0.334376899	-0.4 (-0.47 to -0.33)
1609	South Sudan	Deaths	Both	Prostate cancer	All ages	-2.055878852	-2.551806129	-1.557427733	-2.06 (-2.55 to -1.56)
1610	South Sudan	Deaths	Both	Prostate cancer	Age-standardized	-0.932517869	-1.067692843	-0.797158201	-0.93 (-1.07 to -0.8)
1611	South Sudan	DALYs (Disability-Adjusted Life Years)	Both	Prostate cancer	All ages	-1.908159094	-2.470863608	-1.342207998	-1.91 (-2.47 to -1.34)

1612	South Sudan	DALYs (Disability-Adjusted Life Years)	Both	Prostate cancer	Age-standardized	-1.090485302	-1.266087536	-0.914570752	-1.09 (-1.27 to -0.91)
1613	South Sudan	YLDs (Years Lived with Disability)	Both	Prostate cancer	All ages	-1.138291315	-1.717394892	-0.555775527	-1.14 (-1.72 to -0.56)
1614	South Sudan	YLDs (Years Lived with Disability)	Both	Prostate cancer	Age-standardized	-0.387410293	-0.569123085	-0.205365416	-0.39 (-0.57 to -0.21)
1615	South Sudan	YLLs (Years of Life Lost)	Both	Prostate cancer	All ages	-1.927152725	-2.489247091	-1.361818204	-1.93 (-2.49 to -1.36)
1616	South Sudan	YLLs (Years of Life Lost)	Both	Prostate cancer	Age-standardized	-1.107601749	-1.282919927	-0.931972212	-1.11 (-1.28 to -0.93)
1617	United States Virgin Islands	Deaths	Both	Prostate cancer	All ages	2.002207328	1.622643146	2.383189197	2 (1.62 to 2.38)
1618	United States Virgin Islands	Deaths	Both	Prostate cancer	Age-standardized	-1.853622299	-2.232544035	-1.47323196	-1.85 (-2.23 to -1.47)
161	United States Virgin Islands	DALYs (Disability-Adjusted	Both	Prostate canc	All ages	1.628285322	1.248690553	2.009303241	1.63 (1.25 to

9		Life Years)		er					2.01)
1620	United States Virgin Islands	DALYs (Disability-Adjusted Life Years)	Both	Prostate cancer	Age-standardized	-2.015818504	-2.4037983	-1.626296349	-2.02 (-2.4 to -1.63)
1621	United States Virgin Islands	YLDs (Years Lived with Disability)	Both	Prostate cancer	All ages	2.563302293	2.203750153	2.924119335	2.56 (2.2 to 2.92)
1622	United States Virgin Islands	YLDs (Years Lived with Disability)	Both	Prostate cancer	Age-standardized	-1.119560428	-1.50074554	-0.736900157	-1.12 (-1.5 to -0.74)
1623	United States Virgin Islands	YLLs (Years of Life Lost)	Both	Prostate cancer	All ages	1.549242568	1.166796489	1.933134427	1.55 (1.17 to 1.93)
1624	United States Virgin Islands	YLLs (Years of Life Lost)	Both	Prostate cancer	Age-standardized	-2.089185979	-2.478753708	-1.698062046	-2.09 (-2.48 to -1.7)
1625	Sudan	Deaths	Both	Prostate cancer	All ages	0.186489979	0.152744575	0.220246754	0.19 (0.15 to 0.22)
1626	Sudan	Deaths	Both	Prostate cancer	Age-standardized	0.653126155	0.603144402	0.70313274	0.65 (0.6 to 0.7)
1627	Sudan	DALYs (Disability-Adjusted Life Years)	Both	Prostate cancer	All ages	0.12268375	0.03921695	0.20622024	0.12 (0.04 to 0.21)

1628	Sudan	DALYs (Disability-Adjusted Life Years)	Both	Prostate cancer	Age-standardized	0.533371317	0.490318495	0.576442584	0.53 (0.49 to 0.58)
1629	Sudan	YLDs (Years Lived with Disability)	Both	Prostate cancer	All ages	1.950908864	1.74822719	2.153994278	1.95 (1.75 to 2.15)
1630	Sudan	YLDs (Years Lived with Disability)	Both	Prostate cancer	Age-standardized	2.269988437	2.162432702	2.377657405	2.27 (2.16 to 2.38)
1631	Sudan	YLLs (Years of Life Lost)	Both	Prostate cancer	All ages	0.03058849	-0.045262009	0.106496548	0.03 (-0.05 to 0.11)
1632	Sudan	YLLs (Years of Life Lost)	Both	Prostate cancer	Age-standardized	0.45012755	0.406725878	0.493547982	0.45 (0.41 to 0.49)

6

Table S2. The EAPC of smoking-related prostate cancer-related ASRs of deaths,

EAPC: estimated annual percentage change, ASR: age-standardized rate, YLDs: Years Lived with Disability, YLLs: Years of Life Lost, DALYs: disability-adjusted-life-years.

YLDs, YLLs and DALYs for different age groups between 1990 and 2021.

measure	location	sex	age	cause	rei	metric	year	val	upper	lower
Deaths	Global	Both	30-34 years	Prostate cancer	Smoking	Number	1990	7.838197803	12.71003048	2.816558162
Deaths	Global	Both	30-34 years	Prostate cancer	Smoking	Rate	1990	0.002033664	0.003297688	0.000730772
Deaths	Global	Both	30-34 years	Prostate cancer	Smoking	Number	1991	7.835652076	12.83876055	2.837527912
Deaths	Global	Both	30-34 years	Prostate cancer	Smoking	Rate	1991	0.002006254	0.003287258	0.000726526
Deaths	Global	Both	30-34 years	Prostate cancer	Smoking	Number	1992	7.810091431	12.793119	2.887993165
Deaths	Global	Both	30-34 years	Prostate cancer	Smoking	Rate	1992	0.001957833	0.003206978	0.000723962
Deaths	Global	Both	30-34 years	Prostate cancer	Smoking	Number	1993	7.977479596	13.0055904	2.936188618
Deaths	Global	Both	30-34 years	Prostate cancer	Smoking	Rate	1993	0.001939396	0.003161775	0.000713814
Deaths	Global	Both	30-34 years	Prostate cancer	Smoking	Number	1994	8.149787407	13.43965218	2.95840741
Deaths	Global	Both	30-34 years	Prostate cancer	Smoking	Rate	1994	0.001914658	0.003157425	0.000695029

Deaths	Global	Both	30-34 years	Prostate cancer	Smoking	Number	1995	8.151355214	13.35425364	2.873233909
Deaths	Global	Both	30-34 years	Prostate cancer	Smoking	Rate	1995	0.001853647	0.003036805	0.000653384
Deaths	Global	Both	30-34 years	Prostate cancer	Smoking	Number	1996	8.223644377	13.63119049	2.966540993
Deaths	Global	Both	30-34 years	Prostate cancer	Smoking	Rate	1996	0.001819338	0.003015664	0.000656296
Deaths	Global	Both	30-34 years	Prostate cancer	Smoking	Number	1997	8.271934783	13.56447366	2.903807908
Deaths	Global	Both	30-34 years	Prostate cancer	Smoking	Rate	1997	0.001789398	0.002934288	0.000628156
Deaths	Global	Both	30-34 years	Prostate cancer	Smoking	Number	1998	8.042805519	13.20526498	2.869783199
Deaths	Global	Both	30-34 years	Prostate cancer	Smoking	Rate	1998	0.001713308	0.002813034	0.000611332
Deaths	Global	Both	30-34 years	Prostate cancer	Smoking	Number	1999	7.818539369	12.84373031	2.75148197
Deaths	Global	Both	30-34 years	Prostate cancer	Smoking	Rate	1999	0.001647119	0.002705768	0.00057965
Deaths	Global	Both	30-34 years	Prostate cancer	Smoking	Number	2000	7.719198022	12.76977443	2.782991137

Deaths	Global	Both	30-34 years	Prostate cancer	Smoking	Rate	2000	0.001611185	0.002665364	0.000580878
Deaths	Global	Both	30-34 years	Prostate cancer	Smoking	Number	2001	7.504542415	12.26719395	2.802164222
Deaths	Global	Both	30-34 years	Prostate cancer	Smoking	Rate	2001	0.001550371	0.002534291	0.000578902
Deaths	Global	Both	30-34 years	Prostate cancer	Smoking	Number	2002	7.51980403	12.27415542	2.785907598
Deaths	Global	Both	30-34 years	Prostate cancer	Smoking	Rate	2002	0.001539443	0.002512747	0.000570327
Deaths	Global	Both	30-34 years	Prostate cancer	Smoking	Number	2003	7.621630352	12.31363991	2.894739818
Deaths	Global	Both	30-34 years	Prostate cancer	Smoking	Rate	2003	0.001548072	0.002501092	0.000587967
Deaths	Global	Both	30-34 years	Prostate cancer	Smoking	Number	2004	7.4986015	11.96503674	2.923771218
Deaths	Global	Both	30-34 years	Prostate cancer	Smoking	Rate	2004	0.001514047	0.002415868	0.00059034
Deaths	Global	Both	30-34 years	Prostate cancer	Smoking	Number	2005	7.524471389	11.75865569	3.03505734
Deaths	Global	Both	30-34 years	Prostate cancer	Smoking	Rate	2005	0.001513745	0.002365563	0.000610582

Deaths	Global	Both	30-34 years	Prostate cancer	Smoking	Number	2006	7.368354666	11.65973859	3.053515582
Deaths	Global	Both	30-34 years	Prostate cancer	Smoking	Rate	2006	0.001480102	0.002342124	0.000613368
Deaths	Global	Both	30-34 years	Prostate cancer	Smoking	Number	2007	7.203065926	11.37754627	3.022157086
Deaths	Global	Both	30-34 years	Prostate cancer	Smoking	Rate	2007	0.001445576	0.002283349	0.000606514
Deaths	Global	Both	30-34 years	Prostate cancer	Smoking	Number	2008	7.019440815	10.97816514	2.978732543
Deaths	Global	Both	30-34 years	Prostate cancer	Smoking	Rate	2008	0.001404664	0.002196846	0.000596076
Deaths	Global	Both	30-34 years	Prostate cancer	Smoking	Number	2009	6.820097221	10.70471887	2.837050666
Deaths	Global	Both	30-34 years	Prostate cancer	Smoking	Rate	2009	0.001355618	0.002127758	0.000563915
Deaths	Global	Both	30-34 years	Prostate cancer	Smoking	Number	2010	6.789546736	10.73259805	2.810092075
Deaths	Global	Both	30-34 years	Prostate cancer	Smoking	Rate	2010	0.001335839	0.002111632	0.000552884
Deaths	Global	Both	30-34 years	Prostate cancer	Smoking	Number	2011	6.83222698	10.84503874	2.868879084

Deaths	Global	Both	30-34 years	Prostate cancer	Smoking	Rate	2011	0.001328026	0.002108024	0.000557643
Deaths	Global	Both	30-34 years	Prostate cancer	Smoking	Number	2012	6.700996132	10.70380525	2.720712005
Deaths	Global	Both	30-34 years	Prostate cancer	Smoking	Rate	2012	0.0012839	0.002050832	0.000521284
Deaths	Global	Both	30-34 years	Prostate cancer	Smoking	Number	2013	6.366270209	10.19645083	2.587624026
Deaths	Global	Both	30-34 years	Prostate cancer	Smoking	Rate	2013	0.001199258	0.001920775	0.000487448
Deaths	Global	Both	30-34 years	Prostate cancer	Smoking	Number	2014	6.624621141	10.58473187	2.699930756
Deaths	Global	Both	30-34 years	Prostate cancer	Smoking	Rate	2014	0.001224315	0.001956195	0.000498982
Deaths	Global	Both	30-34 years	Prostate cancer	Smoking	Number	2015	6.488881531	10.49305499	2.625011942
Deaths	Global	Both	30-34 years	Prostate cancer	Smoking	Rate	2015	0.001174141	0.001898683	0.000474987
Deaths	Global	Both	30-34 years	Prostate cancer	Smoking	Number	2016	6.589584124	10.67086129	2.658290833
Deaths	Global	Both	30-34 years	Prostate cancer	Smoking	Rate	2016	0.001164847	0.001886299	0.000469909

Deaths	Global	Both	30-34 years	Prostate cancer	Smoking	Number	2017	6.849264253	11.23997574	2.683756702
Deaths	Global	Both	30-34 years	Prostate cancer	Smoking	Rate	2017	0.00118332	0.001941886	0.000463662
Deaths	Global	Both	30-34 years	Prostate cancer	Smoking	Number	2018	7.115337926	11.77299271	2.744516156
Deaths	Global	Both	30-34 years	Prostate cancer	Smoking	Rate	2018	0.001205293	0.00199427	0.000464904
Deaths	Global	Both	30-34 years	Prostate cancer	Smoking	Number	2019	7.227930868	12.05643806	2.894840462
Deaths	Global	Both	30-34 years	Prostate cancer	Smoking	Rate	2019	0.00120654	0.002012551	0.000483228
Deaths	Global	Both	30-34 years	Prostate cancer	Smoking	Number	2020	7.046763642	11.35134502	2.771111554
Deaths	Global	Both	30-34 years	Prostate cancer	Smoking	Rate	2020	0.001166615	0.001879253	0.000458767
Deaths	Global	Both	30-34 years	Prostate cancer	Smoking	Number	2021	7.021651952	11.27189372	2.834753079
Deaths	Global	Both	30-34 years	Prostate cancer	Smoking	Rate	2021	0.001161602	0.001864725	0.000468957
Deaths	Global	Both	35-39 years	Prostate cancer	Smoking	Number	1990	18.03222188	31.0427947	5.944430298

Deaths	Global	Both	35-39 years	Prostate cancer	Smoking	Rate	1990	0.005119235	0.008812855	0.001687587
Deaths	Global	Both	35-39 years	Prostate cancer	Smoking	Number	1991	18.69972833	31.70273119	6.105000651
Deaths	Global	Both	35-39 years	Prostate cancer	Smoking	Rate	1991	0.005178667	0.008779694	0.001690707
Deaths	Global	Both	35-39 years	Prostate cancer	Smoking	Number	1992	19.17069775	33.15803192	6.614396257
Deaths	Global	Both	35-39 years	Prostate cancer	Smoking	Rate	1992	0.005203846	0.009000679	0.001795464
Deaths	Global	Both	35-39 years	Prostate cancer	Smoking	Number	1993	19.45567569	33.2733401	6.589605152
Deaths	Global	Both	35-39 years	Prostate cancer	Smoking	Rate	1993	0.00521167	0.008913063	0.001765184
Deaths	Global	Both	35-39 years	Prostate cancer	Smoking	Number	1994	19.11518507	31.83468712	6.849712313
Deaths	Global	Both	35-39 years	Prostate cancer	Smoking	Rate	1994	0.005077429	0.008456019	0.00181944
Deaths	Global	Both	35-39 years	Prostate cancer	Smoking	Number	1995	18.62136421	31.06893227	6.478123819
Deaths	Global	Both	35-39 years	Prostate cancer	Smoking	Rate	1995	0.004906785	0.008186756	0.001707005

Deaths	Global	Both	35-39 years	Prostate cancer	Smoking	Number	1996	18.26713068	30.67870622	6.587848064
Deaths	Global	Both	35-39 years	Prostate cancer	Smoking	Rate	1996	0.004749834	0.007977102	0.001712978
Deaths	Global	Both	35-39 years	Prostate cancer	Smoking	Number	1997	18.16617561	30.12580923	6.419809412
Deaths	Global	Both	35-39 years	Prostate cancer	Smoking	Rate	1997	0.004622123	0.00766508	0.001633428
Deaths	Global	Both	35-39 years	Prostate cancer	Smoking	Number	1998	17.91681839	29.90194799	6.298193024
Deaths	Global	Both	35-39 years	Prostate cancer	Smoking	Rate	1998	0.00441757	0.007372624	0.001552882
Deaths	Global	Both	35-39 years	Prostate cancer	Smoking	Number	1999	17.75913841	29.5734425	6.041340157
Deaths	Global	Both	35-39 years	Prostate cancer	Smoking	Rate	1999	0.004227601	0.007040021	0.001438154
Deaths	Global	Both	35-39 years	Prostate cancer	Smoking	Number	2000	17.8085971	30.27219019	6.213655913
Deaths	Global	Both	35-39 years	Prostate cancer	Smoking	Rate	2000	0.00409999	0.006969424	0.001430541
Deaths	Global	Both	35-39 years	Prostate cancer	Smoking	Number	2001	17.49197189	29.39781366	6.294738091

Deaths	Global	Both	35-39 years	Prostate cancer	Smoking	Rate	2001	0.003916258	0.006581843	0.001409322
Deaths	Global	Both	35-39 years	Prostate cancer	Smoking	Number	2002	17.46772198	28.77709437	6.489672177
Deaths	Global	Both	35-39 years	Prostate cancer	Smoking	Rate	2002	0.003822529	0.006297402	0.00142016
Deaths	Global	Both	35-39 years	Prostate cancer	Smoking	Number	2003	16.68007548	27.34744656	6.594466336
Deaths	Global	Both	35-39 years	Prostate cancer	Smoking	Rate	2003	0.003592335	0.005889733	0.001420229
Deaths	Global	Both	35-39 years	Prostate cancer	Smoking	Number	2004	16.04341663	26.04234262	6.525562134
Deaths	Global	Both	35-39 years	Prostate cancer	Smoking	Rate	2004	0.003413823	0.005541459	0.001388552
Deaths	Global	Both	35-39 years	Prostate cancer	Smoking	Number	2005	15.76390621	25.32800008	6.447887074
Deaths	Global	Both	35-39 years	Prostate cancer	Smoking	Rate	2005	0.003319859	0.005334046	0.001357917
Deaths	Global	Both	35-39 years	Prostate cancer	Smoking	Number	2006	15.47232811	24.47182224	6.366621523
Deaths	Global	Both	35-39 years	Prostate cancer	Smoking	Rate	2006	0.003221666	0.005095552	0.001325665

Deaths	Global	Both	35-39 years	Prostate cancer	Smoking	Number	2007	15.11534935	23.90072893	6.322984074
Deaths	Global	Both	35-39 years	Prostate cancer	Smoking	Rate	2007	0.003115725	0.004926655	0.001303356
Deaths	Global	Both	35-39 years	Prostate cancer	Smoking	Number	2008	14.68494318	22.85282575	6.298852289
Deaths	Global	Both	35-39 years	Prostate cancer	Smoking	Rate	2008	0.003000305	0.004669098	0.001286929
Deaths	Global	Both	35-39 years	Prostate cancer	Smoking	Number	2009	14.11975087	22.06578969	5.989558786
Deaths	Global	Both	35-39 years	Prostate cancer	Smoking	Rate	2009	0.002865201	0.004477622	0.00121541
Deaths	Global	Both	35-39 years	Prostate cancer	Smoking	Number	2010	13.77610726	21.46748242	5.73916818
Deaths	Global	Both	35-39 years	Prostate cancer	Smoking	Rate	2010	0.002784448	0.00433904	0.00116009
Deaths	Global	Both	35-39 years	Prostate cancer	Smoking	Number	2011	13.53116752	21.02843965	5.831594496
Deaths	Global	Both	35-39 years	Prostate cancer	Smoking	Rate	2011	0.002731647	0.004245183	0.00117772
Deaths	Global	Both	35-39 years	Prostate cancer	Smoking	Number	2012	13.17525944	20.33021038	5.546215476

Deaths	Global	Both	35-39 years	Prostate cancer	Smoking	Rate	2012	0.0026587	0.004102533	0.001119198
Deaths	Global	Both	35-39 years	Prostate cancer	Smoking	Number	2013	12.72742465	19.90031653	5.414303812
Deaths	Global	Both	35-39 years	Prostate cancer	Smoking	Rate	2013	0.0025632 5	0.004007841	0.001090418
Deaths	Global	Both	35-39 years	Prostate cancer	Smoking	Number	2014	13.11476106	20.23905285	5.683574814
Deaths	Global	Both	35-39 years	Prostate cancer	Smoking	Rate	2014	0.0026264 7	0.004053239	0.001138239
Deaths	Global	Both	35-39 years	Prostate cancer	Smoking	Number	2015	12.95239111	20.07917364	5.528779424
Deaths	Global	Both	35-39 years	Prostate cancer	Smoking	Rate	2015	0.002568662	0.003982014	0.001096443
Deaths	Global	Both	35-39 years	Prostate cancer	Smoking	Number	2016	12.95925046	20.26183373	5.348411838
Deaths	Global	Both	35-39 years	Prostate cancer	Smoking	Rate	2016	0.002538902	0.003969583	0.00104783
Deaths	Global	Both	35-39 years	Prostate cancer	Smoking	Number	2017	13.14886182	21.16643607	5.583922569
Deaths	Global	Both	35-39 years	Prostate cancer	Smoking	Rate	2017	0.002539457	0.004087901	0.00107843

Deaths	Global	Both	35-39 years	Prostate cancer	Smoking	Number	2018	13.50334495	21.06080382	5.632282321
Deaths	Global	Both	35-39 years	Prostate cancer	Smoking	Rate	2018	0.002564417	0.003999652	0.001069625
Deaths	Global	Both	35-39 years	Prostate cancer	Smoking	Number	2019	13.65872986	22.02541054	5.762799257
Deaths	Global	Both	35-39 years	Prostate cancer	Smoking	Rate	2019	0.002544983	0.004103918	0.001073762
Deaths	Global	Both	35-39 years	Prostate cancer	Smoking	Number	2020	13.49376966	21.29307653	5.465566406
Deaths	Global	Both	35-39 years	Prostate cancer	Smoking	Rate	2020	0.002462014	0.003885041	0.000997223
Deaths	Global	Both	35-39 years	Prostate cancer	Smoking	Number	2021	13.9042771	22.26397367	5.700998801
Deaths	Global	Both	35-39 years	Prostate cancer	Smoking	Rate	2021	0.002479072	0.00396957	0.001016463
Deaths	Global	Both	40-44 years	Prostate cancer	Smoking	Number	1990	25.75157263	40.96282321	10.79556191
Deaths	Global	Both	40-44 years	Prostate cancer	Smoking	Rate	1990	0.008988909	0.014298586	0.003768326
Deaths	Global	Both	40-44 years	Prostate cancer	Smoking	Number	1991	27.57976317	44.34674813	11.62147219

Deaths	Global	Both	40-44 years	Prostate cancer	Smoking	Rate	1991	0.009177053	0.014756198	0.003866997
Deaths	Global	Both	40-44 years	Prostate cancer	Smoking	Number	1992	28.68616161	46.15874837	11.83993238
Deaths	Global	Both	40-44 years	Prostate cancer	Smoking	Rate	1992	0.00923254	0.014856031	0.003810641
Deaths	Global	Both	40-44 years	Prostate cancer	Smoking	Number	1993	29.91731946	48.04796918	12.58660348
Deaths	Global	Both	40-44 years	Prostate cancer	Smoking	Rate	1993	0.00929497	0.014927956	0.003910514
Deaths	Global	Both	40-44 years	Prostate cancer	Smoking	Number	1994	30.57033368	48.57274592	12.81974183
Deaths	Global	Both	40-44 years	Prostate cancer	Smoking	Rate	1994	0.009240806	0.014682579	0.003875154
Deaths	Global	Both	40-44 years	Prostate cancer	Smoking	Number	1995	31.49186825	50.11172864	13.39776146
Deaths	Global	Both	40-44 years	Prostate cancer	Smoking	Rate	1995	0.00917118	0.014593726	0.003901747
Deaths	Global	Both	40-44 years	Prostate cancer	Smoking	Number	1996	31.97104687	50.91822221	13.28892684
Deaths	Global	Both	40-44 years	Prostate cancer	Smoking	Rate	1996	0.009105716	0.014502087	0.003784837

Deaths	Global	Both	40-44 years	Prostate cancer	Smoking	Number	1997	32.01378829	50.8715406	13.70036868
Deaths	Global	Both	40-44 years	Prostate cancer	Smoking	Rate	1997	0.008954525	0.014229197	0.003832108
Deaths	Global	Both	40-44 years	Prostate cancer	Smoking	Number	1998	32.16959405	51.19327503	13.52415353
Deaths	Global	Both	40-44 years	Prostate cancer	Smoking	Rate	1998	0.008890332	0.014147683	0.003737511
Deaths	Global	Both	40-44 years	Prostate cancer	Smoking	Number	1999	32.11285903	50.27591198	13.40123914
Deaths	Global	Both	40-44 years	Prostate cancer	Smoking	Rate	1999	0.008803364	0.013782553	0.003673793
Deaths	Global	Both	40-44 years	Prostate cancer	Smoking	Number	2000	31.74933863	50.64602687	13.5255032
Deaths	Global	Both	40-44 years	Prostate cancer	Smoking	Rate	2000	0.008631467	0.013768775	0.003677082
Deaths	Global	Both	40-44 years	Prostate cancer	Smoking	Number	2001	31.18869807	49.64968127	13.04506537
Deaths	Global	Both	40-44 years	Prostate cancer	Smoking	Rate	2001	0.008360915	0.013309845	0.003497058
Deaths	Global	Both	40-44 years	Prostate cancer	Smoking	Number	2002	30.76391558	48.11759517	13.1064344

Deaths	Global	Both	40-44 years	Prostate cancer	Smoking	Rate	2002	0.008062756	0.012610892	0.003434998
Deaths	Global	Both	40-44 years	Prostate cancer	Smoking	Number	2003	30.23162504	46.64060954	12.8307485
Deaths	Global	Both	40-44 years	Prostate cancer	Smoking	Rate	2003	0.007670807	0.011834333	0.003255604
Deaths	Global	Both	40-44 years	Prostate cancer	Smoking	Number	2004	30.59158087	47.76924621	13.19682411
Deaths	Global	Both	40-44 years	Prostate cancer	Smoking	Rate	2004	0.007485999	0.011689507	0.003229366
Deaths	Global	Both	40-44 years	Prostate cancer	Smoking	Number	2005	31.51015043	48.92711579	13.71520446
Deaths	Global	Both	40-44 years	Prostate cancer	Smoking	Rate	2005	0.007448376	0.011565401	0.003242003
Deaths	Global	Both	40-44 years	Prostate cancer	Smoking	Number	2006	31.7315254	49.4246589	13.80806696
Deaths	Global	Both	40-44 years	Prostate cancer	Smoking	Rate	2006	0.007286471	0.011349323	0.003170729
Deaths	Global	Both	40-44 years	Prostate cancer	Smoking	Number	2007	31.13565577	48.82547863	13.99800519
Deaths	Global	Both	40-44 years	Prostate cancer	Smoking	Rate	2007	0.006980274	0.010946138	0.0031382

Deaths	Global	Both	40-44 years	Prostate cancer	Smoking	Number	2008	30.22781695	46.81913594	13.32477168
Deaths	Global	Both	40-44 years	Prostate cancer	Smoking	Rate	2008	0.00666168	0.010318116	0.002936546
Deaths	Global	Both	40-44 years	Prostate cancer	Smoking	Number	2009	29.12865148	45.0184518	12.7156359 5
Deaths	Global	Both	40-44 years	Prostate cancer	Smoking	Rate	2009	0.00633597	0.009792268	0.002765864
Deaths	Global	Both	40-44 years	Prostate cancer	Smoking	Number	2010	28.58756956	44.0290313 7	12.45386392
Deaths	Global	Both	40-44 years	Prostate cancer	Smoking	Rate	2010	0.006150215	0.00947223	0.002679275
Deaths	Global	Both	40-44 years	Prostate cancer	Smoking	Number	2011	28.49030996	44.11049058	12.51462277
Deaths	Global	Both	40-44 years	Prostate cancer	Smoking	Rate	2011	0.006057828	0.009379111	0.002660955
Deaths	Global	Both	40-44 years	Prostate cancer	Smoking	Number	2012	28.03283834	43.53624984	12.19523511
Deaths	Global	Both	40-44 years	Prostate cancer	Smoking	Rate	2012	0.005899356	0.009161964	0.00256642
Deaths	Global	Both	40-44 years	Prostate cancer	Smoking	Number	2013	27.71138717	43.41803517	12.06639409

Deaths	Global	Both	40-44 years	Prostate cancer	Smoking	Rate	2013	0.005780429	0.009056742	0.002516978
Deaths	Global	Both	40-44 years	Prostate cancer	Smoking	Number	2014	28.17569611	43.33752612	12.12260101
Deaths	Global	Both	40-44 years	Prostate cancer	Smoking	Rate	2014	0.005838744	0.008980673	0.002512121
Deaths	Global	Both	40-44 years	Prostate cancer	Smoking	Number	2015	27.82367965	43.45002275	12.12590181
Deaths	Global	Both	40-44 years	Prostate cancer	Smoking	Rate	2015	0.005743223	0.008968733	0.002502967
Deaths	Global	Both	40-44 years	Prostate cancer	Smoking	Number	2016	27.46526547	42.30468118	11.57128065
Deaths	Global	Both	40-44 years	Prostate cancer	Smoking	Rate	2016	0.005661543	0.008720461	0.002385242
Deaths	Global	Both	40-44 years	Prostate cancer	Smoking	Number	2017	26.99203837	43.13368347	11.14794643
Deaths	Global	Both	40-44 years	Prostate cancer	Smoking	Rate	2017	0.005561095	0.008886713	0.00229678
Deaths	Global	Both	40-44 years	Prostate cancer	Smoking	Number	2018	26.92028541	41.26276754	11.30119976
Deaths	Global	Both	40-44 years	Prostate cancer	Smoking	Rate	2018	0.005534794	0.0084836	0.00232352

Deaths	Global	Both	40-44 years	Prostate cancer	Smoking	Number	2019	26.95702045	42.02385576	11.36396569
Deaths	Global	Both	40-44 years	Prostate cancer	Smoking	Rate	2019	0.005509903	0.008589501	0.002322747
Deaths	Global	Both	40-44 years	Prostate cancer	Smoking	Number	2020	26.45824777	41.16268179	11.32964094
Deaths	Global	Both	40-44 years	Prostate cancer	Smoking	Rate	2020	0.005353838	0.008329287	0.002292558
Deaths	Global	Both	40-44 years	Prostate cancer	Smoking	Number	2021	26.61539557	41.3586591	11.23815831
Deaths	Global	Both	40-44 years	Prostate cancer	Smoking	Rate	2021	0.0053204 1	0.008267585	0.002246505
Deaths	Global	Both	45-49 years	Prostate cancer	Smoking	Number	1990	71.88435104	111.4604715	32.35602146
Deaths	Global	Both	45-49 years	Prostate cancer	Smoking	Rate	1990	0.030958547	0.048002858	0.013934819
Deaths	Global	Both	45-49 years	Prostate cancer	Smoking	Number	1991	72.6590276	111.7205866	32.28385704
Deaths	Global	Both	45-49 years	Prostate cancer	Smoking	Rate	1991	0.030867734	0.047462255	0.01371515
Deaths	Global	Both	45-49 years	Prostate cancer	Smoking	Number	1992	75.43228639	116.2492552	33.91754101

Deaths	Global	Both	45-49 years	Prostate cancer	Smoking	Rate	1992	0.030966707	0.047723022	0.013923939
Deaths	Global	Both	45-49 years	Prostate cancer	Smoking	Number	1993	78.68221102	121.0380023	35.2981485
Deaths	Global	Both	45-49 years	Prostate cancer	Smoking	Rate	1993	0.031154669	0.047925685	0.013976503
Deaths	Global	Both	45-49 years	Prostate cancer	Smoking	Number	1994	83.93558179	128.6220269	38.2147779
Deaths	Global	Both	45-49 years	Prostate cancer	Smoking	Rate	1994	0.031646246	0.048494384	0.014408124
Deaths	Global	Both	45-49 years	Prostate cancer	Smoking	Number	1995	87.83250393	134.6308161	39.28512949
Deaths	Global	Both	45-49 years	Prostate cancer	Smoking	Rate	1995	0.031870447	0.04885144	0.014254798
Deaths	Global	Both	45-49 years	Prostate cancer	Smoking	Number	1996	92.75970042	142.3590678	42.09854217
Deaths	Global	Both	45-49 years	Prostate cancer	Smoking	Rate	1996	0.032129664	0.049309657	0.014581893
Deaths	Global	Both	45-49 years	Prostate cancer	Smoking	Number	1997	94.62690731	145.0232382	42.43656112
Deaths	Global	Both	45-49 years	Prostate cancer	Smoking	Rate	1997	0.031746176	0.048653532	0.01423695

Deaths	Global	Both	45-49 years	Prostate cancer	Smoking	Number	1998	96.69370169	149.7180264	43.4354091
Deaths	Global	Both	45-49 years	Prostate cancer	Smoking	Rate	1998	0.031343281	0.048531126	0.014079596
Deaths	Global	Both	45-49 years	Prostate cancer	Smoking	Number	1999	97.00513986	150.5718351	42.79843629
Deaths	Global	Both	45-49 years	Prostate cancer	Smoking	Rate	1999	0.030611603	0.047515474	0.013505766
Deaths	Global	Both	45-49 years	Prostate cancer	Smoking	Number	2000	99.69606327	155.974584	44.32059026
Deaths	Global	Both	45-49 years	Prostate cancer	Smoking	Rate	2000	0.030305904	0.047413615	0.013472704
Deaths	Global	Both	45-49 years	Prostate cancer	Smoking	Number	2001	99.94011625	156.5682592	44.5259935
Deaths	Global	Both	45-49 years	Prostate cancer	Smoking	Rate	2001	0.029685	0.046505137	0.013225461
Deaths	Global	Both	45-49 years	Prostate cancer	Smoking	Number	2002	100.4831108	156.8436364	45.15518707
Deaths	Global	Both	45-49 years	Prostate cancer	Smoking	Rate	2002	0.029278432	0.045700572	0.013157167
Deaths	Global	Both	45-49 years	Prostate cancer	Smoking	Number	2003	100.4542958	155.0121771	46.07662696

Deaths	Global	Both	45-49 years	Prostate cancer	Smoking	Rate	2003	0.028880508	0.044565844	0.013246984
Deaths	Global	Both	45-49 years	Prostate cancer	Smoking	Number	2004	99.84781329	155.4245425	45.76964635
Deaths	Global	Both	45-49 years	Prostate cancer	Smoking	Rate	2004	0.028432864	0.044259005	0.013033456
Deaths	Global	Both	45-49 years	Prostate cancer	Smoking	Number	2005	98.72415018	152.0523136	45.45085941
Deaths	Global	Both	45-49 years	Prostate cancer	Smoking	Rate	2005	0.027839673	0.042877925	0.012816895
Deaths	Global	Both	45-49 years	Prostate cancer	Smoking	Number	2006	96.59270133	147.8225729	44.20073247
Deaths	Global	Both	45-49 years	Prostate cancer	Smoking	Rate	2006	0.026822927	0.041049003	0.012274147
Deaths	Global	Both	45-49 years	Prostate cancer	Smoking	Number	2007	94.63342393	145.1888409	43.90844108
Deaths	Global	Both	45-49 years	Prostate cancer	Smoking	Rate	2007	0.025654431	0.039359636	0.011903258
Deaths	Global	Both	45-49 years	Prostate cancer	Smoking	Number	2008	94.33002909	144.8694784	44.09301912
Deaths	Global	Both	45-49 years	Prostate cancer	Smoking	Rate	2008	0.0247160 8	0.03795828	0.011553125

Deaths	Global	Both	45-49 years	Prostate cancer	Smoking	Number	2009	94.24810866	144.6069599	43.2613788 8
Deaths	Global	Both	45-49 years	Prostate cancer	Smoking	Rate	2009	0.023777194	0.036481875	0.0109141 1
Deaths	Global	Both	45-49 years	Prostate cancer	Smoking	Number	2010	94.17970132	143.1407732	42.2670637 5
Deaths	Global	Both	45-49 years	Prostate cancer	Smoking	Rate	2010	0.02292015	0.034835617	0.01028637 2
Deaths	Global	Both	45-49 years	Prostate cancer	Smoking	Number	2011	93.55297694	144.6519874	42.1982361
Deaths	Global	Both	45-49 years	Prostate cancer	Smoking	Rate	2011	0.02209666	0.034165944	0.00996697 4
Deaths	Global	Both	45-49 years	Prostate cancer	Smoking	Number	2012	92.78750902	143.3850088	41.9475892 7
Deaths	Global	Both	45-49 years	Prostate cancer	Smoking	Rate	2012	0.021378469	0.033036256	0.00966482 7
Deaths	Global	Both	45-49 years	Prostate cancer	Smoking	Number	2013	90.02791645	140.9846571	40.5191746 3
Deaths	Global	Both	45-49 years	Prostate cancer	Smoking	Rate	2013	0.020378253	0.031912557	0.00917171 1
Deaths	Global	Both	45-49 years	Prostate cancer	Smoking	Number	2014	87.47604705	135.9505034	39.1870833 9

Deaths	Global	Both	45-49 years	Prostate cancer	Smoking	Rate	2014	0.019536005	0.030361794	0.008751642
Deaths	Global	Both	45-49 years	Prostate cancer	Smoking	Number	2015	85.71775291	132.2935797	37.8384548
Deaths	Global	Both	45-49 years	Prostate cancer	Smoking	Rate	2015	0.018924871	0.02920794	0.008354021
Deaths	Global	Both	45-49 years	Prostate cancer	Smoking	Number	2016	85.36636646	131.3931904	37.87053302
Deaths	Global	Both	45-49 years	Prostate cancer	Smoking	Rate	2016	0.018614126	0.028650269	0.008257665
Deaths	Global	Both	45-49 years	Prostate cancer	Smoking	Number	2017	85.02857628	135.8104608	37.02915688
Deaths	Global	Both	45-49 years	Prostate cancer	Smoking	Rate	2017	0.018339709	0.029292792	0.007986773
Deaths	Global	Both	45-49 years	Prostate cancer	Smoking	Number	2018	84.31344836	129.6165909	37.24811667
Deaths	Global	Both	45-49 years	Prostate cancer	Smoking	Rate	2018	0.018017175	0.027698129	0.007959653
Deaths	Global	Both	45-49 years	Prostate cancer	Smoking	Number	2019	84.62490759	133.8160371	36.7305752
Deaths	Global	Both	45-49 years	Prostate cancer	Smoking	Rate	2019	0.017958292	0.028397166	0.007794613

Deaths	Global	Both	45-49 years	Prostate cancer	Smoking	Number	2020	83.71576797	128.527256	36.67086527
Deaths	Global	Both	45-49 years	Prostate cancer	Smoking	Rate	2020	0.017695698	0.027167875	0.007751426
Deaths	Global	Both	45-49 years	Prostate cancer	Smoking	Number	2021	83.45536741	129.66151	36.3778426
Deaths	Global	Both	45-49 years	Prostate cancer	Smoking	Rate	2021	0.017625037	0.027383367	0.007682679
Deaths	Global	Both	50-54 years	Prostate cancer	Smoking	Number	1990	217.5898168	335.4659053	101.2929037
Deaths	Global	Both	50-54 years	Prostate cancer	Smoking	Rate	1990	0.102360782	0.157813233	0.047651223
Deaths	Global	Both	50-54 years	Prostate cancer	Smoking	Number	1991	222.1091212	341.2919018	102.7802357
Deaths	Global	Both	50-54 years	Prostate cancer	Smoking	Rate	1991	0.1033638	0.158828361	0.047831244
Deaths	Global	Both	50-54 years	Prostate cancer	Smoking	Number	1992	225.5021197	346.1050239	105.3573002
Deaths	Global	Both	50-54 years	Prostate cancer	Smoking	Rate	1992	0.104251207	0.160006773	0.048707417
Deaths	Global	Both	50-54 years	Prostate cancer	Smoking	Number	1993	227.1814712	346.0240468	105.722383

Deaths	Global	Both	50-54 years	Prostate cancer	Smoking	Rate	1993	0.104575758	0.159281154	0.048665867
Deaths	Global	Both	50-54 years	Prostate cancer	Smoking	Number	1994	226.5045147	346.4491821	105.366841 7
Deaths	Global	Both	50-54 years	Prostate cancer	Smoking	Rate	1994	0.103830167	0.158813066	0.048300392
Deaths	Global	Both	50-54 years	Prostate cancer	Smoking	Number	1995	224.6131505	342.1422833	103.0907264
Deaths	Global	Both	50-54 years	Prostate cancer	Smoking	Rate	1995	0.10267209	0.15639539 9	0.047123422
Deaths	Global	Both	50-54 years	Prostate cancer	Smoking	Number	1996	224.6744648	343.8909339	103.7813539
Deaths	Global	Both	50-54 years	Prostate cancer	Smoking	Rate	1996	0.10137431	0.155165413	0.046826697
Deaths	Global	Both	50-54 years	Prostate cancer	Smoking	Number	1997	228.2283952	349.2150027	103.2078887
Deaths	Global	Both	50-54 years	Prostate cancer	Smoking	Rate	1997	0.099412132	0.152111694	0.044955476
Deaths	Global	Both	50-54 years	Prostate cancer	Smoking	Number	1998	234.5300215	363.6936034	107.3079984
Deaths	Global	Both	50-54 years	Prostate cancer	Smoking	Rate	1998	0.098414706	0.152614999	0.0450291 4

Deaths	Global	Both	50-54 years	Prostate cancer	Smoking	Number	1999	243.2003448	374.3336677	110.8140299
Deaths	Global	Both	50-54 years	Prostate cancer	Smoking	Rate	1999	0.096977169	0.149267137	0.044187564
Deaths	Global	Both	50-54 years	Prostate cancer	Smoking	Number	2000	253.5674211	392.3885086	115.8813817
Deaths	Global	Both	50-54 years	Prostate cancer	Smoking	Rate	2000	0.097107411	0.150271009	0.044378497
Deaths	Global	Both	50-54 years	Prostate cancer	Smoking	Number	2001	266.3881517	407.0276519	123.0282523
Deaths	Global	Both	50-54 years	Prostate cancer	Smoking	Rate	2001	0.097210718	0.148533071	0.044895633
Deaths	Global	Both	50-54 years	Prostate cancer	Smoking	Number	2002	271.5288647	415.6941819	123.8610278
Deaths	Global	Both	50-54 years	Prostate cancer	Smoking	Rate	2002	0.095818441	0.146692207	0.043708689
Deaths	Global	Both	50-54 years	Prostate cancer	Smoking	Number	2003	273.4145219	415.2206991	126.853759
Deaths	Global	Both	50-54 years	Prostate cancer	Smoking	Rate	2003	0.09305449	0.141317112	0.043173683
Deaths	Global	Both	50-54 years	Prostate cancer	Smoking	Number	2004	274.0350004	421.7779551	127.7310873

Deaths	Global	Both	50-54 years	Prostate cancer	Smoking	Rate	2004	0.090630002	0.13949217	0.0422437 6
Deaths	Global	Both	50-54 years	Prostate cancer	Smoking	Number	2005	277.3410401	422.7274139	128.3009819
Deaths	Global	Both	50-54 years	Prostate cancer	Smoking	Rate	2005	0.088174131	0.134396345	0.040790312
Deaths	Global	Both	50-54 years	Prostate cancer	Smoking	Number	2006	275.2314197	415.1001734	127.2257031
Deaths	Global	Both	50-54 years	Prostate cancer	Smoking	Rate	2006	0.085330036	0.128693565	0.039443803
Deaths	Global	Both	50-54 years	Prostate cancer	Smoking	Number	2007	271.4442646	411.0599811	127.1273817
Deaths	Global	Both	50-54 years	Prostate cancer	Smoking	Rate	2007	0.082408012	0.124794075	0.038594718
Deaths	Global	Both	50-54 years	Prostate cancer	Smoking	Number	2008	271.6037507	413.1255025	126.894299
Deaths	Global	Both	50-54 years	Prostate cancer	Smoking	Rate	2008	0.081233081	0.12356036	0.037952402
Deaths	Global	Both	50-54 years	Prostate cancer	Smoking	Number	2009	267.8302728	404.2259176	125.0190716
Deaths	Global	Both	50-54 years	Prostate cancer	Smoking	Rate	2009	0.079233784	0.119584499	0.036985117

Deaths	Global	Both	50-54 years	Prostate cancer	Smoking	Number	2010	265.0131662	401.5603579	122.8779808
Deaths	Global	Both	50-54 years	Prostate cancer	Smoking	Rate	2010	0.077542901	0.117496635	0.0359541 2
Deaths	Global	Both	50-54 years	Prostate cancer	Smoking	Number	2011	262.3243315	404.5203318	122.566888 8
Deaths	Global	Both	50-54 years	Prostate cancer	Smoking	Rate	2011	0.075500394	0.116426274	0.035276363
Deaths	Global	Both	50-54 years	Prostate cancer	Smoking	Number	2012	261.4891211	401.5182969	121.275149 5
Deaths	Global	Both	50-54 years	Prostate cancer	Smoking	Rate	2012	0.073390936	0.112692274	0.034037732
Deaths	Global	Both	50-54 years	Prostate cancer	Smoking	Number	2013	255.7113338	396.9834407	119.215794 6
Deaths	Global	Both	50-54 years	Prostate cancer	Smoking	Rate	2013	0.06928928	0.10756933	0.032303522
Deaths	Global	Both	50-54 years	Prostate cancer	Smoking	Number	2014	254.7789172	391.8831323	117.263937 7
Deaths	Global	Both	50-54 years	Prostate cancer	Smoking	Rate	2014	0.066409174	0.102145952	0.030565328
Deaths	Global	Both	50-54 years	Prostate cancer	Smoking	Number	2015	252.7869867	387.964087	114.759834 2

Deaths	Global	Both	50-54 years	Prostate cancer	Smoking	Rate	2015	0.063507752	0.097468336	0.028831148
Deaths	Global	Both	50-54 years	Prostate cancer	Smoking	Number	2016	254.3233908	391.764273	116.6216992
Deaths	Global	Both	50-54 years	Prostate cancer	Smoking	Rate	2016	0.061968818	0.09545787	0.028416218
Deaths	Global	Both	50-54 years	Prostate cancer	Smoking	Number	2017	255.0295566	401.7893259	113.8456675
Deaths	Global	Both	50-54 years	Prostate cancer	Smoking	Rate	2017	0.060582759	0.095445823	0.027044256
Deaths	Global	Both	50-54 years	Prostate cancer	Smoking	Number	2018	252.9147913	387.4042895	112.270363
Deaths	Global	Both	50-54 years	Prostate cancer	Smoking	Rate	2018	0.05899847	0.090371387	0.026189768
Deaths	Global	Both	50-54 years	Prostate cancer	Smoking	Number	2019	253.5924037	397.8467294	113.8243846
Deaths	Global	Both	50-54 years	Prostate cancer	Smoking	Rate	2019	0.058344248	0.091532979	0.026187685
Deaths	Global	Both	50-54 years	Prostate cancer	Smoking	Number	2020	252.6583906	389.2359545	110.7532643
Deaths	Global	Both	50-54 years	Prostate cancer	Smoking	Rate	2020	0.057467599	0.088532408	0.025191026

Deaths	Global	Both	50-54 years	Prostate cancer	Smoking	Number	2021	253.9308871	397.0321547	115.296954 6
Deaths	Global	Both	50-54 years	Prostate cancer	Smoking	Rate	2021	0.057072998	0.089236153	0.025913913
Deaths	Global	Both	55-59 years	Prostate cancer	Smoking	Number	1990	478.6517694	733.7444159	220.384013 9
Deaths	Global	Both	55-59 years	Prostate cancer	Smoking	Rate	1990	0.258451337	0.396190377	0.118997874
Deaths	Global	Both	55-59 years	Prostate cancer	Smoking	Number	1991	478.8100805	737.9535991	218.513593 5
Deaths	Global	Both	55-59 years	Prostate cancer	Smoking	Rate	1991	0.254901687	0.392860603	0.11632897
Deaths	Global	Both	55-59 years	Prostate cancer	Smoking	Number	1992	485.8328968	753.4889644	222.539538 9
Deaths	Global	Both	55-59 years	Prostate cancer	Smoking	Rate	1992	0.254224459	0.394282326	0.116449492
Deaths	Global	Both	55-59 years	Prostate cancer	Smoking	Number	1993	495.4348666	760.2134947	226.287031 8
Deaths	Global	Both	55-59 years	Prostate cancer	Smoking	Rate	1993	0.255055318	0.391366267	0.116495053
Deaths	Global	Both	55-59 years	Prostate cancer	Smoking	Number	1994	508.2741424	781.6152973	234.888577 6

Deaths	Global	Both	55-59 years	Prostate cancer	Smoking	Rate	1994	0.25800224	0.39675144	0.119230498
Deaths	Global	Both	55-59 years	Prostate cancer	Smoking	Number	1995	513.5321149	791.4120431	234.6990352
Deaths	Global	Both	55-59 years	Prostate cancer	Smoking	Rate	1995	0.257350497	0.39660671	0.117616623
Deaths	Global	Both	55-59 years	Prostate cancer	Smoking	Number	1996	518.8614648	804.3549344	238.890577
Deaths	Global	Both	55-59 years	Prostate cancer	Smoking	Rate	1996	0.257696588	0.399489144	0.118646866
Deaths	Global	Both	55-59 years	Prostate cancer	Smoking	Number	1997	513.5497795	799.7983186	234.6534625
Deaths	Global	Both	55-59 years	Prostate cancer	Smoking	Rate	1997	0.253716453	0.395135975	0.115929257
Deaths	Global	Both	55-59 years	Prostate cancer	Smoking	Number	1998	508.9710575	789.2248302	234.2548923
Deaths	Global	Both	55-59 years	Prostate cancer	Smoking	Rate	1998	0.250482303	0.3884049	0.115284954
Deaths	Global	Both	55-59 years	Prostate cancer	Smoking	Number	1999	499.5894187	775.842506	230.9640717
Deaths	Global	Both	55-59 years	Prostate cancer	Smoking	Rate	1999	0.244698831	0.380007557	0.113126172

Deaths	Global	Both	55-59 years	Prostate cancer	Smoking	Number	2000	486.4382185	757.5117181	226.743598
Deaths	Global	Both	55-59 years	Prostate cancer	Smoking	Rate	2000	0.237231893	0.369432196	0.110595979
Deaths	Global	Both	55-59 years	Prostate cancer	Smoking	Number	2001	486.9055889	756.3424187	227.3460451
Deaths	Global	Both	55-59 years	Prostate cancer	Smoking	Rate	2001	0.233850144	0.363254783	0.109189351
Deaths	Global	Both	55-59 years	Prostate cancer	Smoking	Number	2002	494.6234371	764.4495577	231.0022088
Deaths	Global	Both	55-59 years	Prostate cancer	Smoking	Rate	2002	0.228529824	0.353197018	0.106729463
Deaths	Global	Both	55-59 years	Prostate cancer	Smoking	Number	2003	506.2852317	779.2318468	237.8360883
Deaths	Global	Both	55-59 years	Prostate cancer	Smoking	Rate	2003	0.224560922	0.345625373	0.105491308
Deaths	Global	Both	55-59 years	Prostate cancer	Smoking	Number	2004	528.3653413	822.8762921	248.3890905
Deaths	Global	Both	55-59 years	Prostate cancer	Smoking	Rate	2004	0.221896622	0.345581845	0.10431551
Deaths	Global	Both	55-59 years	Prostate cancer	Smoking	Number	2005	553.9390904	852.1715669	259.1400801

Deaths	Global	Both	55-59 years	Prostate cancer	Smoking	Rate	2005	0.222776603	0.342716177	0.104217861
Deaths	Global	Both	55-59 years	Prostate cancer	Smoking	Number	2006	570.8335439	876.7707294	266.0715385
Deaths	Global	Both	55-59 years	Prostate cancer	Smoking	Rate	2006	0.21822831	0.33518737	0.101718518
Deaths	Global	Both	55-59 years	Prostate cancer	Smoking	Number	2007	569.3739806	871.4623592	262.462225
Deaths	Global	Both	55-59 years	Prostate cancer	Smoking	Rate	2007	0.210132847	0.321621417	0.096864164
Deaths	Global	Both	55-59 years	Prostate cancer	Smoking	Number	2008	577.0141219	881.3894908	267.941151
Deaths	Global	Both	55-59 years	Prostate cancer	Smoking	Rate	2008	0.20505768	0.313225755	0.095220184
Deaths	Global	Both	55-59 years	Prostate cancer	Smoking	Number	2009	577.3387929	881.1242924	266.1648001
Deaths	Global	Both	55-59 years	Prostate cancer	Smoking	Rate	2009	0.199088719	0.303845695	0.091783905
Deaths	Global	Both	55-59 years	Prostate cancer	Smoking	Number	2010	585.2538496	888.3721194	271.9664619
Deaths	Global	Both	55-59 years	Prostate cancer	Smoking	Rate	2010	0.193710726	0.294038575	0.090017042

Deaths	Global	Both	55-59 years	Prostate cancer	Smoking	Number	2011	588.6647846	898.8177426	270.2654285
Deaths	Global	Both	55-59 years	Prostate cancer	Smoking	Rate	2011	0.189815202	0.28982415	0.087147198
Deaths	Global	Both	55-59 years	Prostate cancer	Smoking	Number	2012	592.7236556	900.5414718	273.2108343
Deaths	Global	Both	55-59 years	Prostate cancer	Smoking	Rate	2012	0.187021662	0.284147193	0.086206015
Deaths	Global	Both	55-59 years	Prostate cancer	Smoking	Number	2013	587.6098033	907.7065325	267.9946256
Deaths	Global	Both	55-59 years	Prostate cancer	Smoking	Rate	2013	0.182569534	0.282023134	0.083265551
Deaths	Global	Both	55-59 years	Prostate cancer	Smoking	Number	2014	587.1854016	901.539839	266.6206147
Deaths	Global	Both	55-59 years	Prostate cancer	Smoking	Rate	2014	0.180392246	0.276966688	0.08190989
Deaths	Global	Both	55-59 years	Prostate cancer	Smoking	Number	2015	587.5410398	904.7490823	271.7163158
Deaths	Global	Both	55-59 years	Prostate cancer	Smoking	Rate	2015	0.178471024	0.27482459	0.082536344
Deaths	Global	Both	55-59 years	Prostate cancer	Smoking	Number	2016	591.4826813	911.6644333	273.5056349

Deaths	Global	Both	55-59 years	Prostate cancer	Smoking	Rate	2016	0.176659548	0.272288998	0.081688583
Deaths	Global	Both	55-59 years	Prostate cancer	Smoking	Number	2017	589.4765617	920.8088799	266.2024169
Deaths	Global	Both	55-59 years	Prostate cancer	Smoking	Rate	2017	0.171624085	0.268090356	0.077503923
Deaths	Global	Both	55-59 years	Prostate cancer	Smoking	Number	2018	593.2979796	923.2119703	273.2101627
Deaths	Global	Both	55-59 years	Prostate cancer	Smoking	Rate	2018	0.166692542	0.259384923	0.076760916
Deaths	Global	Both	55-59 years	Prostate cancer	Smoking	Number	2019	598.4661662	935.6558402	272.2237903
Deaths	Global	Both	55-59 years	Prostate cancer	Smoking	Rate	2019	0.161663607	0.252748621	0.073535786
Deaths	Global	Both	55-59 years	Prostate cancer	Smoking	Number	2020	596.3860645	920.8988619	270.3237005
Deaths	Global	Both	55-59 years	Prostate cancer	Smoking	Rate	2020	0.15526517	0.239749932	0.070376989
Deaths	Global	Both	55-59 years	Prostate cancer	Smoking	Number	2021	600.2258053	929.3894794	271.4354734
Deaths	Global	Both	55-59 years	Prostate cancer	Smoking	Rate	2021	0.151676353	0.234855626	0.068591424

Deaths	Global	Both	60-64 years	Prostate cancer	Smoking	Number	1990	1113.185502	1699.807472	528.440687 2
Deaths	Global	Both	60-64 years	Prostate cancer	Smoking	Rate	1990	0.693101794	1.05834976	0.3290226
Deaths	Global	Both	60-64 years	Prostate cancer	Smoking	Number	1991	1128.946956	1728.775549	533.8153705
Deaths	Global	Both	60-64 years	Prostate cancer	Smoking	Rate	1991	0.688740112	1.054679371	0.325666371
Deaths	Global	Both	60-64 years	Prostate cancer	Smoking	Number	1992	1133.721993	1741.16072	534.257098
Deaths	Global	Both	60-64 years	Prostate cancer	Smoking	Rate	1992	0.681741144	1.047012326	0.321264867
Deaths	Global	Both	60-64 years	Prostate cancer	Smoking	Number	1993	1125.779144	1717.061212	531.960756 3
Deaths	Global	Both	60-64 years	Prostate cancer	Smoking	Rate	1993	0.669087778	1.020506266	0.316161871
Deaths	Global	Both	60-64 years	Prostate cancer	Smoking	Number	1994	1118.96566	1710.410948	526.810366 7
Deaths	Global	Both	60-64 years	Prostate cancer	Smoking	Rate	1994	0.658546094	1.006630041	0.310044286
Deaths	Global	Both	60-64 years	Prostate cancer	Smoking	Number	1995	1101.880905	1692.310695	518.063656 1

Deaths	Global	Both	60-64 years	Prostate cancer	Smoking	Rate	1995	0.64140888	0.985100206	0.301566737
Deaths	Global	Both	60-64 years	Prostate cancer	Smoking	Number	1996	1090.98779	1677.919013	508.4065285
Deaths	Global	Both	60-64 years	Prostate cancer	Smoking	Rate	1996	0.625924613	0.962660461	0.291684437
Deaths	Global	Both	60-64 years	Prostate cancer	Smoking	Number	1997	1080.676888	1682.608623	500.6572947
Deaths	Global	Both	60-64 years	Prostate cancer	Smoking	Rate	1997	0.608805347	0.947906945	0.282048077
Deaths	Global	Both	60-64 years	Prostate cancer	Smoking	Number	1998	1092.987951	1700.183288	503.3779519
Deaths	Global	Both	60-64 years	Prostate cancer	Smoking	Rate	1998	0.604928498	0.940988711	0.278601121
Deaths	Global	Both	60-64 years	Prostate cancer	Smoking	Number	1999	1102.141807	1712.696811	504.7025461
Deaths	Global	Both	60-64 years	Prostate cancer	Smoking	Rate	1999	0.600367888	0.932954507	0.274925785
Deaths	Global	Both	60-64 years	Prostate cancer	Smoking	Number	2000	1112.349566	1730.728383	511.6462671
Deaths	Global	Both	60-64 years	Prostate cancer	Smoking	Rate	2000	0.59697477	0.928845761	0.274589861

Deaths	Global	Both	60-64 years	Prostate cancer	Smoking	Number	2001	1113.56582	1724.090544	513.7851293
Deaths	Global	Both	60-64 years	Prostate cancer	Smoking	Rate	2001	0.590979081	0.91498987	0.272670244
Deaths	Global	Both	60-64 years	Prostate cancer	Smoking	Number	2002	1109.55216	1713.883139	513.2791564
Deaths	Global	Both	60-64 years	Prostate cancer	Smoking	Rate	2002	0.584442429	0.902766053	0.27036324
Deaths	Global	Both	60-64 years	Prostate cancer	Smoking	Number	2003	1085.968706	1667.875533	504.3955204
Deaths	Global	Both	60-64 years	Prostate cancer	Smoking	Rate	2003	0.56819893	0.872663353	0.263909073
Deaths	Global	Both	60-64 years	Prostate cancer	Smoking	Number	2004	1056.60018	1644.67967	489.0062652
Deaths	Global	Both	60-64 years	Prostate cancer	Smoking	Rate	2004	0.548325423	0.85351081	0.253771078
Deaths	Global	Both	60-64 years	Prostate cancer	Smoking	Number	2005	1024.012451	1587.707346	474.1896803
Deaths	Global	Both	60-64 years	Prostate cancer	Smoking	Rate	2005	0.527127911	0.817299492	0.244097242
Deaths	Global	Both	60-64 years	Prostate cancer	Smoking	Number	2006	1002.251091	1550.581447	462.1492977

Deaths	Global	Both	60-64 years	Prostate cancer	Smoking	Rate	2006	0.506327843	0.783339192	0.233473487
Deaths	Global	Both	60-64 years	Prostate cancer	Smoking	Number	2007	1012.604205	1556.888686	469.1103878
Deaths	Global	Both	60-64 years	Prostate cancer	Smoking	Rate	2007	0.490319187	0.753869585	0.227150769
Deaths	Global	Both	60-64 years	Prostate cancer	Smoking	Number	2008	1043.870553	1608.087731	486.0731734
Deaths	Global	Both	60-64 years	Prostate cancer	Smoking	Rate	2008	0.483935924	0.745505675	0.225342376
Deaths	Global	Both	60-64 years	Prostate cancer	Smoking	Number	2009	1085.103894	1665.687199	503.9025225
Deaths	Global	Both	60-64 years	Prostate cancer	Smoking	Rate	2009	0.475377149	0.729727021	0.220756506
Deaths	Global	Both	60-64 years	Prostate cancer	Smoking	Number	2010	1133.318577	1747.847891	526.6446125
Deaths	Global	Both	60-64 years	Prostate cancer	Smoking	Rate	2010	0.474953473	0.732491677	0.220707304
Deaths	Global	Both	60-64 years	Prostate cancer	Smoking	Number	2011	1179.787917	1842.902601	548.0888588
Deaths	Global	Both	60-64 years	Prostate cancer	Smoking	Rate	2011	0.469502785	0.73339275	0.218114834

Deaths	Global	Both	60-64 years	Prostate cancer	Smoking	Number	2012	1191.765585	1845.115363	550.124713 2
Deaths	Global	Both	60-64 years	Prostate cancer	Smoking	Rate	2012	0.457576647	0.708429335	0.211219576
Deaths	Global	Both	60-64 years	Prostate cancer	Smoking	Number	2013	1197.009735	1872.598325	548.257444 4
Deaths	Global	Both	60-64 years	Prostate cancer	Smoking	Rate	2013	0.442398259	0.692086468	0.202628376
Deaths	Global	Both	60-64 years	Prostate cancer	Smoking	Number	2014	1207.462504	1888.492185	553.390065 4
Deaths	Global	Both	60-64 years	Prostate cancer	Smoking	Rate	2014	0.432934237	0.677116614	0.198417346
Deaths	Global	Both	60-64 years	Prostate cancer	Smoking	Number	2015	1227.28407	1920.857557	564.637071 2
Deaths	Global	Both	60-64 years	Prostate cancer	Smoking	Rate	2015	0.42242169	0.661144323	0.194343715
Deaths	Global	Both	60-64 years	Prostate cancer	Smoking	Number	2016	1246.885062	1947.398579	579.694204 1
Deaths	Global	Both	60-64 years	Prostate cancer	Smoking	Rate	2016	0.418267351	0.653254474	0.194458308
Deaths	Global	Both	60-64 years	Prostate cancer	Smoking	Number	2017	1267.629876	1978.626402	576.641884 8

Deaths	Global	Both	60-64 years	Prostate cancer	Smoking	Rate	2017	0.416317969	0.649825111	0.189382077
Deaths	Global	Both	60-64 years	Prostate cancer	Smoking	Number	2018	1282.173529	2013.333437	584.669427
Deaths	Global	Both	60-64 years	Prostate cancer	Smoking	Rate	2018	0.414916767	0.651523199	0.189201495
Deaths	Global	Both	60-64 years	Prostate cancer	Smoking	Number	2019	1297.854242	2024.957707	588.7896341
Deaths	Global	Both	60-64 years	Prostate cancer	Smoking	Rate	2019	0.41556817	0.648384034	0.188528282
Deaths	Global	Both	60-64 years	Prostate cancer	Smoking	Number	2020	1292.955189	2005.336299	580.1874516
Deaths	Global	Both	60-64 years	Prostate cancer	Smoking	Rate	2020	0.409887207	0.635723266	0.183928582
Deaths	Global	Both	60-64 years	Prostate cancer	Smoking	Number	2021	1296.178191	2008.569995	587.8860673
Deaths	Global	Both	60-64 years	Prostate cancer	Smoking	Rate	2021	0.404995121	0.627584273	0.183686927
Deaths	Global	Both	65-69 years	Prostate cancer	Smoking	Number	1990	1627.682453	2535.78578	775.5599459
Deaths	Global	Both	65-69 years	Prostate cancer	Smoking	Rate	1990	1.316794433	2.05144965	0.627427676

Deaths	Global	Both	65-69 years	Prostate cancer	Smoking	Number	1991	1657.218727	2584.394288	789.903647 3
Deaths	Global	Both	65-69 years	Prostate cancer	Smoking	Rate	1991	1.30049862	2.028097529	0.619875088
Deaths	Global	Both	65-69 years	Prostate cancer	Smoking	Number	1992	1694.607525	2631.897216	806.026031
Deaths	Global	Both	65-69 years	Prostate cancer	Smoking	Rate	1992	1.290773819	2.004702546	0.613945874
Deaths	Global	Both	65-69 years	Prostate cancer	Smoking	Number	1993	1733.715144	2702.805047	818.1274501
Deaths	Global	Both	65-69 years	Prostate cancer	Smoking	Rate	1993	1.282885133	1.999975847	0.605384077
Deaths	Global	Both	65-69 years	Prostate cancer	Smoking	Number	1994	1764.364951	2751.948746	840.2104517
Deaths	Global	Both	65-69 years	Prostate cancer	Smoking	Rate	1994	1.270749787	1.982037946	0.605145354
Deaths	Global	Both	65-69 years	Prostate cancer	Smoking	Number	1995	1772.753698	2750.922424	830.7647752
Deaths	Global	Both	65-69 years	Prostate cancer	Smoking	Rate	1995	1.248343953	1.937154257	0.58501087
Deaths	Global	Both	65-69 years	Prostate cancer	Smoking	Number	1996	1771.866526	2752.493656	838.8671185

Deaths	Global	Both	65-69 years	Prostate cancer	Smoking	Rate	1996	1.223125046	1.900055043	0.579072615
Deaths	Global	Both	65-69 years	Prostate cancer	Smoking	Number	1997	1746.12183	2731.932073	826.8196904
Deaths	Global	Both	65-69 years	Prostate cancer	Smoking	Rate	1997	1.188040075	1.858773385	0.56255807
Deaths	Global	Both	65-69 years	Prostate cancer	Smoking	Number	1998	1712.087004	2672.487139	811.653804
Deaths	Global	Both	65-69 years	Prostate cancer	Smoking	Rate	1998	1.15063854	1.796092542	0.54548638
Deaths	Global	Both	65-69 years	Prostate cancer	Smoking	Number	1999	1672.373634	2602.124947	788.898139
Deaths	Global	Both	65-69 years	Prostate cancer	Smoking	Rate	1999	1.111183358	1.72894255	0.52417143
Deaths	Global	Both	65-69 years	Prostate cancer	Smoking	Number	2000	1644.285459	2553.693443	777.987698
Deaths	Global	Both	65-69 years	Prostate cancer	Smoking	Rate	2000	1.077823026	1.673936591	0.50996805
Deaths	Global	Both	65-69 years	Prostate cancer	Smoking	Number	2001	1627.065783	2528.55566	769.356290
Deaths	Global	Both	65-69 years	Prostate cancer	Smoking	Rate	2001	1.048050987	1.628732705	0.49556977

Deaths	Global	Both	65-69 years	Prostate cancer	Smoking	Number	2002	1629.160862	2510.300108	769.1953237
Deaths	Global	Both	65-69 years	Prostate cancer	Smoking	Rate	2002	1.027392714	1.583062852	0.485075286
Deaths	Global	Both	65-69 years	Prostate cancer	Smoking	Number	2003	1632.968559	2516.017592	774.4259352
Deaths	Global	Both	65-69 years	Prostate cancer	Smoking	Rate	2003	1.008863293	1.554419269	0.478447607
Deaths	Global	Both	65-69 years	Prostate cancer	Smoking	Number	2004	1628.617635	2545.086781	765.863486
Deaths	Global	Both	65-69 years	Prostate cancer	Smoking	Rate	2004	0.987679031	1.543473919	0.464459729
Deaths	Global	Both	65-69 years	Prostate cancer	Smoking	Number	2005	1619.946655	2515.80333	761.3098323
Deaths	Global	Both	65-69 years	Prostate cancer	Smoking	Rate	2005	0.965359069	1.499218232	0.453679971
Deaths	Global	Both	65-69 years	Prostate cancer	Smoking	Number	2006	1593.417848	2473.403367	749.0446672
Deaths	Global	Both	65-69 years	Prostate cancer	Smoking	Rate	2006	0.935999226	1.452916848	0.440000864
Deaths	Global	Both	65-69 years	Prostate cancer	Smoking	Number	2007	1554.889324	2397.359327	732.9828878

Deaths	Global	Both	65-69 years	Prostate cancer	Smoking	Rate	2007	0.903335944	1.39278135	0.425837247
Deaths	Global	Both	65-69 years	Prostate cancer	Smoking	Number	2008	1527.741716	2365.008335	721.4371713
Deaths	Global	Both	65-69 years	Prostate cancer	Smoking	Rate	2008	0.878225143	1.359529403	0.414719488
Deaths	Global	Both	65-69 years	Prostate cancer	Smoking	Number	2009	1497.149303	2318.223892	702.296758
Deaths	Global	Both	65-69 years	Prostate cancer	Smoking	Rate	2009	0.850416939	1.316807122	0.398921509
Deaths	Global	Both	65-69 years	Prostate cancer	Smoking	Number	2010	1471.35515	2282.253101	684.9278461
Deaths	Global	Both	65-69 years	Prostate cancer	Smoking	Rate	2010	0.826417703	1.281875668	0.384704194
Deaths	Global	Both	65-69 years	Prostate cancer	Smoking	Number	2011	1458.420638	2274.634729	684.6643099
Deaths	Global	Both	65-69 years	Prostate cancer	Smoking	Rate	2011	0.801970653	1.250798468	0.376489929
Deaths	Global	Both	65-69 years	Prostate cancer	Smoking	Number	2012	1480.924809	2288.992325	690.949039 1
Deaths	Global	Both	65-69 years	Prostate cancer	Smoking	Rate	2012	0.778431826	1.203183622	0.363189758

Deaths	Global	Both	65-69 years	Prostate cancer	Smoking	Number	2013	1506.697738	2369.699237	705.5381874
Deaths	Global	Both	65-69 years	Prostate cancer	Smoking	Rate	2013	0.756710304	1.190136406	0.354343146
Deaths	Global	Both	65-69 years	Prostate cancer	Smoking	Number	2014	1573.039941	2466.30453	736.4451771
Deaths	Global	Both	65-69 years	Prostate cancer	Smoking	Rate	2014	0.745053845	1.168139236	0.348809523
Deaths	Global	Both	65-69 years	Prostate cancer	Smoking	Number	2015	1638.701014	2554.756955	771.3656529
Deaths	Global	Both	65-69 years	Prostate cancer	Smoking	Rate	2015	0.741587472	1.156144859	0.349078386
Deaths	Global	Both	65-69 years	Prostate cancer	Smoking	Number	2016	1722.24541	2691.13067	808.8614989
Deaths	Global	Both	65-69 years	Prostate cancer	Smoking	Rate	2016	0.739107532	1.154907969	0.34712569
Deaths	Global	Both	65-69 years	Prostate cancer	Smoking	Number	2017	1773.921443	2776.791174	816.5463235
Deaths	Global	Both	65-69 years	Prostate cancer	Smoking	Rate	2017	0.733852414	1.148728944	0.33779652
Deaths	Global	Both	65-69 years	Prostate cancer	Smoking	Number	2018	1815.91913	2858.885773	837.080064

Deaths	Global	Both	65-69 years	Prostate cancer	Smoking	Rate	2018	0.722774178	1.137896936	0.33317555
Deaths	Global	Both	65-69 years	Prostate cancer	Smoking	Number	2019	1847.339427	2896.283051	860.321357 4
Deaths	Global	Both	65-69 years	Prostate cancer	Smoking	Rate	2019	0.713152005	1.118089094	0.332120829
Deaths	Global	Both	65-69 years	Prostate cancer	Smoking	Number	2020	1881.26537	2943.654073	864.122560 3
Deaths	Global	Both	65-69 years	Prostate cancer	Smoking	Rate	2020	0.697909129	1.092032567	0.320570948
Deaths	Global	Both	65-69 years	Prostate cancer	Smoking	Number	2021	1900.157632	2954.78417	877.265073 5
Deaths	Global	Both	65-69 years	Prostate cancer	Smoking	Rate	2021	0.68885686	1.071186575	0.318031543
Deaths	Global	Both	70-74 years	Prostate cancer	Smoking	Number	1990	1745.84968	2774.0956	821.862233 3
Deaths	Global	Both	70-74 years	Prostate cancer	Smoking	Rate	1990	2.062161436	3.276704192	0.970766626
Deaths	Global	Both	70-74 years	Prostate cancer	Smoking	Number	1991	1808.615042	2871.352272	853.309866 2
Deaths	Global	Both	70-74 years	Prostate cancer	Smoking	Rate	1991	2.061585806	3.272967963	0.972662213

Deaths	Global	Both	70-74 years	Prostate cancer	Smoking	Number	1992	1888.507768	2992.87161	891.3051199
Deaths	Global	Both	70-74 years	Prostate cancer	Smoking	Rate	1992	2.066091143	3.274302405	0.975117839
Deaths	Global	Both	70-74 years	Prostate cancer	Smoking	Number	1993	1971.252016	3118.288729	937.8220681
Deaths	Global	Both	70-74 years	Prostate cancer	Smoking	Rate	1993	2.066518214	3.268988644	0.98314491
Deaths	Global	Both	70-74 years	Prostate cancer	Smoking	Number	1994	2043.225042	3233.252047	967.2463939
Deaths	Global	Both	70-74 years	Prostate cancer	Smoking	Rate	1994	2.059544509	3.259076393	0.974971899
Deaths	Global	Both	70-74 years	Prostate cancer	Smoking	Number	1995	2064.56644	3280.97188	973.9159283
Deaths	Global	Both	70-74 years	Prostate cancer	Smoking	Rate	1995	2.010415369	3.194915973	0.948371296
Deaths	Global	Both	70-74 years	Prostate cancer	Smoking	Number	1996	2068.592814	3291.74334	981.8800542
Deaths	Global	Both	70-74 years	Prostate cancer	Smoking	Rate	1996	1.952050316	3.106289737	0.926561891
Deaths	Global	Both	70-74 years	Prostate cancer	Smoking	Number	1997	2062.75773	3270.170935	980.0153189

Deaths	Global	Both	70-74 years	Prostate cancer	Smoking	Rate	1997	1.886484973	2.990718802	0.896268207
Deaths	Global	Both	70-74 years	Prostate cancer	Smoking	Number	1998	2078.987705	3313.580862	990.0931937
Deaths	Global	Both	70-74 years	Prostate cancer	Smoking	Rate	1998	1.843566949	2.938357041	0.877976856
Deaths	Global	Both	70-74 years	Prostate cancer	Smoking	Number	1999	2091.944082	3312.443923	992.2475711
Deaths	Global	Both	70-74 years	Prostate cancer	Smoking	Rate	1999	1.801324387	2.85226841	0.854401303
Deaths	Global	Both	70-74 years	Prostate cancer	Smoking	Number	2000	2095.101112	3334.976741	995.2154227
Deaths	Global	Both	70-74 years	Prostate cancer	Smoking	Rate	2000	1.758845721	2.799726245	0.835487308
Deaths	Global	Both	70-74 years	Prostate cancer	Smoking	Number	2001	2102.650446	3333.924634	992.7742237
Deaths	Global	Both	70-74 years	Prostate cancer	Smoking	Rate	2001	1.725247751	2.735521726	0.814582138
Deaths	Global	Both	70-74 years	Prostate cancer	Smoking	Number	2002	2089.566892	3320.292677	991.261026
Deaths	Global	Both	70-74 years	Prostate cancer	Smoking	Rate	2002	1.685092086	2.677587847	0.79938389

Deaths	Global	Both	70-74 years	Prostate cancer	Smoking	Number	2003	2056.023632	3249.259835	974.4468283
Deaths	Global	Both	70-74 years	Prostate cancer	Smoking	Rate	2003	1.632934919	2.580626877	0.773925079
Deaths	Global	Both	70-74 years	Prostate cancer	Smoking	Number	2004	2006.403206	3165.973234	945.3614037
Deaths	Global	Both	70-74 years	Prostate cancer	Smoking	Rate	2004	1.570099812	2.477514969	0.739787375
Deaths	Global	Both	70-74 years	Prostate cancer	Smoking	Number	2005	1958.705413	3103.336668	920.1611375
Deaths	Global	Both	70-74 years	Prostate cancer	Smoking	Rate	2005	1.506498239	2.386867977	0.707723134
Deaths	Global	Both	70-74 years	Prostate cancer	Smoking	Number	2006	1912.884551	3022.36743	898.4959566
Deaths	Global	Both	70-74 years	Prostate cancer	Smoking	Rate	2006	1.439820232	2.274923372	0.676294163
Deaths	Global	Both	70-74 years	Prostate cancer	Smoking	Number	2007	1902.023803	2999.029341	887.8097289
Deaths	Global	Both	70-74 years	Prostate cancer	Smoking	Rate	2007	1.395811071	2.200854874	0.651524259
Deaths	Global	Both	70-74 years	Prostate cancer	Smoking	Number	2008	1911.121599	3011.356999	897.9953311

Deaths	Global	Both	70-74 years	Prostate cancer	Smoking	Rate	2008	1.368759493	2.156756263	0.64315093
Deaths	Global	Both	70-74 years	Prostate cancer	Smoking	Number	2009	1919.013156	3029.453184	901.511047 3
Deaths	Global	Both	70-74 years	Prostate cancer	Smoking	Rate	2009	1.344518589	2.122526418	0.631625874
Deaths	Global	Both	70-74 years	Prostate cancer	Smoking	Number	2010	1929.233068	3052.200404	898.1639775
Deaths	Global	Both	70-74 years	Prostate cancer	Smoking	Rate	2010	1.323868855	2.094465993	0.61633368
Deaths	Global	Both	70-74 years	Prostate cancer	Smoking	Number	2011	1922.703795	3068.078343	893.188045
Deaths	Global	Both	70-74 years	Prostate cancer	Smoking	Rate	2011	1.296490874	2.068823904	0.602282136
Deaths	Global	Both	70-74 years	Prostate cancer	Smoking	Number	2012	1893.951695	3009.680124	882.2426396
Deaths	Global	Both	70-74 years	Prostate cancer	Smoking	Rate	2012	1.259747523	2.001865777	0.586816962
Deaths	Global	Both	70-74 years	Prostate cancer	Smoking	Number	2013	1852.843541	2925.946514	861.857903
Deaths	Global	Both	70-74 years	Prostate cancer	Smoking	Rate	2013	1.216027074	1.920307947	0.565640066

Deaths	Global	Both	70-74 years	Prostate cancer	Smoking	Number	2014	1847.629407	2958.323168	847.682317 5
Deaths	Global	Both	70-74 years	Prostate cancer	Smoking	Rate	2014	1.194589096	1.912710734	0.548070976
Deaths	Global	Both	70-74 years	Prostate cancer	Smoking	Number	2015	1837.921506	2925.047693	845.859916 4
Deaths	Global	Both	70-74 years	Prostate cancer	Smoking	Rate	2015	1.171682581	1.864730034	0.539239204
Deaths	Global	Both	70-74 years	Prostate cancer	Smoking	Number	2016	1859.930742	2988.953295	850.055236 3
Deaths	Global	Both	70-74 years	Prostate cancer	Smoking	Rate	2016	1.157903916	1.860779355	0.529203731
Deaths	Global	Both	70-74 years	Prostate cancer	Smoking	Number	2017	1934.105221	3085.121732	884.474733 2
Deaths	Global	Both	70-74 years	Prostate cancer	Smoking	Rate	2017	1.147797168	1.830869359	0.524892639
Deaths	Global	Both	70-74 years	Prostate cancer	Smoking	Number	2018	2019.287966	3272.756973	933.407634 5
Deaths	Global	Both	70-74 years	Prostate cancer	Smoking	Rate	2018	1.14306242	1.852616154	0.528375946
Deaths	Global	Both	70-74 years	Prostate cancer	Smoking	Number	2019	2127.273116	3448.159315	981.691255 7

Deaths	Global	Both	70-74 years	Prostate cancer	Smoking	Rate	2019	1.13412516	1.838336697	0.523374617
Deaths	Global	Both	70-74 years	Prostate cancer	Smoking	Number	2020	2219.631303	3580.872672	1024.729863
Deaths	Global	Both	70-74 years	Prostate cancer	Smoking	Rate	2020	1.132197941	1.826545093	0.522698089
Deaths	Global	Both	70-74 years	Prostate cancer	Smoking	Number	2021	2312.737771	3764.206917	1076.575665
Deaths	Global	Both	70-74 years	Prostate cancer	Smoking	Rate	2021	1.123565162	1.828712191	0.523017753
Deaths	Global	Both	75-79 years	Prostate cancer	Smoking	Number	1990	2114.522619	3447.714396	996.8209311
Deaths	Global	Both	75-79 years	Prostate cancer	Smoking	Rate	1990	3.435147348	5.600983815	1.619385268
Deaths	Global	Both	75-79 years	Prostate cancer	Smoking	Number	1991	2101.620878	3437.196265	993.8583942
Deaths	Global	Both	75-79 years	Prostate cancer	Smoking	Rate	1991	3.402255903	5.564381952	1.608929862
Deaths	Global	Both	75-79 years	Prostate cancer	Smoking	Number	1992	2066.090594	3395.165879	977.4326802
Deaths	Global	Both	75-79 years	Prostate cancer	Smoking	Rate	1992	3.345796028	5.49808055	1.582839779

Deaths	Global	Both	75-79 years	Prostate cancer	Smoking	Number	1993	2010.015332	3299.896743	951.0472942
Deaths	Global	Both	75-79 years	Prostate cancer	Smoking	Rate	1993	3.259725971	5.351580628	1.542353192
Deaths	Global	Both	75-79 years	Prostate cancer	Smoking	Number	1994	1973.144536	3236.74116	930.4112071
Deaths	Global	Both	75-79 years	Prostate cancer	Smoking	Rate	1994	3.171960356	5.203275509	1.495697558
Deaths	Global	Both	75-79 years	Prostate cancer	Smoking	Number	1995	1975.191386	3227.480393	930.1146622
Deaths	Global	Both	75-79 years	Prostate cancer	Smoking	Rate	1995	3.095609949	5.058254348	1.457718084
Deaths	Global	Both	75-79 years	Prostate cancer	Smoking	Number	1996	2014.28804	3304.651578	945.5326881
Deaths	Global	Both	75-79 years	Prostate cancer	Smoking	Rate	1996	3.036837995	4.982252424	1.425530776
Deaths	Global	Both	75-79 years	Prostate cancer	Smoking	Number	1997	2062.235439	3380.188801	965.3890045
Deaths	Global	Both	75-79 years	Prostate cancer	Smoking	Rate	1997	2.97356062	4.87393249	1.392005332
Deaths	Global	Both	75-79 years	Prostate cancer	Smoking	Number	1998	2129.45304	3502.963507	1000.748753

Deaths	Global	Both	75-79 years	Prostate cancer	Smoking	Rate	1998	2.931564243	4.822441429	1.377705544
Deaths	Global	Both	75-79 years	Prostate cancer	Smoking	Number	1999	2187.396029	3614.501735	1030.828686
Deaths	Global	Both	75-79 years	Prostate cancer	Smoking	Rate	1999	2.884780328	4.766875025	1.359476874
Deaths	Global	Both	75-79 years	Prostate cancer	Smoking	Number	2000	2212.626831	3648.0709	1045.513282
Deaths	Global	Both	75-79 years	Prostate cancer	Smoking	Rate	2000	2.808596832	4.630677089	1.327121795
Deaths	Global	Both	75-79 years	Prostate cancer	Smoking	Number	2001	2232.434086	3672.687262	1039.237698
Deaths	Global	Both	75-79 years	Prostate cancer	Smoking	Rate	2001	2.73678145	4.502413949	1.274020349
Deaths	Global	Both	75-79 years	Prostate cancer	Smoking	Number	2002	2246.866889	3698.385666	1048.169977
Deaths	Global	Both	75-79 years	Prostate cancer	Smoking	Rate	2002	2.661095858	4.380214432	1.241409003
Deaths	Global	Both	75-79 years	Prostate cancer	Smoking	Number	2003	2269.610566	3746.30335	1060.436431
Deaths	Global	Both	75-79 years	Prostate cancer	Smoking	Rate	2003	2.598320592	4.288884305	1.214020527

Deaths	Global	Both	75-79 years	Prostate cancer	Smoking	Number	2004	2276.102661	3771.139294	1054.618153
Deaths	Global	Both	75-79 years	Prostate cancer	Smoking	Rate	2004	2.522433538	4.179270292	1.168754048
Deaths	Global	Both	75-79 years	Prostate cancer	Smoking	Number	2005	2264.654354	3720.667767	1052.875149
Deaths	Global	Both	75-79 years	Prostate cancer	Smoking	Rate	2005	2.43922384	4.00747315	1.134035382
Deaths	Global	Both	75-79 years	Prostate cancer	Smoking	Number	2006	2252.506176	3705.428468	1035.527775
Deaths	Global	Both	75-79 years	Prostate cancer	Smoking	Rate	2006	2.361557541	3.884820665	1.085661141
Deaths	Global	Both	75-79 years	Prostate cancer	Smoking	Number	2007	2230.323872	3659.692061	1025.034982
Deaths	Global	Both	75-79 years	Prostate cancer	Smoking	Rate	2007	2.286391153	3.751691697	1.050802955
Deaths	Global	Both	75-79 years	Prostate cancer	Smoking	Number	2008	2206.663531	3640.748742	1016.442356
Deaths	Global	Both	75-79 years	Prostate cancer	Smoking	Rate	2008	2.215727211	3.655702802	1.020617305
Deaths	Global	Both	75-79 years	Prostate cancer	Smoking	Number	2009	2178.235975	3613.383118	1002.321874

Deaths	Global	Both	75-79 years	Prostate cancer	Smoking	Rate	2009	2.142643666 9	3.554340553	0.985943966
Deaths	Global	Both	75-79 years	Prostate cancer	Smoking	Number	2010	2159.61094	3574.217622	989.2125131
Deaths	Global	Both	75-79 years	Prostate cancer	Smoking	Rate	2010	2.076116878	3.436032573	0.950967953
Deaths	Global	Both	75-79 years	Prostate cancer	Smoking	Number	2011	2143.733978	3536.072665	978.5741944
Deaths	Global	Both	75-79 years	Prostate cancer	Smoking	Rate	2011	2.007586672	3.311498734	0.916425513
Deaths	Global	Both	75-79 years	Prostate cancer	Smoking	Number	2012	2139.369911	3506.812386	976.7399585
Deaths	Global	Both	75-79 years	Prostate cancer	Smoking	Rate	2012	1.945902739	3.189684865	0.88841156
Deaths	Global	Both	75-79 years	Prostate cancer	Smoking	Number	2013	2151.557058	3547.002554	982.4391902
Deaths	Global	Both	75-79 years	Prostate cancer	Smoking	Rate	2013	1.902939415	3.137137794	0.868915956
Deaths	Global	Both	75-79 years	Prostate cancer	Smoking	Number	2014	2188.176796	3542.45519	998.6604909
Deaths	Global	Both	75-79 years	Prostate cancer	Smoking	Rate	2014	1.887113569	3.055061761	0.861258453

Deaths	Global	Both	75-79 years	Prostate cancer	Smoking	Number	2015	2219.017257	3657.93799	1020.71114
Deaths	Global	Both	75-79 years	Prostate cancer	Smoking	Rate	2015	1.86817326	3.079589364	0.85932872
Deaths	Global	Both	75-79 years	Prostate cancer	Smoking	Number	2016	2253.839696	3746.08532	1043.23291
Deaths	Global	Both	75-79 years	Prostate cancer	Smoking	Rate	2016	1.857755986	3.087758388	0.859897972
Deaths	Global	Both	75-79 years	Prostate cancer	Smoking	Number	2017	2262.012808	3781.082752	1021.026638
Deaths	Global	Both	75-79 years	Prostate cancer	Smoking	Rate	2017	1.83283048	3.06368014	0.827302453
Deaths	Global	Both	75-79 years	Prostate cancer	Smoking	Number	2018	2268.869114	3810.279079	1041.501074
Deaths	Global	Both	75-79 years	Prostate cancer	Smoking	Rate	2018	1.808254179	3.03673448	0.830060517
Deaths	Global	Both	75-79 years	Prostate cancer	Smoking	Number	2019	2275.955044	3743.668894	1033.899625
Deaths	Global	Both	75-79 years	Prostate cancer	Smoking	Rate	2019	1.782688997	2.932306313	0.809823326
Deaths	Global	Both	75-79 years	Prostate cancer	Smoking	Number	2020	2267.685067	3724.020912	1048.883033

Deaths	Global	Both	75-79 years	Prostate cancer	Smoking	Rate	2020	1.752997862	2.878795117	0.810822341
Deaths	Global	Both	75-79 years	Prostate cancer	Smoking	Number	2021	2278.059498	3793.6749	1038.907173
Deaths	Global	Both	75-79 years	Prostate cancer	Smoking	Rate	2021	1.727315299	2.876515166	0.787740731
Deaths	Global	Both	80-84 years	Prostate cancer	Smoking	Number	1990	1435.610548	2414.823952	648.8479163
Deaths	Global	Both	80-84 years	Prostate cancer	Smoking	Rate	1990	4.058156669	6.826178546	1.834150983
Deaths	Global	Both	80-84 years	Prostate cancer	Smoking	Number	1991	1482.537535	2500.897045	671.599036
Deaths	Global	Both	80-84 years	Prostate cancer	Smoking	Rate	1991	4.06269867	6.853378657	1.840430998
Deaths	Global	Both	80-84 years	Prostate cancer	Smoking	Number	1992	1528.079301	2585.808875	692.6468993
Deaths	Global	Both	80-84 years	Prostate cancer	Smoking	Rate	1992	4.074116673	6.894201787	1.846713242
Deaths	Global	Both	80-84 years	Prostate cancer	Smoking	Number	1993	1569.849076	2676.350933	707.0609614
Deaths	Global	Both	80-84 years	Prostate cancer	Smoking	Rate	1993	4.070053162	6.938813893	1.833154375

Deaths	Global	Both	80-84 years	Prostate cancer	Smoking	Number	1994	1607.289265	2747.509042	726.789749 3
Deaths	Global	Both	80-84 years	Prostate cancer	Smoking	Rate	1994	4.074698307	6.965311526	1.842511504
Deaths	Global	Both	80-84 years	Prostate cancer	Smoking	Number	1995	1612.137144	2754.178113	728.122952 7
Deaths	Global	Both	80-84 years	Prostate cancer	Smoking	Rate	1995	4.022269247	6.871652305	1.816660928
Deaths	Global	Both	80-84 years	Prostate cancer	Smoking	Number	1996	1587.694651	2713.166499	719.788252
Deaths	Global	Both	80-84 years	Prostate cancer	Smoking	Rate	1996	3.942966779	6.738024449	1.787561081
Deaths	Global	Both	80-84 years	Prostate cancer	Smoking	Number	1997	1537.78112	2617.126005	688.956171 6
Deaths	Global	Both	80-84 years	Prostate cancer	Smoking	Rate	1997	3.811458701	6.486662864	1.707608424
Deaths	Global	Both	80-84 years	Prostate cancer	Smoking	Number	1998	1494.577469	2553.2411	669.074863 6
Deaths	Global	Both	80-84 years	Prostate cancer	Smoking	Rate	1998	3.694598341	6.311616847	1.653954333
Deaths	Global	Both	80-84 years	Prostate cancer	Smoking	Number	1999	1456.815286	2486.450239	653.770995 4

Deaths	Global	Both	80-84 years	Prostate cancer	Smoking	Rate	1999	3.54962542	6.05839811	1.592955653
Deaths	Global	Both	80-84 years	Prostate cancer	Smoking	Number	2000	1463.647206	2503.864193	656.519185 2
Deaths	Global	Both	80-84 years	Prostate cancer	Smoking	Rate	2000	3.448644132	5.899602392	1.54688987
Deaths	Global	Both	80-84 years	Prostate cancer	Smoking	Number	2001	1504.500499	2569.614803	673.988992 2
Deaths	Global	Both	80-84 years	Prostate cancer	Smoking	Rate	2001	3.383124085	5.778213923	1.51557835 6
Deaths	Global	Both	80-84 years	Prostate cancer	Smoking	Number	2002	1552.147051	2664.247263	693.372277 1
Deaths	Global	Both	80-84 years	Prostate cancer	Smoking	Rate	2002	3.318361278	5.695939019	1.48237222 49
Deaths	Global	Both	80-84 years	Prostate cancer	Smoking	Number	2003	1603.592078	2750.425433	709.200762 3
Deaths	Global	Both	80-84 years	Prostate cancer	Smoking	Rate	2003	3.260221205	5.591818172	1.44185756 2
Deaths	Global	Both	80-84 years	Prostate cancer	Smoking	Number	2004	1648.148695	2849.730012	725.027487 8
Deaths	Global	Both	80-84 years	Prostate cancer	Smoking	Rate	2004	3.198908462	5.531069787	1.40721318

Deaths	Global	Both	80-84 years	Prostate cancer	Smoking	Number	2005	1666.476054	2872.37961	735.199153 3
Deaths	Global	Both	80-84 years	Prostate cancer	Smoking	Rate	2005	3.101187612	5.345284167	1.368150776
Deaths	Global	Both	80-84 years	Prostate cancer	Smoking	Number	2006	1678.989126	2884.097216	738.5813639
Deaths	Global	Both	80-84 years	Prostate cancer	Smoking	Rate	2006	3.004275527	5.160618702	1.321570153
Deaths	Global	Both	80-84 years	Prostate cancer	Smoking	Number	2007	1698.875688	2916.715537	748.2583343
Deaths	Global	Both	80-84 years	Prostate cancer	Smoking	Rate	2007	2.921374395	5.015563028	1.286699642
Deaths	Global	Both	80-84 years	Prostate cancer	Smoking	Number	2008	1719.149532	2943.204391	758.3401668
Deaths	Global	Both	80-84 years	Prostate cancer	Smoking	Rate	2008	2.843262854	4.867699734	1.254201794
Deaths	Global	Both	80-84 years	Prostate cancer	Smoking	Number	2009	1725.654739	2974.353888	761.8195913
Deaths	Global	Both	80-84 years	Prostate cancer	Smoking	Rate	2009	2.750456393	4.740711153	1.214235686
Deaths	Global	Both	80-84 years	Prostate cancer	Smoking	Number	2010	1736.547342	2983.157419	765.7952768

Deaths	Global	Both	80-84 years	Prostate cancer	Smoking	Rate	2010	2.678613838	4.60150239	1.181234612
Deaths	Global	Both	80-84 years	Prostate cancer	Smoking	Number	2011	1753.64315	3012.509487	772.7496278
Deaths	Global	Both	80-84 years	Prostate cancer	Smoking	Rate	2011	2.621677927	4.503669762	1.155252505
Deaths	Global	Both	80-84 years	Prostate cancer	Smoking	Number	2012	1741.975428	2970.424678	764.0489338
Deaths	Global	Both	80-84 years	Prostate cancer	Smoking	Rate	2012	2.53600811	4.324412934	1.112320106
Deaths	Global	Both	80-84 years	Prostate cancer	Smoking	Number	2013	1710.382948	2938.116783	746.4283105
Deaths	Global	Both	80-84 years	Prostate cancer	Smoking	Rate	2013	2.427974861	4.170804964	1.059592635
Deaths	Global	Both	80-84 years	Prostate cancer	Smoking	Number	2014	1718.525746	2977.22326	750.5339117
Deaths	Global	Both	80-84 years	Prostate cancer	Smoking	Rate	2014	2.379701845	4.122663685	1.039290181
Deaths	Global	Both	80-84 years	Prostate cancer	Smoking	Number	2015	1756.508871	3019.447259	760.486672
Deaths	Global	Both	80-84 years	Prostate cancer	Smoking	Rate	2015	2.366257229	4.067607641	1.024479361

Deaths	Global	Both	80-84 years	Prostate cancer	Smoking	Number	2016	1799.331973	3099.838321	786.514928 5
Deaths	Global	Both	80-84 years	Prostate cancer	Smoking	Rate	2016	2.349702562	4.048001234	1.027090148
Deaths	Global	Both	80-84 years	Prostate cancer	Smoking	Number	2017	1810.120382	3126.094514	788.913768 8
Deaths	Global	Both	80-84 years	Prostate cancer	Smoking	Rate	2017	2.285338287	3.946800196	0.996030351
Deaths	Global	Both	80-84 years	Prostate cancer	Smoking	Number	2018	1852.416286	3209.178814	805.753892 6
Deaths	Global	Both	80-84 years	Prostate cancer	Smoking	Rate	2018	2.264706644	3.923442383	0.985089693
Deaths	Global	Both	80-84 years	Prostate cancer	Smoking	Number	2019	1903.588471	3292.716577	834.358397 6
Deaths	Global	Both	80-84 years	Prostate cancer	Smoking	Rate	2019	2.260872244	3.91072526	0.990958798
Deaths	Global	Both	80-84 years	Prostate cancer	Smoking	Number	2020	1915.346793	3324.481899	850.587715 7
Deaths	Global	Both	80-84 years	Prostate cancer	Smoking	Rate	2020	2.220871026	3.854782619	0.986268189
Deaths	Global	Both	80-84 years	Prostate cancer	Smoking	Number	2021	1913.154834	3447.661078	845.021229 3

Deaths	Global	Both	80-84 years	Prostate cancer	Smoking	Rate	2021	2.184389198	3.936447527	0.964822717
Deaths	Global	Both	85-89 years	Prostate cancer	Smoking	Number	1990	780.3765577	1335.772144	350.2122022
Deaths	Global	Both	85-89 years	Prostate cancer	Smoking	Rate	1990	5.164269826	8.839691185	2.31758667
Deaths	Global	Both	85-89 years	Prostate cancer	Smoking	Number	1991	822.3682909	1411.29819	367.706054
Deaths	Global	Both	85-89 years	Prostate cancer	Smoking	Rate	1991	5.202912942	8.92892114	2.326381754
Deaths	Global	Both	85-89 years	Prostate cancer	Smoking	Number	1992	868.0939482	1502.210333	387.7735944
Deaths	Global	Both	85-89 years	Prostate cancer	Smoking	Rate	1992	5.240681062	9.068840139	2.34098825
Deaths	Global	Both	85-89 years	Prostate cancer	Smoking	Number	1993	909.8337858	1585.956759	406.2528428
Deaths	Global	Both	85-89 years	Prostate cancer	Smoking	Rate	1993	5.286766386	9.215510584	2.360611253
Deaths	Global	Both	85-89 years	Prostate cancer	Smoking	Number	1994	949.2699434	1640.986496	423.5248163
Deaths	Global	Both	85-89 years	Prostate cancer	Smoking	Rate	1994	5.321026512	9.198366296	2.374020996

Deaths	Global	Both	85-89 years	Prostate cancer	Smoking	Number	1995	977.3074355	1679.194799	433.7085158
Deaths	Global	Both	85-89 years	Prostate cancer	Smoking	Rate	1995	5.304795231	9.114618633	2.35415672
Deaths	Global	Both	85-89 years	Prostate cancer	Smoking	Number	1996	1005.702157	1728.806612	444.7571812
Deaths	Global	Both	85-89 years	Prostate cancer	Smoking	Rate	1996	5.281005841	9.078073217	2.335448179
Deaths	Global	Both	85-89 years	Prostate cancer	Smoking	Number	1997	1021.664545	1750.995793	451.1038559
Deaths	Global	Both	85-89 years	Prostate cancer	Smoking	Rate	1997	5.198635608	8.909763117	2.295395862
Deaths	Global	Both	85-89 years	Prostate cancer	Smoking	Number	1998	1040.454526	1790.173688	456.9217853
Deaths	Global	Both	85-89 years	Prostate cancer	Smoking	Rate	1998	5.120401719	8.810003894	2.248654829
Deaths	Global	Both	85-89 years	Prostate cancer	Smoking	Number	1999	1052.60974	1818.333345	461.6041042
Deaths	Global	Both	85-89 years	Prostate cancer	Smoking	Rate	1999	5.032886836	8.694073036	2.207086949
Deaths	Global	Both	85-89 years	Prostate cancer	Smoking	Number	2000	1060.007346	1830.305625	465.0833626

Deaths	Global	Both	85-89 years	Prostate cancer	Smoking	Rate	2000	4.95477986	8.555376039	2.173933688
Deaths	Global	Both	85-89 years	Prostate cancer	Smoking	Number	2001	1058.320308	1833.057704	462.5246576
Deaths	Global	Both	85-89 years	Prostate cancer	Smoking	Rate	2001	4.890017767	8.469727611	2.1371165
Deaths	Global	Both	85-89 years	Prostate cancer	Smoking	Number	2002	1031.732208	1782.872395	448.9113341
Deaths	Global	Both	85-89 years	Prostate cancer	Smoking	Rate	2002	4.730232238	8.174020757	2.058145368
Deaths	Global	Both	85-89 years	Prostate cancer	Smoking	Number	2003	1000.527975	1732.365582	434.6247715
Deaths	Global	Both	85-89 years	Prostate cancer	Smoking	Rate	2003	4.551307046	7.880367039	1.977066942
Deaths	Global	Both	85-89 years	Prostate cancer	Smoking	Number	2004	977.5827303	1699.599148	422.7515461
Deaths	Global	Both	85-89 years	Prostate cancer	Smoking	Rate	2004	4.357673963	7.57613522	1.884457804
Deaths	Global	Both	85-89 years	Prostate cancer	Smoking	Number	2005	985.9016809	1717.886346	429.0518795
Deaths	Global	Both	85-89 years	Prostate cancer	Smoking	Rate	2005	4.213651134	7.342084804	1.833727413

Deaths	Global	Both	85-89 years	Prostate cancer	Smoking	Number	2006	1013.167875	1759.105973	437.765792
Deaths	Global	Both	85-89 years	Prostate cancer	Smoking	Rate	2006	4.093351683	7.107054593	1.76864011
Deaths	Global	Both	85-89 years	Prostate cancer	Smoking	Number	2007	1057.387937	1835.177183	454.6456626
Deaths	Global	Both	85-89 years	Prostate cancer	Smoking	Rate	2007	4.021703668	6.979972587	1.7292141
Deaths	Global	Both	85-89 years	Prostate cancer	Smoking	Number	2008	1096.526325	1911.046018	473.707738
Deaths	Global	Both	85-89 years	Prostate cancer	Smoking	Rate	2008	3.93255934	6.853735925	1.698895637
Deaths	Global	Both	85-89 years	Prostate cancer	Smoking	Number	2009	1123.708728	1964.615323	488.9469669
Deaths	Global	Both	85-89 years	Prostate cancer	Smoking	Rate	2009	3.816429359	6.672383518	1.660600752
Deaths	Global	Both	85-89 years	Prostate cancer	Smoking	Number	2010	1147.49396	2022.409193	495.5530893
Deaths	Global	Both	85-89 years	Prostate cancer	Smoking	Rate	2010	3.70945023	6.537747914	1.601951369
Deaths	Global	Both	85-89 years	Prostate cancer	Smoking	Number	2011	1170.358873	2041.797431	504.9271856

Deaths	Global	Both	85-89 years	Prostate cancer	Smoking	Rate	2011	3.621527728	6.31808429	1.562433408
Deaths	Global	Both	85-89 years	Prostate cancer	Smoking	Number	2012	1190.583047	2067.11994	510.3492835
Deaths	Global	Both	85-89 years	Prostate cancer	Smoking	Rate	2012	3.523232584	6.117124166	1.51025099
Deaths	Global	Both	85-89 years	Prostate cancer	Smoking	Number	2013	1207.817158	2111.091434	514.2159147
Deaths	Global	Both	85-89 years	Prostate cancer	Smoking	Rate	2013	3.421947741	5.981074632	1.456859572
Deaths	Global	Both	85-89 years	Prostate cancer	Smoking	Number	2014	1249.838418	2180.551524	529.41323 8
Deaths	Global	Both	85-89 years	Prostate cancer	Smoking	Rate	2014	3.40196442	5.935294191	1.441022274
Deaths	Global	Both	85-89 years	Prostate cancer	Smoking	Number	2015	1318.463408	2278.893288	563.4022752
Deaths	Global	Both	85-89 years	Prostate cancer	Smoking	Rate	2015	3.462967431	5.985553477	1.479786028
Deaths	Global	Both	85-89 years	Prostate cancer	Smoking	Number	2016	1386.195419	2406.932527	596.6569081
Deaths	Global	Both	85-89 years	Prostate cancer	Smoking	Rate	2016	3.513907113	6.101403318	1.512482963

Deaths	Global	Both	85-89 years	Prostate cancer	Smoking	Number	2017	1383.331985	2438.923658	593.8160694
Deaths	Global	Both	85-89 years	Prostate cancer	Smoking	Rate	2017	3.401104697	5.99641648	1.459975368
Deaths	Global	Both	85-89 years	Prostate cancer	Smoking	Number	2018	1403.0248	2470.594626	604.0041349
Deaths	Global	Both	85-89 years	Prostate cancer	Smoking	Rate	2018	3.346683105	5.893193973	1.440751748
Deaths	Global	Both	85-89 years	Prostate cancer	Smoking	Number	2019	1445.537868	2530.072952	619.9549267
Deaths	Global	Both	85-89 years	Prostate cancer	Smoking	Rate	2019	3.340134102	5.846116615	1.432499721
Deaths	Global	Both	85-89 years	Prostate cancer	Smoking	Number	2020	1457.565428	2574.1063	623.3549998
Deaths	Global	Both	85-89 years	Prostate cancer	Smoking	Rate	2020	3.268598812	5.772448104	1.397877153
Deaths	Global	Both	85-89 years	Prostate cancer	Smoking	Number	2021	1454.81606	2669.547662	620.3125084
Deaths	Global	Both	85-89 years	Prostate cancer	Smoking	Rate	2021	3.181887731	5.83867692	1.356710868
Deaths	Global	Both	90-94 years	Prostate cancer	Smoking	Number	1990	256.673458	453.161864	110.894676

Deaths	Global	Both	90-94 years	Prostate cancer	Smoking	Rate	1990	5.989783526	10.57507655	2.5878605
Deaths	Global	Both	90-94 years	Prostate cancer	Smoking	Number	1991	275.6221402	485.7503242	118.813783
Deaths	Global	Both	90-94 years	Prostate cancer	Smoking	Rate	1991	6.02212013	10.61325046	2.597461658
Deaths	Global	Both	90-94 years	Prostate cancer	Smoking	Number	1992	296.884438	523.763455	127.6275375
Deaths	Global	Both	90-94 years	Prostate cancer	Smoking	Rate	1992	6.129209395	10.81314976	2.634883483
Deaths	Global	Both	90-94 years	Prostate cancer	Smoking	Number	1993	319.4592328	568.9252983	137.0410824
Deaths	Global	Both	90-94 years	Prostate cancer	Smoking	Rate	1993	6.197615158	11.03733964	2.658642489
Deaths	Global	Both	90-94 years	Prostate cancer	Smoking	Number	1994	341.9777583	607.362057	144.8956025
Deaths	Global	Both	90-94 years	Prostate cancer	Smoking	Rate	1994	6.247567996	11.09585538	2.647087733
Deaths	Global	Both	90-94 years	Prostate cancer	Smoking	Number	1995	362.5832685	643.6467365	154.2434881
Deaths	Global	Both	90-94 years	Prostate cancer	Smoking	Rate	1995	6.264914485	11.12128472	2.665104397

Deaths	Global	Both	90-94 years	Prostate cancer	Smoking	Number	1996	381.5494868	675.6289459	161.584264 6
Deaths	Global	Both	90-94 years	Prostate cancer	Smoking	Rate	1996	6.273993166	11.10967656	2.657004155
Deaths	Global	Both	90-94 years	Prostate cancer	Smoking	Number	1997	394.5177954	701.8339882	165.6064242
Deaths	Global	Both	90-94 years	Prostate cancer	Smoking	Rate	1997	6.156842729	10.95281769	2.584453023
Deaths	Global	Both	90-94 years	Prostate cancer	Smoking	Number	1998	402.0261531	714.6121012	168.546091 5
Deaths	Global	Both	90-94 years	Prostate cancer	Smoking	Rate	1998	5.995396546	10.65697565	2.513519698
Deaths	Global	Both	90-94 years	Prostate cancer	Smoking	Number	1999	412.0522176	732.8726119	172.2711991
Deaths	Global	Both	90-94 years	Prostate cancer	Smoking	Rate	1999	5.882900263	10.46327697	2.459528766
Deaths	Global	Both	90-94 years	Prostate cancer	Smoking	Number	2000	420.6993108	746.9298616	176.6084176
Deaths	Global	Both	90-94 years	Prostate cancer	Smoking	Rate	2000	5.771087133	10.24626664	2.422686562
Deaths	Global	Both	90-94 years	Prostate cancer	Smoking	Number	2001	432.1974012	770.4857997	182.2430114

Deaths	Global	Both	90-94 years	Prostate cancer	Smoking	Rate	2001	5.696030382	10.15441211	2.401823164
Deaths	Global	Both	90-94 years	Prostate cancer	Smoking	Number	2002	440.917532	787.6217106	186.288644
Deaths	Global	Both	90-94 years	Prostate cancer	Smoking	Rate	2002	5.602653236	10.00815573	2.36713633
Deaths	Global	Both	90-94 years	Prostate cancer	Smoking	Number	2003	447.2193681	799.1134972	188.0762883
Deaths	Global	Both	90-94 years	Prostate cancer	Smoking	Rate	2003	5.475163399	9.783290447	2.302557723
Deaths	Global	Both	90-94 years	Prostate cancer	Smoking	Number	2004	446.167724	798.5628702	186.697697
Deaths	Global	Both	90-94 years	Prostate cancer	Smoking	Rate	2004	5.277768846	9.446291184	2.208137976
Deaths	Global	Both	90-94 years	Prostate cancer	Smoking	Number	2005	443.3175328	800.1560349	185.1710669
Deaths	Global	Both	90-94 years	Prostate cancer	Smoking	Rate	2005	5.090146818	9.187346302	2.126123709
Deaths	Global	Both	90-94 years	Prostate cancer	Smoking	Number	2006	440.7333123	798.6607008	183.9983329
Deaths	Global	Both	90-94 years	Prostate cancer	Smoking	Rate	2006	4.953366407	8.976083668	2.067942531

Deaths	Global	Both	90-94 years	Prostate cancer	Smoking	Number	2007	438.2629152	793.5310653	183.495974 6
Deaths	Global	Both	90-94 years	Prostate cancer	Smoking	Rate	2007	4.831184048	8.747476664	2.022764862
Deaths	Global	Both	90-94 years	Prostate cancer	Smoking	Number	2008	432.2526659	783.8739861	181.1582451
Deaths	Global	Both	90-94 years	Prostate cancer	Smoking	Rate	2008	4.665001792	8.45980566	1.955114693
Deaths	Global	Both	90-94 years	Prostate cancer	Smoking	Number	2009	427.8280971	780.1922683	179.3749144
Deaths	Global	Both	90-94 years	Prostate cancer	Smoking	Rate	2009	4.454499374	8.123276602	1.867631998
Deaths	Global	Both	90-94 years	Prostate cancer	Smoking	Number	2010	441.4951922	810.8225439	182.5139279
Deaths	Global	Both	90-94 years	Prostate cancer	Smoking	Rate	2010	4.32648143	7.945746048	1.788565614
Deaths	Global	Both	90-94 years	Prostate cancer	Smoking	Number	2011	466.5934541	855.6102813	194.2512274
Deaths	Global	Both	90-94 years	Prostate cancer	Smoking	Rate	2011	4.26625884	7.823202176	1.776120108
Deaths	Global	Both	90-94 years	Prostate cancer	Smoking	Number	2012	488.4637885	899.8997528	203.7744989

Deaths	Global	Both	90-94 years	Prostate cancer	Smoking	Rate	2012	4.167399616	7.677625185	1.738531676
Deaths	Global	Both	90-94 years	Prostate cancer	Smoking	Number	2013	511.9172339	948.6958523	212.456526
Deaths	Global	Both	90-94 years	Prostate cancer	Smoking	Rate	2013	4.097856726	7.594234813	1.7006986
Deaths	Global	Both	90-94 years	Prostate cancer	Smoking	Number	2014	543.296055	1013.762209	226.8591311
Deaths	Global	Both	90-94 years	Prostate cancer	Smoking	Rate	2014	4.113494164	7.675566374	1.717633882
Deaths	Global	Both	90-94 years	Prostate cancer	Smoking	Number	2015	576.104982	1063.517957	239.5405831
Deaths	Global	Both	90-94 years	Prostate cancer	Smoking	Rate	2015	4.147247266	7.656021172	1.724397565
Deaths	Global	Both	90-94 years	Prostate cancer	Smoking	Number	2016	605.2710717	1128.156554	251.7784962
Deaths	Global	Both	90-94 years	Prostate cancer	Smoking	Rate	2016	4.154718884	7.743924261	1.728265106
Deaths	Global	Both	90-94 years	Prostate cancer	Smoking	Number	2017	623.5502862	1174.669745	256.6955843
Deaths	Global	Both	90-94 years	Prostate cancer	Smoking	Rate	2017	4.079438621	7.685014714	1.679373586

Deaths	Global	Both	90-94 years	Prostate cancer	Smoking	Number	2018	650.9352914	1238.765569	263.6306474
Deaths	Global	Both	90-94 years	Prostate cancer	Smoking	Rate	2018	4.057235958	7.721142604	1.643192121
Deaths	Global	Both	90-94 years	Prostate cancer	Smoking	Number	2019	683.2982363	1288.827752	279.1705196
Deaths	Global	Both	90-94 years	Prostate cancer	Smoking	Rate	2019	4.06235772	7.662363359	1.659729903
Deaths	Global	Both	90-94 years	Prostate cancer	Smoking	Number	2020	701.3350946	1322.17613	287.5963908
Deaths	Global	Both	90-94 years	Prostate cancer	Smoking	Rate	2020	4.021115442	7.580716971	1.648938285
Deaths	Global	Both	90-94 years	Prostate cancer	Smoking	Number	2021	703.8441458	1350.670768	288.7314085
Deaths	Global	Both	90-94 years	Prostate cancer	Smoking	Rate	2021	3.934425888	7.550128913	1.613982776
Deaths	Global	Both	95+ years	Prostate cancer	Smoking	Number	1990	43.46837001	81.87794221	17.10194961
Deaths	Global	Both	95+ years	Prostate cancer	Smoking	Rate	1990	4.269614589	8.04233645	1.679813012
Deaths	Global	Both	95+ years	Prostate cancer	Smoking	Number	1991	46.8050281	88.51083934	18.2944849

Deaths	Global	Both	95+ years	Prostate cancer	Smoking	Rate	1991	4.416619415	8.352066162	1.726305494
Deaths	Global	Both	95+ years	Prostate cancer	Smoking	Number	1992	50.13181579	95.29910828	19.62045656
Deaths	Global	Both	95+ years	Prostate cancer	Smoking	Rate	1992	4.523843447	8.599693423	1.770529801
Deaths	Global	Both	95+ years	Prostate cancer	Smoking	Number	1993	52.97762666	101.8543422	20.6942824
Deaths	Global	Both	95+ years	Prostate cancer	Smoking	Rate	1993	4.579460821	8.804433101	1.788842979
Deaths	Global	Both	95+ years	Prostate cancer	Smoking	Number	1994	54.94526058	105.8698513	21.42303485
Deaths	Global	Both	95+ years	Prostate cancer	Smoking	Rate	1994	4.537088924	8.742172207	1.769000877
Deaths	Global	Both	95+ years	Prostate cancer	Smoking	Number	1995	58.12114604	113.1790797	22.69408714
Deaths	Global	Both	95+ years	Prostate cancer	Smoking	Rate	1995	4.539789769	8.840314809	1.772614472
Deaths	Global	Both	95+ years	Prostate cancer	Smoking	Number	1996	62.07607922	120.9489778	24.23721194
Deaths	Global	Both	95+ years	Prostate cancer	Smoking	Rate	1996	4.533925555	8.833896535	1.770242515

Deaths	Global	Both	95+ years	Prostate cancer	Smoking	Number	1997	64.75077302	127.2523554	25.18820625
Deaths	Global	Both	95+ years	Prostate cancer	Smoking	Rate	1997	4.456494997	8.758188645	1.733587259
Deaths	Global	Both	95+ years	Prostate cancer	Smoking	Number	1998	66.92859323	131.8010073	25.95217993
Deaths	Global	Both	95+ years	Prostate cancer	Smoking	Rate	1998	4.307942461	8.483536383	1.670444461
Deaths	Global	Both	95+ years	Prostate cancer	Smoking	Number	1999	69.58948246	137.7540157	26.89358977
Deaths	Global	Both	95+ years	Prostate cancer	Smoking	Rate	1999	4.185745688	8.285781943	1.617625587
Deaths	Global	Both	95+ years	Prostate cancer	Smoking	Number	2000	71.99605082	142.2992179	27.79434695
Deaths	Global	Both	95+ years	Prostate cancer	Smoking	Rate	2000	4.037800424	7.980657771	1.558808082
Deaths	Global	Both	95+ years	Prostate cancer	Smoking	Number	2001	75.85787086	149.9844368	29.19934663
Deaths	Global	Both	95+ years	Prostate cancer	Smoking	Rate	2001	3.9741644	7.857626408	1.529742432
Deaths	Global	Both	95+ years	Prostate cancer	Smoking	Number	2002	78.7724966	157.1525366	30.22136647

Deaths	Global	Both	95+ years	Prostate cancer	Smoking	Rate	2002	3.860867798	7.702500162	1.481236544
Deaths	Global	Both	95+ years	Prostate cancer	Smoking	Number	2003	82.00014368	165.1596247	31.43923017
Deaths	Global	Both	95+ years	Prostate cancer	Smoking	Rate	2003	3.793295196	7.64022089	1.454366729
Deaths	Global	Both	95+ years	Prostate cancer	Smoking	Number	2004	84.47832154	170.3419647	32.13171394
Deaths	Global	Both	95+ years	Prostate cancer	Smoking	Rate	2004	3.69625498	7.453123167	1.405887398
Deaths	Global	Both	95+ years	Prostate cancer	Smoking	Number	2005	85.74970066	173.7888225	32.5960618
Deaths	Global	Both	95+ years	Prostate cancer	Smoking	Rate	2005	3.555456336	7.205839384	1.351536782
Deaths	Global	Both	95+ years	Prostate cancer	Smoking	Number	2006	88.02507143	178.9496208	33.14172031
Deaths	Global	Both	95+ years	Prostate cancer	Smoking	Rate	2006	3.459265688	7.03247692	1.302424571
Deaths	Global	Both	95+ years	Prostate cancer	Smoking	Number	2007	90.48696125	184.4541508	33.70162147
Deaths	Global	Both	95+ years	Prostate cancer	Smoking	Rate	2007	3.375725552	6.881285232	1.257279758

Deaths	Global	Both	95+ years	Prostate cancer	Smoking	Number	2008	93.73408692	192.0447799	34.76742485
Deaths	Global	Both	95+ years	Prostate cancer	Smoking	Rate	2008	3.317909745	6.797817823	1.230664121
Deaths	Global	Both	95+ years	Prostate cancer	Smoking	Number	2009	95.46316098	196.5047676	35.09417391
Deaths	Global	Both	95+ years	Prostate cancer	Smoking	Rate	2009	3.205313824	6.597932038	1.178337692
Deaths	Global	Both	95+ years	Prostate cancer	Smoking	Number	2010	97.28040864	202.8373604	35.19218457
Deaths	Global	Both	95+ years	Prostate cancer	Smoking	Rate	2010	3.110609415	6.485867112	1.125294828
Deaths	Global	Both	95+ years	Prostate cancer	Smoking	Number	2011	97.95728187	205.2476056	35.08650121
Deaths	Global	Both	95+ years	Prostate cancer	Smoking	Rate	2011	3.009722458	6.306201196	1.078027367
Deaths	Global	Both	95+ years	Prostate cancer	Smoking	Number	2012	97.37010318	204.5164174	34.85040328
Deaths	Global	Both	95+ years	Prostate cancer	Smoking	Rate	2012	2.884492221	6.058594946	1.032408449
Deaths	Global	Both	95+ years	Prostate cancer	Smoking	Number	2013	98.65950024	209.8388826	34.9746226

Deaths	Global	Both	95+ years	Prostate cancer	Smoking	Rate	2013	2.820573228	5.999076958	0.999888343
Deaths	Global	Both	95+ years	Prostate cancer	Smoking	Number	2014	102.6161733	219.8172216	36.21392227
Deaths	Global	Both	95+ years	Prostate cancer	Smoking	Rate	2014	2.814403691	6.02881963	0.993221568
Deaths	Global	Both	95+ years	Prostate cancer	Smoking	Number	2015	108.0069841	232.4384826	38.26995306
Deaths	Global	Both	95+ years	Prostate cancer	Smoking	Rate	2015	2.78736133	5.998593918	0.987641569
Deaths	Global	Both	95+ years	Prostate cancer	Smoking	Number	2016	115.7105682	250.3695733	40.79526777
Deaths	Global	Both	95+ years	Prostate cancer	Smoking	Rate	2016	2.800685328	6.060003	0.987418087
Deaths	Global	Both	95+ years	Prostate cancer	Smoking	Number	2017	124.8142649	271.3845856	43.83512617
Deaths	Global	Both	95+ years	Prostate cancer	Smoking	Rate	2017	2.838679221	6.172161368	0.996952247
Deaths	Global	Both	95+ years	Prostate cancer	Smoking	Number	2018	133.5604425	293.0407286	46.55962818
Deaths	Global	Both	95+ years	Prostate cancer	Smoking	Rate	2018	2.862284437	6.280047452	0.997802168

Deaths	Global	Both	95+ years	Prostate cancer	Smoking	Number	2019	141.3339983	312.4879561	49.21410506
Deaths	Global	Both	95+ years	Prostate cancer	Smoking	Rate	2019	2.854064779	6.310306648	0.993817804
Deaths	Global	Both	95+ years	Prostate cancer	Smoking	Number	2020	146.5309523	325.0245697	51.67615074
Deaths	Global	Both	95+ years	Prostate cancer	Smoking	Rate	2020	2.802800117	6.216972508	0.988445916
Deaths	Global	Both	95+ years	Prostate cancer	Smoking	Number	2021	148.261494	330.750343	52.39605865
Deaths	Global	Both	95+ years	Prostate cancer	Smoking	Rate	2021	2.720240317	6.068469929	0.961341124
DALYs (Disability-Adjusted Life Years)	Global	Both	30-34 years	Prostate cancer	Smoking	Number	1990	470.5402835	764.4605555	171.8286944
DALYs (Disability-Adjusted Life Years)	Global	Both	30-34 years	Prostate cancer	Smoking	Rate	1990	0.122084284	0.198343527	0.044581907
DALYs (Disability-Adjusted Life Years)	Global	Both	30-34 years	Prostate cancer	Smoking	Number	1991	471.2153692	777.6470404	172.0361112
DALYs (Disability-Adjusted Life Years)	Global	Both	30-34 years	Prostate cancer	Smoking	Rate	1991	0.120650799	0.199110095	0.044048424
DALYs (Disability-Adjusted Life Years)	Global	Both	30-34 years	Prostate cancer	Smoking	Number	1992	470.6497439	770.852422	175.5167086

DALYs (Disability-Adjusted Life Years)	Global	Both	30-34 years	Prostate cancer	Smoking	Rate	1992	0.117982435	0.193237216	0.043998513
DALYs (Disability-Adjusted Life Years)	Global	Both	30-34 years	Prostate cancer	Smoking	Number	1993	481.1933944	786.0085958	177.419862
DALYs (Disability-Adjusted Life Years)	Global	Both	30-34 years	Prostate cancer	Smoking	Rate	1993	0.116982401	0.191085692	0.043132349
DALYs (Disability-Adjusted Life Years)	Global	Both	30-34 years	Prostate cancer	Smoking	Number	1994	491.6816712	809.7709596	179.986025
DALYs (Disability-Adjusted Life Years)	Global	Both	30-34 years	Prostate cancer	Smoking	Rate	1994	0.115512514	0.190242356	0.042284753
DALYs (Disability-Adjusted Life Years)	Global	Both	30-34 years	Prostate cancer	Smoking	Number	1995	491.8933868	808.3956057	174.794471 1
DALYs (Disability-Adjusted Life Years)	Global	Both	30-34 years	Prostate cancer	Smoking	Rate	1995	0.111858319	0.183832058	0.039748889
DALYs (Disability-Adjusted Life Years)	Global	Both	30-34 years	Prostate cancer	Smoking	Number	1996	496.1485231	824.7917593	179.374224
DALYs (Disability-Adjusted Life Years)	Global	Both	30-34 years	Prostate cancer	Smoking	Rate	1996	0.109764231	0.182470832	0.039683427
DALYs (Disability-Adjusted Life Years)	Global	Both	30-34 years	Prostate cancer	Smoking	Number	1997	499.0732019	823.1359445	175.1366149
DALYs (Disability-Adjusted Life Years)	Global	Both	30-34 years	Prostate cancer	Smoking	Rate	1997	0.107960274	0.178062019	0.037885819

DALYs (Disability-Adjusted Life Years)	Global	Both	30-34 years	Prostate cancer	Smoking	Number	1998	485.1732691	800.33858	172.797472
DALYs (Disability-Adjusted Life Years)	Global	Both	30-34 years	Prostate cancer	Smoking	Rate	1998	0.1033534	0.170491077	0.036809956
DALYs (Disability-Adjusted Life Years)	Global	Both	30-34 years	Prostate cancer	Smoking	Number	1999	471.4545607	774.7525531	166.2876894
DALYs (Disability-Adjusted Life Years)	Global	Both	30-34 years	Prostate cancer	Smoking	Rate	1999	0.099320572	0.16321587	0.035031559
DALYs (Disability-Adjusted Life Years)	Global	Both	30-34 years	Prostate cancer	Smoking	Number	2000	465.4725002	770.0136907	167.8968759
DALYs (Disability-Adjusted Life Years)	Global	Both	30-34 years	Prostate cancer	Smoking	Rate	2000	0.09715549	0.160720682	0.035044183
DALYs (Disability-Adjusted Life Years)	Global	Both	30-34 years	Prostate cancer	Smoking	Number	2001	453.1071317	743.7754177	168.977048
DALYs (Disability-Adjusted Life Years)	Global	Both	30-34 years	Prostate cancer	Smoking	Rate	2001	0.093607839	0.153657281	0.034909131
DALYs (Disability-Adjusted Life Years)	Global	Both	30-34 years	Prostate cancer	Smoking	Number	2002	454.5192989	742.276646	168.8498258
DALYs (Disability-Adjusted Life Years)	Global	Both	30-34 years	Prostate cancer	Smoking	Rate	2002	0.093048506	0.151957757	0.034566682
DALYs (Disability-Adjusted Life Years)	Global	Both	30-34 years	Prostate cancer	Smoking	Number	2003	460.916541	748.5153615	176.259142

DALYs (Disability-Adjusted Life Years)	Global	Both	30-34 years	Prostate cancer	Smoking	Rate	2003	0.093619317	0.15203511	0.035800973
DALYs (Disability-Adjusted Life Years)	Global	Both	30-34 years	Prostate cancer	Smoking	Number	2004	454.2419821	719.2092782	178.2863052
DALYs (Disability-Adjusted Life Years)	Global	Both	30-34 years	Prostate cancer	Smoking	Rate	2004	0.091716282	0.145215994	0.035997899
DALYs (Disability-Adjusted Life Years)	Global	Both	30-34 years	Prostate cancer	Smoking	Number	2005	456.5826636	713.014201	184.773235
DALYs (Disability-Adjusted Life Years)	Global	Both	30-34 years	Prostate cancer	Smoking	Rate	2005	0.091853599	0.14344154	0.037172009
DALYs (Disability-Adjusted Life Years)	Global	Both	30-34 years	Prostate cancer	Smoking	Number	2006	448.0176984	711.0026928	186.2251401
DALYs (Disability-Adjusted Life Years)	Global	Both	30-34 years	Prostate cancer	Smoking	Rate	2006	0.089994571	0.142821103	0.037407566
DALYs (Disability-Adjusted Life Years)	Global	Both	30-34 years	Prostate cancer	Smoking	Number	2007	438.5051932	693.6397181	184.6320207
DALYs (Disability-Adjusted Life Years)	Global	Both	30-34 years	Prostate cancer	Smoking	Rate	2007	0.088003174	0.13920587	0.037053618
DALYs (Disability-Adjusted Life Years)	Global	Both	30-34 years	Prostate cancer	Smoking	Number	2008	428.0437039	666.843928	182.4830552
DALYs (Disability-Adjusted Life Years)	Global	Both	30-34 years	Prostate cancer	Smoking	Rate	2008	0.08565603	0.133442456	0.036516771

DALYs (Disability-Adjusted Life Years)	Global	Both	30-34 years	Prostate cancer	Smoking	Number	2009	416.7292414	659.391421	174.201252
DALYs (Disability-Adjusted Life Years)	Global	Both	30-34 years	Prostate cancer	Smoking	Rate	2009	0.082832524	0.13106605	0.034629422
DALYs (Disability-Adjusted Life Years)	Global	Both	30-34 years	Prostate cancer	Smoking	Number	2010	415.5284963	660.8876843	171.512369
DALYs (Disability-Adjusted Life Years)	Global	Both	30-34 years	Prostate cancer	Smoking	Rate	2010	0.081754959	0.130029218	0.033744946
DALYs (Disability-Adjusted Life Years)	Global	Both	30-34 years	Prostate cancer	Smoking	Number	2011	418.9423228	665.1638789	174.7473345
DALYs (Disability-Adjusted Life Years)	Global	Both	30-34 years	Prostate cancer	Smoking	Rate	2011	0.081432653	0.129292403	0.033966822
DALYs (Disability-Adjusted Life Years)	Global	Both	30-34 years	Prostate cancer	Smoking	Number	2012	411.8140986	658.4089818	167.4561081
DALYs (Disability-Adjusted Life Years)	Global	Both	30-34 years	Prostate cancer	Smoking	Rate	2012	0.078902921	0.126150105	0.032084322
DALYs (Disability-Adjusted Life Years)	Global	Both	30-34 years	Prostate cancer	Smoking	Number	2013	392.6585725	629.5394683	160.521911
DALYs (Disability-Adjusted Life Years)	Global	Both	30-34 years	Prostate cancer	Smoking	Rate	2013	0.073967787	0.118590665	0.030238613
DALYs (Disability-Adjusted Life Years)	Global	Both	30-34 years	Prostate cancer	Smoking	Number	2014	409.1147188	653.1484454	167.571701

DALYs (Disability-Adjusted Life Years)	Global	Both	30-34 years	Prostate cancer	Smoking	Rate	2014	0.075609661	0.120710232	0.03096940 5
DALYs (Disability-Adjusted Life Years)	Global	Both	30-34 years	Prostate cancer	Smoking	Number	2015	401.8663574	652.6393439	163.4852497
DALYs (Disability-Adjusted Life Years)	Global	Both	30-34 years	Prostate cancer	Smoking	Rate	2015	0.072716348	0.118092865	0.029582099
DALYs (Disability-Adjusted Life Years)	Global	Both	30-34 years	Prostate cancer	Smoking	Number	2016	408.7528652	664.5495441	164.087198
DALYs (Disability-Adjusted Life Years)	Global	Both	30-34 years	Prostate cancer	Smoking	Rate	2016	0.072255641	0.117473068	0.029005853
DALYs (Disability-Adjusted Life Years)	Global	Both	30-34 years	Prostate cancer	Smoking	Number	2017	425.4753624	698.8876145	167.7683335
DALYs (Disability-Adjusted Life Years)	Global	Both	30-34 years	Prostate cancer	Smoking	Rate	2017	0.073507691	0.120744042	0.02898467
DALYs (Disability-Adjusted Life Years)	Global	Both	30-34 years	Prostate cancer	Smoking	Number	2018	442.5117104	741.1660846	173.4920855
DALYs (Disability-Adjusted Life Years)	Global	Both	30-34 years	Prostate cancer	Smoking	Rate	2018	0.074958656	0.125548799	0.029388451
DALYs (Disability-Adjusted Life Years)	Global	Both	30-34 years	Prostate cancer	Smoking	Number	2019	450.0136446	751.4282179	181.8125749
DALYs (Disability-Adjusted Life Years)	Global	Both	30-34 years	Prostate cancer	Smoking	Rate	2019	0.075119636	0.125434007	0.030349512

DALYs (Disability-Adjusted Life Years)	Global	Both	30-34 years	Prostate cancer	Smoking	Number	2020	438.95927 9	713.190039 7	173.7263681
DALYs (Disability-Adjusted Life Years)	Global	Both	30-34 years	Prostate cancer	Smoking	Rate	2020	0.072671174	0.118070992	0.02876098
DALYs (Disability-Adjusted Life Years)	Global	Both	30-34 years	Prostate cancer	Smoking	Number	2021	437.5700511	702.8283461	179.6912799
DALYs (Disability-Adjusted Life Years)	Global	Both	30-34 years	Prostate cancer	Smoking	Rate	2021	0.072387825	0.116269875	0.02972658
DALYs (Disability-Adjusted Life Years)	Global	Both	35-39 years	Prostate cancer	Smoking	Number	1990	987.7532282	1690.280789	328.9552008
DALYs (Disability-Adjusted Life Years)	Global	Both	35-39 years	Prostate cancer	Smoking	Rate	1990	0.280416957	0.479860133	0.093388322
DALYs (Disability-Adjusted Life Years)	Global	Both	35-39 years	Prostate cancer	Smoking	Number	1991	1024.38801	1728.353524	337.8669404
DALYs (Disability-Adjusted Life Years)	Global	Both	35-39 years	Prostate cancer	Smoking	Rate	1991	0.283692062	0.478646929	0.093568226
DALYs (Disability-Adjusted Life Years)	Global	Both	35-39 years	Prostate cancer	Smoking	Number	1992	1050.3441	1815.155273	365.4707628
DALYs (Disability-Adjusted Life Years)	Global	Both	35-39 years	Prostate cancer	Smoking	Rate	1992	0.28511372	0.492720121	0.099206278
DALYs (Disability-Adjusted Life Years)	Global	Both	35-39 years	Prostate cancer	Smoking	Number	1993	1066.240775	1819.767788	364.7367911

DALYs (Disability-Adjusted Life Years)	Global	Both	35-39 years	Prostate cancer	Smoking	Rate	1993	0.285618201	0.48746851	0.09770351
DALYs (Disability-Adjusted Life Years)	Global	Both	35-39 years	Prostate cancer	Smoking	Number	1994	1048.14563	1748.000701	379.0297555
DALYs (Disability-Adjusted Life Years)	Global	Both	35-39 years	Prostate cancer	Smoking	Rate	1994	0.278411399	0.464308878	0.100678953
DALYs (Disability-Adjusted Life Years)	Global	Both	35-39 years	Prostate cancer	Smoking	Number	1995	1022.32022	1713.536843	359.0840468
DALYs (Disability-Adjusted Life Years)	Global	Both	35-39 years	Prostate cancer	Smoking	Rate	1995	0.269384407	0.451522035	0.09461971
DALYs (Disability-Adjusted Life Years)	Global	Both	35-39 years	Prostate cancer	Smoking	Number	1996	1004.383625	1681.178866	365.3216338
DALYs (Disability-Adjusted Life Years)	Global	Both	35-39 years	Prostate cancer	Smoking	Rate	1996	0.261160633	0.437141473	0.094991223
DALYs (Disability-Adjusted Life Years)	Global	Both	35-39 years	Prostate cancer	Smoking	Number	1997	1000.372309	1661.866889	356.5741953
DALYs (Disability-Adjusted Life Years)	Global	Both	35-39 years	Prostate cancer	Smoking	Rate	1997	0.25453038	0.422838185	0.090725188
DALYs (Disability-Adjusted Life Years)	Global	Both	35-39 years	Prostate cancer	Smoking	Number	1998	987.7764341	1651.594238	350.1614738
DALYs (Disability-Adjusted Life Years)	Global	Both	35-39 years	Prostate cancer	Smoking	Rate	1998	0.243546132	0.407217032	0.086335804

DALYs (Disability-Adjusted Life Years)	Global	Both	35-39 years	Prostate cancer	Smoking	Number	1999	979.6918682	1629.630027	336.1273502
DALYs (Disability-Adjusted Life Years)	Global	Both	35-39 years	Prostate cancer	Smoking	Rate	1999	0.233217751	0.387936923	0.080015837
DALYs (Disability-Adjusted Life Years)	Global	Both	35-39 years	Prostate cancer	Smoking	Number	2000	982.6297038	1666.424729	343.7953634
DALYs (Disability-Adjusted Life Years)	Global	Both	35-39 years	Prostate cancer	Smoking	Rate	2000	0.226226218	0.383653133	0.079150391
DALYs (Disability-Adjusted Life Years)	Global	Both	35-39 years	Prostate cancer	Smoking	Number	2001	965.1595423	1630.759596	348.6650647
DALYs (Disability-Adjusted Life Years)	Global	Both	35-39 years	Prostate cancer	Smoking	Rate	2001	0.216088467	0.365108902	0.078062223
DALYs (Disability-Adjusted Life Years)	Global	Both	35-39 years	Prostate cancer	Smoking	Number	2002	963.893846	1577.991357	359.7637084
DALYs (Disability-Adjusted Life Years)	Global	Both	35-39 years	Prostate cancer	Smoking	Rate	2002	0.210932591	0.345317907	0.078728474
DALYs (Disability-Adjusted Life Years)	Global	Both	35-39 years	Prostate cancer	Smoking	Number	2003	921.0546691	1508.752628	364.7753743
DALYs (Disability-Adjusted Life Years)	Global	Both	35-39 years	Prostate cancer	Smoking	Rate	2003	0.198364616	0.324935257	0.078560513
DALYs (Disability-Adjusted Life Years)	Global	Both	35-39 years	Prostate cancer	Smoking	Number	2004	886.9081928	1435.801703	359.9520674

DALYs (Disability-Adjusted Life Years)	Global	Both	35-39 years	Prostate cancer	Smoking	Rate	2004	0.188722101	0.305519237	0.076592945
DALYs (Disability-Adjusted Life Years)	Global	Both	35-39 years	Prostate cancer	Smoking	Number	2005	872.5131807	1397.530661	358.4749964
DALYs (Disability-Adjusted Life Years)	Global	Both	35-39 years	Prostate cancer	Smoking	Rate	2005	0.183750201	0.294318236	0.075494393
DALYs (Disability-Adjusted Life Years)	Global	Both	35-39 years	Prostate cancer	Smoking	Number	2006	857.3645104	1352.22914	351.5112818
DALYs (Disability-Adjusted Life Years)	Global	Both	35-39 years	Prostate cancer	Smoking	Rate	2006	0.178521445	0.28156274	0.073192092
DALYs (Disability-Adjusted Life Years)	Global	Both	35-39 years	Prostate cancer	Smoking	Number	2007	838.4317044	1331.642687	351.9342655
DALYs (Disability-Adjusted Life Years)	Global	Both	35-39 years	Prostate cancer	Smoking	Rate	2007	0.172825834	0.274491358	0.072544171
DALYs (Disability-Adjusted Life Years)	Global	Both	35-39 years	Prostate cancer	Smoking	Number	2008	815.5060811	1272.865163	348.8689475
DALYs (Disability-Adjusted Life Years)	Global	Both	35-39 years	Prostate cancer	Smoking	Rate	2008	0.166617386	0.260061171	0.071277987
DALYs (Disability-Adjusted Life Years)	Global	Both	35-39 years	Prostate cancer	Smoking	Number	2009	784.9652225	1223.429491	331.4946167
DALYs (Disability-Adjusted Life Years)	Global	Both	35-39 years	Prostate cancer	Smoking	Rate	2009	0.159286294	0.248260106	0.067267374

DALYs (Disability-Adjusted Life Years)	Global	Both	35-39 years	Prostate cancer	Smoking	Number	2010	766.3369791	1192.916968	317.940090 5
DALYs (Disability-Adjusted Life Years)	Global	Both	35-39 years	Prostate cancer	Smoking	Rate	2010	0.154893199	0.241114198	0.0642625 36
DALYs (Disability-Adjusted Life Years)	Global	Both	35-39 years	Prostate cancer	Smoking	Number	2011	753.1153283	1173.903899	323.057482
DALYs (Disability-Adjusted Life Years)	Global	Both	35-39 years	Prostate cancer	Smoking	Rate	2011	0.152037538	0.236985561	0.0652182 5
DALYs (Disability-Adjusted Life Years)	Global	Both	35-39 years	Prostate cancer	Smoking	Number	2012	733.6797871	1143.708118	306.610660 1
DALYs (Disability-Adjusted Life Years)	Global	Both	35-39 years	Prostate cancer	Smoking	Rate	2012	0.148052836	0.23079446	0.0618724 66
DALYs (Disability-Adjusted Life Years)	Global	Both	35-39 years	Prostate cancer	Smoking	Number	2013	709.3066045	1108.862845	302.818889 9
DALYs (Disability-Adjusted Life Years)	Global	Both	35-39 years	Prostate cancer	Smoking	Rate	2013	0.142851395	0.223320358	0.0609864 63
DALYs (Disability-Adjusted Life Years)	Global	Both	35-39 years	Prostate cancer	Smoking	Number	2014	731.237412	1133.183193	318.737391 6
DALYs (Disability-Adjusted Life Years)	Global	Both	35-39 years	Prostate cancer	Smoking	Rate	2014	0.146443622	0.226940591	0.0638329 73
DALYs (Disability-Adjusted Life Years)	Global	Both	35-39 years	Prostate cancer	Smoking	Number	2015	722.7070031	1132.192912	309.258290 7

DALYs (Disability-Adjusted Life Years)	Global	Both	35-39 years	Prostate cancer	Smoking	Rate	2015	0.143324104	0.224531565	0.061330757
DALYs (Disability-Adjusted Life Years)	Global	Both	35-39 years	Prostate cancer	Smoking	Number	2016	723.2969041	1132.92876	297.5104029
DALYs (Disability-Adjusted Life Years)	Global	Both	35-39 years	Prostate cancer	Smoking	Rate	2016	0.141704199	0.221956933	0.058286539
DALYs (Disability-Adjusted Life Years)	Global	Both	35-39 years	Prostate cancer	Smoking	Number	2017	734.1281058	1177.90785	310.4175464
DALYs (Disability-Adjusted Life Years)	Global	Both	35-39 years	Prostate cancer	Smoking	Rate	2017	0.14178311	0.227490866	0.059951342
DALYs (Disability-Adjusted Life Years)	Global	Both	35-39 years	Prostate cancer	Smoking	Number	2018	754.2936173	1183.03737	314.6165666
DALYs (Disability-Adjusted Life Years)	Global	Both	35-39 years	Prostate cancer	Smoking	Rate	2018	0.143247712	0.22467033	0.059748753
DALYs (Disability-Adjusted Life Years)	Global	Both	35-39 years	Prostate cancer	Smoking	Number	2019	763.4059184	1237.973643	324.1290736
DALYs (Disability-Adjusted Life Years)	Global	Both	35-39 years	Prostate cancer	Smoking	Rate	2019	0.142242755	0.230667299	0.060393837
DALYs (Disability-Adjusted Life Years)	Global	Both	35-39 years	Prostate cancer	Smoking	Number	2020	754.7314928	1193.783148	306.2528241
DALYs (Disability-Adjusted Life Years)	Global	Both	35-39 years	Prostate cancer	Smoking	Rate	2020	0.137704989	0.217812423	0.055877543

DALYs (Disability-Adjusted Life Years)	Global	Both	35-39 years	Prostate cancer	Smoking	Number	2021	778.1794057	1250.604006	321.1413077
DALYs (Disability-Adjusted Life Years)	Global	Both	35-39 years	Prostate cancer	Smoking	Rate	2021	0.138746021	0.222977283	0.057258106
DALYs (Disability-Adjusted Life Years)	Global	Both	40-44 years	Prostate cancer	Smoking	Number	1990	1311.809237	2091.049407	548.1458291
DALYs (Disability-Adjusted Life Years)	Global	Both	40-44 years	Prostate cancer	Smoking	Rate	1990	0.457903432	0.729906965	0.191337162
DALYs (Disability-Adjusted Life Years)	Global	Both	40-44 years	Prostate cancer	Smoking	Number	1991	1406.898808	2261.38241	591.784876
DALYs (Disability-Adjusted Life Years)	Global	Both	40-44 years	Prostate cancer	Smoking	Rate	1991	0.46813978	0.752465678	0.196913979
DALYs (Disability-Adjusted Life Years)	Global	Both	40-44 years	Prostate cancer	Smoking	Number	1992	1465.715559	2360.223617	603.7772644
DALYs (Disability-Adjusted Life Years)	Global	Both	40-44 years	Prostate cancer	Smoking	Rate	1992	0.47173541	0.759629691	0.194323594
DALYs (Disability-Adjusted Life Years)	Global	Both	40-44 years	Prostate cancer	Smoking	Number	1993	1530.798956	2446.870896	642.1008897
DALYs (Disability-Adjusted Life Years)	Global	Both	40-44 years	Prostate cancer	Smoking	Rate	1993	0.475601767	0.760214866	0.199493422
DALYs (Disability-Adjusted Life Years)	Global	Both	40-44 years	Prostate cancer	Smoking	Number	1994	1567.032813	2481.109295	655.7747821

DALYs (Disability-Adjusted Life Years)	Global	Both	40-44 years	Prostate cancer	Smoking	Rate	1994	0.47368298	0.749990194	0.198227727
DALYs (Disability-Adjusted Life Years)	Global	Both	40-44 years	Prostate cancer	Smoking	Number	1995	1617.272002	2584.55625	685.601499
DALYs (Disability-Adjusted Life Years)	Global	Both	40-44 years	Prostate cancer	Smoking	Rate	1995	0.470988044	0.752684206	0.199663451
DALYs (Disability-Adjusted Life Years)	Global	Both	40-44 years	Prostate cancer	Smoking	Number	1996	1644.017793	2618.276048	678.8851995
DALYs (Disability-Adjusted Life Years)	Global	Both	40-44 years	Prostate cancer	Smoking	Rate	1996	0.468234894	0.745714684	0.193354197
DALYs (Disability-Adjusted Life Years)	Global	Both	40-44 years	Prostate cancer	Smoking	Number	1997	1648.255549	2626.616569	706.4289352
DALYs (Disability-Adjusted Life Years)	Global	Both	40-44 years	Prostate cancer	Smoking	Rate	1997	0.461030901	0.734686685	0.197594098
DALYs (Disability-Adjusted Life Years)	Global	Both	40-44 years	Prostate cancer	Smoking	Number	1998	1657.962067	2647.973916	697.4453195
DALYs (Disability-Adjusted Life Years)	Global	Both	40-44 years	Prostate cancer	Smoking	Rate	1998	0.45819146	0.731789382	0.192744753
DALYs (Disability-Adjusted Life Years)	Global	Both	40-44 years	Prostate cancer	Smoking	Number	1999	1655.457107	2608.316139	690.204906
DALYs (Disability-Adjusted Life Years)	Global	Both	40-44 years	Prostate cancer	Smoking	Rate	1999	0.453824184	0.715039331	0.189211594

DALYs (Disability-Adjusted Life Years)	Global	Both	40-44 years	Prostate cancer	Smoking	Number	2000	1637.813426	2614.965786	698.963326 5
DALYs (Disability-Adjusted Life Years)	Global	Both	40-44 years	Prostate cancer	Smoking	Rate	2000	0.445260691	0.710912155	0.1900221 9
DALYs (Disability-Adjusted Life Years)	Global	Both	40-44 years	Prostate cancer	Smoking	Number	2001	1613.421568	2562.544885	674.7214834
DALYs (Disability-Adjusted Life Years)	Global	Both	40-44 years	Prostate cancer	Smoking	Rate	2001	0.432518217	0.686954585	0.180876058
DALYs (Disability-Adjusted Life Years)	Global	Both	40-44 years	Prostate cancer	Smoking	Number	2002	1594.006695	2511.264732	675.9453008
DALYs (Disability-Adjusted Life Years)	Global	Both	40-44 years	Prostate cancer	Smoking	Rate	2002	0.417764988	0.658164413	0.177155015
DALYs (Disability-Adjusted Life Years)	Global	Both	40-44 years	Prostate cancer	Smoking	Number	2003	1568.009547	2443.551451	669.2292232
DALYs (Disability-Adjusted Life Years)	Global	Both	40-44 years	Prostate cancer	Smoking	Rate	2003	0.397858156	0.620013364	0.169806558
DALYs (Disability-Adjusted Life Years)	Global	Both	40-44 years	Prostate cancer	Smoking	Number	2004	1587.792578	2477.119361	680.3236681
DALYs (Disability-Adjusted Life Years)	Global	Both	40-44 years	Prostate cancer	Smoking	Rate	2004	0.388545232	0.606170434	0.166480509
DALYs (Disability-Adjusted Life Years)	Global	Both	40-44 years	Prostate cancer	Smoking	Number	2005	1637.429864	2540.009877	709.7544704

DALYs (Disability-Adjusted Life Years)	Global	Both	40-44 years	Prostate cancer	Smoking	Rate	2005	0.387055996	0.600408039	0.167771903
DALYs (Disability-Adjusted Life Years)	Global	Both	40-44 years	Prostate cancer	Smoking	Number	2006	1653.884935	2553.915335	712.8261101
DALYs (Disability-Adjusted Life Years)	Global	Both	40-44 years	Prostate cancer	Smoking	Rate	2006	0.379779533	0.586452391	0.16368537
DALYs (Disability-Adjusted Life Years)	Global	Both	40-44 years	Prostate cancer	Smoking	Number	2007	1621.514117	2525.35259	727.1710327
DALYs (Disability-Adjusted Life Years)	Global	Both	40-44 years	Prostate cancer	Smoking	Rate	2007	0.363525732	0.566156434	0.163023793
DALYs (Disability-Adjusted Life Years)	Global	Both	40-44 years	Prostate cancer	Smoking	Number	2008	1575.842862	2419.453285	690.7468477
DALYs (Disability-Adjusted Life Years)	Global	Both	40-44 years	Prostate cancer	Smoking	Rate	2008	0.347288104	0.533205032	0.1522848
DALYs (Disability-Adjusted Life Years)	Global	Both	40-44 years	Prostate cancer	Smoking	Number	2009	1520.465155	2342.474404	663.1127573
DALYs (Disability-Adjusted Life Years)	Global	Both	40-44 years	Prostate cancer	Smoking	Rate	2009	0.330726665	0.50952746	0.144238143
DALYs (Disability-Adjusted Life Years)	Global	Both	40-44 years	Prostate cancer	Smoking	Number	2010	1493.228049	2297.884302	653.6890002
DALYs (Disability-Adjusted Life Years)	Global	Both	40-44 years	Prostate cancer	Smoking	Rate	2010	0.321247115	0.494357646	0.14063204

DALYs (Disability-Adjusted Life Years)	Global	Both	40-44 years	Prostate cancer	Smoking	Number	2011	1489.198619	2328.796163	658.113013
DALYs (Disability-Adjusted Life Years)	Global	Both	40-44 years	Prostate cancer	Smoking	Rate	2011	0.316644837	0.495166509	0.139932675
DALYs (Disability-Adjusted Life Years)	Global	Both	40-44 years	Prostate cancer	Smoking	Number	2012	1466.757548	2281.687184	638.8634031
DALYs (Disability-Adjusted Life Years)	Global	Both	40-44 years	Prostate cancer	Smoking	Rate	2012	0.308671037	0.480168485	0.1344452 8
DALYs (Disability-Adjusted Life Years)	Global	Both	40-44 years	Prostate cancer	Smoking	Number	2013	1451.608163	2272.287334	636.157049
DALYs (Disability-Adjusted Life Years)	Global	Both	40-44 years	Prostate cancer	Smoking	Rate	2013	0.302796765	0.473985522	0.132698549
DALYs (Disability-Adjusted Life Years)	Global	Both	40-44 years	Prostate cancer	Smoking	Number	2014	1476.304978	2271.946271	627.5834212
DALYs (Disability-Adjusted Life Years)	Global	Both	40-44 years	Prostate cancer	Smoking	Rate	2014	0.305929135	0.470806891	0.130051755
DALYs (Disability-Adjusted Life Years)	Global	Both	40-44 years	Prostate cancer	Smoking	Number	2015	1459.117639	2290.029602	629.9387116
DALYs (Disability-Adjusted Life Years)	Global	Both	40-44 years	Prostate cancer	Smoking	Rate	2015	0.301183661	0.472696293	0.130028753
DALYs (Disability-Adjusted Life Years)	Global	Both	40-44 years	Prostate cancer	Smoking	Number	2016	1441.503969	2230.112338	611.0303469

DALYs (Disability-Adjusted Life Years)	Global	Both	40-44 years	Prostate cancer	Smoking	Rate	2016	0.297143936	0.459703456	0.125954535
DALYs (Disability-Adjusted Life Years)	Global	Both	40-44 years	Prostate cancer	Smoking	Number	2017	1417.321837	2244.920893	589.772127
DALYs (Disability-Adjusted Life Years)	Global	Both	40-44 years	Prostate cancer	Smoking	Rate	2017	0.292006882	0.462514817	0.121509131
DALYs (Disability-Adjusted Life Years)	Global	Both	40-44 years	Prostate cancer	Smoking	Number	2018	1419.601255	2190.772081	607.583385
DALYs (Disability-Adjusted Life Years)	Global	Both	40-44 years	Prostate cancer	Smoking	Rate	2018	0.291869156	0.45042141	0.124918775
DALYs (Disability-Adjusted Life Years)	Global	Both	40-44 years	Prostate cancer	Smoking	Number	2019	1424.733805	2232.939975	605.7289301
DALYs (Disability-Adjusted Life Years)	Global	Both	40-44 years	Prostate cancer	Smoking	Rate	2019	0.291209662	0.45640364	0.123808473
DALYs (Disability-Adjusted Life Years)	Global	Both	40-44 years	Prostate cancer	Smoking	Number	2020	1399.949207	2180.879758	602.0289364
DALYs (Disability-Adjusted Life Years)	Global	Both	40-44 years	Prostate cancer	Smoking	Rate	2020	0.283280333	0.44130197	0.121820818
DALYs (Disability-Adjusted Life Years)	Global	Both	40-44 years	Prostate cancer	Smoking	Number	2021	1409.666907	2198.641572	599.4043913
DALYs (Disability-Adjusted Life Years)	Global	Both	40-44 years	Prostate cancer	Smoking	Rate	2021	0.281792037	0.439507861	0.119820777

DALYs (Disability-Adjusted Life Years)	Global	Both	45-49 years	Prostate cancer	Smoking	Number	1990	3321.238868	5163.504989	1497.348219
DALYs (Disability-Adjusted Life Years)	Global	Both	45-49 years	Prostate cancer	Smoking	Rate	1990	1.430363197	2.223774861	0.644865326
DALYs (Disability-Adjusted Life Years)	Global	Both	45-49 years	Prostate cancer	Smoking	Number	1991	3369.042465	5206.176029	1503.479101
DALYs (Disability-Adjusted Life Years)	Global	Both	45-49 years	Prostate cancer	Smoking	Rate	1991	1.431270256	2.211739678	0.638723002
DALYs (Disability-Adjusted Life Years)	Global	Both	45-49 years	Prostate cancer	Smoking	Number	1992	3516.746812	5428.210582	1585.362742
DALYs (Disability-Adjusted Life Years)	Global	Both	45-49 years	Prostate cancer	Smoking	Rate	1992	1.443706317	2.228406629	0.650828259
DALYs (Disability-Adjusted Life Years)	Global	Both	45-49 years	Prostate cancer	Smoking	Number	1993	3681.87889	5667.848948	1663.605903
DALYs (Disability-Adjusted Life Years)	Global	Both	45-49 years	Prostate cancer	Smoking	Rate	1993	1.45786088	2.244217016	0.658714216
DALYs (Disability-Adjusted Life Years)	Global	Both	45-49 years	Prostate cancer	Smoking	Number	1994	3939.562577	6050.515857	1804.775042
DALYs (Disability-Adjusted Life Years)	Global	Both	45-49 years	Prostate cancer	Smoking	Rate	1994	1.485333913	2.281226969	0.680454625
DALYs (Disability-Adjusted Life Years)	Global	Both	45-49 years	Prostate cancer	Smoking	Number	1995	4127.899655	6336.692144	1855.806608

DALYs (Disability-Adjusted Life Years)	Global	Both	45-49 years	Prostate cancer	Smoking	Rate	1995	1.497828271	2.299299263	0.673388366
DALYs (Disability-Adjusted Life Years)	Global	Both	45-49 years	Prostate cancer	Smoking	Number	1996	4365.774276	6753.612292	1978.992178
DALYs (Disability-Adjusted Life Years)	Global	Both	45-49 years	Prostate cancer	Smoking	Rate	1996	1.512196136	2.339284114	0.685473901
DALYs (Disability-Adjusted Life Years)	Global	Both	45-49 years	Prostate cancer	Smoking	Number	1997	4457.236083	6891.7623	2011.129085
DALYs (Disability-Adjusted Life Years)	Global	Both	45-49 years	Prostate cancer	Smoking	Rate	1997	1.495348489	2.312102422	0.674709345
DALYs (Disability-Adjusted Life Years)	Global	Both	45-49 years	Prostate cancer	Smoking	Number	1998	4556.771808	7121.488515	2055.09634
DALYs (Disability-Adjusted Life Years)	Global	Both	45-49 years	Prostate cancer	Smoking	Rate	1998	1.477078431	2.308431829	0.666159862
DALYs (Disability-Adjusted Life Years)	Global	Both	45-49 years	Prostate cancer	Smoking	Number	1999	4586.192178	7138.918735	2058.057975
DALYs (Disability-Adjusted Life Years)	Global	Both	45-49 years	Prostate cancer	Smoking	Rate	1999	1.447250051	2.252805836	0.649454797
DALYs (Disability-Adjusted Life Years)	Global	Both	45-49 years	Prostate cancer	Smoking	Number	2000	4717.675805	7412.435617	2128.748196
DALYs (Disability-Adjusted Life Years)	Global	Both	45-49 years	Prostate cancer	Smoking	Rate	2000	1.434093021	2.253254066	0.647103162

DALYs (Disability-Adjusted Life Years)	Global	Both	45-49 years	Prostate cancer	Smoking	Number	2001	4737.213243	7439.995567	2130.833233
DALYs (Disability-Adjusted Life Years)	Global	Both	45-49 years	Prostate cancer	Smoking	Rate	2001	1.40708438	2.209886069	0.632916866
DALYs (Disability-Adjusted Life Years)	Global	Both	45-49 years	Prostate cancer	Smoking	Number	2002	4764.926333	7424.388611	2160.426193
DALYs (Disability-Adjusted Life Years)	Global	Both	45-49 years	Prostate cancer	Smoking	Rate	2002	1.388388243	2.163293436	0.629497733
DALYs (Disability-Adjusted Life Years)	Global	Both	45-49 years	Prostate cancer	Smoking	Number	2003	4773.641002	7406.579502	2180.416878
DALYs (Disability-Adjusted Life Years)	Global	Both	45-49 years	Prostate cancer	Smoking	Rate	2003	1.372416954	2.129384106	0.626867644
DALYs (Disability-Adjusted Life Years)	Global	Both	45-49 years	Prostate cancer	Smoking	Number	2004	4756.991184	7429.527544	2189.165607
DALYs (Disability-Adjusted Life Years)	Global	Both	45-49 years	Prostate cancer	Smoking	Rate	2004	1.354610352	2.115647167	0.62339119
DALYs (Disability-Adjusted Life Years)	Global	Both	45-49 years	Prostate cancer	Smoking	Number	2005	4722.341593	7300.587424	2172.6174
DALYs (Disability-Adjusted Life Years)	Global	Both	45-49 years	Prostate cancer	Smoking	Rate	2005	1.33167464	2.058725938	0.612666288
DALYs (Disability-Adjusted Life Years)	Global	Both	45-49 years	Prostate cancer	Smoking	Number	2006	4640.367362	7178.378466	2152.262968

DALYs (Disability-Adjusted Life Years)	Global	Both	45-49 years	Prostate cancer	Smoking	Rate	2006	1.28858842	1.993371353	0.597664134
DALYs (Disability-Adjusted Life Years)	Global	Both	45-49 years	Prostate cancer	Smoking	Number	2007	4556.160124	7027.647901	2099.836389
DALYs (Disability-Adjusted Life Years)	Global	Both	45-49 years	Prostate cancer	Smoking	Rate	2007	1.235141792	1.905144109	0.569250336
DALYs (Disability-Adjusted Life Years)	Global	Both	45-49 years	Prostate cancer	Smoking	Number	2008	4548.698624	6988.155435	2125.113494
DALYs (Disability-Adjusted Life Years)	Global	Both	45-49 years	Prostate cancer	Smoking	Rate	2008	1.191836791	1.831016173	0.55681606
DALYs (Disability-Adjusted Life Years)	Global	Both	45-49 years	Prostate cancer	Smoking	Number	2009	4549.327487	7021.875453	2069.432827
DALYs (Disability-Adjusted Life Years)	Global	Both	45-49 years	Prostate cancer	Smoking	Rate	2009	1.147717913	1.771499692	0.522082689
DALYs (Disability-Adjusted Life Years)	Global	Both	45-49 years	Prostate cancer	Smoking	Number	2010	4554.151597	6955.788785	2038.533996
DALYs (Disability-Adjusted Life Years)	Global	Both	45-49 years	Prostate cancer	Smoking	Rate	2010	1.108326271	1.692803431	0.496110139
DALYs (Disability-Adjusted Life Years)	Global	Both	45-49 years	Prostate cancer	Smoking	Number	2011	4512.970603	6972.679624	2021.960742
DALYs (Disability-Adjusted Life Years)	Global	Both	45-49 years	Prostate cancer	Smoking	Rate	2011	1.065936969	1.646905694	0.47757517

DALYs (Disability-Adjusted Life Years)	Global	Both	45-49 years	Prostate cancer	Smoking	Number	2012	4472.630648	6883.85103	2022.824265
DALYs (Disability-Adjusted Life Years)	Global	Both	45-49 years	Prostate cancer	Smoking	Rate	2012	1.030505029	1.586056096	0.466063653
DALYs (Disability-Adjusted Life Years)	Global	Both	45-49 years	Prostate cancer	Smoking	Number	2013	4338.311131	6794.978298	1930.678071
DALYs (Disability-Adjusted Life Years)	Global	Both	45-49 years	Prostate cancer	Smoking	Rate	2013	0.981997647	1.538076109	0.437018293
DALYs (Disability-Adjusted Life Years)	Global	Both	45-49 years	Prostate cancer	Smoking	Number	2014	4214.547687	6520.783241	1878.277153
DALYs (Disability-Adjusted Life Years)	Global	Both	45-49 years	Prostate cancer	Smoking	Rate	2014	0.941233931	1.456284968	0.419475189
DALYs (Disability-Adjusted Life Years)	Global	Both	45-49 years	Prostate cancer	Smoking	Number	2015	4125.571054	6382.396473	1811.09773
DALYs (Disability-Adjusted Life Years)	Global	Both	45-49 years	Prostate cancer	Smoking	Rate	2015	0.910848679	1.409113386	0.399856396
DALYs (Disability-Adjusted Life Years)	Global	Both	45-49 years	Prostate cancer	Smoking	Number	2016	4110.611056	6403.20691	1797.029312
DALYs (Disability-Adjusted Life Years)	Global	Both	45-49 years	Prostate cancer	Smoking	Rate	2016	0.896318228	1.396218468	0.391842017
DALYs (Disability-Adjusted Life Years)	Global	Both	45-49 years	Prostate cancer	Smoking	Number	2017	4098.158532	6560.486999	1789.662755

DALYs (Disability-Adjusted Life Years)	Global	Both	45-49 years	Prostate cancer	Smoking	Rate	2017	0.883926782	1.415023386	0.386010162
DALYs (Disability-Adjusted Life Years)	Global	Both	45-49 years	Prostate cancer	Smoking	Number	2018	4074.220803	6322.96878	1804.841985
DALYs (Disability-Adjusted Life Years)	Global	Both	45-49 years	Prostate cancer	Smoking	Rate	2018	0.870631548	1.351172742	0.385681691
DALYs (Disability-Adjusted Life Years)	Global	Both	45-49 years	Prostate cancer	Smoking	Number	2019	4088.743697	6463.897541	1783.61088
DALYs (Disability-Adjusted Life Years)	Global	Both	45-49 years	Prostate cancer	Smoking	Rate	2019	0.867674268	1.371706809	0.378500924
DALYs (Disability-Adjusted Life Years)	Global	Both	45-49 years	Prostate cancer	Smoking	Number	2020	4041.046106	6246.941068	1762.7302
DALYs (Disability-Adjusted Life Years)	Global	Both	45-49 years	Prostate cancer	Smoking	Rate	2020	0.854189532	1.320467901	0.372602946
DALYs (Disability-Adjusted Life Years)	Global	Both	45-49 years	Prostate cancer	Smoking	Number	2021	4028.43021	6253.248338	1761.566819
DALYs (Disability-Adjusted Life Years)	Global	Both	45-49 years	Prostate cancer	Smoking	Rate	2021	0.850768924	1.320630886	0.372027373
DALYs (Disability-Adjusted Life Years)	Global	Both	50-54 years	Prostate cancer	Smoking	Number	1990	9090.771673	13969.71213	4206.339644
DALYs (Disability-Adjusted Life Years)	Global	Both	50-54 years	Prostate cancer	Smoking	Rate	1990	4.276571921	6.571771988	1.978788452

DALYs (Disability-Adjusted Life Years)	Global	Both	50-54 years	Prostate cancer	Smoking	Number	1991	9302.719792	14334.94304	4310.045477
DALYs (Disability-Adjusted Life Years)	Global	Both	50-54 years	Prostate cancer	Smoking	Rate	1991	4.329243474	6.671109094	2.005782897
DALYs (Disability-Adjusted Life Years)	Global	Both	50-54 years	Prostate cancer	Smoking	Number	1992	9464.598016	14596.82622	4388.928612
DALYs (Disability-Adjusted Life Years)	Global	Both	50-54 years	Prostate cancer	Smoking	Rate	1992	4.375549863	6.74821486	2.029032396
DALYs (Disability-Adjusted Life Years)	Global	Both	50-54 years	Prostate cancer	Smoking	Number	1993	9569.022184	14740.03272	4445.501174
DALYs (Disability-Adjusted Life Years)	Global	Both	50-54 years	Prostate cancer	Smoking	Rate	1993	4.404794728	6.785104809	2.046344941
DALYs (Disability-Adjusted Life Years)	Global	Both	50-54 years	Prostate cancer	Smoking	Number	1994	9588.462784	14717.06849	4495.953056
DALYs (Disability-Adjusted Life Years)	Global	Both	50-54 years	Prostate cancer	Smoking	Rate	1994	4.3953724	6.74633652	2.060954756
DALYs (Disability-Adjusted Life Years)	Global	Both	50-54 years	Prostate cancer	Smoking	Number	1995	9550.519547	14708.11524	4395.864006
DALYs (Disability-Adjusted Life Years)	Global	Both	50-54 years	Prostate cancer	Smoking	Rate	1995	4.365602807	6.723172373	2.009377202
DALYs (Disability-Adjusted Life Years)	Global	Both	50-54 years	Prostate cancer	Smoking	Number	1996	9604.045249	14800.38291	4409.348217

DALYs (Disability-Adjusted Life Years)	Global	Both	50-54 years	Prostate cancer	Smoking	Rate	1996	4.333396133	6.678011236	1.989521292
DALYs (Disability-Adjusted Life Years)	Global	Both	50-54 years	Prostate cancer	Smoking	Number	1997	9798.299787	15133.81664	4470.345893
DALYs (Disability-Adjusted Life Years)	Global	Both	50-54 years	Prostate cancer	Smoking	Rate	1997	4.267960917	6.592014874	1.947201246
DALYs (Disability-Adjusted Life Years)	Global	Both	50-54 years	Prostate cancer	Smoking	Number	1998	10103.71633	15716.84855	4611.733696
DALYs (Disability-Adjusted Life Years)	Global	Both	50-54 years	Prostate cancer	Smoking	Rate	1998	4.239773941	6.595185646	1.935199654
DALYs (Disability-Adjusted Life Years)	Global	Both	50-54 years	Prostate cancer	Smoking	Number	1999	10496.19542	16199.79961	4792.00071
DALYs (Disability-Adjusted Life Years)	Global	Both	50-54 years	Prostate cancer	Smoking	Rate	1999	4.185402437	6.459738796	1.910830605
DALYs (Disability-Adjusted Life Years)	Global	Both	50-54 years	Prostate cancer	Smoking	Number	2000	10948.53079	16952.31352	5038.190216
DALYs (Disability-Adjusted Life Years)	Global	Both	50-54 years	Prostate cancer	Smoking	Rate	2000	4.19290253	6.492140323	1.929449795
DALYs (Disability-Adjusted Life Years)	Global	Both	50-54 years	Prostate cancer	Smoking	Number	2001	11531.14073	17828.91891	5310.713427
DALYs (Disability-Adjusted Life Years)	Global	Both	50-54 years	Prostate cancer	Smoking	Rate	2001	4.207959187	6.50615276	1.937992595

DALYs (Disability-Adjusted Life Years)	Global	Both	50-54 years	Prostate cancer	Smoking	Number	2002	11769.68361	17930.53189	5457.179901
DALYs (Disability-Adjusted Life Years)	Global	Both	50-54 years	Prostate cancer	Smoking	Rate	2002	4.153343839	6.327414282	1.925756484
DALYs (Disability-Adjusted Life Years)	Global	Both	50-54 years	Prostate cancer	Smoking	Number	2003	11879.6744	18158.83628	5548.851983
DALYs (Disability-Adjusted Life Years)	Global	Both	50-54 years	Prostate cancer	Smoking	Rate	2003	4.043154106	6.180217655	1.888508298
DALYs (Disability-Adjusted Life Years)	Global	Both	50-54 years	Prostate cancer	Smoking	Number	2004	11948.63252	18266.24471	5569.499508
DALYs (Disability-Adjusted Life Years)	Global	Both	50-54 years	Prostate cancer	Smoking	Rate	2004	3.951701751	6.041088891	1.841968185
DALYs (Disability-Adjusted Life Years)	Global	Both	50-54 years	Prostate cancer	Smoking	Number	2005	12137.3353	18587.02961	5685.212257
DALYs (Disability-Adjusted Life Years)	Global	Both	50-54 years	Prostate cancer	Smoking	Rate	2005	3.8587833335	5.909313589	1.807480956
DALYs (Disability-Adjusted Life Years)	Global	Both	50-54 years	Prostate cancer	Smoking	Number	2006	12099.17569	18392.57029	5640.320901
DALYs (Disability-Adjusted Life Years)	Global	Both	50-54 years	Prostate cancer	Smoking	Rate	2006	3.751109142	5.702251154	1.748669482
DALYs (Disability-Adjusted Life Years)	Global	Both	50-54 years	Prostate cancer	Smoking	Number	2007	11981.72577	18130.45771	5546.20549

DALYs (Disability-Adjusted Life Years)	Global	Both	50-54 years	Prostate cancer	Smoking	Rate	2007	3.637543061	5.504242203	1.683777586
DALYs (Disability-Adjusted Life Years)	Global	Both	50-54 years	Prostate cancer	Smoking	Number	2008	12035.00105	18291.99829	5613.575393
DALYs (Disability-Adjusted Life Years)	Global	Both	50-54 years	Prostate cancer	Smoking	Rate	2008	3.599509235	5.470894145	1.678945967
DALYs (Disability-Adjusted Life Years)	Global	Both	50-54 years	Prostate cancer	Smoking	Number	2009	11900.01663	18150.1398	5548.176805
DALYs (Disability-Adjusted Life Years)	Global	Both	50-54 years	Prostate cancer	Smoking	Rate	2009	3.520450964	5.369461167	1.641349335
DALYs (Disability-Adjusted Life Years)	Global	Both	50-54 years	Prostate cancer	Smoking	Number	2010	11811.75311	17972.32811	5469.59153
DALYs (Disability-Adjusted Life Years)	Global	Both	50-54 years	Prostate cancer	Smoking	Rate	2010	3.456121139	5.258706518	1.60040349
DALYs (Disability-Adjusted Life Years)	Global	Both	50-54 years	Prostate cancer	Smoking	Number	2011	11693.72379	18046.0182	5381.600217
DALYs (Disability-Adjusted Life Years)	Global	Both	50-54 years	Prostate cancer	Smoking	Rate	2011	3.365607559	5.193881466	1.548895348
DALYs (Disability-Adjusted Life Years)	Global	Both	50-54 years	Prostate cancer	Smoking	Number	2012	11672.7568	17976.94781	5381.175104
DALYs (Disability-Adjusted Life Years)	Global	Both	50-54 years	Prostate cancer	Smoking	Rate	2012	3.276138386	5.045506368	1.5103094

DALYs (Disability-Adjusted Life Years)	Global	Both	50-54 years	Prostate cancer	Smoking	Number	2013	11400.37041	17712.69483	5254.293042
DALYs (Disability-Adjusted Life Years)	Global	Both	50-54 years	Prostate cancer	Smoking	Rate	2013	3.089121811	4.799552118	1.423738936
DALYs (Disability-Adjusted Life Years)	Global	Both	50-54 years	Prostate cancer	Smoking	Number	2014	11356.2538	17469.45899	5236.595395
DALYs (Disability-Adjusted Life Years)	Global	Both	50-54 years	Prostate cancer	Smoking	Rate	2014	2.960054305	4.553486406	1.364940148
DALYs (Disability-Adjusted Life Years)	Global	Both	50-54 years	Prostate cancer	Smoking	Number	2015	11264.11237	17490.75025	5112.264805
DALYs (Disability-Adjusted Life Years)	Global	Both	50-54 years	Prostate cancer	Smoking	Rate	2015	2.829886393	4.394206529	1.284355849
DALYs (Disability-Adjusted Life Years)	Global	Both	50-54 years	Prostate cancer	Smoking	Number	2016	11326.01678	17600.77335	5167.686785
DALYs (Disability-Adjusted Life Years)	Global	Both	50-54 years	Prostate cancer	Smoking	Rate	2016	2.759714218	4.288630803	1.259166304
DALYs (Disability-Adjusted Life Years)	Global	Both	50-54 years	Prostate cancer	Smoking	Number	2017	11347.92018	17988.44694	5038.778783
DALYs (Disability-Adjusted Life Years)	Global	Both	50-54 years	Prostate cancer	Smoking	Rate	2017	2.695720146	4.273189979	1.196971538
DALYs (Disability-Adjusted Life Years)	Global	Both	50-54 years	Prostate cancer	Smoking	Number	2018	11255.58696	17249.44965	4993.68225

DALYs (Disability-Adjusted Life Years)	Global	Both	50-54 years	Prostate cancer	Smoking	Rate	2018	2.625636909	4.02384983	1.164896729
DALYs (Disability-Adjusted Life Years)	Global	Both	50-54 years	Prostate cancer	Smoking	Number	2019	11278.59254	17526.99619	4997.723705
DALYs (Disability-Adjusted Life Years)	Global	Both	50-54 years	Prostate cancer	Smoking	Rate	2019	2.59487661	4.032452834	1.149831089
DALYs (Disability-Adjusted Life Years)	Global	Both	50-54 years	Prostate cancer	Smoking	Number	2020	11226.5243	17396.24997	4849.763805
DALYs (Disability-Adjusted Life Years)	Global	Both	50-54 years	Prostate cancer	Smoking	Rate	2020	2.553492844	3.956807879	1.103087371
DALYs (Disability-Adjusted Life Years)	Global	Both	50-54 years	Prostate cancer	Smoking	Number	2021	11289.08722	17608.4898	5079.701519
DALYs (Disability-Adjusted Life Years)	Global	Both	50-54 years	Prostate cancer	Smoking	Rate	2021	2.537312672	3.957648958	1.141703557
DALYs (Disability-Adjusted Life Years)	Global	Both	55-59 years	Prostate cancer	Smoking	Number	1990	17749.67625	27286.74739	8287.704954
DALYs (Disability-Adjusted Life Years)	Global	Both	55-59 years	Prostate cancer	Smoking	Rate	1990	9.58406058	14.73366817	4.475003669
DALYs (Disability-Adjusted Life Years)	Global	Both	55-59 years	Prostate cancer	Smoking	Number	1991	17785.00874	27382.23478	8231.378279
DALYs (Disability-Adjusted Life Years)	Global	Both	55-59 years	Prostate cancer	Smoking	Rate	1991	9.468114621	14.57734102	4.382096977

DALYs (Disability-Adjusted Life Years)	Global	Both	55-59 years	Prostate cancer	Smoking	Number	1992	18090.97086	28074.56098	8459.167717
DALYs (Disability-Adjusted Life Years)	Global	Both	55-59 years	Prostate cancer	Smoking	Rate	1992	9.466562087	14.69073035	4.426475341
DALYs (Disability-Adjusted Life Years)	Global	Both	55-59 years	Prostate cancer	Smoking	Number	1993	18510.69282	28393.93459	8628.740731
DALYs (Disability-Adjusted Life Years)	Global	Both	55-59 years	Prostate cancer	Smoking	Rate	1993	9.529508228	14.61750978	4.44217062
DALYs (Disability-Adjusted Life Years)	Global	Both	55-59 years	Prostate cancer	Smoking	Number	1994	19041.93597	29296.93972	8970.152875
DALYs (Disability-Adjusted Life Years)	Global	Both	55-59 years	Prostate cancer	Smoking	Rate	1994	9.665772326	14.87125835	4.553289937
DALYs (Disability-Adjusted Life Years)	Global	Both	55-59 years	Prostate cancer	Smoking	Number	1995	19318.79664	29898.4237	8981.844478
DALYs (Disability-Adjusted Life Years)	Global	Both	55-59 years	Prostate cancer	Smoking	Rate	1995	9.681384619	14.98323859	4.501144279
DALYs (Disability-Adjusted Life Years)	Global	Both	55-59 years	Prostate cancer	Smoking	Number	1996	19589.72312	30242.43882	9072.250204
DALYs (Disability-Adjusted Life Years)	Global	Both	55-59 years	Prostate cancer	Smoking	Rate	1996	9.729388576	15.02014281	4.50580373
DALYs (Disability-Adjusted Life Years)	Global	Both	55-59 years	Prostate cancer	Smoking	Number	1997	19434.483	30074.22128	8983.422109

DALYs (Disability-Adjusted Life Years)	Global	Both	55-59 years	Prostate cancer	Smoking	Rate	1997	9.601499787	14.85800416	4.438210445
DALYs (Disability-Adjusted Life Years)	Global	Both	55-59 years	Prostate cancer	Smoking	Number	1998	19318.83633	29865.18988	8880.658972
DALYs (Disability-Adjusted Life Years)	Global	Both	55-59 years	Prostate cancer	Smoking	Rate	1998	9.507469116	14.69769533	4.370480162
DALYs (Disability-Adjusted Life Years)	Global	Both	55-59 years	Prostate cancer	Smoking	Number	1999	19025.61684	29465.62037	8770.845135
DALYs (Disability-Adjusted Life Years)	Global	Both	55-59 years	Prostate cancer	Smoking	Rate	1999	9.318744607	14.43225695	4.295958784
DALYs (Disability-Adjusted Life Years)	Global	Both	55-59 years	Prostate cancer	Smoking	Number	2000	18586.06881	28736.67396	8607.917022
DALYs (Disability-Adjusted Life Years)	Global	Both	55-59 years	Prostate cancer	Smoking	Rate	2000	9.064271945	14.01463807	4.198009894
DALYs (Disability-Adjusted Life Years)	Global	Both	55-59 years	Prostate cancer	Smoking	Number	2001	18698.7253	29144.24413	8662.669484
DALYs (Disability-Adjusted Life Years)	Global	Both	55-59 years	Prostate cancer	Smoking	Rate	2001	8.980590311	13.99734539	4.16049139
DALYs (Disability-Adjusted Life Years)	Global	Both	55-59 years	Prostate cancer	Smoking	Number	2002	19143.14054	29592.41604	8849.451601
DALYs (Disability-Adjusted Life Years)	Global	Both	55-59 years	Prostate cancer	Smoking	Rate	2002	8.844664877	13.67252162	4.088693473

DALYs (Disability-Adjusted Life Years)	Global	Both	55-59 years	Prostate cancer	Smoking	Number	2003	19670.94329	30438.37867	9145.755928
DALYs (Disability-Adjusted Life Years)	Global	Both	55-59 years	Prostate cancer	Smoking	Rate	2003	8.724973357	13.50082907	4.056565852
DALYs (Disability-Adjusted Life Years)	Global	Both	55-59 years	Prostate cancer	Smoking	Number	2004	20583.13198	31913.34101	9589.802898
DALYs (Disability-Adjusted Life Years)	Global	Both	55-59 years	Prostate cancer	Smoking	Rate	2004	8.644260142	13.40258722	4.027411914
DALYs (Disability-Adjusted Life Years)	Global	Both	55-59 years	Prostate cancer	Smoking	Number	2005	21679.31242	33467.50746	10078.5186
DALYs (Disability-Adjusted Life Years)	Global	Both	55-59 years	Prostate cancer	Smoking	Rate	2005	8.718726784	13.45956219	4.053258165
DALYs (Disability-Adjusted Life Years)	Global	Both	55-59 years	Prostate cancer	Smoking	Number	2006	22426.92878	34525.17599	10370.78799
DALYs (Disability-Adjusted Life Years)	Global	Both	55-59 years	Prostate cancer	Smoking	Rate	2006	8.573761674	13.19889288	3.964727648
DALYs (Disability-Adjusted Life Years)	Global	Both	55-59 years	Prostate cancer	Smoking	Number	2007	22411.48904	34496.14344	10259.89307
DALYs (Disability-Adjusted Life Years)	Global	Both	55-59 years	Prostate cancer	Smoking	Rate	2007	8.271171779	12.73112766	3.786510475
DALYs (Disability-Adjusted Life Years)	Global	Both	55-59 years	Prostate cancer	Smoking	Number	2008	22745.99962	34724.87808	10480.39141

DALYs (Disability-Adjusted Life Years)	Global	Both	55-59 years	Prostate cancer	Smoking	Rate	2008	8.08341033	12.34043097	3.724492465
DALYs (Disability-Adjusted Life Years)	Global	Both	55-59 years	Prostate cancer	Smoking	Number	2009	22807.73726	34878.03387	10389.86149
DALYs (Disability-Adjusted Life Years)	Global	Both	55-59 years	Prostate cancer	Smoking	Rate	2009	7.86498891	12.02729347	3.582825621
DALYs (Disability-Adjusted Life Years)	Global	Both	55-59 years	Prostate cancer	Smoking	Number	2010	23150.83787	35201.33769	10604.42947
DALYs (Disability-Adjusted Life Years)	Global	Both	55-59 years	Prostate cancer	Smoking	Rate	2010	7.662599064	11.65114363	3.509915788
DALYs (Disability-Adjusted Life Years)	Global	Both	55-59 years	Prostate cancer	Smoking	Number	2011	23268.39294	35646.50901	10685.05457
DALYs (Disability-Adjusted Life Years)	Global	Both	55-59 years	Prostate cancer	Smoking	Rate	2011	7.502902863	11.49423148	3.445400237
DALYs (Disability-Adjusted Life Years)	Global	Both	55-59 years	Prostate cancer	Smoking	Number	2012	23425.96204	35718.88338	10662.98322
DALYs (Disability-Adjusted Life Years)	Global	Both	55-59 years	Prostate cancer	Smoking	Rate	2012	7.391576688	11.27035318	3.364483305
DALYs (Disability-Adjusted Life Years)	Global	Both	55-59 years	Prostate cancer	Smoking	Number	2013	23209.41036	35602.69439	10564.6469
DALYs (Disability-Adjusted Life Years)	Global	Both	55-59 years	Prostate cancer	Smoking	Rate	2013	7.211130925	11.06170672	3.282420828

DALYs (Disability-Adjusted Life Years)	Global	Both	55-59 years	Prostate cancer	Smoking	Number	2014	23210.8525	35604.25955	10611.49594
DALYs (Disability-Adjusted Life Years)	Global	Both	55-59 years	Prostate cancer	Smoking	Rate	2014	7.130725317	10.93816761	3.260012217
DALYs (Disability-Adjusted Life Years)	Global	Both	55-59 years	Prostate cancer	Smoking	Number	2015	23244.34265	35364.43483	10759.01477
DALYs (Disability-Adjusted Life Years)	Global	Both	55-59 years	Prostate cancer	Smoking	Rate	2015	7.060684028	10.7422741	3.268150228
DALYs (Disability-Adjusted Life Years)	Global	Both	55-59 years	Prostate cancer	Smoking	Number	2016	23418.95661	35985.02265	10705.5431
DALYs (Disability-Adjusted Life Years)	Global	Both	55-59 years	Prostate cancer	Smoking	Rate	2016	6.99459582	10.74773284	3.197450178
DALYs (Disability-Adjusted Life Years)	Global	Both	55-59 years	Prostate cancer	Smoking	Number	2017	23338.27676	36209.75809	10569.86062
DALYs (Disability-Adjusted Life Years)	Global	Both	55-59 years	Prostate cancer	Smoking	Rate	2017	6.794859448	10.54234721	3.077378765
DALYs (Disability-Adjusted Life Years)	Global	Both	55-59 years	Prostate cancer	Smoking	Number	2018	23515.26635	36354.92008	10757.83259
DALYs (Disability-Adjusted Life Years)	Global	Both	55-59 years	Prostate cancer	Smoking	Rate	2018	6.606831083	10.21425028	3.02251234
DALYs (Disability-Adjusted Life Years)	Global	Both	55-59 years	Prostate cancer	Smoking	Number	2019	23726.7897	36856.54063	10974.39943

DALYs (Disability-Adjusted Life Years)	Global	Both	55-59 years	Prostate cancer	Smoking	Rate	2019	6.409315393	9.956053732	2.96451345
DALYs (Disability-Adjusted Life Years)	Global	Both	55-59 years	Prostate cancer	Smoking	Number	2020	23626.38139	36418.22224	10783.59193
DALYs (Disability-Adjusted Life Years)	Global	Both	55-59 years	Prostate cancer	Smoking	Rate	2020	6.150972236	9.481243454	2.807436885
DALYs (Disability-Adjusted Life Years)	Global	Both	55-59 years	Prostate cancer	Smoking	Number	2021	23772.89964	36539.88952	11000.7612
DALYs (Disability-Adjusted Life Years)	Global	Both	55-59 years	Prostate cancer	Smoking	Rate	2021	6.007383708	9.233586998	2.779879384
DALYs (Disability-Adjusted Life Years)	Global	Both	60-64 years	Prostate cancer	Smoking	Number	1990	35636.67774	54614.19894	16939.20181
DALYs (Disability-Adjusted Life Years)	Global	Both	60-64 years	Prostate cancer	Smoking	Rate	1990	22.18843602	34.00439479	10.54684161
DALYs (Disability-Adjusted Life Years)	Global	Both	60-64 years	Prostate cancer	Smoking	Number	1991	36179.36686	55644.13002	17129.70795
DALYs (Disability-Adjusted Life Years)	Global	Both	60-64 years	Prostate cancer	Smoking	Rate	1991	22.07205666	33.94698408	10.45037316
DALYs (Disability-Adjusted Life Years)	Global	Both	60-64 years	Prostate cancer	Smoking	Number	1992	36393.6229	55782.85433	17176.91871
DALYs (Disability-Adjusted Life Years)	Global	Both	60-64 years	Prostate cancer	Smoking	Rate	1992	21.8845804	33.54390861	10.32899801

DALYs (Disability-Adjusted Life Years)	Global	Both	60-64 years	Prostate cancer	Smoking	Number	1993	36200.9956	55428.0325	17144.76526
DALYs (Disability-Adjusted Life Years)	Global	Both	60-64 years	Prostate cancer	Smoking	Rate	1993	21.51544894	32.94271285	10.18970101
DALYs (Disability-Adjusted Life Years)	Global	Both	60-64 years	Prostate cancer	Smoking	Number	1994	36064.95347	55110.39259	17111.28785
DALYs (Disability-Adjusted Life Years)	Global	Both	60-64 years	Prostate cancer	Smoking	Rate	1994	21.22534684	32.43418013	10.07052511
DALYs (Disability-Adjusted Life Years)	Global	Both	60-64 years	Prostate cancer	Smoking	Number	1995	35646.66574	54776.42092	16915.06745
DALYs (Disability-Adjusted Life Years)	Global	Both	60-64 years	Prostate cancer	Smoking	Rate	1995	20.75005369	31.88555371	9.846322239
DALYs (Disability-Adjusted Life Years)	Global	Both	60-64 years	Prostate cancer	Smoking	Number	1996	35412.41565	54968.731	16664.00108
DALYs (Disability-Adjusted Life Years)	Global	Both	60-64 years	Prostate cancer	Smoking	Rate	1996	20.31691166	31.53681644	9.560518017
DALYs (Disability-Adjusted Life Years)	Global	Both	60-64 years	Prostate cancer	Smoking	Number	1997	35129.54887	54342.75636	16538.88682
DALYs (Disability-Adjusted Life Years)	Global	Both	60-64 years	Prostate cancer	Smoking	Rate	1997	19.79042713	30.61429464	9.31727406
DALYs (Disability-Adjusted Life Years)	Global	Both	60-64 years	Prostate cancer	Smoking	Number	1998	35591.67691	55297.42425	16600.08235

DALYs (Disability-Adjusted Life Years)	Global	Both	60-64 years	Prostate cancer	Smoking	Rate	1998	19.69867978	30.60508376	9.187533012
DALYs (Disability-Adjusted Life Years)	Global	Both	60-64 years	Prostate cancer	Smoking	Number	1999	35920.09199	55872.78498	16716.82891
DALYs (Disability-Adjusted Life Years)	Global	Both	60-64 years	Prostate cancer	Smoking	Rate	1999	19.56669243	30.43548994	9.106130621
DALYs (Disability-Adjusted Life Years)	Global	Both	60-64 years	Prostate cancer	Smoking	Number	2000	36292.3397	56323.39656	16913.24246
DALYs (Disability-Adjusted Life Years)	Global	Both	60-64 years	Prostate cancer	Smoking	Rate	2000	19.47734041	30.22759009	9.076983838
DALYs (Disability-Adjusted Life Years)	Global	Both	60-64 years	Prostate cancer	Smoking	Number	2001	36427.37808	56613.51988	16963.88975
DALYs (Disability-Adjusted Life Years)	Global	Both	60-64 years	Prostate cancer	Smoking	Rate	2001	19.33232686	30.04528815	9.00288406
DALYs (Disability-Adjusted Life Years)	Global	Both	60-64 years	Prostate cancer	Smoking	Number	2002	36442.88502	56350.24324	17052.58871
DALYs (Disability-Adjusted Life Years)	Global	Both	60-64 years	Prostate cancer	Smoking	Rate	2002	19.19582423	29.68177092	8.982233304
DALYs (Disability-Adjusted Life Years)	Global	Both	60-64 years	Prostate cancer	Smoking	Number	2003	35822.99301	55734.28719	16830.95951
DALYs (Disability-Adjusted Life Years)	Global	Both	60-64 years	Prostate cancer	Smoking	Rate	2003	18.74325306	29.16121076	8.806269573

DALYs (Disability-Adjusted Life Years)	Global	Both	60-64 years	Prostate cancer	Smoking	Number	2004	35014.90982	54261.03763	16376.44062
DALYs (Disability-Adjusted Life Years)	Global	Both	60-64 years	Prostate cancer	Smoking	Rate	2004	18.1710789	28.15890719	8.49859663
DALYs (Disability-Adjusted Life Years)	Global	Both	60-64 years	Prostate cancer	Smoking	Number	2005	34161.71081	53350.29731	15910.78434
DALYs (Disability-Adjusted Life Years)	Global	Both	60-64 years	Prostate cancer	Smoking	Rate	2005	17.5853245	27.46297735	8.19034817
DALYs (Disability-Adjusted Life Years)	Global	Both	60-64 years	Prostate cancer	Smoking	Number	2006	33623.06468	52470.44853	15524.34945
DALYs (Disability-Adjusted Life Years)	Global	Both	60-64 years	Prostate cancer	Smoking	Rate	2006	16.98605666	26.50757807	7.842755618
DALYs (Disability-Adjusted Life Years)	Global	Both	60-64 years	Prostate cancer	Smoking	Number	2007	34186.79525	53255.80647	15862.54005
DALYs (Disability-Adjusted Life Years)	Global	Both	60-64 years	Prostate cancer	Smoking	Rate	2007	16.55379424	25.78731512	7.680896151
DALYs (Disability-Adjusted Life Years)	Global	Both	60-64 years	Prostate cancer	Smoking	Number	2008	35338.17253	55292.35234	16329.79716
DALYs (Disability-Adjusted Life Years)	Global	Both	60-64 years	Prostate cancer	Smoking	Rate	2008	16.38269335	25.633404	7.57045541
DALYs (Disability-Adjusted Life Years)	Global	Both	60-64 years	Prostate cancer	Smoking	Number	2009	36748.85213	57484.72658	16973.34707

DALYs (Disability-Adjusted Life Years)	Global	Both	60-64 years	Prostate cancer	Smoking	Rate	2009	16.09943956	25.18369494	7.435915942
DALYs (Disability-Adjusted Life Years)	Global	Both	60-64 years	Prostate cancer	Smoking	Number	2010	38365.24458	59567.75407	17686.67196
DALYs (Disability-Adjusted Life Years)	Global	Both	60-64 years	Prostate cancer	Smoking	Rate	2010	16.07818534	24.96377649	7.41216674
DALYs (Disability-Adjusted Life Years)	Global	Both	60-64 years	Prostate cancer	Smoking	Number	2011	39875.50976	62830.30732	18578.26552
DALYs (Disability-Adjusted Life Years)	Global	Both	60-64 years	Prostate cancer	Smoking	Rate	2011	15.86866812	25.00365013	7.393318141
DALYs (Disability-Adjusted Life Years)	Global	Both	60-64 years	Prostate cancer	Smoking	Number	2012	40200.81834	63195.67638	18423.71517
DALYs (Disability-Adjusted Life Years)	Global	Both	60-64 years	Prostate cancer	Smoking	Rate	2012	15.43504519	24.26388719	7.073758398
DALYs (Disability-Adjusted Life Years)	Global	Both	60-64 years	Prostate cancer	Smoking	Number	2013	40326.21604	63656.59526	18594.79446
DALYs (Disability-Adjusted Life Years)	Global	Both	60-64 years	Prostate cancer	Smoking	Rate	2013	14.90401227	23.52659808	6.872379112
DALYs (Disability-Adjusted Life Years)	Global	Both	60-64 years	Prostate cancer	Smoking	Number	2014	40718.46541	64262.95779	18828.63368
DALYs (Disability-Adjusted Life Years)	Global	Both	60-64 years	Prostate cancer	Smoking	Rate	2014	14.59955708	23.04140666	6.75098409

DALYs (Disability-Adjusted Life Years)	Global	Both	60-64 years	Prostate cancer	Smoking	Number	2015	41401.79185	65168.49827	19134.62052
DALYs (Disability-Adjusted Life Years)	Global	Both	60-64 years	Prostate cancer	Smoking	Rate	2015	14.25017671	22.43049334	6.585988468
DALYs (Disability-Adjusted Life Years)	Global	Both	60-64 years	Prostate cancer	Smoking	Number	2016	42079.321	65696.18851	19471.09821
DALYs (Disability-Adjusted Life Years)	Global	Both	60-64 years	Prostate cancer	Smoking	Rate	2016	14.11550003	22.03777363	6.531576098
DALYs (Disability-Adjusted Life Years)	Global	Both	60-64 years	Prostate cancer	Smoking	Number	2017	42777.24489	66653.05018	19523.50817
DALYs (Disability-Adjusted Life Years)	Global	Both	60-64 years	Prostate cancer	Smoking	Rate	2017	14.04900282	21.89035063	6.411956216
DALYs (Disability-Adjusted Life Years)	Global	Both	60-64 years	Prostate cancer	Smoking	Number	2018	43308.28166	68364.96898	19807.41205
DALYs (Disability-Adjusted Life Years)	Global	Both	60-64 years	Prostate cancer	Smoking	Rate	2018	14.01474277	22.12319256	6.409762156
DALYs (Disability-Adjusted Life Years)	Global	Both	60-64 years	Prostate cancer	Smoking	Number	2019	43868.57225	68633.71139	20056.08837
DALYs (Disability-Adjusted Life Years)	Global	Both	60-64 years	Prostate cancer	Smoking	Rate	2019	14.04655602	21.97626278	6.421885971
DALYs (Disability-Adjusted Life Years)	Global	Both	60-64 years	Prostate cancer	Smoking	Number	2020	43738.80954	68359.68335	20157.89108

DALYs (Disability-Adjusted Life Years)	Global	Both	60-64 years	Prostate cancer	Smoking	Rate	2020	13.86589315	21.67109885	6.390369716
DALYs (Disability-Adjusted Life Years)	Global	Both	60-64 years	Prostate cancer	Smoking	Number	2021	43915.71765	67834.53979	20348.96466
DALYs (Disability-Adjusted Life Years)	Global	Both	60-64 years	Prostate cancer	Smoking	Rate	2021	13.72160981	21.19512413	6.358100655
DALYs (Disability-Adjusted Life Years)	Global	Both	65-69 years	Prostate cancer	Smoking	Number	1990	44108.26959	69196.00907	21178.63158
DALYs (Disability-Adjusted Life Years)	Global	Both	65-69 years	Prostate cancer	Smoking	Rate	1990	35.68357191	55.9795428	17.13350421
DALYs (Disability-Adjusted Life Years)	Global	Both	65-69 years	Prostate cancer	Smoking	Number	1991	44968.54253	70321.64278	21454.83707
DALYs (Disability-Adjusted Life Years)	Global	Both	65-69 years	Prostate cancer	Smoking	Rate	1991	35.28896129	55.18474895	16.83663451
DALYs (Disability-Adjusted Life Years)	Global	Both	65-69 years	Prostate cancer	Smoking	Number	1992	46064.56464	71966.35782	21924.44948
DALYs (Disability-Adjusted Life Years)	Global	Both	65-69 years	Prostate cancer	Smoking	Rate	1992	35.0871415	54.81640388	16.69974018
DALYs (Disability-Adjusted Life Years)	Global	Both	65-69 years	Prostate cancer	Smoking	Number	1993	47211.33192	74263.73871	22436.57548
DALYs (Disability-Adjusted Life Years)	Global	Both	65-69 years	Prostate cancer	Smoking	Rate	1993	34.93464082	54.95242207	16.60223667

DALYs (Disability-Adjusted Life Years)	Global	Both	65-69 years	Prostate cancer	Smoking	Number	1994	48108.25598	75404.24192	22961.96831
DALYs (Disability-Adjusted Life Years)	Global	Both	65-69 years	Prostate cancer	Smoking	Rate	1994	34.64904243	54.30844923	16.53791429
DALYs (Disability-Adjusted Life Years)	Global	Both	65-69 years	Prostate cancer	Smoking	Number	1995	48453.28893	75840.68198	22991.32319
DALYs (Disability-Adjusted Life Years)	Global	Both	65-69 years	Prostate cancer	Smoking	Rate	1995	34.12000792	53.40575897	16.19011107
DALYs (Disability-Adjusted Life Years)	Global	Both	65-69 years	Prostate cancer	Smoking	Number	1996	48515.75225	76072.52332	23041.415
DALYs (Disability-Adjusted Life Years)	Global	Both	65-69 years	Prostate cancer	Smoking	Rate	1996	33.49057664	52.51310253	15.90556137
DALYs (Disability-Adjusted Life Years)	Global	Both	65-69 years	Prostate cancer	Smoking	Number	1997	47815.55041	75673.16986	22708.59848
DALYs (Disability-Adjusted Life Years)	Global	Both	65-69 years	Prostate cancer	Smoking	Rate	1997	32.53311947	51.48710523	15.45065446
DALYs (Disability-Adjusted Life Years)	Global	Both	65-69 years	Prostate cancer	Smoking	Number	1998	46889.1653	73875.57975	22354.23233
DALYs (Disability-Adjusted Life Years)	Global	Both	65-69 years	Prostate cancer	Smoking	Rate	1998	31.51269799	49.64939807	15.02355966
DALYs (Disability-Adjusted Life Years)	Global	Both	65-69 years	Prostate cancer	Smoking	Number	1999	45843.9477	71881.96196	21794.37878

DALYs (Disability-Adjusted Life Years)	Global	Both	65-69 years	Prostate cancer	Smoking	Rate	1999	30.46031742	47.76088204	14.48094523
DALYs (Disability-Adjusted Life Years)	Global	Both	65-69 years	Prostate cancer	Smoking	Number	2000	45108.76262	70723.96516	21373.38079
DALYs (Disability-Adjusted Life Years)	Global	Both	65-69 years	Prostate cancer	Smoking	Rate	2000	29.56862678	46.35929714	14.01017192
DALYs (Disability-Adjusted Life Years)	Global	Both	65-69 years	Prostate cancer	Smoking	Number	2001	44732.8472	69630.83081	21157.51618
DALYs (Disability-Adjusted Life Years)	Global	Both	65-69 years	Prostate cancer	Smoking	Rate	2001	28.81401916	44.85169666	13.62830928
DALYs (Disability-Adjusted Life Years)	Global	Both	65-69 years	Prostate cancer	Smoking	Number	2002	44899.89028	69505.77051	21221.94324
DALYs (Disability-Adjusted Life Years)	Global	Both	65-69 years	Prostate cancer	Smoking	Rate	2002	28.31508	43.83221072	13.38312893
DALYs (Disability-Adjusted Life Years)	Global	Both	65-69 years	Prostate cancer	Smoking	Number	2003	45105.83557	70353.44693	21142.66682
DALYs (Disability-Adjusted Life Years)	Global	Both	65-69 years	Prostate cancer	Smoking	Rate	2003	27.86680832	43.46501944	13.06213788
DALYs (Disability-Adjusted Life Years)	Global	Both	65-69 years	Prostate cancer	Smoking	Number	2004	45087.00219	70574.66891	21071.94783
DALYs (Disability-Adjusted Life Years)	Global	Both	65-69 years	Prostate cancer	Smoking	Rate	2004	27.3431195	42.80017548	12.77913278

DALYs (Disability-Adjusted Life Years)	Global	Both	65-69 years	Prostate cancer	Smoking	Number	2005	45020.02887	70151.66381	20894.71585
DALYs (Disability-Adjusted Life Years)	Global	Both	65-69 years	Prostate cancer	Smoking	Rate	2005	26.82834835	41.80479936	12.45158499
DALYs (Disability-Adjusted Life Years)	Global	Both	65-69 years	Prostate cancer	Smoking	Number	2006	44462.48292	68789.44424	20625.07342
DALYs (Disability-Adjusted Life Years)	Global	Both	65-69 years	Prostate cancer	Smoking	Rate	2006	26.11797632	40.40802397	12.11549927
DALYs (Disability-Adjusted Life Years)	Global	Both	65-69 years	Prostate cancer	Smoking	Number	2007	43526.36612	67341.29679	20232.17708
DALYs (Disability-Adjusted Life Years)	Global	Both	65-69 years	Prostate cancer	Smoking	Rate	2007	25.28728601	39.12292213	11.75418244
DALYs (Disability-Adjusted Life Years)	Global	Both	65-69 years	Prostate cancer	Smoking	Number	2008	42869.88106	66532.84524	20068.10817
DALYs (Disability-Adjusted Life Years)	Global	Both	65-69 years	Prostate cancer	Smoking	Rate	2008	24.64383019	38.24652879	11.53618899
DALYs (Disability-Adjusted Life Years)	Global	Both	65-69 years	Prostate cancer	Smoking	Number	2009	42099.18445	65268.62687	19749.20248
DALYs (Disability-Adjusted Life Years)	Global	Both	65-69 years	Prostate cancer	Smoking	Rate	2009	23.91335285	37.07415536	11.21802366
DALYs (Disability-Adjusted Life Years)	Global	Both	65-69 years	Prostate cancer	Smoking	Number	2010	41437.44275	64433.34801	19367.45518

DALYs (Disability-Adjusted Life Years)	Global	Both	65-69 years	Prostate cancer	Smoking	Rate	2010	23.27421511	36.1903511	10.87814035
DALYs (Disability-Adjusted Life Years)	Global	Both	65-69 years	Prostate cancer	Smoking	Number	2011	41123.38279	64512.25711	19140.34649
DALYs (Disability-Adjusted Life Years)	Global	Both	65-69 years	Prostate cancer	Smoking	Rate	2011	22.61332931	35.47463306	10.52508157
DALYs (Disability-Adjusted Life Years)	Global	Both	65-69 years	Prostate cancer	Smoking	Number	2012	41884.23159	65382.69672	19562.24211
DALYs (Disability-Adjusted Life Years)	Global	Both	65-69 years	Prostate cancer	Smoking	Rate	2012	22.01598534	34.3676949	10.28267725
DALYs (Disability-Adjusted Life Years)	Global	Both	65-69 years	Prostate cancer	Smoking	Number	2013	42674.75757	67319.98143	20121.31294
DALYs (Disability-Adjusted Life Years)	Global	Both	65-69 years	Prostate cancer	Smoking	Rate	2013	21.43258595	33.81018125	10.10554702
DALYs (Disability-Adjusted Life Years)	Global	Both	65-69 years	Prostate cancer	Smoking	Number	2014	44602.20126	70654.35559	20879.42243
DALYs (Disability-Adjusted Life Years)	Global	Both	65-69 years	Prostate cancer	Smoking	Rate	2014	21.12536414	33.4646934	9.889319125
DALYs (Disability-Adjusted Life Years)	Global	Both	65-69 years	Prostate cancer	Smoking	Number	2015	46440.02598	73165.45363	21863.72798
DALYs (Disability-Adjusted Life Years)	Global	Both	65-69 years	Prostate cancer	Smoking	Rate	2015	21.01624468	33.11072816	9.89434108

DALYs (Disability-Adjusted Life Years)	Global	Both	65-69 years	Prostate cancer	Smoking	Number	2016	48770.8677	76799.3001	22800.37773
DALYs (Disability-Adjusted Life Years)	Global	Both	65-69 years	Prostate cancer	Smoking	Rate	2016	20.93018537	32.95868339	9.784860406
DALYs (Disability-Adjusted Life Years)	Global	Both	65-69 years	Prostate cancer	Smoking	Number	2017	50177.60111	78405.0467	23485.36059
DALYs (Disability-Adjusted Life Years)	Global	Both	65-69 years	Prostate cancer	Smoking	Rate	2017	20.75793934	32.43533305	9.715643628
DALYs (Disability-Adjusted Life Years)	Global	Both	65-69 years	Prostate cancer	Smoking	Number	2018	51327.24684	81457.80472	23998.59811
DALYs (Disability-Adjusted Life Years)	Global	Both	65-69 years	Prostate cancer	Smoking	Rate	2018	20.4293286	32.42192719	9.551949053
DALYs (Disability-Adjusted Life Years)	Global	Both	65-69 years	Prostate cancer	Smoking	Number	2019	52230.87589	82204.98423	24586.50728
DALYs (Disability-Adjusted Life Years)	Global	Both	65-69 years	Prostate cancer	Smoking	Rate	2019	20.16335133	31.73463874	9.491443054
DALYs (Disability-Adjusted Life Years)	Global	Both	65-69 years	Prostate cancer	Smoking	Number	2020	53163.95882	83949.73045	24815.84481
DALYs (Disability-Adjusted Life Years)	Global	Both	65-69 years	Prostate cancer	Smoking	Rate	2020	19.72268921	31.14355063	9.206146524
DALYs (Disability-Adjusted Life Years)	Global	Both	65-69 years	Prostate cancer	Smoking	Number	2021	53761.45585	83908.22702	25164.04802

DALYs (Disability-Adjusted Life Years)	Global	Both	65-69 years	Prostate cancer	Smoking	Rate	2021	19.48993444	30.41892779	9.122625837
DALYs (Disability-Adjusted Life Years)	Global	Both	70-74 years	Prostate cancer	Smoking	Number	1990	38754.84272	61277.07007	18215.05694
DALYs (Disability-Adjusted Life Years)	Global	Both	70-74 years	Prostate cancer	Smoking	Rate	1990	45.77641651	72.37920438	21.51524751
DALYs (Disability-Adjusted Life Years)	Global	Both	70-74 years	Prostate cancer	Smoking	Number	1991	40297.68154	64191.17303	19017.11136
DALYs (Disability-Adjusted Life Years)	Global	Both	70-74 years	Prostate cancer	Smoking	Rate	1991	45.93411332	73.16958454	21.67703239
DALYs (Disability-Adjusted Life Years)	Global	Both	70-74 years	Prostate cancer	Smoking	Number	1992	42179.00788	67284.1126	19922.01809
DALYs (Disability-Adjusted Life Years)	Global	Both	70-74 years	Prostate cancer	Smoking	Rate	1992	46.14525611	73.61108675	21.79535919
DALYs (Disability-Adjusted Life Years)	Global	Both	70-74 years	Prostate cancer	Smoking	Number	1993	44080.80675	70206.44397	20975.67977
DALYs (Disability-Adjusted Life Years)	Global	Both	70-74 years	Prostate cancer	Smoking	Rate	1993	46.21113347	73.59936426	21.98938742
DALYs (Disability-Adjusted Life Years)	Global	Both	70-74 years	Prostate cancer	Smoking	Number	1994	45751.32348	73120.86978	21658.29522
DALYs (Disability-Adjusted Life Years)	Global	Both	70-74 years	Prostate cancer	Smoking	Rate	1994	46.11674442	73.70489435	21.83128245

DALYs (Disability-Adjusted Life Years)	Global	Both	70-74 years	Prostate cancer	Smoking	Number	1995	46347.50883	73559.99888	21868.83313
DALYs (Disability-Adjusted Life Years)	Global	Both	70-74 years	Prostate cancer	Smoking	Rate	1995	45.13186996	71.63060945	21.29524019
DALYs (Disability-Adjusted Life Years)	Global	Both	70-74 years	Prostate cancer	Smoking	Number	1996	46525.88649	73629.77137	22065.07014
DALYs (Disability-Adjusted Life Years)	Global	Both	70-74 years	Prostate cancer	Smoking	Rate	1996	43.90466351	69.481542	20.82194565
DALYs (Disability-Adjusted Life Years)	Global	Both	70-74 years	Prostate cancer	Smoking	Number	1997	46382.96066	73045.53098	22085.38775
DALYs (Disability-Adjusted Life Years)	Global	Both	70-74 years	Prostate cancer	Smoking	Rate	1997	42.41930936	66.80343238	20.19808313
DALYs (Disability-Adjusted Life Years)	Global	Both	70-74 years	Prostate cancer	Smoking	Number	1998	46725.19433	73667.62116	22245.40247
DALYs (Disability-Adjusted Life Years)	Global	Both	70-74 years	Prostate cancer	Smoking	Rate	1998	41.434119	65.32563482	19.72637388
DALYs (Disability-Adjusted Life Years)	Global	Both	70-74 years	Prostate cancer	Smoking	Number	1999	46972.35054	73579.81516	22366.74636
DALYs (Disability-Adjusted Life Years)	Global	Both	70-74 years	Prostate cancer	Smoking	Rate	1999	40.44679841	63.35786727	19.25948501
DALYs (Disability-Adjusted Life Years)	Global	Both	70-74 years	Prostate cancer	Smoking	Number	2000	46995.61128	74307.32673	22474.32268

DALYs (Disability-Adjusted Life Years)	Global	Both	70-74 years	Prostate cancer	Smoking	Rate	2000	39.45300268	62.38129648	18.86728333
DALYs (Disability-Adjusted Life Years)	Global	Both	70-74 years	Prostate cancer	Smoking	Number	2001	47166.9418	74309.00863	22521.88159
DALYs (Disability-Adjusted Life Years)	Global	Both	70-74 years	Prostate cancer	Smoking	Rate	2001	38.70099304	60.97135654	18.47945085
DALYs (Disability-Adjusted Life Years)	Global	Both	70-74 years	Prostate cancer	Smoking	Number	2002	46874.78632	73909.37528	22311.07392
DALYs (Disability-Adjusted Life Years)	Global	Both	70-74 years	Prostate cancer	Smoking	Rate	2002	37.80129355	59.6028315	17.99234772
DALYs (Disability-Adjusted Life Years)	Global	Both	70-74 years	Prostate cancer	Smoking	Number	2003	46152.36909	73000.07712	21859.80862
DALYs (Disability-Adjusted Life Years)	Global	Both	70-74 years	Prostate cancer	Smoking	Rate	2003	36.65513075	57.97811519	17.36149539
DALYs (Disability-Adjusted Life Years)	Global	Both	70-74 years	Prostate cancer	Smoking	Number	2004	45116.74764	71666.89367	21137.52024
DALYs (Disability-Adjusted Life Years)	Global	Both	70-74 years	Prostate cancer	Smoking	Rate	2004	35.30586312	56.08253411	16.5410504
DALYs (Disability-Adjusted Life Years)	Global	Both	70-74 years	Prostate cancer	Smoking	Number	2005	44214.65614	70347.51212	20656.24397
DALYs (Disability-Adjusted Life Years)	Global	Both	70-74 years	Prostate cancer	Smoking	Rate	2005	34.00679918	54.1063513	15.88732791

DALYs (Disability-Adjusted Life Years)	Global	Both	70-74 years	Prostate cancer	Smoking	Number	2006	43328.46049	68410.4608	20281.51869
DALYs (Disability-Adjusted Life Years)	Global	Both	70-74 years	Prostate cancer	Smoking	Rate	2006	32.61315169	51.49226883	15.26581462
DALYs (Disability-Adjusted Life Years)	Global	Both	70-74 years	Prostate cancer	Smoking	Number	2007	43160.63961	68481.25353	20171.26414
DALYs (Disability-Adjusted Life Years)	Global	Both	70-74 years	Prostate cancer	Smoking	Rate	2007	31.67368279	50.25536047	14.80279782
DALYs (Disability-Adjusted Life Years)	Global	Both	70-74 years	Prostate cancer	Smoking	Number	2008	43393.73084	68998.26958	20340.97412
DALYs (Disability-Adjusted Life Years)	Global	Both	70-74 years	Prostate cancer	Smoking	Rate	2008	31.07891253	49.41707347	14.56835684
DALYs (Disability-Adjusted Life Years)	Global	Both	70-74 years	Prostate cancer	Smoking	Number	2009	43568.18043	69439.40828	20427.67947
DALYs (Disability-Adjusted Life Years)	Global	Both	70-74 years	Prostate cancer	Smoking	Rate	2009	30.52518338	48.65134717	14.31224934
DALYs (Disability-Adjusted Life Years)	Global	Both	70-74 years	Prostate cancer	Smoking	Number	2010	43788.1031	69467.37024	20364.88321
DALYs (Disability-Adjusted Life Years)	Global	Both	70-74 years	Prostate cancer	Smoking	Rate	2010	30.04805737	47.66955813	13.97469029
DALYs (Disability-Adjusted Life Years)	Global	Both	70-74 years	Prostate cancer	Smoking	Number	2011	43622.1762	70021.56919	20297.9184

DALYs (Disability-Adjusted Life Years)	Global	Both	70-74 years	Prostate cancer	Smoking	Rate	2011	29.41469897	47.21597037	13.68700994
DALYs (Disability-Adjusted Life Years)	Global	Both	70-74 years	Prostate cancer	Smoking	Number	2012	42948.78779	68545.61932	20023.44563
DALYs (Disability-Adjusted Life Years)	Global	Both	70-74 years	Prostate cancer	Smoking	Rate	2012	28.56705859	45.59259583	13.31844213
DALYs (Disability-Adjusted Life Years)	Global	Both	70-74 years	Prostate cancer	Smoking	Number	2013	42004.54354	67016.21929	19346.34149
DALYs (Disability-Adjusted Life Years)	Global	Both	70-74 years	Prostate cancer	Smoking	Rate	2013	27.56771471	43.98295657	12.69706508
DALYs (Disability-Adjusted Life Years)	Global	Both	70-74 years	Prostate cancer	Smoking	Number	2014	41939.12026	68010.46028	19286.2756
DALYs (Disability-Adjusted Life Years)	Global	Both	70-74 years	Prostate cancer	Smoking	Rate	2014	27.11583587	43.97232148	12.4695864
DALYs (Disability-Adjusted Life Years)	Global	Both	70-74 years	Prostate cancer	Smoking	Number	2015	41779.51565	66998.22604	19312.4986
DALYs (Disability-Adjusted Life Years)	Global	Both	70-74 years	Prostate cancer	Smoking	Rate	2015	26.63461448	42.71164693	12.31179794
DALYs (Disability-Adjusted Life Years)	Global	Both	70-74 years	Prostate cancer	Smoking	Number	2016	42346.84984	67804.34241	19445.18948
DALYs (Disability-Adjusted Life Years)	Global	Both	70-74 years	Prostate cancer	Smoking	Rate	2016	26.36312319	42.21174039	12.10564488

DALYs (Disability-Adjusted Life Years)	Global	Both	70-74 years	Prostate cancer	Smoking	Number	2017	44200.05478	71203.28257	20184.07163
DALYs (Disability-Adjusted Life Years)	Global	Both	70-74 years	Prostate cancer	Smoking	Rate	2017	26.23057791	42.2556773	11.97826261
DALYs (Disability-Adjusted Life Years)	Global	Both	70-74 years	Prostate cancer	Smoking	Number	2018	46241.01988	75552.98521	21431.31486
DALYs (Disability-Adjusted Life Years)	Global	Both	70-74 years	Prostate cancer	Smoking	Rate	2018	26.17574757	42.76843102	12.13166771
DALYs (Disability-Adjusted Life Years)	Global	Both	70-74 years	Prostate cancer	Smoking	Number	2019	48745.85409	78859.61173	22316.15704
DALYs (Disability-Adjusted Life Years)	Global	Both	70-74 years	Prostate cancer	Smoking	Rate	2019	25.98815316	42.04287125	11.89753914
DALYs (Disability-Adjusted Life Years)	Global	Both	70-74 years	Prostate cancer	Smoking	Number	2020	50822.58342	82807.30346	23440.91768
DALYs (Disability-Adjusted Life Years)	Global	Both	70-74 years	Prostate cancer	Smoking	Rate	2020	25.92377581	42.23866293	11.95683206
DALYs (Disability-Adjusted Life Years)	Global	Both	70-74 years	Prostate cancer	Smoking	Number	2021	52952.64906	86948.30927	24525.89982
DALYs (Disability-Adjusted Life Years)	Global	Both	70-74 years	Prostate cancer	Smoking	Rate	2021	25.72524758	42.24088544	11.91507613
DALYs (Disability-Adjusted Life Years)	Global	Both	75-79 years	Prostate cancer	Smoking	Number	1990	36857.90496	60239.77789	17295.64184

DALYs (Disability-Adjusted Life Years)	Global	Both	75-79 years	Prostate cancer	Smoking	Rate	1990	59.87750301	97.86252056	28.09763191
DALYs (Disability-Adjusted Life Years)	Global	Both	75-79 years	Prostate cancer	Smoking	Number	1991	36656.58127	60453.83276	17296.05509
DALYs (Disability-Adjusted Life Years)	Global	Both	75-79 years	Prostate cancer	Smoking	Rate	1991	59.3423254	97.86703754	28.00010513
DALYs (Disability-Adjusted Life Years)	Global	Both	75-79 years	Prostate cancer	Smoking	Number	1992	36067.64489	59714.11985	17001.32454
DALYs (Disability-Adjusted Life Years)	Global	Both	75-79 years	Prostate cancer	Smoking	Rate	1992	58.40740157	96.70014741	27.53168922
DALYs (Disability-Adjusted Life Years)	Global	Both	75-79 years	Prostate cancer	Smoking	Number	1993	35173.01491	58205.17135	16604.85286
DALYs (Disability-Adjusted Life Years)	Global	Both	75-79 years	Prostate cancer	Smoking	Rate	1993	57.04155006	94.3937619	26.92878469
DALYs (Disability-Adjusted Life Years)	Global	Both	75-79 years	Prostate cancer	Smoking	Number	1994	34658.70234	57292.43177	16136.70172
DALYs (Disability-Adjusted Life Years)	Global	Both	75-79 years	Prostate cancer	Smoking	Rate	1994	55.71615653	92.1013737	25.94081538
DALYs (Disability-Adjusted Life Years)	Global	Both	75-79 years	Prostate cancer	Smoking	Number	1995	34869.5805	57644.16043	16266.02066
DALYs (Disability-Adjusted Life Years)	Global	Both	75-79 years	Prostate cancer	Smoking	Rate	1995	54.64919556	90.34255507	25.49284882

DALYs (Disability-Adjusted Life Years)	Global	Both	75-79 years	Prostate cancer	Smoking	Number	1996	35680.98481	59016.25448	16665.19138
DALYs (Disability-Adjusted Life Years)	Global	Both	75-79 years	Prostate cancer	Smoking	Rate	1996	53.79437707	88.97575734	25.12524791
DALYs (Disability-Adjusted Life Years)	Global	Both	75-79 years	Prostate cancer	Smoking	Number	1997	36559.12523	60314.3413	17084.50179
DALYs (Disability-Adjusted Life Years)	Global	Both	75-79 years	Prostate cancer	Smoking	Rate	1997	52.71501645	86.96793137	24.63433649
DALYs (Disability-Adjusted Life Years)	Global	Both	75-79 years	Prostate cancer	Smoking	Number	1998	37720.97045	62413.72506	17695.46415
DALYs (Disability-Adjusted Life Years)	Global	Both	75-79 years	Prostate cancer	Smoking	Rate	1998	51.92950777	85.92339968	24.36089879
DALYs (Disability-Adjusted Life Years)	Global	Both	75-79 years	Prostate cancer	Smoking	Number	1999	38702.18383	64129.43385	18160.71618
DALYs (Disability-Adjusted Life Years)	Global	Both	75-79 years	Prostate cancer	Smoking	Rate	1999	51.04119102	84.57514174	23.95070489
DALYs (Disability-Adjusted Life Years)	Global	Both	75-79 years	Prostate cancer	Smoking	Number	2000	39125.64275	64820.04135	18288.02974
DALYs (Disability-Adjusted Life Years)	Global	Both	75-79 years	Prostate cancer	Smoking	Rate	2000	49.66411632	82.27928916	23.21390199
DALYs (Disability-Adjusted Life Years)	Global	Both	75-79 years	Prostate cancer	Smoking	Number	2001	39477.5187	65062.05572	18179.40388

DALYs (Disability-Adjusted Life Years)	Global	Both	75-79 years	Prostate cancer	Smoking	Rate	2001	48.39620642	79.76075453	22.2864610 5
DALYs (Disability-Adjusted Life Years)	Global	Both	75-79 years	Prostate cancer	Smoking	Number	2002	39742.38755	65445.21567	18370.24014
DALYs (Disability-Adjusted Life Years)	Global	Both	75-79 years	Prostate cancer	Smoking	Rate	2002	47.06923378	77.51059627	21.75694973
DALYs (Disability-Adjusted Life Years)	Global	Both	75-79 years	Prostate cancer	Smoking	Number	2003	40153.21441	66608.30667	18565.16604
DALYs (Disability-Adjusted Life Years)	Global	Both	75-79 years	Prostate cancer	Smoking	Rate	2003	45.96864562	76.25525602	21.25397807
DALYs (Disability-Adjusted Life Years)	Global	Both	75-79 years	Prostate cancer	Smoking	Number	2004	40274.90856	66913.12467	18625.59558
DALYs (Disability-Adjusted Life Years)	Global	Both	75-79 years	Prostate cancer	Smoking	Rate	2004	44.63365465	74.1547878	20.64134794
DALYs (Disability-Adjusted Life Years)	Global	Both	75-79 years	Prostate cancer	Smoking	Number	2005	40128.09412	66446.57182	18578.92313
DALYs (Disability-Adjusted Life Years)	Global	Both	75-79 years	Prostate cancer	Smoking	Rate	2005	43.22134353	71.56856488	20.01106797
DALYs (Disability-Adjusted Life Years)	Global	Both	75-79 years	Prostate cancer	Smoking	Number	2006	39944.70936	65764.0773	18363.19133
DALYs (Disability-Adjusted Life Years)	Global	Both	75-79 years	Prostate cancer	Smoking	Rate	2006	41.87856646	68.94793645	19.2522149

DALYs (Disability-Adjusted Life Years)	Global	Both	75-79 years	Prostate cancer	Smoking	Number	2007	39547.42573	64908.46987	18074.36105
DALYs (Disability-Adjusted Life Years)	Global	Both	75-79 years	Prostate cancer	Smoking	Rate	2007	40.54159374	66.54017972	18.52872568
DALYs (Disability-Adjusted Life Years)	Global	Both	75-79 years	Prostate cancer	Smoking	Number	2008	39103.96394	64332.23452	17993.7141
DALYs (Disability-Adjusted Life Years)	Global	Both	75-79 years	Prostate cancer	Smoking	Rate	2008	39.26458009	64.59647361	18.06762172
DALYs (Disability-Adjusted Life Years)	Global	Both	75-79 years	Prostate cancer	Smoking	Number	2009	38584.46428	63497.44632	17777.04032
DALYs (Disability-Adjusted Life Years)	Global	Both	75-79 years	Prostate cancer	Smoking	Rate	2009	37.95399536	62.45990007	17.48656405
DALYs (Disability-Adjusted Life Years)	Global	Both	75-79 years	Prostate cancer	Smoking	Number	2010	38256.59526	62796.60327	17565.10406
DALYs (Disability-Adjusted Life Years)	Global	Both	75-79 years	Prostate cancer	Smoking	Rate	2010	36.77753325	60.36878475	16.88600865
DALYs (Disability-Adjusted Life Years)	Global	Both	75-79 years	Prostate cancer	Smoking	Number	2011	37998.75793	62112.95554	17364.15801
DALYs (Disability-Adjusted Life Years)	Global	Both	75-79 years	Prostate cancer	Smoking	Rate	2011	35.58547879	58.16819764	16.26137037
DALYs (Disability-Adjusted Life Years)	Global	Both	75-79 years	Prostate cancer	Smoking	Number	2012	37911.54507	61764.27954	17260.58472

DALYs (Disability-Adjusted Life Years)	Global	Both	75-79 years	Prostate cancer	Smoking	Rate	2012	34.48313402	56.17882166	15.69967816
DALYs (Disability-Adjusted Life Years)	Global	Both	75-79 years	Prostate cancer	Smoking	Number	2013	38111.06482	62205.14615	17438.74852
DALYs (Disability-Adjusted Life Years)	Global	Both	75-79 years	Prostate cancer	Smoking	Rate	2013	33.70723872	55.01719044	15.42365877
DALYs (Disability-Adjusted Life Years)	Global	Both	75-79 years	Prostate cancer	Smoking	Number	2014	38757.95794	62650.38066	17646.2544
DALYs (Disability-Adjusted Life Years)	Global	Both	75-79 years	Prostate cancer	Smoking	Rate	2014	33.42539252	54.03054435	15.21837091
DALYs (Disability-Adjusted Life Years)	Global	Both	75-79 years	Prostate cancer	Smoking	Number	2015	39310.04275	64319.37504	17905.02331
DALYs (Disability-Adjusted Life Years)	Global	Both	75-79 years	Prostate cancer	Smoking	Rate	2015	33.09481732	54.14997843	15.07409899
DALYs (Disability-Adjusted Life Years)	Global	Both	75-79 years	Prostate cancer	Smoking	Number	2016	39943.48069	65896.9455	18406.13284
DALYs (Disability-Adjusted Life Years)	Global	Both	75-79 years	Prostate cancer	Smoking	Rate	2016	32.92392111	54.31639401	15.1714887
DALYs (Disability-Adjusted Life Years)	Global	Both	75-79 years	Prostate cancer	Smoking	Number	2017	40101.42061	66974.34558	18079.63462
DALYs (Disability-Adjusted Life Years)	Global	Both	75-79 years	Prostate cancer	Smoking	Rate	2017	32.49278949	54.26698804	14.64930052

DALYs (Disability-Adjusted Life Years)	Global	Both	75-79 years	Prostate cancer	Smoking	Number	2018	40255.61473	67170.15681	18333.99137
DALYs (Disability-Adjusted Life Years)	Global	Both	75-79 years	Prostate cancer	Smoking	Rate	2018	32.08311274	53.53359346	14.6119123
DALYs (Disability-Adjusted Life Years)	Global	Both	75-79 years	Prostate cancer	Smoking	Number	2019	40408.91563	66671.77333	18364.49829
DALYs (Disability-Adjusted Life Years)	Global	Both	75-79 years	Prostate cancer	Smoking	Rate	2019	31.65112134	52.22204938	14.38437421
DALYs (Disability-Adjusted Life Years)	Global	Both	75-79 years	Prostate cancer	Smoking	Number	2020	40337.30267	66493.21353	18507.51206
DALYs (Disability-Adjusted Life Years)	Global	Both	75-79 years	Prostate cancer	Smoking	Rate	2020	31.18211007	51.40152082	14.30693774
DALYs (Disability-Adjusted Life Years)	Global	Both	75-79 years	Prostate cancer	Smoking	Number	2021	40615.69328	68076.75569	18445.49063
DALYs (Disability-Adjusted Life Years)	Global	Both	75-79 years	Prostate cancer	Smoking	Rate	2021	30.79643374	51.61850327	13.98610449
DALYs (Disability-Adjusted Life Years)	Global	Both	80-84 years	Prostate cancer	Smoking	Number	1990	19338.23047	32523.97783	8770.327025
DALYs (Disability-Adjusted Life Years)	Global	Both	80-84 years	Prostate cancer	Smoking	Rate	1990	54.6649431	91.93816366	24.79179409
DALYs (Disability-Adjusted Life Years)	Global	Both	80-84 years	Prostate cancer	Smoking	Number	1991	19997.77301	33695.35999	9099.124961

DALYs (Disability-Adjusted Life Years)	Global	Both	80-84 years	Prostate cancer	Smoking	Rate	1991	54.80126061	92.33769197	24.93495241
DALYs (Disability-Adjusted Life Years)	Global	Both	80-84 years	Prostate cancer	Smoking	Number	1992	20633.72636	34803.77168	9386.726409
DALYs (Disability-Adjusted Life Years)	Global	Both	80-84 years	Prostate cancer	Smoking	Rate	1992	55.01298821	92.79271457	25.02659288
DALYs (Disability-Adjusted Life Years)	Global	Both	80-84 years	Prostate cancer	Smoking	Number	1993	21219.3014	36062.74302	9605.373704
DALYs (Disability-Adjusted Life Years)	Global	Both	80-84 years	Prostate cancer	Smoking	Rate	1993	55.0140049	93.49770213	24.90327396
DALYs (Disability-Adjusted Life Years)	Global	Both	80-84 years	Prostate cancer	Smoking	Number	1994	21742.34689	37057.52033	9820.701991
DALYs (Disability-Adjusted Life Years)	Global	Both	80-84 years	Prostate cancer	Smoking	Rate	1994	55.11982567	93.94588682	24.8968239
DALYs (Disability-Adjusted Life Years)	Global	Both	80-84 years	Prostate cancer	Smoking	Number	1995	21818.71096	37161.14487	9873.684233
DALYs (Disability-Adjusted Life Years)	Global	Both	80-84 years	Prostate cancer	Smoking	Rate	1995	54.43750887	92.71675843	24.63476298
DALYs (Disability-Adjusted Life Years)	Global	Both	80-84 years	Prostate cancer	Smoking	Number	1996	21485.51031	36616.81252	9755.803786
DALYs (Disability-Adjusted Life Years)	Global	Both	80-84 years	Prostate cancer	Smoking	Rate	1996	53.35827853	90.93617293	24.22809084

DALYs (Disability-Adjusted Life Years)	Global	Both	80-84 years	Prostate cancer	Smoking	Number	1997	20794.98226	35295.36887	9329.276192
DALYs (Disability-Adjusted Life Years)	Global	Both	80-84 years	Prostate cancer	Smoking	Rate	1997	51.5412857	87.48113696	23.12302476
DALYs (Disability-Adjusted Life Years)	Global	Both	80-84 years	Prostate cancer	Smoking	Number	1998	20214.31362	34432.52081	9042.367515
DALYs (Disability-Adjusted Life Years)	Global	Both	80-84 years	Prostate cancer	Smoking	Rate	1998	49.96982163	85.1172568	22.35274966
DALYs (Disability-Adjusted Life Years)	Global	Both	80-84 years	Prostate cancer	Smoking	Number	1999	19734.98219	33598.28883	8823.691101
DALYs (Disability-Adjusted Life Years)	Global	Both	80-84 years	Prostate cancer	Smoking	Rate	1999	48.08557072	81.86442117	21.49949862
DALYs (Disability-Adjusted Life Years)	Global	Both	80-84 years	Prostate cancer	Smoking	Number	2000	19881.45458	33992.01694	8907.180283
DALYs (Disability-Adjusted Life Years)	Global	Both	80-84 years	Prostate cancer	Smoking	Rate	2000	46.84466408	80.09195748	20.98708958
DALYs (Disability-Adjusted Life Years)	Global	Both	80-84 years	Prostate cancer	Smoking	Number	2001	20474.91057	34872.07583	9151.385825
DALYs (Disability-Adjusted Life Years)	Global	Both	80-84 years	Prostate cancer	Smoking	Rate	2001	46.04130284	78.41576638	20.57844037
DALYs (Disability-Adjusted Life Years)	Global	Both	80-84 years	Prostate cancer	Smoking	Number	2002	21139.84479	36202.72511	9422.438241

DALYs (Disability-Adjusted Life Years)	Global	Both	80-84 years	Prostate cancer	Smoking	Rate	2002	45.19522961	77.39841471	20.14438914
DALYs (Disability-Adjusted Life Years)	Global	Both	80-84 years	Prostate cancer	Smoking	Number	2003	21822.28134	37217.00473	9627.61825
DALYs (Disability-Adjusted Life Years)	Global	Both	80-84 years	Prostate cancer	Smoking	Rate	2003	44.36631072	75.6649211	19.57365942
DALYs (Disability-Adjusted Life Years)	Global	Both	80-84 years	Prostate cancer	Smoking	Number	2004	22409.32354	38507.46365	9853.676672
DALYs (Disability-Adjusted Life Years)	Global	Both	80-84 years	Prostate cancer	Smoking	Rate	2004	43.49448257	74.73952544	19.12510066
DALYs (Disability-Adjusted Life Years)	Global	Both	80-84 years	Prostate cancer	Smoking	Number	2005	22673.16406	38828.81648	9960.487658
DALYs (Disability-Adjusted Life Years)	Global	Both	80-84 years	Prostate cancer	Smoking	Rate	2005	42.19306683	72.25753074	18.53572445
DALYs (Disability-Adjusted Life Years)	Global	Both	80-84 years	Prostate cancer	Smoking	Number	2006	22855.992	38978.52152	10056.09742
DALYs (Disability-Adjusted Life Years)	Global	Both	80-84 years	Prostate cancer	Smoking	Rate	2006	40.89704712	69.74566808	17.99373618
DALYs (Disability-Adjusted Life Years)	Global	Both	80-84 years	Prostate cancer	Smoking	Number	2007	23130.26029	39516.10855	10190.05024
DALYs (Disability-Adjusted Life Years)	Global	Both	80-84 years	Prostate cancer	Smoking	Rate	2007	39.77462899	67.95161562	17.52273698

DALYs (Disability-Adjusted Life Years)	Global	Both	80-84 years	Prostate cancer	Smoking	Number	2008	23397.97283	39826.76716	10318.19863
DALYs (Disability-Adjusted Life Years)	Global	Both	80-84 years	Prostate cancer	Smoking	Rate	2008	38.6973825	65.8685969 9	17.0650373
DALYs (Disability-Adjusted Life Years)	Global	Both	80-84 years	Prostate cancer	Smoking	Number	2009	23473.52193	40260.2623	10342.36905
DALYs (Disability-Adjusted Life Years)	Global	Both	80-84 years	Prostate cancer	Smoking	Rate	2009	37.41356657	64.16932271	16.48431429
DALYs (Disability-Adjusted Life Years)	Global	Both	80-84 years	Prostate cancer	Smoking	Number	2010	23610.42997	40624.26636	10401.15218
DALYs (Disability-Adjusted Life Years)	Global	Both	80-84 years	Prostate cancer	Smoking	Rate	2010	36.41894632	62.6626867	16.04371473
DALYs (Disability-Adjusted Life Years)	Global	Both	80-84 years	Prostate cancer	Smoking	Number	2011	23821.2554	40995.41005	10461.80499
DALYs (Disability-Adjusted Life Years)	Global	Both	80-84 years	Prostate cancer	Smoking	Rate	2011	35.61252441	61.28770362	15.64028761
DALYs (Disability-Adjusted Life Years)	Global	Both	80-84 years	Prostate cancer	Smoking	Number	2012	23638.32193	40589.60019	10344.41275
DALYs (Disability-Adjusted Life Years)	Global	Both	80-84 years	Prostate cancer	Smoking	Rate	2012	34.41321568	59.09127855	15.05963528
DALYs (Disability-Adjusted Life Years)	Global	Both	80-84 years	Prostate cancer	Smoking	Number	2013	23207.86631	40230.47276	10105.79779

DALYs (Disability-Adjusted Life Years)	Global	Both	80-84 years	Prostate cancer	Smoking	Rate	2013	32.94473676	57.1091852	14.34568968
DALYs (Disability-Adjusted Life Years)	Global	Both	80-84 years	Prostate cancer	Smoking	Number	2014	23317.5999	40313.72858	10138.79514
DALYs (Disability-Adjusted Life Years)	Global	Both	80-84 years	Prostate cancer	Smoking	Rate	2014	32.28868444	55.82380974	14.03953915
DALYs (Disability-Adjusted Life Years)	Global	Both	80-84 years	Prostate cancer	Smoking	Number	2015	23846.2749	41268.74131	10339.87668
DALYs (Disability-Adjusted Life Years)	Global	Both	80-84 years	Prostate cancer	Smoking	Rate	2015	32.12418753	55.59462812	13.92922538
DALYs (Disability-Adjusted Life Years)	Global	Both	80-84 years	Prostate cancer	Smoking	Number	2016	24454.24128	42250.98692	10609.8442
DALYs (Disability-Adjusted Life Years)	Global	Both	80-84 years	Prostate cancer	Smoking	Rate	2016	31.93418128	55.17450572	13.85512984
DALYs (Disability-Adjusted Life Years)	Global	Both	80-84 years	Prostate cancer	Smoking	Number	2017	24625.83928	42470.61171	10675.46919
DALYs (Disability-Adjusted Life Years)	Global	Both	80-84 years	Prostate cancer	Smoking	Rate	2017	31.09095611	53.62058565	13.4781414
DALYs (Disability-Adjusted Life Years)	Global	Both	80-84 years	Prostate cancer	Smoking	Number	2018	25217.89166	43912.29856	10908.73228
DALYs (Disability-Adjusted Life Years)	Global	Both	80-84 years	Prostate cancer	Smoking	Rate	2018	30.8306114	53.68581289	13.33667741

DALYs (Disability-Adjusted Life Years)	Global	Both	80-84 years	Prostate cancer	Smoking	Number	2019	25915.46408	44910.31807	11323.35071
DALYs (Disability-Adjusted Life Years)	Global	Both	80-84 years	Prostate cancer	Smoking	Rate	2019	30.77952737	53.33951805	13.44862596
DALYs (Disability-Adjusted Life Years)	Global	Both	80-84 years	Prostate cancer	Smoking	Number	2020	26112.15249	45589.89537	11616.24171
DALYs (Disability-Adjusted Life Years)	Global	Both	80-84 years	Prostate cancer	Smoking	Rate	2020	30.277401	52.86211253	13.46919249
DALYs (Disability-Adjusted Life Years)	Global	Both	80-84 years	Prostate cancer	Smoking	Number	2021	26112.60266	46682.97429	11410.91934
DALYs (Disability-Adjusted Life Years)	Global	Both	80-84 years	Prostate cancer	Smoking	Rate	2021	29.81467373	53.30137579	13.02868356
DALYs (Disability-Adjusted Life Years)	Global	Both	85-89 years	Prostate cancer	Smoking	Number	1990	8234.183288	14106.5879	3709.547252
DALYs (Disability-Adjusted Life Years)	Global	Both	85-89 years	Prostate cancer	Smoking	Rate	1990	54.49105803	93.35265843	24.54853718
DALYs (Disability-Adjusted Life Years)	Global	Both	85-89 years	Prostate cancer	Smoking	Number	1991	8680.46328	14901.84699	3897.18573
DALYs (Disability-Adjusted Life Years)	Global	Both	85-89 years	Prostate cancer	Smoking	Rate	1991	54.9190615	94.2801582	24.65649308
DALYs (Disability-Adjusted Life Years)	Global	Both	85-89 years	Prostate cancer	Smoking	Number	1992	9164.553922	15883.83332	4110.904561

DALYs (Disability-Adjusted Life Years)	Global	Both	85-89 years	Prostate cancer	Smoking	Rate	1992	55.32638982	95.89066328	24.81752088
DALYs (Disability-Adjusted Life Years)	Global	Both	85-89 years	Prostate cancer	Smoking	Number	1993	9609.726133	16688.76798	4316.93981
DALYs (Disability-Adjusted Life Years)	Global	Both	85-89 years	Prostate cancer	Smoking	Rate	1993	55.83918502	96.97333619	25.08441941
DALYs (Disability-Adjusted Life Years)	Global	Both	85-89 years	Prostate cancer	Smoking	Number	1994	10034.07833	17309.52879	4507.798629
DALYs (Disability-Adjusted Life Years)	Global	Both	85-89 years	Prostate cancer	Smoking	Rate	1994	56.24490399	97.02662791	25.26796111
DALYs (Disability-Adjusted Life Years)	Global	Both	85-89 years	Prostate cancer	Smoking	Number	1995	10342.68212	17809.9214	4622.57032
DALYs (Disability-Adjusted Life Years)	Global	Both	85-89 years	Prostate cancer	Smoking	Rate	1995	56.13976607	96.67171527	25.09117203
DALYs (Disability-Adjusted Life Years)	Global	Both	85-89 years	Prostate cancer	Smoking	Number	1996	10648.81482	18347.17363	4737.725086
DALYs (Disability-Adjusted Life Years)	Global	Both	85-89 years	Prostate cancer	Smoking	Rate	1996	55.91760231	96.34217288	24.87809505
DALYs (Disability-Adjusted Life Years)	Global	Both	85-89 years	Prostate cancer	Smoking	Number	1997	10820.35251	18585.79507	4787.540608
DALYs (Disability-Adjusted Life Years)	Global	Both	85-89 years	Prostate cancer	Smoking	Rate	1997	55.05825776	94.57191847	24.36091103

DALYs (Disability-Adjusted Life Years)	Global	Both	85-89 years	Prostate cancer	Smoking	Number	1998	11014.403	18894.50392	4847.730293
DALYs (Disability-Adjusted Life Years)	Global	Both	85-89 years	Prostate cancer	Smoking	Rate	1998	54.20531759	92.98575565	23.857195
DALYs (Disability-Adjusted Life Years)	Global	Both	85-89 years	Prostate cancer	Smoking	Number	1999	11132.07312	19133.83065	4878.555741
DALYs (Disability-Adjusted Life Years)	Global	Both	85-89 years	Prostate cancer	Smoking	Rate	1999	53.22624533	91.48538227	23.32604197
DALYs (Disability-Adjusted Life Years)	Global	Both	85-89 years	Prostate cancer	Smoking	Number	2000	11191.47548	19366.16303	4906.777902
DALYs (Disability-Adjusted Life Years)	Global	Both	85-89 years	Prostate cancer	Smoking	Rate	2000	52.31218211	90.5230279	22.9356942
DALYs (Disability-Adjusted Life Years)	Global	Both	85-89 years	Prostate cancer	Smoking	Number	2001	11154.66388	19309.68845	4857.904822
DALYs (Disability-Adjusted Life Years)	Global	Both	85-89 years	Prostate cancer	Smoking	Rate	2001	51.54063862	89.22130551	22.44617315
DALYs (Disability-Adjusted Life Years)	Global	Both	85-89 years	Prostate cancer	Smoking	Number	2002	10864.98365	18839.23602	4715.136399
DALYs (Disability-Adjusted Life Years)	Global	Both	85-89 years	Prostate cancer	Smoking	Rate	2002	49.81321272	86.3731508	21.61771246
DALYs (Disability-Adjusted Life Years)	Global	Both	85-89 years	Prostate cancer	Smoking	Number	2003	10534.50871	18224.70813	4551.447617

DALYs (Disability-Adjusted Life Years)	Global	Both	85-89 years	Prostate cancer	Smoking	Rate	2003	47.92048287	82.90247204	20.7041043 5
DALYs (Disability-Adjusted Life Years)	Global	Both	85-89 years	Prostate cancer	Smoking	Number	2004	10309.46473	17872.441	4437.684698
DALYs (Disability-Adjusted Life Years)	Global	Both	85-89 years	Prostate cancer	Smoking	Rate	2004	45.95548246	79.66821464	19.78142869
DALYs (Disability-Adjusted Life Years)	Global	Both	85-89 years	Prostate cancer	Smoking	Number	2005	10428.87594	18057.75835	4512.686196
DALYs (Disability-Adjusted Life Years)	Global	Both	85-89 years	Prostate cancer	Smoking	Rate	2005	44.57203571	77.17716221	19.28679672
DALYs (Disability-Adjusted Life Years)	Global	Both	85-89 years	Prostate cancer	Smoking	Number	2006	10741.28516	18613.1075	4624.492368
DALYs (Disability-Adjusted Life Years)	Global	Both	85-89 years	Prostate cancer	Smoking	Rate	2006	43.3964191	75.19977373	18.68364966
DALYs (Disability-Adjusted Life Years)	Global	Both	85-89 years	Prostate cancer	Smoking	Number	2007	11215.62565	19476.07126	4807.858937
DALYs (Disability-Adjusted Life Years)	Global	Both	85-89 years	Prostate cancer	Smoking	Rate	2007	42.65787536	74.07592286	18.28636705
DALYs (Disability-Adjusted Life Years)	Global	Both	85-89 years	Prostate cancer	Smoking	Number	2008	11619.35169	20227.90432	4994.666261
DALYs (Disability-Adjusted Life Years)	Global	Both	85-89 years	Prostate cancer	Smoking	Rate	2008	41.67140266	72.5449378	17.91276781

DALYs (Disability-Adjusted Life Years)	Global	Both	85-89 years	Prostate cancer	Smoking	Number	2009	11894.96778	20780.73968	5150.169594
DALYs (Disability-Adjusted Life Years)	Global	Both	85-89 years	Prostate cancer	Smoking	Rate	2009	40.39863989	70.57720833	17.49141743
DALYs (Disability-Adjusted Life Years)	Global	Both	85-89 years	Prostate cancer	Smoking	Number	2010	12143.14827	21393.33252	5233.913074
DALYs (Disability-Adjusted Life Years)	Global	Both	85-89 years	Prostate cancer	Smoking	Rate	2010	39.2545893	69.1572287	16.91942679
DALYs (Disability-Adjusted Life Years)	Global	Both	85-89 years	Prostate cancer	Smoking	Number	2011	12373.95584	21619.96953	5321.931451
DALYs (Disability-Adjusted Life Years)	Global	Both	85-89 years	Prostate cancer	Smoking	Rate	2011	38.28964363	66.90026531	16.46804477
DALYs (Disability-Adjusted Life Years)	Global	Both	85-89 years	Prostate cancer	Smoking	Number	2012	12583.36469	21869.14079	5386.128949
DALYs (Disability-Adjusted Life Years)	Global	Both	85-89 years	Prostate cancer	Smoking	Rate	2012	37.23731883	64.71624942	15.93890075
DALYs (Disability-Adjusted Life Years)	Global	Both	85-89 years	Prostate cancer	Smoking	Number	2013	12766.2816	22340.36668	5426.56832
DALYs (Disability-Adjusted Life Years)	Global	Both	85-89 years	Prostate cancer	Smoking	Rate	2013	36.16900803	63.29399016	15.37437441
DALYs (Disability-Adjusted Life Years)	Global	Both	85-89 years	Prostate cancer	Smoking	Number	2014	13207.51624	23151.44284	5587.938526

DALYs (Disability-Adjusted Life Years)	Global	Both	85-89 years	Prostate cancer	Smoking	Rate	2014	35.94984734	63.01645373	15.2099405 6
DALYs (Disability-Adjusted Life Years)	Global	Both	85-89 years	Prostate cancer	Smoking	Number	2015	13921.92705	24097.35571	5924.554519
DALYs (Disability-Adjusted Life Years)	Global	Both	85-89 years	Prostate cancer	Smoking	Rate	2015	36.56618733	63.29213044	15.5609471
DALYs (Disability-Adjusted Life Years)	Global	Both	85-89 years	Prostate cancer	Smoking	Number	2016	14632.18828	25302.47835	6276.03603
DALYs (Disability-Adjusted Life Years)	Global	Both	85-89 years	Prostate cancer	Smoking	Rate	2016	37.09155996	64.13998883	15.9093064
DALYs (Disability-Adjusted Life Years)	Global	Both	85-89 years	Prostate cancer	Smoking	Number	2017	14609.60396	25800.15856	6248.297797
DALYs (Disability-Adjusted Life Years)	Global	Both	85-89 years	Prostate cancer	Smoking	Rate	2017	35.91964418	63.43310314	15.36226678
DALYs (Disability-Adjusted Life Years)	Global	Both	85-89 years	Prostate cancer	Smoking	Number	2018	14819.22664	26065.14026	6341.710445
DALYs (Disability-Adjusted Life Years)	Global	Both	85-89 years	Prostate cancer	Smoking	Rate	2018	35.34880883	62.17407172	15.12709911
DALYs (Disability-Adjusted Life Years)	Global	Both	85-89 years	Prostate cancer	Smoking	Number	2019	15268.17778	26764.38457	6540.633405
DALYs (Disability-Adjusted Life Years)	Global	Both	85-89 years	Prostate cancer	Smoking	Rate	2019	35.27943639	61.84316273	15.11312375

DALYs (Disability-Adjusted Life Years)	Global	Both	85-89 years	Prostate cancer	Smoking	Number	2020	15412.7333	27304.49322	6590.858099
DALYs (Disability-Adjusted Life Years)	Global	Both	85-89 years	Prostate cancer	Smoking	Rate	2020	34.56314261	61.23048226	14.78003699
DALYs (Disability-Adjusted Life Years)	Global	Both	85-89 years	Prostate cancer	Smoking	Number	2021	15406.90147	28347.52896	6558.557364
DALYs (Disability-Adjusted Life Years)	Global	Both	85-89 years	Prostate cancer	Smoking	Rate	2021	33.6970646	62.00004047	14.34448917
DALYs (Disability-Adjusted Life Years)	Global	Both	90-94 years	Prostate cancer	Smoking	Number	1990	2283.775533	4029.969227	989.3361837
DALYs (Disability-Adjusted Life Years)	Global	Both	90-94 years	Prostate cancer	Smoking	Rate	1990	53.29464593	94.04417376	23.0873485
DALYs (Disability-Adjusted Life Years)	Global	Both	90-94 years	Prostate cancer	Smoking	Number	1991	2453.051821	4322.056244	1060.359262
DALYs (Disability-Adjusted Life Years)	Global	Both	90-94 years	Prostate cancer	Smoking	Rate	1991	53.59719194	94.43342213	23.16798953
DALYs (Disability-Adjusted Life Years)	Global	Both	90-94 years	Prostate cancer	Smoking	Number	1992	2642.413718	4661.374245	1137.76239
DALYs (Disability-Adjusted Life Years)	Global	Both	90-94 years	Prostate cancer	Smoking	Rate	1992	54.55289976	96.23454503	23.48922017
DALYs (Disability-Adjusted Life Years)	Global	Both	90-94 years	Prostate cancer	Smoking	Number	1993	2843.603113	5068.653616	1223.410025

DALYs (Disability-Adjusted Life Years)	Global	Both	90-94 years	Prostate cancer	Smoking	Rate	1993	55.16684429	98.33356264	23.73456058
DALYs (Disability-Adjusted Life Years)	Global	Both	90-94 years	Prostate cancer	Smoking	Number	1994	3044.180848	5412.086841	1293.05212
DALYs (Disability-Adjusted Life Years)	Global	Both	90-94 years	Prostate cancer	Smoking	Rate	1994	55.61392919	98.87303988	23.62267968
DALYs (Disability-Adjusted Life Years)	Global	Both	90-94 years	Prostate cancer	Smoking	Number	1995	3228.096153	5730.946328	1372.48755
DALYs (Disability-Adjusted Life Years)	Global	Both	90-94 years	Prostate cancer	Smoking	Rate	1995	55.77683281	99.02246401	23.71459987
DALYs (Disability-Adjusted Life Years)	Global	Both	90-94 years	Prostate cancer	Smoking	Number	1996	3396.902995	6020.872071	1436.848036
DALYs (Disability-Adjusted Life Years)	Global	Both	90-94 years	Prostate cancer	Smoking	Rate	1996	55.85683355	99.00396025	23.6267511
DALYs (Disability-Adjusted Life Years)	Global	Both	90-94 years	Prostate cancer	Smoking	Number	1997	3512.506139	6253.671484	1472.120152
DALYs (Disability-Adjusted Life Years)	Global	Both	90-94 years	Prostate cancer	Smoking	Rate	1997	54.81615313	97.5947657	22.97389968
DALYs (Disability-Adjusted Life Years)	Global	Both	90-94 years	Prostate cancer	Smoking	Number	1998	3579.633179	6365.498336	1497.481368
DALYs (Disability-Adjusted Life Years)	Global	Both	90-94 years	Prostate cancer	Smoking	Rate	1998	53.38289619	94.92836833	22.33186709

DALYs (Disability-Adjusted Life Years)	Global	Both	90-94 years	Prostate cancer	Smoking	Number	1999	3669.206549	6530.367144	1538.915779
DALYs (Disability-Adjusted Life Years)	Global	Both	90-94 years	Prostate cancer	Smoking	Rate	1999	52.38553573	93.23453908	21.97121542
DALYs (Disability-Adjusted Life Years)	Global	Both	90-94 years	Prostate cancer	Smoking	Number	2000	3746.388881	6656.385064	1577.482924
DALYs (Disability-Adjusted Life Years)	Global	Both	90-94 years	Prostate cancer	Smoking	Rate	2000	51.39237482	91.31124586	21.63966322
DALYs (Disability-Adjusted Life Years)	Global	Both	90-94 years	Prostate cancer	Smoking	Number	2001	3848.528775	6862.390378	1627.703333
DALYs (Disability-Adjusted Life Years)	Global	Both	90-94 years	Prostate cancer	Smoking	Rate	2001	50.72065857	90.44104377	21.45188197
DALYs (Disability-Adjusted Life Years)	Global	Both	90-94 years	Prostate cancer	Smoking	Number	2002	3925.928969	7016.666757	1663.458177
DALYs (Disability-Adjusted Life Years)	Global	Both	90-94 years	Prostate cancer	Smoking	Rate	2002	49.88601505	89.15941838	21.13723918
DALYs (Disability-Adjusted Life Years)	Global	Both	90-94 years	Prostate cancer	Smoking	Number	2003	3981.711757	7118.17736	1682.34363
DALYs (Disability-Adjusted Life Years)	Global	Both	90-94 years	Prostate cancer	Smoking	Rate	2003	48.74682098	87.1455642	20.5963939
DALYs (Disability-Adjusted Life Years)	Global	Both	90-94 years	Prostate cancer	Smoking	Number	2004	3973.277441	7115.790932	1669.173235

DALYs (Disability-Adjusted Life Years)	Global	Both	90-94 years	Prostate cancer	Smoking	Rate	2004	47.00035158	84.17350175	19.74484039
DALYs (Disability-Adjusted Life Years)	Global	Both	90-94 years	Prostate cancer	Smoking	Number	2005	3948.606978	7132.575565	1656.477049
DALYs (Disability-Adjusted Life Years)	Global	Both	90-94 years	Prostate cancer	Smoking	Rate	2005	45.33768182	81.89582891	19.01957572
DALYs (Disability-Adjusted Life Years)	Global	Both	90-94 years	Prostate cancer	Smoking	Number	2006	3925.800069	7117.173861	1646.147885
DALYs (Disability-Adjusted Life Years)	Global	Both	90-94 years	Prostate cancer	Smoking	Rate	2006	44.12175264	79.98934715	18.50092428
DALYs (Disability-Adjusted Life Years)	Global	Both	90-94 years	Prostate cancer	Smoking	Number	2007	3903.36313	7070.021068	1632.84803
DALYs (Disability-Adjusted Life Years)	Global	Both	90-94 years	Prostate cancer	Smoking	Rate	2007	43.02865936	77.93626112	17.99967344
DALYs (Disability-Adjusted Life Years)	Global	Both	90-94 years	Prostate cancer	Smoking	Number	2008	3850.405419	6973.129281	1609.116923
DALYs (Disability-Adjusted Life Years)	Global	Both	90-94 years	Prostate cancer	Smoking	Rate	2008	41.55474239	75.25612485	17.36607757
DALYs (Disability-Adjusted Life Years)	Global	Both	90-94 years	Prostate cancer	Smoking	Number	2009	3812.623855	6963.538167	1597.984363
DALYs (Disability-Adjusted Life Years)	Global	Both	90-94 years	Prostate cancer	Smoking	Rate	2009	39.69662276	72.5035981	16.63803849

DALYs (Disability-Adjusted Life Years)	Global	Both	90-94 years	Prostate cancer	Smoking	Number	2010	3936.5604	7211.892935	1626.997603
DALYs (Disability-Adjusted Life Years)	Global	Both	90-94 years	Prostate cancer	Smoking	Rate	2010	38.57676316	70.67375004	15.94394467
DALYs (Disability-Adjusted Life Years)	Global	Both	90-94 years	Prostate cancer	Smoking	Number	2011	4160.967092	7626.016201	1732.267754
DALYs (Disability-Adjusted Life Years)	Global	Both	90-94 years	Prostate cancer	Smoking	Rate	2011	38.04546009	69.72785138	15.83884762
DALYs (Disability-Adjusted Life Years)	Global	Both	90-94 years	Prostate cancer	Smoking	Number	2012	4355.679821	8027.193456	1822.516515
DALYs (Disability-Adjusted Life Years)	Global	Both	90-94 years	Prostate cancer	Smoking	Rate	2012	37.16111377	68.48516454	15.54906383
DALYs (Disability-Adjusted Life Years)	Global	Both	90-94 years	Prostate cancer	Smoking	Number	2013	4562.574683	8463.304568	1895.611288
DALYs (Disability-Adjusted Life Years)	Global	Both	90-94 years	Prostate cancer	Smoking	Rate	2013	36.52304731	67.74807968	15.17421753
DALYs (Disability-Adjusted Life Years)	Global	Both	90-94 years	Prostate cancer	Smoking	Number	2014	4839.69763	9033.894013	2022.56579
DALYs (Disability-Adjusted Life Years)	Global	Both	90-94 years	Prostate cancer	Smoking	Rate	2014	36.64312997	68.3989327	15.31358915
DALYs (Disability-Adjusted Life Years)	Global	Both	90-94 years	Prostate cancer	Smoking	Number	2015	5130.748948	9506.952442	2133.258548

DALYs (Disability-Adjusted Life Years)	Global	Both	90-94 years	Prostate cancer	Smoking	Rate	2015	36.93508164	68.4383641	15.35683765
DALYs (Disability-Adjusted Life Years)	Global	Both	90-94 years	Prostate cancer	Smoking	Number	2016	5389.242356	10048.66892	2236.796284
DALYs (Disability-Adjusted Life Years)	Global	Both	90-94 years	Prostate cancer	Smoking	Rate	2016	36.99299046	68.97635862	15.35388059
DALYs (Disability-Adjusted Life Years)	Global	Both	90-94 years	Prostate cancer	Smoking	Number	2017	5552.133103	10453.90705	2280.700597
DALYs (Disability-Adjusted Life Years)	Global	Both	90-94 years	Prostate cancer	Smoking	Rate	2017	36.32359204	68.39235441	14.92097479
DALYs (Disability-Adjusted Life Years)	Global	Both	90-94 years	Prostate cancer	Smoking	Number	2018	5795.621785	11004.78764	2339.300995
DALYs (Disability-Adjusted Life Years)	Global	Both	90-94 years	Prostate cancer	Smoking	Rate	2018	36.12372138	68.59210231	14.580706
DALYs (Disability-Adjusted Life Years)	Global	Both	90-94 years	Prostate cancer	Smoking	Number	2019	6082.392236	11483.41897	2478.123934
DALYs (Disability-Adjusted Life Years)	Global	Both	90-94 years	Prostate cancer	Smoking	Rate	2019	36.16115444	68.27144172	14.73298973
DALYs (Disability-Adjusted Life Years)	Global	Both	90-94 years	Prostate cancer	Smoking	Number	2020	6243.133275	11745.53864	2556.64522
DALYs (Disability-Adjusted Life Years)	Global	Both	90-94 years	Prostate cancer	Smoking	Rate	2020	35.79509968	67.34322463	14.65856429

DALYs (Disability-Adjusted Life Years)	Global	Both	90-94 years	Prostate cancer	Smoking	Number	2021	6266.174542	11982.76777	2564.044085
DALYs (Disability-Adjusted Life Years)	Global	Both	90-94 years	Prostate cancer	Smoking	Rate	2021	35.02735582	66.98260118	14.33277735
DALYs (Disability-Adjusted Life Years)	Global	Both	95+ years	Prostate cancer	Smoking	Number	1990	364.8818777	687.8004029	143.472464 7
DALYs (Disability-Adjusted Life Years)	Global	Both	95+ years	Prostate cancer	Smoking	Rate	1990	35.83996796	67.55814938	14.09236482
DALYs (Disability-Adjusted Life Years)	Global	Both	95+ years	Prostate cancer	Smoking	Number	1991	392.9050246	742.2794038	153.5991403
DALYs (Disability-Adjusted Life Years)	Global	Both	95+ years	Prostate cancer	Smoking	Rate	1991	37.07533207	70.04302228	14.49393308
DALYs (Disability-Adjusted Life Years)	Global	Both	95+ years	Prostate cancer	Smoking	Number	1992	420.751431	799.6476661	164.8661237
DALYs (Disability-Adjusted Life Years)	Global	Both	95+ years	Prostate cancer	Smoking	Rate	1992	37.96817598	72.15938217	14.87734928
DALYs (Disability-Adjusted Life Years)	Global	Both	95+ years	Prostate cancer	Smoking	Number	1993	444.6652909	854.1169968	173.5748327
DALYs (Disability-Adjusted Life Years)	Global	Both	95+ years	Prostate cancer	Smoking	Rate	1993	38.43749534	73.8310787	15.00405352
DALYs (Disability-Adjusted Life Years)	Global	Both	95+ years	Prostate cancer	Smoking	Number	1994	460.9512187	888.6957976	179.5410245

DALYs (Disability-Adjusted Life Years)	Global	Both	95+ years	Prostate cancer	Smoking	Rate	1994	38.06291293	73.38379722	14.82554792
DALYs (Disability-Adjusted Life Years)	Global	Both	95+ years	Prostate cancer	Smoking	Number	1995	487.3900323	948.6882205	190.09577 31
DALYs (Disability-Adjusted Life Years)	Global	Both	95+ years	Prostate cancer	Smoking	Rate	1995	38.06959142	74.10117266	14.84820766
DALYs (Disability-Adjusted Life Years)	Global	Both	95+ years	Prostate cancer	Smoking	Number	1996	520.0307939	1012.408513	202.997378
DALYs (Disability-Adjusted Life Years)	Global	Both	95+ years	Prostate cancer	Smoking	Rate	1996	37.98211703	73.94450304	14.82656461
DALYs (Disability-Adjusted Life Years)	Global	Both	95+ years	Prostate cancer	Smoking	Number	1997	542.0191329	1064.600552	210.804907
DALYs (Disability-Adjusted Life Years)	Global	Both	95+ years	Prostate cancer	Smoking	Rate	1997	37.30465972	73.27151185	14.50872275
DALYs (Disability-Adjusted Life Years)	Global	Both	95+ years	Prostate cancer	Smoking	Number	1998	559.5772534	1103.946653	217.2347173
DALYs (Disability-Adjusted Life Years)	Global	Both	95+ years	Prostate cancer	Smoking	Rate	1998	36.01788853	71.05690454	13.98258379
DALYs (Disability-Adjusted Life Years)	Global	Both	95+ years	Prostate cancer	Smoking	Number	1999	580.9575848	1152.095603	224.7311922
DALYs (Disability-Adjusted Life Years)	Global	Both	95+ years	Prostate cancer	Smoking	Rate	1999	34.94408378	69.29752932	13.51738201

DALYs (Disability-Adjusted Life Years)	Global	Both	95+ years	Prostate cancer	Smoking	Number	2000	599.5747685	1187.162737	231.574179 9
DALYs (Disability-Adjusted Life Years)	Global	Both	95+ years	Prostate cancer	Smoking	Rate	2000	33.62633404	66.58040476	12.98752239
DALYs (Disability-Adjusted Life Years)	Global	Both	95+ years	Prostate cancer	Smoking	Number	2001	630.6198236	1249.19394	242.7380755
DALYs (Disability-Adjusted Life Years)	Global	Both	95+ years	Prostate cancer	Smoking	Rate	2001	33.0379277	65.4447855	12.71695353
DALYs (Disability-Adjusted Life Years)	Global	Both	95+ years	Prostate cancer	Smoking	Number	2002	654.5635652	1308.513593	251.1913143
DALYs (Disability-Adjusted Life Years)	Global	Both	95+ years	Prostate cancer	Smoking	Rate	2002	32.08205275	64.13403428	12.31161253
DALYs (Disability-Adjusted Life Years)	Global	Both	95+ years	Prostate cancer	Smoking	Number	2003	681.4760334	1375.09381	261.0145037
DALYs (Disability-Adjusted Life Years)	Global	Both	95+ years	Prostate cancer	Smoking	Rate	2003	31.52481993	63.61131225	12.07443083
DALYs (Disability-Adjusted Life Years)	Global	Both	95+ years	Prostate cancer	Smoking	Number	2004	701.359522	1418.609348	266.5328575
DALYs (Disability-Adjusted Life Years)	Global	Both	95+ years	Prostate cancer	Smoking	Rate	2004	30.68720565	62.06967386	11.66184867
DALYs (Disability-Adjusted Life Years)	Global	Both	95+ years	Prostate cancer	Smoking	Number	2005	711.5645578	1447.167709	270.3965347

DALYs (Disability-Adjusted Life Years)	Global	Both	95+ years	Prostate cancer	Smoking	Rate	2005	29.50373816	60.00419313	11.21150354
DALYs (Disability-Adjusted Life Years)	Global	Both	95+ years	Prostate cancer	Smoking	Number	2006	729.7368886	1488.136893	274.4183388
DALYs (Disability-Adjusted Life Years)	Global	Both	95+ years	Prostate cancer	Smoking	Rate	2006	28.67766807	58.48175765	10.7842678
DALYs (Disability-Adjusted Life Years)	Global	Both	95+ years	Prostate cancer	Smoking	Number	2007	749.7317257	1532.711587	278.9573677
DALYs (Disability-Adjusted Life Years)	Global	Both	95+ years	Prostate cancer	Smoking	Rate	2007	27.96964898	57.1796599	10.40684206
DALYs (Disability-Adjusted Life Years)	Global	Both	95+ years	Prostate cancer	Smoking	Number	2008	776.0336224	1591.422561	287.221779
DALYs (Disability-Adjusted Life Years)	Global	Both	95+ years	Prostate cancer	Smoking	Rate	2008	27.46929748	56.33165687	10.1668024
DALYs (Disability-Adjusted Life Years)	Global	Both	95+ years	Prostate cancer	Smoking	Number	2009	789.3906005	1626.029268	289.7085027
DALYs (Disability-Adjusted Life Years)	Global	Both	95+ years	Prostate cancer	Smoking	Rate	2009	26.50493215	54.59628656	9.727382365
DALYs (Disability-Adjusted Life Years)	Global	Both	95+ years	Prostate cancer	Smoking	Number	2010	803.7331271	1676.417835	290.2322546
DALYs (Disability-Adjusted Life Years)	Global	Both	95+ years	Prostate cancer	Smoking	Rate	2010	25.69993144	53.60463813	9.280380257

DALYs (Disability-Adjusted Life Years)	Global	Both	95+ years	Prostate cancer	Smoking	Number	2011	808.75434407	1695.221973	289.2285269
DALYs (Disability-Adjusted Life Years)	Global	Both	95+ years	Prostate cancer	Smoking	Rate	2011	24.84885305	52.08543507	8.88650212
DALYs (Disability-Adjusted Life Years)	Global	Both	95+ years	Prostate cancer	Smoking	Number	2012	803.5965878	1687.324339	287.5957064
DALYs (Disability-Adjusted Life Years)	Global	Both	95+ years	Prostate cancer	Smoking	Rate	2012	23.80574767	49.98530117	8.519736046
DALYs (Disability-Adjusted Life Years)	Global	Both	95+ years	Prostate cancer	Smoking	Number	2013	813.7579827	1730.085309	288.4567328
DALYs (Disability-Adjusted Life Years)	Global	Both	95+ years	Prostate cancer	Smoking	Rate	2013	23.26450038	49.46135236	8.246680107
DALYs (Disability-Adjusted Life Years)	Global	Both	95+ years	Prostate cancer	Smoking	Number	2014	845.8503499	1811.562529	298.5864664
DALYs (Disability-Adjusted Life Years)	Global	Both	95+ years	Prostate cancer	Smoking	Rate	2014	23.19872463	49.68484114	8.189185254
DALYs (Disability-Adjusted Life Years)	Global	Both	95+ years	Prostate cancer	Smoking	Number	2015	890.4795554	1920.144627	315.4971314
DALYs (Disability-Adjusted Life Years)	Global	Both	95+ years	Prostate cancer	Smoking	Rate	2015	22.98081275	49.55361845	8.14210776
DALYs (Disability-Adjusted Life Years)	Global	Both	95+ years	Prostate cancer	Smoking	Number	2016	953.87853	2068.829253	336.9664423

DALYs (Disability-Adjusted Life Years)	Global	Both	95+ years	Prostate cancer	Smoking	Rate	2016	23.08789633	50.07442124	8.156013625
DALYs (Disability-Adjusted Life Years)	Global	Both	95+ years	Prostate cancer	Smoking	Number	2017	1029.40717	2243.517304	362.2640941
DALYs (Disability-Adjusted Life Years)	Global	Both	95+ years	Prostate cancer	Smoking	Rate	2017	23.41204146	51.02482444	8.239054704
DALYs (Disability-Adjusted Life Years)	Global	Both	95+ years	Prostate cancer	Smoking	Number	2018	1101.631665	2422.35293	384.6483824
DALYs (Disability-Adjusted Life Years)	Global	Both	95+ years	Prostate cancer	Smoking	Rate	2018	23.60866069	51.91254955	8.243257192
DALYs (Disability-Adjusted Life Years)	Global	Both	95+ years	Prostate cancer	Smoking	Number	2019	1164.903697	2576.186587	406.1444136
DALYs (Disability-Adjusted Life Years)	Global	Both	95+ years	Prostate cancer	Smoking	Rate	2019	23.52378516	52.02289251	8.201582633
DALYs (Disability-Adjusted Life Years)	Global	Both	95+ years	Prostate cancer	Smoking	Number	2020	1214.953722	2697.533227	429.0067654
DALYs (Disability-Adjusted Life Years)	Global	Both	95+ years	Prostate cancer	Smoking	Rate	2020	23.23927047	51.5976067	8.205912769
DALYs (Disability-Adjusted Life Years)	Global	Both	95+ years	Prostate cancer	Smoking	Number	2021	1231.656547	2749.171497	435.6262369
DALYs (Disability-Adjusted Life Years)	Global	Both	95+ years	Prostate cancer	Smoking	Rate	2021	22.5979228	50.44065687	7.992689275

YLDs (Years Lived with Disability)	Global	Both	30-34 years	Prostate cancer	Smoking	Number	1990	18.70482761	33.77324352	7.900293061
YLDs (Years Lived with Disability)	Global	Both	30-34 years	Prostate cancer	Smoking	Rate	1990	0.004853071	0.008762655	0.002049775
YLDs (Years Lived with Disability)	Global	Both	30-34 years	Prostate cancer	Smoking	Number	1991	19.24528674	34.38372804	8.089657421
YLDs (Years Lived with Disability)	Global	Both	30-34 years	Prostate cancer	Smoking	Rate	1991	0.004927597	0.008803669	0.00207129
YLDs (Years Lived with Disability)	Global	Both	30-34 years	Prostate cancer	Smoking	Number	1992	19.84842351	35.18445014	8.455590309
YLDs (Years Lived with Disability)	Global	Both	30-34 years	Prostate cancer	Smoking	Rate	1992	0.004975601	0.008820035	0.002119647
YLDs (Years Lived with Disability)	Global	Both	30-34 years	Prostate cancer	Smoking	Number	1993	20.52553748	36.5613155	8.568084852
YLDs (Years Lived with Disability)	Global	Both	30-34 years	Prostate cancer	Smoking	Rate	1993	0.004989941	0.008888382	0.002082978
YLDs (Years Lived with Disability)	Global	Both	30-34 years	Prostate cancer	Smoking	Number	1994	21.0267173	37.5236784	8.780025003
YLDs (Years Lived with Disability)	Global	Both	30-34 years	Prostate cancer	Smoking	Rate	1994	0.004939881	0.00881557	0.002062722
YLDs (Years Lived with Disability)	Global	Both	30-34 years	Prostate cancer	Smoking	Number	1995	21.24334344	37.8851557	8.756319001

YLDs (Years Lived with Disability)	Global	Both	30-34 years	Prostate cancer	Smoking	Rate	1995	0.004830812	0.00861522	0.001991218
YLDs (Years Lived with Disability)	Global	Both	30-34 years	Prostate cancer	Smoking	Number	1996	21.53222671	39.88650863	8.774325334
YLDs (Years Lived with Disability)	Global	Both	30-34 years	Prostate cancer	Smoking	Rate	1996	0.004763631	0.008824196	0.001941167
YLDs (Years Lived with Disability)	Global	Both	30-34 years	Prostate cancer	Smoking	Number	1997	21.86703887	39.76228692	8.957854737
YLDs (Years Lived with Disability)	Global	Both	30-34 years	Prostate cancer	Smoking	Rate	1997	0.004730311	0.008601438	0.001937777
YLDs (Years Lived with Disability)	Global	Both	30-34 years	Prostate cancer	Smoking	Number	1998	21.28047337	38.91805631	8.704541377
YLDs (Years Lived with Disability)	Global	Both	30-34 years	Prostate cancer	Smoking	Rate	1998	0.004533245	0.008290468	0.001854274
YLDs (Years Lived with Disability)	Global	Both	30-34 years	Prostate cancer	Smoking	Number	1999	20.51339373	37.51332125	8.359503591
YLDs (Years Lived with Disability)	Global	Both	30-34 years	Prostate cancer	Smoking	Rate	1999	0.004321524	0.007902871	0.001761083
YLDs (Years Lived with Disability)	Global	Both	30-34 years	Prostate cancer	Smoking	Number	2000	20.25930131	36.51007403	8.269708445
YLDs (Years Lived with Disability)	Global	Both	30-34 years	Prostate cancer	Smoking	Rate	2000	0.004228611	0.007620545	0.00172609

YLDs (Years Lived with Disability)	Global	Both	30-34 years	Prostate cancer	Smoking	Number	2001	20.25937638	36.7446393	8.32975040 8
YLDs (Years Lived with Disability)	Global	Both	30-34 years	Prostate cancer	Smoking	Rate	2001	0.004185404	0.00759111	0.001720885 1
YLDs (Years Lived with Disability)	Global	Both	30-34 years	Prostate cancer	Smoking	Number	2002	20.78544773	36.94485326	8.77030628 8
YLDs (Years Lived with Disability)	Global	Both	30-34 years	Prostate cancer	Smoking	Rate	2002	0.004255166	0.007563295	0.001795444
YLDs (Years Lived with Disability)	Global	Both	30-34 years	Prostate cancer	Smoking	Number	2003	21.34663212	37.97868219	9.10283683 9
YLDs (Years Lived with Disability)	Global	Both	30-34 years	Prostate cancer	Smoking	Rate	2003	0.004335833	0.007714061	0.001848928
YLDs (Years Lived with Disability)	Global	Both	30-34 years	Prostate cancer	Smoking	Number	2004	21.81502915	39.04639545	9.34937999 1
YLDs (Years Lived with Disability)	Global	Both	30-34 years	Prostate cancer	Smoking	Rate	2004	0.004404686	0.007883882	0.001887739
YLDs (Years Lived with Disability)	Global	Both	30-34 years	Prostate cancer	Smoking	Number	2005	22.71082299	40.1131964	9.33390099 6
YLDs (Years Lived with Disability)	Global	Both	30-34 years	Prostate cancer	Smoking	Rate	2005	0.004568879	0.008069823	0.001877759
YLDs (Years Lived with Disability)	Global	Both	30-34 years	Prostate cancer	Smoking	Number	2006	23.14906937	41.46208374	9.40541768 6

YLDs (Years Lived with Disability)	Global	Both	30-34 years	Prostate cancer	Smoking	Rate	2006	0.004650018	0.008328605	0.001889293
YLDs (Years Lived with Disability)	Global	Both	30-34 years	Prostate cancer	Smoking	Number	2007	23.17407476	41.7272533	9.317154793
YLDs (Years Lived with Disability)	Global	Both	30-34 years	Prostate cancer	Smoking	Rate	2007	0.004650782	0.008374201	0.001869851
YLDs (Years Lived with Disability)	Global	Both	30-34 years	Prostate cancer	Smoking	Number	2008	23.26158819	41.9587625 8	9.376657305
YLDs (Years Lived with Disability)	Global	Both	30-34 years	Prostate cancer	Smoking	Rate	2008	0.004654888	0.008396388	0.001876367
YLDs (Years Lived with Disability)	Global	Both	30-34 years	Prostate cancer	Smoking	Number	2009	23.32409282	42.1413910 6	9.305942508
YLDs (Years Lived with Disability)	Global	Both	30-34 years	Prostate cancer	Smoking	Rate	2009	0.004636088	0.008376369	0.001849726
YLDs (Years Lived with Disability)	Global	Both	30-34 years	Prostate cancer	Smoking	Number	2010	23.79251077	42.5697483 1	9.421246901
YLDs (Years Lived with Disability)	Global	Both	30-34 years	Prostate cancer	Smoking	Rate	2010	0.004681161	0.00837557	0.001853624
YLDs (Years Lived with Disability)	Global	Both	30-34 years	Prostate cancer	Smoking	Number	2011	24.69015219	44.2473513 4	9.963621755
YLDs (Years Lived with Disability)	Global	Both	30-34 years	Prostate cancer	Smoking	Rate	2011	0.004799192	0.008600657	0.001936697

YLDs (Years Lived with Disability)	Global	Both	30-34 years	Prostate cancer	Smoking	Number	2012	25.10600014	44.92862085	10.1224756
YLDs (Years Lived with Disability)	Global	Both	30-34 years	Prostate cancer	Smoking	Rate	2012	0.004810269	0.008608252	0.00193945
YLDs (Years Lived with Disability)	Global	Both	30-34 years	Prostate cancer	Smoking	Number	2013	25.21979146	44.9427683	10.12400008
YLDs (Years Lived with Disability)	Global	Both	30-34 years	Prostate cancer	Smoking	Rate	2013	0.004750825	0.008466177	0.00190712 7
YLDs (Years Lived with Disability)	Global	Both	30-34 years	Prostate cancer	Smoking	Number	2014	26.71574921	47.59496582	10.78669407
YLDs (Years Lived with Disability)	Global	Both	30-34 years	Prostate cancer	Smoking	Rate	2014	0.004937414	0.008796162	0.00199352
YLDs (Years Lived with Disability)	Global	Both	30-34 years	Prostate cancer	Smoking	Number	2015	27.19984492	48.85813636	10.76929881
YLDs (Years Lived with Disability)	Global	Both	30-34 years	Prostate cancer	Smoking	Rate	2015	0.004921719	0.008840713	0.00194866 8
YLDs (Years Lived with Disability)	Global	Both	30-34 years	Prostate cancer	Smoking	Number	2016	28.2144482	50.95589772	11.34514574
YLDs (Years Lived with Disability)	Global	Both	30-34 years	Prostate cancer	Smoking	Rate	2016	0.004987495	0.009007524	0.00200549 2
YLDs (Years Lived with Disability)	Global	Both	30-34 years	Prostate cancer	Smoking	Number	2017	30.04033848	54.07672148	11.85713426

YLDs (Years Lived with Disability)	Global	Both	30-34 years	Prostate cancer	Smoking	Rate	2017	0.00518995	0.009342621	0.0020485 1
YLDs (Years Lived with Disability)	Global	Both	30-34 years	Prostate cancer	Smoking	Number	2018	31.87300244	57.32818394	12.43217732
YLDs (Years Lived with Disability)	Global	Both	30-34 years	Prostate cancer	Smoking	Rate	2018	0.005399083	0.009711028	0.002105931
YLDs (Years Lived with Disability)	Global	Both	30-34 years	Prostate cancer	Smoking	Number	2019	33.04719402	62.32720781	12.66870107
YLDs (Years Lived with Disability)	Global	Both	30-34 years	Prostate cancer	Smoking	Rate	2019	0.005516484	0.010404123	0.002114754
YLDs (Years Lived with Disability)	Global	Both	30-34 years	Prostate cancer	Smoking	Number	2020	32.52974844	60.87363943	12.97646544
YLDs (Years Lived with Disability)	Global	Both	30-34 years	Prostate cancer	Smoking	Rate	2020	0.005385408	0.010077834	0.002148297
YLDs (Years Lived with Disability)	Global	Both	30-34 years	Prostate cancer	Smoking	Number	2021	32.72259913	60.49776328	13.2201364
YLDs (Years Lived with Disability)	Global	Both	30-34 years	Prostate cancer	Smoking	Rate	2021	0.005413345	0.01000823	0.002187026
YLDs (Years Lived with Disability)	Global	Both	35-39 years	Prostate cancer	Smoking	Number	1990	36.15235757	67.17548593	14.34508007
YLDs (Years Lived with Disability)	Global	Both	35-39 years	Prostate cancer	Smoking	Rate	1990	0.010263428	0.0190707	0.004072478

YLDs (Years Lived with Disability)	Global	Both	35-39 years	Prostate cancer	Smoking	Number	1991	37.86755059	69.54314038	14.55549664
YLDs (Years Lived with Disability)	Global	Both	35-39 years	Prostate cancer	Smoking	Rate	1991	0.010486967	0.019259145	0.004030971
YLDs (Years Lived with Disability)	Global	Both	35-39 years	Prostate cancer	Smoking	Number	1992	39.68561853	72.86220027	15.50448793
YLDs (Years Lived with Disability)	Global	Both	35-39 years	Prostate cancer	Smoking	Rate	1992	0.010772579	0.019778293	0.004208661
YLDs (Years Lived with Disability)	Global	Both	35-39 years	Prostate cancer	Smoking	Number	1993	41.3562239	76.02856582	16.36777151
YLDs (Years Lived with Disability)	Global	Both	35-39 years	Prostate cancer	Smoking	Rate	1993	0.011078258	0.020366077	0.004384501
YLDs (Years Lived with Disability)	Global	Both	35-39 years	Prostate cancer	Smoking	Number	1994	41.58176564	75.45453494	16.737027
YLDs (Years Lived with Disability)	Global	Both	35-39 years	Prostate cancer	Smoking	Rate	1994	0.011045066	0.020042446	0.004445736
YLDs (Years Lived with Disability)	Global	Both	35-39 years	Prostate cancer	Smoking	Number	1995	41.49206691	76.55730575	16.68307157
YLDs (Years Lived with Disability)	Global	Both	35-39 years	Prostate cancer	Smoking	Rate	1995	0.010933283	0.020173077	0.004396039
YLDs (Years Lived with Disability)	Global	Both	35-39 years	Prostate cancer	Smoking	Number	1996	41.49455779	76.19694485	16.5153069

YLDs (Years Lived with Disability)	Global	Both	35-39 years	Prostate cancer	Smoking	Rate	1996	0.010789448	0.019812791	0.00429432 3
YLDs (Years Lived with Disability)	Global	Both	35-39 years	Prostate cancer	Smoking	Number	1997	41.91115441	76.87922454	16.84709055
YLDs (Years Lived with Disability)	Global	Both	35-39 years	Prostate cancer	Smoking	Rate	1997	0.010663692	0.019560816	0.0042865
YLDs (Years Lived with Disability)	Global	Both	35-39 years	Prostate cancer	Smoking	Number	1998	41.7477309	77.02522872	16.77537654
YLDs (Years Lived with Disability)	Global	Both	35-39 years	Prostate cancer	Smoking	Rate	1998	0.010293319	0.018991338	0.00413613 6
YLDs (Years Lived with Disability)	Global	Both	35-39 years	Prostate cancer	Smoking	Number	1999	41.8094499	76.6484326	16.91252163
YLDs (Years Lived with Disability)	Global	Both	35-39 years	Prostate cancer	Smoking	Rate	1999	0.009952829	0.018246324	0.00402606 2
YLDs (Years Lived with Disability)	Global	Both	35-39 years	Prostate cancer	Smoking	Number	2000	42.32174052	78.56224576	17.21264286
YLDs (Years Lived with Disability)	Global	Both	35-39 years	Prostate cancer	Smoking	Rate	2000	0.009743535	0.018087017	0.00396278 6
YLDs (Years Lived with Disability)	Global	Both	35-39 years	Prostate cancer	Smoking	Number	2001	42.13380871	78.39405166	17.09289469
YLDs (Years Lived with Disability)	Global	Both	35-39 years	Prostate cancer	Smoking	Rate	2001	0.00943329	0.017551555	0.00382690 9

YLDs (Years Lived with Disability)	Global	Both	35-39 years	Prostate cancer	Smoking	Number	2002	42.76638128	77.42460129	17.72433427
YLDs (Years Lived with Disability)	Global	Both	35-39 years	Prostate cancer	Smoking	Rate	2002	0.009358731	0.016943123	0.003878684
YLDs (Years Lived with Disability)	Global	Both	35-39 years	Prostate cancer	Smoking	Number	2003	41.55748166	75.65842298	17.13930949
YLDs (Years Lived with Disability)	Global	Both	35-39 years	Prostate cancer	Smoking	Rate	2003	0.008950103	0.016294314	0.003691239
YLDs (Years Lived with Disability)	Global	Both	35-39 years	Prostate cancer	Smoking	Number	2004	41.07589396	73.59730014	17.19414085
YLDs (Years Lived with Disability)	Global	Both	35-39 years	Prostate cancer	Smoking	Rate	2004	0.008740396	0.015660513	0.003658681
YLDs (Years Lived with Disability)	Global	Both	35-39 years	Prostate cancer	Smoking	Number	2005	41.5173926	73.70877614	16.99253073
YLDs (Years Lived with Disability)	Global	Both	35-39 years	Prostate cancer	Smoking	Rate	2005	0.008743512	0.015522977	0.003578606
YLDs (Years Lived with Disability)	Global	Both	35-39 years	Prostate cancer	Smoking	Number	2006	41.96209647	73.14180447	17.24956728
YLDs (Years Lived with Disability)	Global	Both	35-39 years	Prostate cancer	Smoking	Rate	2006	0.008737397	0.015229672	0.003591725
YLDs (Years Lived with Disability)	Global	Both	35-39 years	Prostate cancer	Smoking	Number	2007	41.93158948	74.68745964	17.08153666

YLDs (Years Lived with Disability)	Global	Both	35-39 years	Prostate cancer	Smoking	Rate	2007	0.008643354	0.015395318	0.003521015
YLDs (Years Lived with Disability)	Global	Both	35-39 years	Prostate cancer	Smoking	Number	2008	41.49732866	73.16029604	16.93874278
YLDs (Years Lived with Disability)	Global	Both	35-39 years	Prostate cancer	Smoking	Rate	2008	0.008478387	0.0149475	0.003460782
YLDs (Years Lived with Disability)	Global	Both	35-39 years	Prostate cancer	Smoking	Number	2009	40.53290165	71.12357151	16.52052111
YLDs (Years Lived with Disability)	Global	Both	35-39 years	Prostate cancer	Smoking	Rate	2009	0.008224996	0.014432499	0.003352368
YLDs (Years Lived with Disability)	Global	Both	35-39 years	Prostate cancer	Smoking	Number	2010	40.05273781	69.87010757	16.07643213
YLDs (Years Lived with Disability)	Global	Both	35-39 years	Prostate cancer	Smoking	Rate	2010	0.00809552	0.01412253	0.003249393
YLDs (Years Lived with Disability)	Global	Both	35-39 years	Prostate cancer	Smoking	Number	2011	39.80698592	70.01661488	16.14093468
YLDs (Years Lived with Disability)	Global	Both	35-39 years	Prostate cancer	Smoking	Rate	2011	0.008036161	0.014134825	0.003258502
YLDs (Years Lived with Disability)	Global	Both	35-39 years	Prostate cancer	Smoking	Number	2012	39.12237185	67.64154822	15.5942131
YLDs (Years Lived with Disability)	Global	Both	35-39 years	Prostate cancer	Smoking	Rate	2012	0.007894695	0.013649719	0.003146833

YLDs (Years Lived with Disability)	Global	Both	35-39 years	Prostate cancer	Smoking	Number	2013	38.30575902	66.94626983	15.38068781
YLDs (Years Lived with Disability)	Global	Both	35-39 years	Prostate cancer	Smoking	Rate	2013	0.00771462	0.0134827	0.003097606
YLDs (Years Lived with Disability)	Global	Both	35-39 years	Prostate cancer	Smoking	Number	2014	39.72092565	68.81324964	16.58604758
YLDs (Years Lived with Disability)	Global	Both	35-39 years	Prostate cancer	Smoking	Rate	2014	0.007954839	0.013781108	0.003321658
YLDs (Years Lived with Disability)	Global	Both	35-39 years	Prostate cancer	Smoking	Number	2015	39.63353057	68.38334337	16.13138772
YLDs (Years Lived with Disability)	Global	Both	35-39 years	Prostate cancer	Smoking	Rate	2015	0.007859949	0.013561487	0.003199107
YLDs (Years Lived with Disability)	Global	Both	35-39 years	Prostate cancer	Smoking	Number	2016	39.81990341	69.65371937	16.43716924
YLDs (Years Lived with Disability)	Global	Both	35-39 years	Prostate cancer	Smoking	Rate	2016	0.007801288	0.013646159	0.003220276
YLDs (Years Lived with Disability)	Global	Both	35-39 years	Prostate cancer	Smoking	Number	2017	40.69806172	70.73936973	16.59170073
YLDs (Years Lived with Disability)	Global	Both	35-39 years	Prostate cancer	Smoking	Rate	2017	0.007860069	0.013661986	0.003204377
YLDs (Years Lived with Disability)	Global	Both	35-39 years	Prostate cancer	Smoking	Number	2018	42.1124235	73.55977421	16.92035408

YLDs (Years Lived with Disability)	Global	Both	35-39 years	Prostate cancer	Smoking	Rate	2018	0.00799756	0.013969718	0.0032133 4
YLDs (Years Lived with Disability)	Global	Both	35-39 years	Prostate cancer	Smoking	Number	2019	42.83327523	78.0062544	17.0664140 1
YLDs (Years Lived with Disability)	Global	Both	35-39 years	Prostate cancer	Smoking	Rate	2019	0.007980974	0.014534633	0.00317992 5
YLDs (Years Lived with Disability)	Global	Both	35-39 years	Prostate cancer	Smoking	Number	2020	42.51480735	76.14941078	17.2434479 4
YLDs (Years Lived with Disability)	Global	Both	35-39 years	Prostate cancer	Smoking	Rate	2020	0.007757065	0.013893887	0.00314616 4
YLDs (Years Lived with Disability)	Global	Both	35-39 years	Prostate cancer	Smoking	Number	2021	44.03620896	78.24913664	17.9756358 3
YLDs (Years Lived with Disability)	Global	Both	35-39 years	Prostate cancer	Smoking	Rate	2021	0.007851466	0.013951483	0.00320497 8
YLDs (Years Lived with Disability)	Global	Both	40-44 years	Prostate cancer	Smoking	Number	1990	78.43241388	135.9792663	32.2905818 7
YLDs (Years Lived with Disability)	Global	Both	40-44 years	Prostate cancer	Smoking	Rate	1990	0.027377816	0.047465265	0.01127143 2
YLDs (Years Lived with Disability)	Global	Both	40-44 years	Prostate cancer	Smoking	Number	1991	86.62963142	149.0384356	36.3357599 9
YLDs (Years Lived with Disability)	Global	Both	40-44 years	Prostate cancer	Smoking	Rate	1991	0.028825653	0.049591925	0.01209057 4

YLDs (Years Lived with Disability)	Global	Both	40-44 years	Prostate cancer	Smoking	Number	1992	92.55134056	159.5361871	37.76569662
YLDs (Years Lived with Disability)	Global	Both	40-44 years	Prostate cancer	Smoking	Rate	1992	0.029787324	0.051346162	0.012154757
YLDs (Years Lived with Disability)	Global	Both	40-44 years	Prostate cancer	Smoking	Number	1993	99.01141668	170.3527427	40.88361739
YLDs (Years Lived with Disability)	Global	Both	40-44 years	Prostate cancer	Smoking	Rate	1993	0.030761717	0.052926653	0.012702073
YLDs (Years Lived with Disability)	Global	Both	40-44 years	Prostate cancer	Smoking	Number	1994	103.399329	177.6592864	42.64794115
YLDs (Years Lived with Disability)	Global	Both	40-44 years	Prostate cancer	Smoking	Rate	1994	0.031255569	0.053702883	0.012891628
YLDs (Years Lived with Disability)	Global	Both	40-44 years	Prostate cancer	Smoking	Number	1995	109.7996402	191.0489919	44.98213986
YLDs (Years Lived with Disability)	Global	Both	40-44 years	Prostate cancer	Smoking	Rate	1995	0.031976265	0.055638007	0.013099868
YLDs (Years Lived with Disability)	Global	Both	40-44 years	Prostate cancer	Smoking	Number	1996	113.9171668	199.7391682	47.18050835
YLDs (Years Lived with Disability)	Global	Both	40-44 years	Prostate cancer	Smoking	Rate	1996	0.0324449	0.056887978	0.013437543
YLDs (Years Lived with Disability)	Global	Both	40-44 years	Prostate cancer	Smoking	Number	1997	116.854813	203.8542753	48.41419778

YLDs (Years Lived with Disability)	Global	Both	40-44 years	Prostate cancer	Smoking	Rate	1997	0.032685271	0.057019751	0.013541857
YLDs (Years Lived with Disability)	Global	Both	40-44 years	Prostate cancer	Smoking	Number	1998	119.9004582	211.6764245	49.43330425
YLDs (Years Lived with Disability)	Global	Both	40-44 years	Prostate cancer	Smoking	Rate	1998	0.033135478	0.058498522	0.0136613
YLDs (Years Lived with Disability)	Global	Both	40-44 years	Prostate cancer	Smoking	Number	1999	120.6046744	212.1748545	49.39915139
YLDs (Years Lived with Disability)	Global	Both	40-44 years	Prostate cancer	Smoking	Rate	1999	0.033062359	0.058165252	0.013542199
YLDs (Years Lived with Disability)	Global	Both	40-44 years	Prostate cancer	Smoking	Number	2000	120.1550939	210.9766667	48.79361114
YLDs (Years Lived with Disability)	Global	Both	40-44 years	Prostate cancer	Smoking	Rate	2000	0.032665711	0.057356726	0.013265172
YLDs (Years Lived with Disability)	Global	Both	40-44 years	Prostate cancer	Smoking	Number	2001	121.9295111	213.7346347	49.7111619
YLDs (Years Lived with Disability)	Global	Both	40-44 years	Prostate cancer	Smoking	Rate	2001	0.032686271	0.057296943	0.013326327
YLDs (Years Lived with Disability)	Global	Both	40-44 years	Prostate cancer	Smoking	Number	2002	122.1152774	216.3529387	49.74123446
YLDs (Years Lived with Disability)	Global	Both	40-44 years	Prostate cancer	Smoking	Rate	2002	0.032004563	0.056702825	0.013036423

YLDs (Years Lived with Disability)	Global	Both	40-44 years	Prostate cancer	Smoking	Number	2003	121.0973226	215.1673829	49.82067445
YLDs (Years Lived with Disability)	Global	Both	40-44 years	Prostate cancer	Smoking	Rate	2003	0.030726571	0.054595393	0.012641225
YLDs (Years Lived with Disability)	Global	Both	40-44 years	Prostate cancer	Smoking	Number	2004	123.5615876	214.5968689	50.68751018
YLDs (Years Lived with Disability)	Global	Both	40-44 years	Prostate cancer	Smoking	Rate	2004	0.030236485	0.052513528	0.012403629
YLDs (Years Lived with Disability)	Global	Both	40-44 years	Prostate cancer	Smoking	Number	2005	129.5129859	223.6549995	52.67755575
YLDs (Years Lived with Disability)	Global	Both	40-44 years	Prostate cancer	Smoking	Rate	2005	0.030614305	0.052867613	0.012451931
YLDs (Years Lived with Disability)	Global	Both	40-44 years	Prostate cancer	Smoking	Number	2006	135.7353328	236.5427985	54.75066622
YLDs (Years Lived with Disability)	Global	Both	40-44 years	Prostate cancer	Smoking	Rate	2006	0.031168735	0.054317028	0.012572327
YLDs (Years Lived with Disability)	Global	Both	40-44 years	Prostate cancer	Smoking	Number	2007	132.3060778	236.6736852	54.93774943
YLDs (Years Lived with Disability)	Global	Both	40-44 years	Prostate cancer	Smoking	Rate	2007	0.029661576	0.053059652	0.012316443
YLDs (Years Lived with Disability)	Global	Both	40-44 years	Prostate cancer	Smoking	Number	2008	130.558785	228.4375853	52.7551641

YLDs (Years Lived with Disability)	Global	Both	40-44 years	Prostate cancer	Smoking	Rate	2008	0.028772864	0.050343634	0.011626312
YLDs (Years Lived with Disability)	Global	Both	40-44 years	Prostate cancer	Smoking	Number	2009	128.225652	223.2656203	51.47778631
YLDs (Years Lived with Disability)	Global	Both	40-44 years	Prostate cancer	Smoking	Rate	2009	0.027891229	0.048564016	0.011197282
YLDs (Years Lived with Disability)	Global	Both	40-44 years	Prostate cancer	Smoking	Number	2010	126.8739892	220.7597368	51.32938954
YLDs (Years Lived with Disability)	Global	Both	40-44 years	Prostate cancer	Smoking	Rate	2010	0.027295163	0.047493368	0.0110428
YLDs (Years Lived with Disability)	Global	Both	40-44 years	Prostate cancer	Smoking	Number	2011	127.6222082	222.7652904	52.51863854
YLDs (Years Lived with Disability)	Global	Both	40-44 years	Prostate cancer	Smoking	Rate	2011	0.027136013	0.047366065	0.011166916
YLDs (Years Lived with Disability)	Global	Both	40-44 years	Prostate cancer	Smoking	Number	2012	127.2998101	220.5113042	49.96846309
YLDs (Years Lived with Disability)	Global	Both	40-44 years	Prostate cancer	Smoking	Rate	2012	0.026789543	0.046405388	0.010515588
YLDs (Years Lived with Disability)	Global	Both	40-44 years	Prostate cancer	Smoking	Number	2013	127.5859832	224.5973824	50.85281202
YLDs (Years Lived with Disability)	Global	Both	40-44 years	Prostate cancer	Smoking	Rate	2013	0.026613672	0.046849668	0.010607592

YLDs (Years Lived with Disability)	Global	Both	40-44 years	Prostate cancer	Smoking	Number	2014	129.9768511	233.2845082	52.16528326
YLDs (Years Lived with Disability)	Global	Both	40-44 years	Prostate cancer	Smoking	Rate	2014	0.026934615	0.048342672	0.010810016
YLDs (Years Lived with Disability)	Global	Both	40-44 years	Prostate cancer	Smoking	Number	2015	129.7525749	231.1387704	52.29613971
YLDs (Years Lived with Disability)	Global	Both	40-44 years	Prostate cancer	Smoking	Rate	2015	0.026782868	0.047710492	0.010794704
YLDs (Years Lived with Disability)	Global	Both	40-44 years	Prostate cancer	Smoking	Number	2016	129.3429143	232.2441454	52.19290658
YLDs (Years Lived with Disability)	Global	Both	40-44 years	Prostate cancer	Smoking	Rate	2016	0.026662058	0.047873569	0.010758767
YLDs (Years Lived with Disability)	Global	Both	40-44 years	Prostate cancer	Smoking	Number	2017	127.4599548	225.8267926	50.85949754
YLDs (Years Lived with Disability)	Global	Both	40-44 years	Prostate cancer	Smoking	Rate	2017	0.026260221	0.046526467	0.010478441
YLDs (Years Lived with Disability)	Global	Both	40-44 years	Prostate cancer	Smoking	Number	2018	132.7946716	235.2820656	53.42241675
YLDs (Years Lived with Disability)	Global	Both	40-44 years	Prostate cancer	Smoking	Rate	2018	0.027302504	0.04837385	0.010983616
YLDs (Years Lived with Disability)	Global	Both	40-44 years	Prostate cancer	Smoking	Number	2019	135.8592224	244.9079029	54.25489204

YLDs (Years Lived with Disability)	Global	Both	40-44 years	Prostate cancer	Smoking	Rate	2019	0.02776906	0.050058156	0.011089474
YLDs (Years Lived with Disability)	Global	Both	40-44 years	Prostate cancer	Smoking	Number	2020	134.2515685	239.3693089	52.91272048
YLDs (Years Lived with Disability)	Global	Both	40-44 years	Prostate cancer	Smoking	Rate	2020	0.027165863	0.048436484	0.010706912
YLDs (Years Lived with Disability)	Global	Both	40-44 years	Prostate cancer	Smoking	Number	2021	136.029059	241.9014381	55.71036572
YLDs (Years Lived with Disability)	Global	Both	40-44 years	Prostate cancer	Smoking	Rate	2021	0.027192172	0.048356033	0.011136487
YLDs (Years Lived with Disability)	Global	Both	45-49 years	Prostate cancer	Smoking	Number	1990	237.4071852	405.1100503	101.5367177
YLDs (Years Lived with Disability)	Global	Both	45-49 years	Prostate cancer	Smoking	Rate	1990	0.102244528	0.174469386	0.043728979
YLDs (Years Lived with Disability)	Global	Both	45-49 years	Prostate cancer	Smoking	Number	1991	249.1569885	422.6115856	104.8999724
YLDs (Years Lived with Disability)	Global	Both	45-49 years	Prostate cancer	Smoking	Rate	1991	0.105849359	0.179538073	0.044564654
YLDs (Years Lived with Disability)	Global	Both	45-49 years	Prostate cancer	Smoking	Number	1992	272.7273513	464.8274461	114.7371436
YLDs (Years Lived with Disability)	Global	Both	45-49 years	Prostate cancer	Smoking	Rate	1992	0.111960918	0.190822472	0.047102265

YLDs (Years Lived with Disability)	Global	Both	45-49 years	Prostate cancer	Smoking	Number	1993	295.1455879	504.3812831	121.695901
YLDs (Years Lived with Disability)	Global	Both	45-49 years	Prostate cancer	Smoking	Rate	1993	0.116864574	0.199712637	0.048186061
YLDs (Years Lived with Disability)	Global	Both	45-49 years	Prostate cancer	Smoking	Number	1994	324.976922	564.409515	134.7973756
YLDs (Years Lived with Disability)	Global	Both	45-49 years	Prostate cancer	Smoking	Rate	1994	0.122526101	0.21279941	0.050822676
YLDs (Years Lived with Disability)	Global	Both	45-49 years	Prostate cancer	Smoking	Number	1995	348.961965	601.8244337	146.8131182
YLDs (Years Lived with Disability)	Global	Both	45-49 years	Prostate cancer	Smoking	Rate	1995	0.12662253	0.218374894	0.053271847
YLDs (Years Lived with Disability)	Global	Both	45-49 years	Prostate cancer	Smoking	Number	1996	378.1755499	646.0637012	157.3038526
YLDs (Years Lived with Disability)	Global	Both	45-49 years	Prostate cancer	Smoking	Rate	1996	0.130990649	0.223780473	0.054486161
YLDs (Years Lived with Disability)	Global	Both	45-49 years	Prostate cancer	Smoking	Number	1997	389.7736878	671.1465327	166.1484689
YLDs (Years Lived with Disability)	Global	Both	45-49 years	Prostate cancer	Smoking	Rate	1997	0.130764331	0.225161498	0.05574079
YLDs (Years Lived with Disability)	Global	Both	45-49 years	Prostate cancer	Smoking	Number	1998	401.3230633	691.3500716	172.4271413

YLDs (Years Lived with Disability)	Global	Both	45-49 years	Prostate cancer	Smoking	Rate	1998	0.130088946	0.224101255	0.0558922 9
YLDs (Years Lived with Disability)	Global	Both	45-49 years	Prostate cancer	Smoking	Number	1999	415.9255022	730.0341322	178.3089141
YLDs (Years Lived with Disability)	Global	Both	45-49 years	Prostate cancer	Smoking	Rate	1999	0.131252285	0.230374545	0.056268376
YLDs (Years Lived with Disability)	Global	Both	45-49 years	Prostate cancer	Smoking	Number	2000	432.3941981	750.7741751	183.680145
YLDs (Years Lived with Disability)	Global	Both	45-49 years	Prostate cancer	Smoking	Rate	2000	0.131440465	0.228222551	0.055835633
YLDs (Years Lived with Disability)	Global	Both	45-49 years	Prostate cancer	Smoking	Number	2001	441.8378898	771.5220874	188.3684855
YLDs (Years Lived with Disability)	Global	Both	45-49 years	Prostate cancer	Smoking	Rate	2001	0.131238169	0.229163566	0.055950691
YLDs (Years Lived with Disability)	Global	Both	45-49 years	Prostate cancer	Smoking	Number	2002	447.718424	775.1765835	189.3387823
YLDs (Years Lived with Disability)	Global	Both	45-49 years	Prostate cancer	Smoking	Rate	2002	0.130454692	0.225868351	0.055168899
YLDs (Years Lived with Disability)	Global	Both	45-49 years	Prostate cancer	Smoking	Number	2003	459.4143897	803.8952482	191.6945534
YLDs (Years Lived with Disability)	Global	Both	45-49 years	Prostate cancer	Smoking	Rate	2003	0.132081172	0.23111907	0.05511199

YLDs (Years Lived with Disability)	Global	Both	45-49 years	Prostate cancer	Smoking	Number	2004	469.8446106	812.276537	196.132131
YLDs (Years Lived with Disability)	Global	Both	45-49 years	Prostate cancer	Smoking	Rate	2004	0.133793894	0.231305496	0.055851287
YLDs (Years Lived with Disability)	Global	Both	45-49 years	Prostate cancer	Smoking	Number	2005	482.8872924	843.3795847	201.9101554
YLDs (Years Lived with Disability)	Global	Both	45-49 years	Prostate cancer	Smoking	Rate	2005	0.13617159	0.237828455	0.056937565
YLDs (Years Lived with Disability)	Global	Both	45-49 years	Prostate cancer	Smoking	Number	2006	491.399284	853.6912756	204.8487265
YLDs (Years Lived with Disability)	Global	Both	45-49 years	Prostate cancer	Smoking	Rate	2006	0.136457176	0.237062415	0.056884655
YLDs (Years Lived with Disability)	Global	Both	45-49 years	Prostate cancer	Smoking	Number	2007	490.3750896	857.8322318	205.9181706
YLDs (Years Lived with Disability)	Global	Both	45-49 years	Prostate cancer	Smoking	Rate	2007	0.132937112	0.232552064	0.055822915
YLDs (Years Lived with Disability)	Global	Both	45-49 years	Prostate cancer	Smoking	Number	2008	495.1646029	868.1298408	207.8451984
YLDs (Years Lived with Disability)	Global	Both	45-49 years	Prostate cancer	Smoking	Rate	2008	0.12974159	0.227464857	0.054458995
YLDs (Years Lived with Disability)	Global	Both	45-49 years	Prostate cancer	Smoking	Number	2009	499.1261994	862.8327829	203.3963776

YLDs (Years Lived with Disability)	Global	Both	45-49 years	Prostate cancer	Smoking	Rate	2009	0.125921047	0.217678029	0.051313445
YLDs (Years Lived with Disability)	Global	Both	45-49 years	Prostate cancer	Smoking	Number	2010	507.1556291	884.6441341	210.0635835
YLDs (Years Lived with Disability)	Global	Both	45-49 years	Prostate cancer	Smoking	Rate	2010	0.123424505	0.215292424	0.051122362
YLDs (Years Lived with Disability)	Global	Both	45-49 years	Prostate cancer	Smoking	Number	2011	494.2759634	861.2659767	204.6026944
YLDs (Years Lived with Disability)	Global	Both	45-49 years	Prostate cancer	Smoking	Rate	2011	0.11674506	0.203425931	0.048325946
YLDs (Years Lived with Disability)	Global	Both	45-49 years	Prostate cancer	Smoking	Number	2012	488.1941271	856.6288241	201.3357622
YLDs (Years Lived with Disability)	Global	Both	45-49 years	Prostate cancer	Smoking	Rate	2012	0.11248112	0.197369374	0.046388252
YLDs (Years Lived with Disability)	Global	Both	45-49 years	Prostate cancer	Smoking	Number	2013	472.8611519	827.45264	195.1746238
YLDs (Years Lived with Disability)	Global	Both	45-49 years	Prostate cancer	Smoking	Rate	2013	0.107034402	0.187297896	0.044178717
YLDs (Years Lived with Disability)	Global	Both	45-49 years	Prostate cancer	Smoking	Number	2014	458.5965856	804.8291114	187.2380872
YLDs (Years Lived with Disability)	Global	Both	45-49 years	Prostate cancer	Smoking	Rate	2014	0.102418266	0.179742294	0.041815837

YLDs (Years Lived with Disability)	Global	Both	45-49 years	Prostate cancer	Smoking	Number	2015	444.5145803	763.4545956	180.8219565
YLDs (Years Lived with Disability)	Global	Both	45-49 years	Prostate cancer	Smoking	Rate	2015	0.098140479	0.16855645	0.039922095
YLDs (Years Lived with Disability)	Global	Both	45-49 years	Prostate cancer	Smoking	Number	2016	444.1960933	787.6606281	179.2526646
YLDs (Years Lived with Disability)	Global	Both	45-49 years	Prostate cancer	Smoking	Rate	2016	0.096856903	0.171749302	0.039086021
YLDs (Years Lived with Disability)	Global	Both	45-49 years	Prostate cancer	Smoking	Number	2017	446.3404466	781.7912815	178.3178617
YLDs (Years Lived with Disability)	Global	Both	45-49 years	Prostate cancer	Smoking	Rate	2017	0.096270623	0.168623602	0.038461161
YLDs (Years Lived with Disability)	Global	Both	45-49 years	Prostate cancer	Smoking	Number	2018	453.70657	787.8725991	186.0825916
YLDs (Years Lived with Disability)	Global	Both	45-49 years	Prostate cancer	Smoking	Rate	2018	0.096953816	0.168362682	0.039764505
YLDs (Years Lived with Disability)	Global	Both	45-49 years	Prostate cancer	Smoking	Number	2019	455.5684674	809.5461359	182.2102785
YLDs (Years Lived with Disability)	Global	Both	45-49 years	Prostate cancer	Smoking	Rate	2019	0.096676404	0.171794175	0.038666931
YLDs (Years Lived with Disability)	Global	Both	45-49 years	Prostate cancer	Smoking	Number	2020	445.9775601	796.7892398	177.5792468

YLDs (Years Lived with Disability)	Global	Both	45-49 years	Prostate cancer	Smoking	Rate	2020	0.094269987	0.16842397	0.037536403
YLDs (Years Lived with Disability)	Global	Both	45-49 years	Prostate cancer	Smoking	Number	2021	443.7114157	789.0263254	180.644128 4
YLDs (Years Lived with Disability)	Global	Both	45-49 years	Prostate cancer	Smoking	Rate	2021	0.093707937	0.1666354	0.038150446
YLDs (Years Lived with Disability)	Global	Both	50-54 years	Prostate cancer	Smoking	Number	1990	784.0578225	1309.734782	336.358827 4
YLDs (Years Lived with Disability)	Global	Both	50-54 years	Prostate cancer	Smoking	Rate	1990	0.368844339	0.616138563	0.158233291
YLDs (Years Lived with Disability)	Global	Both	50-54 years	Prostate cancer	Smoking	Number	1991	824.7387548	1372.798823	354.572222 2
YLDs (Years Lived with Disability)	Global	Both	50-54 years	Prostate cancer	Smoking	Rate	1991	0.383811934	0.638864814	0.165008676
YLDs (Years Lived with Disability)	Global	Both	50-54 years	Prostate cancer	Smoking	Number	1992	859.8473573	1431.212819	369.182068 9
YLDs (Years Lived with Disability)	Global	Both	50-54 years	Prostate cancer	Smoking	Rate	1992	0.397513448	0.661659697	0.170675453
YLDs (Years Lived with Disability)	Global	Both	50-54 years	Prostate cancer	Smoking	Number	1993	902.9790031	1513.244395	392.103323
YLDs (Years Lived with Disability)	Global	Both	50-54 years	Prostate cancer	Smoking	Rate	1993	0.415657637	0.696573882	0.180492282

YLDs (Years Lived with Disability)	Global	Both	50-54 years	Prostate cancer	Smoking	Number	1994	949.6253543	1582.029052	415.8602933
YLDs (Years Lived with Disability)	Global	Both	50-54 years	Prostate cancer	Smoking	Rate	1994	0.435310348	0.725205592	0.190631272
YLDs (Years Lived with Disability)	Global	Both	50-54 years	Prostate cancer	Smoking	Number	1995	982.3843031	1643.483705	429.3736019
YLDs (Years Lived with Disability)	Global	Both	50-54 years	Prostate cancer	Smoking	Rate	1995	0.44905407	0.751246782	0.196269385
YLDs (Years Lived with Disability)	Global	Both	50-54 years	Prostate cancer	Smoking	Number	1996	1026.460532	1711.262446	443.1970527
YLDs (Years Lived with Disability)	Global	Both	50-54 years	Prostate cancer	Smoking	Rate	1996	0.463144434	0.772130688	0.19997286
YLDs (Years Lived with Disability)	Global	Both	50-54 years	Prostate cancer	Smoking	Number	1997	1070.329498	1795.128651	463.9308695
YLDs (Years Lived with Disability)	Global	Both	50-54 years	Prostate cancer	Smoking	Rate	1997	0.466216034	0.781925343	0.202079837
YLDs (Years Lived with Disability)	Global	Both	50-54 years	Prostate cancer	Smoking	Number	1998	1126.34415	1887.123083	486.537524
YLDs (Years Lived with Disability)	Global	Both	50-54 years	Prostate cancer	Smoking	Rate	1998	0.472642384	0.791884393	0.204163404
YLDs (Years Lived with Disability)	Global	Both	50-54 years	Prostate cancer	Smoking	Number	1999	1183.527534	1979.206722	513.4188253

YLDs (Years Lived with Disability)	Global	Both	50-54 years	Prostate cancer	Smoking	Rate	1999	0.471936623	0.789217074	0.204727934
YLDs (Years Lived with Disability)	Global	Both	50-54 years	Prostate cancer	Smoking	Number	2000	1248.171818	2103.8967	536.782317 3
YLDs (Years Lived with Disability)	Global	Both	50-54 years	Prostate cancer	Smoking	Rate	2000	0.478005942	0.805718499	0.205568763
YLDs (Years Lived with Disability)	Global	Both	50-54 years	Prostate cancer	Smoking	Number	2001	1350.792814	2277.838267	585.265071 7
YLDs (Years Lived with Disability)	Global	Both	50-54 years	Prostate cancer	Smoking	Rate	2001	0.492933107	0.831231765	0.213575707
YLDs (Years Lived with Disability)	Global	Both	50-54 years	Prostate cancer	Smoking	Number	2002	1393.870864	2354.358965	606.356900 6
YLDs (Years Lived with Disability)	Global	Both	50-54 years	Prostate cancer	Smoking	Rate	2002	0.491876006	0.830817771	0.213974205
YLDs (Years Lived with Disability)	Global	Both	50-54 years	Prostate cancer	Smoking	Number	2003	1433.882973	2410.284559	634.751117 4
YLDs (Years Lived with Disability)	Global	Both	50-54 years	Prostate cancer	Smoking	Rate	2003	0.488010835	0.820321465	0.21603257
YLDs (Years Lived with Disability)	Global	Both	50-54 years	Prostate cancer	Smoking	Number	2004	1475.958734	2505.182161	644.838517 2
YLDs (Years Lived with Disability)	Global	Both	50-54 years	Prostate cancer	Smoking	Rate	2004	0.488135249	0.828524328	0.213263693

YLDs (Years Lived with Disability)	Global	Both	50-54 years	Prostate cancer	Smoking	Number	2005	1539.4512	2569.089938	682.167506 2
YLDs (Years Lived with Disability)	Global	Both	50-54 years	Prostate cancer	Smoking	Rate	2005	0.48943269	0.816782369	0.216879286
YLDs (Years Lived with Disability)	Global	Both	50-54 years	Prostate cancer	Smoking	Number	2006	1583.103505	2644.158983	694.572202 8
YLDs (Years Lived with Disability)	Global	Both	50-54 years	Prostate cancer	Smoking	Rate	2006	0.49080980 2	0.819768981	0.215338318
YLDs (Years Lived with Disability)	Global	Both	50-54 years	Prostate cancer	Smoking	Number	2007	1613.62989	2704.907953	703.994242 1
YLDs (Years Lived with Disability)	Global	Both	50-54 years	Prostate cancer	Smoking	Rate	2007	0.48988337 1	0.821185474	0.213726254
YLDs (Years Lived with Disability)	Global	Both	50-54 years	Prostate cancer	Smoking	Number	2008	1663.610288	2817.072053	715.879783 3
YLDs (Years Lived with Disability)	Global	Both	50-54 years	Prostate cancer	Smoking	Rate	2008	0.49756377 9	0.842548898	0.214110151
YLDs (Years Lived with Disability)	Global	Both	50-54 years	Prostate cancer	Smoking	Number	2009	1674.718362	2824.023428	719.561088 3
YLDs (Years Lived with Disability)	Global	Both	50-54 years	Prostate cancer	Smoking	Rate	2009	0.49544165	0.835447236	0.212871932
YLDs (Years Lived with Disability)	Global	Both	50-54 years	Prostate cancer	Smoking	Number	2010	1693.226967	2861.1266	721.084891 8

YLDs (Years Lived with Disability)	Global	Both	50-54 years	Prostate cancer	Smoking	Rate	2010	0.495438523	0.837166171	0.210989572
YLDs (Years Lived with Disability)	Global	Both	50-54 years	Prostate cancer	Smoking	Number	2011	1676.560854	2832.671135	714.134412 3
YLDs (Years Lived with Disability)	Global	Both	50-54 years	Prostate cancer	Smoking	Rate	2011	0.482536272	0.815280022	0.20553728
YLDs (Years Lived with Disability)	Global	Both	50-54 years	Prostate cancer	Smoking	Number	2012	1686.46683	2861.432899	703.041280 7
YLDs (Years Lived with Disability)	Global	Both	50-54 years	Prostate cancer	Smoking	Rate	2012	0.473332805	0.803105069	0.197319328
YLDs (Years Lived with Disability)	Global	Both	50-54 years	Prostate cancer	Smoking	Number	2013	1633.37601	2778.582524	688.438460 3
YLDs (Years Lived with Disability)	Global	Both	50-54 years	Prostate cancer	Smoking	Rate	2013	0.44259066	0.75290359 6	0.186543962
YLDs (Years Lived with Disability)	Global	Both	50-54 years	Prostate cancer	Smoking	Number	2014	1626.025604	2769.44204	689.977089 6
YLDs (Years Lived with Disability)	Global	Both	50-54 years	Prostate cancer	Smoking	Rate	2014	0.423830268	0.721866469	0.179845369
YLDs (Years Lived with Disability)	Global	Both	50-54 years	Prostate cancer	Smoking	Number	2015	1611.53965	2793.112341	681.136272 2
YLDs (Years Lived with Disability)	Global	Both	50-54 years	Prostate cancer	Smoking	Rate	2015	0.404867599	0.701714467	0.171122074

YLDs (Years Lived with Disability)	Global	Both	50-54 years	Prostate cancer	Smoking	Number	2016	1618.487121	2796.063683	683.522032 9
YLDs (Years Lived with Disability)	Global	Both	50-54 years	Prostate cancer	Smoking	Rate	2016	0.394362997	0.681293066	0.166548003
YLDs (Years Lived with Disability)	Global	Both	50-54 years	Prostate cancer	Smoking	Number	2017	1616.778573	2775.760905	690.780266 1
YLDs (Years Lived with Disability)	Global	Both	50-54 years	Prostate cancer	Smoking	Rate	2017	0.384068843	0.659387313	0.164096174
YLDs (Years Lived with Disability)	Global	Both	50-54 years	Prostate cancer	Smoking	Number	2018	1606.189961	2729.365701	678.345804 2
YLDs (Years Lived with Disability)	Global	Both	50-54 years	Prostate cancer	Smoking	Rate	2018	0.374682517	0.636690326	0.158240506
YLDs (Years Lived with Disability)	Global	Both	50-54 years	Prostate cancer	Smoking	Number	2019	1602.369161	2795.77115	663.043898 8
YLDs (Years Lived with Disability)	Global	Both	50-54 years	Prostate cancer	Smoking	Rate	2019	0.368658611	0.643225752	0.152547146
YLDs (Years Lived with Disability)	Global	Both	50-54 years	Prostate cancer	Smoking	Number	2020	1579.34571	2700.960079	655.026370 3
YLDs (Years Lived with Disability)	Global	Both	50-54 years	Prostate cancer	Smoking	Rate	2020	0.35922498	0.614338156	0.148986909
YLDs (Years Lived with Disability)	Global	Both	50-54 years	Prostate cancer	Smoking	Number	2021	1589.71244	2761.326977	666.080612 5

YLDs (Years Lived with Disability)	Global	Both	50-54 years	Prostate cancer	Smoking	Rate	2021	0.357300589	0.62063033	0.149706947
YLDs (Years Lived with Disability)	Global	Both	55-59 years	Prostate cancer	Smoking	Number	1990	1735.960027	2868.423894	745.3065733
YLDs (Years Lived with Disability)	Global	Both	55-59 years	Prostate cancer	Smoking	Rate	1990	0.937343635	1.548825341	0.402433444
YLDs (Years Lived with Disability)	Global	Both	55-59 years	Prostate cancer	Smoking	Number	1991	1756.430923	2945.575689	758.4882238
YLDs (Years Lived with Disability)	Global	Both	55-59 years	Prostate cancer	Smoking	Rate	1991	0.935062194	1.568121144	0.403792517
YLDs (Years Lived with Disability)	Global	Both	55-59 years	Prostate cancer	Smoking	Number	1992	1821.467672	3071.935291	777.7472462
YLDs (Years Lived with Disability)	Global	Both	55-59 years	Prostate cancer	Smoking	Rate	1992	0.953129434	1.607468521	0.406976091
YLDs (Years Lived with Disability)	Global	Both	55-59 years	Prostate cancer	Smoking	Number	1993	1915.664121	3212.980701	816.2530504
YLDs (Years Lived with Disability)	Global	Both	55-59 years	Prostate cancer	Smoking	Rate	1993	0.986204956	1.654077799	0.420216047
YLDs (Years Lived with Disability)	Global	Both	55-59 years	Prostate cancer	Smoking	Number	1994	2018.199742	3382.074926	873.371401
YLDs (Years Lived with Disability)	Global	Both	55-59 years	Prostate cancer	Smoking	Rate	1994	1.024447265	1.716756441	0.443327251

YLDs (Years Lived with Disability)	Global	Both	55-59 years	Prostate cancer	Smoking	Number	1995	2122.395683	3569.145598	933.411453 8
YLDs (Years Lived with Disability)	Global	Both	55-59 years	Prostate cancer	Smoking	Rate	1995	1.063613293	1.788634765	0.467768022
YLDs (Years Lived with Disability)	Global	Both	55-59 years	Prostate cancer	Smoking	Number	1996	2219.921262	3723.99242	963.906422 7
YLDs (Years Lived with Disability)	Global	Both	55-59 years	Prostate cancer	Smoking	Rate	1996	1.102541187	1.849549843	0.478731633
YLDs (Years Lived with Disability)	Global	Both	55-59 years	Prostate cancer	Smoking	Number	1997	2251.274562	3755.82174	976.669208 2
YLDs (Years Lived with Disability)	Global	Both	55-59 years	Prostate cancer	Smoking	Rate	1997	1.112229856	1.855543141	0.482518068
YLDs (Years Lived with Disability)	Global	Both	55-59 years	Prostate cancer	Smoking	Number	1998	2296.003052	3818.704306	996.954332 2
YLDs (Years Lived with Disability)	Global	Both	55-59 years	Prostate cancer	Smoking	Rate	1998	1.129942702	1.879316779	0.49063579
YLDs (Years Lived with Disability)	Global	Both	55-59 years	Prostate cancer	Smoking	Number	1999	2320.424064	3874.857771	989.495291 7
YLDs (Years Lived with Disability)	Global	Both	55-59 years	Prostate cancer	Smoking	Rate	1999	1.136543399	1.897904822	0.484654663
YLDs (Years Lived with Disability)	Global	Both	55-59 years	Prostate cancer	Smoking	Number	2000	2317.220389	3913.927228	983.816704 5

YLDs (Years Lived with Disability)	Global	Both	55-59 years	Prostate cancer	Smoking	Rate	2000	1.130089207	1.908789918	0.479799265
YLDs (Years Lived with Disability)	Global	Both	55-59 years	Prostate cancer	Smoking	Number	2001	2396.879191	4066.288899	1021.892845
YLDs (Years Lived with Disability)	Global	Both	55-59 years	Prostate cancer	Smoking	Rate	2001	1.151168846	1.952949952	0.490792866
YLDs (Years Lived with Disability)	Global	Both	55-59 years	Prostate cancer	Smoking	Number	2002	2546.736309	4326.149253	1084.175439
YLDs (Years Lived with Disability)	Global	Both	55-59 years	Prostate cancer	Smoking	Rate	2002	1.17666321	1.998801622	0.500919293
YLDs (Years Lived with Disability)	Global	Both	55-59 years	Prostate cancer	Smoking	Number	2003	2664.834129	4508.102381	1136.973058
YLDs (Years Lived with Disability)	Global	Both	55-59 years	Prostate cancer	Smoking	Rate	2003	1.181977216	1.999551958	0.504300149
YLDs (Years Lived with Disability)	Global	Both	55-59 years	Prostate cancer	Smoking	Number	2004	2827.829448	4823.04148	1213.919527
YLDs (Years Lived with Disability)	Global	Both	55-59 years	Prostate cancer	Smoking	Rate	2004	1.187598341	2.025523874	0.509807555
YLDs (Years Lived with Disability)	Global	Both	55-59 years	Prostate cancer	Smoking	Number	2005	3083.963406	5217.217436	1335.64566
YLDs (Years Lived with Disability)	Global	Both	55-59 years	Prostate cancer	Smoking	Rate	2005	1.240271547	2.098198159	0.53715401

YLDs (Years Lived with Disability)	Global	Both	55-59 years	Prostate cancer	Smoking	Number	2006	3289.726685	5599.322165	1424.527426
YLDs (Years Lived with Disability)	Global	Both	55-59 years	Prostate cancer	Smoking	Rate	2006	1.257654708	2.140607582	0.544593456
YLDs (Years Lived with Disability)	Global	Both	55-59 years	Prostate cancer	Smoking	Number	2007	3327.235994	5631.566643	1442.988021
YLDs (Years Lived with Disability)	Global	Both	55-59 years	Prostate cancer	Smoking	Rate	2007	1.22794788	2.078382877	0.532548363
YLDs (Years Lived with Disability)	Global	Both	55-59 years	Prostate cancer	Smoking	Number	2008	3410.973346	5763.816281	1497.725636
YLDs (Years Lived with Disability)	Global	Both	55-59 years	Prostate cancer	Smoking	Rate	2008	1.212182258	2.048329062	0.532257587
YLDs (Years Lived with Disability)	Global	Both	55-59 years	Prostate cancer	Smoking	Number	2009	3459.627054	5818.224972	1503.22272
YLDs (Years Lived with Disability)	Global	Both	55-59 years	Prostate cancer	Smoking	Rate	2009	1.193013059	2.006348738	0.518369266
YLDs (Years Lived with Disability)	Global	Both	55-59 years	Prostate cancer	Smoking	Number	2010	3539.054114	5941.114969	1532.857629
YLDs (Years Lived with Disability)	Global	Both	55-59 years	Prostate cancer	Smoking	Rate	2010	1.171376729	1.966424812	0.50735414
YLDs (Years Lived with Disability)	Global	Both	55-59 years	Prostate cancer	Smoking	Number	2011	3543.93454	5931.407673	1540.703679

YLDs (Years Lived with Disability)	Global	Both	55-59 years	Prostate cancer	Smoking	Rate	2011	1.142743149	1.912584841	0.496800534
YLDs (Years Lived with Disability)	Global	Both	55-59 years	Prostate cancer	Smoking	Number	2012	3572.250906	6006.377977	1549.085661
YLDs (Years Lived with Disability)	Global	Both	55-59 years	Prostate cancer	Smoking	Rate	2012	1.127149719	1.895188055	0.488781867
YLDs (Years Lived with Disability)	Global	Both	55-59 years	Prostate cancer	Smoking	Number	2013	3534.580821	6020.150706	1531.831624
YLDs (Years Lived with Disability)	Global	Both	55-59 years	Prostate cancer	Smoking	Rate	2013	1.098189255	1.870452298	0.475937916
YLDs (Years Lived with Disability)	Global	Both	55-59 years	Prostate cancer	Smoking	Number	2014	3555.46985	6010.996862	1542.835728
YLDs (Years Lived with Disability)	Global	Both	55-59 years	Prostate cancer	Smoking	Rate	2014	1.09229417	1.846669247	0.473982495
YLDs (Years Lived with Disability)	Global	Both	55-59 years	Prostate cancer	Smoking	Number	2015	3575.069315	6091.174552	1536.548691
YLDs (Years Lived with Disability)	Global	Both	55-59 years	Prostate cancer	Smoking	Rate	2015	1.085960364	1.850250597	0.466740874
YLDs (Years Lived with Disability)	Global	Both	55-59 years	Prostate cancer	Smoking	Number	2016	3614.467822	6085.178574	1563.307904
YLDs (Years Lived with Disability)	Global	Both	55-59 years	Prostate cancer	Smoking	Rate	2016	1.079541755	1.817474848	0.466916913

YLDs (Years Lived with Disability)	Global	Both	55-59 years	Prostate cancer	Smoking	Number	2017	3599.092715	6121.263369	1583.267726
YLDs (Years Lived with Disability)	Global	Both	55-59 years	Prostate cancer	Smoking	Rate	2017	1.047863533	1.782184891	0.460962983
YLDs (Years Lived with Disability)	Global	Both	55-59 years	Prostate cancer	Smoking	Number	2018	3646.400206	6217.314445	1615.379238
YLDs (Years Lived with Disability)	Global	Both	55-59 years	Prostate cancer	Smoking	Rate	2018	1.024489787	1.746811868	0.453855704
YLDs (Years Lived with Disability)	Global	Both	55-59 years	Prostate cancer	Smoking	Number	2019	3685.775324	6327.85222	1604.086477
YLDs (Years Lived with Disability)	Global	Both	55-59 years	Prostate cancer	Smoking	Rate	2019	0.995638129	1.709342104	0.433311724
YLDs (Years Lived with Disability)	Global	Both	55-59 years	Prostate cancer	Smoking	Number	2020	3646.007742	6296.524876	1597.317111
YLDs (Years Lived with Disability)	Global	Both	55-59 years	Prostate cancer	Smoking	Rate	2020	0.949214017	1.639258635	0.415850952
YLDs (Years Lived with Disability)	Global	Both	55-59 years	Prostate cancer	Smoking	Number	2021	3664.006756	6281.052718	1580.227256
YLDs (Years Lived with Disability)	Global	Both	55-59 years	Prostate cancer	Smoking	Rate	2021	0.925890187	1.587214616	0.399321564
YLDs (Years Lived with Disability)	Global	Both	60-64 years	Prostate cancer	Smoking	Number	1990	3511.318966	5815.681088	1490.693106

YLDs (Years Lived with Disability)	Global	Both	60-64 years	Prostate cancer	Smoking	Rate	1990	2.186249705	3.621012841	0.9281490 5
YLDs (Years Lived with Disability)	Global	Both	60-64 years	Prostate cancer	Smoking	Number	1991	3611.991177	5950.068526	1525.081721
YLDs (Years Lived with Disability)	Global	Both	60-64 years	Prostate cancer	Smoking	Rate	1991	2.203578471	3.629976449	0.930411256
YLDs (Years Lived with Disability)	Global	Both	60-64 years	Prostate cancer	Smoking	Number	1992	3708.62413	6114.766406	1582.028781
YLDs (Years Lived with Disability)	Global	Both	60-64 years	Prostate cancer	Smoking	Rate	1992	2.230107268	3.67699301	0.951321503
YLDs (Years Lived with Disability)	Global	Both	60-64 years	Prostate cancer	Smoking	Number	1993	3756.45222	6197.358026	1597.14305
YLDs (Years Lived with Disability)	Global	Both	60-64 years	Prostate cancer	Smoking	Rate	1993	2.232583789	3.683294836	0.949234936
YLDs (Years Lived with Disability)	Global	Both	60-64 years	Prostate cancer	Smoking	Number	1994	3821.307274	6296.969809	1662.209855
YLDs (Years Lived with Disability)	Global	Both	60-64 years	Prostate cancer	Smoking	Rate	1994	2.248958184	3.705962586	0.978262199
YLDs (Years Lived with Disability)	Global	Both	60-64 years	Prostate cancer	Smoking	Number	1995	3882.919875	6430.614723	1696.527
YLDs (Years Lived with Disability)	Global	Both	60-64 years	Prostate cancer	Smoking	Rate	1995	2.260261772	3.743284203	0.987554533

YLDs (Years Lived with Disability)	Global	Both	60-64 years	Prostate cancer	Smoking	Number	1996	3940.305919	6534.642092	1719.08131
YLDs (Years Lived with Disability)	Global	Both	60-64 years	Prostate cancer	Smoking	Rate	1996	2.260643501	3.749073417	0.986276211
YLDs (Years Lived with Disability)	Global	Both	60-64 years	Prostate cancer	Smoking	Number	1997	3943.259893	6504.424284	1715.512111
YLDs (Years Lived with Disability)	Global	Both	60-64 years	Prostate cancer	Smoking	Rate	1997	2.221457436	3.664303669	0.966443308
YLDs (Years Lived with Disability)	Global	Both	60-64 years	Prostate cancer	Smoking	Number	1998	4043.977063	6681.661273	1760.785052
YLDs (Years Lived with Disability)	Global	Both	60-64 years	Prostate cancer	Smoking	Rate	1998	2.238192076	3.698052951	0.974529551
YLDs (Years Lived with Disability)	Global	Both	60-64 years	Prostate cancer	Smoking	Number	1999	4112.114154	6813.004438	1785.537465
YLDs (Years Lived with Disability)	Global	Both	60-64 years	Prostate cancer	Smoking	Rate	1999	2.239985156	3.711236663	0.972632876
YLDs (Years Lived with Disability)	Global	Both	60-64 years	Prostate cancer	Smoking	Number	2000	4201.034007	6928.027137	1835.838328
YLDs (Years Lived with Disability)	Global	Both	60-64 years	Prostate cancer	Smoking	Rate	2000	2.254607174	3.718127408	0.985256072
YLDs (Years Lived with Disability)	Global	Both	60-64 years	Prostate cancer	Smoking	Number	2001	4312.338204	7074.203574	1890.722863

YLDs (Years Lived with Disability)	Global	Both	60-64 years	Prostate cancer	Smoking	Rate	2001	2.288595448	3.754341458	1.003423093
YLDs (Years Lived with Disability)	Global	Both	60-64 years	Prostate cancer	Smoking	Number	2002	4462.827203	7378.127605	1942.421906
YLDs (Years Lived with Disability)	Global	Both	60-64 years	Prostate cancer	Smoking	Rate	2002	2.35073723	3.886334482	1.02314593
YLDs (Years Lived with Disability)	Global	Both	60-64 years	Prostate cancer	Smoking	Number	2003	4539.227945	7628.422184	1993.04402
YLDs (Years Lived with Disability)	Global	Both	60-64 years	Prostate cancer	Smoking	Rate	2003	2.375008087	3.991331697	1.042797525
YLDs (Years Lived with Disability)	Global	Both	60-64 years	Prostate cancer	Smoking	Number	2004	4583.635939	7690.074367	2007.284499
YLDs (Years Lived with Disability)	Global	Both	60-64 years	Prostate cancer	Smoking	Rate	2004	2.378689842	3.990784177	1.041685533
YLDs (Years Lived with Disability)	Global	Both	60-64 years	Prostate cancer	Smoking	Number	2005	4659.893358	7821.06444	2029.453891
YLDs (Years Lived with Disability)	Global	Both	60-64 years	Prostate cancer	Smoking	Rate	2005	2.398759748	4.02602659	1.044696076
YLDs (Years Lived with Disability)	Global	Both	60-64 years	Prostate cancer	Smoking	Number	2006	4710.664565	7947.463272	2033.755427
YLDs (Years Lived with Disability)	Global	Both	60-64 years	Prostate cancer	Smoking	Rate	2006	2.37978352	4.014983843	1.027434151

YLDs (Years Lived with Disability)	Global	Both	60-64 years	Prostate cancer	Smoking	Number	2007	4906.025553	8337.080172	2097.340162
YLDs (Years Lived with Disability)	Global	Both	60-64 years	Prostate cancer	Smoking	Rate	2007	2.375576211	4.036947853	1.015565724
YLDs (Years Lived with Disability)	Global	Both	60-64 years	Prostate cancer	Smoking	Number	2008	5118.528549	8683.801901	2173.947246
YLDs (Years Lived with Disability)	Global	Both	60-64 years	Prostate cancer	Smoking	Rate	2008	2.37293775	4.025790058	1.00783681
YLDs (Years Lived with Disability)	Global	Both	60-64 years	Prostate cancer	Smoking	Number	2009	5326.738356	9089.301908	2257.008183
YLDs (Years Lived with Disability)	Global	Both	60-64 years	Prostate cancer	Smoking	Rate	2009	2.333610365	3.981965647	0.98878102
YLDs (Years Lived with Disability)	Global	Both	60-64 years	Prostate cancer	Smoking	Number	2010	5588.664031	9541.57953	2394.866304
YLDs (Years Lived with Disability)	Global	Both	60-64 years	Prostate cancer	Smoking	Rate	2010	2.342108778	3.998704709	1.00364548
YLDs (Years Lived with Disability)	Global	Both	60-64 years	Prostate cancer	Smoking	Number	2011	5804.430127	9919.239002	2498.000894
YLDs (Years Lived with Disability)	Global	Both	60-64 years	Prostate cancer	Smoking	Rate	2011	2.309903393	3.947413153	0.994092549
YLDs (Years Lived with Disability)	Global	Both	60-64 years	Prostate cancer	Smoking	Number	2012	5785.796209	9796.757911	2472.323699

YLDs (Years Lived with Disability)	Global	Both	60-64 years	Prostate cancer	Smoking	Rate	2012	2.221447962	3.76145082	0.949245055
YLDs (Years Lived with Disability)	Global	Both	60-64 years	Prostate cancer	Smoking	Number	2013	5768.148114	9843.963786	2474.248983
YLDs (Years Lived with Disability)	Global	Both	60-64 years	Prostate cancer	Smoking	Rate	2013	2.131827846	3.638193004	0.914448238
YLDs (Years Lived with Disability)	Global	Both	60-64 years	Prostate cancer	Smoking	Number	2014	5848.046685	9976.194155	2487.361795
YLDs (Years Lived with Disability)	Global	Both	60-64 years	Prostate cancer	Smoking	Rate	2014	2.096810145	3.576952483	0.891840597
YLDs (Years Lived with Disability)	Global	Both	60-64 years	Prostate cancer	Smoking	Number	2015	5961.171965	10176.00549	2556.377884
YLDs (Years Lived with Disability)	Global	Both	60-64 years	Prostate cancer	Smoking	Rate	2015	2.05178931	3.502502426	0.879885506
YLDs (Years Lived with Disability)	Global	Both	60-64 years	Prostate cancer	Smoking	Number	2016	6075.660946	10363.98472	2613.428743
YLDs (Years Lived with Disability)	Global	Both	60-64 years	Prostate cancer	Smoking	Rate	2016	2.03807928	3.476596654	0.876674163
YLDs (Years Lived with Disability)	Global	Both	60-64 years	Prostate cancer	Smoking	Number	2017	6187.650243	10488.46343	2679.623062
YLDs (Years Lived with Disability)	Global	Both	60-64 years	Prostate cancer	Smoking	Rate	2017	2.032162565	3.444645691	0.880048073

YLDs (Years Lived with Disability)	Global	Both	60-64 years	Prostate cancer	Smoking	Number	2018	6313.484711	10896.30727	2734.668374
YLDs (Years Lived with Disability)	Global	Both	60-64 years	Prostate cancer	Smoking	Rate	2018	2.043070304	3.526091032	0.88495023
YLDs (Years Lived with Disability)	Global	Both	60-64 years	Prostate cancer	Smoking	Number	2019	6433.241422	11000.21156	2805.446698
YLDs (Years Lived with Disability)	Global	Both	60-64 years	Prostate cancer	Smoking	Rate	2019	2.059900032	3.522227416	0.898293748
YLDs (Years Lived with Disability)	Global	Both	60-64 years	Prostate cancer	Smoking	Number	2020	6415.725007	10986.65838	2786.393811
YLDs (Years Lived with Disability)	Global	Both	60-64 years	Prostate cancer	Smoking	Rate	2020	2.033886136	3.48294416	0.883330828
YLDs (Years Lived with Disability)	Global	Both	60-64 years	Prostate cancer	Smoking	Number	2021	6476.995564	11098.54448	2797.261961
YLDs (Years Lived with Disability)	Global	Both	60-64 years	Prostate cancer	Smoking	Rate	2021	2.023758478	3.467776574	0.874013661
YLDs (Years Lived with Disability)	Global	Both	65-69 years	Prostate cancer	Smoking	Number	1990	4523.976072	7549.427661	1949.701652
YLDs (Years Lived with Disability)	Global	Both	65-69 years	Prostate cancer	Smoking	Rate	1990	3.659894776	6.107483865	1.577307832
YLDs (Years Lived with Disability)	Global	Both	65-69 years	Prostate cancer	Smoking	Number	1991	4667.023608	7783.809814	2000.881352

YLDs (Years Lived with Disability)	Global	Both	65-69 years	Prostate cancer	Smoking	Rate	1991	3.662436143	6.108327017	1.570187082
YLDs (Years Lived with Disability)	Global	Both	65-69 years	Prostate cancer	Smoking	Number	1992	4848.163424	8098.294492	2100.951198
YLDs (Years Lived with Disability)	Global	Both	65-69 years	Prostate cancer	Smoking	Rate	1992	3.692821096	6.168429182	1.6002837
YLDs (Years Lived with Disability)	Global	Both	65-69 years	Prostate cancer	Smoking	Number	1993	5046.166319	8426.418655	2192.837809
YLDs (Years Lived with Disability)	Global	Both	65-69 years	Prostate cancer	Smoking	Rate	1993	3.733976584	6.235238388	1.622618937
YLDs (Years Lived with Disability)	Global	Both	65-69 years	Prostate cancer	Smoking	Number	1994	5214.532264	8674.225155	2293.953806
YLDs (Years Lived with Disability)	Global	Both	65-69 years	Prostate cancer	Smoking	Rate	1994	3.755666173	6.247443174	1.652175933
YLDs (Years Lived with Disability)	Global	Both	65-69 years	Prostate cancer	Smoking	Number	1995	5368.310743	8991.209565	2374.485555
YLDs (Years Lived with Disability)	Global	Both	65-69 years	Prostate cancer	Smoking	Rate	1995	3.780275996	6.331461668	1.672073614
YLDs (Years Lived with Disability)	Global	Both	65-69 years	Prostate cancer	Smoking	Number	1996	5471.044106	9158.014923	2419.958527
YLDs (Years Lived with Disability)	Global	Both	65-69 years	Prostate cancer	Smoking	Rate	1996	3.776678984	6.321806555	1.670504996

YLDs (Years Lived with Disability)	Global	Both	65-69 years	Prostate cancer	Smoking	Number	1997	5427.832062	9091.451932	2382.685391
YLDs (Years Lived with Disability)	Global	Both	65-69 years	Prostate cancer	Smoking	Rate	1997	3.693030979	6.18571342	1.621150187
YLDs (Years Lived with Disability)	Global	Both	65-69 years	Prostate cancer	Smoking	Number	1998	5347.509454	8958.509951	2376.676445
YLDs (Years Lived with Disability)	Global	Both	65-69 years	Prostate cancer	Smoking	Rate	1998	3.593888894	6.02072604	1.597287701
YLDs (Years Lived with Disability)	Global	Both	65-69 years	Prostate cancer	Smoking	Number	1999	5272.130507	8868.308295	2345.711494
YLDs (Years Lived with Disability)	Global	Both	65-69 years	Prostate cancer	Smoking	Rate	1999	3.502987347	5.892413267	1.558572511
YLDs (Years Lived with Disability)	Global	Both	65-69 years	Prostate cancer	Smoking	Number	2000	5203.514486	8802.932675	2283.62347
YLDs (Years Lived with Disability)	Global	Both	65-69 years	Prostate cancer	Smoking	Rate	2000	3.410884467	5.770289755	1.496906724
YLDs (Years Lived with Disability)	Global	Both	65-69 years	Prostate cancer	Smoking	Number	2001	5208.916006	8828.191894	2282.532501
YLDs (Years Lived with Disability)	Global	Both	65-69 years	Prostate cancer	Smoking	Rate	2001	3.355248213	5.686552641	1.470260431
YLDs (Years Lived with Disability)	Global	Both	65-69 years	Prostate cancer	Smoking	Number	2002	5300.602666	8971.770439	2337.898513

YLDs (Years Lived with Disability)	Global	Both	65-69 years	Prostate cancer	Smoking	Rate	2002	3.34270279	5.657840055	1.474341764
YLDs (Years Lived with Disability)	Global	Both	65-69 years	Prostate cancer	Smoking	Number	2003	5403.054726	9147.466128	2369.882586
YLDs (Years Lived with Disability)	Global	Both	65-69 years	Prostate cancer	Smoking	Rate	2003	3.338057891	5.651390378	1.464135692
YLDs (Years Lived with Disability)	Global	Both	65-69 years	Prostate cancer	Smoking	Number	2004	5500.579817	9253.416076	2410.109302
YLDs (Years Lived with Disability)	Global	Both	65-69 years	Prostate cancer	Smoking	Rate	2004	3.335839686	5.611756143	1.461616507
YLDs (Years Lived with Disability)	Global	Both	65-69 years	Prostate cancer	Smoking	Number	2005	5662.012822	9535.314368	2464.851714
YLDs (Years Lived with Disability)	Global	Both	65-69 years	Prostate cancer	Smoking	Rate	2005	3.374108284	5.682287237	1.468855131
YLDs (Years Lived with Disability)	Global	Both	65-69 years	Prostate cancer	Smoking	Number	2006	5766.54388	9718.218397	2508.336244
YLDs (Years Lived with Disability)	Global	Both	65-69 years	Prostate cancer	Smoking	Rate	2006	3.387360458	5.708637514	1.473436981
YLDs (Years Lived with Disability)	Global	Both	65-69 years	Prostate cancer	Smoking	Number	2007	5791.342511	9830.047909	2516.72015
YLDs (Years Lived with Disability)	Global	Both	65-69 years	Prostate cancer	Smoking	Rate	2007	3.364566067	5.710911687	1.462125786

YLDs (Years Lived with Disability)	Global	Both	65-69 years	Prostate cancer	Smoking	Number	2008	5813.531424	9894.935458	2533.445066
YLDs (Years Lived with Disability)	Global	Both	65-69 years	Prostate cancer	Smoking	Rate	2008	3.341919261	5.688121897	1.456355568
YLDs (Years Lived with Disability)	Global	Both	65-69 years	Prostate cancer	Smoking	Number	2009	5789.809629	9889.625014	2530.625
YLDs (Years Lived with Disability)	Global	Both	65-69 years	Prostate cancer	Smoking	Rate	2009	3.288751161	5.6175457	1.437456078
YLDs (Years Lived with Disability)	Global	Both	65-69 years	Prostate cancer	Smoking	Number	2010	5744.060843	9905.376145	2509.827337
YLDs (Years Lived with Disability)	Global	Both	65-69 years	Prostate cancer	Smoking	Rate	2010	3.226273119	5.563563768	1.409697545
YLDs (Years Lived with Disability)	Global	Both	65-69 years	Prostate cancer	Smoking	Number	2011	5697.040458	9843.494438	2495.033357
YLDs (Years Lived with Disability)	Global	Both	65-69 years	Prostate cancer	Smoking	Rate	2011	3.132744517	5.412837324	1.371993428
YLDs (Years Lived with Disability)	Global	Both	65-69 years	Prostate cancer	Smoking	Number	2012	5813.907362	10016.89956	2551.180615
YLDs (Years Lived with Disability)	Global	Both	65-69 years	Prostate cancer	Smoking	Rate	2012	3.056016415	5.265273003	1.341000011
YLDs (Years Lived with Disability)	Global	Both	65-69 years	Prostate cancer	Smoking	Number	2013	5930.083122	10259.92414	2616.11401

YLDs (Years Lived with Disability)	Global	Both	65-69 years	Prostate cancer	Smoking	Rate	2013	2.978271546	5.152851911	1.313893542
YLDs (Years Lived with Disability)	Global	Both	65-69 years	Prostate cancer	Smoking	Number	2014	6227.156858	10776.12166	2743.776298
YLDs (Years Lived with Disability)	Global	Both	65-69 years	Prostate cancer	Smoking	Rate	2014	2.949427438	5.103996838	1.299560824
YLDs (Years Lived with Disability)	Global	Both	65-69 years	Prostate cancer	Smoking	Number	2015	6522.942849	11300.49431	2845.701801
YLDs (Years Lived with Disability)	Global	Both	65-69 years	Prostate cancer	Smoking	Rate	2015	2.951931229	5.113992692	1.287810764
YLDs (Years Lived with Disability)	Global	Both	65-69 years	Prostate cancer	Smoking	Number	2016	6888.213277	11832.60495	3060.377421
YLDs (Years Lived with Disability)	Global	Both	65-69 years	Prostate cancer	Smoking	Rate	2016	2.956100384	5.078003053	1.313371481
YLDs (Years Lived with Disability)	Global	Both	65-69 years	Prostate cancer	Smoking	Number	2017	7047.731836	12021.12708	3064.845166
YLDs (Years Lived with Disability)	Global	Both	65-69 years	Prostate cancer	Smoking	Rate	2017	2.915571624	4.973012285	1.267893814
YLDs (Years Lived with Disability)	Global	Both	65-69 years	Prostate cancer	Smoking	Number	2018	7189.877711	12189.27442	3157.762036
YLDs (Years Lived with Disability)	Global	Both	65-69 years	Prostate cancer	Smoking	Rate	2018	2.861723225	4.851588734	1.256856002

YLDs (Years Lived with Disability)	Global	Both	65-69 years	Prostate cancer	Smoking	Number	2019	7309.171768	12228.34911	3199.909813
YLDs (Years Lived with Disability)	Global	Both	65-69 years	Prostate cancer	Smoking	Rate	2019	2.82165282	4.720665603	1.235302006
YLDs (Years Lived with Disability)	Global	Both	65-69 years	Prostate cancer	Smoking	Number	2020	7394.987989	12423.31795	3243.881353
YLDs (Years Lived with Disability)	Global	Both	65-69 years	Prostate cancer	Smoking	Rate	2020	2.74338204	4.60878468	1.203410453
YLDs (Years Lived with Disability)	Global	Both	65-69 years	Prostate cancer	Smoking	Number	2021	7522.0795	12601.79838	3285.53034
YLDs (Years Lived with Disability)	Global	Both	65-69 years	Prostate cancer	Smoking	Rate	2021	2.726950637	4.568481644	1.191090716
YLDs (Years Lived with Disability)	Global	Both	70-74 years	Prostate cancer	Smoking	Number	1990	3828.56799	6330.968724	1670.013082
YLDs (Years Lived with Disability)	Global	Both	70-74 years	Prostate cancer	Smoking	Rate	1990	4.522225112	7.478008963	1.972584819
YLDs (Years Lived with Disability)	Global	Both	70-74 years	Prostate cancer	Smoking	Number	1991	4047.377836	6702.65743	1779.616306
YLDs (Years Lived with Disability)	Global	Both	70-74 years	Prostate cancer	Smoking	Rate	1991	4.613484079	7.640157304	2.028531021
YLDs (Years Lived with Disability)	Global	Both	70-74 years	Prostate cancer	Smoking	Number	1992	4311.340726	7134.928912	1901.34747

YLDs (Years Lived with Disability)	Global	Both	70-74 years	Prostate cancer	Smoking	Rate	1992	4.716752052	7.805852686	2.0801382 1
YLDs (Years Lived with Disability)	Global	Both	70-74 years	Prostate cancer	Smoking	Number	1993	4593.807696	7637.721998	2014.476909
YLDs (Years Lived with Disability)	Global	Both	70-74 years	Prostate cancer	Smoking	Rate	1993	4.815816139	8.006836006	2.111832069
YLDs (Years Lived with Disability)	Global	Both	70-74 years	Prostate cancer	Smoking	Number	1994	4873.337438	8122.533585	2128.986856
YLDs (Years Lived with Disability)	Global	Both	70-74 years	Prostate cancer	Smoking	Rate	1994	4.912261329	8.18740917	2.145991312
YLDs (Years Lived with Disability)	Global	Both	70-74 years	Prostate cancer	Smoking	Number	1995	5055.070479	8430.42227	2206.988495
YLDs (Years Lived with Disability)	Global	Both	70-74 years	Prostate cancer	Smoking	Rate	1995	4.92248212	8.209302532	2.149101867
YLDs (Years Lived with Disability)	Global	Both	70-74 years	Prostate cancer	Smoking	Number	1996	5151.823706	8548.745726	2258.290949
YLDs (Years Lived with Disability)	Global	Both	70-74 years	Prostate cancer	Smoking	Rate	1996	4.861574992	8.067117746	2.131061044
YLDs (Years Lived with Disability)	Global	Both	70-74 years	Prostate cancer	Smoking	Number	1997	5117.960613	8513.30134	2249.176916
YLDs (Years Lived with Disability)	Global	Both	70-74 years	Prostate cancer	Smoking	Rate	1997	4.680605797	7.785798019	2.056973726

YLDs (Years Lived with Disability)	Global	Both	70-74 years	Prostate cancer	Smoking	Number	1998	5140.967114	8540.517306	2277.840425
YLDs (Years Lived with Disability)	Global	Both	70-74 years	Prostate cancer	Smoking	Rate	1998	4.558813425	7.573404786	2.019901952
YLDs (Years Lived with Disability)	Global	Both	70-74 years	Prostate cancer	Smoking	Number	1999	5149.007079	8539.462639	2287.824889
YLDs (Years Lived with Disability)	Global	Both	70-74 years	Prostate cancer	Smoking	Rate	1999	4.433690223	7.35313264	1.969992794
YLDs (Years Lived with Disability)	Global	Both	70-74 years	Prostate cancer	Smoking	Number	2000	5123.70776	8536.336407	2293.145792
YLDs (Years Lived with Disability)	Global	Both	70-74 years	Prostate cancer	Smoking	Rate	2000	4.301373054	7.166288383	1.925105018
YLDs (Years Lived with Disability)	Global	Both	70-74 years	Prostate cancer	Smoking	Number	2001	5166.363192	8634.542409	2308.735221
YLDs (Years Lived with Disability)	Global	Both	70-74 years	Prostate cancer	Smoking	Rate	2001	4.239057659	7.084736743	1.894342569
YLDs (Years Lived with Disability)	Global	Both	70-74 years	Prostate cancer	Smoking	Number	2002	5169.404723	8656.781141	2300.34026
YLDs (Years Lived with Disability)	Global	Both	70-74 years	Prostate cancer	Smoking	Rate	2002	4.168769625	6.981099026	1.855066322
YLDs (Years Lived with Disability)	Global	Both	70-74 years	Prostate cancer	Smoking	Number	2003	5139.245476	8625.426597	2264.833257

YLDs (Years Lived with Disability)	Global	Both	70-74 years	Prostate cancer	Smoking	Rate	2003	4.081691116	6.850485596	1.798775681
YLDs (Years Lived with Disability)	Global	Both	70-74 years	Prostate cancer	Smoking	Number	2004	5101.531665	8557.759029	2254.86983
YLDs (Years Lived with Disability)	Global	Both	70-74 years	Prostate cancer	Smoking	Rate	2004	3.992175591	6.696827336	1.764536004
YLDs (Years Lived with Disability)	Global	Both	70-74 years	Prostate cancer	Smoking	Number	2005	5135.52586	8685.594229	2246.933352
YLDs (Years Lived with Disability)	Global	Both	70-74 years	Prostate cancer	Smoking	Rate	2005	3.949884763	6.680347299	1.728182869
YLDs (Years Lived with Disability)	Global	Both	70-74 years	Prostate cancer	Smoking	Number	2006	5128.562946	8669.890956	2248.399259
YLDs (Years Lived with Disability)	Global	Both	70-74 years	Prostate cancer	Smoking	Rate	2006	3.860247962	6.525790801	1.692360755
YLDs (Years Lived with Disability)	Global	Both	70-74 years	Prostate cancer	Smoking	Number	2007	5157.983185	8820.903822	2240.010613
YLDs (Years Lived with Disability)	Global	Both	70-74 years	Prostate cancer	Smoking	Rate	2007	3.785215528	6.473270836	1.643844629
YLDs (Years Lived with Disability)	Global	Both	70-74 years	Prostate cancer	Smoking	Number	2008	5201.161889	8857.84583	2240.402884
YLDs (Years Lived with Disability)	Global	Both	70-74 years	Prostate cancer	Smoking	Rate	2008	3.725110799	6.344055014	1.604593196

YLDs (Years Lived with Disability)	Global	Both	70-74 years	Prostate cancer	Smoking	Number	2009	5233.374328	8955.864708	2272.899601
YLDs (Years Lived with Disability)	Global	Both	70-74 years	Prostate cancer	Smoking	Rate	2009	3.666660151	6.274749366	1.592462123
YLDs (Years Lived with Disability)	Global	Both	70-74 years	Prostate cancer	Smoking	Number	2010	5267.469185	9094.292732	2289.862613
YLDs (Years Lived with Disability)	Global	Both	70-74 years	Prostate cancer	Smoking	Rate	2010	3.614616872	6.240640959	1.571338292
YLDs (Years Lived with Disability)	Global	Both	70-74 years	Prostate cancer	Smoking	Number	2011	5251.1727	9101.624052	2277.428736
YLDs (Years Lived with Disability)	Global	Both	70-74 years	Prostate cancer	Smoking	Rate	2011	3.540897719	6.137280506	1.535684061
YLDs (Years Lived with Disability)	Global	Both	70-74 years	Prostate cancer	Smoking	Number	2012	5179.620178	8936.643176	2242.823468
YLDs (Years Lived with Disability)	Global	Both	70-74 years	Prostate cancer	Smoking	Rate	2012	3.445184852	5.944140041	1.491796922
YLDs (Years Lived with Disability)	Global	Both	70-74 years	Prostate cancer	Smoking	Number	2013	5074.429848	8731.742219	2166.671691
YLDs (Years Lived with Disability)	Global	Both	70-74 years	Prostate cancer	Smoking	Rate	2013	3.330364351	5.730670022	1.42199348
YLDs (Years Lived with Disability)	Global	Both	70-74 years	Prostate cancer	Smoking	Number	2014	5120.764403	8881.009744	2180.682663

YLDs (Years Lived with Disability)	Global	Both	70-74 years	Prostate cancer	Smoking	Rate	2014	3.31084215	5.742037532	1.409925454
YLDs (Years Lived with Disability)	Global	Both	70-74 years	Prostate cancer	Smoking	Number	2015	5142.523168	9012.532406	2178.729895
YLDs (Years Lived with Disability)	Global	Both	70-74 years	Prostate cancer	Smoking	Rate	2015	3.278379844	5.745526782	1.388949343
YLDs (Years Lived with Disability)	Global	Both	70-74 years	Prostate cancer	Smoking	Number	2016	5218.850239	9000.773275	2211.151458
YLDs (Years Lived with Disability)	Global	Both	70-74 years	Prostate cancer	Smoking	Rate	2016	3.249006533	5.603450919	1.376557135
YLDs (Years Lived with Disability)	Global	Both	70-74 years	Prostate cancer	Smoking	Number	2017	5482.847579	9533.751333	2300.939684
YLDs (Years Lived with Disability)	Global	Both	70-74 years	Prostate cancer	Smoking	Rate	2017	3.253802769	5.657816679	1.36549554
YLDs (Years Lived with Disability)	Global	Both	70-74 years	Prostate cancer	Smoking	Number	2018	5764.358844	9954.278928	2422.301856
YLDs (Years Lived with Disability)	Global	Both	70-74 years	Prostate cancer	Smoking	Rate	2018	3.26304226	5.634838789	1.371197307
YLDs (Years Lived with Disability)	Global	Both	70-74 years	Prostate cancer	Smoking	Number	2019	6087.990558	10508.77337	2543.302559
YLDs (Years Lived with Disability)	Global	Both	70-74 years	Prostate cancer	Smoking	Rate	2019	3.245724872	5.602601839	1.355925292

YLDs (Years Lived with Disability)	Global	Both	70-74 years	Prostate cancer	Smoking	Number	2020	6361.370024	11012.81753	2697.7273
YLDs (Years Lived with Disability)	Global	Both	70-74 years	Prostate cancer	Smoking	Rate	2020	3.244831712	5.617459669	1.376066957
YLDs (Years Lived with Disability)	Global	Both	70-74 years	Prostate cancer	Smoking	Number	2021	6691.388687	11673.77554	2815.931506
YLDs (Years Lived with Disability)	Global	Both	70-74 years	Prostate cancer	Smoking	Rate	2021	3.250784119	5.671307694	1.368024762
YLDs (Years Lived with Disability)	Global	Both	75-79 years	Prostate cancer	Smoking	Number	1990	3114.666984	5422.0301	1364.082344
YLDs (Years Lived with Disability)	Global	Both	75-79 years	Prostate cancer	Smoking	Rate	1990	5.059931701	8.808358044	2.216019732
YLDs (Years Lived with Disability)	Global	Both	75-79 years	Prostate cancer	Smoking	Number	1991	3175.08768	5509.411718	1397.754313
YLDs (Years Lived with Disability)	Global	Both	75-79 years	Prostate cancer	Smoking	Rate	1991	5.140061614	8.919034225	2.262785792
YLDs (Years Lived with Disability)	Global	Both	75-79 years	Prostate cancer	Smoking	Number	1992	3188.439501	5526.62751	1398.871797
YLDs (Years Lived with Disability)	Global	Both	75-79 years	Prostate cancer	Smoking	Rate	1992	5.163310964	8.949737452	2.265311945
YLDs (Years Lived with Disability)	Global	Both	75-79 years	Prostate cancer	Smoking	Number	1993	3176.771686	5508.210574	1400.256065

YLDs (Years Lived with Disability)	Global	Both	75-79 years	Prostate cancer	Smoking	Rate	1993	5.151903571	8.932895571	2.270853852
YLDs (Years Lived with Disability)	Global	Both	75-79 years	Prostate cancer	Smoking	Number	1994	3183.535067	5553.897819	1399.518563
YLDs (Years Lived with Disability)	Global	Both	75-79 years	Prostate cancer	Smoking	Rate	1994	5.117743196	8.928258108	2.249818661
YLDs (Years Lived with Disability)	Global	Both	75-79 years	Prostate cancer	Smoking	Number	1995	3257.364984	5694.410328	1421.447255
YLDs (Years Lived with Disability)	Global	Both	75-79 years	Prostate cancer	Smoking	Rate	1995	5.105090841	8.924539359	2.227756913
YLDs (Years Lived with Disability)	Global	Both	75-79 years	Prostate cancer	Smoking	Number	1996	3378.813806	5946.435494	1483.953956
YLDs (Years Lived with Disability)	Global	Both	75-79 years	Prostate cancer	Smoking	Rate	1996	5.094062982	8.965133525	2.237280699
YLDs (Years Lived with Disability)	Global	Both	75-79 years	Prostate cancer	Smoking	Number	1997	3475.729536	6115.559211	1536.338272
YLDs (Years Lived with Disability)	Global	Both	75-79 years	Prostate cancer	Smoking	Rate	1997	5.011693756	8.818094044	2.215263543
YLDs (Years Lived with Disability)	Global	Both	75-79 years	Prostate cancer	Smoking	Number	1998	3602.235989	6293.23648	1587.070537
YLDs (Years Lived with Disability)	Global	Both	75-79 years	Prostate cancer	Smoking	Rate	1998	4.959107349	8.663739791	2.184879944

YLDs (Years Lived with Disability)	Global	Both	75-79 years	Prostate cancer	Smoking	Number	1999	3706.757184	6458.755335	1638.900613
YLDs (Years Lived with Disability)	Global	Both	75-79 years	Prostate cancer	Smoking	Rate	1999	4.888543301	8.517931863	2.16141393
YLDs (Years Lived with Disability)	Global	Both	75-79 years	Prostate cancer	Smoking	Number	2000	3735.921538	6479.847466	1655.517309
YLDs (Years Lived with Disability)	Global	Both	75-79 years	Prostate cancer	Smoking	Rate	2000	4.742190256	8.22519135	2.10143012
YLDs (Years Lived with Disability)	Global	Both	75-79 years	Prostate cancer	Smoking	Number	2001	3770.627699	6535.28084	1677.558887
YLDs (Years Lived with Disability)	Global	Both	75-79 years	Prostate cancer	Smoking	Rate	2001	4.622480908	8.011719353	2.05654987
YLDs (Years Lived with Disability)	Global	Both	75-79 years	Prostate cancer	Smoking	Number	2002	3798.07116	6599.837343	1715.307129
YLDs (Years Lived with Disability)	Global	Both	75-79 years	Prostate cancer	Smoking	Rate	2002	4.498277793	7.816573336	2.03153854
YLDs (Years Lived with Disability)	Global	Both	75-79 years	Prostate cancer	Smoking	Number	2003	3854.990727	6735.822855	1741.987261
YLDs (Years Lived with Disability)	Global	Both	75-79 years	Prostate cancer	Smoking	Rate	2003	4.413312986	7.711378986	1.99428106
YLDs (Years Lived with Disability)	Global	Both	75-79 years	Prostate cancer	Smoking	Number	2004	3894.59086	6760.206054	1757.308001

YLDs (Years Lived with Disability)	Global	Both	75-79 years	Prostate cancer	Smoking	Rate	2004	4.316082387	7.491828366	1.947492403
YLDs (Years Lived with Disability)	Global	Both	75-79 years	Prostate cancer	Smoking	Number	2005	3946.186231	6860.3688188	1772.783121
YLDs (Years Lived with Disability)	Global	Both	75-79 years	Prostate cancer	Smoking	Rate	2005	4.250375565	7.389196123	1.909437016
YLDs (Years Lived with Disability)	Global	Both	75-79 years	Prostate cancer	Smoking	Number	2006	3980.750134	6919.692006	1780.857024
YLDs (Years Lived with Disability)	Global	Both	75-79 years	Prostate cancer	Smoking	Rate	2006	4.173471574	7.254697461	1.867074275
YLDs (Years Lived with Disability)	Global	Both	75-79 years	Prostate cancer	Smoking	Number	2007	3968.361963	6899.167086	1774.717142
YLDs (Years Lived with Disability)	Global	Both	75-79 years	Prostate cancer	Smoking	Rate	2007	4.068121136	7.072602677	1.819331095
YLDs (Years Lived with Disability)	Global	Both	75-79 years	Prostate cancer	Smoking	Number	2008	3922.125434	6778.50216	1735.496962
YLDs (Years Lived with Disability)	Global	Both	75-79 years	Prostate cancer	Smoking	Rate	2008	3.938235225	6.806344271	1.742625365
YLDs (Years Lived with Disability)	Global	Both	75-79 years	Prostate cancer	Smoking	Number	2009	3865.402667	6746.580846	1701.296489
YLDs (Years Lived with Disability)	Global	Both	75-79 years	Prostate cancer	Smoking	Rate	2009	3.802242111	6.63634193	1.673497358

YLDs (Years Lived with Disability)	Global	Both	75-79 years	Prostate cancer	Smoking	Number	2010	3822.868406	6654.905688	1649.778499
YLDs (Years Lived with Disability)	Global	Both	75-79 years	Prostate cancer	Smoking	Rate	2010	3.675070114	6.397616242	1.585995387
YLDs (Years Lived with Disability)	Global	Both	75-79 years	Prostate cancer	Smoking	Number	2011	3786.996043	6629.150928	1632.737771
YLDs (Years Lived with Disability)	Global	Both	75-79 years	Prostate cancer	Smoking	Rate	2011	3.546486114	6.208137385	1.529043539
YLDs (Years Lived with Disability)	Global	Both	75-79 years	Prostate cancer	Smoking	Number	2012	3753.349668	6546.750509	1604.463694
YLDs (Years Lived with Disability)	Global	Both	75-79 years	Prostate cancer	Smoking	Rate	2012	3.413927325	5.954715768	1.459369078
YLDs (Years Lived with Disability)	Global	Both	75-79 years	Prostate cancer	Smoking	Number	2013	3761.46275	6573.553118	1615.326822
YLDs (Years Lived with Disability)	Global	Both	75-79 years	Prostate cancer	Smoking	Rate	2013	3.326816594	5.81396309	1.428671885
YLDs (Years Lived with Disability)	Global	Both	75-79 years	Prostate cancer	Smoking	Number	2014	3839.524252	6671.22752	1649.277498
YLDs (Years Lived with Disability)	Global	Both	75-79 years	Prostate cancer	Smoking	Rate	2014	3.311258179	5.753357771	1.422359451
YLDs (Years Lived with Disability)	Global	Both	75-79 years	Prostate cancer	Smoking	Number	2015	3915.693694	6773.733989	1686.103253

YLDs (Years Lived with Disability)	Global	Both	75-79 years	Prostate cancer	Smoking	Rate	2015	3.296591874	5.702753628	1.419517132
YLDs (Years Lived with Disability)	Global	Both	75-79 years	Prostate cancer	Smoking	Number	2016	4008.360174	6930.823022	1735.260804
YLDs (Years Lived with Disability)	Global	Both	75-79 years	Prostate cancer	Smoking	Rate	2016	3.303941767	5.712818875	1.43031075
YLDs (Years Lived with Disability)	Global	Both	75-79 years	Prostate cancer	Smoking	Number	2017	4060.46362	7076.89998	1757.560442
YLDs (Years Lived with Disability)	Global	Both	75-79 years	Prostate cancer	Smoking	Rate	2017	3.290052763	5.734166467	1.424090234
YLDs (Years Lived with Disability)	Global	Both	75-79 years	Prostate cancer	Smoking	Number	2018	4121.664849	7155.528492	1784.853669
YLDs (Years Lived with Disability)	Global	Both	75-79 years	Prostate cancer	Smoking	Rate	2018	3.284904203	5.702847388	1.42250123
YLDs (Years Lived with Disability)	Global	Both	75-79 years	Prostate cancer	Smoking	Number	2019	4169.937331	7202.635695	1804.228397
YLDs (Years Lived with Disability)	Global	Both	75-79 years	Prostate cancer	Smoking	Rate	2019	3.266189909	5.641613807	1.413199316
YLDs (Years Lived with Disability)	Global	Both	75-79 years	Prostate cancer	Smoking	Number	2020	4186.854659	7308.642902	1811.386449
YLDs (Years Lived with Disability)	Global	Both	75-79 years	Prostate cancer	Smoking	Rate	2020	3.236581382	5.649830115	1.400263476

YLDs (Years Lived with Disability)	Global	Both	75-79 years	Prostate cancer	Smoking	Number	2021	4231.89011	7420.917059	1813.97128
YLDs (Years Lived with Disability)	Global	Both	75-79 years	Prostate cancer	Smoking	Rate	2021	3.208787365	5.62683441	1.375425158
YLDs (Years Lived with Disability)	Global	Both	80-84 years	Prostate cancer	Smoking	Number	1990	1359.814252	2399.407708	584.5233972
YLDs (Years Lived with Disability)	Global	Both	80-84 years	Prostate cancer	Smoking	Rate	1990	3.843897136	6.7826002	1.652319653
YLDs (Years Lived with Disability)	Global	Both	80-84 years	Prostate cancer	Smoking	Number	1991	1435.449417	2542.86362	614.5068562
YLDs (Years Lived with Disability)	Global	Both	80-84 years	Prostate cancer	Smoking	Rate	1991	3.93365989	6.968382522	1.683975028
YLDs (Years Lived with Disability)	Global	Both	80-84 years	Prostate cancer	Smoking	Number	1992	1503.052036	2674.610681	647.428414
YLDs (Years Lived with Disability)	Global	Both	80-84 years	Prostate cancer	Smoking	Rate	1992	4.00738977	7.130962353	1.726153141
YLDs (Years Lived with Disability)	Global	Both	80-84 years	Prostate cancer	Smoking	Number	1993	1573.474717	2800.702826	680.8645143
YLDs (Years Lived with Disability)	Global	Both	80-84 years	Prostate cancer	Smoking	Rate	1993	4.079453143	7.261213557	1.765236424
YLDs (Years Lived with Disability)	Global	Both	80-84 years	Prostate cancer	Smoking	Number	1994	1643.082896	2935.073651	709.5544481

YLDs (Years Lived with Disability)	Global	Both	80-84 years	Prostate cancer	Smoking	Rate	1994	4.165440062	7.440813487	1.798817657
YLDs (Years Lived with Disability)	Global	Both	80-84 years	Prostate cancer	Smoking	Number	1995	1683.526845	3002.947683	731.246948 5
YLDs (Years Lived with Disability)	Global	Both	80-84 years	Prostate cancer	Smoking	Rate	1995	4.200385978	7.492330389	1.824455273
YLDs (Years Lived with Disability)	Global	Both	80-84 years	Prostate cancer	Smoking	Number	1996	1688.589836	3010.997819	727.4958836
YLDs (Years Lived with Disability)	Global	Both	80-84 years	Prostate cancer	Smoking	Rate	1996	4.193535341	7.477674858	1.80670263
YLDs (Years Lived with Disability)	Global	Both	80-84 years	Prostate cancer	Smoking	Number	1997	1643.088475	2936.93099	709.4815658
YLDs (Years Lived with Disability)	Global	Both	80-84 years	Prostate cancer	Smoking	Rate	1997	4.072467652	7.279313702	1.758481524
YLDs (Years Lived with Disability)	Global	Both	80-84 years	Prostate cancer	Smoking	Number	1998	1596.654246	2865.763184	689.374037 1
YLDs (Years Lived with Disability)	Global	Both	80-84 years	Prostate cancer	Smoking	Rate	1998	3.946932326	7.08417203	1.704133928
YLDs (Years Lived with Disability)	Global	Both	80-84 years	Prostate cancer	Smoking	Number	1999	1551.198909	2794.129543	672.250484 3
YLDs (Years Lived with Disability)	Global	Both	80-84 years	Prostate cancer	Smoking	Rate	1999	3.779597271	6.808078795	1.637982132

YLDs (Years Lived with Disability)	Global	Both	80-84 years	Prostate cancer	Smoking	Number	2000	1553.608993	2801.744156	671.00136 61
YLDs (Years Lived with Disability)	Global	Both	80-84 years	Prostate cancer	Smoking	Rate	2000	3.6606120 1	6.60146687 5	1.5810127 71
YLDs (Years Lived with Disability)	Global	Both	80-84 years	Prostate cancer	Smoking	Number	2001	1596.690859	2897.534236	687.41252 95
YLDs (Years Lived with Disability)	Global	Both	80-84 years	Prostate cancer	Smoking	Rate	2001	3.590429716	6.51559628 5	1.5457634 52
YLDs (Years Lived with Disability)	Global	Both	80-84 years	Prostate cancer	Smoking	Number	2002	1656.74391	3026.343433	708.53693 99
YLDs (Years Lived with Disability)	Global	Both	80-84 years	Prostate cancer	Smoking	Rate	2002	3.541980661	6.47007050 8	1.5147930 37
YLDs (Years Lived with Disability)	Global	Both	80-84 years	Prostate cancer	Smoking	Number	2003	1719.771626	3148.648081	737.01530 3
YLDs (Years Lived with Disability)	Global	Both	80-84 years	Prostate cancer	Smoking	Rate	2003	3.496422813	6.40143424 5	1.4984065 79
YLDs (Years Lived with Disability)	Global	Both	80-84 years	Prostate cancer	Smoking	Number	2004	1781.967479	3285.633737	762.51316 46
YLDs (Years Lived with Disability)	Global	Both	80-84 years	Prostate cancer	Smoking	Rate	2004	3.458638693	6.37711973 1	1.4799695 09
YLDs (Years Lived with Disability)	Global	Both	80-84 years	Prostate cancer	Smoking	Number	2005	1826.900927	3379.196646	780.54421 67

YLDs (Years Lived with Disability)	Global	Both	80-84 years	Prostate cancer	Smoking	Rate	2005	3.399726334	6.28843286	1.452534557
YLDs (Years Lived with Disability)	Global	Both	80-84 years	Prostate cancer	Smoking	Number	2006	1857.506904	3418.427189	787.7587021
YLDs (Years Lived with Disability)	Global	Both	80-84 years	Prostate cancer	Smoking	Rate	2006	3.323703796	6.116714507	1.40956493
YLDs (Years Lived with Disability)	Global	Both	80-84 years	Prostate cancer	Smoking	Number	2007	1884.353592	3434.44192	793.8295621
YLDs (Years Lived with Disability)	Global	Both	80-84 years	Prostate cancer	Smoking	Rate	2007	3.240320864	5.905841586	1.365063597
YLDs (Years Lived with Disability)	Global	Both	80-84 years	Prostate cancer	Smoking	Number	2008	1903.353585	3494.509793	805.1162428
YLDs (Years Lived with Disability)	Global	Both	80-84 years	Prostate cancer	Smoking	Rate	2008	3.147913807	5.779491373	1.331563697
YLDs (Years Lived with Disability)	Global	Both	80-84 years	Prostate cancer	Smoking	Number	2009	1908.054996	3511.679698	804.0052288
YLDs (Years Lived with Disability)	Global	Both	80-84 years	Prostate cancer	Smoking	Rate	2009	3.041177325	5.597134617	1.281473792
YLDs (Years Lived with Disability)	Global	Both	80-84 years	Prostate cancer	Smoking	Number	2010	1916.279844	3544.514024	805.0122166
YLDs (Years Lived with Disability)	Global	Both	80-84 years	Prostate cancer	Smoking	Rate	2010	2.955850143	5.467391579	1.241726506

YLDs (Years Lived with Disability)	Global	Both	80-84 years	Prostate cancer	Smoking	Number	2011	1927.734049	3564.402779	807.879843
YLDs (Years Lived with Disability)	Global	Both	80-84 years	Prostate cancer	Smoking	Rate	2011	2.881941976	5.328744386	1.207783976
YLDs (Years Lived with Disability)	Global	Both	80-84 years	Prostate cancer	Smoking	Number	2012	1908.521661	3517.247178	806.6464007
YLDs (Years Lived with Disability)	Global	Both	80-84 years	Prostate cancer	Smoking	Rate	2012	2.778469966	5.120489774	1.174334483
YLDs (Years Lived with Disability)	Global	Both	80-84 years	Prostate cancer	Smoking	Number	2013	1885.850585	3504.436889	785.6223726
YLDs (Years Lived with Disability)	Global	Both	80-84 years	Prostate cancer	Smoking	Rate	2013	2.677060022	4.974724918	1.115230583
YLDs (Years Lived with Disability)	Global	Both	80-84 years	Prostate cancer	Smoking	Number	2014	1900.854119	3533.364728	792.6683674
YLDs (Years Lived with Disability)	Global	Both	80-84 years	Prostate cancer	Smoking	Rate	2014	2.632178228	4.892771948	1.097635215
YLDs (Years Lived with Disability)	Global	Both	80-84 years	Prostate cancer	Smoking	Number	2015	1949.81815	3604.102916	810.9481342
YLDs (Years Lived with Disability)	Global	Both	80-84 years	Prostate cancer	Smoking	Rate	2015	2.626671216	4.855218621	1.092457839
YLDs (Years Lived with Disability)	Global	Both	80-84 years	Prostate cancer	Smoking	Number	2016	2009.523972	3731.015805	835.9163982

YLDs (Years Lived with Disability)	Global	Both	80-84 years	Prostate cancer	Smoking	Rate	2016	2.624187031	4.872240104	1.09160229
YLDs (Years Lived with Disability)	Global	Both	80-84 years	Prostate cancer	Smoking	Number	2017	2045.358754	3802.235691	850.860431
YLDs (Years Lived with Disability)	Global	Both	80-84 years	Prostate cancer	Smoking	Rate	2017	2.582334698	4.800451331	1.07424011
YLDs (Years Lived with Disability)	Global	Both	80-84 years	Prostate cancer	Smoking	Number	2018	2114.033886	3901.832885	881.497803 5
YLDs (Years Lived with Disability)	Global	Both	80-84 years	Prostate cancer	Smoking	Rate	2018	2.584552195	4.770259746	1.077691847
YLDs (Years Lived with Disability)	Global	Both	80-84 years	Prostate cancer	Smoking	Number	2019	2182.365735	4016.171219	900.723368
YLDs (Years Lived with Disability)	Global	Both	80-84 years	Prostate cancer	Smoking	Rate	2019	2.591973103	4.76996482	1.069779784
YLDs (Years Lived with Disability)	Global	Both	80-84 years	Prostate cancer	Smoking	Number	2020	2216.833316	4081.558171	920.54290 21
YLDs (Years Lived with Disability)	Global	Both	80-84 years	Prostate cancer	Smoking	Rate	2020	2.570448808	4.732622999	1.067382193
YLDs (Years Lived with Disability)	Global	Both	80-84 years	Prostate cancer	Smoking	Number	2021	2240.958502	4166.035713	925.707918
YLDs (Years Lived with Disability)	Global	Both	80-84 years	Prostate cancer	Smoking	Rate	2021	2.558666688	4.756668537	1.056948627

YLDs (Years Lived with Disability)	Global	Both	85-89 years	Prostate cancer	Smoking	Number	1990	469.8483966	841.4730894	201.4310186
YLDs (Years Lived with Disability)	Global	Both	85-89 years	Prostate cancer	Smoking	Rate	1990	3.109298804	5.568586142	1.333002793
YLDs (Years Lived with Disability)	Global	Both	85-89 years	Prostate cancer	Smoking	Number	1991	501.9789216	908.3658961	217.4875688
YLDs (Years Lived with Disability)	Global	Both	85-89 years	Prostate cancer	Smoking	Rate	1991	3.175891698	5.746997701	1.375987984
YLDs (Years Lived with Disability)	Global	Both	85-89 years	Prostate cancer	Smoking	Number	1992	535.9501388	966.8431534	230.9163329
YLDs (Years Lived with Disability)	Global	Both	85-89 years	Prostate cancer	Smoking	Rate	1992	3.235529689	5.836829774	1.394041342
YLDs (Years Lived with Disability)	Global	Both	85-89 years	Prostate cancer	Smoking	Number	1993	570.087208	1037.163916	244.3672327
YLDs (Years Lived with Disability)	Global	Both	85-89 years	Prostate cancer	Smoking	Rate	1993	3.312602736	6.026642902	1.419943392
YLDs (Years Lived with Disability)	Global	Both	85-89 years	Prostate cancer	Smoking	Number	1994	604.5944518	1097.505439	260.104607
YLDs (Years Lived with Disability)	Global	Both	85-89 years	Prostate cancer	Smoking	Rate	1994	3.388986588	6.151944007	1.457987287
YLDs (Years Lived with Disability)	Global	Both	85-89 years	Prostate cancer	Smoking	Number	1995	636.3095999	1155.077576	274.6685114

YLDs (Years Lived with Disability)	Global	Both	85-89 years	Prostate cancer	Smoking	Rate	1995	3.453869283	6.269726181	1.490892381
YLDs (Years Lived with Disability)	Global	Both	85-89 years	Prostate cancer	Smoking	Number	1996	663.274341	1206.320452	284.4884189
YLDs (Years Lived with Disability)	Global	Both	85-89 years	Prostate cancer	Smoking	Rate	1996	3.482895651	6.334465237	1.493866739
YLDs (Years Lived with Disability)	Global	Both	85-89 years	Prostate cancer	Smoking	Number	1997	677.738953	1234.011491	289.3101557
YLDs (Years Lived with Disability)	Global	Both	85-89 years	Prostate cancer	Smoking	Rate	1997	3.448605388	6.279141338	1.472125156
YLDs (Years Lived with Disability)	Global	Both	85-89 years	Prostate cancer	Smoking	Number	1998	687.9521776	1258.72959	293.5119273
YLDs (Years Lived with Disability)	Global	Both	85-89 years	Prostate cancer	Smoking	Rate	1998	3.385627554	6.194601488	1.444463875
YLDs (Years Lived with Disability)	Global	Both	85-89 years	Prostate cancer	Smoking	Number	1999	692.1788001	1265.023451	295.9366971
YLDs (Years Lived with Disability)	Global	Both	85-89 years	Prostate cancer	Smoking	Rate	1999	3.309543356	6.048509372	1.414974469
YLDs (Years Lived with Disability)	Global	Both	85-89 years	Prostate cancer	Smoking	Number	2000	690.3972346	1277.146207	294.337167
YLDs (Years Lived with Disability)	Global	Both	85-89 years	Prostate cancer	Smoking	Rate	2000	3.227115668	5.969749482	1.375816756

YLDs (Years Lived with Disability)	Global	Both	85-89 years	Prostate cancer	Smoking	Number	2001	685.2430675	1268.013123	289.59328117
YLDs (Years Lived with Disability)	Global	Both	85-89 years	Prostate cancer	Smoking	Rate	2001	3.166197179	5.858913081	1.338079106
YLDs (Years Lived with Disability)	Global	Both	85-89 years	Prostate cancer	Smoking	Number	2002	668.3441017	1248.045869	283.9518228
YLDs (Years Lived with Disability)	Global	Both	85-89 years	Prostate cancer	Smoking	Rate	2002	3.064189324	5.721975876	1.301847569
YLDs (Years Lived with Disability)	Global	Both	85-89 years	Prostate cancer	Smoking	Number	2003	645.7675466	1210.898433	272.9125182
YLDs (Years Lived with Disability)	Global	Both	85-89 years	Prostate cancer	Smoking	Rate	2003	2.937535438	5.508262342	1.24145321
YLDs (Years Lived with Disability)	Global	Both	85-89 years	Prostate cancer	Smoking	Number	2004	633.0792825	1184.665663	263.0612491
YLDs (Years Lived with Disability)	Global	Both	85-89 years	Prostate cancer	Smoking	Rate	2004	2.822014977	5.280767095	1.172622143
YLDs (Years Lived with Disability)	Global	Both	85-89 years	Prostate cancer	Smoking	Number	2005	646.7094792	1217.595028	268.2837325
YLDs (Years Lived with Disability)	Global	Both	85-89 years	Prostate cancer	Smoking	Rate	2005	2.76397554	5.203886725	1.146619461
YLDs (Years Lived with Disability)	Global	Both	85-89 years	Prostate cancer	Smoking	Number	2006	670.7719272	1260.119346	278.5215365

YLDs (Years Lived with Disability)	Global	Both	85-89 years	Prostate cancer	Smoking	Rate	2006	2.710020191	5.091073033	1.125269196
YLDs (Years Lived with Disability)	Global	Both	85-89 years	Prostate cancer	Smoking	Number	2007	703.0022647	1333.87316	291.6579285
YLDs (Years Lived with Disability)	Global	Both	85-89 years	Prostate cancer	Smoking	Rate	2007	2.673821678	5.07329656	1.109301251
YLDs (Years Lived with Disability)	Global	Both	85-89 years	Prostate cancer	Smoking	Number	2008	728.8648833	1385.115106	302.2829059
YLDs (Years Lived with Disability)	Global	Both	85-89 years	Prostate cancer	Smoking	Rate	2008	2.613985947	4.967548177	1.084101163
YLDs (Years Lived with Disability)	Global	Both	85-89 years	Prostate cancer	Smoking	Number	2009	749.3194358	1425.866515	308.5576925
YLDs (Years Lived with Disability)	Global	Both	85-89 years	Prostate cancer	Smoking	Rate	2009	2.544898533	4.842641774	1.047948287
YLDs (Years Lived with Disability)	Global	Both	85-89 years	Prostate cancer	Smoking	Number	2010	766.5810499	1452.694837	317.1058646
YLDs (Years Lived with Disability)	Global	Both	85-89 years	Prostate cancer	Smoking	Rate	2010	2.478090822	4.696058876	1.025093345
YLDs (Years Lived with Disability)	Global	Both	85-89 years	Prostate cancer	Smoking	Number	2011	775.5139278	1471.590357	322.1769989
YLDs (Years Lived with Disability)	Global	Both	85-89 years	Prostate cancer	Smoking	Rate	2011	2.399729909	4.553650512	0.996936035

YLDs (Years Lived with Disability)	Global	Both	85-89 years	Prostate cancer	Smoking	Number	2012	784.5376406	1494.793971	324.2131319
YLDs (Years Lived with Disability)	Global	Both	85-89 years	Prostate cancer	Smoking	Rate	2012	2.321642817	4.423468685	0.95942763
YLDs (Years Lived with Disability)	Global	Both	85-89 years	Prostate cancer	Smoking	Number	2013	799.7623586	1523.466236	329.3308539
YLDs (Years Lived with Disability)	Global	Both	85-89 years	Prostate cancer	Smoking	Rate	2013	2.26586034	4.316234299	0.933049315
YLDs (Years Lived with Disability)	Global	Both	85-89 years	Prostate cancer	Smoking	Number	2014	828.5395193	1582.854553	339.3593877
YLDs (Years Lived with Disability)	Global	Both	85-89 years	Prostate cancer	Smoking	Rate	2014	2.255221095	4.308408828	0.923710254
YLDs (Years Lived with Disability)	Global	Both	85-89 years	Prostate cancer	Smoking	Number	2015	867.9939052	1671.899518	359.0160997
YLDs (Years Lived with Disability)	Global	Both	85-89 years	Prostate cancer	Smoking	Rate	2015	2.279801325	4.391273617	0.942962128
YLDs (Years Lived with Disability)	Global	Both	85-89 years	Prostate cancer	Smoking	Number	2016	914.166255	1744.012546	377.3106262
YLDs (Years Lived with Disability)	Global	Both	85-89 years	Prostate cancer	Smoking	Rate	2016	2.317346648	4.420948163	0.956455688
YLDs (Years Lived with Disability)	Global	Both	85-89 years	Prostate cancer	Smoking	Number	2017	928.238217	1774.752442	383.4493272

YLDs (Years Lived with Disability)	Global	Both	85-89 years	Prostate cancer	Smoking	Rate	2017	2.282196461	4.363463675	0.942760901
YLDs (Years Lived with Disability)	Global	Both	85-89 years	Prostate cancer	Smoking	Number	2018	948.4981241	1821.763999	391.2211665
YLDs (Years Lived with Disability)	Global	Both	85-89 years	Prostate cancer	Smoking	Rate	2018	2.262485059	4.345516059	0.933193247
YLDs (Years Lived with Disability)	Global	Both	85-89 years	Prostate cancer	Smoking	Number	2019	978.6211547	1862.319886	402.810419
YLDs (Years Lived with Disability)	Global	Both	85-89 years	Prostate cancer	Smoking	Rate	2019	2.261252343	4.303172056	0.930754459
YLDs (Years Lived with Disability)	Global	Both	85-89 years	Prostate cancer	Smoking	Number	2020	992.4907716	1893.829633	408.6916857
YLDs (Years Lived with Disability)	Global	Both	85-89 years	Prostate cancer	Smoking	Rate	2020	2.225666233	4.246923787	0.916493443
YLDs (Years Lived with Disability)	Global	Both	85-89 years	Prostate cancer	Smoking	Number	2021	1005.396461	1924.71163	415.6276346
YLDs (Years Lived with Disability)	Global	Both	85-89 years	Prostate cancer	Smoking	Rate	2021	2.198943737	4.20961556	0.909036206
YLDs (Years Lived with Disability)	Global	Both	90-94 years	Prostate cancer	Smoking	Number	1990	70.18301371	128.4504962	29.63817885
YLDs (Years Lived with Disability)	Global	Both	90-94 years	Prostate cancer	Smoking	Rate	1990	1.637804947	2.997546657	0.691642513

YLDs (Years Lived with Disability)	Global	Both	90-94 years	Prostate cancer	Smoking	Number	1991	75.78018946	136.909419	32.13463653
YLDs (Years Lived with Disability)	Global	Both	90-94 years	Prostate cancer	Smoking	Rate	1991	1.655735654	2.991359719	0.702115735
YLDs (Years Lived with Disability)	Global	Both	90-94 years	Prostate cancer	Smoking	Number	1992	81.68580073	148.4354823	34.45432729
YLDs (Years Lived with Disability)	Global	Both	90-94 years	Prostate cancer	Smoking	Rate	1992	1.686411658	3.064465617	0.711313089
YLDs (Years Lived with Disability)	Global	Both	90-94 years	Prostate cancer	Smoking	Number	1993	88.02371936	160.0710569	37.41430726
YLDs (Years Lived with Disability)	Global	Both	90-94 years	Prostate cancer	Smoking	Rate	1993	1.707689374	3.105431638	0.72584998
YLDs (Years Lived with Disability)	Global	Both	90-94 years	Prostate cancer	Smoking	Number	1994	94.50118863	173.2952486	39.70451302
YLDs (Years Lived with Disability)	Global	Both	90-94 years	Prostate cancer	Smoking	Rate	1994	1.726435674	3.165918902	0.725359
YLDs (Years Lived with Disability)	Global	Both	90-94 years	Prostate cancer	Smoking	Number	1995	100.8963366	186.331525	42.47087453
YLDs (Years Lived with Disability)	Global	Both	90-94 years	Prostate cancer	Smoking	Rate	1995	1.743342772	3.219539265	0.733835287
YLDs (Years Lived with Disability)	Global	Both	90-94 years	Prostate cancer	Smoking	Number	1996	106.4332536	195.4146625	44.64517562

YLDs (Years Lived with Disability)	Global	Both	90-94 years	Prostate cancer	Smoking	Rate	1996	1.750130792	3.213292901	0.734121094
YLDs (Years Lived with Disability)	Global	Both	90-94 years	Prostate cancer	Smoking	Number	1997	110.6812463	202.3189357	46.19554005
YLDs (Years Lived with Disability)	Global	Both	90-94 years	Prostate cancer	Smoking	Rate	1997	1.727290973	3.157388293	0.720927366
YLDs (Years Lived with Disability)	Global	Both	90-94 years	Prostate cancer	Smoking	Number	1998	113.2955405	207.789182	47.16901388
YLDs (Years Lived with Disability)	Global	Both	90-94 years	Prostate cancer	Smoking	Rate	1998	1.689570907	3.098750006	0.703429219
YLDs (Years Lived with Disability)	Global	Both	90-94 years	Prostate cancer	Smoking	Number	1999	116.4981434	216.7599919	48.49598689
YLDs (Years Lived with Disability)	Global	Both	90-94 years	Prostate cancer	Smoking	Rate	1999	1.663252687	3.094698581	0.692380824
YLDs (Years Lived with Disability)	Global	Both	90-94 years	Prostate cancer	Smoking	Number	2000	119.1355192	219.2085917	49.23480385
YLDs (Years Lived with Disability)	Global	Both	90-94 years	Prostate cancer	Smoking	Rate	2000	1.634282359	3.007069065	0.675395314
YLDs (Years Lived with Disability)	Global	Both	90-94 years	Prostate cancer	Smoking	Number	2001	122.3547248	227.3602792	50.53482157
YLDs (Years Lived with Disability)	Global	Both	90-94 years	Prostate cancer	Smoking	Rate	2001	1.612541463	2.996434162	0.666010203

YLDs (Years Lived with Disability)	Global	Both	90-94 years	Prostate cancer	Smoking	Number	2002	124.7767987	233.3183364	51.47600402
YLDs (Years Lived with Disability)	Global	Both	90-94 years	Prostate cancer	Smoking	Rate	2002	1.585514487	2.964730675	0.654095561
YLDs (Years Lived with Disability)	Global	Both	90-94 years	Prostate cancer	Smoking	Number	2003	126.5349623	234.5485529	51.6931098
YLDs (Years Lived with Disability)	Global	Both	90-94 years	Prostate cancer	Smoking	Rate	2003	1.549126991	2.871502765	0.632862177
YLDs (Years Lived with Disability)	Global	Both	90-94 years	Prostate cancer	Smoking	Number	2004	127.3751217	239.6046616	51.75666414
YLDs (Years Lived with Disability)	Global	Both	90-94 years	Prostate cancer	Smoking	Rate	2004	1.506734829	2.834310844	0.612235478
YLDs (Years Lived with Disability)	Global	Both	90-94 years	Prostate cancer	Smoking	Number	2005	127.7611867	240.9853782	51.95026034
YLDs (Years Lived with Disability)	Global	Both	90-94 years	Prostate cancer	Smoking	Rate	2005	1.466946715	2.766980472	0.596489949
YLDs (Years Lived with Disability)	Global	Both	90-94 years	Prostate cancer	Smoking	Number	2006	127.8644804	242.492435	52.073355
YLDs (Years Lived with Disability)	Global	Both	90-94 years	Prostate cancer	Smoking	Rate	2006	1.437058657	2.725353061	0.585248267
YLDs (Years Lived with Disability)	Global	Both	90-94 years	Prostate cancer	Smoking	Number	2007	127.2022242	240.8179311	51.60513921

YLDs (Years Lived with Disability)	Global	Both	90-94 years	Prostate cancer	Smoking	Rate	2007	1.402211629	2.654652508	0.568868404
YLDs (Years Lived with Disability)	Global	Both	90-94 years	Prostate cancer	Smoking	Number	2008	125.681165	240.4763506	51.62781764
YLDs (Years Lived with Disability)	Global	Both	90-94 years	Prostate cancer	Smoking	Rate	2008	1.356389229	2.595293667	0.557183057
YLDs (Years Lived with Disability)	Global	Both	90-94 years	Prostate cancer	Smoking	Number	2009	125.3621175	241.8485417	51.15042825
YLDs (Years Lived with Disability)	Global	Both	90-94 years	Prostate cancer	Smoking	Rate	2009	1.305256662	2.518100576	0.532572667
YLDs (Years Lived with Disability)	Global	Both	90-94 years	Prostate cancer	Smoking	Number	2010	129.8009151	250.3485818	52.7813745
YLDs (Years Lived with Disability)	Global	Both	90-94 years	Prostate cancer	Smoking	Rate	2010	1.271998566	2.453318879	0.517236973
YLDs (Years Lived with Disability)	Global	Both	90-94 years	Prostate cancer	Smoking	Number	2011	136.4940136	265.0732879	55.50221442
YLDs (Years Lived with Disability)	Global	Both	90-94 years	Prostate cancer	Smoking	Rate	2011	1.248021777	2.423675788	0.507479929
YLDs (Years Lived with Disability)	Global	Both	90-94 years	Prostate cancer	Smoking	Number	2012	142.5234277	276.9058319	58.72324486
YLDs (Years Lived with Disability)	Global	Both	90-94 years	Prostate cancer	Smoking	Rate	2012	1.215959277	2.362462243	0.501005876

YLDs (Years Lived with Disability)	Global	Both	90-94 years	Prostate cancer	Smoking	Number	2013	148.3889941	287.9720108	60.41664907
YLDs (Years Lived with Disability)	Global	Both	90-94 years	Prostate cancer	Smoking	Rate	2013	1.187842091	2.305193033	0.483630469
YLDs (Years Lived with Disability)	Global	Both	90-94 years	Prostate cancer	Smoking	Number	2014	156.1873738	304.6916196	63.17583242
YLDs (Years Lived with Disability)	Global	Both	90-94 years	Prostate cancer	Smoking	Rate	2014	1.182552026	2.306932266	0.478327453
YLDs (Years Lived with Disability)	Global	Both	90-94 years	Prostate cancer	Smoking	Number	2015	164.2653936	321.4953322	66.27743968
YLDs (Years Lived with Disability)	Global	Both	90-94 years	Prostate cancer	Smoking	Rate	2015	1.182508789	2.314370955	0.477116045
YLDs (Years Lived with Disability)	Global	Both	90-94 years	Prostate cancer	Smoking	Number	2016	171.2844802	331.684096	69.96868206
YLDs (Years Lived with Disability)	Global	Both	90-94 years	Prostate cancer	Smoking	Rate	2016	1.175735795	2.276755394	0.480281015
YLDs (Years Lived with Disability)	Global	Both	90-94 years	Prostate cancer	Smoking	Number	2017	177.140136	342.166324	71.84377811
YLDs (Years Lived with Disability)	Global	Both	90-94 years	Prostate cancer	Smoking	Rate	2017	1.158899816	2.238546831	0.470021888
YLDs (Years Lived with Disability)	Global	Both	90-94 years	Prostate cancer	Smoking	Number	2018	184.8931168	359.6544872	75.37497479

YLDs (Years Lived with Disability)	Global	Both	90-94 years	Prostate cancer	Smoking	Rate	2018	1.15242638	2.241702266	0.469807156
YLDs (Years Lived with Disability)	Global	Both	90-94 years	Prostate cancer	Smoking	Number	2019	192.9614516	374.9737708	77.70285136
YLDs (Years Lived with Disability)	Global	Both	90-94 years	Prostate cancer	Smoking	Rate	2019	1.147198106	2.229301221	0.461960475
YLDs (Years Lived with Disability)	Global	Both	90-94 years	Prostate cancer	Smoking	Number	2020	197.9127867	386.0043394	79.79492592
YLDs (Years Lived with Disability)	Global	Both	90-94 years	Prostate cancer	Smoking	Rate	2020	1.134735976	2.213161757	0.457505423
YLDs (Years Lived with Disability)	Global	Both	90-94 years	Prostate cancer	Smoking	Number	2021	199.5126966	389.0376221	80.55757327
YLDs (Years Lived with Disability)	Global	Both	90-94 years	Prostate cancer	Smoking	Rate	2021	1.115258148	2.174685548	0.450309637
YLDs (Years Lived with Disability)	Global	Both	95+ years	Prostate cancer	Smoking	Number	1990	10.28190169	20.40620305	4.067197836
YLDs (Years Lived with Disability)	Global	Both	95+ years	Prostate cancer	Smoking	Rate	1990	1.009924169	2.004368285	0.399494327
YLDs (Years Lived with Disability)	Global	Both	95+ years	Prostate cancer	Smoking	Number	1991	10.97844104	21.9787352	4.341213998
YLDs (Years Lived with Disability)	Global	Both	95+ years	Prostate cancer	Smoking	Rate	1991	1.03594844	2.073958985	0.409645946

YLDs (Years Lived with Disability)	Global	Both	95+ years	Prostate cancer	Smoking	Number	1992	11.66983118	23.31181553	4.55352033 4
YLDs (Years Lived with Disability)	Global	Both	95+ years	Prostate cancer	Smoking	Rate	1992	1.053073551	2.103634234	0.41090498 7
YLDs (Years Lived with Disability)	Global	Both	95+ years	Prostate cancer	Smoking	Number	1993	12.24592143	24.58332292	4.79383076 8
YLDs (Years Lived with Disability)	Global	Both	95+ years	Prostate cancer	Smoking	Rate	1993	1.058554732	2.12501713	0.41438549 8
YLDs (Years Lived with Disability)	Global	Both	95+ years	Prostate cancer	Smoking	Number	1994	12.68141698	25.53755525	4.97357717
YLDs (Years Lived with Disability)	Global	Both	95+ years	Prostate cancer	Smoking	Rate	1994	1.047164321	2.108756203	0.41069168 9
YLDs (Years Lived with Disability)	Global	Both	95+ years	Prostate cancer	Smoking	Number	1995	13.40898714	26.81017889	5.27291645 5
YLDs (Years Lived with Disability)	Global	Both	95+ years	Prostate cancer	Smoking	Rate	1995	1.04736377	2.094118649	0.41186270 1
YLDs (Years Lived with Disability)	Global	Both	95+ years	Prostate cancer	Smoking	Number	1996	14.10113076	28.26798878	5.52598758 5
YLDs (Years Lived with Disability)	Global	Both	95+ years	Prostate cancer	Smoking	Rate	1996	1.029921314	2.06464323	0.40360822 8
YLDs (Years Lived with Disability)	Global	Both	95+ years	Prostate cancer	Smoking	Number	1997	14.58953057	29.46955158	5.70287988 9

YLDs (Years Lived with Disability)	Global	Both	95+ years	Prostate cancer	Smoking	Rate	1997	1.0041296 34	2.028252375	0.392502738
YLDs (Years Lived with Disability)	Global	Both	95+ years	Prostate cancer	Smoking	Number	1998	15.14248241	30.61725603	5.89838750 1
YLDs (Years Lived with Disability)	Global	Both	95+ years	Prostate cancer	Smoking	Rate	1998	0.974664785	1.970717909	0.379657075
YLDs (Years Lived with Disability)	Global	Both	95+ years	Prostate cancer	Smoking	Number	1999	15.74766307	32.15788127	6.15227682 7
YLDs (Years Lived with Disability)	Global	Both	95+ years	Prostate cancer	Smoking	Rate	1999	0.947207975	1.934268055	0.370053998
YLDs (Years Lived with Disability)	Global	Both	95+ years	Prostate cancer	Smoking	Number	2000	16.36585875	33.44227657	6.33020052
YLDs (Years Lived with Disability)	Global	Both	95+ years	Prostate cancer	Smoking	Rate	2000	0.917856892	1.87556452	0.355020672
YLDs (Years Lived with Disability)	Global	Both	95+ years	Prostate cancer	Smoking	Number	2001	17.22719323	35.48511974	6.68637647 8
YLDs (Years Lived with Disability)	Global	Both	95+ years	Prostate cancer	Smoking	Rate	2001	0.902525964	1.859051645	0.350296667
YLDs (Years Lived with Disability)	Global	Both	95+ years	Prostate cancer	Smoking	Number	2002	17.88498311	37.18273151	6.93528779 9
YLDs (Years Lived with Disability)	Global	Both	95+ years	Prostate cancer	Smoking	Rate	2002	0.87659473	1.822433171	0.339918506

YLDs (Years Lived with Disability)	Global	Both	95+ years	Prostate cancer	Smoking	Number	2003	18.5359036	38.6256425	7.129366671
YLDs (Years Lived with Disability)	Global	Both	95+ years	Prostate cancer	Smoking	Rate	2003	0.857463791	1.786807407	0.329801768
YLDs (Years Lived with Disability)	Global	Both	95+ years	Prostate cancer	Smoking	Number	2004	19.18496055	39.79323766	7.34703924
YLDs (Years Lived with Disability)	Global	Both	95+ years	Prostate cancer	Smoking	Rate	2004	0.839416606	1.741108845	0.321461529
YLDs (Years Lived with Disability)	Global	Both	95+ years	Prostate cancer	Smoking	Number	2005	19.63009276	41.05463525	7.491227244
YLDs (Years Lived with Disability)	Global	Both	95+ years	Prostate cancer	Smoking	Rate	2005	0.813926313	1.70225624	0.310610197
YLDs (Years Lived with Disability)	Global	Both	95+ years	Prostate cancer	Smoking	Number	2006	20.23865225	42.62052451	7.696272752
YLDs (Years Lived with Disability)	Global	Both	95+ years	Prostate cancer	Smoking	Rate	2006	0.795351531	1.674928696	0.302453061
YLDs (Years Lived with Disability)	Global	Both	95+ years	Prostate cancer	Smoking	Number	2007	20.92056131	44.1178088	7.922067334
YLDs (Years Lived with Disability)	Global	Both	95+ years	Prostate cancer	Smoking	Rate	2007	0.780466847	1.645868227	0.295542305
YLDs (Years Lived with Disability)	Global	Both	95+ years	Prostate cancer	Smoking	Number	2008	21.6639041	45.94271111	8.221636601

YLDs (Years Lived with Disability)	Global	Both	95+ years	Prostate cancer	Smoking	Rate	2008	0.7668382	1.626236238	0.291021645
YLDs (Years Lived with Disability)	Global	Both	95+ years	Prostate cancer	Smoking	Number	2009	22.25366183	47.1302339	8.349685519
YLDs (Years Lived with Disability)	Global	Both	95+ years	Prostate cancer	Smoking	Rate	2009	0.747198911	1.58246583	0.280352778
YLDs (Years Lived with Disability)	Global	Both	95+ years	Prostate cancer	Smoking	Number	2010	22.79328796	48.0846745	8.452149098
YLDs (Years Lived with Disability)	Global	Both	95+ years	Prostate cancer	Smoking	Rate	2010	0.728831397	1.537541251	0.270263406
YLDs (Years Lived with Disability)	Global	Both	95+ years	Prostate cancer	Smoking	Number	2011	22.97317403	48.89180675	8.531582481
YLDs (Years Lived with Disability)	Global	Both	95+ years	Prostate cancer	Smoking	Rate	2011	0.705847248	1.502193262	0.262131563
YLDs (Years Lived with Disability)	Global	Both	95+ years	Prostate cancer	Smoking	Number	2012	22.91326416	48.67933348	8.509735324
YLDs (Years Lived with Disability)	Global	Both	95+ years	Prostate cancer	Smoking	Rate	2012	0.678782604	1.442076717	0.252092424
YLDs (Years Lived with Disability)	Global	Both	95+ years	Prostate cancer	Smoking	Number	2013	23.25521734	49.55416081	8.645029288
YLDs (Years Lived with Disability)	Global	Both	95+ years	Prostate cancer	Smoking	Rate	2013	0.664842649	1.416702284	0.24715246

YLDs (Years Lived with Disability)	Global	Both	95+ years	Prostate cancer	Smoking	Number	2014	24.16968759	51.836942	8.94079368
YLDs (Years Lived with Disability)	Global	Both	95+ years	Prostate cancer	Smoking	Rate	2014	0.662890223	1.421706503	0.245214784
YLDs (Years Lived with Disability)	Global	Both	95+ years	Prostate cancer	Smoking	Number	2015	25.51600347	55.50960684	9.255021211
YLDs (Years Lived with Disability)	Global	Both	95+ years	Prostate cancer	Smoking	Rate	2015	0.658497429	1.432549319	0.238846482
YLDs (Years Lived with Disability)	Global	Both	95+ years	Prostate cancer	Smoking	Number	2016	27.21967723	59.23924253	9.871582199
YLDs (Years Lived with Disability)	Global	Both	95+ years	Prostate cancer	Smoking	Rate	2016	0.658831357	1.433840313	0.238934056
YLDs (Years Lived with Disability)	Global	Both	95+ years	Prostate cancer	Smoking	Number	2017	29.03415965	63.67491662	10.57620306
YLDs (Years Lived with Disability)	Global	Both	95+ years	Prostate cancer	Smoking	Rate	2017	0.660330498	1.448173114	0.240536992
YLDs (Years Lived with Disability)	Global	Both	95+ years	Prostate cancer	Smoking	Number	2018	30.79052538	67.86717035	11.13424666
YLDs (Years Lived with Disability)	Global	Both	95+ years	Prostate cancer	Smoking	Rate	2018	0.659860359	1.454436222	0.238613921
YLDs (Years Lived with Disability)	Global	Both	95+ years	Prostate cancer	Smoking	Number	2019	32.59774617	71.97942679	11.65816899

YLDs (Years Lived with Disability)	Global	Both	95+ years	Prostate cancer	Smoking	Rate	2019	0.658271048	1.453535237	0.235422261
YLDs (Years Lived with Disability)	Global	Both	95+ years	Prostate cancer	Smoking	Number	2020	33.99437265	74.01345114	12.17007837
YLDs (Years Lived with Disability)	Global	Both	95+ years	Prostate cancer	Smoking	Rate	2020	0.650234167	1.4157071	0.2327856 1
YLDs (Years Lived with Disability)	Global	Both	95+ years	Prostate cancer	Smoking	Number	2021	34.78296225	76.24063678	12.47160653
YLDs (Years Lived with Disability)	Global	Both	95+ years	Prostate cancer	Smoking	Rate	2021	0.638183346	1.39883154	0.228823857
YLLs (Years of Life Lost)	Global	Both	80-84 years	Prostate cancer	Smoking	Number	1990	17978.41622	30240.37033	8125.560707
YLLs (Years of Life Lost)	Global	Both	80-84 years	Prostate cancer	Smoking	Rate	1990	50.82104597	85.48290529	22.96918089
YLLs (Years of Life Lost)	Global	Both	80-84 years	Prostate cancer	Smoking	Number	1991	18562.32359	31312.07949	8408.287087
YLLs (Years of Life Lost)	Global	Both	80-84 years	Prostate cancer	Smoking	Rate	1991	50.86760072	85.80662595	23.04180229
YLLs (Years of Life Lost)	Global	Both	80-84 years	Prostate cancer	Smoking	Number	1992	19130.67433	32371.41503	8672.013993
YLLs (Years of Life Lost)	Global	Both	80-84 years	Prostate cancer	Smoking	Rate	1992	51.00559844	86.30764225	23.12104925

YLLs (Years of Life Lost)	Global	Both	80-84 years	Prostate cancer	Smoking	Number	1993	19645.82668	33491.73114	8848.285066
YLLs (Years of Life Lost)	Global	Both	80-84 years	Prostate cancer	Smoking	Rate	1993	50.93455175	86.83199445	22.94041584
YLLs (Years of Life Lost)	Global	Both	80-84 years	Prostate cancer	Smoking	Number	1994	20099.264	34354.78431	9089.720796
YLLs (Years of Life Lost)	Global	Both	80-84 years	Prostate cancer	Smoking	Rate	1994	50.95438561	87.09408103	23.04368651
YLLs (Years of Life Lost)	Global	Both	80-84 years	Prostate cancer	Smoking	Number	1995	20135.18411	34394.73675	9092.835421
YLLs (Years of Life Lost)	Global	Both	80-84 years	Prostate cancer	Smoking	Rate	1995	50.23712289	85.81459236	22.68655146
YLLs (Years of Life Lost)	Global	Both	80-84 years	Prostate cancer	Smoking	Number	1996	19796.92047	33825.04088	8973.306815
YLLs (Years of Life Lost)	Global	Both	80-84 years	Prostate cancer	Smoking	Rate	1996	49.16474319	84.00293624	22.28479554
YLLs (Years of Life Lost)	Global	Both	80-84 years	Prostate cancer	Smoking	Number	1997	19151.89378	32584.77725	8578.857416
YLLs (Years of Life Lost)	Global	Both	80-84 years	Prostate cancer	Smoking	Rate	1997	47.46881805	80.76281543	21.26307855
YLLs (Years of Life Lost)	Global	Both	80-84 years	Prostate cancer	Smoking	Number	1998	18617.65937	31795.41461	8336.05263

YLLs (Years of Life Lost)	Global	Both	80-84 years	Prostate cancer	Smoking	Rate	1998	46.02288931	78.59832527	20.60673793
YLLs (Years of Life Lost)	Global	Both	80-84 years	Prostate cancer	Smoking	Number	1999	18183.78328	31027.88852	8162.259265
YLLs (Years of Life Lost)	Global	Both	80-84 years	Prostate cancer	Smoking	Rate	1999	44.30597345	75.60147322	19.88787684
YLLs (Years of Life Lost)	Global	Both	80-84 years	Prostate cancer	Smoking	Number	2000	18327.84559	31349.31069	8221.104872
YLLs (Years of Life Lost)	Global	Both	80-84 years	Prostate cancer	Smoking	Rate	2000	43.18405207	73.86521556	19.37055936
YLLs (Years of Life Lost)	Global	Both	80-84 years	Prostate cancer	Smoking	Number	2001	18878.21971	32244.04935	8456.738667
YLLs (Years of Life Lost)	Global	Both	80-84 years	Prostate cancer	Smoking	Rate	2001	42.45087313	72.5062039	19.01640863
YLLs (Years of Life Lost)	Global	Both	80-84 years	Prostate cancer	Smoking	Number	2002	19483.10088	33442.23839	8703.391376
YLLs (Years of Life Lost)	Global	Both	80-84 years	Prostate cancer	Smoking	Rate	2002	41.65324895	71.49672374	18.60712676
YLLs (Years of Life Lost)	Global	Both	80-84 years	Prostate cancer	Smoking	Number	2003	20102.50972	34478.99703	8891.61904
YLLs (Years of Life Lost)	Global	Both	80-84 years	Prostate cancer	Smoking	Rate	2003	40.86988791	70.09834909	18.07731863

YLLs (Years of Life Lost)	Global	Both	80-84 years	Prostate cancer	Smoking	Number	2004	20627.35606	35662.8191	9075.264173
YLLs (Years of Life Lost)	Global	Both	80-84 years	Prostate cancer	Smoking	Rate	2004	40.03584388	69.21832608	17.61427197
YLLs (Years of Life Lost)	Global	Both	80-84 years	Prostate cancer	Smoking	Number	2005	20846.26313	35924.28815	9197.73373
YLLs (Years of Life Lost)	Global	Both	80-84 years	Prostate cancer	Smoking	Rate	2005	38.7933405	66.8524202	17.11629629
YLLs (Years of Life Lost)	Global	Both	80-84 years	Prostate cancer	Smoking	Number	2006	20998.48509	36059.65422	9236.140475
YLLs (Years of Life Lost)	Global	Both	80-84 years	Prostate cancer	Smoking	Rate	2006	37.57334333	64.52283402	16.52655778
YLLs (Years of Life Lost)	Global	Both	80-84 years	Prostate cancer	Smoking	Number	2007	21245.9067	36469.68256	9356.424731
YLLs (Years of Life Lost)	Global	Both	80-84 years	Prostate cancer	Smoking	Rate	2007	36.53430812	62.71300343	16.08924057
YLLs (Years of Life Lost)	Global	Both	80-84 years	Prostate cancer	Smoking	Number	2008	21494.61924	36793.01177	9480.184338
YLLs (Years of Life Lost)	Global	Both	80-84 years	Prostate cancer	Smoking	Rate	2008	35.54946869	60.85113699	15.67906426
YLLs (Years of Life Lost)	Global	Both	80-84 years	Prostate cancer	Smoking	Number	2009	21565.46693	37167.59694	9521.262591

YLLs (Years of Life Lost)	Global	Both	80-84 years	Prostate cancer	Smoking	Rate	2009	34.37238924	59.24003934	15.17558349
YLLs (Years of Life Lost)	Global	Both	80-84 years	Prostate cancer	Smoking	Number	2010	21694.15012	37254.50637	9565.182848
YLLs (Years of Life Lost)	Global	Both	80-84 years	Prostate cancer	Smoking	Rate	2010	33.46309618	57.46485217	14.75423705
YLLs (Years of Life Lost)	Global	Both	80-84 years	Prostate cancer	Smoking	Number	2011	21893.52135	37615.40946	9646.451791
YLLs (Years of Life Lost)	Global	Both	80-84 years	Prostate cancer	Smoking	Rate	2011	32.73058243	56.23463857	14.42134321
YLLs (Years of Life Lost)	Global	Both	80-84 years	Prostate cancer	Smoking	Number	2012	21729.80027	37041.36839	9533.008346
YLLs (Years of Life Lost)	Global	Both	80-84 years	Prostate cancer	Smoking	Rate	2012	31.63474571	53.92568064	13.87837399
YLLs (Years of Life Lost)	Global	Both	80-84 years	Prostate cancer	Smoking	Number	2013	21322.01572	36622.65955	9307.065796
YLLs (Years of Life Lost)	Global	Both	80-84 years	Prostate cancer	Smoking	Rate	2013	30.26767674	51.98771238	13.21184932
YLLs (Years of Life Lost)	Global	Both	80-84 years	Prostate cancer	Smoking	Number	2014	21416.74579	37111.3626	9353.349314
YLLs (Years of Life Lost)	Global	Both	80-84 years	Prostate cancer	Smoking	Rate	2014	29.65650621	51.38938317	12.95190524

YLLs (Years of Life Lost)	Global	Both	80-84 years	Prostate cancer	Smoking	Number	2015	21896.45675	37641.2558	9477.512474
YLLs (Years of Life Lost)	Global	Both	80-84 years	Prostate cancer	Smoking	Rate	2015	29.49751631	50.70790994	12.7675030 7
YLLs (Years of Life Lost)	Global	Both	80-84 years	Prostate cancer	Smoking	Number	2016	22444.71731	38662.87159	9809.561152
YLLs (Years of Life Lost)	Global	Both	80-84 years	Prostate cancer	Smoking	Rate	2016	29.30999425	50.48887577	12.81006025
YLLs (Years of Life Lost)	Global	Both	80-84 years	Prostate cancer	Smoking	Number	2017	22580.48053	38996.8186	9842.086136
YLLs (Years of Life Lost)	Global	Both	80-84 years	Prostate cancer	Smoking	Rate	2017	28.50862141	49.23480419	12.42596707
YLLs (Years of Life Lost)	Global	Both	80-84 years	Prostate cancer	Smoking	Number	2018	23103.85777	40025.8095	10051.80473
YLLs (Years of Life Lost)	Global	Both	80-84 years	Prostate cancer	Smoking	Rate	2018	28.24605921	48.93431202	12.28902439
YLLs (Years of Life Lost)	Global	Both	80-84 years	Prostate cancer	Smoking	Number	2019	23733.09835	41051.12001	10403.16511
YLLs (Years of Life Lost)	Global	Both	80-84 years	Prostate cancer	Smoking	Rate	2019	28.18755426	48.75598862	12.35573109
YLLs (Years of Life Lost)	Global	Both	80-84 years	Prostate cancer	Smoking	Number	2020	23895.31918	41472.52964	10611.6885

YLLs (Years of Life Lost)	Global	Both	80-84 years	Prostate cancer	Smoking	Rate	2020	27.70695219	48.08797018	12.30439919
YLLs (Years of Life Lost)	Global	Both	80-84 years	Prostate cancer	Smoking	Number	2021	23871.64416	43016.83521	10545.34033
YLLs (Years of Life Lost)	Global	Both	80-84 years	Prostate cancer	Smoking	Rate	2021	27.25600704	49.1154759	12.04038852
YLLs (Years of Life Lost)	Global	Both	85-89 years	Prostate cancer	Smoking	Number	1990	7764.334891	13289.30509	3484.93999
YLLs (Years of Life Lost)	Global	Both	85-89 years	Prostate cancer	Smoking	Rate	1990	51.38175923	87.94415546	23.0621618
YLLs (Years of Life Lost)	Global	Both	85-89 years	Prostate cancer	Smoking	Number	1991	8178.484359	14034.61732	3657.110557
YLLs (Years of Life Lost)	Global	Both	85-89 years	Prostate cancer	Smoking	Rate	1991	51.7431698	88.79341886	23.13759913
YLLs (Years of Life Lost)	Global	Both	85-89 years	Prostate cancer	Smoking	Number	1992	8628.603783	14930.79437	3854.682233
YLLs (Years of Life Lost)	Global	Both	85-89 years	Prostate cancer	Smoking	Rate	1992	52.09086013	90.13716941	23.2707073
YLLs (Years of Life Lost)	Global	Both	85-89 years	Prostate cancer	Smoking	Number	1993	9039.638925	15755.81017	4036.49892
YLLs (Years of Life Lost)	Global	Both	85-89 years	Prostate cancer	Smoking	Rate	1993	52.52658228	91.55220316	23.45486301

YLLs (Years of Life Lost)	Global	Both	85-89 years	Prostate cancer	Smoking	Number	1994	9429.483881	16299.02388	4207.18871
YLLs (Years of Life Lost)	Global	Both	85-89 years	Prostate cancer	Smoking	Rate	1994	52.8559174	91.36235565	23.58292583
YLLs (Years of Life Lost)	Global	Both	85-89 years	Prostate cancer	Smoking	Number	1995	9706.372523	16675.98403	4307.16728
YLLs (Years of Life Lost)	Global	Both	85-89 years	Prostate cancer	Smoking	Rate	1995	52.68589678	90.51673747	23.37917386
YLLs (Years of Life Lost)	Global	Both	85-89 years	Prostate cancer	Smoking	Number	1996	9985.540478	17165.40249	4415.644253
YLLs (Years of Life Lost)	Global	Both	85-89 years	Prostate cancer	Smoking	Rate	1996	52.43470666	90.13661764	23.18682816
YLLs (Years of Life Lost)	Global	Both	85-89 years	Prostate cancer	Smoking	Number	1997	10142.61356	17382.90934	4478.048435
YLLs (Years of Life Lost)	Global	Both	85-89 years	Prostate cancer	Smoking	Rate	1997	51.60965238	88.45115739	22.78609175
YLLs (Years of Life Lost)	Global	Both	85-89 years	Prostate cancer	Smoking	Number	1998	10326.45082	17767.26717	4534.689515
YLLs (Years of Life Lost)	Global	Both	85-89 years	Prostate cancer	Smoking	Rate	1998	50.81969003	87.43827149	22.31662355
YLLs (Years of Life Lost)	Global	Both	85-89 years	Prostate cancer	Smoking	Number	1999	10439.89432	18033.856	4578.160704

YLLs (Years of Life Lost)	Global	Both	85-89 years	Prostate cancer	Smoking	Rate	1999	49.91670197	86.22602758	21.88975066
YLLs (Years of Life Lost)	Global	Both	85-89 years	Prostate cancer	Smoking	Number	2000	10501.07824	18130.06272	4607.374743
YLLs (Years of Life Lost)	Global	Both	85-89 years	Prostate cancer	Smoking	Rate	2000	49.08506644	84.7451388	21.53619753
YLLs (Years of Life Lost)	Global	Both	85-89 years	Prostate cancer	Smoking	Number	2001	10469.42081	18135.59993	4576.482962
YLLs (Years of Life Lost)	Global	Both	85-89 years	Prostate cancer	Smoking	Rate	2001	48.37444144	83.79637539	21.14585047
YLLs (Years of Life Lost)	Global	Both	85-89 years	Prostate cancer	Smoking	Number	2002	10196.63955	17616.75566	4438.102651
YLLs (Years of Life Lost)	Global	Both	85-89 years	Prostate cancer	Smoking	Rate	2002	46.74902339	80.76838634	20.34758252
YLLs (Years of Life Lost)	Global	Both	85-89 years	Prostate cancer	Smoking	Number	2003	9888.74116	17123.83276	4296.414798
YLLs (Years of Life Lost)	Global	Both	85-89 years	Prostate cancer	Smoking	Rate	2003	44.98294743	77.89469421	19.5439842
YLLs (Years of Life Lost)	Global	Both	85-89 years	Prostate cancer	Smoking	Number	2004	9676.385445	16824.62899	4184.314443
YLLs (Years of Life Lost)	Global	Both	85-89 years	Prostate cancer	Smoking	Rate	2004	43.13346748	74.99748658	18.65200513

YLLs (Years of Life Lost)	Global	Both	85-89 years	Prostate cancer	Smoking	Number	2005	9782.16646	17045.37767	4256.444961
YLLs (Years of Life Lost)	Global	Both	85-89 years	Prostate cancer	Smoking	Rate	2005	41.80806017	72.85034231	18.19164576
YLLs (Years of Life Lost)	Global	Both	85-89 years	Prostate cancer	Smoking	Number	2006	10070.51324	17486.08173	4351.007885
YLLs (Years of Life Lost)	Global	Both	85-89 years	Prostate cancer	Smoking	Rate	2006	40.68639891	70.64641888	17.57873092
YLLs (Years of Life Lost)	Global	Both	85-89 years	Prostate cancer	Smoking	Number	2007	10512.62338	18246.24215	4519.908954
YLLs (Years of Life Lost)	Global	Both	85-89 years	Prostate cancer	Smoking	Rate	2007	39.98405368	69.39835085	17.19116872
YLLs (Years of Life Lost)	Global	Both	85-89 years	Prostate cancer	Smoking	Number	2008	10890.48681	18981.76793	4704.694425
YLLs (Years of Life Lost)	Global	Both	85-89 years	Prostate cancer	Smoking	Rate	2008	39.05741671	68.07582001	16.87281881
YLLs (Years of Life Lost)	Global	Both	85-89 years	Prostate cancer	Smoking	Number	2009	11145.64834	19486.23434	4849.896295
YLLs (Years of Life Lost)	Global	Both	85-89 years	Prostate cancer	Smoking	Rate	2009	37.85374135	66.18070588	16.47160527
YLLs (Years of Life Lost)	Global	Both	85-89 years	Prostate cancer	Smoking	Number	2010	11376.56722	20048.9557	4912.684489

YLLs (Years of Life Lost)	Global	Both	85-89 years	Prostate cancer	Smoking	Rate	2010	36.77649848	64.81132442	15.88100612
YLLs (Years of Life Lost)	Global	Both	85-89 years	Prostate cancer	Smoking	Number	2011	11598.44191	20231.43137	5003.897016
YLLs (Years of Life Lost)	Global	Both	85-89 years	Prostate cancer	Smoking	Rate	2011	35.88991372	62.60360933	15.48392737
YLLs (Years of Life Lost)	Global	Both	85-89 years	Prostate cancer	Smoking	Number	2012	11798.82705	20481.41386	5057.346823
YLLs (Years of Life Lost)	Global	Both	85-89 years	Prostate cancer	Smoking	Rate	2012	34.91567601	60.60961886	14.96595232
YLLs (Years of Life Lost)	Global	Both	85-89 years	Prostate cancer	Smoking	Number	2013	11966.51924	20911.50034	5094.048651
YLLs (Years of Life Lost)	Global	Both	85-89 years	Prostate cancer	Smoking	Rate	2013	33.90314769	59.24577315	14.43229065
YLLs (Years of Life Lost)	Global	Both	85-89 years	Prostate cancer	Smoking	Number	2014	12378.97672	21597.22716	5242.573073
YLLs (Years of Life Lost)	Global	Both	85-89 years	Prostate cancer	Smoking	Rate	2014	33.69462625	58.78599773	14.26988226
YLLs (Years of Life Lost)	Global	Both	85-89 years	Prostate cancer	Smoking	Number	2015	13053.93315	22557.68581	5579.414351
YLLs (Years of Life Lost)	Global	Both	85-89 years	Prostate cancer	Smoking	Rate	2015	34.28638601	59.24816026	14.65443035

YLLs (Years of Life Lost)	Global	Both	85-89 years	Prostate cancer	Smoking	Number	2016	13718.02203	23814.84811	5905.410937
YLLs (Years of Life Lost)	Global	Both	85-89 years	Prostate cancer	Smoking	Rate	2016	34.77421331	60.36895164	14.96979807
YLLs (Years of Life Lost)	Global	Both	85-89 years	Prostate cancer	Smoking	Number	2017	13681.36575	24116.66562	5873.097151
YLLs (Years of Life Lost)	Global	Both	85-89 years	Prostate cancer	Smoking	Rate	2017	33.63744772	59.29401304	14.43978635
YLLs (Years of Life Lost)	Global	Both	85-89 years	Prostate cancer	Smoking	Number	2018	13870.72852	24422.5533	5972.073306
YLLs (Years of Life Lost)	Global	Both	85-89 years	Prostate cancer	Smoking	Rate	2018	33.08632377	58.25595278	14.24539098
YLLs (Years of Life Lost)	Global	Both	85-89 years	Prostate cancer	Smoking	Number	2019	14289.55662	25015.98758	6129.010873
YLLs (Years of Life Lost)	Global	Both	85-89 years	Prostate cancer	Smoking	Rate	2019	33.01818404	57.80322678	14.16200757
YLLs (Years of Life Lost)	Global	Both	85-89 years	Prostate cancer	Smoking	Number	2020	14420.24253	25464.91434	6167.460049
YLLs (Years of Life Lost)	Global	Both	85-89 years	Prostate cancer	Smoking	Rate	2020	32.33747637	57.10521609	13.83056444
YLLs (Years of Life Lost)	Global	Both	85-89 years	Prostate cancer	Smoking	Number	2021	14401.505	26425.75438	6142.861509

YLLs (Years of Life Lost)	Global	Both	85-89 years	Prostate cancer	Smoking	Rate	2021	31.49812086	57.79684867	13.43530375
YLLs (Years of Life Lost)	Global	Both	90-94 years	Prostate cancer	Smoking	Number	1990	2213.592519	3907.98688	956.442322 7
YLLs (Years of Life Lost)	Global	Both	90-94 years	Prostate cancer	Smoking	Rate	1990	51.65684099	91.19756914	22.31973073
YLLs (Years of Life Lost)	Global	Both	90-94 years	Prostate cancer	Smoking	Number	1991	2377.271632	4189.518811	1025.426926
YLLs (Years of Life Lost)	Global	Both	90-94 years	Prostate cancer	Smoking	Rate	1991	51.94145629	91.53758674	22.40474633
YLLs (Years of Life Lost)	Global	Both	90-94 years	Prostate cancer	Smoking	Number	1992	2560.727917	4517.436867	1100.937396
YLLs (Years of Life Lost)	Global	Both	90-94 years	Prostate cancer	Smoking	Rate	1992	52.8664881	93.26294324	22.72896442
YLLs (Years of Life Lost)	Global	Both	90-94 years	Prostate cancer	Smoking	Number	1993	2755.579393	4907.189402	1182.231629
YLLs (Years of Life Lost)	Global	Both	90-94 years	Prostate cancer	Smoking	Rate	1993	53.45915492	95.2011033	22.93568603
YLLs (Years of Life Lost)	Global	Both	90-94 years	Prostate cancer	Smoking	Number	1994	2949.679659	5238.39122	1249.823361
YLLs (Years of Life Lost)	Global	Both	90-94 years	Prostate cancer	Smoking	Rate	1994	53.88749352	95.6998066	22.83293648

YLLs (Years of Life Lost)	Global	Both	90-94 years	Prostate cancer	Smoking	Number	1995	3127.199817	5550.95924	1330.606107
YLLs (Years of Life Lost)	Global	Both	90-94 years	Prostate cancer	Smoking	Rate	1995	54.03349004	95.91254745	22.99094911
YLLs (Years of Life Lost)	Global	Both	90-94 years	Prostate cancer	Smoking	Number	1996	3290.469742	5826.038255	1393.781507
YLLs (Years of Life Lost)	Global	Both	90-94 years	Prostate cancer	Smoking	Rate	1996	54.10670276	95.80021848	22.91858842
YLLs (Years of Life Lost)	Global	Both	90-94 years	Prostate cancer	Smoking	Number	1997	3401.824893	6051.245887	1428.052876
YLLs (Years of Life Lost)	Global	Both	90-94 years	Prostate cancer	Smoking	Rate	1997	53.08886216	94.43571285	22.286186
YLLs (Years of Life Lost)	Global	Both	90-94 years	Prostate cancer	Smoking	Number	1998	3466.337639	6160.96065	1453.311296
YLLs (Years of Life Lost)	Global	Both	90-94 years	Prostate cancer	Smoking	Rate	1998	51.69332529	91.87810772	21.67316096
YLLs (Years of Life Lost)	Global	Both	90-94 years	Prostate cancer	Smoking	Number	1999	3552.708406	6318.22386	1485.263477
YLLs (Years of Life Lost)	Global	Both	90-94 years	Prostate cancer	Smoking	Rate	1999	50.72228305	90.20575358	21.20521751
YLLs (Years of Life Lost)	Global	Both	90-94 years	Prostate cancer	Smoking	Number	2000	3627.253362	6439.635586	1522.66276

YLLs (Years of Life Lost)	Global	Both	90-94 years	Prostate cancer	Smoking	Rate	2000	49.75809247	88.33791053	20.88764882
YLLs (Years of Life Lost)	Global	Both	90-94 years	Prostate cancer	Smoking	Number	2001	3726.17405	6642.352146	1571.148357
YLLs (Years of Life Lost)	Global	Both	90-94 years	Prostate cancer	Smoking	Rate	2001	49.10811711	87.54110858	20.70653075
YLLs (Years of Life Lost)	Global	Both	90-94 years	Prostate cancer	Smoking	Number	2002	3801.15217	6789.629492	1605.95124
YLLs (Years of Life Lost)	Global	Both	90-94 years	Prostate cancer	Smoking	Rate	2002	48.30050056	86.27450005	20.40650974
YLLs (Years of Life Lost)	Global	Both	90-94 years	Prostate cancer	Smoking	Number	2003	3855.176794	6888.131615	1621.268595
YLLs (Years of Life Lost)	Global	Both	90-94 years	Prostate cancer	Smoking	Rate	2003	47.19769399	84.32918787	19.84867183
YLLs (Years of Life Lost)	Global	Both	90-94 years	Prostate cancer	Smoking	Number	2004	3845.902319	6882.979398	1609.058339
YLLs (Years of Life Lost)	Global	Both	90-94 years	Prostate cancer	Smoking	Rate	2004	45.49361675	81.41954758	19.03373443
YLLs (Years of Life Lost)	Global	Both	90-94 years	Prostate cancer	Smoking	Number	2005	3820.845792	6895.831426	1595.952522
YLLs (Years of Life Lost)	Global	Both	90-94 years	Prostate cancer	Smoking	Rate	2005	43.87073511	79.17754612	18.32463652

YLLs (Years of Life Lost)	Global	Both	90-94 years	Prostate cancer	Smoking	Number	2006	3797.935589	6881.800605	1585.602693
YLLs (Years of Life Lost)	Global	Both	90-94 years	Prostate cancer	Smoking	Rate	2006	42.68469398	77.34400597	17.82046171
YLLs (Years of Life Lost)	Global	Both	90-94 years	Prostate cancer	Smoking	Number	2007	3776.160906	6836.701397	1581.252831
YLLs (Years of Life Lost)	Global	Both	90-94 years	Prostate cancer	Smoking	Rate	2007	41.62644773	75.36426556	17.43091461
YLLs (Years of Life Lost)	Global	Both	90-94 years	Prostate cancer	Smoking	Number	2008	3724.724254	6753.450002	1561.288097
YLLs (Years of Life Lost)	Global	Both	90-94 years	Prostate cancer	Smoking	Rate	2008	40.19835316	72.88527948	16.84989439
YLLs (Years of Life Lost)	Global	Both	90-94 years	Prostate cancer	Smoking	Number	2009	3687.261738	6723.178498	1546.105488
YLLs (Years of Life Lost)	Global	Both	90-94 years	Prostate cancer	Smoking	Rate	2009	38.3913661	70.0009995	16.09788132
YLLs (Years of Life Lost)	Global	Both	90-94 years	Prostate cancer	Smoking	Number	2010	3806.759485	6990.908558	1573.830173
YLLs (Years of Life Lost)	Global	Both	90-94 years	Prostate cancer	Smoking	Rate	2010	37.3047646	68.50818896	15.4229245
YLLs (Years of Life Lost)	Global	Both	90-94 years	Prostate cancer	Smoking	Number	2011	4024.473079	7378.721838	1675.658677

YLLs (Years of Life Lost)	Global	Both	90-94 years	Prostate cancer	Smoking	Rate	2011	36.79743832	67.46673574	15.32124718
YLLs (Years of Life Lost)	Global	Both	90-94 years	Prostate cancer	Smoking	Number	2012	4213.156394	7760.98141	1757.734969
YLLs (Years of Life Lost)	Global	Both	90-94 years	Prostate cancer	Smoking	Rate	2012	35.94515449	66.21393789	14.99637068
YLLs (Years of Life Lost)	Global	Both	90-94 years	Prostate cancer	Smoking	Number	2013	4414.185689	8178.634945	1831.924187
YLLs (Years of Life Lost)	Global	Both	90-94 years	Prostate cancer	Smoking	Rate	2013	35.33520522	65.46932199	14.66440736
YLLs (Years of Life Lost)	Global	Both	90-94 years	Prostate cancer	Smoking	Number	2014	4683.510256	8737.590336	1955.645206
YLLs (Years of Life Lost)	Global	Both	90-94 years	Prostate cancer	Smoking	Rate	2014	35.46057794	66.15550863	14.80690881
YLLs (Years of Life Lost)	Global	Both	90-94 years	Prostate cancer	Smoking	Number	2015	4966.483555	9166.788891	2065.456442
YLLs (Years of Life Lost)	Global	Both	90-94 years	Prostate cancer	Smoking	Rate	2015	35.75257286	65.98960493	14.86874589
YLLs (Years of Life Lost)	Global	Both	90-94 years	Prostate cancer	Smoking	Number	2016	5217.957875	9724.433727	2170.695015
YLLs (Years of Life Lost)	Global	Both	90-94 years	Prostate cancer	Smoking	Rate	2016	35.81725467	66.75073422	14.90014638

YLLs (Years of Life Lost)	Global	Both	90-94 years	Prostate cancer	Smoking	Number	2017	5374.992967	10123.48866	2212.930933
YLLs (Years of Life Lost)	Global	Both	90-94 years	Prostate cancer	Smoking	Rate	2017	35.16469222	66.23066583	14.47760688
YLLs (Years of Life Lost)	Global	Both	90-94 years	Prostate cancer	Smoking	Number	2018	5610.728668	10675.30251	2272.668797
YLLs (Years of Life Lost)	Global	Both	90-94 years	Prostate cancer	Smoking	Rate	2018	34.971295	66.53844362	14.16539199
YLLs (Years of Life Lost)	Global	Both	90-94 years	Prostate cancer	Smoking	Number	2019	5889.430784	11106.72059	2406.632724
YLLs (Years of Life Lost)	Global	Both	90-94 years	Prostate cancer	Smoking	Rate	2019	35.01395634	66.03188727	14.30795882
YLLs (Years of Life Lost)	Global	Both	90-94 years	Prostate cancer	Smoking	Number	2020	6045.220488	11394.1751	2479.025455
YLLs (Years of Life Lost)	Global	Both	90-94 years	Prostate cancer	Smoking	Rate	2020	34.6603637	65.32867639	14.21353019
YLLs (Years of Life Lost)	Global	Both	90-94 years	Prostate cancer	Smoking	Number	2021	6066.661845	11641.78173	2489.034759
YLLs (Years of Life Lost)	Global	Both	90-94 years	Prostate cancer	Smoking	Rate	2021	33.91209767	65.07651969	13.91348191
YLLs (Years of Life Lost)	Global	Both	95+ years	Prostate cancer	Smoking	Number	1990	354.599976	667.8065854	139.526016

YLLs (Years of Life Lost)	Global	Both	95+ years	Prostate cancer	Smoking	Rate	1990	34.83004379	65.59428704	13.70472948
YLLs (Years of Life Lost)	Global	Both	95+ years	Prostate cancer	Smoking	Number	1991	381.9265835	722.1133126	149.294941
YLLs (Years of Life Lost)	Global	Both	95+ years	Prostate cancer	Smoking	Rate	1991	36.03938363	68.14010813	14.08777832
YLLs (Years of Life Lost)	Global	Both	95+ years	Prostate cancer	Smoking	Number	1992	409.0815999	777.5471968	160.1043816
YLLs (Years of Life Lost)	Global	Both	95+ years	Prostate cancer	Smoking	Rate	1992	36.91510243	70.16505856	14.44765457
YLLs (Years of Life Lost)	Global	Both	95+ years	Prostate cancer	Smoking	Number	1993	432.4193694	831.3385534	168.9213954
YLLs (Years of Life Lost)	Global	Both	95+ years	Prostate cancer	Smoking	Rate	1993	37.37894061	71.86207791	14.60180383
YLLs (Years of Life Lost)	Global	Both	95+ years	Prostate cancer	Smoking	Number	1994	448.2698017	863.6759204	174.790104
YLLs (Years of Life Lost)	Global	Both	95+ years	Prostate cancer	Smoking	Rate	1994	37.0157486	71.31778813	14.43324204
YLLs (Years of Life Lost)	Global	Both	95+ years	Prostate cancer	Smoking	Number	1995	473.9810452	923.0282632	185.0983994
YLLs (Years of Life Lost)	Global	Both	95+ years	Prostate cancer	Smoking	Rate	1995	37.02222765	72.09689678	14.45786735

YLLs (Years of Life Lost)	Global	Both	95+ years	Prostate cancer	Smoking	Number	1996	505.9296631	985.8773242	197.5287963
YLLs (Years of Life Lost)	Global	Both	95+ years	Prostate cancer	Smoking	Rate	1996	36.95219571	72.00671257	14.42714921
YLLs (Years of Life Lost)	Global	Both	95+ years	Prostate cancer	Smoking	Number	1997	527.4296024	1037.0033	205.229828 9
YLLs (Years of Life Lost)	Global	Both	95+ years	Prostate cancer	Smoking	Rate	1997	36.30053008	71.3721212	14.12501601
YLLs (Years of Life Lost)	Global	Both	95+ years	Prostate cancer	Smoking	Number	1998	544.434771	1072.546082	211.1780121
YLLs (Years of Life Lost)	Global	Both	95+ years	Prostate cancer	Smoking	Rate	1998	35.04322374	69.03576759	13.5927364
YLLs (Years of Life Lost)	Global	Both	95+ years	Prostate cancer	Smoking	Number	1999	565.2099217	1119.264181	218.5075428
YLLs (Years of Life Lost)	Global	Both	95+ years	Prostate cancer	Smoking	Rate	1999	33.9968758	67.32274843	13.14303502
YLLs (Years of Life Lost)	Global	Both	95+ years	Prostate cancer	Smoking	Number	2000	583.2089097	1153.159936	225.2130245
YLLs (Years of Life Lost)	Global	Both	95+ years	Prostate cancer	Smoking	Rate	2000	32.70847715	64.67340397	12.63076565
YLLs (Years of Life Lost)	Global	Both	95+ years	Prostate cancer	Smoking	Number	2001	613.3926304	1213.102169	236.1469063

YLLs (Years of Life Lost)	Global	Both	95+ years	Prostate cancer	Smoking	Rate	2001	32.13540174	63.55395159	12.37164473
YLLs (Years of Life Lost)	Global	Both	95+ years	Prostate cancer	Smoking	Number	2002	636.6785821	1270.279461	244.3569261
YLLs (Years of Life Lost)	Global	Both	95+ years	Prostate cancer	Smoking	Rate	2002	31.20545802	62.26006892	11.97663941
YLLs (Years of Life Lost)	Global	Both	95+ years	Prostate cancer	Smoking	Number	2003	662.9401298	1335.165352	254.2380356
YLLs (Years of Life Lost)	Global	Both	95+ years	Prostate cancer	Smoking	Rate	2003	30.66735614	61.76423709	11.76095402
YLLs (Years of Life Lost)	Global	Both	95+ years	Prostate cancer	Smoking	Number	2004	682.1745614	1375.239969	259.5313233
YLLs (Years of Life Lost)	Global	Both	95+ years	Prostate cancer	Smoking	Rate	2004	29.84778904	60.17209496	11.35550433
YLLs (Years of Life Lost)	Global	Both	95+ years	Prostate cancer	Smoking	Number	2005	691.9344651	1402.227738	263.1623145
YLLs (Years of Life Lost)	Global	Both	95+ years	Prostate cancer	Smoking	Rate	2005	28.68981185	58.14083848	10.91154968
YLLs (Years of Life Lost)	Global	Both	95+ years	Prostate cancer	Smoking	Number	2006	709.4982363	1442.282233	267.2431322
YLLs (Years of Life Lost)	Global	Both	95+ years	Prostate cancer	Smoking	Rate	2006	27.88231654	56.67973182	10.50229193

YLLs (Years of Life Lost)	Global	Both	95+ years	Prostate cancer	Smoking	Number	2007	728.8111644	1484.962594	271.5431805
YLLs (Years of Life Lost)	Global	Both	95+ years	Prostate cancer	Smoking	Rate	2007	27.18918213	55.39832594	10.13024684
YLLs (Years of Life Lost)	Global	Both	95+ years	Prostate cancer	Smoking	Number	2008	754.3697183	1545.47467	279.9477501
YLLs (Years of Life Lost)	Global	Both	95+ years	Prostate cancer	Smoking	Rate	2008	26.70245928	54.70523731	9.909323262
YLLs (Years of Life Lost)	Global	Both	95+ years	Prostate cancer	Smoking	Number	2009	767.1369386	1578.921433	282.1623948
YLLs (Years of Life Lost)	Global	Both	95+ years	Prostate cancer	Smoking	Rate	2009	25.75773324	53.01457281	9.474010871
YLLs (Years of Life Lost)	Global	Both	95+ years	Prostate cancer	Smoking	Number	2010	780.9398392	1628.683061	282.7022361
YLLs (Years of Life Lost)	Global	Both	95+ years	Prostate cancer	Smoking	Rate	2010	24.97110005	52.07828519	9.039602627
YLLs (Years of Life Lost)	Global	Both	95+ years	Prostate cancer	Smoking	Number	2011	785.7811667	1646.820842	281.6403053
YLLs (Years of Life Lost)	Global	Both	95+ years	Prostate cancer	Smoking	Rate	2011	24.1430058	50.59831773	8.653355175
YLLs (Years of Life Lost)	Global	Both	95+ years	Prostate cancer	Smoking	Number	2012	780.6833236	1640.395519	279.5310096

YLLs (Years of Life Lost)	Global	Both	95+ years	Prostate cancer	Smoking	Rate	2012	23.12696506	48.59508166	8.280827447
YLLs (Years of Life Lost)	Global	Both	95+ years	Prostate cancer	Smoking	Number	2013	790.5027654	1682.537535	280.3068624
YLLs (Years of Life Lost)	Global	Both	95+ years	Prostate cancer	Smoking	Rate	2013	22.59965773	48.10201059	8.013683729
YLLs (Years of Life Lost)	Global	Both	95+ years	Prostate cancer	Smoking	Number	2014	821.6806623	1761.580847	290.05640 9
YLLs (Years of Life Lost)	Global	Both	95+ years	Prostate cancer	Smoking	Rate	2014	22.5358344	48.31401795	7.955235533
YLLs (Years of Life Lost)	Global	Both	95+ years	Prostate cancer	Smoking	Number	2015	864.9635519	1861.770827	306.4613113
YLLs (Years of Life Lost)	Global	Both	95+ years	Prostate cancer	Smoking	Rate	2015	22.32231532	48.04715222	7.908918252
YLLs (Years of Life Lost)	Global	Both	95+ years	Prostate cancer	Smoking	Number	2016	926.6588528	2006.931046	326.8764497
YLLs (Years of Life Lost)	Global	Both	95+ years	Prostate cancer	Smoking	Rate	2016	22.42906497	48.57622274	7.911793112
YLLs (Years of Life Lost)	Global	Both	95+ years	Prostate cancer	Smoking	Number	2017	1000.373011	2177.359251	351.4908838
YLLs (Years of Life Lost)	Global	Both	95+ years	Prostate cancer	Smoking	Rate	2017	22.75171096	49.52017678	7.994037133

YLLs (Years of Life Lost)	Global	Both	95+ years	Prostate cancer	Smoking	Number	2018	1070.84114	2352.172758	373.354037 1
YLLs (Years of Life Lost)	Global	Both	95+ years	Prostate cancer	Smoking	Rate	2018	22.94880033	50.40854424	8.00121222 55
YLLs (Years of Life Lost)	Global	Both	95+ years	Prostate cancer	Smoking	Number	2019	1132.305951	2505.757223	394.247119 4
YLLs (Years of Life Lost)	Global	Both	95+ years	Prostate cancer	Smoking	Rate	2019	22.86551411	50.60065887	7.96133153 4
YLLs (Years of Life Lost)	Global	Both	95+ years	Prostate cancer	Smoking	Number	2020	1180.959349	2623.285493	416.367993 8
YLLs (Years of Life Lost)	Global	Both	95+ years	Prostate cancer	Smoking	Rate	2020	22.58903631	50.1774183	7.96416213 6
YLLs (Years of Life Lost)	Global	Both	95+ years	Prostate cancer	Smoking	Number	2021	1196.873584	2672.152545	422.567438 3
YLLs (Years of Life Lost)	Global	Both	95+ years	Prostate cancer	Smoking	Rate	2021	21.95973945	49.02754512	7.75309186 1
YLLs (Years of Life Lost)	Global	Both	30-34 years	Prostate cancer	Smoking	Number	1990	451.8354559	732.569695	162.505773 9
YLLs (Years of Life Lost)	Global	Both	30-34 years	Prostate cancer	Smoking	Rate	1990	0.117231213	0.190069267	0.04216302 4
YLLs (Years of Life Lost)	Global	Both	30-34 years	Prostate cancer	Smoking	Number	1991	451.9700825	740.4957049	163.737899 9

YLLs (Years of Life Lost)	Global	Both	30-34 years	Prostate cancer	Smoking	Rate	1991	0.115723203	0.189597803	0.041923735
YLLs (Years of Life Lost)	Global	Both	30-34 years	Prostate cancer	Smoking	Number	1992	450.8013204	738.4371648	166.697237 2
YLLs (Years of Life Lost)	Global	Both	30-34 years	Prostate cancer	Smoking	Rate	1992	0.113006834	0.185111362	0.041787648
YLLs (Years of Life Lost)	Global	Both	30-34 years	Prostate cancer	Smoking	Number	1993	460.6678569	750.9880242	169.499429 2
YLLs (Years of Life Lost)	Global	Both	30-34 years	Prostate cancer	Smoking	Rate	1993	0.11199246	0.18257187 9	0.041206821
YLLs (Years of Life Lost)	Global	Both	30-34 years	Prostate cancer	Smoking	Number	1994	470.6549539	776.3087414	170.80601
YLLs (Years of Life Lost)	Global	Both	30-34 years	Prostate cancer	Smoking	Rate	1994	0.110572633	0.182380958	0.04012806
YLLs (Years of Life Lost)	Global	Both	30-34 years	Prostate cancer	Smoking	Number	1995	470.6500434	771.1941222	165.818997 1
YLLs (Years of Life Lost)	Global	Both	30-34 years	Prostate cancer	Smoking	Rate	1995	0.107027506	0.175372307	0.037707834
YLLs (Years of Life Lost)	Global	Both	30-34 years	Prostate cancer	Smoking	Number	1996	474.6162964	786.7566156	171.156730 1
YLLs (Years of Life Lost)	Global	Both	30-34 years	Prostate cancer	Smoking	Rate	1996	0.105000601	0.174056218	0.03786545

YLLs (Years of Life Lost)	Global	Both	30-34 years	Prostate cancer	Smoking	Number	1997	477.206163	782.5769353	167.5146426
YLLs (Years of Life Lost)	Global	Both	30-34 years	Prostate cancer	Smoking	Rate	1997	0.103229963	0.169288232	0.036237022
YLLs (Years of Life Lost)	Global	Both	30-34 years	Prostate cancer	Smoking	Number	1998	463.8927957	761.6262215	165.5234952
YLLs (Years of Life Lost)	Global	Both	30-34 years	Prostate cancer	Smoking	Rate	1998	0.098820155	0.162244428	0.035260426
YLLs (Years of Life Lost)	Global	Both	30-34 years	Prostate cancer	Smoking	Number	1999	450.941167	740.7404439	158.7136913
YLLs (Years of Life Lost)	Global	Both	30-34 years	Prostate cancer	Smoking	Rate	1999	0.094999048	0.156050594	0.033435957
YLLs (Years of Life Lost)	Global	Both	30-34 years	Prostate cancer	Smoking	Number	2000	445.2131989	736.4425942	160.5408679
YLLs (Years of Life Lost)	Global	Both	30-34 years	Prostate cancer	Smoking	Rate	2000	0.092926879	0.153713573	0.033508804
YLLs (Years of Life Lost)	Global	Both	30-34 years	Prostate cancer	Smoking	Number	2001	432.8477553	707.5093893	161.6376789
YLLs (Years of Life Lost)	Global	Both	30-34 years	Prostate cancer	Smoking	Rate	2001	0.089422435	0.146165047	0.033392884
YLLs (Years of Life Lost)	Global	Both	30-34 years	Prostate cancer	Smoking	Number	2002	433.7338512	707.936101	160.7144181

YLLs (Years of Life Lost)	Global	Both	30-34 years	Prostate cancer	Smoking	Rate	2002	0.088793341	0.144927612	0.032901214
YLLs (Years of Life Lost)	Global	Both	30-34 years	Prostate cancer	Smoking	Number	2003	439.5699089	710.1705263	166.9696519
YLLs (Years of Life Lost)	Global	Both	30-34 years	Prostate cancer	Smoking	Rate	2003	0.089283484	0.144246678	0.033914133
YLLs (Years of Life Lost)	Global	Both	30-34 years	Prostate cancer	Smoking	Number	2004	432.4269533	689.9574189	168.6252123
YLLs (Years of Life Lost)	Global	Both	30-34 years	Prostate cancer	Smoking	Rate	2004	0.087311596	0.139309733	0.034047222
YLLs (Years of Life Lost)	Global	Both	30-34 years	Prostate cancer	Smoking	Number	2005	433.8718407	678.0136497	175.0273653
YLLs (Years of Life Lost)	Global	Both	30-34 years	Prostate cancer	Smoking	Rate	2005	0.08728472	0.136400259	0.035211353
YLLs (Years of Life Lost)	Global	Both	30-34 years	Prostate cancer	Smoking	Number	2006	424.868629	672.2477251	176.1009852
YLLs (Years of Life Lost)	Global	Both	30-34 years	Prostate cancer	Smoking	Rate	2006	0.085344552	0.135036284	0.035373898
YLLs (Years of Life Lost)	Global	Both	30-34 years	Prostate cancer	Smoking	Number	2007	415.3311184	655.9492389	174.2879928
YLLs (Years of Life Lost)	Global	Both	30-34 years	Prostate cancer	Smoking	Rate	2007	0.083352392	0.131641805	0.034977685

YLLs (Years of Life Lost)	Global	Both	30-34 years	Prostate cancer	Smoking	Number	2008	404.7821157	633.0181019	171.7884814
YLLs (Years of Life Lost)	Global	Both	30-34 years	Prostate cancer	Smoking	Rate	2008	0.081001142	0.126673554	0.034376675
YLLs (Years of Life Lost)	Global	Both	30-34 years	Prostate cancer	Smoking	Number	2009	393.4051486	617.5092188	163.6496028
YLLs (Years of Life Lost)	Global	Both	30-34 years	Prostate cancer	Smoking	Rate	2009	0.078196436	0.1227412	0.032528338
YLLs (Years of Life Lost)	Global	Both	30-34 years	Prostate cancer	Smoking	Number	2010	391.7359855	619.2092553	162.1329425
YLLs (Years of Life Lost)	Global	Both	30-34 years	Prostate cancer	Smoking	Rate	2010	0.077073798	0.121829015	0.03189955
YLLs (Years of Life Lost)	Global	Both	30-34 years	Prostate cancer	Smoking	Number	2011	394.2521706	625.8050525	165.5438129
YLLs (Years of Life Lost)	Global	Both	30-34 years	Prostate cancer	Smoking	Rate	2011	0.076633461	0.121641961	0.032177871
YLLs (Years of Life Lost)	Global	Both	30-34 years	Prostate cancer	Smoking	Number	2012	386.7080985	617.7184732	157.0065176
YLLs (Years of Life Lost)	Global	Both	30-34 years	Prostate cancer	Smoking	Rate	2012	0.074092652	0.118353869	0.030082197
YLLs (Years of Life Lost)	Global	Both	30-34 years	Prostate cancer	Smoking	Number	2013	367.438781	588.4996034	149.3468359

YLLs (Years of Life Lost)	Global	Both	30-34 years	Prostate cancer	Smoking	Rate	2013	0.069216962	0.110859705	0.028133487
YLLs (Years of Life Lost)	Global	Both	30-34 years	Prostate cancer	Smoking	Number	2014	382.3989696	611.0259201	155.850138
YLLs (Years of Life Lost)	Global	Both	30-34 years	Prostate cancer	Smoking	Rate	2014	0.070672247	0.112925448	0.02880311
YLLs (Years of Life Lost)	Global	Both	30-34 years	Prostate cancer	Smoking	Number	2015	374.6665125	605.9093203	151.5668058
YLLs (Years of Life Lost)	Global	Both	30-34 years	Prostate cancer	Smoking	Rate	2015	0.067794629	0.109637227	0.027425497
YLLs (Years of Life Lost)	Global	Both	30-34 years	Prostate cancer	Smoking	Number	2016	380.538417	616.237852	153.5088981
YLLs (Years of Life Lost)	Global	Both	30-34 years	Prostate cancer	Smoking	Rate	2016	0.067268145	0.108932963	0.027135917
YLLs (Years of Life Lost)	Global	Both	30-34 years	Prostate cancer	Smoking	Number	2017	395.4350244	648.9621474	154.9415978
YLLs (Years of Life Lost)	Global	Both	30-34 years	Prostate cancer	Smoking	Rate	2017	0.068317741	0.112118617	0.026768646
YLLs (Years of Life Lost)	Global	Both	30-34 years	Prostate cancer	Smoking	Number	2018	410.6387088	679.4843576	158.3767586
YLLs (Years of Life Lost)	Global	Both	30-34 years	Prostate cancer	Smoking	Rate	2018	0.069559573	0.115100308	0.026828011

YLLs (Years of Life Lost)	Global	Both	30-34 years	Prostate cancer	Smoking	Number	2019	416.9664506	695.5251782	166.993821 1
YLLs (Years of Life Lost)	Global	Both	30-34 years	Prostate cancer	Smoking	Rate	2019	0.069603152	0.116102254	0.027875855
YLLs (Years of Life Lost)	Global	Both	30-34 years	Prostate cancer	Smoking	Number	2020	406.4295306	654.670716	159.8255176
YLLs (Years of Life Lost)	Global	Both	30-34 years	Prostate cancer	Smoking	Rate	2020	0.067285765	0.108382922	0.026459648
YLLs (Years of Life Lost)	Global	Both	30-34 years	Prostate cancer	Smoking	Number	2021	404.8474519	649.8543639	163.4431411
YLLs (Years of Life Lost)	Global	Both	30-34 years	Prostate cancer	Smoking	Rate	2021	0.06697448	0.107506315	0.027038627
YLLs (Years of Life Lost)	Global	Both	35-39 years	Prostate cancer	Smoking	Number	1990	951.6008706	1638.218101	313.7034008
YLLs (Years of Life Lost)	Global	Both	35-39 years	Prostate cancer	Smoking	Rate	1990	0.270153529	0.465079862	0.089058431
YLLs (Years of Life Lost)	Global	Both	35-39 years	Prostate cancer	Smoking	Number	1991	986.5204598	1672.401228	322.1035929
YLLs (Years of Life Lost)	Global	Both	35-39 years	Prostate cancer	Smoking	Rate	1991	0.273205095	0.463151607	0.089202755
YLLs (Years of Life Lost)	Global	Both	35-39 years	Prostate cancer	Smoking	Number	1992	1010.658482	1747.759341	348.8631474

YLLs (Years of Life Lost)	Global	Both	35-39 years	Prostate cancer	Smoking	Rate	1992	0.274341141	0.474425635	0.094698175
YLLs (Years of Life Lost)	Global	Both	35-39 years	Prostate cancer	Smoking	Number	1993	1024.884551	1752.542095	347.2132084
YLLs (Years of Life Lost)	Global	Both	35-39 years	Prostate cancer	Smoking	Rate	1993	0.274539943	0.469460494	0.093009397
YLLs (Years of Life Lost)	Global	Both	35-39 years	Prostate cancer	Smoking	Number	1994	1006.563864	1676.031621	360.7564342
YLLs (Years of Life Lost)	Global	Both	35-39 years	Prostate cancer	Smoking	Rate	1994	0.267366333	0.445192248	0.095825142
YLLs (Years of Life Lost)	Global	Both	35-39 years	Prostate cancer	Smoking	Number	1995	980.8281532	1636.222615	341.2941934
YLLs (Years of Life Lost)	Global	Both	35-39 years	Prostate cancer	Smoking	Rate	1995	0.258451124	0.431149506	0.089932031
YLLs (Years of Life Lost)	Global	Both	35-39 years	Prostate cancer	Smoking	Number	1996	962.8890672	1616.978901	347.2697712
YLLs (Years of Life Lost)	Global	Both	35-39 years	Prostate cancer	Smoking	Rate	1996	0.250371185	0.420448146	0.090297363
YLLs (Years of Life Lost)	Global	Both	35-39 years	Prostate cancer	Smoking	Number	1997	958.4611543	1589.407219	338.7041676
YLLs (Years of Life Lost)	Global	Both	35-39 years	Prostate cancer	Smoking	Rate	1997	0.243866688	0.404401861	0.086178416

YLLs (Years of Life Lost)	Global	Both	35-39 years	Prostate cancer	Smoking	Number	1998	946.0287032	1579.04845	332.5088458
YLLs (Years of Life Lost)	Global	Both	35-39 years	Prostate cancer	Smoking	Rate	1998	0.233252812	0.389330144	0.081983372
YLLs (Years of Life Lost)	Global	Both	35-39 years	Prostate cancer	Smoking	Number	1999	937.8824183	1561.896827	319.0042473
YLLs (Years of Life Lost)	Global	Both	35-39 years	Prostate cancer	Smoking	Rate	1999	0.223264921	0.37181289	0.075939645
YLLs (Years of Life Lost)	Global	Both	35-39 years	Prostate cancer	Smoking	Number	2000	940.3079633	1598.674092	327.8950822
YLLs (Years of Life Lost)	Global	Both	35-39 years	Prostate cancer	Smoking	Rate	2000	0.216482683	0.368055223	0.075489744
YLLs (Years of Life Lost)	Global	Both	35-39 years	Prostate cancer	Smoking	Number	2001	923.0257336	1551.4111	332.0840445
YLLs (Years of Life Lost)	Global	Both	35-39 years	Prostate cancer	Smoking	Rate	2001	0.206655177	0.347343658	0.074349917
YLLs (Years of Life Lost)	Global	Both	35-39 years	Prostate cancer	Smoking	Number	2002	921.1274647	1517.534885	342.1553694
YLLs (Years of Life Lost)	Global	Both	35-39 years	Prostate cancer	Smoking	Rate	2002	0.20157386	0.332087985	0.074875173
YLLs (Years of Life Lost)	Global	Both	35-39 years	Prostate cancer	Smoking	Number	2003	879.4971874	1441.992681	347.6763106

YLLs (Years of Life Lost)	Global	Both	35-39 years	Prostate cancer	Smoking	Rate	2003	0.189414513	0.31055738	0.074877942
YLLs (Years of Life Lost)	Global	Both	35-39 years	Prostate cancer	Smoking	Number	2004	845.8322988	1372.999022	344.037429
YLLs (Years of Life Lost)	Global	Both	35-39 years	Prostate cancer	Smoking	Rate	2004	0.179981705	0.292155673	0.073206525
YLLs (Years of Life Lost)	Global	Both	35-39 years	Prostate cancer	Smoking	Number	2005	830.9957881	1335.115189	339.9145439
YLLs (Years of Life Lost)	Global	Both	35-39 years	Prostate cancer	Smoking	Rate	2005	0.17500669	0.281173615	0.071585584
YLLs (Years of Life Lost)	Global	Both	35-39 years	Prostate cancer	Smoking	Number	2006	815.4024139	1289.69057	335.5424406
YLLs (Years of Life Lost)	Global	Both	35-39 years	Prostate cancer	Smoking	Rate	2006	0.169784048	0.268540885	0.069867041
YLLs (Years of Life Lost)	Global	Both	35-39 years	Prostate cancer	Smoking	Number	2007	796.5001149	1259.405622	333.1865506
YLLs (Years of Life Lost)	Global	Both	35-39 years	Prostate cancer	Smoking	Rate	2007	0.16418248	0.25960114	0.068679706
YLLs (Years of Life Lost)	Global	Both	35-39 years	Prostate cancer	Smoking	Number	2008	774.0087524	1204.580821	331.9947973
YLLs (Years of Life Lost)	Global	Both	35-39 years	Prostate cancer	Smoking	Rate	2008	0.158138999	0.246109885	0.067830402

YLLs (Years of Life Lost)	Global	Both	35-39 years	Prostate cancer	Smoking	Number	2009	744.4323209	1163.242696	315.786159 2
YLLs (Years of Life Lost)	Global	Both	35-39 years	Prostate cancer	Smoking	Rate	2009	0.151061298	0.236046913	0.0640797 9
YLLs (Years of Life Lost)	Global	Both	35-39 years	Prostate cancer	Smoking	Number	2010	726.2842412	1131.638862	302.574278 4
YLLs (Years of Life Lost)	Global	Both	35-39 years	Prostate cancer	Smoking	Rate	2010	0.146797679	0.228728573	0.061156775
YLLs (Years of Life Lost)	Global	Both	35-39 years	Prostate cancer	Smoking	Number	2011	713.3083424	1108.451182	307.396484 7
YLLs (Years of Life Lost)	Global	Both	35-39 years	Prostate cancer	Smoking	Rate	2011	0.144001377	0.223772087	0.062056637
YLLs (Years of Life Lost)	Global	Both	35-39 years	Prostate cancer	Smoking	Number	2012	694.5574153	1071.846686	292.386794 5
YLLs (Years of Life Lost)	Global	Both	35-39 years	Prostate cancer	Smoking	Rate	2012	0.140158141	0.21629319	0.059002163
YLLs (Years of Life Lost)	Global	Both	35-39 years	Prostate cancer	Smoking	Number	2013	671.0008455	1049.131155	285.439706 3
YLLs (Years of Life Lost)	Global	Both	35-39 years	Prostate cancer	Smoking	Rate	2013	0.135136775	0.211290644	0.057486368
YLLs (Years of Life Lost)	Global	Both	35-39 years	Prostate cancer	Smoking	Number	2014	691.5164863	1067.080447	299.649155

YLLs (Years of Life Lost)	Global	Both	35-39 years	Prostate cancer	Smoking	Rate	2014	0.138488782	0.213702312	0.060010206
YLLs (Years of Life Lost)	Global	Both	35-39 years	Prostate cancer	Smoking	Number	2015	683.0734726	1058.99523	291.579883 1
YLLs (Years of Life Lost)	Global	Both	35-39 years	Prostate cancer	Smoking	Rate	2015	0.135464155	0.21001532	0.057824852
YLLs (Years of Life Lost)	Global	Both	35-39 years	Prostate cancer	Smoking	Number	2016	683.4770007	1068.605349	282.07779 8
YLLs (Years of Life Lost)	Global	Both	35-39 years	Prostate cancer	Smoking	Rate	2016	0.133902911	0.209355057	0.055263071
YLLs (Years of Life Lost)	Global	Both	35-39 years	Prostate cancer	Smoking	Number	2017	693.4300441	1116.181114	294.4726852
YLLs (Years of Life Lost)	Global	Both	35-39 years	Prostate cancer	Smoking	Rate	2017	0.133923041	0.215569502	0.056871891
YLLs (Years of Life Lost)	Global	Both	35-39 years	Prostate cancer	Smoking	Number	2018	712.1811938	1110.724234	297.0702079
YLLs (Years of Life Lost)	Global	Both	35-39 years	Prostate cancer	Smoking	Rate	2018	0.135250152	0.210937361	0.056416529
YLLs (Years of Life Lost)	Global	Both	35-39 years	Prostate cancer	Smoking	Number	2019	720.5726431	1161.97566	304.0187686
YLLs (Years of Life Lost)	Global	Both	35-39 years	Prostate cancer	Smoking	Rate	2019	0.13426178	0.21650686	0.056646754

YLLs (Years of Life Lost)	Global	Both	35-39 years	Prostate cancer	Smoking	Number	2020	712.2166854	1123.924185	288.4776588
YLLs (Years of Life Lost)	Global	Both	35-39 years	Prostate cancer	Smoking	Rate	2020	0.129947924	0.205066264	0.052634365
YLLs (Years of Life Lost)	Global	Both	35-39 years	Prostate cancer	Smoking	Number	2021	734.1431968	1175.527515	301.0102219
YLLs (Years of Life Lost)	Global	Both	35-39 years	Prostate cancer	Smoking	Rate	2021	0.130894556	0.20959147	0.05366882
YLLs (Years of Life Lost)	Global	Both	40-44 years	Prostate cancer	Smoking	Number	1990	1233.376823	1961.753983	517.0705834
YLLs (Years of Life Lost)	Global	Both	40-44 years	Prostate cancer	Smoking	Rate	1990	0.430525616	0.684774779	0.180489958
YLLs (Years of Life Lost)	Global	Both	40-44 years	Prostate cancer	Smoking	Number	1991	1320.269176	2122.894989	556.3218222
YLLs (Years of Life Lost)	Global	Both	40-44 years	Prostate cancer	Smoking	Rate	1991	0.439314128	0.706384559	0.185113794
YLLs (Years of Life Lost)	Global	Both	40-44 years	Prostate cancer	Smoking	Number	1992	1373.164218	2209.644327	566.7142954
YLLs (Years of Life Lost)	Global	Both	40-44 years	Prostate cancer	Smoking	Rate	1992	0.441948086	0.711166274	0.182395008
YLLs (Years of Life Lost)	Global	Both	40-44 years	Prostate cancer	Smoking	Number	1993	1431.787539	2299.660154	602.3399607

YLLs (Years of Life Lost)	Global	Both	40-44 years	Prostate cancer	Smoking	Rate	1993	0.44484005	0.714478168	0.187140152
YLLs (Years of Life Lost)	Global	Both	40-44 years	Prostate cancer	Smoking	Number	1994	1463.633484	2325.785387	613.7281483
YLLs (Years of Life Lost)	Global	Both	40-44 years	Prostate cancer	Smoking	Rate	1994	0.442427411	0.703038854	0.185517863
YLLs (Years of Life Lost)	Global	Both	40-44 years	Prostate cancer	Smoking	Number	1995	1507.472361	2398.769885	641.3181881
YLLs (Years of Life Lost)	Global	Both	40-44 years	Prostate cancer	Smoking	Rate	1995	0.439011779	0.698578801	0.186767098
YLLs (Years of Life Lost)	Global	Both	40-44 years	Prostate cancer	Smoking	Number	1996	1530.100626	2436.942919	635.9712036
YLLs (Years of Life Lost)	Global	Both	40-44 years	Prostate cancer	Smoking	Rate	1996	0.435789994	0.694068954	0.181131804
YLLs (Years of Life Lost)	Global	Both	40-44 years	Prostate cancer	Smoking	Number	1997	1531.400736	2433.483708	655.3687976
YLLs (Years of Life Lost)	Global	Both	40-44 years	Prostate cancer	Smoking	Rate	1997	0.42834563	0.680665803	0.183312149
YLLs (Years of Life Lost)	Global	Both	40-44 years	Prostate cancer	Smoking	Number	1998	1538.061609	2447.638304	646.6113485
YLLs (Years of Life Lost)	Global	Both	40-44 years	Prostate cancer	Smoking	Rate	1998	0.425055982	0.676424987	0.178696367

YLLs (Years of Life Lost)	Global	Both	40-44 years	Prostate cancer	Smoking	Number	1999	1534.852433	2402.777623	640.544013 5
YLLs (Years of Life Lost)	Global	Both	40-44 years	Prostate cancer	Smoking	Rate	1999	0.420761824	0.658693354	0.175597642
YLLs (Years of Life Lost)	Global	Both	40-44 years	Prostate cancer	Smoking	Number	2000	1517.658332	2421.163414	646.586325 6
YLLs (Years of Life Lost)	Global	Both	40-44 years	Prostate cancer	Smoking	Rate	2000	0.41259498	0.658224482	0.175782827
YLLs (Years of Life Lost)	Global	Both	40-44 years	Prostate cancer	Smoking	Number	2001	1491.492056	2374.325138	623.801307 7
YLLs (Years of Life Lost)	Global	Both	40-44 years	Prostate cancer	Smoking	Rate	2001	0.399831946	0.63649755	0.167225625
YLLs (Years of Life Lost)	Global	Both	40-44 years	Prostate cancer	Smoking	Number	2002	1471.891418	2302.23244	627.037823 4
YLLs (Years of Life Lost)	Global	Both	40-44 years	Prostate cancer	Smoking	Rate	2002	0.385760426	0.603380219	0.164337107
YLLs (Years of Life Lost)	Global	Both	40-44 years	Prostate cancer	Smoking	Number	2003	1446.912224	2232.528068	614.040958 2
YLLs (Years of Life Lost)	Global	Both	40-44 years	Prostate cancer	Smoking	Rate	2003	0.367131584	0.566469446	0.15580339
YLLs (Years of Life Lost)	Global	Both	40-44 years	Prostate cancer	Smoking	Number	2004	1464.230991	2286.563381	631.627711 2

YLLs (Years of Life Lost)	Global	Both	40-44 years	Prostate cancer	Smoking	Rate	2004	0.358308747	0.559539899	0.154564229
YLLs (Years of Life Lost)	Global	Both	40-44 years	Prostate cancer	Smoking	Number	2005	1507.916878	2341.511833	656.2850008
YLLs (Years of Life Lost)	Global	Both	40-44 years	Prostate cancer	Smoking	Rate	2005	0.356441691	0.553487032	0.155132779
YLLs (Years of Life Lost)	Global	Both	40-44 years	Prostate cancer	Smoking	Number	2006	1518.149602	2364.899091	660.4787264
YLLs (Years of Life Lost)	Global	Both	40-44 years	Prostate cancer	Smoking	Rate	2006	0.348610798	0.543048826	0.151664906
YLLs (Years of Life Lost)	Global	Both	40-44 years	Prostate cancer	Smoking	Number	2007	1489.208039	2335.429789	669.4579719
YLLs (Years of Life Lost)	Global	Both	40-44 years	Prostate cancer	Smoking	Rate	2007	0.333864156	0.523577819	0.150085156
YLLs (Years of Life Lost)	Global	Both	40-44 years	Prostate cancer	Smoking	Number	2008	1445.284077	2238.583281	637.0788659
YLLs (Years of Life Lost)	Global	Both	40-44 years	Prostate cancer	Smoking	Rate	2008	0.318515239	0.493344458	0.140400999
YLLs (Years of Life Lost)	Global	Both	40-44 years	Prostate cancer	Smoking	Number	2009	1392.239503	2151.826217	607.7449958
YLLs (Years of Life Lost)	Global	Both	40-44 years	Prostate cancer	Smoking	Rate	2009	0.302835436	0.468058283	0.132194727

YLLs (Years of Life Lost)	Global	Both	40-44 years	Prostate cancer	Smoking	Number	2010	1366.354059	2104.497031	595.2012758
YLLs (Years of Life Lost)	Global	Both	40-44 years	Prostate cancer	Smoking	Rate	2010	0.293951952	0.452753081	0.128049224
YLLs (Years of Life Lost)	Global	Both	40-44 years	Prostate cancer	Smoking	Number	2011	1361.576411	2107.9905	598.0360064
YLLs (Years of Life Lost)	Global	Both	40-44 years	Prostate cancer	Smoking	Rate	2011	0.289508824	0.448217115	0.127159005
YLLs (Years of Life Lost)	Global	Both	40-44 years	Prostate cancer	Smoking	Number	2012	1339.457738	2080.363126	582.7082963
YLLs (Years of Life Lost)	Global	Both	40-44 years	Prostate cancer	Smoking	Rate	2012	0.281881494	0.437800947	0.122627748
YLLs (Years of Life Lost)	Global	Both	40-44 years	Prostate cancer	Smoking	Number	2013	1324.02218	2074.554547	576.4546437
YLLs (Years of Life Lost)	Global	Both	40-44 years	Prostate cancer	Smoking	Rate	2013	0.276183093	0.432739648	0.120244985
YLLs (Years of Life Lost)	Global	Both	40-44 years	Prostate cancer	Smoking	Number	2014	1346.328127	2070.654224	579.2501757
YLLs (Years of Life Lost)	Global	Both	40-44 years	Prostate cancer	Smoking	Rate	2014	0.278994521	0.429093897	0.120035838
YLLs (Years of Life Lost)	Global	Both	40-44 years	Prostate cancer	Smoking	Number	2015	1329.365064	2076.074759	579.3700529

YLLs (Years of Life Lost)	Global	Both	40-44 years	Prostate cancer	Smoking	Rate	2015	0.274400792	0.42853282	0.119590627
YLLs (Years of Life Lost)	Global	Both	40-44 years	Prostate cancer	Smoking	Number	2016	1312.161055	2020.811584	552.8359433
YLLs (Years of Life Lost)	Global	Both	40-44 years	Prostate cancer	Smoking	Rate	2016	0.270481877	0.416559316	0.113958651
YLLs (Years of Life Lost)	Global	Both	40-44 years	Prostate cancer	Smoking	Number	2017	1289.861882	2061.281857	532.7880841
YLLs (Years of Life Lost)	Global	Both	40-44 years	Prostate cancer	Smoking	Rate	2017	0.265746662	0.424680177	0.109768849
YLLs (Years of Life Lost)	Global	Both	40-44 years	Prostate cancer	Smoking	Number	2018	1286.806583	1972.595495	540.2432054
YLLs (Years of Life Lost)	Global	Both	40-44 years	Prostate cancer	Smoking	Rate	2018	0.264566652	0.405564436	0.111073675
YLLs (Years of Life Lost)	Global	Both	40-44 years	Prostate cancer	Smoking	Number	2019	1288.874583	2009.203602	543.3570081
YLLs (Years of Life Lost)	Global	Both	40-44 years	Prostate cancer	Smoking	Rate	2019	0.263440602	0.410672856	0.111059912
YLLs (Years of Life Lost)	Global	Both	40-44 years	Prostate cancer	Smoking	Number	2020	1265.697638	1969.193778	541.9606786
YLLs (Years of Life Lost)	Global	Both	40-44 years	Prostate cancer	Smoking	Rate	2020	0.256114469	0.39846722	0.10966598

YLLs (Years of Life Lost)	Global	Both	40-44 years	Prostate cancer	Smoking	Number	2021	1273.637848	1979.138872	537.7899931
YLLs (Years of Life Lost)	Global	Both	40-44 years	Prostate cancer	Smoking	Rate	2021	0.254599865	0.39562933	0.107504076
YLLs (Years of Life Lost)	Global	Both	45-49 years	Prostate cancer	Smoking	Number	1990	3083.831683	4781.988252	1387.839739
YLLs (Years of Life Lost)	Global	Both	45-49 years	Prostate cancer	Smoking	Rate	1990	1.328118669	2.059466444	0.597703136
YLLs (Years of Life Lost)	Global	Both	45-49 years	Prostate cancer	Smoking	Number	1991	3119.885476	4797.163924	1386.063442
YLLs (Years of Life Lost)	Global	Both	45-49 years	Prostate cancer	Smoking	Rate	1991	1.325420896	2.037979072	0.58884131
YLLs (Years of Life Lost)	Global	Both	45-49 years	Prostate cancer	Smoking	Number	1992	3244.01946	4999.288042	1458.739583
YLLs (Years of Life Lost)	Global	Both	45-49 years	Prostate cancer	Smoking	Rate	1992	1.3317454	2.052323956	0.598846509
YLLs (Years of Life Lost)	Global	Both	45-49 years	Prostate cancer	Smoking	Number	1993	3386.733302	5209.408626	1519.708072
YLLs (Years of Life Lost)	Global	Both	45-49 years	Prostate cancer	Smoking	Rate	1993	1.340996307	2.062694964	0.601737051
YLLs (Years of Life Lost)	Global	Both	45-49 years	Prostate cancer	Smoking	Number	1994	3614.585655	5538.960797	1645.96043

YLLs (Years of Life Lost)	Global	Both	45-49 years	Prostate cancer	Smoking	Rate	1994	1.362807812	2.088355282	0.620576726
YLLs (Years of Life Lost)	Global	Both	45-49 years	Prostate cancer	Smoking	Number	1995	3778.93769	5792.445809	1690.521694
YLLs (Years of Life Lost)	Global	Both	45-49 years	Prostate cancer	Smoking	Rate	1995	1.371205741	2.10181686	0.613413939
YLLs (Years of Life Lost)	Global	Both	45-49 years	Prostate cancer	Smoking	Number	1996	3987.598726	6119.829799	1809.617815
YLLs (Years of Life Lost)	Global	Both	45-49 years	Prostate cancer	Smoking	Rate	1996	1.381205487	2.119757548	0.626806815
YLLs (Years of Life Lost)	Global	Both	45-49 years	Prostate cancer	Smoking	Number	1997	4067.462396	6233.698948	1823.860913
YLLs (Years of Life Lost)	Global	Both	45-49 years	Prostate cancer	Smoking	Rate	1997	1.364584158	2.091330171	0.611883151
YLLs (Years of Life Lost)	Global	Both	45-49 years	Prostate cancer	Smoking	Number	1998	4155.448745	6434.445316	1866.434391
YLLs (Years of Life Lost)	Global	Both	45-49 years	Prostate cancer	Smoking	Rate	1998	1.346989485	2.085726648	0.605005056
YLLs (Years of Life Lost)	Global	Both	45-49 years	Prostate cancer	Smoking	Number	1999	4170.266675	6473.687785	1839.494043
YLLs (Years of Life Lost)	Global	Both	45-49 years	Prostate cancer	Smoking	Rate	1999	1.315997765	2.042881025	0.580483273

YLLs (Years of Life Lost)	Global	Both	45-49 years	Prostate cancer	Smoking	Number	2000	4285.281607	6704.589629	1904.875454
YLLs (Years of Life Lost)	Global	Both	45-49 years	Prostate cancer	Smoking	Rate	2000	1.302652556	2.038080952	0.579049665
YLLs (Years of Life Lost)	Global	Both	45-49 years	Prostate cancer	Smoking	Number	2001	4295.375353	6729.191518	1913.595605
YLLs (Years of Life Lost)	Global	Both	45-49 years	Prostate cancer	Smoking	Rate	2001	1.275846211	1.998757453	0.568391235
YLLs (Years of Life Lost)	Global	Both	45-49 years	Prostate cancer	Smoking	Number	2002	4317.207909	6738.727533	1940.063955
YLLs (Years of Life Lost)	Global	Both	45-49 years	Prostate cancer	Smoking	Rate	2002	1.257933551	1.963507812	0.565289324
YLLs (Years of Life Lost)	Global	Both	45-49 years	Prostate cancer	Smoking	Number	2003	4314.226612	6657.787393	1978.828149
YLLs (Years of Life Lost)	Global	Both	45-49 years	Prostate cancer	Smoking	Rate	2003	1.240335782	1.914107133	0.568911089
YLLs (Years of Life Lost)	Global	Both	45-49 years	Prostate cancer	Smoking	Number	2004	4287.146574	6673.871879	1965.216988
YLLs (Years of Life Lost)	Global	Both	45-49 years	Prostate cancer	Smoking	Rate	2004	1.220816458	1.900465144	0.559619132
YLLs (Years of Life Lost)	Global	Both	45-49 years	Prostate cancer	Smoking	Number	2005	4239.454301	6529.853363	1951.786058

YLLs (Years of Life Lost)	Global	Both	45-49 years	Prostate cancer	Smoking	Rate	2005	1.19550305	1.841383126	0.55039305 1
YLLs (Years of Life Lost)	Global	Both	45-49 years	Prostate cancer	Smoking	Number	2006	4148.968078	6349.540923	1898.501726
YLLs (Years of Life Lost)	Global	Both	45-49 years	Prostate cancer	Smoking	Rate	2006	1.152131244	1.763210597	0.527196911
YLLs (Years of Life Lost)	Global	Both	45-49 years	Prostate cancer	Smoking	Number	2007	4065.785034	6237.770405	1886.308778
YLLs (Years of Life Lost)	Global	Both	45-49 years	Prostate cancer	Smoking	Rate	2007	1.10220468	1.691014079	0.511364557
YLLs (Years of Life Lost)	Global	Both	45-49 years	Prostate cancer	Smoking	Number	2008	4053.534021	6224.705024	1894.70855
YLLs (Years of Life Lost)	Global	Both	45-49 years	Prostate cancer	Smoking	Rate	2008	1.062095201	1.630979115	0.496446026
YLLs (Years of Life Lost)	Global	Both	45-49 years	Prostate cancer	Smoking	Number	2009	4050.201288	6213.977522	1858.968168
YLLs (Years of Life Lost)	Global	Both	45-49 years	Prostate cancer	Smoking	Rate	2009	1.021796866	1.567680791	0.468986036
YLLs (Years of Life Lost)	Global	Both	45-49 years	Prostate cancer	Smoking	Number	2010	4046.995968	6150.01381	1815.819585
YLLs (Years of Life Lost)	Global	Both	45-49 years	Prostate cancer	Smoking	Rate	2010	0.984901766	1.49670509	0.441908994

YLLs (Years of Life Lost)	Global	Both	45-49 years	Prostate cancer	Smoking	Number	2011	4018.694639	6213.519352	1812.47284
YLLs (Years of Life Lost)	Global	Both	45-49 years	Prostate cancer	Smoking	Rate	2011	0.949191909	1.467596527	0.428095367
YLLs (Years of Life Lost)	Global	Both	45-49 years	Prostate cancer	Smoking	Number	2012	3984.436521	6157.002204	1801.148459
YLLs (Years of Life Lost)	Global	Both	45-49 years	Prostate cancer	Smoking	Rate	2012	0.918023909	1.41858835	0.41498905
YLLs (Years of Life Lost)	Global	Both	45-49 years	Prostate cancer	Smoking	Number	2013	3865.449979	6053.294456	1739.603706
YLLs (Years of Life Lost)	Global	Both	45-49 years	Prostate cancer	Smoking	Rate	2013	0.874963245	1.370192394	0.393767689
YLLs (Years of Life Lost)	Global	Both	45-49 years	Prostate cancer	Smoking	Number	2014	3755.951102	5837.389953	1682.442806
YLLs (Years of Life Lost)	Global	Both	45-49 years	Prostate cancer	Smoking	Rate	2014	0.838815665	1.303662908	0.375739551
YLLs (Years of Life Lost)	Global	Both	45-49 years	Prostate cancer	Smoking	Number	2015	3681.056474	5681.226983	1624.874587
YLLs (Years of Life Lost)	Global	Both	45-49 years	Prostate cancer	Smoking	Rate	2015	0.8127082	1.254308319	0.358741821
YLLs (Years of Life Lost)	Global	Both	45-49 years	Prostate cancer	Smoking	Number	2016	3666.414962	5643.331416	1626.443327

YLLs (Years of Life Lost)	Global	Both	45-49 years	Prostate cancer	Smoking	Rate	2016	0.799461325	1.230527711	0.354645765
YLLs (Years of Life Lost)	Global	Both	45-49 years	Prostate cancer	Smoking	Number	2017	3651.818086	5832.872633	1590.270626
YLLs (Years of Life Lost)	Global	Both	45-49 years	Prostate cancer	Smoking	Rate	2017	0.787656159	1.258085137	0.343003519
YLLs (Years of Life Lost)	Global	Both	45-49 years	Prostate cancer	Smoking	Number	2018	3620.514233	5565.983927	1599.501209
YLLs (Years of Life Lost)	Global	Both	45-49 years	Prostate cancer	Smoking	Rate	2018	0.773677732	1.189410549	0.341801851
YLLs (Years of Life Lost)	Global	Both	45-49 years	Prostate cancer	Smoking	Number	2019	3633.175229	5745.076122	1576.919845
YLLs (Years of Life Lost)	Global	Both	45-49 years	Prostate cancer	Smoking	Rate	2019	0.770997864	1.21916537	0.334638919
YLLs (Years of Life Lost)	Global	Both	45-49 years	Prostate cancer	Smoking	Number	2020	3595.068546	5519.105396	1574.842228
YLLs (Years of Life Lost)	Global	Both	45-49 years	Prostate cancer	Smoking	Rate	2020	0.759919546	1.16661922	0.332887503
YLLs (Years of Life Lost)	Global	Both	45-49 years	Prostate cancer	Smoking	Number	2021	3584.718794	5568.737298	1562.479844
YLLs (Years of Life Lost)	Global	Both	45-49 years	Prostate cancer	Smoking	Rate	2021	0.757060987	1.176068193	0.32998196

YLLs (Years of Life Lost)	Global	Both	50-54 years	Prostate cancer	Smoking	Number	1990	8306.713851	12806.48335	3867.160983
YLLs (Years of Life Lost)	Global	Both	50-54 years	Prostate cancer	Smoking	Rate	1990	3.907727582	6.024554246	1.819228627
YLLs (Years of Life Lost)	Global	Both	50-54 years	Prostate cancer	Smoking	Number	1991	8477.981037	13027.25885	3923.26943
YLLs (Years of Life Lost)	Global	Both	50-54 years	Prostate cancer	Smoking	Rate	1991	3.94543154	6.062546936	1.8257874
YLLs (Years of Life Lost)	Global	Both	50-54 years	Prostate cancer	Smoking	Number	1992	8604.750658	13207.08418	4020.074993
YLLs (Years of Life Lost)	Global	Both	50-54 years	Prostate cancer	Smoking	Rate	1992	3.978036416	6.105727395	1.858508788
YLLs (Years of Life Lost)	Global	Both	50-54 years	Prostate cancer	Smoking	Number	1993	8666.043181	13199.89933	4032.911
YLLs (Years of Life Lost)	Global	Both	50-54 years	Prostate cancer	Smoking	Rate	1993	3.989137091	6.07615343	1.856422189
YLLs (Years of Life Lost)	Global	Both	50-54 years	Prostate cancer	Smoking	Number	1994	8638.83743	13214.52054	4018.0614
YLLs (Years of Life Lost)	Global	Both	50-54 years	Prostate cancer	Smoking	Rate	1994	3.960062052	6.057565239	1.841888171
YLLs (Years of Life Lost)	Global	Both	50-54 years	Prostate cancer	Smoking	Number	1995	8568.135244	13052.70673	3931.892641

YLLs (Years of Life Lost)	Global	Both	50-54 years	Prostate cancer	Smoking	Rate	1995	3.916548737	5.966474691	1.797292961
YLLs (Years of Life Lost)	Global	Both	50-54 years	Prostate cancer	Smoking	Number	1996	8577.584716	13130.10701	3961.994573
YLLs (Years of Life Lost)	Global	Both	50-54 years	Prostate cancer	Smoking	Rate	1996	3.870251699	5.924373891	1.787672956
YLLs (Years of Life Lost)	Global	Both	50-54 years	Prostate cancer	Smoking	Number	1997	8727.970289	13354.45434	3946.970303
YLLs (Years of Life Lost)	Global	Both	50-54 years	Prostate cancer	Smoking	Rate	1997	3.801744883	5.816957069	1.719228372
YLLs (Years of Life Lost)	Global	Both	50-54 years	Prostate cancer	Smoking	Number	1998	8977.372182	13920.98587	4107.892491
YLLs (Years of Life Lost)	Global	Both	50-54 years	Prostate cancer	Smoking	Rate	1998	3.767131557	5.841596417	1.72377519
YLLs (Years of Life Lost)	Global	Both	50-54 years	Prostate cancer	Smoking	Number	1999	9312.667888	14333.46627	4243.601522
YLLs (Years of Life Lost)	Global	Both	50-54 years	Prostate cancer	Smoking	Rate	1999	3.713465814	5.715530462	1.692154104
YLLs (Years of Life Lost)	Global	Both	50-54 years	Prostate cancer	Smoking	Number	2000	9700.358977	15010.44138	4433.388346
YLLs (Years of Life Lost)	Global	Both	50-54 years	Prostate cancer	Smoking	Rate	2000	3.714896588	5.748471536	1.697831934

YLLs (Years of Life Lost)	Global	Both	50-54 years	Prostate cancer	Smoking	Number	2001	10180.34792	15554.7602	4701.743438
YLLs (Years of Life Lost)	Global	Both	50-54 years	Prostate cancer	Smoking	Rate	2001	3.715026079	5.676263745	1.715766459
YLLs (Years of Life Lost)	Global	Both	50-54 years	Prostate cancer	Smoking	Number	2002	10375.81274	15884.90337	4733.122616
YLLs (Years of Life Lost)	Global	Both	50-54 years	Prostate cancer	Smoking	Rate	2002	3.661467833	5.605542828	1.670247588
YLLs (Years of Life Lost)	Global	Both	50-54 years	Prostate cancer	Smoking	Number	2003	10445.79143	15863.38736	4846.351728
YLLs (Years of Life Lost)	Global	Both	50-54 years	Prostate cancer	Smoking	Rate	2003	3.555143271	5.3989796	1.64941784
YLLs (Years of Life Lost)	Global	Both	50-54 years	Prostate cancer	Smoking	Number	2004	10472.67379	16119.29236	4881.510061
YLLs (Years of Life Lost)	Global	Both	50-54 years	Prostate cancer	Smoking	Rate	2004	3.463566502	5.331039827	1.614433436
YLLs (Years of Life Lost)	Global	Both	50-54 years	Prostate cancer	Smoking	Number	2005	10597.8841	16153.28846	4902.648886
YLLs (Years of Life Lost)	Global	Both	50-54 years	Prostate cancer	Smoking	Rate	2005	3.369350661	5.135562216	1.55868314
YLLs (Years of Life Lost)	Global	Both	50-54 years	Prostate cancer	Smoking	Number	2006	10516.07219	15860.47117	4861.074486

YLLs (Years of Life Lost)	Global	Both	50-54 years	Prostate cancer	Smoking	Rate	2006	3.26029934	4.917224108	1.507079606
YLLs (Years of Life Lost)	Global	Both	50-54 years	Prostate cancer	Smoking	Number	2007	10368.09588	15700.94788	4855.615498
YLLs (Years of Life Lost)	Global	Both	50-54 years	Prostate cancer	Smoking	Rate	2007	3.14765969	4.766665095	1.47412074
YLLs (Years of Life Lost)	Global	Both	50-54 years	Prostate cancer	Smoking	Number	2008	10371.39076	15775.98642	4845.353274
YLLs (Years of Life Lost)	Global	Both	50-54 years	Prostate cancer	Smoking	Rate	2008	3.101945456	4.718388356	1.449180917
YLLs (Years of Life Lost)	Global	Both	50-54 years	Prostate cancer	Smoking	Number	2009	10225.29827	15432.57439	4772.685123
YLLs (Years of Life Lost)	Global	Both	50-54 years	Prostate cancer	Smoking	Rate	2009	3.025009314	4.565508023	1.411931131
YLLs (Years of Life Lost)	Global	Both	50-54 years	Prostate cancer	Smoking	Number	2010	10118.52615	15332.31023	4691.642578
YLLs (Years of Life Lost)	Global	Both	50-54 years	Prostate cancer	Smoking	Rate	2010	2.960682616	4.486236799	1.372775483
YLLs (Years of Life Lost)	Global	Both	50-54 years	Prostate cancer	Smoking	Number	2011	10017.16293	15447.18329	4680.001742
YLLs (Years of Life Lost)	Global	Both	50-54 years	Prostate cancer	Smoking	Rate	2011	2.883071287	4.445902587	1.346966076

YLLs (Years of Life Lost)	Global	Both	50-54 years	Prostate cancer	Smoking	Number	2012	9986.28997	15334.34692	4631.071793
YLLs (Years of Life Lost)	Global	Both	50-54 years	Prostate cancer	Smoking	Rate	2012	2.802805581	4.303819862	1.299781391
YLLs (Years of Life Lost)	Global	Both	50-54 years	Prostate cancer	Smoking	Number	2013	9766.994397	15163.78902	4553.145508
YLLs (Years of Life Lost)	Global	Both	50-54 years	Prostate cancer	Smoking	Rate	2013	2.646531152	4.108883285	1.233751237
YLLs (Years of Life Lost)	Global	Both	50-54 years	Prostate cancer	Smoking	Number	2014	9730.228195	14968.71429	4478.063661
YLLs (Years of Life Lost)	Global	Both	50-54 years	Prostate cancer	Smoking	Rate	2014	2.536224038	3.901657005	1.167225729
YLLs (Years of Life Lost)	Global	Both	50-54 years	Prostate cancer	Smoking	Number	2015	9652.572725	14815.41053	4381.638769
YLLs (Years of Life Lost)	Global	Both	50-54 years	Prostate cancer	Smoking	Rate	2015	2.425018795	3.722080113	1.100800447
YLLs (Years of Life Lost)	Global	Both	50-54 years	Prostate cancer	Smoking	Number	2016	9707.529663	14954.21675	4451.079505
YLLs (Years of Life Lost)	Global	Both	50-54 years	Prostate cancer	Smoking	Rate	2016	2.365351221	3.643766857	1.084556701
YLLs (Years of Life Lost)	Global	Both	50-54 years	Prostate cancer	Smoking	Number	2017	9731.141609	15332.02206	4343.411098

YLLs (Years of Life Lost)	Global	Both	50-54 years	Prostate cancer	Smoking	Rate	2017	2.311651304	3.642151169	1.031785615
YLLs (Years of Life Lost)	Global	Both	50-54 years	Prostate cancer	Smoking	Number	2018	9649.396994	14780.99668	4283.310404
YLLs (Years of Life Lost)	Global	Both	50-54 years	Prostate cancer	Smoking	Rate	2018	2.250954393	3.448023687	0.999185376
YLLs (Years of Life Lost)	Global	Both	50-54 years	Prostate cancer	Smoking	Number	2019	9676.223379	15180.87691	4342.850328
YLLs (Years of Life Lost)	Global	Both	50-54 years	Prostate cancer	Smoking	Rate	2019	2.226218	3.492678919	0.999163743
YLLs (Years of Life Lost)	Global	Both	50-54 years	Prostate cancer	Smoking	Number	2020	9647.178592	14862.81877	4229.1197
YLLs (Years of Life Lost)	Global	Both	50-54 years	Prostate cancer	Smoking	Rate	2020	2.194267864	3.380574463	0.961920769
YLLs (Years of Life Lost)	Global	Both	50-54 years	Prostate cancer	Smoking	Number	2021	9699.374785	15166.03737	4403.759954
YLLs (Years of Life Lost)	Global	Both	50-54 years	Prostate cancer	Smoking	Rate	2021	2.180012083	3.408688234	0.989780282
YLLs (Years of Life Lost)	Global	Both	55-59 years	Prostate cancer	Smoking	Number	1990	16013.71622	24547.84294	7372.420595
YLLs (Years of Life Lost)	Global	Both	55-59 years	Prostate cancer	Smoking	Rate	1990	8.646716945	13.25477775	3.980789543

YLLs (Years of Life Lost)	Global	Both	55-59 years	Prostate cancer	Smoking	Number	1991	16028.57782	24703.59986	7314.420201
YLLs (Years of Life Lost)	Global	Both	55-59 years	Prostate cancer	Smoking	Rate	1991	8.533052426	13.15132978	3.8939406 7
YLLs (Years of Life Lost)	Global	Both	55-59 years	Prostate cancer	Smoking	Number	1992	16269.50318	25233.14272	7452.162058
YLLs (Years of Life Lost)	Global	Both	55-59 years	Prostate cancer	Smoking	Rate	1992	8.513432654	13.20388574	3.899533937
YLLs (Years of Life Lost)	Global	Both	55-59 years	Prostate cancer	Smoking	Number	1993	16595.0287	25463.98335	7579.781294
YLLs (Years of Life Lost)	Global	Both	55-59 years	Prostate cancer	Smoking	Rate	1993	8.543303272	13.10913866	3.902154766
YLLs (Years of Life Lost)	Global	Both	55-59 years	Prostate cancer	Smoking	Number	1994	17023.73623	26178.70858	7867.198478
YLLs (Years of Life Lost)	Global	Both	55-59 years	Prostate cancer	Smoking	Rate	1994	8.641325062	13.2884302	3.993425325
YLLs (Years of Life Lost)	Global	Both	55-59 years	Prostate cancer	Smoking	Number	1995	17196.40096	26501.50305	7859.254288
YLLs (Years of Life Lost)	Global	Both	55-59 years	Prostate cancer	Smoking	Rate	1995	8.617771326	13.2809123	3.938571588
YLLs (Years of Life Lost)	Global	Both	55-59 years	Prostate cancer	Smoking	Number	1996	17369.80185	26927.5206	7997.353215

YLLs (Years of Life Lost)	Global	Both	55-59 years	Prostate cancer	Smoking	Rate	1996	8.626847389	13.37376285	3.971947768
YLLs (Years of Life Lost)	Global	Both	55-59 years	Prostate cancer	Smoking	Number	1997	17183.20844	26762.12824	7851.117226
YLLs (Years of Life Lost)	Global	Both	55-59 years	Prostate cancer	Smoking	Rate	1997	8.489269932	13.22168275	3.878801425
YLLs (Years of Life Lost)	Global	Both	55-59 years	Prostate cancer	Smoking	Number	1998	17022.83327	26397.38717	7834.216661
YLLs (Years of Life Lost)	Global	Both	55-59 years	Prostate cancer	Smoking	Rate	1998	8.377526414	12.99106939	3.855489621
YLLs (Years of Life Lost)	Global	Both	55-59 years	Prostate cancer	Smoking	Number	1999	16705.19278	25944.03152	7721.893238
YLLs (Years of Life Lost)	Global	Both	55-59 years	Prostate cancer	Smoking	Rate	1999	8.182201208	12.7073832	3.782182284
YLLs (Years of Life Lost)	Global	Both	55-59 years	Prostate cancer	Smoking	Number	2000	16268.84843	25333.38502	7583.246148
YLLs (Years of Life Lost)	Global	Both	55-59 years	Prostate cancer	Smoking	Rate	2000	7.934182739	12.35488222	3.698286389
YLLs (Years of Life Lost)	Global	Both	55-59 years	Prostate cancer	Smoking	Number	2001	16301.84611	25324.04075	7610.998858
YLLs (Years of Life Lost)	Global	Both	55-59 years	Prostate cancer	Smoking	Rate	2001	7.829421465	12.16258495	3.655396905

YLLs (Years of Life Lost)	Global	Both	55-59 years	Prostate cancer	Smoking	Number	2002	16596.40423	25650.51246	7751.643566
YLLs (Years of Life Lost)	Global	Both	55-59 years	Prostate cancer	Smoking	Rate	2002	7.668001668	11.85125221	3.58147554
YLLs (Years of Life Lost)	Global	Both	55-59 years	Prostate cancer	Smoking	Number	2003	17006.10916	26172.71756	7990.065004
YLLs (Years of Life Lost)	Global	Both	55-59 years	Prostate cancer	Smoking	Rate	2003	7.542996141	11.60881103	3.543963463
YLLs (Years of Life Lost)	Global	Both	55-59 years	Prostate cancer	Smoking	Number	2004	17755.30253	27650.43383	8348.318717
YLLs (Years of Life Lost)	Global	Both	55-59 years	Prostate cancer	Smoking	Rate	2004	7.456661801	11.61230192	3.506028082
YLLs (Years of Life Lost)	Global	Both	55-59 years	Prostate cancer	Smoking	Number	2005	18595.34901	28606.2453	8699.615502
YLLs (Years of Life Lost)	Global	Both	55-59 years	Prostate cancer	Smoking	Rate	2005	7.478455237	11.50451787	3.498707396
YLLs (Years of Life Lost)	Global	Both	55-59 years	Prostate cancer	Smoking	Number	2006	19137.20209	29393.91128	8919.556779
YLLs (Years of Life Lost)	Global	Both	55-59 years	Prostate cancer	Smoking	Rate	2006	7.316106965	11.23722256	3.409925399
YLLs (Years of Life Lost)	Global	Both	55-59 years	Prostate cancer	Smoking	Number	2007	19084.25305	29209.51492	8796.637714

YLLs (Years of Life Lost)	Global	Both	55-59 years	Prostate cancer	Smoking	Rate	2007	7.043223899	10.78004746	3.24648226
YLLs (Years of Life Lost)	Global	Both	55-59 years	Prostate cancer	Smoking	Number	2008	19335.02628	29534.4868	8977.907332
YLLs (Years of Life Lost)	Global	Both	55-59 years	Prostate cancer	Smoking	Rate	2008	6.871228072	10.49588409	3.190543835
YLLs (Years of Life Lost)	Global	Both	55-59 years	Prostate cancer	Smoking	Number	2009	19348.11021	29528.64138	8918.966634
YLLs (Years of Life Lost)	Global	Both	55-59 years	Prostate cancer	Smoking	Rate	2009	6.671975851	10.18261629	3.07560425
YLLs (Years of Life Lost)	Global	Both	55-59 years	Prostate cancer	Smoking	Number	2010	19611.78376	29768.82718	9113.142681
YLLs (Years of Life Lost)	Global	Both	55-59 years	Prostate cancer	Smoking	Rate	2010	6.491222335	9.853059683	3.016321006
YLLs (Years of Life Lost)	Global	Both	55-59 years	Prostate cancer	Smoking	Number	2011	19724.4584	30115.93366	9055.823223
YLLs (Years of Life Lost)	Global	Both	55-59 years	Prostate cancer	Smoking	Rate	2011	6.360159714	9.710895182	2.920053919
YLLs (Years of Life Lost)	Global	Both	55-59 years	Prostate cancer	Smoking	Number	2012	19853.71113	30162.88226	9151.026851
YLLs (Years of Life Lost)	Global	Both	55-59 years	Prostate cancer	Smoking	Rate	2012	6.264426969	9.517272203	2.887416817

YLLs (Years of Life Lost)	Global	Both	55-59 years	Prostate cancer	Smoking	Number	2013	19674.82953	30393.40374	8973.146961
YLLs (Years of Life Lost)	Global	Both	55-59 years	Prostate cancer	Smoking	Rate	2013	6.11294167	9.443187495	2.787944051
YLLs (Years of Life Lost)	Global	Both	55-59 years	Prostate cancer	Smoking	Number	2014	19655.38265	30178.31445	8924.652939
YLLs (Years of Life Lost)	Global	Both	55-59 years	Prostate cancer	Smoking	Rate	2014	6.038431147	9.27123512	2.741788509
YLLs (Years of Life Lost)	Global	Both	55-59 years	Prostate cancer	Smoking	Number	2015	19669.27334	30290.16547	9095.465636
YLLs (Years of Life Lost)	Global	Both	55-59 years	Prostate cancer	Smoking	Rate	2015	5.974723664	9.200917863	2.762831796
YLLs (Years of Life Lost)	Global	Both	55-59 years	Prostate cancer	Smoking	Number	2016	19804.48879	30526.45318	9157.45068
YLLs (Years of Life Lost)	Global	Both	55-59 years	Prostate cancer	Smoking	Rate	2016	5.915054065	9.117408829	2.735077711
YLLs (Years of Life Lost)	Global	Both	55-59 years	Prostate cancer	Smoking	Number	2017	19739.18405	30834.50614	8913.755697
YLLs (Years of Life Lost)	Global	Both	55-59 years	Prostate cancer	Smoking	Rate	2017	5.746995915	8.97736099	2.595209481
YLLs (Years of Life Lost)	Global	Both	55-59 years	Prostate cancer	Smoking	Number	2018	19868.86614	30917.26792	9148.219375

YLLs (Years of Life Lost)	Global	Both	55-59 years	Prostate cancer	Smoking	Rate	2018	5.582341295	8.686491729	2.570276654
YLLs (Years of Life Lost)	Global	Both	55-59 years	Prostate cancer	Smoking	Number	2019	20041.01438	31329.34241	9115.184366
YLLs (Years of Life Lost)	Global	Both	55-59 years	Prostate cancer	Smoking	Rate	2019	5.413677263	8.462992215	2.462283865
YLLs (Years of Life Lost)	Global	Both	55-59 years	Prostate cancer	Smoking	Number	2020	19980.37364	30848.23276	9056.016285
YLLs (Years of Life Lost)	Global	Both	55-59 years	Prostate cancer	Smoking	Rate	2020	5.201758218	8.031133508	2.357673984
YLLs (Years of Life Lost)	Global	Both	55-59 years	Prostate cancer	Smoking	Number	2021	20108.89289	31134.65831	9091.942286
YLLs (Years of Life Lost)	Global	Both	55-59 years	Prostate cancer	Smoking	Rate	2021	5.081493522	7.867691444	2.297523095
YLLs (Years of Life Lost)	Global	Both	60-64 years	Prostate cancer	Smoking	Number	1990	32125.35877	49055.2258	15249.6941
YLLs (Years of Life Lost)	Global	Both	60-64 years	Prostate cancer	Smoking	Rate	1990	20.00218631	30.5432158	9.494904782
YLLs (Years of Life Lost)	Global	Both	60-64 years	Prostate cancer	Smoking	Number	1991	32567.37569	49869.21261	15397.94762
YLLs (Years of Life Lost)	Global	Both	60-64 years	Prostate cancer	Smoking	Rate	1991	19.86847819	30.4238626	9.393872856

YLLs (Years of Life Lost)	Global	Both	60-64 years	Prostate cancer	Smoking	Number	1992	32684.99877	50199.9686	15400.55994
YLLs (Years of Life Lost)	Global	Both	60-64 years	Prostate cancer	Smoking	Rate	1992	19.65447313	30.18675145	9.260820033
YLLs (Years of Life Lost)	Global	Both	60-64 years	Prostate cancer	Smoking	Number	1993	32444.54338	49482.88723	15327.86052
YLLs (Years of Life Lost)	Global	Both	60-64 years	Prostate cancer	Smoking	Rate	1993	19.28286515	29.4093164	9.109854435
YLLs (Years of Life Lost)	Global	Both	60-64 years	Prostate cancer	Smoking	Number	1994	32243.6462	49288.31529	15177.91292
YLLs (Years of Life Lost)	Global	Both	60-64 years	Prostate cancer	Smoking	Rate	1994	18.97638865	29.00770655	8.93267383
YLLs (Years of Life Lost)	Global	Both	60-64 years	Prostate cancer	Smoking	Number	1995	31763.74586	48786.71137	14932.61211
YLLs (Years of Life Lost)	Global	Both	60-64 years	Prostate cancer	Smoking	Rate	1995	18.48979191	28.39892201	8.692327783
YLLs (Years of Life Lost)	Global	Both	60-64 years	Prostate cancer	Smoking	Number	1996	31472.10973	48403.50796	14665.32048
YLLs (Years of Life Lost)	Global	Both	60-64 years	Prostate cancer	Smoking	Rate	1996	18.05626816	27.77019803	8.413829306
YLLs (Years of Life Lost)	Global	Both	60-64 years	Prostate cancer	Smoking	Number	1997	31186.28898	48557.19936	14447.00657

YLLs (Years of Life Lost)	Global	Both	60-64 years	Prostate cancer	Smoking	Rate	1997	17.5689697	27.3549688 6	8.138801663
YLLs (Years of Life Lost)	Global	Both	60-64 years	Prostate cancer	Smoking	Number	1998	31547.69985	49073.98673	14528.75286
YLLs (Years of Life Lost)	Global	Both	60-64 years	Prostate cancer	Smoking	Rate	1998	17.46048771	27.16064075	8.041128574
YLLs (Years of Life Lost)	Global	Both	60-64 years	Prostate cancer	Smoking	Number	1999	31807.97784	49427.29964	14565.80075
YLLs (Years of Life Lost)	Global	Both	60-64 years	Prostate cancer	Smoking	Rate	1999	17.32670727	26.92445136	7.934404603
YLLs (Years of Life Lost)	Global	Both	60-64 years	Prostate cancer	Smoking	Number	2000	32091.30569	49931.66484	14761.25119
YLLs (Years of Life Lost)	Global	Both	60-64 years	Prostate cancer	Smoking	Rate	2000	17.22273324	26.79728123	7.922055086
YLLs (Years of Life Lost)	Global	Both	60-64 years	Prostate cancer	Smoking	Number	2001	32115.03988	49723.29796	14817.83396
YLLs (Years of Life Lost)	Global	Both	60-64 years	Prostate cancer	Smoking	Rate	2001	17.04373141	26.38858735	7.863953555
YLLs (Years of Life Lost)	Global	Both	60-64 years	Prostate cancer	Smoking	Number	2002	31980.05782	49399.39355	14792.68346
YLLs (Years of Life Lost)	Global	Both	60-64 years	Prostate cancer	Smoking	Rate	2002	16.845087	26.02049962	7.791857081

YLLs (Years of Life Lost)	Global	Both	60-64 years	Prostate cancer	Smoking	Number	2003	31283.76506	48046.39954	14529.45562
YLLs (Years of Life Lost)	Global	Both	60-64 years	Prostate cancer	Smoking	Rate	2003	16.36824498	25.13876563	7.602080134
YLLs (Years of Life Lost)	Global	Both	60-64 years	Prostate cancer	Smoking	Number	2004	30431.27388	47366.78968	14081.99621
YLLs (Years of Life Lost)	Global	Both	60-64 years	Prostate cancer	Smoking	Rate	2004	15.79238906	24.58111922	7.307888712
YLLs (Years of Life Lost)	Global	Both	60-64 years	Prostate cancer	Smoking	Number	2005	29501.81745	45744.3704	13659.72026
YLLs (Years of Life Lost)	Global	Both	60-64 years	Prostate cancer	Smoking	Rate	2005	15.18656475	23.54769648	7.031574462
YLLs (Years of Life Lost)	Global	Both	60-64 years	Prostate cancer	Smoking	Number	2006	28912.40012	44731.16359	13331.47244
YLLs (Years of Life Lost)	Global	Both	60-64 years	Prostate cancer	Smoking	Rate	2006	14.60627314	22.59776396	6.734934734
YLLs (Years of Life Lost)	Global	Both	60-64 years	Prostate cancer	Smoking	Number	2007	29280.7697	45018.34752	13565.16827
YLLs (Years of Life Lost)	Global	Both	60-64 years	Prostate cancer	Smoking	Rate	2007	14.17821802	21.79860546	6.568471908
YLLs (Years of Life Lost)	Global	Both	60-64 years	Prostate cancer	Smoking	Number	2008	30219.64398	46553.22105	14072.29931

YLLs (Years of Life Lost)	Global	Both	60-64 years	Prostate cancer	Smoking	Rate	2008	14.0097556	21.58196337	6.523884735
YLLs (Years of Life Lost)	Global	Both	60-64 years	Prostate cancer	Smoking	Number	2009	31422.11377	48234.14164	14593.57675
YLLs (Years of Life Lost)	Global	Both	60-64 years	Prostate cancer	Smoking	Rate	2009	13.7658292	21.13107222	6.393353622
YLLs (Years of Life Lost)	Global	Both	60-64 years	Prostate cancer	Smoking	Number	2010	32776.58055	50546.75969	15232.1558
YLLs (Years of Life Lost)	Global	Both	60-64 years	Prostate cancer	Smoking	Rate	2010	13.73607657	21.18323967	6.383523076
YLLs (Years of Life Lost)	Global	Both	60-64 years	Prostate cancer	Smoking	Number	2011	34071.07963	53221.82937	15826.17124
YLLs (Years of Life Lost)	Global	Both	60-64 years	Prostate cancer	Smoking	Rate	2011	13.55876472	21.17990596	6.298107798
YLLs (Years of Life Lost)	Global	Both	60-64 years	Prostate cancer	Smoking	Number	2012	34415.02213	53280.34446	15885.9022
YLLs (Years of Life Lost)	Global	Both	60-64 years	Prostate cancer	Smoking	Rate	2012	13.21359723	20.45691005	6.099368833
YLLs (Years of Life Lost)	Global	Both	60-64 years	Prostate cancer	Smoking	Number	2013	34558.06792	54064.48474	15827.45177
YLLs (Years of Life Lost)	Global	Both	60-64 years	Prostate cancer	Smoking	Rate	2013	12.77218442	19.98148656	5.84960749

YLLs (Years of Life Lost)	Global	Both	60-64 years	Prostate cancer	Smoking	Number	2014	34870.41872	54537.24018	15980.70042
YLLs (Years of Life Lost)	Global	Both	60-64 years	Prostate cancer	Smoking	Rate	2014	12.50274693	19.55426224	5.729861024
YLLs (Years of Life Lost)	Global	Both	60-64 years	Prostate cancer	Smoking	Number	2015	35440.61988	55470.9021	16304.47473
YLLs (Years of Life Lost)	Global	Both	60-64 years	Prostate cancer	Smoking	Rate	2015	12.1983874	19.09265571	5.611874167
YLLs (Years of Life Lost)	Global	Both	60-64 years	Prostate cancer	Smoking	Number	2016	36003.66006	56230.13186	16738.20539
YLLs (Years of Life Lost)	Global	Both	60-64 years	Prostate cancer	Smoking	Rate	2016	12.07742075	18.86238677	5.614827734
YLLs (Years of Life Lost)	Global	Both	60-64 years	Prostate cancer	Smoking	Number	2017	36589.59465	57110.95946	16643.2802
YLLs (Years of Life Lost)	Global	Both	60-64 years	Prostate cancer	Smoking	Rate	2017	12.01684025	18.75651488	5.466025009
YLLs (Years of Life Lost)	Global	Both	60-64 years	Prostate cancer	Smoking	Number	2018	36994.79694	58096.72805	16867.6656
YLLs (Years of Life Lost)	Global	Both	60-64 years	Prostate cancer	Smoking	Rate	2018	11.97167246	18.80034645	5.458447793
YLLs (Years of Life Lost)	Global	Both	60-64 years	Prostate cancer	Smoking	Number	2019	37435.33083	58410.82861	16981.71551

YLLs (Years of Life Lost)	Global	Both	60-64 years	Prostate cancer	Smoking	Rate	2019	11.9866557	18.70293319	5.437483052
YLLs (Years of Life Lost)	Global	Both	60-64 years	Prostate cancer	Smoking	Number	2020	37323.08447	57883.68641	16748.4674
YLLs (Years of Life Lost)	Global	Both	60-64 years	Prostate cancer	Smoking	Rate	2020	11.83200702	18.35004243	5.309528584
YLLs (Years of Life Lost)	Global	Both	60-64 years	Prostate cancer	Smoking	Number	2021	37438.72208	58016.83515	16979.68572
YLLs (Years of Life Lost)	Global	Both	60-64 years	Prostate cancer	Smoking	Rate	2021	11.69785134	18.12755016	5.305358416
YLLs (Years of Life Lost)	Global	Both	65-69 years	Prostate cancer	Smoking	Number	1990	39584.29352	61671.27995	18861.13189
YLLs (Years of Life Lost)	Global	Both	65-69 years	Prostate cancer	Smoking	Rate	1990	32.02367713	49.89204004	15.25864792
YLLs (Years of Life Lost)	Global	Both	65-69 years	Prostate cancer	Smoking	Number	1991	40301.51892	62847.06049	19209.12917
YLLs (Years of Life Lost)	Global	Both	65-69 years	Prostate cancer	Smoking	Rate	1991	31.62652515	49.31908753	15.07432035
YLLs (Years of Life Lost)	Global	Both	65-69 years	Prostate cancer	Smoking	Number	1992	41216.40122	64016.20083	19603.71431
YLLs (Years of Life Lost)	Global	Both	65-69 years	Prostate cancer	Smoking	Rate	1992	31.39432041	48.76081028	14.93204817

YLLs (Years of Life Lost)	Global	Both	65-69 years	Prostate cancer	Smoking	Number	1993	42165.1656	65733.8340 8	19897.026 92
YLLs (Years of Life Lost)	Global	Both	65-69 years	Prostate cancer	Smoking	Rate	1993	31.200664 24	48.6406078 8	14.723064 58
YLLs (Years of Life Lost)	Global	Both	65-69 years	Prostate cancer	Smoking	Number	1994	42893.723 72	66903.0164 6	20426.032 02
YLLs (Years of Life Lost)	Global	Both	65-69 years	Prostate cancer	Smoking	Rate	1994	30.893376 25	48.1856057 5	14.711455 15
YLLs (Years of Life Lost)	Global	Both	65-69 years	Prostate cancer	Smoking	Number	1995	43084.978 19	66858.9076 7	20190.461 76
YLLs (Years of Life Lost)	Global	Both	65-69 years	Prostate cancer	Smoking	Rate	1995	30.339731 93	47.0809414 5	14.217790 59
YLLs (Years of Life Lost)	Global	Both	65-69 years	Prostate cancer	Smoking	Number	1996	43044.708 15	66870.0710 2	20378.931 16
YLLs (Years of Life Lost)	Global	Both	65-69 years	Prostate cancer	Smoking	Rate	1996	29.713897 65	46.1606207 1	14.067640 39
YLLs (Years of Life Lost)	Global	Both	65-69 years	Prostate cancer	Smoking	Number	1997	42387.718 34	66318.0128 3	20071.119 47
YLLs (Years of Life Lost)	Global	Both	65-69 years	Prostate cancer	Smoking	Rate	1997	28.840088 49	45.1219700 7	13.656145 79
YLLs (Years of Life Lost)	Global	Both	65-69 years	Prostate cancer	Smoking	Number	1998	41541.655 84	64844.0491 2	19694.320 67

YLLs (Years of Life Lost)	Global	Both	65-69 years	Prostate cancer	Smoking	Rate	1998	27.91880909	43.57959719	13.2359186 9
YLLs (Years of Life Lost)	Global	Both	65-69 years	Prostate cancer	Smoking	Number	1999	40571.8172	63125.58103	19138.4952
YLLs (Years of Life Lost)	Global	Both	65-69 years	Prostate cancer	Smoking	Rate	1999	26.95733008	41.94283721	12.7162835 7
YLLs (Years of Life Lost)	Global	Both	65-69 years	Prostate cancer	Smoking	Number	2000	39905.24814	61972.91058	18879.67738
YLLs (Years of Life Lost)	Global	Both	65-69 years	Prostate cancer	Smoking	Rate	2000	26.15774231	40.62301328	12.3755585 7
YLLs (Years of Life Lost)	Global	Both	65-69 years	Prostate cancer	Smoking	Number	2001	39523.93119	61421.46974	18688.10677
YLLs (Years of Life Lost)	Global	Both	65-69 years	Prostate cancer	Smoking	Rate	2001	25.45877095	39.56375498	12.0376747 8
YLLs (Years of Life Lost)	Global	Both	65-69 years	Prostate cancer	Smoking	Number	2002	39599.28761	61016.25608	18695.70761
YLLs (Years of Life Lost)	Global	Both	65-69 years	Prostate cancer	Smoking	Rate	2002	24.97237721	38.47849429	11.7900167 1
YLLs (Years of Life Lost)	Global	Both	65-69 years	Prostate cancer	Smoking	Number	2003	39702.78085	61169.6875	18827.02793
YLLs (Years of Life Lost)	Global	Both	65-69 years	Prostate cancer	Smoking	Rate	2003	24.52875042	37.79120672	11.6315144 5

YLLs (Years of Life Lost)	Global	Both	65-69 years	Prostate cancer	Smoking	Number	2004	39586.42238	61858.90833	18615.95685
YLLs (Years of Life Lost)	Global	Both	65-69 years	Prostate cancer	Smoking	Rate	2004	24.00727981	37.51448178	11.28969122
YLLs (Years of Life Lost)	Global	Both	65-69 years	Prostate cancer	Smoking	Number	2005	39358.01605	61125.28156	18497.58055
YLLs (Years of Life Lost)	Global	Both	65-69 years	Prostate cancer	Smoking	Rate	2005	23.45424006	36.42579509	11.02308344
YLLs (Years of Life Lost)	Global	Both	65-69 years	Prostate cancer	Smoking	Number	2006	38695.93904	60067.1649	18190.05031
YLLs (Years of Life Lost)	Global	Both	65-69 years	Prostate cancer	Smoking	Rate	2006	22.73061586	35.28441705	10.68512766
YLLs (Years of Life Lost)	Global	Both	65-69 years	Prostate cancer	Smoking	Number	2007	37735.02361	58180.21943	17787.84764
YLLs (Years of Life Lost)	Global	Both	65-69 years	Prostate cancer	Smoking	Rate	2007	21.92271994	33.80065877	10.33411311
YLLs (Years of Life Lost)	Global	Both	65-69 years	Prostate cancer	Smoking	Number	2008	37056.34964	57360.53945	17498.11209
YLLs (Years of Life Lost)	Global	Both	65-69 years	Prostate cancer	Smoking	Rate	2008	21.30191093	32.97381189	10.05882201
YLLs (Years of Life Lost)	Global	Both	65-69 years	Prostate cancer	Smoking	Number	2009	36309.37482	56219.27255	17032.10876

YLLs (Years of Life Lost)	Global	Both	65-69 years	Prostate cancer	Smoking	Rate	2009	20.62460124	31.9339037	9.674648858
YLLs (Years of Life Lost)	Global	Both	65-69 years	Prostate cancer	Smoking	Number	2010	35693.38191	55364.47688	16614.52996
YLLs (Years of Life Lost)	Global	Both	65-69 years	Prostate cancer	Smoking	Rate	2010	20.04794199	31.09662804	9.331901741
YLLs (Years of Life Lost)	Global	Both	65-69 years	Prostate cancer	Smoking	Number	2011	35426.34234	55251.87823	16629.90916
YLLs (Years of Life Lost)	Global	Both	65-69 years	Prostate cancer	Smoking	Rate	2011	19.48058479	30.38244504	9.144617655
YLLs (Years of Life Lost)	Global	Both	65-69 years	Prostate cancer	Smoking	Number	2012	36070.32423	55756.07499	16831.95085
YLLs (Years of Life Lost)	Global	Both	65-69 years	Prostate cancer	Smoking	Rate	2012	18.95996893	29.30756714	8.847529706
YLLs (Years of Life Lost)	Global	Both	65-69 years	Prostate cancer	Smoking	Number	2013	36744.67445	57790.56056	17209.33472
YLLs (Years of Life Lost)	Global	Both	65-69 years	Prostate cancer	Smoking	Rate	2013	18.45431441	29.02421073	8.643061297
YLLs (Years of Life Lost)	Global	Both	65-69 years	Prostate cancer	Smoking	Number	2014	38375.0444	60164.50237	17967.66457
YLLs (Years of Life Lost)	Global	Both	65-69 years	Prostate cancer	Smoking	Rate	2014	18.17593671	28.4962846	8.510195598

YLLs (Years of Life Lost)	Global	Both	65-69 years	Prostate cancer	Smoking	Number	2015	39917.08313	62228.11952	18793.00156
YLLs (Years of Life Lost)	Global	Both	65-69 years	Prostate cancer	Smoking	Rate	2015	18.06431346	28.16108214	8.504696341
YLLs (Years of Life Lost)	Global	Both	65-69 years	Prostate cancer	Smoking	Number	2016	41882.65443	65442.00013	19668.21022
YLLs (Years of Life Lost)	Global	Both	65-69 years	Prostate cancer	Smoking	Rate	2016	17.97408499	28.08465911	8.440679964
YLLs (Years of Life Lost)	Global	Both	65-69 years	Prostate cancer	Smoking	Number	2017	43129.86928	67514.16895	19849.62522
YLLs (Years of Life Lost)	Global	Both	65-69 years	Prostate cancer	Smoking	Rate	2017	17.84236772	27.92989288	8.211578616
YLLs (Years of Life Lost)	Global	Both	65-69 years	Prostate cancer	Smoking	Number	2018	44137.36913	69483.03443	20345.63697
YLLs (Years of Life Lost)	Global	Both	65-69 years	Prostate cancer	Smoking	Rate	2018	17.56760538	27.65571563	8.097993346
YLLs (Years of Life Lost)	Global	Both	65-69 years	Prostate cancer	Smoking	Number	2019	44921.70413	70426.99294	20917.86155
YLLs (Years of Life Lost)	Global	Both	65-69 years	Prostate cancer	Smoking	Rate	2019	17.34169851	27.18783051	8.075188942
YLLs (Years of Life Lost)	Global	Both	65-69 years	Prostate cancer	Smoking	Number	2020	45768.97083	71618.76119	21024.37777

YLLs (Years of Life Lost)	Global	Both	65-69 years	Prostate cancer	Smoking	Rate	2020	16.97930717	26.56902533	7.799593517
YLLs (Years of Life Lost)	Global	Both	65-69 years	Prostate cancer	Smoking	Number	2021	46239.37635	71886.71382	21348.34884
YLLs (Years of Life Lost)	Global	Both	65-69 years	Prostate cancer	Smoking	Rate	2021	16.7629838	26.06081471	7.739335045
YLLs (Years of Life Lost)	Global	Both	70-74 years	Prostate cancer	Smoking	Number	1990	34926.27473	55495.28445	16443.06396
YLLs (Years of Life Lost)	Global	Both	70-74 years	Prostate cancer	Smoking	Rate	1990	41.2541914	65.54987911	19.42220615
YLLs (Years of Life Lost)	Global	Both	70-74 years	Prostate cancer	Smoking	Number	1991	36250.3037	57548.68446	17103.42866
YLLs (Years of Life Lost)	Global	Both	70-74 years	Prostate cancer	Smoking	Rate	1991	41.32062924	65.5980119	19.49568313
YLLs (Years of Life Lost)	Global	Both	70-74 years	Prostate cancer	Smoking	Number	1992	37867.66715	60010.39341	17872.02945
YLLs (Years of Life Lost)	Global	Both	70-74 years	Prostate cancer	Smoking	Rate	1992	41.42850406	65.65339283	19.55260253
YLLs (Years of Life Lost)	Global	Both	70-74 years	Prostate cancer	Smoking	Number	1993	39486.99905	62464.60735	18785.36427
YLLs (Years of Life Lost)	Global	Both	70-74 years	Prostate cancer	Smoking	Rate	1993	41.39531733	65.48338201	19.69321888

YLLs (Years of Life Lost)	Global	Both	70-74 years	Prostate cancer	Smoking	Number	1994	40877.98604	64680.37387	19350.95824
YLLs (Years of Life Lost)	Global	Both	70-74 years	Prostate cancer	Smoking	Rate	1994	41.20448309	65.19698325	19.50551651
YLLs (Years of Life Lost)	Global	Both	70-74 years	Prostate cancer	Smoking	Number	1995	41292.43835	65620.62322	19479.0661
YLLs (Years of Life Lost)	Global	Both	70-74 years	Prostate cancer	Smoking	Rate	1995	40.20938784	63.89947397	18.96815386
YLLs (Years of Life Lost)	Global	Both	70-74 years	Prostate cancer	Smoking	Number	1996	41374.06278	65835.75568	19639.20175
YLLs (Years of Life Lost)	Global	Both	70-74 years	Prostate cancer	Smoking	Rate	1996	39.04308852	62.12663354	18.53274832
YLLs (Years of Life Lost)	Global	Both	70-74 years	Prostate cancer	Smoking	Number	1997	41265.00005	65415.04093	19606.81572
YLLs (Years of Life Lost)	Global	Both	70-74 years	Prostate cancer	Smoking	Rate	1997	37.73870356	59.82500509	17.93131723
YLLs (Years of Life Lost)	Global	Both	70-74 years	Prostate cancer	Smoking	Number	1998	41584.22722	66274.51552	19801.43264
YLLs (Years of Life Lost)	Global	Both	70-74 years	Prostate cancer	Smoking	Rate	1998	36.87530558	58.7697109	17.55915471
YLLs (Years of Life Lost)	Global	Both	70-74 years	Prostate cancer	Smoking	Number	1999	41823.34346	66218.94036	19837.93503

YLLs (Years of Life Lost)	Global	Both	70-74 years	Prostate cancer	Smoking	Rate	1999	36.01310819	57.0195892	17.0819844
YLLs (Years of Life Lost)	Global	Both	70-74 years	Prostate cancer	Smoking	Number	2000	41871.90352	66647.27014	19888.87704
YLLs (Years of Life Lost)	Global	Both	70-74 years	Prostate cancer	Smoking	Rate	2000	35.15162963	55.95064849	16.69679142
YLLs (Years of Life Lost)	Global	Both	70-74 years	Prostate cancer	Smoking	Number	2001	42000.57861	66593.41925	19829.45008
YLLs (Years of Life Lost)	Global	Both	70-74 years	Prostate cancer	Smoking	Rate	2001	34.46193538	54.64063083	16.27028126
YLLs (Years of Life Lost)	Global	Both	70-74 years	Prostate cancer	Smoking	Number	2002	41705.3816	66266.40215	19782.8094
YLLs (Years of Life Lost)	Global	Both	70-74 years	Prostate cancer	Smoking	Rate	2002	33.63252392	53.43929897	15.95347615
YLLs (Years of Life Lost)	Global	Both	70-74 years	Prostate cancer	Smoking	Number	2003	41013.12361	64815.25797	19436.85437
YLLs (Years of Life Lost)	Global	Both	70-74 years	Prostate cancer	Smoking	Rate	2003	32.57343963	51.47756881	15.43713686
YLLs (Years of Life Lost)	Global	Both	70-74 years	Prostate cancer	Smoking	Number	2004	40015.21597	63139.96616	18852.10828
YLLs (Years of Life Lost)	Global	Both	70-74 years	Prostate cancer	Smoking	Rate	2004	31.31368753	49.4098338	14.75261381

YLLs (Years of Life Lost)	Global	Both	70-74 years	Prostate cancer	Smoking	Number	2005	39079.13028	61916.1972	18356.89891
YLLs (Years of Life Lost)	Global	Both	70-74 years	Prostate cancer	Smoking	Rate	2005	30.05691442	47.62157773	14.11883365
YLLs (Years of Life Lost)	Global	Both	70-74 years	Prostate cancer	Smoking	Number	2006	38199.89754	60357.64867	17944.02347
YLLs (Years of Life Lost)	Global	Both	70-74 years	Prostate cancer	Smoking	Rate	2006	28.75290373	45.43095068	13.50639171
YLLs (Years of Life Lost)	Global	Both	70-74 years	Prostate cancer	Smoking	Number	2007	38002.65642	59920.12104	17738.43065
YLLs (Years of Life Lost)	Global	Both	70-74 years	Prostate cancer	Smoking	Rate	2007	27.88846727	43.97272432	13.01744902
YLLs (Years of Life Lost)	Global	Both	70-74 years	Prostate cancer	Smoking	Number	2008	38192.56896	60177.65565	17946.40891
YLLs (Years of Life Lost)	Global	Both	70-74 years	Prostate cancer	Smoking	Rate	2008	27.35380173	43.09968421	12.85335144
YLLs (Years of Life Lost)	Global	Both	70-74 years	Prostate cancer	Smoking	Number	2009	38334.8061	60513.80039	18008.93884
YLLs (Years of Life Lost)	Global	Both	70-74 years	Prostate cancer	Smoking	Rate	2009	26.85852323	42.39779664	12.61760659
YLLs (Years of Life Lost)	Global	Both	70-74 years	Prostate cancer	Smoking	Number	2010	38520.63391	60940.78295	17933.91504

YLLs (Years of Life Lost)	Global	Both	70-74 years	Prostate cancer	Smoking	Rate	2010	26.4334405	41.81848521	12.3065232 3
YLLs (Years of Life Lost)	Global	Both	70-74 years	Prostate cancer	Smoking	Number	2011	38371.0035	61230.21003	17824.73447
YLLs (Years of Life Lost)	Global	Both	70-74 years	Prostate cancer	Smoking	Rate	2011	25.87380125	41.28790337	12.01932696
YLLs (Years of Life Lost)	Global	Both	70-74 years	Prostate cancer	Smoking	Number	2012	37769.16761	60019.53959	17592.53159
YLLs (Years of Life Lost)	Global	Both	70-74 years	Prostate cancer	Smoking	Rate	2012	25.12187374	39.92153894	11.7015382
YLLs (Years of Life Lost)	Global	Both	70-74 years	Prostate cancer	Smoking	Number	2013	36930.11369	58321.57036	17175.39213
YLLs (Years of Life Lost)	Global	Both	70-74 years	Prostate cancer	Smoking	Rate	2013	24.23735036	38.276631	11.27226415
YLLs (Years of Life Lost)	Global	Both	70-74 years	Prostate cancer	Smoking	Number	2014	36818.35586	58951.17451	16889.68547
YLLs (Years of Life Lost)	Global	Both	70-74 years	Prostate cancer	Smoking	Rate	2014	23.80499372	38.11501917	10.92006547
YLLs (Years of Life Lost)	Global	Both	70-74 years	Prostate cancer	Smoking	Number	2015	36636.99248	58306.64149	16858.38067
YLLs (Years of Life Lost)	Global	Both	70-74 years	Prostate cancer	Smoking	Rate	2015	23.35623463	37.17072574	10.74728759

YLLs (Years of Life Lost)	Global	Both	70-74 years	Prostate cancer	Smoking	Number	2016	37127.9996	59658.76033	16969.50211
YLLs (Years of Life Lost)	Global	Both	70-74 years	Prostate cancer	Smoking	Rate	2016	23.11411666	37.14069061	10.56440033
YLLs (Years of Life Lost)	Global	Both	70-74 years	Prostate cancer	Smoking	Number	2017	38717.2072	61750.93458	17705.75512
YLLs (Years of Life Lost)	Global	Both	70-74 years	Prostate cancer	Smoking	Rate	2017	22.97677514	36.64616953	10.50750257
YLLs (Years of Life Lost)	Global	Both	70-74 years	Prostate cancer	Smoking	Number	2018	40476.66103	65598.64493	18709.4116
YLLs (Years of Life Lost)	Global	Both	70-74 years	Prostate cancer	Smoking	Rate	2018	22.91270531	37.1335575	10.59087443
YLLs (Years of Life Lost)	Global	Both	70-74 years	Prostate cancer	Smoking	Number	2019	42657.86353	69144.52741	19687.9558
YLLs (Years of Life Lost)	Global	Both	70-74 years	Prostate cancer	Smoking	Rate	2019	22.74242828	36.86341335	10.49635134
YLLs (Years of Life Lost)	Global	Both	70-74 years	Prostate cancer	Smoking	Number	2020	44461.2134	71717.75326	20527.55836
YLLs (Years of Life Lost)	Global	Both	70-74 years	Prostate cancer	Smoking	Rate	2020	22.6789441	36.58206317	10.4707747
YLLs (Years of Life Lost)	Global	Both	70-74 years	Prostate cancer	Smoking	Number	2021	46261.26037	75289.85461	21538.0475

YLLs (Years of Life Lost)	Global	Both	70-74 years	Prostate cancer	Smoking	Rate	2021	22.47446346	36.57702087	10.46352948
YLLs (Years of Life Lost)	Global	Both	75-79 years	Prostate cancer	Smoking	Number	1990	33743.23797	55018.49474	15906.98984
YLLs (Years of Life Lost)	Global	Both	75-79 years	Prostate cancer	Smoking	Rate	1990	54.81757131	89.38028594	25.84169755
YLLs (Years of Life Lost)	Global	Both	75-79 years	Prostate cancer	Smoking	Number	1991	33481.49359	54759.18001	15836.65508
YLLs (Years of Life Lost)	Global	Both	75-79 years	Prostate cancer	Smoking	Rate	1991	54.20226379	88.64812172	25.63752282
YLLs (Years of Life Lost)	Global	Both	75-79 years	Prostate cancer	Smoking	Number	1992	32879.20539	54030.84426	15555.35566
YLLs (Years of Life Lost)	Global	Both	75-79 years	Prostate cancer	Smoking	Rate	1992	53.2440906	87.49673641	25.19010895
YLLs (Years of Life Lost)	Global	Both	75-79 years	Prostate cancer	Smoking	Number	1993	31996.24323	52531.02462	15140.56594
YLLs (Years of Life Lost)	Global	Both	75-79 years	Prostate cancer	Smoking	Rate	1993	51.88964648	85.19176073	24.55408932
YLLs (Years of Life Lost)	Global	Both	75-79 years	Prostate cancer	Smoking	Number	1994	31475.16728	51632.59694	14841.2496
YLLs (Years of Life Lost)	Global	Both	75-79 years	Prostate cancer	Smoking	Rate	1994	50.59841333	83.00281483	23.85829041

YLLs (Years of Life Lost)	Global	Both	75-79 years	Prostate cancer	Smoking	Number	1995	31612.21551	51650.62123	14885.93883
YLLs (Years of Life Lost)	Global	Both	75-79 years	Prostate cancer	Smoking	Rate	1995	49.54410472	80.9492073	23.32992169
YLLs (Years of Life Lost)	Global	Both	75-79 years	Prostate cancer	Smoking	Number	1996	32302.17101	52990.5915	15162.65268
YLLs (Years of Life Lost)	Global	Both	75-79 years	Prostate cancer	Smoking	Rate	1996	48.70031409	79.89117664	22.85994795
YLLs (Years of Life Lost)	Global	Both	75-79 years	Prostate cancer	Smoking	Number	1997	33083.3957	54221.28258	15487.96868
YLLs (Years of Life Lost)	Global	Both	75-79 years	Prostate cancer	Smoking	Rate	1997	47.7033227	78.18228104	22.33227733
YLLs (Years of Life Lost)	Global	Both	75-79 years	Prostate cancer	Smoking	Number	1998	34118.73446	56121.01646	16035.60038
YLLs (Years of Life Lost)	Global	Both	75-79 years	Prostate cancer	Smoking	Rate	1998	46.97040042	77.2603866	22.07580623
YLLs (Years of Life Lost)	Global	Both	75-79 years	Prostate cancer	Smoking	Number	1999	34995.42664	57823.89436	16493.40388
YLLs (Years of Life Lost)	Global	Both	75-79 years	Prostate cancer	Smoking	Rate	1999	46.15264772	76.25927392	21.75182108
YLLs (Years of Life Lost)	Global	Both	75-79 years	Prostate cancer	Smoking	Number	2000	35389.72121	58348.41868	16723.50968

YLLs (Years of Life Lost)	Global	Both	75-79 years	Prostate cancer	Smoking	Rate	2000	44.92192606	74.06453795	21.22797919
YLLs (Years of Life Lost)	Global	Both	75-79 years	Prostate cancer	Smoking	Number	2001	35706.891	58741.77546	16623.31235
YLLs (Years of Life Lost)	Global	Both	75-79 years	Prostate cancer	Smoking	Rate	2001	43.77372551	72.0126083	20.37882021
YLLs (Years of Life Lost)	Global	Both	75-79 years	Prostate cancer	Smoking	Number	2002	35944.31639	59162.40399	16764.39232
YLLs (Years of Life Lost)	Global	Both	75-79 years	Prostate cancer	Smoking	Rate	2002	42.57095599	70.06949497	19.85505024
YLLs (Years of Life Lost)	Global	Both	75-79 years	Prostate cancer	Smoking	Number	2003	36298.22368	59911.59739	16956.7818
YLLs (Years of Life Lost)	Global	Both	75-79 years	Prostate cancer	Smoking	Rate	2003	41.55533263	68.58865547	19.41264989
YLLs (Years of Life Lost)	Global	Both	75-79 years	Prostate cancer	Smoking	Number	2004	36380.3177	60262.57288	16855.57421
YLLs (Years of Life Lost)	Global	Both	75-79 years	Prostate cancer	Smoking	Rate	2004	40.31757226	66.78448102	18.67976626
YLLs (Years of Life Lost)	Global	Both	75-79 years	Prostate cancer	Smoking	Number	2005	36181.90789	59440.00212	16817.4191
YLLs (Years of Life Lost)	Global	Both	75-79 years	Prostate cancer	Smoking	Rate	2005	38.97096797	64.02189807	18.1137795

YLLs (Years of Life Lost)	Global	Both	75-79 years	Prostate cancer	Smoking	Number	2006	35963.95922	59157.17817	16534.02428
YLLs (Years of Life Lost)	Global	Both	75-79 years	Prostate cancer	Smoking	Rate	2006	37.70509489	62.02117521	17.33449175
YLLs (Years of Life Lost)	Global	Both	75-79 years	Prostate cancer	Smoking	Number	2007	35579.06377	58376.21925	16350.38797
YLLs (Years of Life Lost)	Global	Both	75-79 years	Prostate cancer	Smoking	Rate	2007	36.4734726	59.84371729	16.76141428
YLLs (Years of Life Lost)	Global	Both	75-79 years	Prostate cancer	Smoking	Number	2008	35181.83851	58034.62827	16206.17735
YLLs (Years of Life Lost)	Global	Both	75-79 years	Prostate cancer	Smoking	Rate	2008	35.32634486	58.27300049	16.27274282
YLLs (Years of Life Lost)	Global	Both	75-79 years	Prostate cancer	Smoking	Number	2009	34719.06161	57586.2019	15976.09257
YLLs (Years of Life Lost)	Global	Both	75-79 years	Prostate cancer	Smoking	Rate	2009	34.15175325	56.6452452	15.71504372
YLLs (Years of Life Lost)	Global	Both	75-79 years	Prostate cancer	Smoking	Number	2010	34433.72686	56985.33507	15773.41652
YLLs (Years of Life Lost)	Global	Both	75-79 years	Prostate cancer	Smoking	Rate	2010	33.10246314	54.78218961	15.16359066
YLLs (Years of Life Lost)	Global	Both	75-79 years	Prostate cancer	Smoking	Number	2011	34211.76189	56428.98296	15615.55938

YLLs (Years of Life Lost)	Global	Both	75-79 years	Prostate cancer	Smoking	Rate	2011	32.03899267	52.84521088	14.62382424
YLLs (Years of Life Lost)	Global	Both	75-79 years	Prostate cancer	Smoking	Number	2012	34158.1954	55987.80636	15595.05972
YLLs (Years of Life Lost)	Global	Both	75-79 years	Prostate cancer	Smoking	Rate	2012	31.06920669	50.92472561	14.18476966
YLLs (Years of Life Lost)	Global	Both	75-79 years	Prostate cancer	Smoking	Number	2013	34349.60207	56625.36353	15685.06064
YLLs (Years of Life Lost)	Global	Both	75-79 years	Prostate cancer	Smoking	Rate	2013	30.38042213	50.08216525	13.87261379
YLLs (Years of Life Lost)	Global	Both	75-79 years	Prostate cancer	Smoking	Number	2014	34918.43369	56526.88846	15934.92565
YLLs (Years of Life Lost)	Global	Both	75-79 years	Prostate cancer	Smoking	Rate	2014	30.11413434	48.749561	13.74249762
YLLs (Years of Life Lost)	Global	Both	75-79 years	Prostate cancer	Smoking	Number	2015	35394.34906	58350.76227	16280.59132
YLLs (Years of Life Lost)	Global	Both	75-79 years	Prostate cancer	Smoking	Rate	2015	29.79822545	49.12505005	13.7065024
YLLs (Years of Life Lost)	Global	Both	75-79 years	Prostate cancer	Smoking	Number	2016	35935.12052	59727.47686	16631.54839
YLLs (Years of Life Lost)	Global	Both	75-79 years	Prostate cancer	Smoking	Rate	2016	29.61997935	49.23113115	13.70876493

YLLs (Years of Life Lost)	Global	Both	75-79 years	Prostate cancer	Smoking	Number	2017	36040.95699	60242.81898	16266.58846
YLLs (Years of Life Lost)	Global	Both	75-79 years	Prostate cancer	Smoking	Rate	2017	29.20273673	48.81266564	13.18025213
YLLs (Years of Life Lost)	Global	Both	75-79 years	Prostate cancer	Smoking	Number	2018	36133.94988	60679.16078	16583.40199
YLLs (Years of Life Lost)	Global	Both	75-79 years	Prostate cancer	Smoking	Rate	2018	28.79820854	48.36036835	13.21671919
YLLs (Years of Life Lost)	Global	Both	75-79 years	Prostate cancer	Smoking	Number	2019	36238.9783	59596.16719	16463.63218
YLLs (Years of Life Lost)	Global	Both	75-79 years	Prostate cancer	Smoking	Rate	2019	28.38493143	46.67993411	12.8954814
YLLs (Years of Life Lost)	Global	Both	75-79 years	Prostate cancer	Smoking	Number	2020	36150.44801	59370.39151	16720.50187
YLLs (Years of Life Lost)	Global	Both	75-79 years	Prostate cancer	Smoking	Rate	2020	27.94552868	45.89533657	12.92551795
YLLs (Years of Life Lost)	Global	Both	75-79 years	Prostate cancer	Smoking	Number	2021	36383.80317	60582.9324	16593.70122
YLLs (Years of Life Lost)	Global	Both	75-79 years	Prostate cancer	Smoking	Rate	2021	27.58764638	45.93638845	12.58200412

7

EAPC: estimated annual percentage change, ASR: age-standardized rate, YLDs: Years Lived with Disability, YLLs: Years of Life Lost, DALYs: disability-adjusted-life-years.

Table S3. The EAPC of smoking-related prostate cancer-related ASRs of deaths, YLDs, YLLs and DALYs for different SDI regions between 1990 and 2021.

	location	measure	sex	cause	age	EAPC	LCI	UCI	EAPC_95CI
1	High-middle SDI	Deaths	Both	Prostate cancer	All ages	0.403135778	0.280854676	0.525565989	0.4 (0.28,0.53)
2	High-middle SDI	Deaths	Both	Prostate cancer	Age-standardized	-1.427051039	-1.585136672	-1.268711471	-1.43 (-1.59,-1.27)
3	High-middle SDI	DALYs (Disability-Adjusted Life Years)	Both	Prostate cancer	All ages	0.344373261	0.222194958	0.466700508	0.34 (0.22,0.47)
4	High-middle SDI	DALYs (Disability-Adjusted Life Years)	Both	Prostate cancer	Age-standardized	-1.283034128	-1.441420297	-1.124393428	-1.28 (-1.44,-1.12)
5	High-middle SDI	YLDs (Years Lived with Disability)	Both	Prostate cancer	All ages	2.1504994	1.993799955	2.307439593	2.15 (1.99,2.31)
6	High-middle SDI	YLDs (Years Lived with Disability)	Both	Prostate cancer	Age-standardized	0.570487012	0.362777591	0.778626305	0.57 (0.36,0.78)
7	High-middle SDI	YLLs (Years of Life Lost)	Both	Prostate cancer	All ages	0.169610009	0.044904243	0.294471221	0.17 (0.04,0.29)
8	High-middle SDI	YLLs (Years of Life Lost)	Both	Prostate cancer	Age-standardized	-1.459065977	-1.619023228	-1.298848652	-1.46 (-1.62,-1.3)
9	High SDI	Deaths	Both	Prostate cancer	All ages	-1.65495452	-1.82830148	-1.481301472	-1.65 (-1.83,-1.48)
10	High SDI	Deaths	Both	Prostate cancer	Age-standardized	-3.165673449	-3.308541108	-3.022594694	-3.17 (-3.31,-3.02)
11	High SDI	DALYs (Disability-Adjusted Life Years)	Both	Prostate cancer	All ages	-1.613694656	-1.750071353	-1.47712866	-1.61 (-1.75,-1.48)
12	High	DALYs	Both	Prostate	Age-	-2.908	-3.020	-2.796	-2.91

2	SDI	(Disability-Adjusted Life Years)		cancer	standardized	810077	549015	942395	(-3.02,-2.8)
13	High SDI	YLDs (Years Lived with Disability)	Both	Prostate cancer	All ages	-0.155067411	-0.270252166	-0.039749621	-0.16 (-0.27,-0.04)
14	High SDI	YLDs (Years Lived with Disability)	Both	Prostate cancer	Age-standardized	-1.3317335	-1.485826087	-1.177399886	-1.33 (-1.49,-1.18)
15	High SDI	YLLs (Years of Life Lost)	Both	Prostate cancer	All ages	-1.892831357	-2.049809404	-1.735601731	-1.89 (-2.05,-1.74)
16	High SDI	YLLs (Years of Life Lost)	Both	Prostate cancer	Age-standardized	-3.220631372	-3.347634012	-3.093461848	-3.22 (-3.35,-3.09)
17	Low-middle SDI	Deaths	Both	Prostate cancer	All ages	1.076311916	1.010870941	1.141795287	1.08 (1.01,1.14)
18	Low-middle SDI	Deaths	Both	Prostate cancer	Age-standardized	-0.314426624	-0.394851822	-0.233936488	-0.31 (-0.39,-0.23)
19	Low-middle SDI	DALYs (Disability-Adjusted Life Years)	Both	Prostate cancer	All ages	0.966916099	0.888971682	1.044920734	0.97 (0.89,1.04)
20	Low-middle SDI	DALYs (Disability-Adjusted Life Years)	Both	Prostate cancer	Age-standardized	-0.319674986	-0.384593124	-0.254714542	-0.32 (-0.38,-0.25)
21	Low-middle SDI	YLDs (Years Lived with Disability)	Both	Prostate cancer	All ages	2.612167606	2.537997867	2.686390996	2.61 (2.54,2.69)
22	Low-middle SDI	YLDs (Years Lived with Disability)	Both	Prostate cancer	Age-standardized	1.259619325	1.186415934	1.332875675	1.26 (1.19,1.33)
23	Low-middle SDI	YLLs (Years of Life Lost)	Both	Prostate cancer	All ages	0.903138292	0.825855288	0.980480534	0.9 (0.83,0.98)
24	Low-middle SDI	YLLs (Years of Life Lost)	Both	Prostate cancer	Age-standardiz	-0.378937964	-0.44421385	-0.313619279	-0.38 (-0.44,-0.31)

					ed				
25	Low SDI	Deaths	Both	Prostate cancer	All ages	-0.620665034	-0.779655295	-0.461420007	-0.62 (-0.78,-0.46)
26	Low SDI	Deaths	Both	Prostate cancer	Age-standardized	-0.661195611	-0.777250313	-0.545005168	-0.66 (-0.78,-0.55)
27	Low SDI	DALYs (Disability-Adjusted Life Years)	Both	Prostate cancer	All ages	-0.672084699	-0.849204161	-0.494648837	-0.67 (-0.85,-0.49)
28	Low SDI	DALYs (Disability-Adjusted Life Years)	Both	Prostate cancer	Age-standardized	-0.667348832	-0.781424749	-0.553141757	-0.67 (-0.78,-0.55)
29	Low SDI	YLDs (Years Lived with Disability)	Both	Prostate cancer	All ages	0.35300135	0.154246645	0.552150481	0.35 (0.15,0.55)
30	Low SDI	YLDs (Years Lived with Disability)	Both	Prostate cancer	Age-standardized	0.294757854	0.17292412	0.416739767	0.29 (0.17,0.42)
31	Low SDI	YLLs (Years of Life Lost)	Both	Prostate cancer	All ages	-0.699238771	-0.875503183	-0.522660924	-0.7 (-0.88,-0.52)
32	Low SDI	YLLs (Years of Life Lost)	Both	Prostate cancer	Age-standardized	-0.692292116	-0.806105306	-0.578348339	-0.69 (-0.81,-0.58)
33	Middle SDI	Deaths	Both	Prostate cancer	All ages	1.344144276	1.264309243	1.42404225	1.34 (1.26,1.42)
34	Middle SDI	Deaths	Both	Prostate cancer	Age-standardized	-1.056023127	-1.170901425	-0.941011294	-1.06 (-1.17,-0.94)
35	Middle SDI	DALYs (Disability-Adjusted Life Years)	Both	Prostate cancer	All ages	1.142206777	1.052147481	1.232346336	1.14 (1.05,1.23)
36	Middle SDI	DALYs (Disability-Adjusted Life Years)	Both	Prostate cancer	Age-standardized	-1.027486839	-1.128153873	-0.926717311	-1.03 (-1.13,-0.93)
3	Middle	YLDs (Years Lived	Both	Prostate	All	3.1375	3.0303	3.2448	3.14

7	SDI	with Disability)		cancer	ages	3957	17048	73678	(3.03,3.24)
38	Middle SDI	YLDs (Years Lived with Disability)	Both	Prostate cancer	Age-standardized	0.924220003	0.804243576	1.044339225	0.92 (0.8,1.04)
39	Middle SDI	YLLs (Years of Life Lost)	Both	Prostate cancer	All ages	1.027996649	0.939986466	1.116083569	1.03 (0.94,1.12)
40	Middle SDI	YLLs (Years of Life Lost)	Both	Prostate cancer	Age-standardized	-1.135066422	-1.236553809	-1.03347475	-1.14 (-1.24,-1.03)

8

EAPC: estimated annual percentage change, ASR: age-standardized rate, YLDs: Years Lived with Disability, YLLs: Years of Life Lost, DALYs: disability-adjusted-life-years.

Table S4. The EAPC of smoking-related prostate cancer-related ASRs of deaths, YLDs, YLLs and DALYs for different GBD regions between 1990 and 2021.

	location	measure	sex	cause	age	EAPC	LCI	UCI	EAPC_95CI
1	East Asia	Deaths	Both	Prostate cancer	All ages	2.672847562	2.540636704	2.805228886	2.67 (2.54,2.81)
2	East Asia	Deaths	Both	Prostate cancer	Age-standardized	-0.2228541 42	-0.345724006	-0.099832784	-0.22 (-0.35,-0.1)
3	East Asia	DALYs (Disability-Adjusted Life Years)	Both	Prostate cancer	All ages	2.228908824	2.071242489	2.386818702	2.23 (2.07,2.39)
4	East Asia	DALYs (Disability-Adjusted Life Years)	Both	Prostate cancer	Age-standardized	-0.342701053	-0.447772361	-0.237518849	-0.34 (-0.45,-0.24)
5	East Asia	YLDs (Years Lived with Disability)	Both	Prostate cancer	All ages	5.630549809	5.4613143	5.800056894	5.63 (5.46,5.8)
6	East Asia	YLDs (Years Lived with Disability)	Both	Prostate cancer	Age-standardized	2.951274903	2.83259792	3.070088849	2.95 (2.83,3.07)
7	East Asia	YLLs (Years of Life Lost)	Both	Prostate cancer	All ages	2.027239911	1.873954168	2.180756296	2.03 (1.87,2.18)
8	East Asia	YLLs (Years of Life Lost)	Both	Prostate cancer	Age-standardized	-0.529252922	-0.636134011	-0.422256867	-0.53 (-0.64,-0.42)
9	Southeast Asia	Deaths	Both	Prostate cancer	All ages	1.804809629	1.765272446	1.844362174	1.8 (1.77,1.84)
10	Southeast Asia	Deaths	Both	Prostate cancer	Age-standardiz	-0.126027118	-0.202111216	-0.0498850	-0.13 (-0.2,-0.05)

					ed			15	
11	Southeast Asia	DALYs (Disability-Adjusted Life Years)	Both	Prostate cancer	All ages	1.843345045	1.794324905	1.892388791	1.84 (1.79,1.89)
12	Southeast Asia	DALYs (Disability-Adjusted Life Years)	Both	Prostate cancer	Age-standardized	-0.030055222	-0.085892826	0.025813588	-0.03 (-0.09,0.03)
13	Southeast Asia	YLDs (Years Lived with Disability)	Both	Prostate cancer	All ages	3.464960132	3.387331818	3.542646733	3.46 (3.39,3.54)
14	Southeast Asia	YLDs (Years Lived with Disability)	Both	Prostate cancer	Age-standardized	1.550792406	1.511185128	1.590415137	1.55 (1.51,1.59)
15	Southeast Asia	YLLs (Years of Life Lost)	Both	Prostate cancer	All ages	1.777016673	1.730319611	1.82373517	1.78 (1.73,1.82)
16	Southeast Asia	YLLs (Years of Life Lost)	Both	Prostate cancer	Age-standardized	-0.093117654	-0.15121474	-0.034986763	-0.09 (-0.15,-0.03)
17	Oceania	Deaths	Both	Prostate cancer	All ages	0.695979735	0.564382876	0.827748799	0.7 (0.56,0.83)
18	Oceania	Deaths	Both	Prostate cancer	Age-standardized	0.015543742	-0.065123298	0.096275896	0.02 (-0.07,0.1)
19	Oceania	DALYs (Disability-Adjusted Life Years)	Both	Prostate cancer	All ages	0.751641169	0.588973016	0.914572382	0.75 (0.59,0.91)
20	Oceania	DALYs (Disability-Adjusted Life Years)	Both	Prostate cancer	Age-standardized	0.113192591	0.044020921	0.182412088	0.11 (0.04,0.18)
21	Oceania	YLDs (Years Lived with Disability)	Both	Prostate cancer	All ages	1.201884298	1.075548394	1.328378111	1.2 (1.08,1.33)

22	Oceania	YLDs (Years Lived with Disability)	Both	Prostate cancer	Age-standardized	0.597553163	0.551820925	0.6433062	0.6 (0.55,0.64)
23	Oceania	YLLs (Years of Life Lost)	Both	Prostate cancer	All ages	0.736769559	0.572726564	0.901080122	0.74 (0.57,0.9)
24	Oceania	YLLs (Years of Life Lost)	Both	Prostate cancer	Age-standardized	0.097574766	0.026706808	0.168492933	0.1 (0.03,0.17)
25	Central Asia	Deaths	Both	Prostate cancer	All ages	2.108642112	1.825157026	2.392916432	2.11 (1.83,2.39)
26	Central Asia	Deaths	Both	Prostate cancer	Age-standardized	1.776701794	1.456168273	2.098247988	1.78 (1.46,2.1)
27	Central Asia	DALYs (Disability-Adjusted Life Years)	Both	Prostate cancer	All ages	1.754502714	1.522891788	1.986642029	1.75 (1.52,1.99)
28	Central Asia	DALYs (Disability-Adjusted Life Years)	Both	Prostate cancer	Age-standardized	1.327499119	1.048658943	1.607108745	1.33 (1.05,1.61)
29	Central Asia	YLDs (Years Lived with Disability)	Both	Prostate cancer	All ages	3.066435721	2.778014436	3.355666389	3.07 (2.78,3.36)
30	Central Asia	YLDs (Years Lived with Disability)	Both	Prostate cancer	Age-standardized	2.601242378	2.278043013	2.925463055	2.6 (2.28,2.93)
31	Central Asia	YLLs (Years of Life Lost)	Both	Prostate cancer	All ages	1.691752061	1.461722567	1.922303067	1.69 (1.46,1.92)
32	Central Asia	YLLs (Years of Life Lost)	Both	Prostate cancer	Age-standardized	1.267294171	0.989428868	1.545924002	1.27 (0.99,1.55)

33	Central Europe	Deaths	Both	Prostate cancer	All ages	0.65444393	0.428956178	0.880437959	0.65 (0.43,0.88)
34	Central Europe	Deaths	Both	Prostate cancer	Age-standardized	-1.1681787 21	-1.403253908	-0.932543065	-1.17 (-1.4,-0.93)
35	Central Europe	DALYs (Disability-Adjusted Life Years)	Both	Prostate cancer	All ages	0.495436315	0.278185667	0.713157632	0.5 (0.28,0.71)
36	Central Europe	DALYs (Disability-Adjusted Life Years)	Both	Prostate cancer	Age-standardized	-1.063366057	-1.286971876	-0.839253723	-1.06 (-1.29,-0.84)
37	Central Europe	YLDs (Years Lived with Disability)	Both	Prostate cancer	All ages	2.864585178	2.576458777	3.153520894	2.86 (2.58,3.15)
38	Central Europe	YLDs (Years Lived with Disability)	Both	Prostate cancer	Age-standardized	1.388782263	1.088282527	1.690175279	1.39 (1.09,1.69)
39	Central Europe	YLLs (Years of Life Lost)	Both	Prostate cancer	All ages	0.327454491	0.107721205	0.547670084	0.33 (0.11,0.55)
40	Central Europe	YLLs (Years of Life Lost)	Both	Prostate cancer	Age-standardized	-1.236744448	-1.462969809	-1.00999971	-1.24 (-1.46,-1.01)
41	Australasia	Deaths	Both	Prostate cancer	All ages	-2.814844385	-3.17899801	-2.449321142	-2.81 (-3.18,-2.45)
42	Australasia	Deaths	Both	Prostate cancer	Age-standardized	-4.222660191	-4.598048927	-3.84579437	-4.22 (-4.6,-3.85)
43	Australasia	DALYs (Disability-Adjusted Life Years)	Both	Prostate cancer	All ages	-2.830235093	-3.282446318	-2.375909515	-2.83 (-3.28,-2.38)

44	Australasia	DALYs (Disability-Adjusted Life Years)	Both	Prostate cancer	Age-standardized	-4.042985487	-4.521482221	-3.562090735	-4.04 (-4.52,-3.56)
45	Australasia	YLDs (Years Lived with Disability)	Both	Prostate cancer	All ages	-0.660199053	-1.419696729	0.105150064	-0.66 (-1.42,0.11)
46	Australasia	YLDs (Years Lived with Disability)	Both	Prostate cancer	Age-standardized	-1.754671484	-2.55364962	-0.949142398	-1.75 (-2.55,-0.95)
47	Australasia	YLLs (Years of Life Lost)	Both	Prostate cancer	All ages	-3.287888387	-3.689182735	-2.884921982	-3.29 (-3.69,-2.88)
48	Australasia	YLLs (Years of Life Lost)	Both	Prostate cancer	Age-standardized	-4.541336774	-4.966340229	-4.114432645	-4.54 (-4.97,-4.11)
49	Eastern Europe	Deaths	Both	Prostate cancer	All ages	2.689607395	2.352648612	3.027675493	2.69 (2.35,3.03)
50	Eastern Europe	Deaths	Both	Prostate cancer	Age-standardized	1.69866754	1.365972052	2.032454974	1.7 (1.37,2.03)
51	Eastern Europe	DALYs (Disability-Adjusted Life Years)	Both	Prostate cancer	All ages	2.568190287	2.250744887	2.886621221	2.57 (2.25,2.89)
52	Eastern Europe	DALYs (Disability-Adjusted Life Years)	Both	Prostate cancer	Age-standardized	1.677503279	1.329630393	2.026570442	1.68 (1.33,2.03)
53	Eastern Europe	YLDs (Years Lived with Disability)	Both	Prostate cancer	All ages	4.658315664	4.469531054	4.847441422	4.66 (4.47,4.85)
54	Eastern Europe	YLDs (Years Lived with Disability)	Both	Prostate cancer	Age-standardized	3.789999424	3.533861344	4.046771178	3.79 (3.53,4.05)

55	Eastern Europe	YLLs (Years of Life Lost)	Both	Prostate cancer	All ages	2.374840705	2.036014657	2.714791876	2.37 (2.04,2.71)
56	Eastern Europe	YLLs (Years of Life Lost)	Both	Prostate cancer	Age-standardized	1.483230223	1.116680541	1.851108653	1.48 (1.12,1.85)
57	High-income Asia Pacific	Deaths	Both	Prostate cancer	All ages	1.369719152	1.063422662	1.676943946	1.37 (1.06,1.68)
58	High-income Asia Pacific	Deaths	Both	Prostate cancer	Age-standardized	-1.954160859	-2.220924679	-1.686669246	-1.95 (-2.22,-1.69)
59	High-income Asia Pacific	DALYs (Disability-Adjusted Life Years)	Both	Prostate cancer	All ages	0.944827199	0.601272619	1.289555022	0.94 (0.6,1.29)
60	High-income Asia Pacific	DALYs (Disability-Adjusted Life Years)	Both	Prostate cancer	Age-standardized	-1.77181428	-2.053983979	-1.488831688	-1.77 (-2.05,-1.49)
61	High-income Asia Pacific	YLDs (Years Lived with Disability)	Both	Prostate cancer	All ages	3.750973545	3.079601599	4.426718231	3.75 (3.08,4.43)
62	High-income Asia Pacific	YLDs (Years Lived with Disability)	Both	Prostate cancer	Age-standardized	1.104517701	0.544105153	1.668053874	1.1 (0.54,1.67)
63	High-income Asia Pacific	YLLs (Years of Life Lost)	Both	Prostate cancer	All ages	0.568915629	0.247283902	0.891579274	0.57 (0.25,0.89)
64	High-income Asia Pacific	YLLs (Years of Life Lost)	Both	Prostate cancer	Age-standardized	-2.164080189	-2.435314703	-1.892091629	-2.16 (-2.44,-1.89)
65	Western Europe	Deaths	Both	Prostate cancer	All ages	-1.836683998	-2.004996053	-1.668082859	-1.84 (-2,-1.67)

66	Western Europe	Deaths	Both	Prostate cancer	Age-standardized	-3.1704514 07	-3.335079 219	-3.0055432 2	-3.17 (-3.34,-3.01)
67	Western Europe	DALYs (Disability-Adjusted Life Years)	Both	Prostate cancer	All ages	-1.8512132 24	-2.006509 859	-1.6956704 8	-1.85 (-2.01,-1.7)
68	Western Europe	DALYs (Disability-Adjusted Life Years)	Both	Prostate cancer	Age-standardized	-2.8776174 57	-3.033939 436	-2.7210434 66	-2.88 (-3.03,-2.72)
69	Western Europe	YLDs (Years Lived with Disability)	Both	Prostate cancer	All ages	0.32286424 1	-0.029826 234	0.67679899 3	0.32 (-0.03,0.68)
70	Western Europe	YLDs (Years Lived with Disability)	Both	Prostate cancer	Age-standardized	-0.4681952 14	-0.849153 858	-0.0857728 45	-0.47 (-0.85,-0.09)
71	Western Europe	YLLs (Years of Life Lost)	Both	Prostate cancer	All ages	-2.1785003 68	-2.339069 065	-2.0176676 72	-2.18 (-2.34,-2.02)
72	Western Europe	YLLs (Years of Life Lost)	Both	Prostate cancer	Age-standardized	-3.2626953 31	-3.418317 498	-3.1068224 1	-3.26 (-3.42,-3.11)
73	Southern Latin America	Deaths	Both	Prostate cancer	All ages	-0.3050849 84	-0.650991 925	0.04202631 4	-0.31 (-0.65,0.04)
74	Southern Latin America	Deaths	Both	Prostate cancer	Age-standardized	-1.4334243 81	-1.798651 833	-1.0668385 86	-1.43 (-1.8,-1.07)
75	Southern Latin America	DALYs (Disability-Adjusted Life Years)	Both	Prostate cancer	All ages	-0.5204911 35	-0.844404 637	-0.1955194 98	-0.52 (-0.84,-0.2)
76	Southern Latin America	DALYs (Disability-Adjusted Life Years)	Both	Prostate cancer	Age-standardized	-1.5142729 37	-1.883516 103	-1.1436401 93	-1.51 (-1.88,-1.14)

77	Southern Latin America	YLDs (Years Lived with Disability)	Both	Prostate cancer	All ages	1.460396895	1.022873055	1.899815625	1.46 (1.02,1.9)
78	Southern Latin America	YLDs (Years Lived with Disability)	Both	Prostate cancer	Age-standardized	0.514846301	0.02145309	1.010673358	0.51 (0.02,1.01)
79	Southern Latin America	YLLs (Years of Life Lost)	Both	Prostate cancer	All ages	-0.643560486	-0.964422514	-0.321658907	-0.64 (-0.96,-0.32)
80	Southern Latin America	YLLs (Years of Life Lost)	Both	Prostate cancer	Age-standardized	-1.640829399	-2.006571733	-1.273721999	-1.64 (-2.01,-1.27)
81	High-income North America	Deaths	Both	Prostate cancer	All ages	-2.487482798	-2.829075536	-2.144689232	-2.49 (-2.83,-2.14)
82	High-income North America	Deaths	Both	Prostate cancer	Age-standardized	-3.570693192	-3.806908167	-3.333898159	-3.57 (-3.81,-3.33)
83	High-income North America	DALYs (Disability-Adjusted Life Years)	Both	Prostate cancer	All ages	-2.245848091	-2.580719209	-1.909825888	-2.25 (-2.58,-1.91)
84	High-income North America	DALYs (Disability-Adjusted Life Years)	Both	Prostate cancer	Age-standardized	-3.384375636	-3.601384206	-3.166878545	-3.38 (-3.6,-3.17)
85	High-income North America	YLDs (Years Lived with Disability)	Both	Prostate cancer	All ages	-1.217441279	-1.442821299	-0.991545861	-1.22 (-1.44,-0.99)
86	High-income North America	YLDs (Years Lived with Disability)	Both	Prostate cancer	Age-standardized	-2.413883964	-2.570054088	-2.257463516	-2.41 (-2.57,-2.26)
87	High-income North America	YLLs (Years of Life Lost)	Both	Prostate cancer	All ages	-2.530561015	-2.895274187	-2.164478025	-2.53 (-2.9,-2.16)

88	High-income North America	YLLs (Years of Life Lost)	Both	Prostate cancer	Age-standardized	-3.660400444	-3.905820062	-3.414354036	-3.66 (-3.91,-3.41)
89	Caribbean	Deaths	Both	Prostate cancer	All ages	0.899083966	0.736489946	1.061940421	0.9 (0.74,1.06)
90	Caribbean	Deaths	Both	Prostate cancer	Age-standardized	-0.726191104	-0.911541599	-0.5404939	-0.73 (-0.91,-0.54)
91	Caribbean	DALYs (Disability-Adjusted Life Years)	Both	Prostate cancer	All ages	0.960828959	0.806626155	1.115267645	0.96 (0.81,1.12)
92	Caribbean	DALYs (Disability-Adjusted Life Years)	Both	Prostate cancer	Age-standardized	-0.554576144	-0.744733902	-0.364054065	-0.55 (-0.74,-0.36)
93	Caribbean	YLDs (Years Lived with Disability)	Both	Prostate cancer	All ages	2.066912818	1.881429775	2.252733547	2.07 (1.88,2.25)
94	Caribbean	YLDs (Years Lived with Disability)	Both	Prostate cancer	Age-standardized	0.590162197	0.360949844	0.819898044	0.59 (0.36,0.82)
95	Caribbean	YLLs (Years of Life Lost)	Both	Prostate cancer	All ages	0.863177922	0.709703008	1.016886722	0.86 (0.71,1.02)
96	Caribbean	YLLs (Years of Life Lost)	Both	Prostate cancer	Age-standardized	-0.655090261	-0.843954847	-0.465865939	-0.66 (-0.84,-0.47)
97	Andean Latin America	Deaths	Both	Prostate cancer	All ages	1.248659026	1.155221629	1.342182732	1.25 (1.16,1.34)
98	Andean Latin America	Deaths	Both	Prostate cancer	Age-standardized	-0.943079845	-1.05468606	-0.831347743	-0.94 (-1.05,-0.83)

99	Andean Latin America	DALYs (Disability-Adjusted Life Years)	Both	Prostate cancer	All ages	1.095611559	0.992709934	1.198618031	1.1 (0.99,1.2)
100	Andean Latin America	DALYs (Disability-Adjusted Life Years)	Both	Prostate cancer	Age-standardized	-0.9233176 12	-1.041733724	-0.804759799	-0.92 (-1.04,-0.8)
101	Andean Latin America	YLDs (Years Lived with Disability)	Both	Prostate cancer	All ages	3.437899485	3.2666389	3.609444094	3.44 (3.27,3.61)
102	Andean Latin America	YLDs (Years Lived with Disability)	Both	Prostate cancer	Age-standardized	1.42120134	1.237198188	1.605538926	1.42 (1.24,1.61)
103	Andean Latin America	YLLs (Years of Life Lost)	Both	Prostate cancer	All ages	0.98190065	0.879307166	1.08459847	0.98 (0.88,1.08)
104	Andean Latin America	YLLs (Years of Life Lost)	Both	Prostate cancer	Age-standardized	-1.035977408	-1.152814422	-0.919002293	-1.04 (-1.15,-0.92)
105	Central Latin America	Deaths	Both	Prostate cancer	All ages	0.105858985	-0.13221525	0.344500764	0.11 (-0.13,0.34)
106	Central Latin America	Deaths	Both	Prostate cancer	Age-standardized	-2.440446141	-2.725755506	-2.154299951	-2.44 (-2.73,-2.15)
107	Central Latin America	DALYs (Disability-Adjusted Life Years)	Both	Prostate cancer	All ages	0.124920868	-0.095101397	0.345427691	0.12 (-0.1,0.35)
108	Central Latin America	DALYs (Disability-Adjusted Life Years)	Both	Prostate cancer	Age-standardized	-2.267006 67	-2.538112657	-1.995146558	-2.27 (-2.54,-2)
109	Central Latin America	YLDs (Years Lived with Disability)	Both	Prostate cancer	All ages	1.983107296	1.63202393	2.335403463	1.98 (1.63,2.34)
1	Central	YLDs (Years	Both	Prostate	Age-	-0.39	-0.80	0.01	-0.39

10	Latin America	Lived with Disability)		cancer	standardized	464462	0319511	2689269	(-0.8,0.01)
111	Central Latin America	YLLs (Years of Life Lost)	Both	Prostate cancer	All ages	-0.060695617	-0.273853382	0.152917758	-0.06 (-0.27,0.15)
112	Central Latin America	YLLs (Years of Life Lost)	Both	Prostate cancer	Age-standardized	-2.449577476	-2.713965149	-2.184471296	-2.45 (-2.71,-2.18)
113	Tropical Latin America	Deaths	Both	Prostate cancer	All ages	0.761636699	0.648911382	0.874488266	0.76 (0.65,0.87)
114	Tropical Latin America	Deaths	Both	Prostate cancer	Age-standardized	-1.57960951	-1.714111629	-1.444923328	-1.58 (-1.71,-1.44)
115	Tropical Latin America	DALYs (Disability-Adjusted Life Years)	Both	Prostate cancer	All ages	0.399583717	0.266272821	0.53307186	0.4 (0.27,0.53)
116	Tropical Latin America	DALYs (Disability-Adjusted Life Years)	Both	Prostate cancer	Age-standardized	-1.768368715	-1.923763671	-1.612727547	-1.77 (-1.92,-1.61)
117	Tropical Latin America	YLDs (Years Lived with Disability)	Both	Prostate cancer	All ages	1.745572218	1.518210406	1.973443233	1.75 (1.52,1.97)
118	Tropical Latin America	YLDs (Years Lived with Disability)	Both	Prostate cancer	Age-standardized	-0.421283569	-0.669359526	-0.172588049	-0.42 (-0.67,-0.17)
119	Tropical Latin America	YLLs (Years of Life Lost)	Both	Prostate cancer	All ages	0.324688022	0.19576158	0.453780359	0.32 (0.2,0.45)
120	Tropical Latin America	YLLs (Years of Life Lost)	Both	Prostate cancer	Age-standardized	-1.841926513	-1.993815488	-1.689802143	-1.84 (-1.99,-1.69)
1	North	Deaths	Both	Prostate	All	0.460	0.37	0.54	0.46

21	Africa and Middle East			cancer	ages	02909	0349547	978876	(0.37,0.55)
122	North Africa and Middle East	Deaths	Both	Prostate cancer	Age-standardized	-0.888524281	-0.983841604	-0.793115201	-0.89 (-0.98,-0.79)
123	North Africa and Middle East	DALYs (Disability-Adjusted Life Years)	Both	Prostate cancer	All ages	0.483659063	0.379320721	0.588105858	0.48 (0.38,0.59)
124	North Africa and Middle East	DALYs (Disability-Adjusted Life Years)	Both	Prostate cancer	Age-standardized	-0.757037753	-0.837404224	-0.676606149	-0.76 (-0.84,-0.68)
125	North Africa and Middle East	YLDs (Years Lived with Disability)	Both	Prostate cancer	All ages	3.332178751	3.264780475	3.399621017	3.33 (3.26,3.4)
126	North Africa and Middle East	YLDs (Years Lived with Disability)	Both	Prostate cancer	Age-standardized	2.094577741	2.013435271	2.175784752	2.09 (2.01,2.18)
127	North Africa and Middle East	YLLs (Years of Life Lost)	Both	Prostate cancer	All ages	0.239076412	0.135605722	0.342654019	0.24 (0.14,0.34)
128	North Africa and Middle East	YLLs (Years of Life Lost)	Both	Prostate cancer	Age-standardized	-0.984346232	-1.069213552	-0.899406109	-0.98 (-1.07,-0.9)
129	South Asia	Deaths	Both	Prostate cancer	All ages	0.563186085	0.367388155	0.759365980	0.56 (0.37,0.76)
130	South Asia	Deaths	Both	Prostate cancer	Age-standardized	-1.164005692	-1.345021927	-0.982657320	-1.16 (-1.35,-0.98)
131	South Asia	DALYs (Disability-Adjusted Life Years)	Both	Prostate cancer	All ages	0.324498328	0.121702672	0.527704745	0.32 (0.12,0.53)
13	South Asia	DALYs (Disability-Adj	Both	Prostate cancer	Age-stand	-1.2355553	-1.407547	-1.0632	-1.24 (-1.41,-1.0

2		usted Life Years)			ardized	98	396	63365	6)
133	South Asia	YLDs (Years Lived with Disability)	Both	Prostate cancer	All ages	1.9439732	1.691779402	2.196792434	1.94 (1.69,2.2)
134	South Asia	YLDs (Years Lived with Disability)	Both	Prostate cancer	Age-standardized	0.277415526	0.07839311	0.476833731	0.28 (0.08,0.48)
135	South Asia	YLLs (Years of Life Lost)	Both	Prostate cancer	All ages	0.276369717	0.075678282	0.477463618	0.28 (0.08,0.48)
136	South Asia	YLLs (Years of Life Lost)	Both	Prostate cancer	Age-standardized	-1.279169256	-1.450332918	-1.107708312	-1.28 (-1.45,-1.11)
137	Central Sub-Saharan Africa	Deaths	Both	Prostate cancer	All ages	-0.18281481	-0.572730613	0.208630094	-0.18 (-0.57,0.21)
138	Central Sub-Saharan Africa	Deaths	Both	Prostate cancer	Age-standardized	-0.062970345	-0.405804656	0.281044109	-0.06 (-0.41,0.28)
139	Central Sub-Saharan Africa	DALYs (Disability-Adjusted Life Years)	Both	Prostate cancer	All ages	-0.094253956	-0.507828904	0.321040166	-0.09 (-0.51,0.32)
140	Central Sub-Saharan Africa	DALYs (Disability-Adjusted Life Years)	Both	Prostate cancer	Age-standardized	0.072211435	-0.256272708	0.401777368	0.07 (-0.26,0.4)
141	Central Sub-Saharan Africa	YLDs (Years Lived with Disability)	Both	Prostate cancer	All ages	0.925403536	0.420113384	1.433236188	0.93 (0.42,1.43)
142	Central Sub-Saharan Africa	YLDs (Years Lived with Disability)	Both	Prostate cancer	Age-standardized	1.015703947	0.607778401	1.425283473	1.02 (0.61,1.43)
14	Central Sub-Sahar	YLLs (Years of Life Lost)	Both	Prostate cancer	All ages	-0.1187605	-0.529813	0.29399	-0.12 (-0.53,0.2

3	an Africa					4	34	0903	9)
144	Central Sub-Saharan Africa	YLLs (Years of Life Lost)	Both	Prostate cancer	Age-standardized	0.049993862	-0.27637476	0.3774306	0.05 (-0.28,0.38)
145	Eastern Sub-Saharan Africa	Deaths	Both	Prostate cancer	All ages	-0.988177644	-1.266818749	-0.708750161	-0.99 (-1.27,-0.71)
146	Eastern Sub-Saharan Africa	Deaths	Both	Prostate cancer	Age-standardized	-1.072639808	-1.236014797	-0.908994564	-1.07 (-1.24,-0.91)
147	Eastern Sub-Saharan Africa	DALYs (Disability-Adjusted Life Years)	Both	Prostate cancer	All ages	-0.917428577	-1.202746343	-0.631286839	-0.92 (-1.2,-0.63)
148	Eastern Sub-Saharan Africa	DALYs (Disability-Adjusted Life Years)	Both	Prostate cancer	Age-standardized	-0.954379925	-1.116914444	-0.791578247	-0.95 (-1.12,-0.79)
149	Eastern Sub-Saharan Africa	YLDs (Years Lived with Disability)	Both	Prostate cancer	All ages	0.018880541	-0.297844757	0.336611985	0.02 (-0.3,0.34)
150	Eastern Sub-Saharan Africa	YLDs (Years Lived with Disability)	Both	Prostate cancer	Age-standardized	-0.066514585	-0.258676436	0.126017485	-0.07 (-0.26,0.13)
151	Eastern Sub-Saharan Africa	YLLs (Years of Life Lost)	Both	Prostate cancer	All ages	-0.941477898	-1.225653096	-0.656485124	-0.94 (-1.23,-0.66)
152	Eastern Sub-Saharan Africa	YLLs (Years of Life Lost)	Both	Prostate cancer	Age-standardized	-0.976831478	-1.138367724	-0.815031288	-0.98 (-1.14,-0.82)
153	Western Sub-Saharan Africa	Deaths	Both	Prostate cancer	All ages	0.162126016	0.095313023	0.228983607	0.16 (0.1,0.23)
15	Western Sub-Sahar	Deaths	Both	Prostate cancer	Age-stand	0.85068230	0.748728	0.95273	0.85 (0.75,0.95

4	an Africa				ardiz ed	1	138	963 9	)
155	Western Sub-Saharan Africa	DALYs (Disability-Adjusted Life Years)	Both	Prostate cancer	All ages	0.12942053	0.056497036	0.202397173	0.13 (0.06,0.2)
156	Western Sub-Saharan Africa	DALYs (Disability-Adjusted Life Years)	Both	Prostate cancer	Age-standardized	0.763998503	0.633830899	0.894334476	0.76 (0.63,0.89)
157	Western Sub-Saharan Africa	YLDs (Years Lived with Disability)	Both	Prostate cancer	All ages	1.250696822	1.200190034	1.301228817	1.25 (1.2,1.3)
158	Western Sub-Saharan Africa	YLDs (Years Lived with Disability)	Both	Prostate cancer	Age-standardized	1.810936642	1.69225991	1.929751872	1.81 (1.69,1.93)
159	Western Sub-Saharan Africa	YLLs (Years of Life Lost)	Both	Prostate cancer	All ages	0.100332797	0.026299141	0.174421249	0.1 (0.03,0.17)
160	Western Sub-Saharan Africa	YLLs (Years of Life Lost)	Both	Prostate cancer	Age-standardized	0.737341881	0.606401159	0.868453025	0.74 (0.61,0.87)
161	Southern Sub-Saharan Africa	Deaths	Both	Prostate cancer	All ages	-0.5580182 38	-0.633270896	-0.482708589	-0.56 (-0.63,-0.48)
162	Southern Sub-Saharan Africa	Deaths	Both	Prostate cancer	Age-standardized	-1.770715287	-1.833188898	-1.708201916	-1.77 (-1.83,-1.71)
163	Southern Sub-Saharan Africa	DALYs (Disability-Adjusted Life Years)	Both	Prostate cancer	All ages	-0.145126817	-0.219496499	-0.070701705	-0.15 (-0.22,-0.07)
164	Southern Sub-Saharan Africa	DALYs (Disability-Adjusted Life Years)	Both	Prostate cancer	Age-standardized	-1.373055738	-1.427861329	-1.318219675	-1.37 (-1.43,-1.32)
16	Southern Sub-Sahar	YLDs (Years Lived with	Both	Prostate cancer	All ages	0.94726818	0.804558	1.09017	0.95 (0.8,1.09)

5	an Africa	Disability)				9	893	951 9	
1 6 6	Southern Sub-Saharan Africa	YLDs (Years Lived with Disability)	Both	Prostate cancer	Age-standardized	-0.34270791	-0.418629999	-0.266727937	-0.34 (-0.42,-0.27)
1 6 7	Southern Sub-Saharan Africa	YLLs (Years of Life Lost)	Both	Prostate cancer	All ages	-0.1811402 44	-0.253172023	-0.109056448	-0.18 (-0.25,-0.11)
1 6 8	Southern Sub-Saharan Africa	YLLs (Years of Life Lost)	Both	Prostate cancer	Age-standardized	-1.4062231 14	-1.462203612	-1.350210814	-1.41 (-1.46,-1.35)

9

[9] EAPC: estimated annual percentage change, ASR: age-standardized rate, YLDs: Years Lived with Disability, YLLs: Years of Life Lost, DALYs: disability-adjusted-life-years.

Table S5. Ten year predictions of smoking related prostate cancer burden: the ARIMA models results and confidence intervals from 2022 to 2031.

measure	year	observed	fitted	resid
Deaths	1990	0.279	0.279	0
Deaths	1991	0.279	0.279	0
Deaths	1992	0.278	0.278	0
Deaths	1993	0.276	0.277	-0.001
Deaths	1994	0.274	0.275	0
Deaths	1995	0.27	0.273	-0.003
Deaths	1996	0.265	0.266	0
Deaths	1997	0.259	0.261	-0.002
Deaths	1998	0.253	0.252	0.001
Deaths	1999	0.247	0.248	-0.001
Deaths	2000	0.242	0.242	0
Deaths	2001	0.237	0.236	0.001
Deaths	2002	0.232	0.233	-0.001
Deaths	2003	0.226	0.227	-0.001
Deaths	2004	0.219	0.221	-0.001
Deaths	2005	0.213	0.213	0
Deaths	2006	0.206	0.206	0
Deaths	2007	0.199	0.199	0.001
Deaths	2008	0.195	0.194	0.001
Deaths	2009	0.189	0.19	-0.001
Deaths	2010	0.184	0.184	0.001
Deaths	2011	0.18	0.18	0
Deaths	2012	0.175	0.176	-0.001
Deaths	2013	0.17	0.17	0
Deaths	2014	0.167	0.164	0.003
Deaths	2015	0.166	0.165	0.001
Deaths	2016	0.165	0.165	0.001
Deaths	2017	0.162	0.165	-0.002
Deaths	2018	0.161	0.16	0.001
Deaths	2019	0.16	0.159	0
Deaths	2020	0.157	0.158	-0.001
Deaths	2021	0.155	0.155	0
DALYs	1990	5.726	5.72	0.006
DALYs	1991	5.709	5.681	0.028
DALYs	1992	5.697	5.684	0.013
DALYs	1993	5.669	5.676	-0.007

measure	year	observed	fitted	resid
DALYs	1994	5.637	5.634	0.003
DALYs	1995	5.555	5.599	-0.044
DALYs	1996	5.467	5.476	-0.01
DALYs	1997	5.329	5.381	-0.053
DALYs	1998	5.225	5.203	0.023
DALYs	1999	5.109	5.128	-0.019
DALYs	2000	4.999	5.001	-0.001
DALYs	2001	4.912	4.897	0.016
DALYs	2002	4.817	4.829	-0.011
DALYs	2003	4.707	4.727	-0.021
DALYs	2004	4.581	4.603	-0.022
DALYs	2005	4.462	4.466	-0.004
DALYs	2006	4.325	4.351	-0.025
DALYs	2007	4.199	4.201	-0.002
DALYs	2008	4.104	4.082	0.022
DALYs	2009	3.993	4.013	-0.02
DALYs	2010	3.907	3.89	0.017
DALYs	2011	3.816	3.825	-0.009
DALYs	2012	3.71	3.728	-0.018
DALYs	2013	3.593	3.612	-0.018
DALYs	2014	3.538	3.484	0.054
DALYs	2015	3.497	3.481	0.015
DALYs	2016	3.474	3.451	0.023
DALYs	2017	3.426	3.444	-0.018
DALYs	2018	3.388	3.375	0.014
DALYs	2019	3.359	3.346	0.013
DALYs	2020	3.304	3.324	-0.019
DALYs	2021	3.262	3.248	0.014
YLDs	1990	0.506	0.506	0
YLDs	1991	0.513	0.513	-0.001
YLDs	1992	0.52	0.519	0.001
YLDs	1993	0.528	0.528	0
YLDs	1994	0.535	0.535	0
YLDs	1995	0.54	0.542	-0.003
YLDs	1996	0.541	0.544	-0.004
YLDs	1997	0.53	0.542	-0.011
YLDs	1998	0.523	0.52	0.004
YLDs	1999	0.515	0.517	-0.002
YLDs	2000	0.505	0.506	-0.001

measure	year	observed	fitted	resid
YLDs	2001	0.502	0.496	0.006
YLDs	2002	0.5	0.499	0.001
YLDs	2003	0.496	0.498	-0.002
YLDs	2004	0.492	0.492	-0.001
YLDs	2005	0.493	0.487	0.006
YLDs	2006	0.489	0.494	-0.005
YLDs	2007	0.482	0.485	-0.003
YLDs	2008	0.476	0.475	0.001
YLDs	2009	0.466	0.469	-0.003
YLDs	2010	0.459	0.457	0.002
YLDs	2011	0.448	0.452	-0.004
YLDs	2012	0.435	0.436	-0.002
YLDs	2013	0.42	0.422	-0.001
YLDs	2014	0.416	0.406	0.009
YLDs	2015	0.412	0.411	0.001
YLDs	2016	0.41	0.408	0.002
YLDs	2017	0.406	0.408	-0.003
YLDs	2018	0.403	0.402	0.002
YLDs	2019	0.401	0.401	0
YLDs	2020	0.394	0.398	-0.004
YLDs	2021	0.391	0.387	0.004
YLLs	1990	5.22	5.215	0.005
YLLs	1991	5.197	5.175	0.022
YLLs	1992	5.177	5.167	0.01
YLLs	1993	5.141	5.15	-0.009
YLLs	1994	5.102	5.101	0.001
YLLs	1995	5.016	5.059	-0.043
YLLs	1996	4.926	4.933	-0.007
YLLs	1997	4.799	4.84	-0.041
YLLs	1998	4.702	4.681	0.021
YLLs	1999	4.594	4.61	-0.016
YLLs	2000	4.494	4.493	0.001
YLLs	2001	4.41	4.399	0.011
YLLs	2002	4.317	4.33	-0.012
YLLs	2003	4.21	4.229	-0.018
YLLs	2004	4.09	4.11	-0.02
YLLs	2005	3.969	3.977	-0.009
YLLs	2006	3.836	3.856	-0.02
YLLs	2007	3.717	3.715	0.002

measure	year	observed	fitted	resid
YLLs	2008	3.628	3.606	0.022
YLLs	2009	3.527	3.543	-0.016
YLLs	2010	3.449	3.432	0.017
YLLs	2011	3.368	3.372	-0.004
YLLs	2012	3.276	3.29	-0.014
YLLs	2013	3.173	3.188	-0.015
YLLs	2014	3.123	3.076	0.047
YLLs	2015	3.085	3.07	0.015
YLLs	2016	3.064	3.043	0.021
YLLs	2017	3.02	3.036	-0.016
YLLs	2018	2.985	2.973	0.012
YLLs	2019	2.959	2.945	0.013
YLLs	2020	2.911	2.926	-0.015
YLLs	2021	2.871	2.86	0.01

measure	year	mean	low	up
Deaths	2022	0.152	0.15	0.154
Deaths	2023	0.15	0.145	0.155
Deaths	2024	0.148	0.14	0.156
Deaths	2025	0.146	0.134	0.157
Deaths	2026	0.144	0.129	0.159
Deaths	2027	0.142	0.123	0.162
Deaths	2028	0.14	0.117	0.164
Deaths	2029	0.139	0.11	0.167
Deaths	2030	0.137	0.104	0.17
Deaths	2031	0.136	0.098	0.173
DALYs	2022	3.215	3.169	3.26
DALYs	2023	3.164	3.07	3.259
DALYs	2024	3.111	2.963	3.259
DALYs	2025	3.055	2.851	3.26
DALYs	2026	2.998	2.737	3.259
DALYs	2027	2.938	2.621	3.256
DALYs	2028	2.878	2.505	3.25
DALYs	2029	2.816	2.389	3.243
DALYs	2030	2.753	2.274	3.233
DALYs	2031	2.69	2.159	3.221
YLDs	2022	0.388	0.381	0.396
YLDs	2023	0.386	0.369	0.403
YLDs	2024	0.383	0.354	0.412
YLDs	2025	0.38	0.338	0.423

measure	year	observed	fitted	resid
YLDs	2026	0.378	0.321	0.435
YLDs	2027	0.375	0.302	0.448
YLDs	2028	0.372	0.281	0.463
YLDs	2029	0.37	0.26	0.48
YLDs	2030	0.367	0.237	0.497
YLDs	2031	0.365	0.214	0.515
YLLs	2022	2.827	2.787	2.866
YLLs	2023	2.78	2.697	2.862
YLLs	2024	2.73	2.599	2.861
YLLs	2025	2.678	2.497	2.859
YLLs	2026	2.624	2.392	2.857
YLLs	2027	2.569	2.285	2.852
YLLs	2028	2.512	2.178	2.847
YLLs	2029	2.454	2.07	2.839
YLLs	2030	2.395	1.962	2.829
YLLs	2031	2.336	1.855	2.817

10

EAPC: estimated annual percentage change, ASR: age-standardized rate, YLDs: Years Lived with Disability, YLLs: Years of Life Lost, DALYs: disability-adjusted-life-years, ARIMA: Autoregressive Integrated Moving Average.

Table S6. The ARIMA models parameters and statistical validation: fitting results of smoking related prostate cancer burden indicators between 1990 and 2021.

measure	vb
Deaths	Use the auto. arima() function in the forecast package for model filtering, and optimize the model selection to (1,1,0) (AIC=-326.827, BIC=-323.959, AICC=-326.399), The observed values and fitted values have good consistency (cor=1, P=<0001), The residuals were tested for normal distribution using Q-Q, ACF, and PACF plots, Ljung Box test confirms that the residual of the model is white noise (Q=1.949, P=0.856)
DALYs	Use the auto. arima() function in the forecast package for model filtering, and optimize the model selection to (1,1,0) (AIC=-140.627, BIC=-136.325, AICC=-139.738), The observed values and fitted values have good consistency (cor=1, P=<0001), The residuals were tested for normal distribution using Q-Q, ACF, and PACF plots, Ljung Box test confirms that the residual of the model is white noise (Q=2.813, P=0.729)
YLDs	Use the auto. arima() function in the forecast package for model filtering, and optimize the model selection to (0,2,0) (AIC=-245.334, BIC=-243.933, AICC=-245.192), The observed values and fitted values have good consistency (cor=0.997, P=<0001), The residuals were tested for normal distribution using Q-Q, ACF, and PACF plots, Ljung Box test confirms that the residual of the model is white noise (Q=2.708, P=0.845)
YLLs	Use the auto. arima() function in the forecast package for model filtering, and optimize the model selection to (1,1,0) (AIC=-149.006, BIC=-144.704, AICC=-148.117), The observed values and fitted values have good consistency (cor=1, P=<0001), The residuals were tested for normal distribution using Q-Q, ACF, and PACF plots, Ljung Box test confirms that the residual of the model is white noise (Q=2.954, P=0.707)

11

EAPC: estimated annual percentage change, ASR: age-standardized rate, YLDs: Years Lived with Disability, YLLs: Years of Life Lost, DALYs: disability-adjusted-life-years, ARIMA: Autoregressive Integrated Moving Average.

© 2025 Cai Q. et al.
